# Regio- and Enantioselective Asymmetric Transfer Hydrogenation of One Carbonyl Group in a Diketone through Steric Hindrance

**DOI:** 10.1021/acs.joc.3c01950

**Published:** 2024-02-03

**Authors:** Noha Khamis, Ye Zheng, Marianna N. Diamantakis, Guy J. Clarkson, Jie Liu, Martin Wills

**Affiliations:** † Department of Chemistry, The University of Warwick, Coventry CV4 7AL, U.K.; ‡ Department of Chemistry, Faculty of Science, University of Alexandria, Alexandria, Egypt; § Department of Physics, The University of Warwick, Coventry CV4 7AL, U.K.

## Abstract

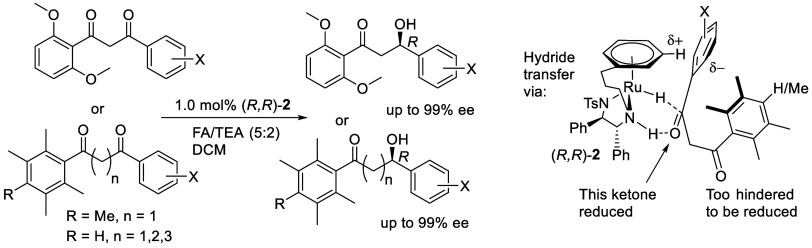

On the basis of steric hindrance, one carbonyl group in a diketone can be reduced in a regioselective manner, with high enantioselectivity. The methodology can be extended to ketones with varied length of hydrocarbon chain spacing, and the products can be converted by oxidation to hydroxy esters or lactones without loss of enantiopurity.

The asymmetric transfer hydrogenation (ATH) of ketones using ruthenium-based catalysts such as **1** and its tethered variants such as **2** or **3** (Figure 1A) has been widely applied in synthetic chemistry.^1^ Acetophenone and its derivatives are known to be excellent substrates and give reduction products for which the major product enantiomer arises through the transition state model illustrated in Figure 1B.^1−3^ Several classes of ketone have been shown to be highly compatible with ATH reduction using Ru-based catalysts such as **1**–**3**.^4,5^

**Figure 1:**
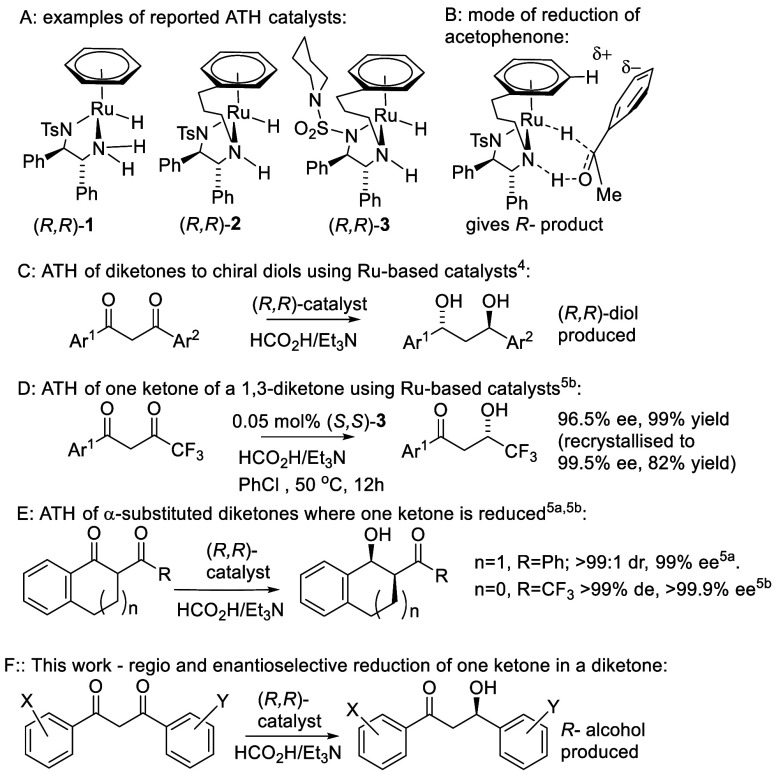
(A) Examples of Ru-based ATH catalysts, (B) mode of hydrogen transfer, (C–E) known precedents, (F) work reported here. In all cases, the descriptor ‘(*R,R*)-’ refers to the configuration of the ligand in the complex.

Ikariya et al.^4a,4b^ reported the first ATH of 1,2-diketones using catalyst **1**, in a reaction which generated 1,2-diols in >99% ee and 98.6:1/4 dr. Reductions of symmetrical and unsymmetrical diketones were reported. In later examples, an extended series of diketones were reduced by ATH,^4c^ and other Ru-based ATH catalysts have been successfully applied (Figure 1C).^4d,4e^ Although in the majority of diketone reductions, both ketones are reduced, sometimes just one ketone can be reduced (Figure 1D, 1E).^5^ In an important precedent,^5b^ an unsymmetrical diketone was reduced, under carefully controlled reaction conditions, to a 3-hydroxy ketone (Figure 1D). In this case the reactive ketone was adjacent to a trifluoromethyl group. Catalyst **1** was applied to the successful reduction of just one ketone of a diketone in high ee, on the basis of differing levels of steric hindrance.^5a^ In other cases of selective keto reduction,^5b,5e^ a substituted carbon atom is generally found between the carbonyl groups (Figure 1E). Herein we report a systematic study of substrates containing two ketones in which one is resistant to ATH due to a high level of steric hindrance from an adjacent aromatic ring. The less hindered ketone is reduced in high enantioselectivity, creating hydroxyketone products with a unique structure and which may form the basis for the synthesis of unusual target molecules.

We first aimed to establish which aromatic groups might present sufficient steric hindrance to prevent the ATH of an adjacent ketone. There are examples of ketones which are resistant to ATH due to steric hindrance;^6^ however, we initially tested ketones **4**–**7** using catalyst (*R,R*)-**2** in formic acid/triethylamine 5:2 azeotrope (FA:TEA) and DCM at rt (Figure 2), which represents a catalyst/reductant system adopted for ATH reactions.^2^ The *diortho*-hydroxy ketone **4** was completely converted to the corresponding alcohol with 73% ee in 24 h (*R* configuration tentatively assigned by analogy with acetophenone).

**Figure 2:**
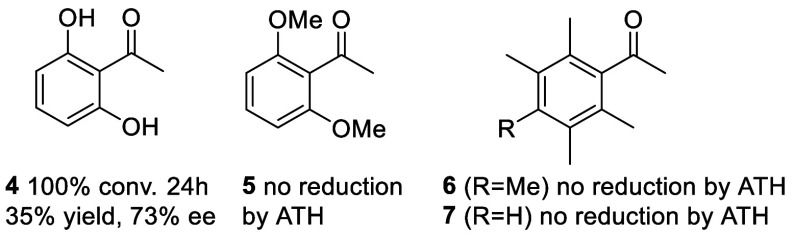
ATH and attempted ATH of ketones **4**–**7** using catalyst (*R,R*)-**2** in FA:TEA (5:2 azeotrope)/DCM at rt.

In contrast, the attempted ATH of ketone **5**, synthesized via O,O′-dimethylation of **4**, yielded no alcohol even after 7 days. In the ATH of a 1:1 mixture of ketone **5** and acetophenone under the same conditions, only acetophenone was reduced, thus ruling out catalyst inhibition by **5** and confirming that it was likely too hindered for reduction. Ketones **6** and **7**, prepared by acetylation of the penta- and tetramethylbenzene respectively, also provide resistance to ATH under the same conditions, even after 7 days. Considering these results, ketones **5**–**7** formed the basis of diketones in which one ketone was designed to be resistant to ATH, providing a potentially valuable element for directing selectivity.

Toward this end, a series of 1,3-diketones **8a**–**24a** were prepared by deprotonation of **5**–**7** with NaH to generate an enolate, followed by addition of the requisite ester (Figure 3, Supporting Information). The products, **8b**–**24b**, from the ATH of the diketones, using 1.0 mol % catalyst (*R,R*)-**2** in FA/TEA/DCM, are shown in Figure 4.

**Figure 3:**
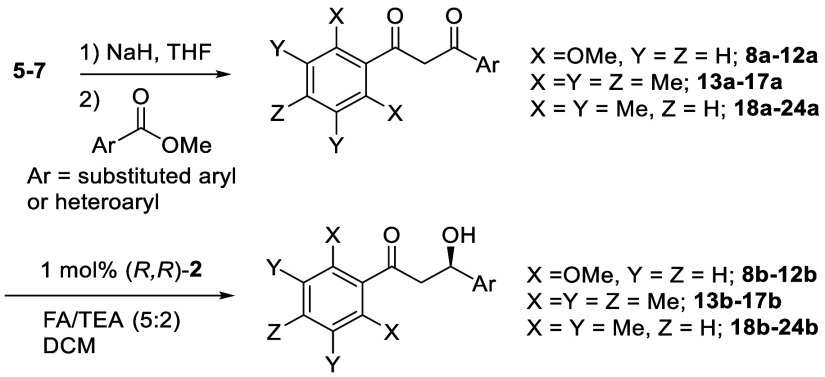
Synthetic route to 1,3-diketones **8a**–**24a** and subsequent ATH to alcohols **8b**–**24b**. The diketones were predominantly in the enol form (by NMR). Yields of **8a**–**12a** were 51–88%, those of **13a**–**17a** were 29–67%, and those of **18a**–**24a** were 48–86%. Racemic standards were prepared using a ca. 1:1 mixture of each enantiomer of the same catalyst.

**Figure 4:**
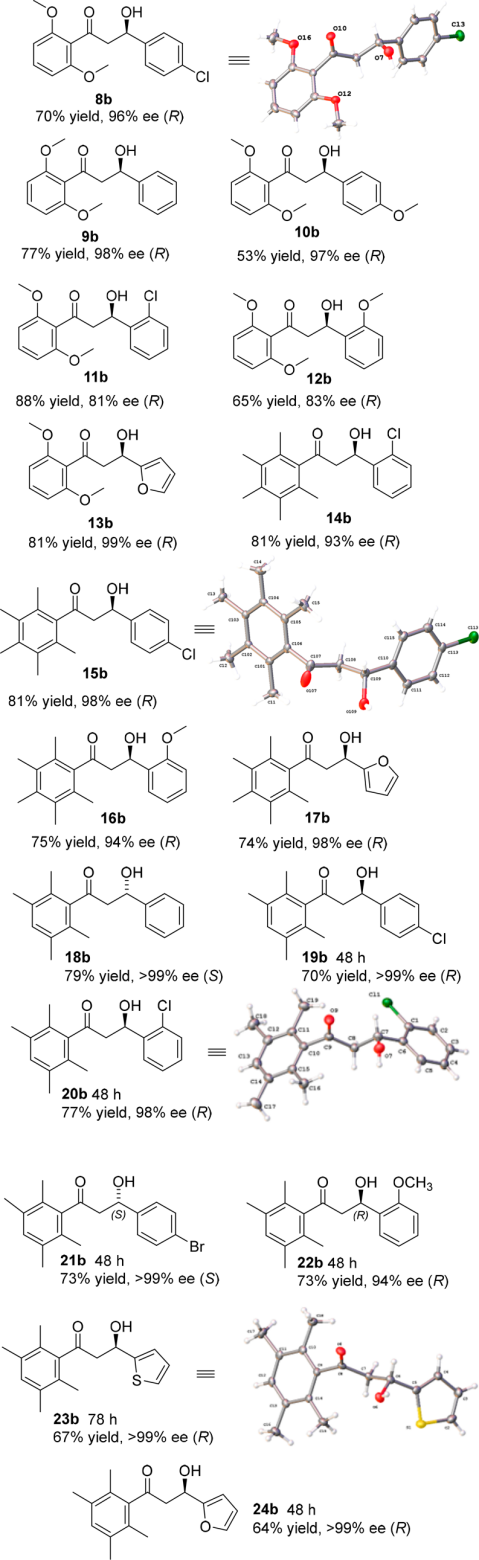
Products of ATH of diketones **8a**–**24a** using catalyst (*R,R*)-**2**, except for **18a** and **21a**, for which (*S,S*)-**2** was used. Reaction time is 24h unless a different time is listed. Full conversion was observed in all cases, isolated yields are listed. Where an X-ray structure was not obtained, the configuration was assigned by analogy.

In all cases, the less hindered ketone was reduced selectively, and in high ee. The *R* configuration of product **8b** was confirmed by an X-ray crystallographic structure analysis, indicating the preference for the *para*-chlorophenyl ring of the substrate to adopt the position adjacent to the η^6^-arene ring of the catalyst, while the bulky *diortho*-methoxyphenyl ring prevented reduction of the adjacent ketone, as predicted. Unsubstituted product **9b** and *para*-methoxy substituted **10b** were formed in 98% and 97% ee, respectively. The configurations were assigned as *R* by analogy with **8b**. Introducing *ortho*-chloro and *ortho*-methoxy groups onto one phenyl ring of the 1,3-diketone substrates provided a route to products **11b** and **12b** in 81% and 83% ee, respectively, indicating that an *ortho*-substituent causes a slight decrease of preference for the aromatic ring to create a CH/π interaction with η^6^-arene ring of the catalyst.^1^ However, the electron-rich heterocyclic product **13b** was formed in 99% ee with an *R*-configuration assigned to it.

Similar results were obtained with the pentamethylphenyl series, with products **14b**–**17b** formed in consistently high ee, including the *ortho*-substituted examples, and an X-ray crystal structure of **15b** (formed in high ee of 98%) also confirming that an *R*- alcohol was formed, analogous to the previous series.^7^ In the tetramethyl series, products **18b**–**24b** were formed in excellent ee, of >99% in several cases and only slighty lower for the two *ortho*-substituted examples. The ATH of **18a** was carried out on a 1 mmol scale. The X-ray structures of two derivatives (**20b** and **23b**) again served to confirm that the absolute stereochemistry of this series was consistent with the others. The conversion of the ATH products into esters via the Baeyer–Villiger reaction was explored. However, both the reaction of product **8b** and its TBS-protected derivative using mCPBA failed to give the anticipated products. Similar attempted oxidations of a pentamethyl derivative also failed (Supporting Information). Donohoe et al. have reported the conversion of pentamethylphenyl ketones to esters through reaction with bromine followed by an alcohol.^8^ For the conversion of β-hydroxy ketones to esters, however, it was necessary to convert tetramethylketones to the *p*-hydroxy derivative first, followed by oxidation and trapping with an alcohol.^8b,8c^ Following Donohoe’s protocol, (*S*)-**18b** (>99% ee) was reacted with phthaloyl peroxide to give **25**, followed by CAN oxidation to give ester **26** with retention of configuration in 98% ee (Figure 5). Apart from confirming the configuration of **18b**, this confirms that the Donohoe protocol works without significant decrease in ee.

**Figure 5:**
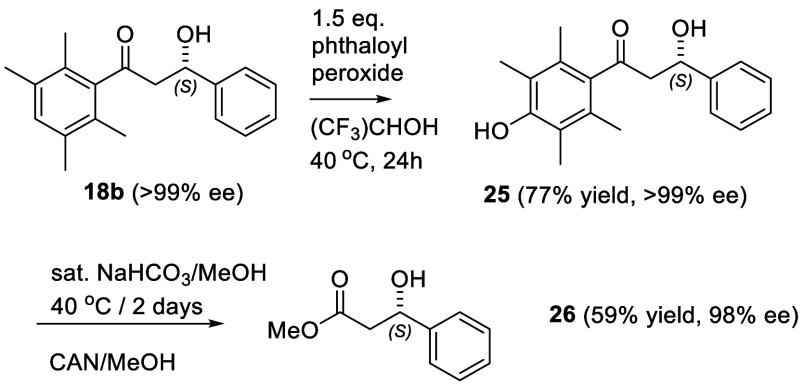
Synthetic route to methyl (*S*)-3-hydroxy-3-phenylpropanoate **24**.

1,4-Diketones **27a**–**30a**, the precursors to alcohols **27b**–**30b** were prepared by the reaction between unsaturated carboxylic acid **31** with the requisite aldehyde in the presence of thiazolium salt **32** (Supporting Information).^9^ Two 1,5-diketones, **33a** and **33b**, the precursors to alcohols **33b** and **34b**, were prepared through the reaction of cyclopropane **35** with **36** and **37** respectively, following a reported method (Supporting Information).^10^ Reduction of ketones **27a**–**30a**, **33a**, and **34a** using 1 mol % catalyst (*S,S*)-**2** again gave ATH products **27b**–**30b**, **33b**, and **34b** in high ee (Figure 6) in all cases other than the thiophene derivative **29b**. The oxidation of **27b** (97% ee) following the protocol in Figure 5 resulted in formation of lactone **38** in >99% ee,^11^ although with only a 13% yield,^12^ presumably the result of intramolecular trapping of the intermediate ester by the hydroxy group following the oxidation with CAN.

**Figure 6:**
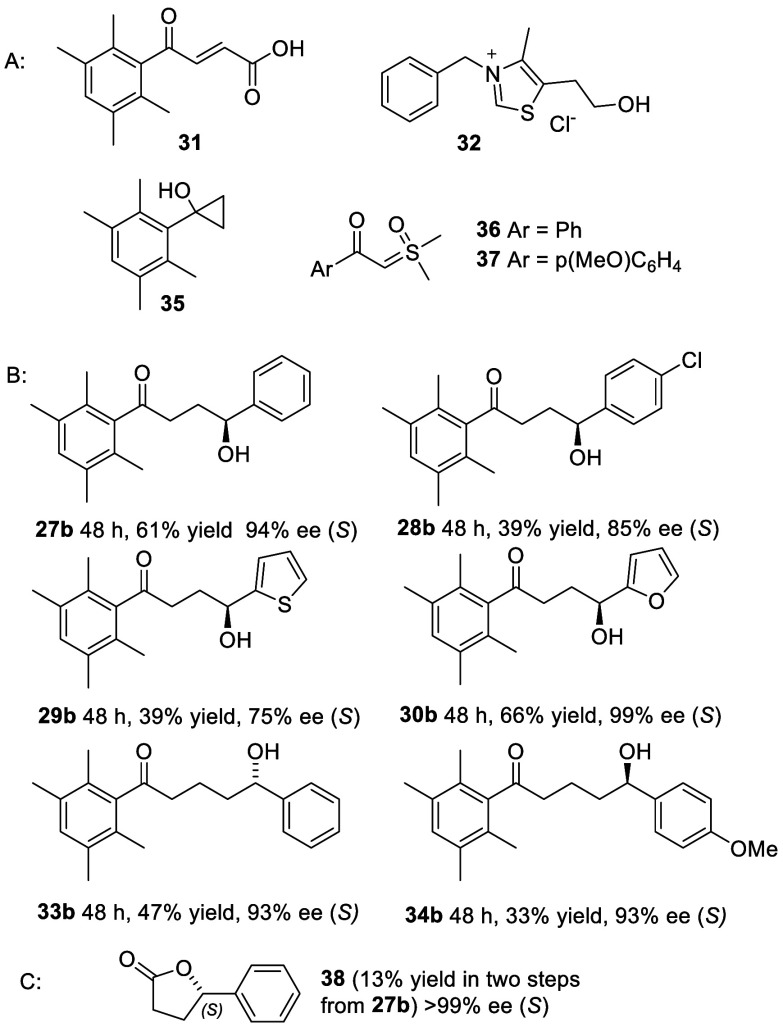
(A) Reagents used to prepare 1,4- and 1,5-diketones for this study. (B) ATH products of 1,4-diketones and 1,5-diketones rt. 1 mol % (*S,S*)-**2** was used in all cases except for **34b**. Isolated yields are listed. The configurations were assigned by analogy with the 1,3-series. (C) Oxidation product of **27b**.

In a final set of studies, diketones **39a**–**41a** were prepared in order to test the ATH of diketones in which the ketones are in different environments (Figure 7). The unhindered diketone **39a** was converted to diol **39b** in high dr and ee; following the reaction over time revealed that the internal α-alkoxy ketone was reduced ahead of the peripheral acetophenone, i.e. via **42**, likely due to the activating effect of the electron-withdrawing ArO group.^13^ The ATH of **40a** and **41a** resulted in the reduction of only the unhindered ketone in **40b** and **41b**, in 97% and 99% ee respectively, again demonstrating the complete control of regioselectivity which can be achieved by strategically placed bulky 2,6-substituents flanking the ketone (Figure 7). The absolute configuration of **40b** was confirmed by an X-ray crystal analysis (see the Supporting Information).

**Figure 7:**
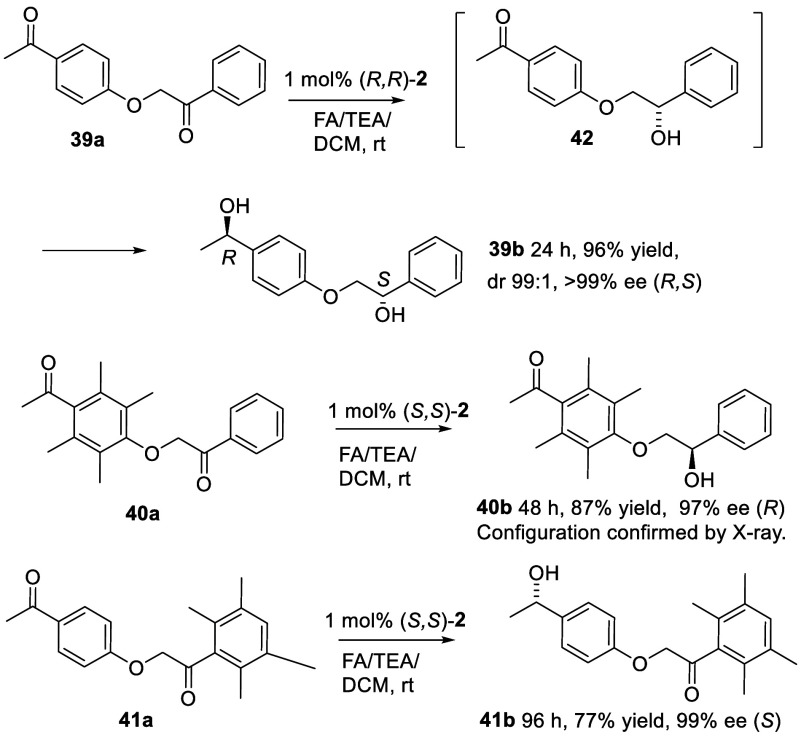
ATH of diketones with the ketones in nonsymmetrical positions. Diketone **39a** was also reduced with (*S,S*)-**2**, giving the product of opposite configuration.

In conclusion, we have demonstrated that certain bulky 2,6-disubstituted-aryls can prevent the ATH of adjacent ketones and hence facilitate the selective reduction of one ketone in a diketone, with high enantioselectivity. The products can subsequently be elaborated to further derivatives. This application may be of value when a regioselective reduction of one carbonyl is required, leaving the others available for further transformation.

## Data Availability

The data underlying this study are available in the published article, in its Supporting Information, and openly available in http://wrap.warwick.ac.uk/.

## Supporting Information Available

Procedures, characterization data, NMR spectra and HPLC chromatograms relating to ee and dr determination, and X-ray data (CCDC 2276986–2276989 and 2324377) are available as Supporting Information (PDF)
