# Supplementary material for: Regio- and Enantioselective Asymmetric Transfer Hydrogenation of One Carbonyl Group in a Diketone through Steric Hindrance
Source: J Org Chem. 2024 Feb 3;89(4):2759–63. doi: 10.1021/acs.joc.3c01950 (PMC10877611; doi:10.1021/acs.joc.3c01950)

Supporting information.

Regio and enantioselective Asymmetric Transfer Hydrogenation of one carbonyl group in a diketone through steric hindrance.

Noha Khamis,[a,b] Ye Zheng,[a] Marianna N. Diamantakis,[a] Guy Clarkson,[a] Jie Liu[c] and Martin Wills [a]*

a Department of Chemistry, The University of Warwick, Coventry, CV4 7AL, UK.

b Department of Chemistry, Faculty of science, University of Alexandria, Alexandria, Egypt.

c Department of Physics, The University of Warwick, Coventry, CV4 7AL, UK.

Contents

General Experimental	S2
Procedures for the preparation and ATH of **4-7**	S3
Procedures for 3-(aryl)-1-(2,6-dimethoxyphenyl)-3-hydroxypropan-1-ones **8b-13b**	S17
Procedures for 3-(aryl)-3-hydroxy-1-(2,3,4,5,6-pentamethylphenyl)propan-1-ones **14b-17b**	S85
Procedures for 3-hydroxy-3-aryl-1-(2,3,5,6-tetramethylphenyl)propan-1-ones **18b-24b**	S138
Procedures for 4-hydroxy-4-aryl-1-(2,3,5,6-tetramethylphenyl)butan-1-ones **27b-30b**	S222
Procedures for 5-hydroxy-5-phenyl-1-(2,3,5,6-tetramethylphenyl)pentan-1-ones **33b** and **34b**	S271
Procedures for Unsymmetrical hydroxyketones **39b-41b**	S298
Summary of unsuccessful reactions	S346

General Experimental:

General; Solvents and reagents for reactions were degassed prior to use and all reactions were carried out under a nitrogen atmosphere. Reactions at elevated temperature were maintained by thermostatically controlled oil-baths or aluminium heating blocks. A temperature of 0 °C refers to an ice slush bath, -78 °C to a dry ice acetone bath. Reactions were monitored by TLC using aluminium backed silica gel 60 (F254) plates, visualized using UV 254 nm and phosphomolybdic acid (PMA), potassium permanganate as appropriate. Flash column chromatography was performed using silica gel of 230-400 mesh size. Reagents and dry solvents were used as received from commercial sources unless otherwise stated. ^{1}H NMR spectra were recorded on a Bruker AV (250 MHz), Bruker DPX (300 or 400 MHz) or Bruker DRX (500 MHz). Chemical shifts are reported in δ units, parts per million relative to the singlet at 7.26 ppm for chloroform and at 2.50 ppm for dimethyl sulfoxide for TMS. Coupling constants (J) are measured in Hertz. Structural assignments were made with additional information from gCOSY, gHSQC, and gHMBC experiments. IR spectra were recorded on a Perkin-Elmer Spectrum One FT-IR Golden Gate. Mass spectra were recorded on a Bruker Esquire2000 or a Bruker MicroTOF mass spectrometer. Melting points were recorded on a Stuart Scientific SMP 1 instrument and are uncorrected. The chiral GC measurements were performed using a Hewlett-Packard 1050 instrument linked to a PC running DataApex Clarity software. HPLC measurements were performed out using a Hewlett Packard 1050 Series with a quaternary pump, autosampler and variable wavelength detector linked to a PC running DataApex Clarity software. Optical rotations were measured on an Optical Activity Ltd. AA-1000 Polarimeter and are reported in deg dm^{-1} cm^3 g^{-1}. Racemic standards of ATH products were prepared using a 1:1 mixture of each enantiomer of ATH catalyst **2**. Due to the low mass of each enantiomer which was used, the chiral HPLC of the racemic compound may not be an exact 1:1 mixture.

Procedures for the preparation and ATH of 4-7.

2-(1-Hydroxyethyl)benzene-1,3-diol.

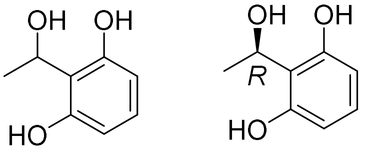

This compound is novel.

Synthesis of a racemic standard: (*R,R*)-3C-Tethered Ru(II)-TsDPEN catalyst (0.82 mg, 1.3 mmol, 0.5 mol%) and (*S,S*)-3C-tethered Ru(II)-TsDPEN catalyst (0.82 mg, 1.3 mmol, 0.5 mol%) were added to FA: TEA (5:2 azeotropic mixture, 0.18 mL) at rt and the mixture was stirred under a nitrogen atmosphere for 15 minutes; after which 2',6'-dihydroxyacetophenone **4** (40 mg, 0.26 mmol) was added. The reaction mixture was stirred under a nitrogen atmosphere and followed by TLC (3:2 hexane: EtOAc). After 24 h, the reaction was quenched using saturated $NaHCO_3$ solution (20 mL). EtOAc (20 mL) was added and the organic layer was separated. The aqueous layer was extracted with EtOAc (3 x 20 mL) and the combined organic layers were dried ($MgSO_4$) and filtered. The solvent was removed to give the crude product. The product was isolated via flash chromatography on silica eluted with 0-50% EtOAc in hexane to give 2-(1-hydroxyethyl)benzene-1,3-diol as a colorless oil (27.0 mg, 0.175 mmol, 67%). TLC: Rf ca 0.40 (3:2 hexane: EtOAc), strong UV and $KMnO_4$; HRMS (ESI+) *m/z*: [M+Na]+ Calcd for $C_8H_{10}NaO_3$ 177.0523; Found 177.0522; -0.6 ppm error; ν_{max} 3342 (br), 2972, 2931, 1600, 1494, 1422, 1283, 1262, 1013, 974 cm^{-1}; ^{1}H NMR (400 MHz, acetone-d_6): δ 8.65 (2H, s, ArOH), 6.73 (1H, t, *J* = 8.1, ArH), 6.16 (2H, d, *J* = 8.1, ArH), 5.40-5.33 (2H, m, OH + ArCH), 1.33 (3H, d, *J* = 6.4, CH_3); $^{13}C\{^1H\}$ NMR (100 MHz, acetone-d_6): δ 205.5 (C), 155.8 (C), 127.8 (CH), 107.2 (CH), 65.9 (CH), 22.5 (CH_3); *m/z* (ES-API+) 177.0 (M^+ + 23, 100%).

Enantiomeric excess and conversion determined by HPLC analysis (Chiralpak IC, 30 cm x 6 mm column, hexane:iPrOH 95:5, 1.0 mL/min, T = 25 °C) ketone 6.87 min, *R* and *S* isomer 17.5 min and 25.3 min, *R*-configuration product using (*R,R*)-**2** assigned by analogy with ATH of acetophenone.

(*R*)- 2-(1-Hydroxyethyl)benzene-1,3-diol.

(*R,R*)-3C-tethered Ru(II)-TsDPEN catalyst (1.63 mg, 2.63 mmol, 1 mol%) was added to FA: TEA (5:2 azeotropic mixture, 0.18 mL) at rt and the mixture was stirred under

a nitrogen atmosphere for 10-15 minutes; after which 2',6'-dihydroxyacetophenone **4** (40.0 mg, 0.263 mmol) was added. The reaction mixture was stirred under a nitrogen atmosphere overnight. The reaction was followed by TLC (3:2 hexane: EtOAc). After 24 h, the reaction was quenched using saturated $NaHCO_3$ solution (20 mL). EtOAc (20 mL) was added and the organic layer was separated. The aqueous layer was extracted with EtOAc (3 x 20 mL) and the combined organic layers were dried ($MgSO_4$) and filtered. The solvent was removed to give the crude product. The product was isolated via flash chromatography on silica eluted with 0-50% EtOAc in hexane to give (*R*)-2-(1-hydroxyethyl)benzene-1,3-diol as a colorless oil (14 mg, 0.091 mmol, 35%). The reaction was also followed by HPLC (Chiralpak IC, 30 cm x 6 mm column, hexane:iPrOH 95:5, 1.0 mL/min, T = 25°C): 100% conversion; $[\alpha]_D^{26}$ +30.7 (c 0.187 in MeOH) 73% ee (*R*).

^1H NMR (400 MHz, acetone-d_6) of 2-(1-hydroxyethyl)benzene-1,3-diol.

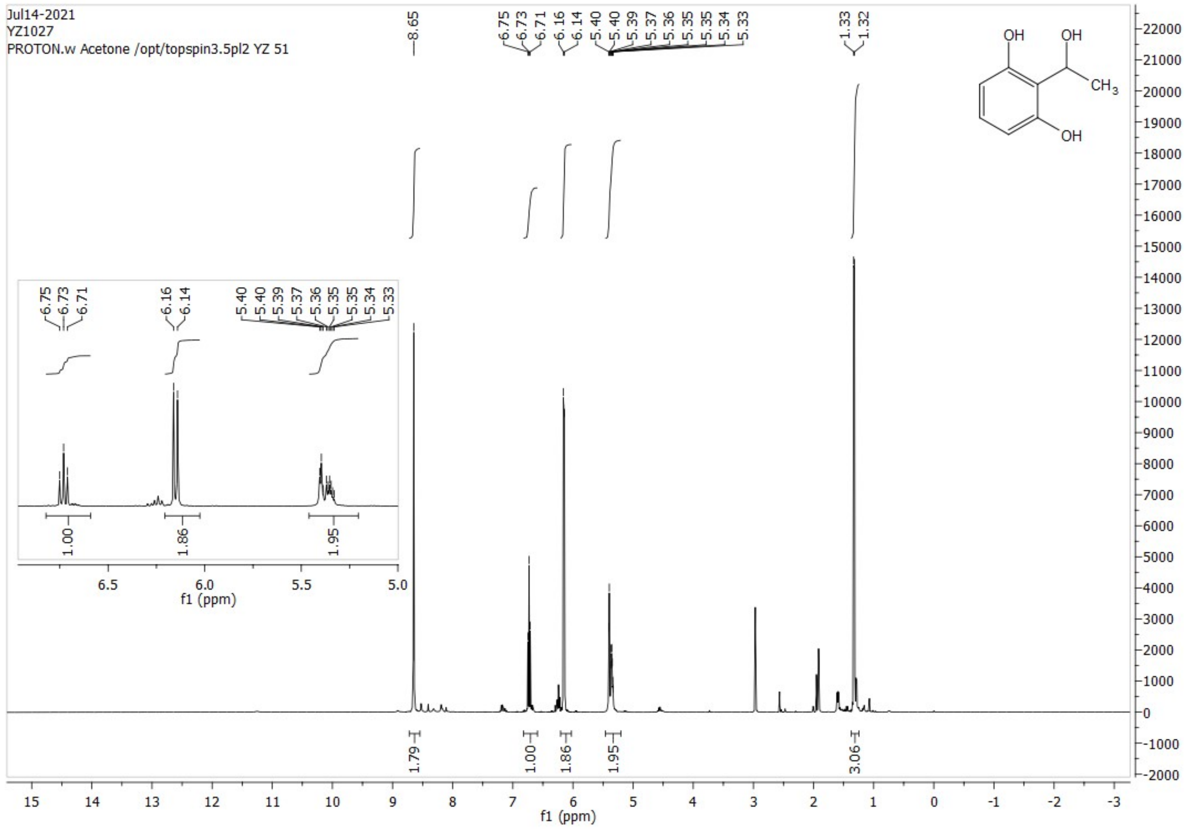

$^{13}C\{^{1}H\}$ NMR (100 MHz, acetone-d_6) of 2-(1-hydroxyethyl)benzene-1,3-diol.

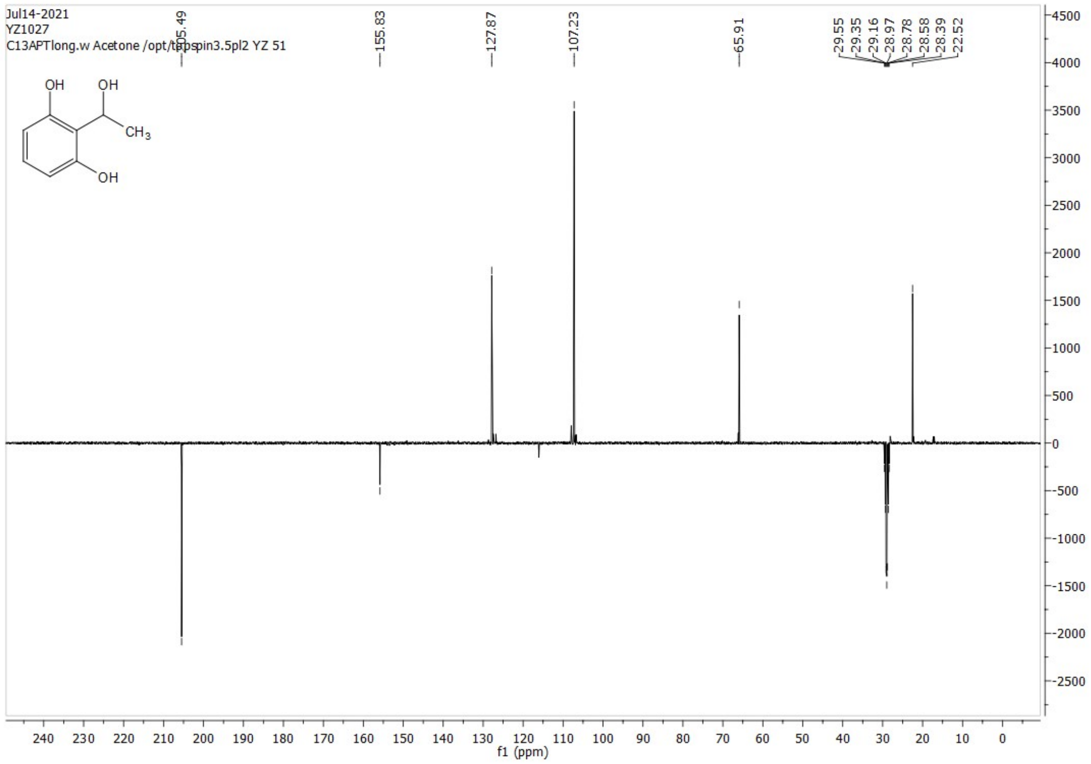

COSY (400 MHz, acetone-d_6) of 2-(1-hydroxyethyl)benzene-1,3-diol.

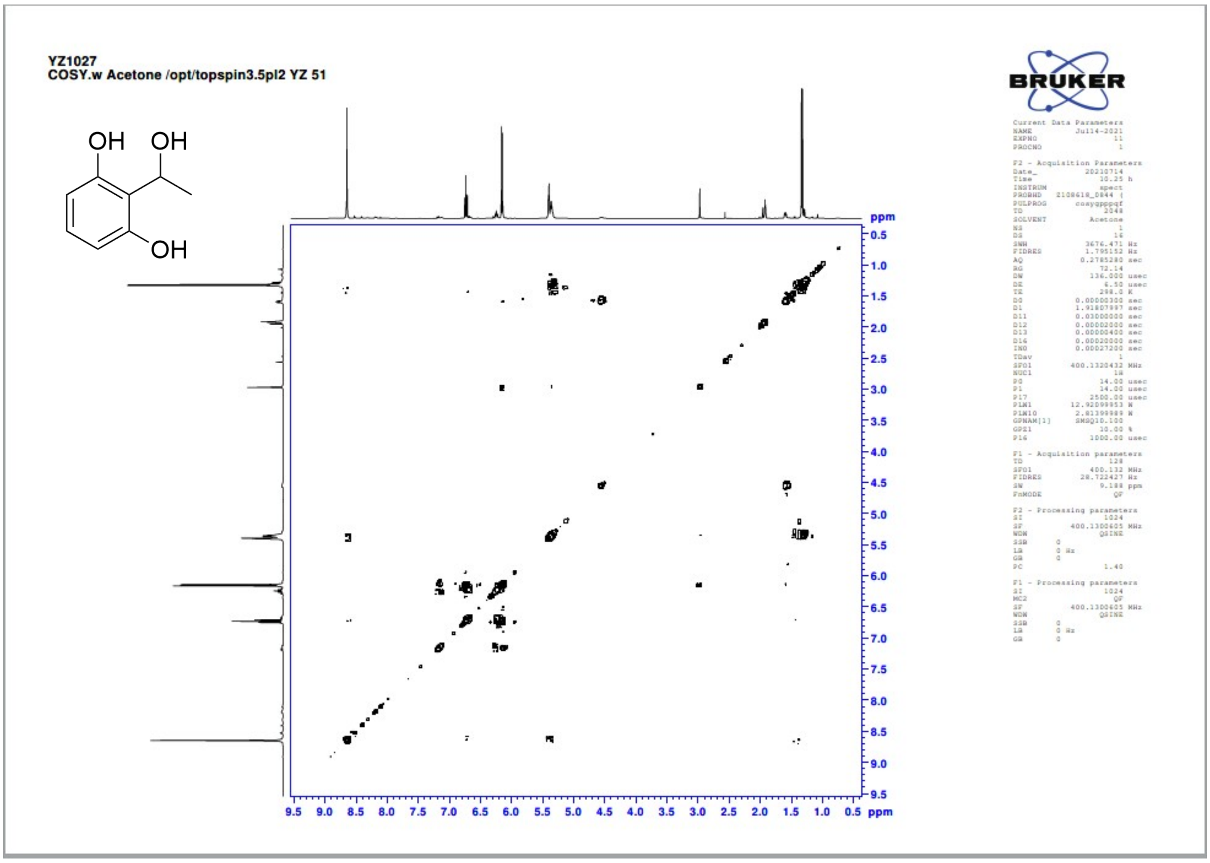

HSQC (400 MHz, acetone-d$_6$) of 2-(1-hydroxyethyl)benzene-1,3-diol.

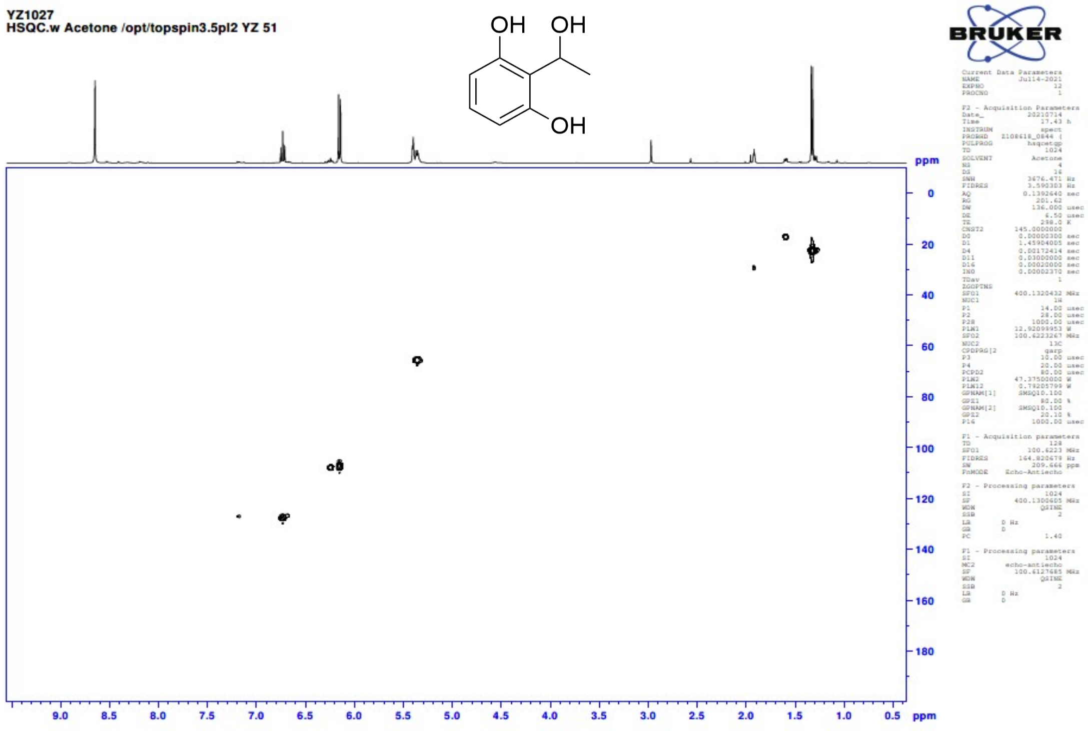

HMBC (400 MHz, acetone-d$_6$) of 2-(1-hydroxyethyl)benzene-1,3-diol.

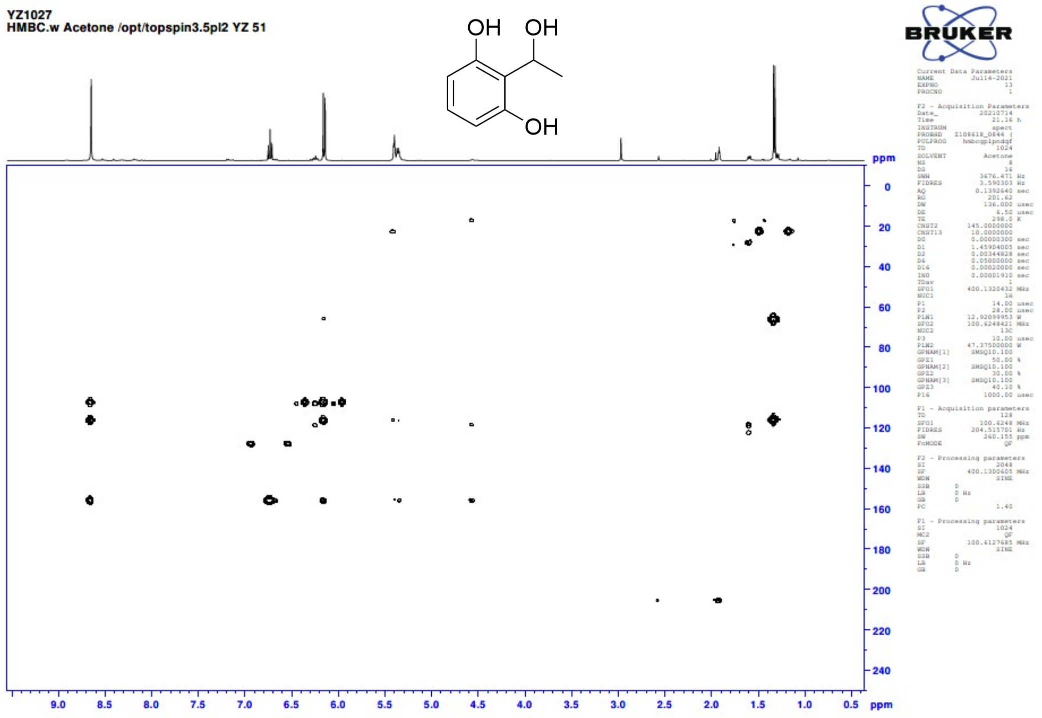

HPLC of 2',6'-dihydroxyacetophenone **4**.

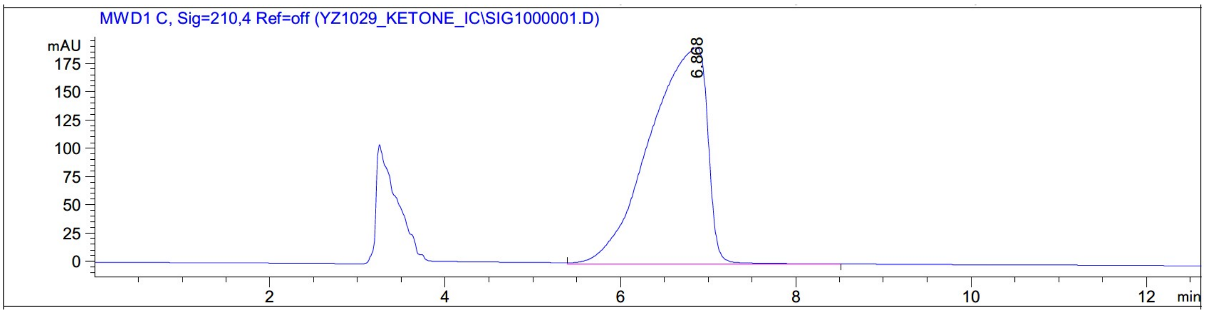

Signal 2: MWD1 C, Sig=210,4 Ref=off

Peak #	RetTime [min]	Type	Width [min]	Area [mAU*s]	Height [mAU]	Area %
1	6.868	VB	0.7781	8761.99512	191.38637	100.0000
Totals :				8761.99512	191.38637	

HPLC of racemic 2-(1-hydroxyethyl)benzene-1,3-diol.

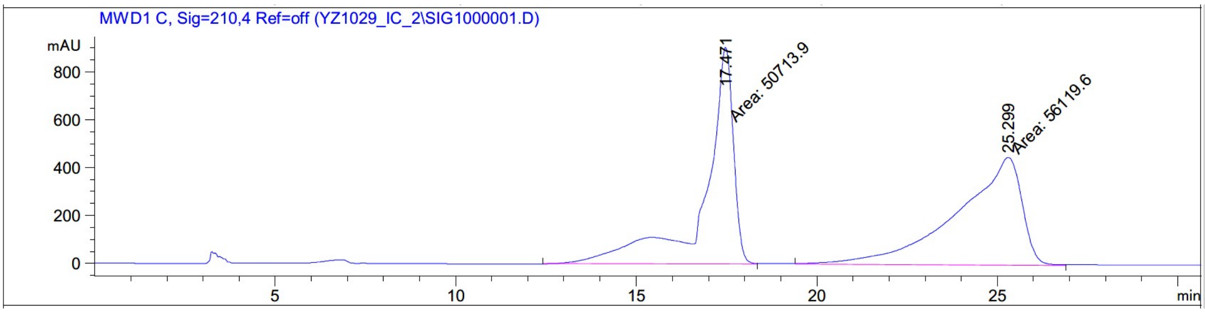

Signal 2: MWD1 C, Sig=210,4 Ref=off

Peak #	RetTime [min]	Type	Width [min]	Area [mAU*s]	Height [mAU]	Area %
1	17.471	MM	0.9319	5.07139e4	907.01807	47.4701
2	25.299	MM	2.0744	5.61196e4	450.89786	52.5299
Totals :				1.06833e5	1357.91592	

HPLC of (*R*)- 2-(1-hydroxyethyl)benzene-1,3-diol.

(*R,R*)-3C-tethered Ru(II)-TsDPEN catalyst (after 24 h, 100% conversion, 73% ee (*R*))

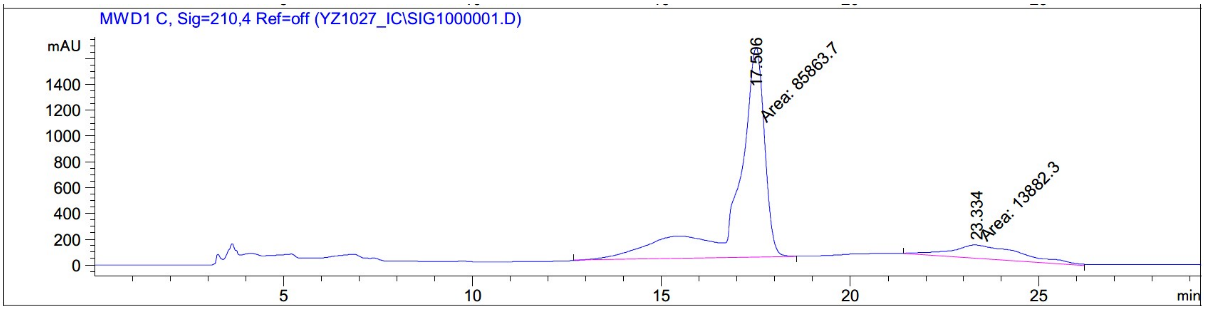

Signal 2: MWD1 C, Sig=210,4 Ref=off

Peak #	RetTime [min]	Type	Width [min]	Area [mAU*s]	Height [mAU]	Area %
1	17.506	MM	0.8803	8.58637e4	1625.73071	86.0823
2	23.334	MM	2.2345	1.38823e4	103.54422	13.9177
Totals :				9.97460e4	1729.27493	

1-(2,6-Dimethoxyphenyl)ethan-1-one 5.

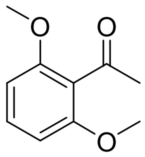

This compound has been reported and fully characterized: L. R. Mills, C. Zhou, E. Fung and S. A. L. Rousseaux, *Org. Lett.*, 2019, **21**, 8805-8809.

To a solution of 1-(2,6-dihydroxyphenyl)ethan-1-one **4** (760 mg, 5.00 mmol) in DMF (3 mL) was added potassium carbonate (2.07 g, 15.0 mmol) and iodomethane (2.13 g, 15.0 mmol) at rt. The reaction mixture was left stirring under the nitrogen atmosphere overnight. TLC (9:1 hexane: EtOAc) after this time indicated full conversion. Solvent was removed to give the crude product. The product was isolated via flash chromatography on silica eluted with 0-20% EtOAc in hexane to give 1-(2,6-dimethoxyphenyl)ethan-1-one **5** as a white solid (746 mg, 4.14 mmol, 83%). TLC: Rf ca 0.20 (9:1 hexane: EtOAc), strong UV and $KMnO_4$; ^{1}H NMR (400 MHz, $CDCl_3$): δ 7.25 (1H, t, J = 8.4, ArH), 6.56 (2H, d, J = 8.4, ArH), 3.79 (6H, s, OCH_3), 2.47 (3H, s, CH_3); ^{13}C{^{1}H} NMR (100 MHz, $CDCl_3$): δ 202.7 (C), 156.7 (C), 130.7 (CH), 120.5 (C), 104.0 (CH), 55.8 (CH_3), 32.3 (CH_3); *m/z* (ES-API+) 203.0 (M^+ + 23, 100%). The data matched that reported.

^{1}H NMR (400 MHz, $CDCl_3$) of 1-(2,6-dimethoxyphenyl)ethan-1-one **5.**

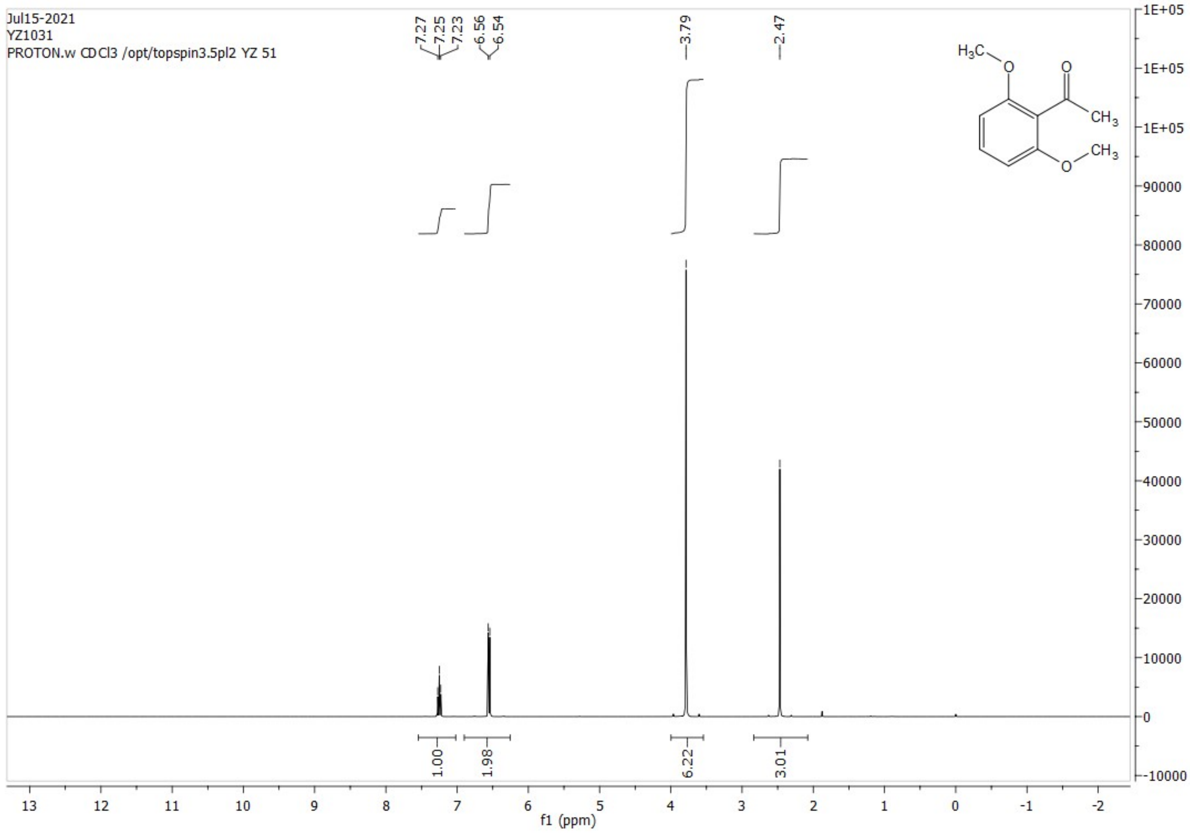

$^{13}C\{^{1}H\}$ NMR (100 MHz, $CDCl_3$) of 1-(2,6-dimethoxyphenyl)ethan-1-one **5**.

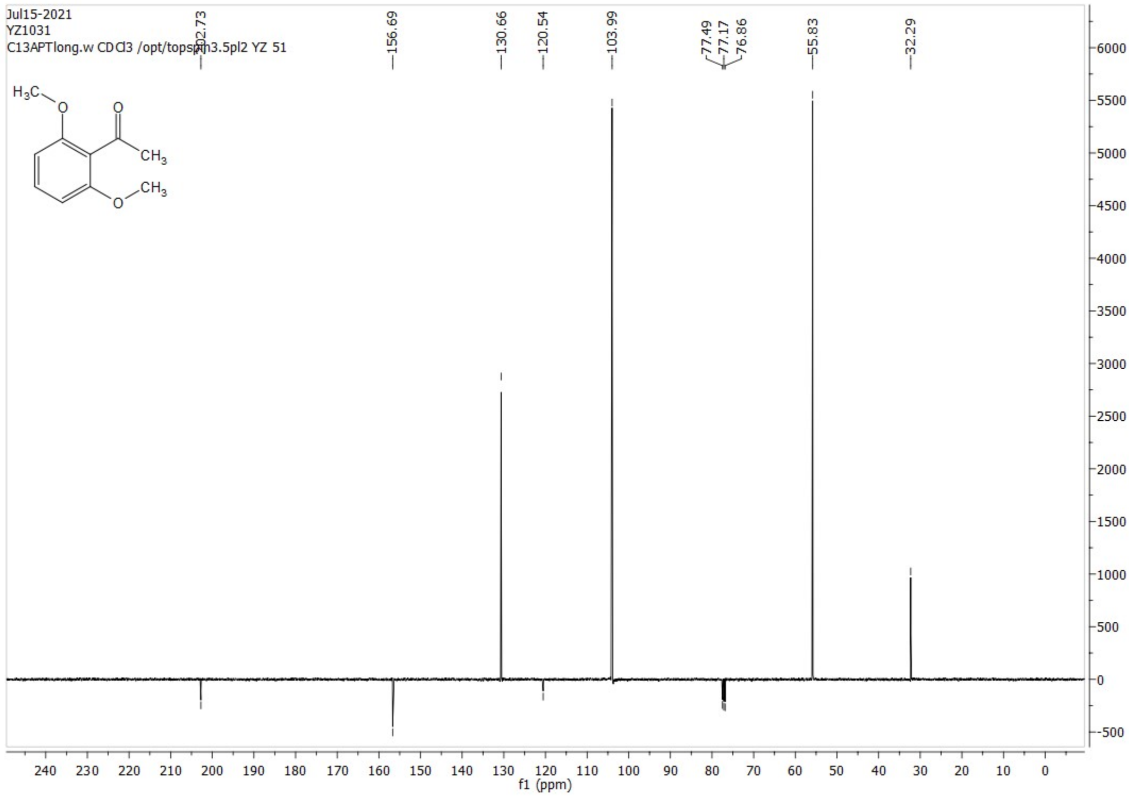

HPLC of 1-(2,6-dimethoxyphenyl)ethan-1-one **5**.

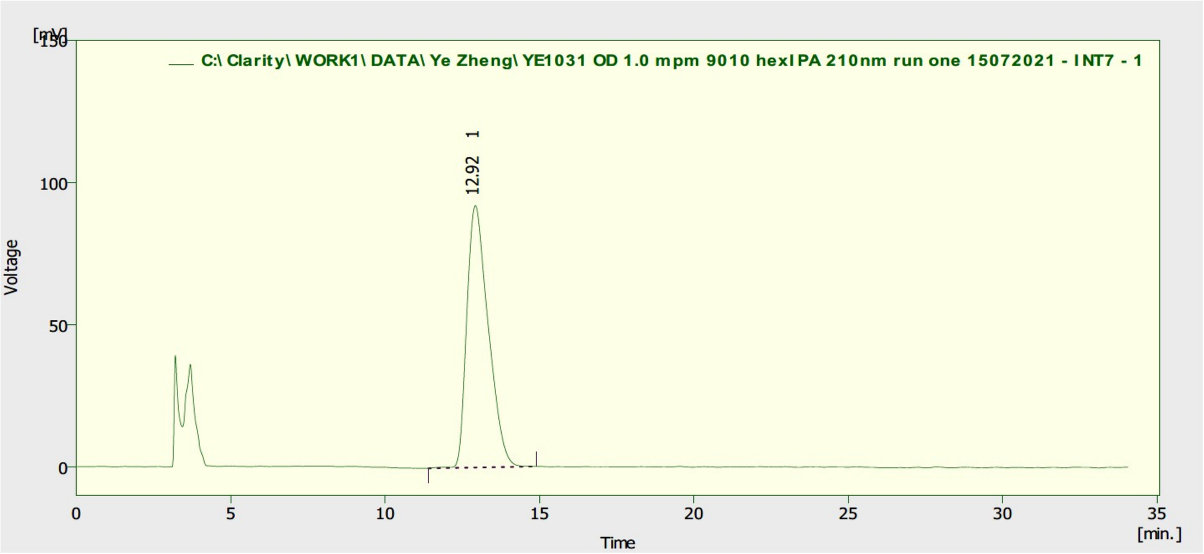

Result Table (Uncal - C:\Clarity\WORK1\DATA\Ye Zheng\YE1031 OD 1.0 mpm 9010 hexIPA 210nm run one 15072021 - INT7 - 1)

	Reten. Time [min]	Area [mV.s]	Height [mV]	Area [%]	Height [%]	W05 [min]	Compound Name
1	12.923	4450.146	92.107	100.0	100.0	0.76	
	Total	4450.146	92.107	100.0	100.0		

1-(2,3,4,5,6-Pentamethylphenyl)ethan-1-one 6.

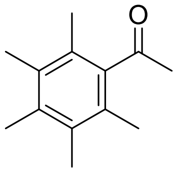

This compound has been reported and fully characterized: Kaithal, A.; Gracia, L. L.; Camp, C.; Quadrelli, E. A.; Leitner, W. *J. Am. Chem. Soc.*, **2019**, *141*, 17487-17492.

To a solution of pentamethylbenzene (444 mg, 3.00 mmol) was in DCM (15 mL) at 0 °C was added dropwise acetyl chloride (259 mg, 3.30 mmol) and $AlCl_3$ (519 mg, 3.90 mmol). The reaction mixture was warmed to rt and left stirring under the nitrogen atmosphere for 2 h, followed by TLC (9:1 hexane: EtOAc). The mixture was poured into ice water (20 mL). DCM (20 mL) was added and the organic layer was separated. The aqueous layer was extracted with DCM (3 × 20 mL), and the combined organic layers were washed with brine (20 mL), dried ($MgSO_4$) and filtered. Solvent was removed to give the crude product. The product was isolated via flash chromatography on silica eluted with 0-10% EtOAc in hexane to give 1-(2,3,4,5,6-pentamethylphenyl)ethan-1-one **6** as a white solid (506 mg, 2.66 mmol, 89%). TLC: Rf ca 0.60 (9:1 hexane: EtOAc), strong UV and $KMnO_4$; ^{1}H NMR (400 MHz, $CDCl_3$); δ 2.47 (3H, s, CH_3), 2.24 (3H, s, CH_3), 2.20 (6H, s, CH_3), 2.14 (6H, s, CH_3); ^{13}C{^{1}H} NMR (100 MHz, $CDCl_3$): δ 210.1 (C), 141.0 (C), 135.4 (C), 133.1 (C), 127.0 (C), 33.1 (CH_3), 17.1 (CH_3), 16.7 (CH_3), 16.0 (CH_3); *m/z* (ES-API+) 213.1 (M^+ + 23, 100%). The data matched that reported.

^{1}H NMR (400 MHz, $CDCl_3$) of 1-(2,3,4,5,6-pentamethylphenyl)ethan-1-one **6.**

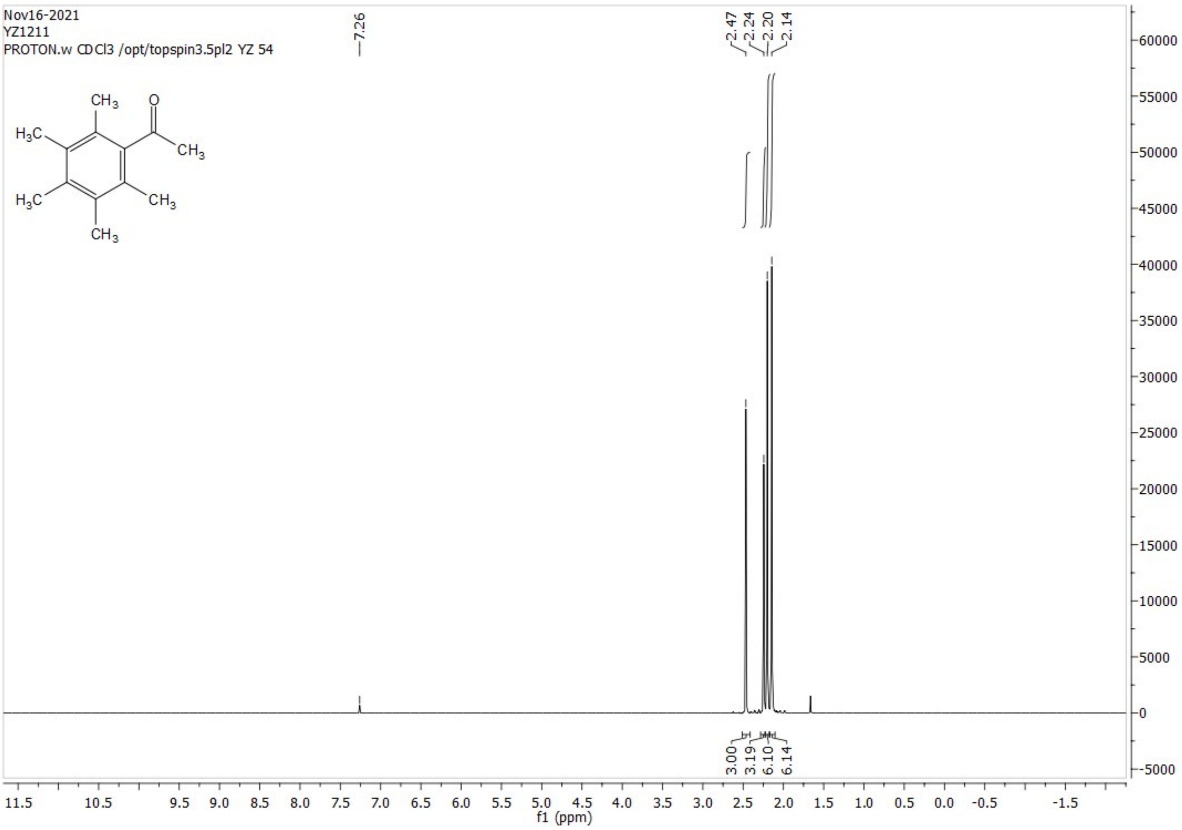

^{13}C{^{1}H} NMR (100 MHz, $CDCl_3$) of 1-(2,3,4,5,6-pentamethylphenyl)ethan-1-one **6.**

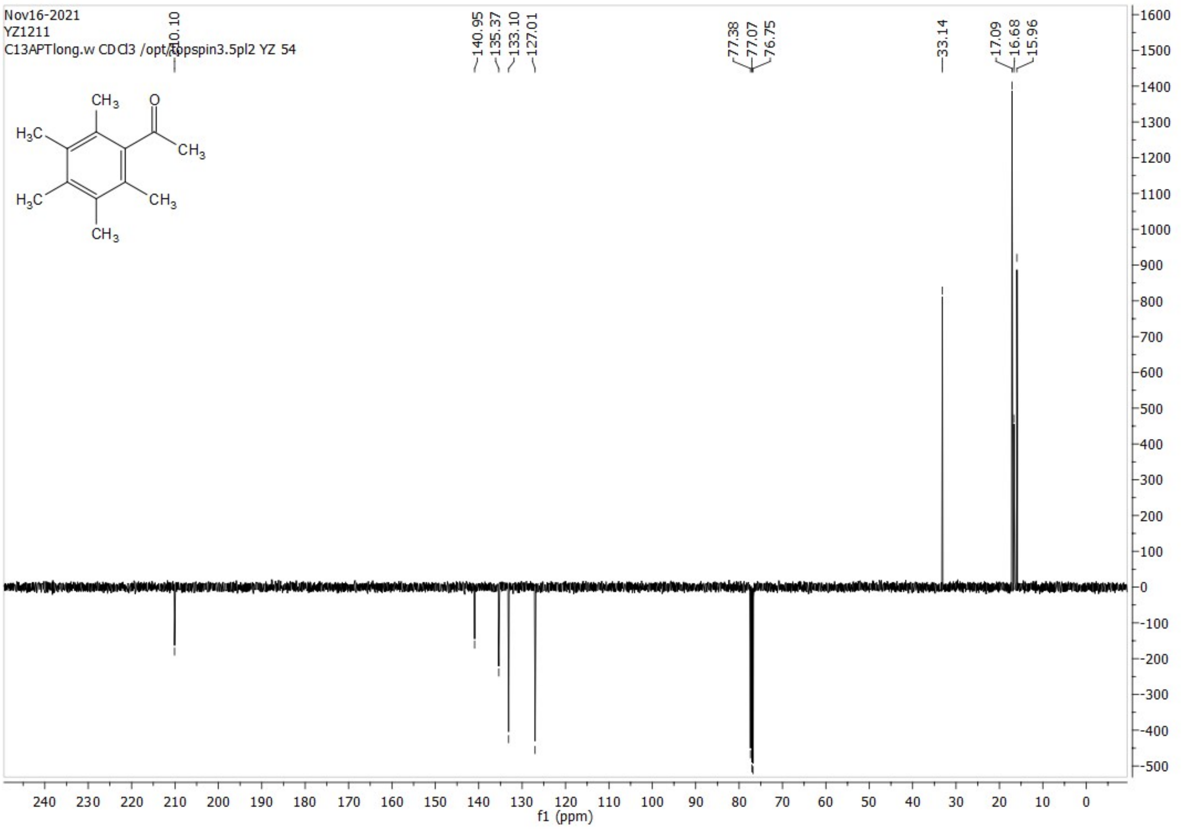

1-(2,3,5,6-Tetramethylphenyl)ethan-1-one 7.

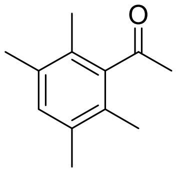

This compound has been reported and fully characterized. Waiba, S.; Jana, S. K.; Jati, A.; Jana, A.; Maji, B., Manganese complex-catalysed α-alkylation of ketones with secondary alcohols enables the synthesis of β-branched carbonyl compounds. *Chem Commun* **2020**, *56*, 8376-8379.

To a solution of durene (4.00 g, 29.8 mmol) was in DCM (80 mL) at 0 °C was added dropwise acetyl chloride (2.60 g, 32.8 mmol) and $AlCl_3$ (5.16 g, 38.8 mmol). The reaction mixture was warmed to rt and left stirring under the nitrogen atmosphere for 3h and followed by TLC (9:1 hexane: EtOAc). The mixture was poured into ice water (50 mL). DCM (50 mL) was added, and the organic layer was separated. The aqueous layer was extracted with DCM (3 × 50 mL), and the combined organic layers were washed with brine (30 mL), dried ($MgSO_4$) and filtered. Solvent was removed to give the crude product. The product was isolated via flash chromatography on silica eluted with 5-10% EtOAc in petroleum ether to give 1-(2,3,5,6-tetramethylphenyl)ethan-1-one **7** as a white solid (4.58 g, 26.0 mmol, 87%). TLC: Rf ca 0.65 (10:1 hexane: EtOAc), strong UV and $KMnO_4$; ^{1}H NMR (300 MHz, $CDCl_3$): δ 6.98 (1H, s, CH), 2.47 (3H, s, CH_3), 2.24 (3H, s, CH_3), 2.12 (3H, s, CH_3); $^{13}C\{^1H\}$ (126 MHz, $CDCl_3$): δ 209.9 (C=O), 143.1 (C), 134.4 (C), 131.5 (CH), 127.6 (C), 32.8 (CH_3), 19.5 (CH_3), 15.9 (CH_3); *m/z* (ES-API+) 199.1 (M+ + 23, 100%). The data matched that reported.

^{1}H NMR (300 MHz, $CDCl_3$) of 1-(2,3,5,6-tetramethylphenyl)ethan-1-one **7**.

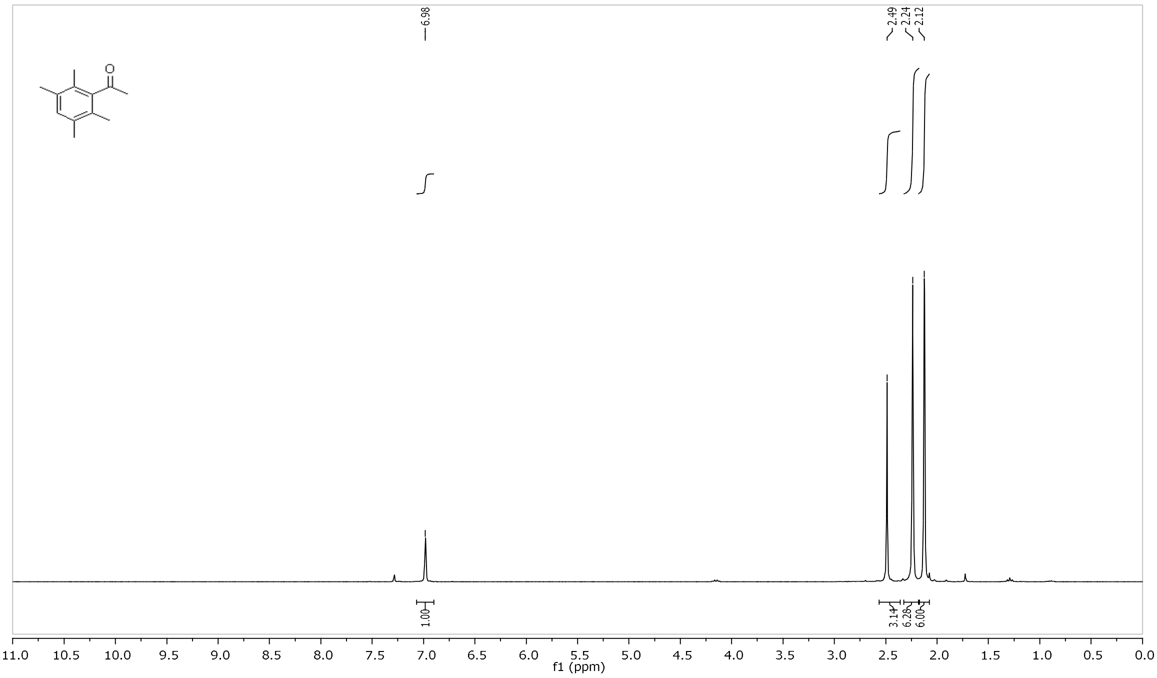

^{13}C{^{1}H} NMR (126 MHz, $CDCl_3$) of 1-(2,3,5,6-tetramethylphenyl)ethan-1-one **7**.

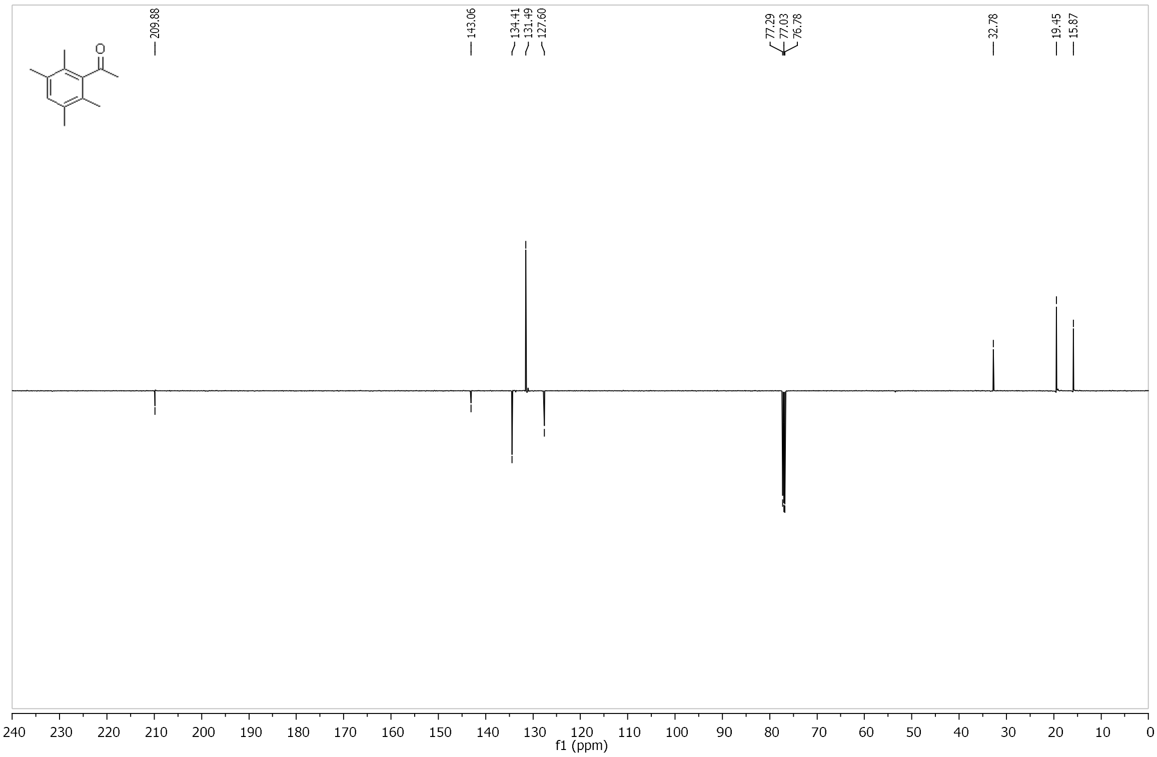

Procedure for the attempted ATH of ketones 5, 6 and 7.

(*R,R*)-3C-Tethered Ru(II)-TsDPEN catalyst (2.6 mg, 4.21 mmol, 1 mol%) was added to FA: TEA (5:2 azeotropic mixture, 0.36 mL) at rt and the mixture was stirred under a nitrogen atmosphere for 10-15 minutes; after which a solution of 1-(2,3,4,5,6-pentamethylphenyl)ethan-1-one **6** (80.0 mg, 0.421 mmol) in DCM (0.50 mL) was added. The reaction mixture was stirred under a nitrogen atmosphere, and followed by TLC (9:1 hexane: EtOAc). After 7 days, TLC (4:1 hexane: EtOAc) and NMR indicated no reduction.

Procedures for 3-(aryl)-1-(2,6-dimethoxyphenyl)-3-hydroxypropan-1-ones 8b-13b. 1-(4-Chlorophenyl)-3-(2,6-dimethoxyphenyl)propane-1,3-dione 8a.

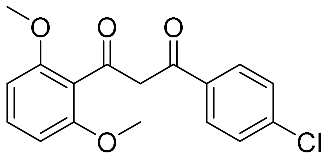

This compound is novel.

To a solution of sodium hydride (393 mg, 60% dispersion in mineral oil, 9.84 mmol) in THF (4 mL) at 0 °C was added dropwise a solution of 1-(2,6-dimethoxyphenyl)ethan-1-one **5** (355 mg, 1.97 mmol) in THF (4 mL). The reaction mixture was stirred under a nitrogen atmosphere at 0 °C for 30 min and then at rt for 30 min, after which ethyl 4-chlorobenzoate (1.82 g, 9.84 mmol) was added dropwise. The reaction mixture was then heated to 65 °C and left stirring under the nitrogen atmosphere overnight. The reaction was followed by TLC (4:1 hexane: EtOAc). The mixture was quenched by 2M HCl solution (20 mL). EtOAc (20 mL) was added and the organic layer was separated. The aqueous layer was extracted with EtOAc (3 × 20 mL), and the combined organic layers were washed with saturated $NaHCO_3$ solution (2 × 20 mL) and brine (20 mL), dried ($MgSO_4$) and filtered. Solvent was removed to give the crude product. The product was isolated via flash chromatography on silica eluted with 0-50% EtOAc in hexane to give 1-(4-chlorophenyl)-3-(2,6-dimethoxyphenyl)propane-1,3-dione **8a** as a white solid (514 mg, 1.62 mmol, 82%). TLC: Rf ca 0.40 (4:1 hexane: EtOAc), strong UV and $KMnO_4$; Mp: 120 °C; HRMS: (ESI+) *m/z*: [M+H]+ Calcd for $C_{17}H_{15}{}^{35}ClNaO_4$ 341.0553; Found 341.0551; -0.6 ppm error; ν_{max} 2924, 2832, 1588, 1486, 1469, 1249, 1109, 1086, 1030, 1008, 779 cm^{-1}; enol: keto = 91:9; ^1H NMR (500 MHz, $CDCl_3$); δ 7.85 (2H, d, J = 8.6, ArH), 7.42 (2H, d, J = 8.6, ArH), 7.33 (1H, t, J = 8.4, ArH), 6.62 (2H, d, J = 8.4, ArH), 6.36 (0.91H, s, CH of enol form), 4.40 (0.18H, s, CH_2 of keto form), 3.82 (5.46H, s, OCH_3 of enol form), 3.75 (0.54H, s, OCH_3 of keto form); $^{13}C\{^1H\}$ NMR (125 MHz, $CDCl_3$); δ keto form: 197.4 (C), 192.6 (C), 156.9 (C), 139.7 (C), 135.2 (C), 131.7 (CH), 130.4 (CH), 128.8 (CH), 118.9 (C), 104.0 (CH), 56.0 (CH_2), 55.8 (CH_3); enol form: 189.5 (C), 180.3 (C), 157.7 (C), 138.3 (C), 133.5 (C), 131.4 (CH), 128.9 (CH), 128.4 (CH), 116.9 (C), 104.1 (CH), 100.5 (CH), 56.1 (CH_3); *m/z* (ES-API+) 341.0 (M^+ + 23, 100%).

Enantiomeric excess and conversion determined by HPLC analysis (Chiralpak IC, 30 cm x 6 mm column, hexane:iPrOH 80:20, 1.0 mL/min, T = 25°C) ketone 15.0 min, *R* isomer 24.7 min and *S* isomer 27.9 min.

^{1}H NMR (500 MHz, $CDCl_3$) of 1-(4-chlorophenyl)-3-(2,6-dimethoxyphenyl)propane-1,3-dione **8a.**

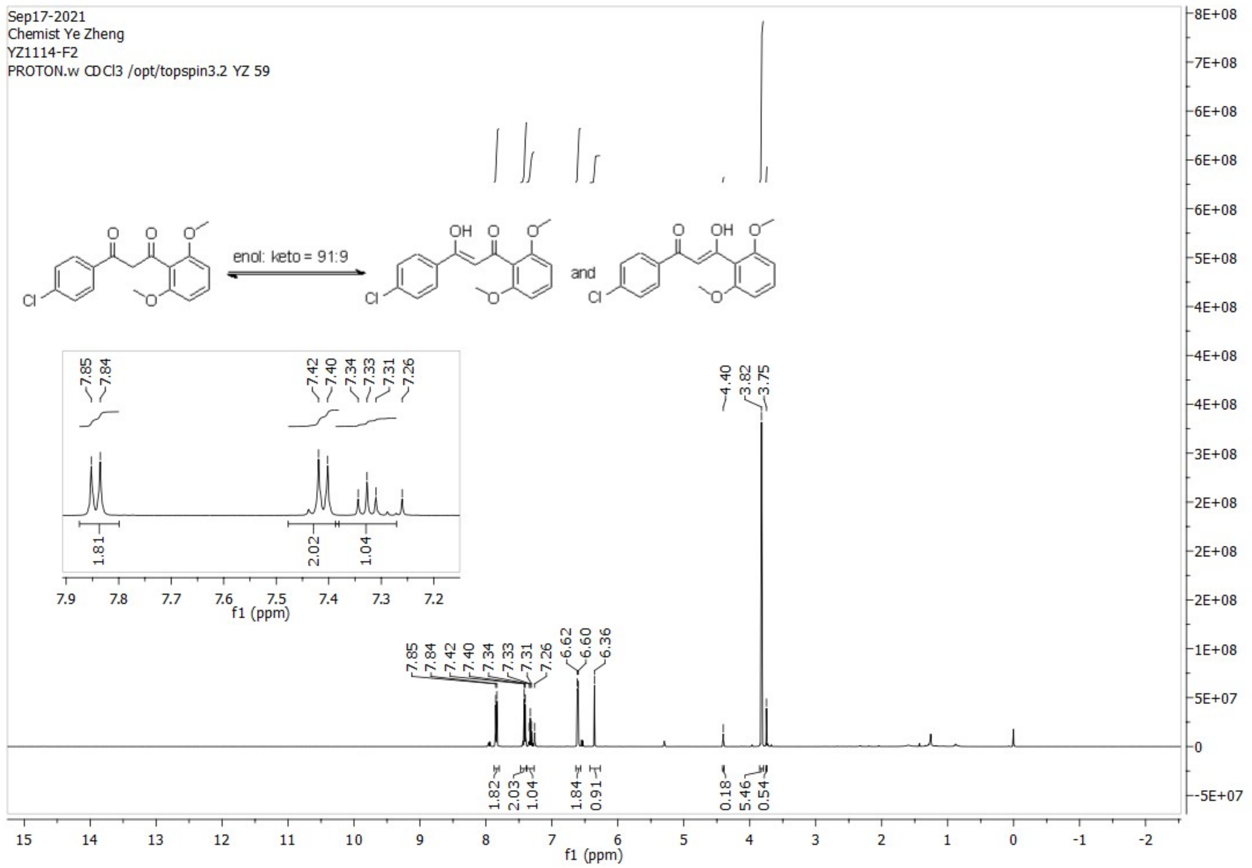

$^{13}C\{^{1}H\}$ NMR (125 MHz, $CDCl_3$) of 1-(4-chlorophenyl)-3-(2,6-dimethoxyphenyl)propane-1,3-dione **8a.**

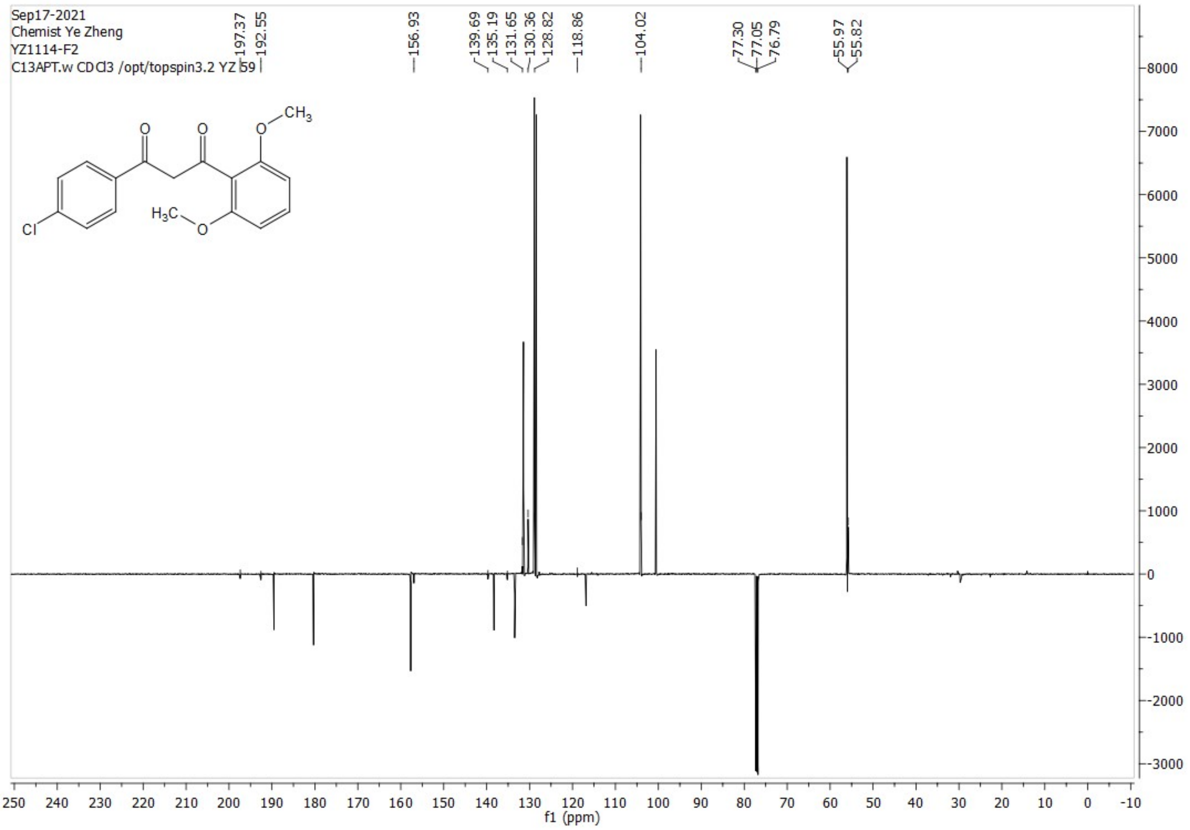

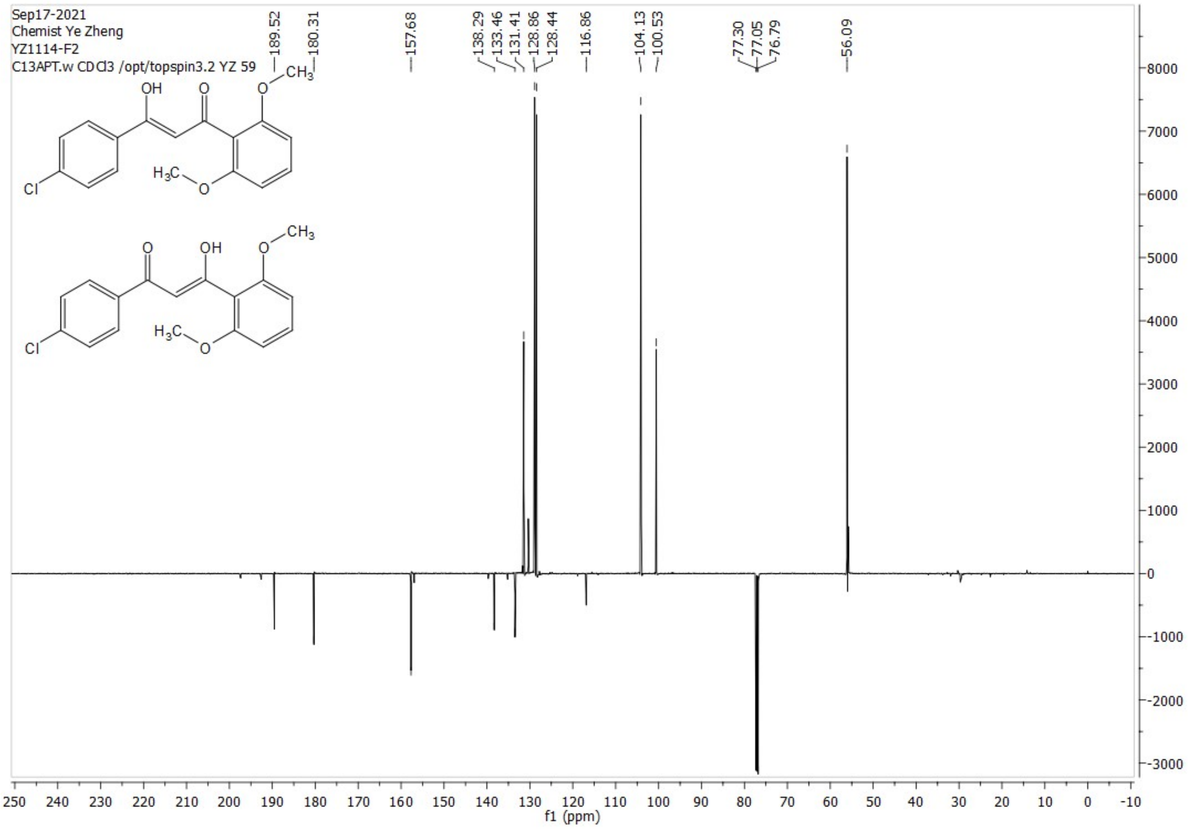

COSY (500 MHz, $CDCl_3$)) of 1-(4-chlorophenyl)-3-(2,6-dimethoxyphenyl)propane-1,3-dione **8a.**

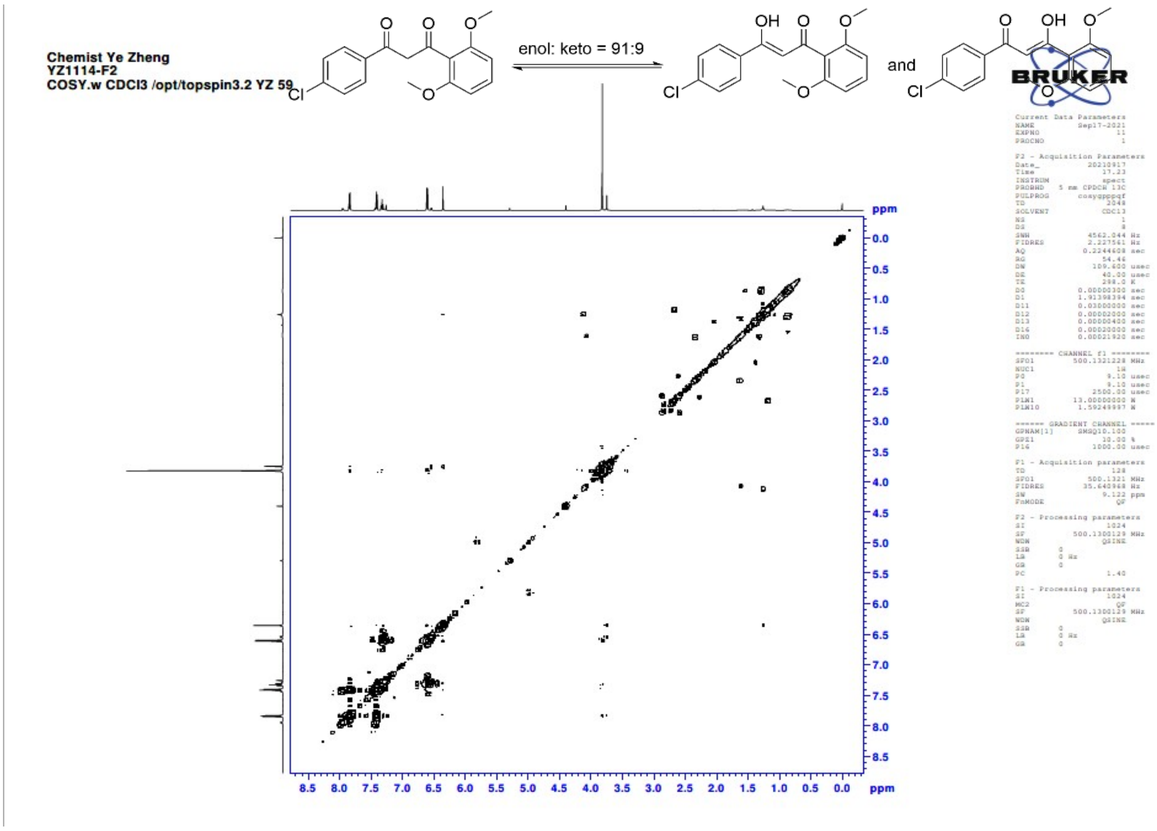

HSQC (500 MHz, $CDCl_3$)) of 1-(4-chlorophenyl)-3-(2,6-dimethoxyphenyl)propane-1,3-dione **8a.**

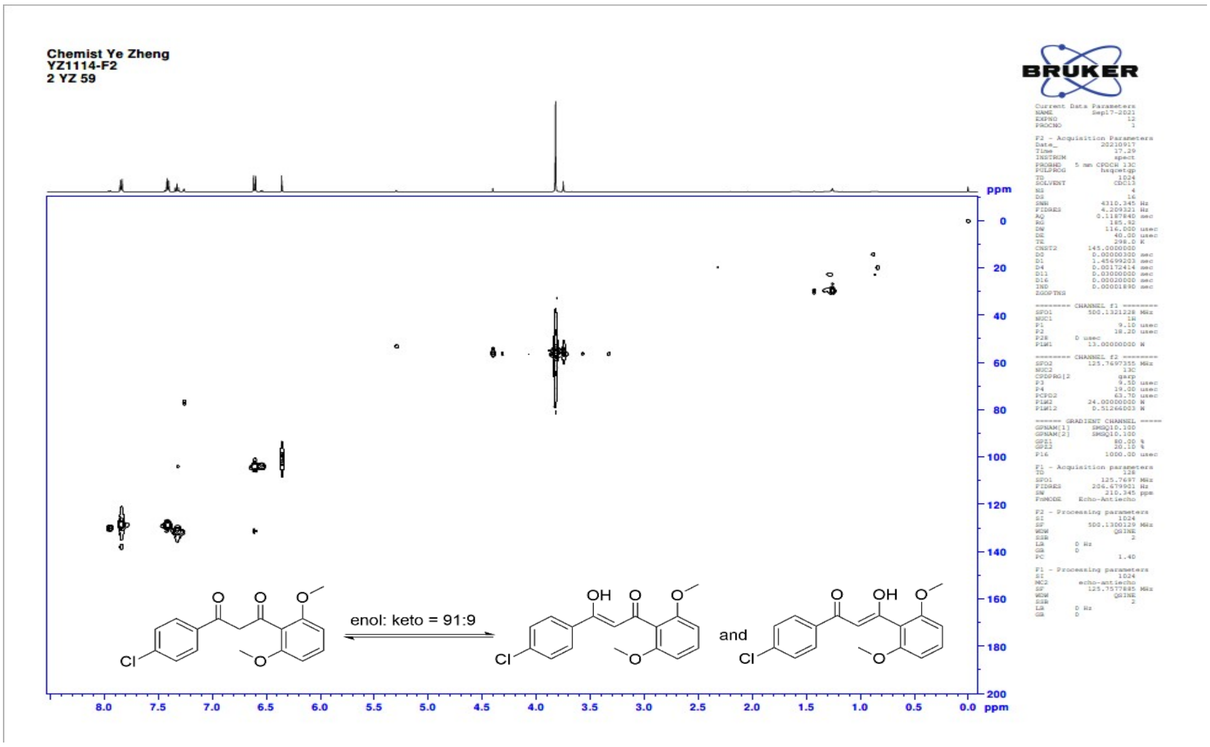

HMBC (500 MHz, $CDCl_3$) of of 3-(4-chlorophenyl)-1-(2,6-dimethoxyphenyl)-3-hydroxypropan-1-one **8b.**

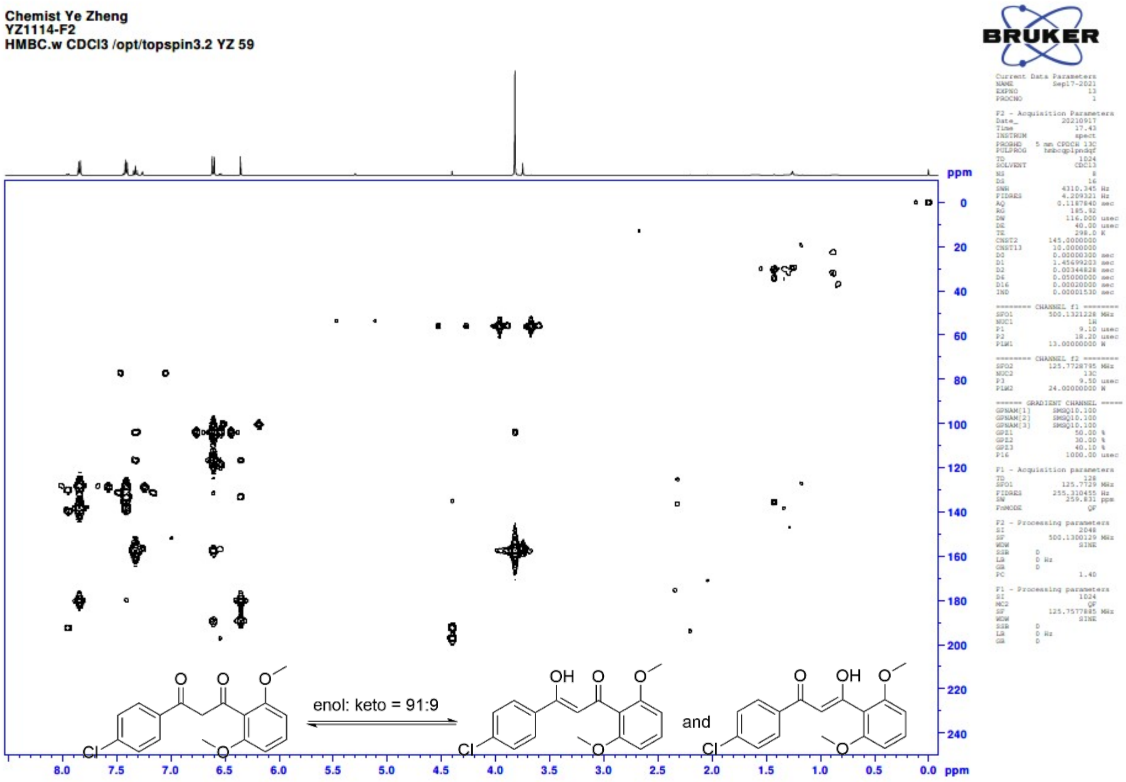

HPLC of 1-(4-chlorophenyl)-3-(2,6-dimethoxyphenyl)propane-1,3-dione **8a.**

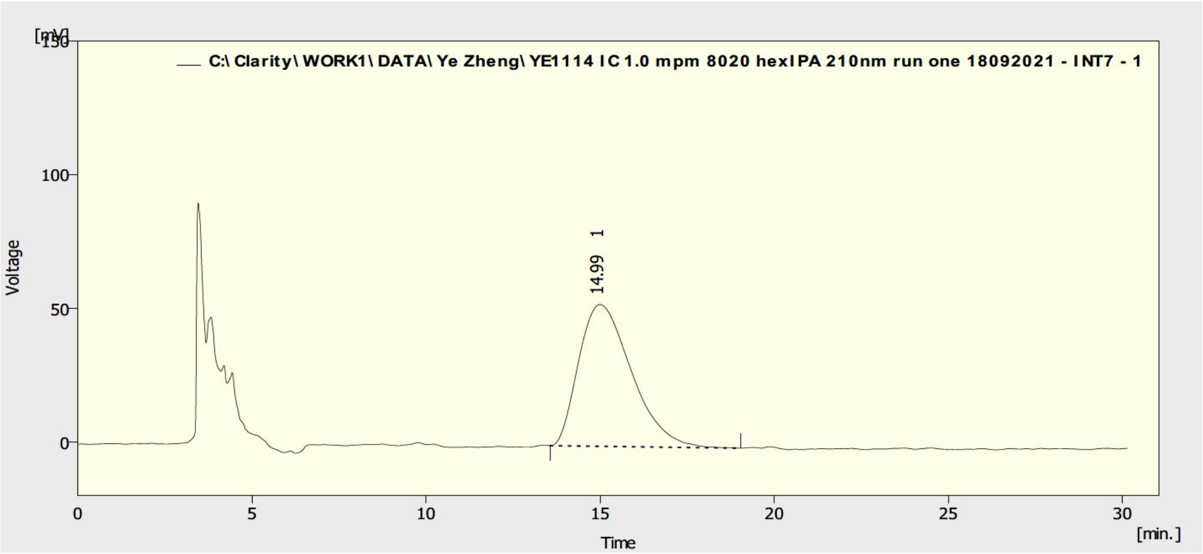

Result Table (Uncal - C:\Clarity\WORK1\DATA\Ye Zheng\YE1114 IC 1.0 mpm 8020 hexIPA 210nm run one 18092021 - INT7 - 1)

	Reten. Time [min]	Area [mV.s]	Height [mV]	Area [%]	Height [%]	W05 [min]	Compound Name
1	14.993	5582.022	53.035	100.0	100.0	1.65	
	Total	5582.022	53.035	100.0	100.0		

3-(4-Chlorophenyl)-1-(2,6-dimethoxyphenyl)-3-hydroxypropan-1-one 8b.

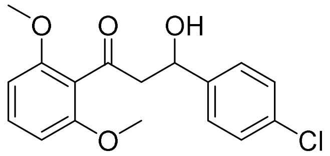 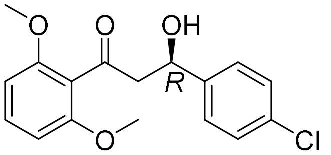

This compound is novel.

Synthesis of a racemic standard: (*R,R*)-3C-Tethered Ru(II)-TsDPEN catalyst (1.2 mg, 1.9 mmol, 0.5 mol%) and (*S,S*)-3C-tethered Ru(II)-TsDPEN catalyst (1.2 mg, 1.9 mmol, 0.5 mol%) were added to FA: TEA (5:2 azeotropic mixture, 0.54 mL) at rt and the mixture was stirred under a nitrogen atmosphere for 15 minutes; after which a solution of 1-(4-chlorophenyl)-3-(2,6-dimethoxyphenyl)propane-1,3-dione **8a** (120 mg, 0.377 mmol) in DCM (0.75 mL) was added. The reaction mixture was stirred under a nitrogen atmosphere and followed by TLC (4:1 hexane: EtOAc). After 24 h, the reaction was quenched using saturated $NaHCO_3$ solution (20 mL). EtOAc (20 mL) and the organic layer was separated. The aqueous layer was extracted with EtOAc (3 x 20 mL) and the combined organic layers were dried ($MgSO_4$) and filtered. The solvent was removed to give the crude product. The product was isolated via flash chromatography on silica eluted with 0-50% EtOAc in hexane to give 3-(4-chlorophenyl)-1-(2,6-dimethoxyphenyl)-3-hydroxypropan-1-one **8b** as a colorless oil (93.0 mg, 0.291 mmol, 77%). TLC: Rf ca 0.20 (4:1 hexane: EtOAc), strong UV and $KMnO_4$; HRMS (ESI+): *m/z:* [M+H]+, Calcd for $C_{17}H_{17}{}^{35}ClNaO_4$ 343.0703; Found 343.0708; 1.4 ppm error; ν_{max} 3474 (br), 3006, 2941, 2838, 1693, 1591, 1491, 1470, 1432, 1280, 1251, 1107, 1011, 985, 778, 734, 720 cm^{-1}; ^1H NMR (400 MHz, $CDCl_3$) δ 7.31 (5H, m, ArH), 6.57 (2H, t, J = 8.4, ArH), 5.27 (1H, dt, J = 9.0, 2.7, ArCH), 3.79 (6H, s, OCH_3), 3.64 (1H, d, J = 2.8, OH), 3.20 (1H, dd, J = 17.5, 3.1, CH_2), 3.11 (1H, dd, J = 17.5, 9.1, CH_2); $^{13}C\{^1H\}$ NMR (100 MHz, $CDCl_3$) δ 205.1 (C), 156.8 (C), 142.0 (C), 133.1 (C), 131.4 (CH), 128.5 (CH), 127.3 (CH), 119.5 (C), 104.1 (CH), 69.5 (CH), 56.0 (CH_3), 53.2 (CH_2) ppm; *m/z* (ES-API+) 343.1 (M^+ + 23, 100%).

Enantiomeric excess and conversion determined by HPLC analysis (Chiralpak IC, 30 cm x 6 mm column, hexane:iPrOH 80:20, 1.0 mL/min, T = 25°C) ketone 15.0 min, *R* isomer 24.7 min and *S* isomer 27.9 min.

(*R*)-3-(4-Chlorophenyl)-1-(2,6-dimethoxyphenyl)-3-hydroxypropan-1-one **8b.**

(*R,R*)-3C-tethered Ru(II)-TsDPEN catalyst (2.3 mg, 3.8 mmol, 1 mol%) was added to FA: TEA (5:2 azeotropic mixture, 0.54 mL) at rt and the mixture was stirred under a nitrogen atmosphere for 15 minutes; after which a solution of 1-(4-chlorophenyl)-3-(2,6-dimethoxyphenyl)propane-1,3-dione **8a** (120 mg, 0.377 mmol) in DCM (0.75 mL) was added. The reaction mixture was stirred under a nitrogen atmosphere and followed by TLC (4:1 hexane: EtOAc). After 24 h, the reaction was quenched using saturated $NaHCO_3$ solution (20 mL). EtOAc (20 mL) and the organic layer was separated. The aqueous layer was extracted with EtOAc (3 x 20 mL) and the combined organic layers were dried ($MgSO_4$) and filtered. The solvent was removed to give the crude product. The product was isolated via flash chromatography on silica gel eluted with 0-50% EtOAc in hexane to give (*R*)-3-(4-chlorophenyl)-1-(2,6-dimethoxyphenyl)-3-hydroxypropan-1-one **8b** as a colorless oil (84.0 mg, 0.263 mmol, 70%). The reaction was also followed by HPLC (Chiralpak IC, 30 cm x 6 mm column, hexane:iPrOH 80:20, 1.0 mL/min, T = 25°C): 100% conversion; $[\alpha]_D^{26}$ +22.6 (c 0.420 in $CHCl_3$) 96% ee (*R*).

^1H NMR (400 MHz, $CDCl_3$) of 3-(4-chlorophenyl)-1-(2,6-dimethoxyphenyl)-3-hydroxypropan-1-one **8b.**

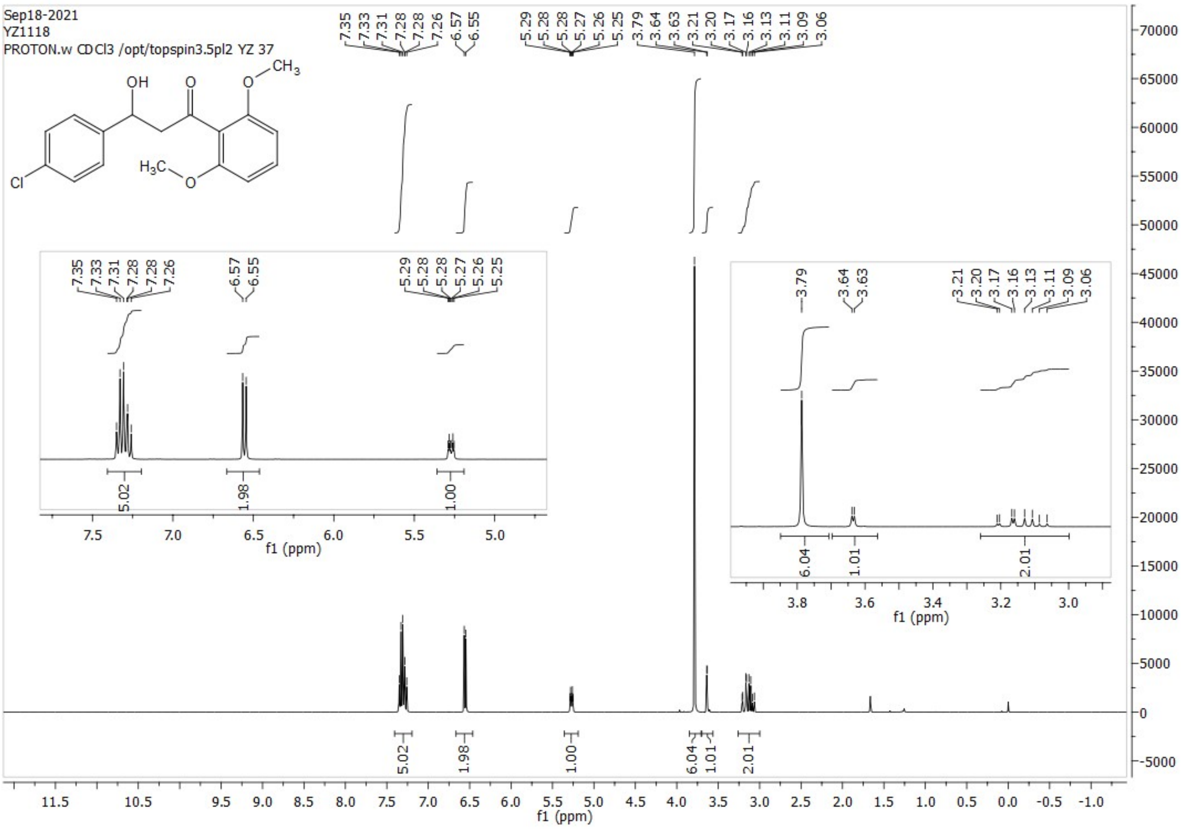

$^{13}C\{^{1}H\}$ NMR (100 MHz, $CDCl_3$) of of 3-(4-chlorophenyl)-1-(2,6-dimethoxyphenyl)-3-hydroxypropan-1-one **8b.**

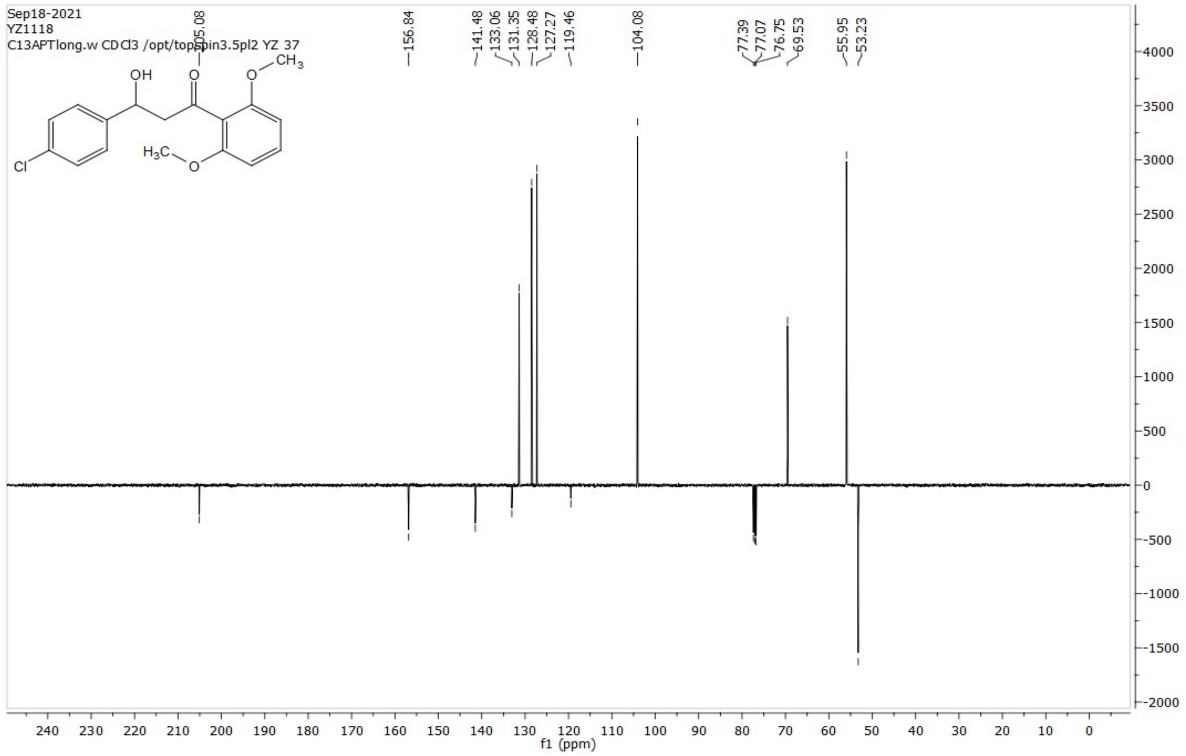

COSY (400 MHz, $CDCl_3$) of of 3-(4-chlorophenyl)-1-(2,6-dimethoxyphenyl)-3-hydroxypropan-1-one **8b.**

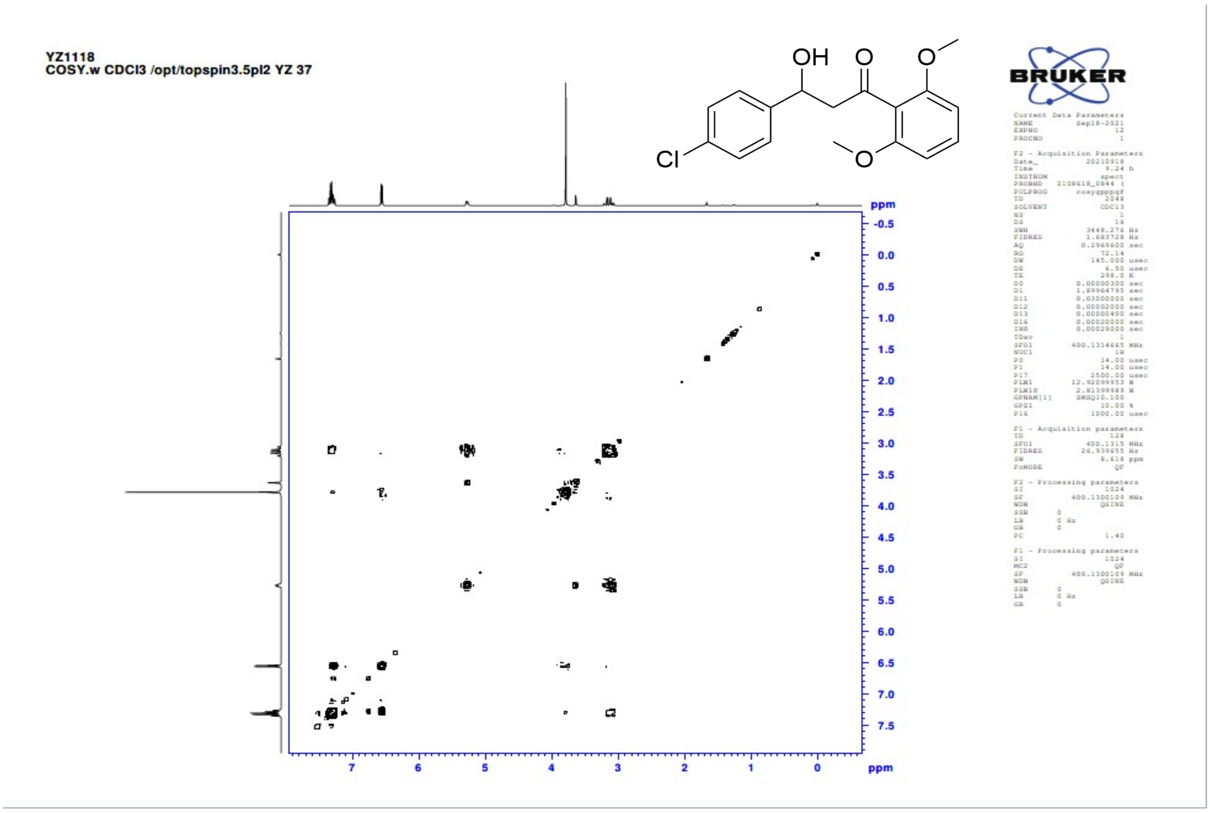

HSQC (400 MHz, $CDCl_3$) of 3-(4-chlorophenyl)-1-(2,6-dimethoxyphenyl)-3-hydroxypropan-1-one **8b.**

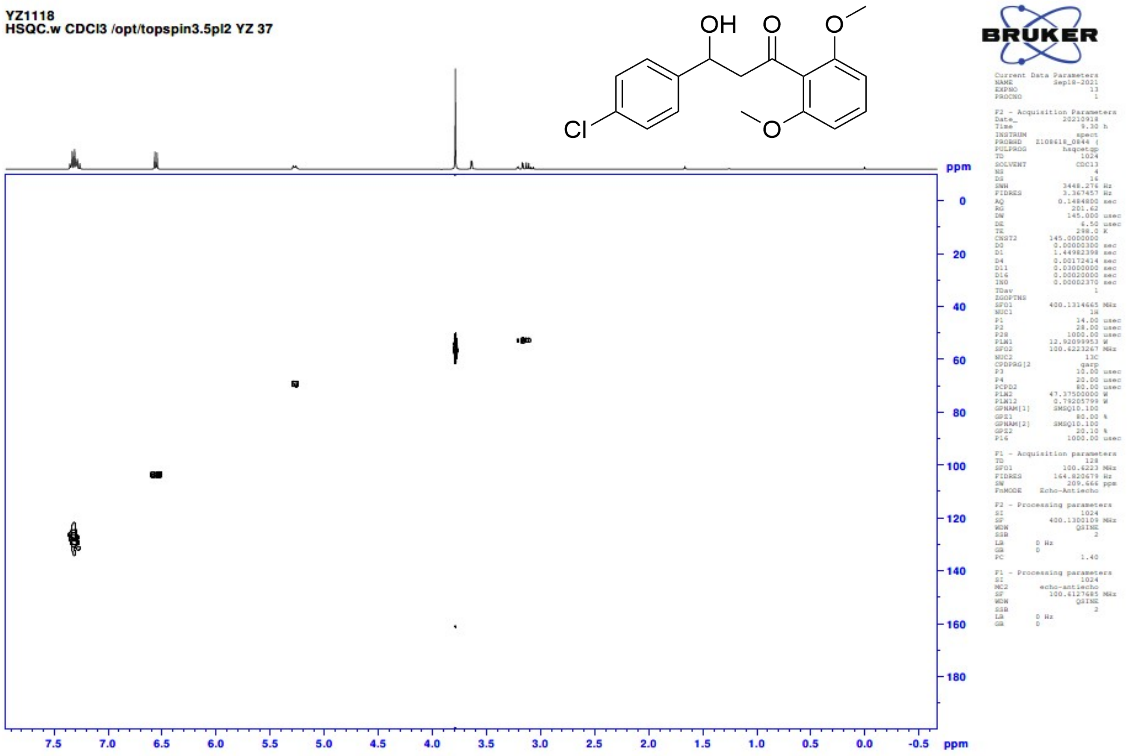

HMBC (400 MHz, $CDCl_3$) of 3-(4-chlorophenyl)-1-(2,6-dimethoxyphenyl)-3-hydroxypropan-1-one **8b.**

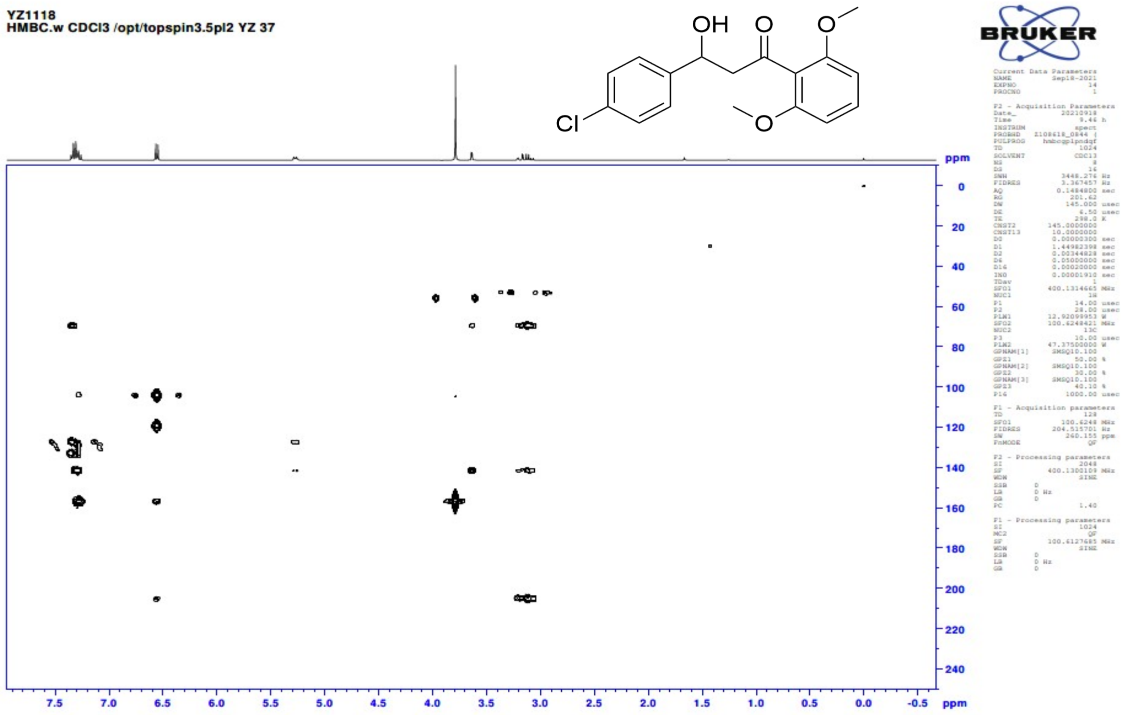

HPLC of racemic 3-(4-chlorophenyl)-1-(2,6-dimethoxyphenyl)-3-hydroxypropan-1-one **8b**.

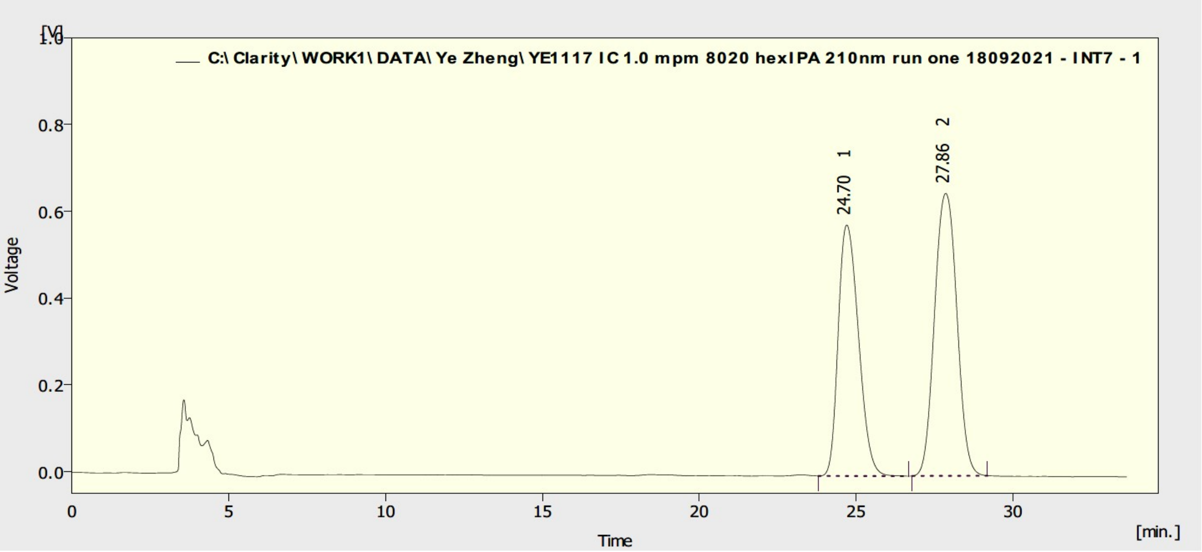

Result Table (Uncal - C:\Clarity\WORK1\DATA\Ye Zheng\YE1117 IC 1.0 mpm 8020 hexIPA 210nm run one 18092021 - INT7 - 1)

	Reten. Time [min]	Area [mV.s]	Height [mV]	Area [%]	Height [%]	W05 [min]	Compound Name
1	24.703	26079.857	578.401	44.7	47.0	0.71	
2	27.860	32257.691	651.161	55.3	53.0	0.79	
	Total	58337.548	1229.562	100.0	100.0		

HPLC *of* (*R*)-3-(4-chlorophenyl)-1-(2,6-dimethoxyphenyl)-3-hydroxypropan-1-one **8b**. ((*R,R*)-3C-tethered Ru(II) catalyst **2** (24 h, 100% conversion, 96% ee, (*R*)).

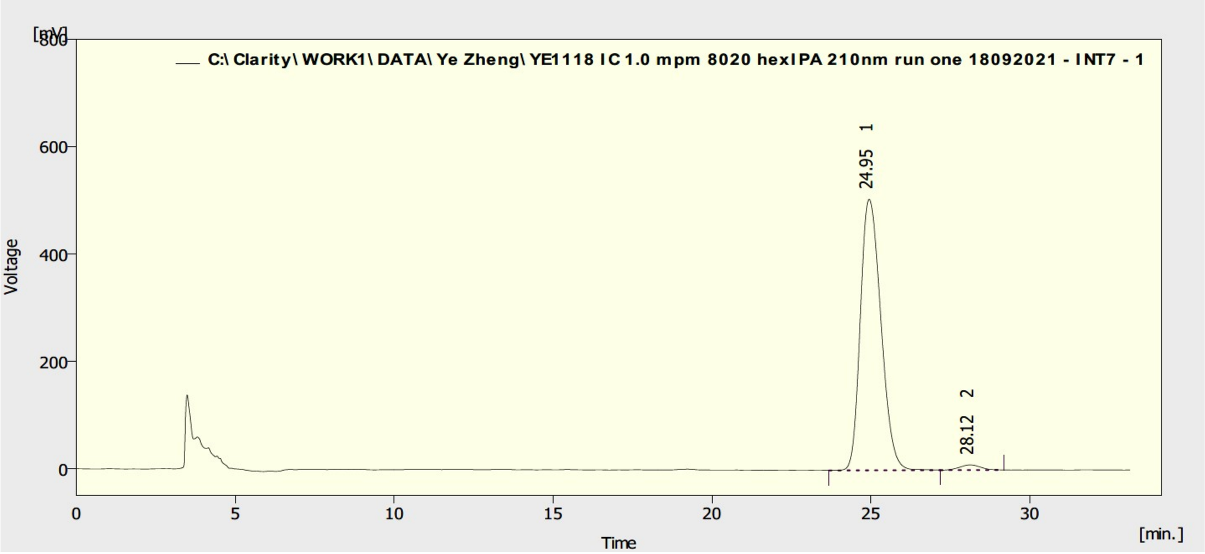

Result Table (Uncal - C:\Clarity\WORK1\DATA\Ye Zheng\YE1118 IC 1.0 mpm 8020 hexIPA 210nm run one 18092021 - INT7 - 1)

	Reten. Time [min]	Area [mV.s]	Height [mV]	Area [%]	Height [%]	W05 [min]	Compound Name
1	24.953	22730.794	505.105	98.1	98.1	0.71	
2	28.123	442.447	9.691	1.9	1.9	0.71	
	Total	23173.242	514.796	100.0	100.0		

X-ray Crystallography Data for (*R*)-8b. CCDC Deposition Number 2276988.

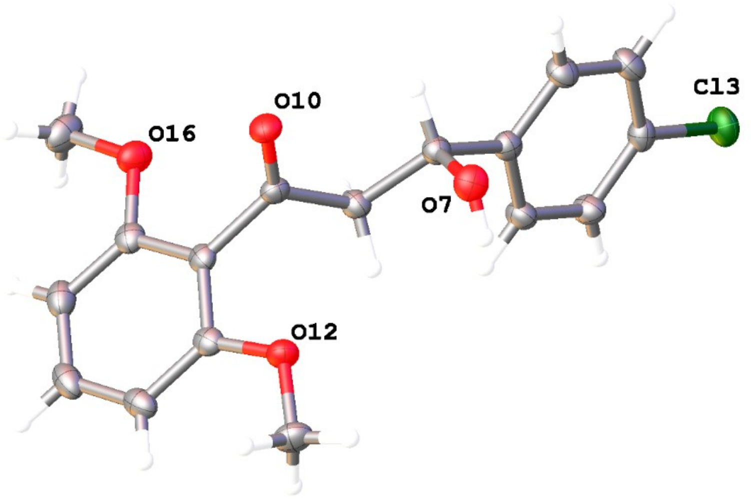

*solid state structure of **8b** with only key atoms labelled and thermal ellipsoids drawn at 50% probability level*

Crystal structure determination of 8b.

The asymmetric unit contains the molecule, there are two in the unit cell.

The OH was located in a difference map but refined with restraints.

It forms a short contact tabulated below

Specified hydrogen bonds (with esds except fixed and riding H)

D-H	H...A	D...A	<(DHA)	
0.84	2.23	3.0414(18)	163.5	O7-H7A...O10_$1

Symmetry operator used to define symmetry related atom in above contact was $1 1+X,+Y,+Z.

The Flack (and associated Hooft y) parameter is small with a small error so you can be confident in the assignment of the handedness of the crystal measured.

Flack x: -0.003(3) Shelx2018

Hooft y: -0.005(2) Olex2

Experimental

Single crystals of $C_{17}H_{17}ClO_4$ **(8b)** were grown from DCM/Hexane. A suitable crystal was selected and mounted on a glass fibre with Fomblin oil and placed on a Rigaku Oxford Diffraction SuperNova diffractometer with a dual source (Cu at zero)

equipped with an AtlasS2 CCD area detector. The crystal was kept at 150(2) K during data collection. Using Olex2 [1], the structure was solved with the SHELXT [2] structure solution program using Intrinsic Phasing and refined with the SHELXL [3] refinement package using Least Squares minimisation.

1. Dolomanov, O.V., Bourhis, L.J., Gildea, R.J, Howard, J.A.K. & Puschmann, H. (2009), J. Appl. Cryst. 42, 339-341.
2. Sheldrick, G.M. (2015). Acta Cryst. A71, 3-8.
3. Sheldrick, G.M. (2015). Acta Cryst. C71, 3-8.

Crystal Data for $C_{17}H_{17}ClO_4$ (M =320.75 g/mol): monoclinic, space group $P2_1$ (no. 4), a = 5.09612(4) Å, b = 13.00130(9) Å, c = 11.56297(8) Å, β = 99.9243(8)°, V = 754.654(10) Å^3, Z = 2, T = 150(2) K, μ(Cu Kα) = 2.384 mm^{-1}, $Dcalc$ = 1.412 g/cm^3, 24755 reflections measured (7.762° ≤ 2Θ ≤ 146.806°), 3044 unique (R_{int} = 0.0244, R_{sigma} = 0.0120) which were used in all calculations. The final R_1 was 0.0217 (I > 2σ(I)) and wR_2 was 0.0585 (all data).

**Table 1 Crystal data and structure refinement for 8b.**	
Identification code (local)	yz16
Empirical formula	$C_{17}H_{17}ClO_4$
Formula weight	320.75
Temperature/K	150(2)
Crystal system	Monoclinic
Space group	$P2_1$
a/Å	5.09612(4)
b/Å	13.00130(9)
c/Å	11.56297(8)
α/°	90
β/°	99.9243(8)
γ/°	90
Volume/Å^3	754.654(10)
Z	2
ρ_{calc}g/cm^3	1.412

μ/mm^{-1}	2.384
F(000)	336.0
Crystal size/mm^3	0.3 × 0.16 × 0.12 colorless block
Radiation	Cu Kα (λ = 1.54184)
2Θ range for data collection/°	7.762 to 146.806
Index ranges	-5 ≤ h ≤ 6, -16 ≤ k ≤ 16, -14 ≤ l ≤ 14
Reflections collected	24755
Independent reflections	3044 [R_{int} = 0.0244, R_{sigma} = 0.0120]
Data/restraints/parameters	3044/1/202
Goodness-of-fit on F^2	1.033
Final R indexes [I>=2σ (I)]	R_1 = 0.0217, wR_2 = 0.0585
Final R indexes [all data]	R_1 = 0.0217, wR_2 = 0.0585
Largest diff. peak/hole / e Å^{-3}	0.15/-0.15
Flack parameter	-0.003(3)

1-(2,6-Dimethoxyphenyl)-3-phenylpropane-1,3-dione 9a.

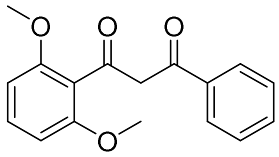

This compound is novel.

To a solution of sodium hydride (360 mg, 60% dispersion in mineral oil, 15.0 mmol) in THF (6 mL) at 0 °C was added dropwise a solution of 1-(2,6-dimethoxyphenyl)ethan-1-one **5** (540 mg, 3.00 mmol) in THF (6 mL). The reaction mixture was stirred under a nitrogen atmosphere at 0 °C for 30 min and then stirred under a nitrogen atmosphere at rt for 30 min, after which ethyl benzoate (2.25 g, 15.0 mmol) was added dropwise. The reaction mixture was heated to 65 °C and left stirring under the nitrogen atmosphere overnight. The reaction was followed by TLC (4:1 hexane: EtOAc). The mixture was quenched by 2M HCl solution (20 mL). EtOAc (20 mL) was added and the organic layer was separated. The aqueous layer was extracted with EtOAc (3 × 20 mL), and the combined organic layers were washed with saturated $NaHCO_3$ solution (2 × 20 mL) and brine (20 mL), dried ($MgSO_4$) and filtered. Solvent was removed to give the crude product. The product was isolated via flash chromatography on silica eluted with 0-20% EtOAc in hexane to give 1-(2,6-dimethoxyphenyl)-3-phenylpropane-1,3-dione **9a** as a yellow oil (708 mg, 2.50 mmol, 83%). TLC: Rf ca 0.50 (4:1 hexane: EtOAc), strong UV and $KMnO_4$; HRMS (ESI+) *m/z*: [M+H]+, Calcd for $C_{17}H_{16}NaO_4$ 307.0935; Found 307.0941; 2.0 ppm error; ν_{max} 3068, 2939, 2838, 1681, 1593, 1471, 1321, 1290, 1107, 1024, 770 cm^{-1}; enol: keto = 9:1; ^1H NMR (400 MHz, $CDCl_3$): δ 7.95 (2H, d, J = 7.3, ArH), 7.60-7.45 (3H, m, ArH), 7.37-7.26 (1H, m, ArH), 6.65 (2H, d, J = 8.4, ArH), 6.43 (0.9H, s, CH of enol form), 4.46 (0.2H, s, CH_2 of keto form), 3.85 (5.4H, s, OCH_3 of enol form), 3.76 (0.6H, s, OCH_3 of keto form); $^{13}C\{^1H\}$ NMR (100 MHz, $CDCl_3$): δ keto form: 189.5 (C), 183.1 (C), 157.0 (C), 135.0 (C), 133.2 (CH), 131.6 (CH), 128.9 (CH), 128.5 (CH), 118.4 (C), 104.0 (CH), 56.0 (CH_2), 55.8 (CH_3) ppm; enol form: 189.5 (C), 181.5 (C), 157.7 (C), 135.0 (C), 132.1 (CH), 131.3 (CH), 128.6 (CH), 127.1 (CH), 117.1 (C), 104.2 (CH), 100.6 (CH), 56.1 (CH_3); *m/z* (ES-API+) 307.1 (M^+ + 23, 100%).

Enantiomeric excess and conversion determined by HPLC analysis (Chiralpak IB, 30 cm x 6 mm column, hexane:iPrOH 90:10, 1.0 mL/min, T = 25°C) ketone 10.0 min, *R* and *S* isomers 17.8 min and 20.3 min, configuration assigned by analogy.

^{1}H NMR (400 MHz, $CDCl_3$) of 1-(2,6-dimethoxyphenyl)-3-phenylpropane-1,3-dione **9a.**

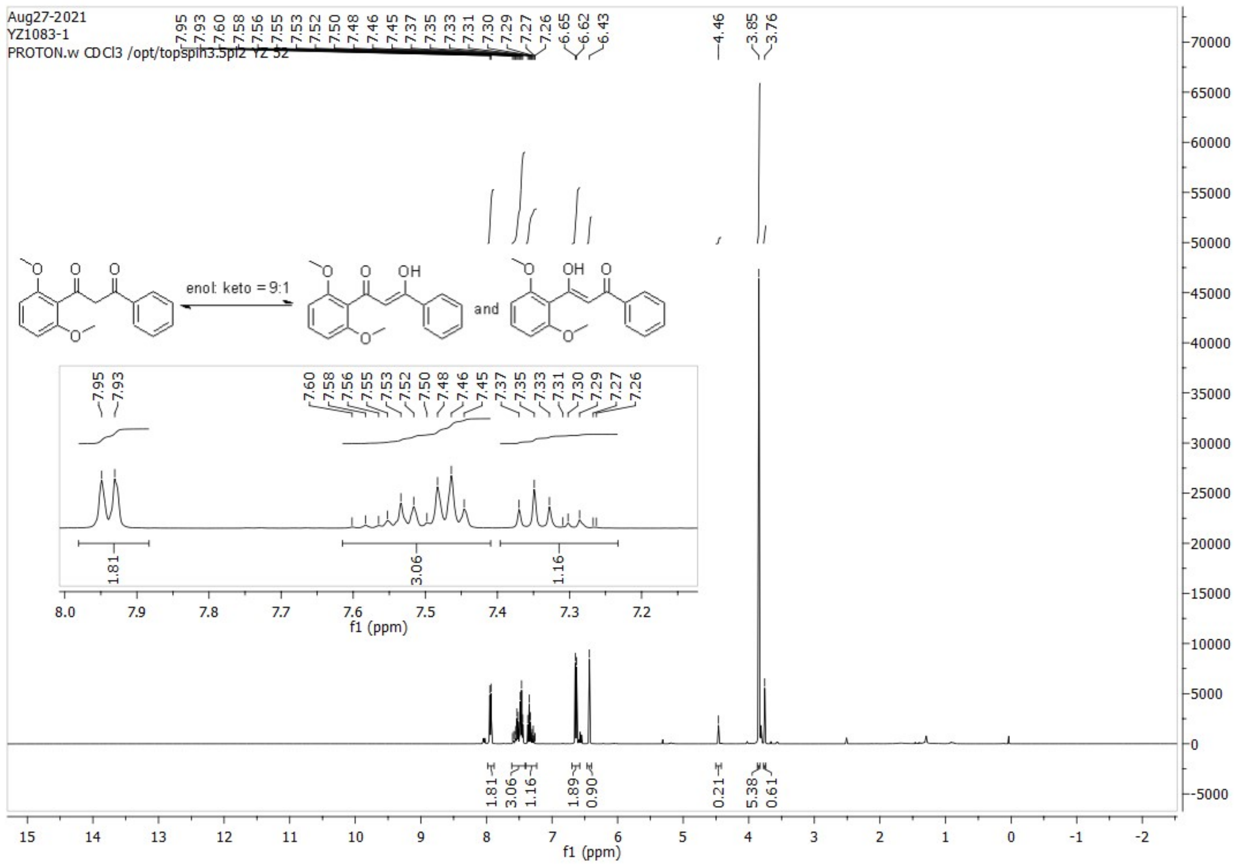

$^{13}C\{^{1}H\}$ NMR (100 MHz, $CDCl_3$) of 1-(2,6-dimethoxyphenyl)-3-phenylpropane-1,3-dione **9a.**

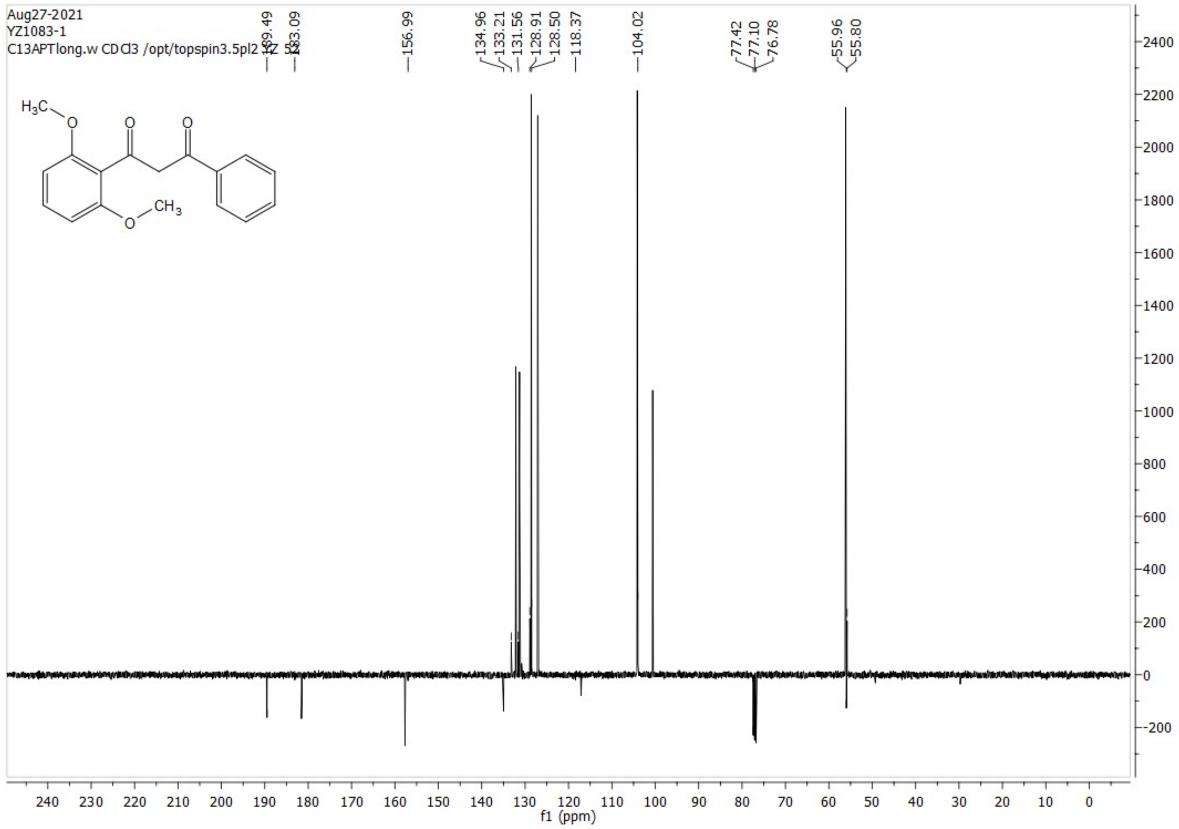

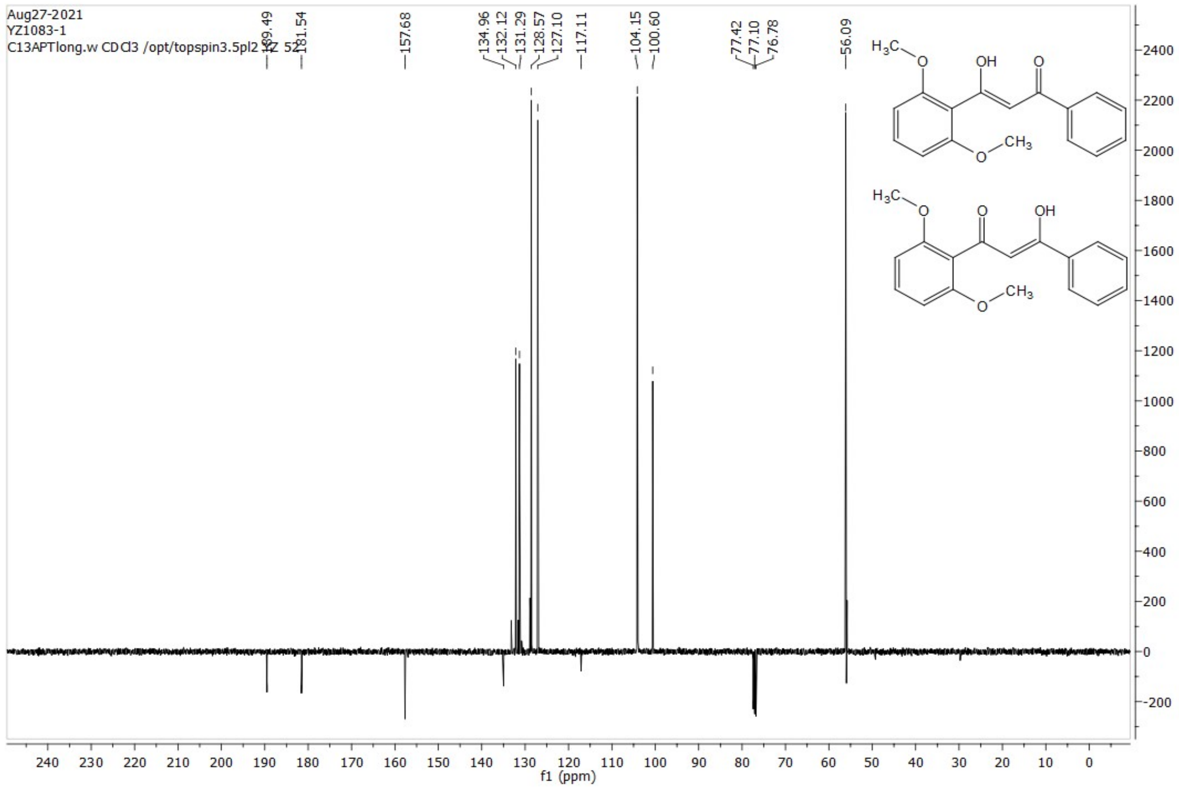

COSY (400 MHz, $CDCl_3$) of 1-(2,6-dimethoxyphenyl)-3-phenylpropane-1,3-dione **9a.**

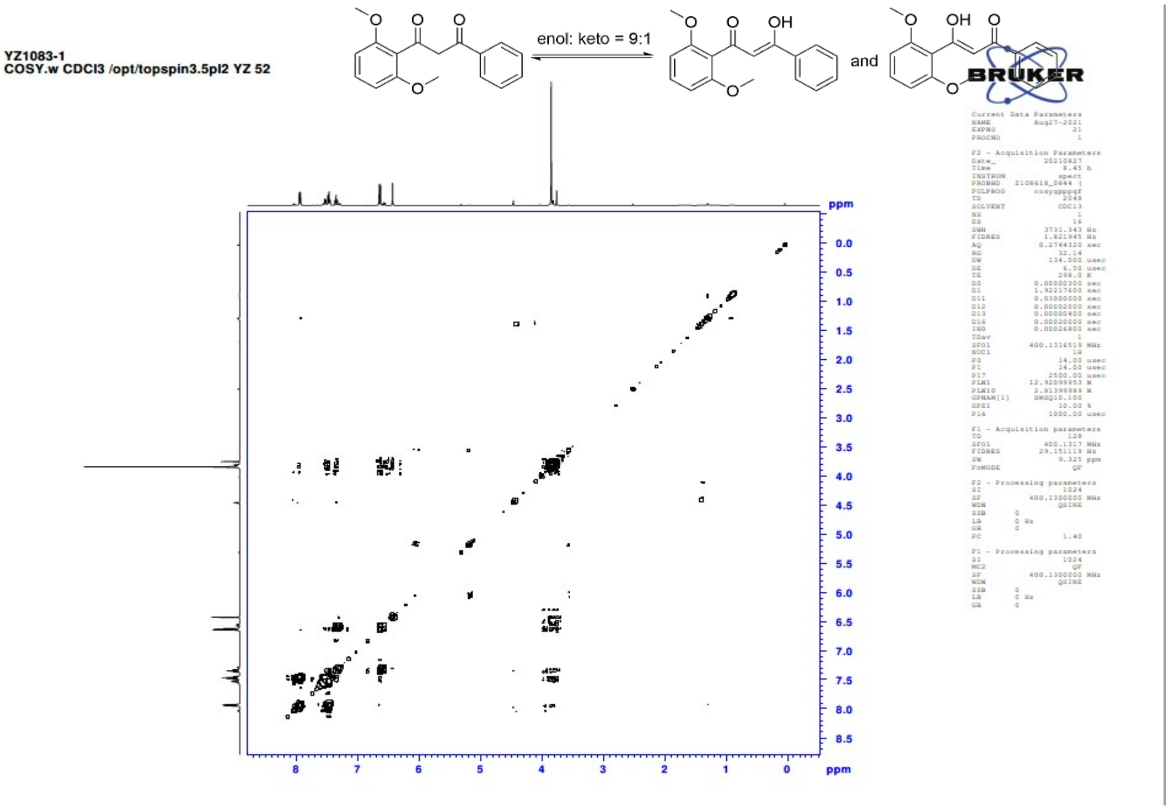

HSQC (400 MHz, $CDCl_3$) of 1-(2,6-dimethoxyphenyl)-3-phenylpropane-1,3-dione **9a.**

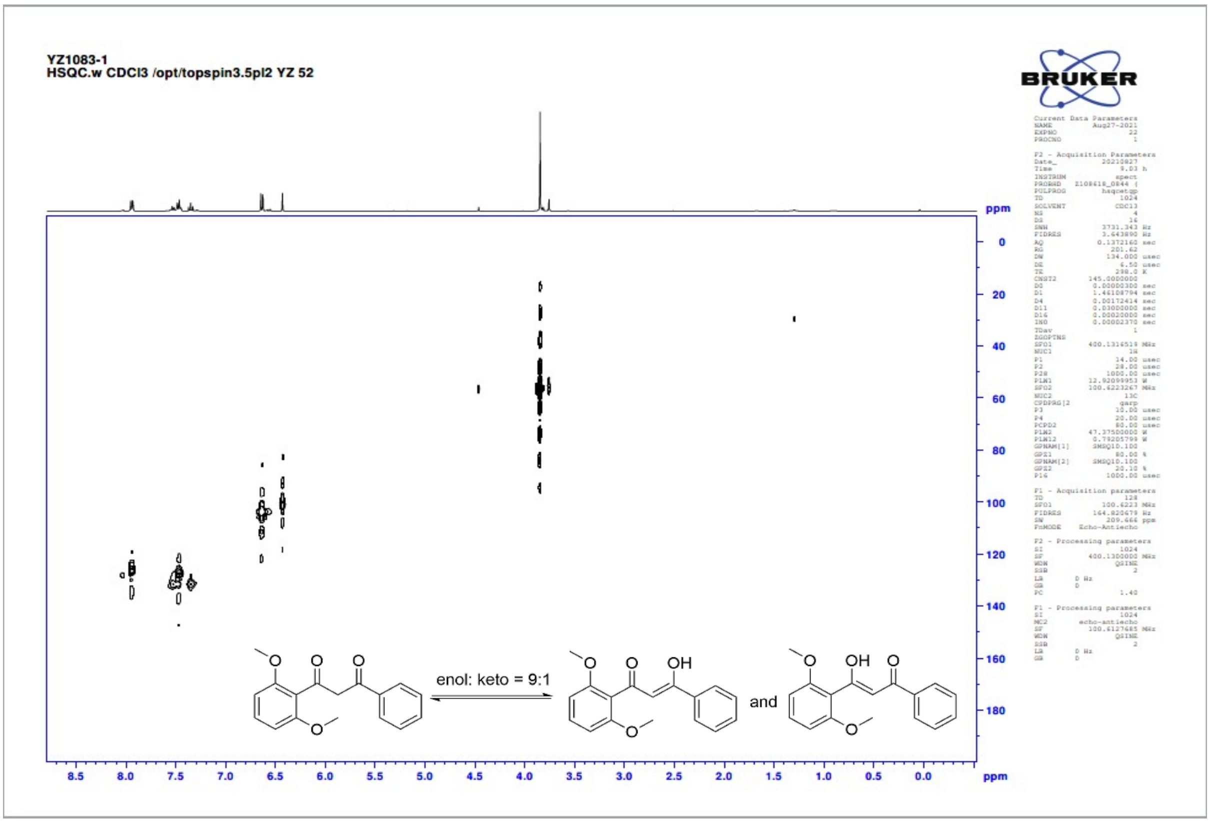

HMBC (400 MHz, $CDCl_3$) of 1-(2,6-dimethoxyphenyl)-3-phenylpropane-1,3-dione **9a.**

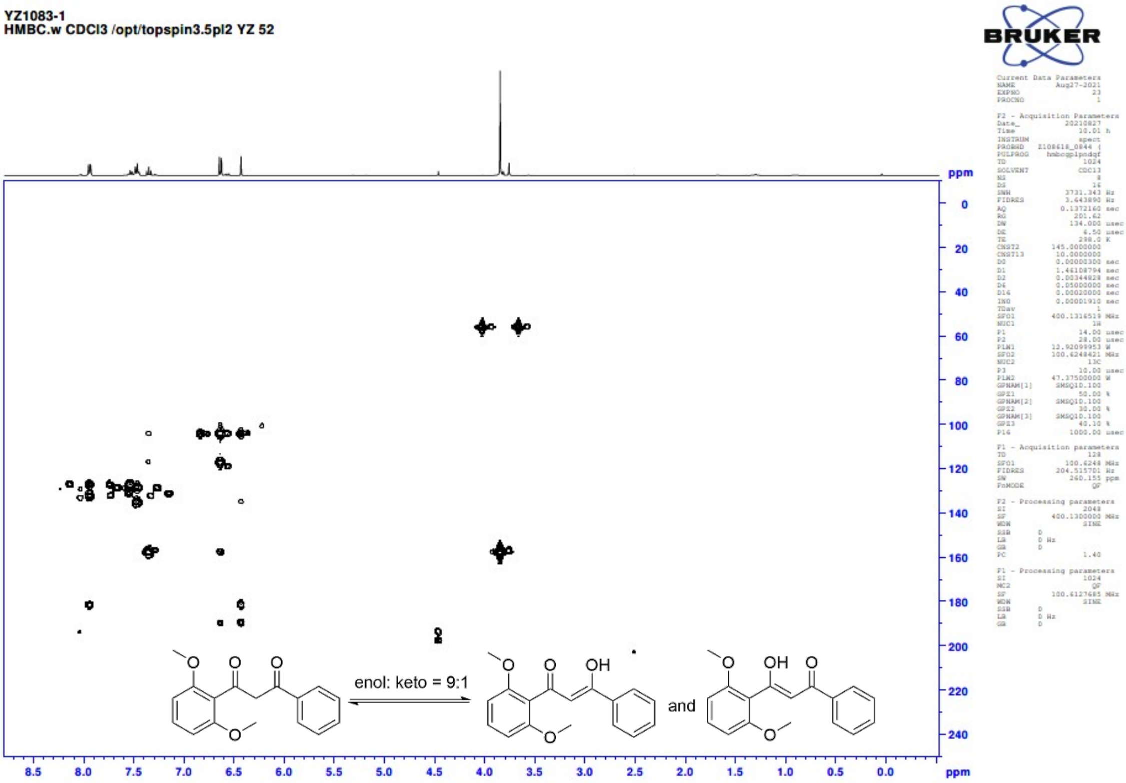

HPLC of 1-(2,6-dimethoxyphenyl)-3-phenylpropane-1,3-dione **9a.**

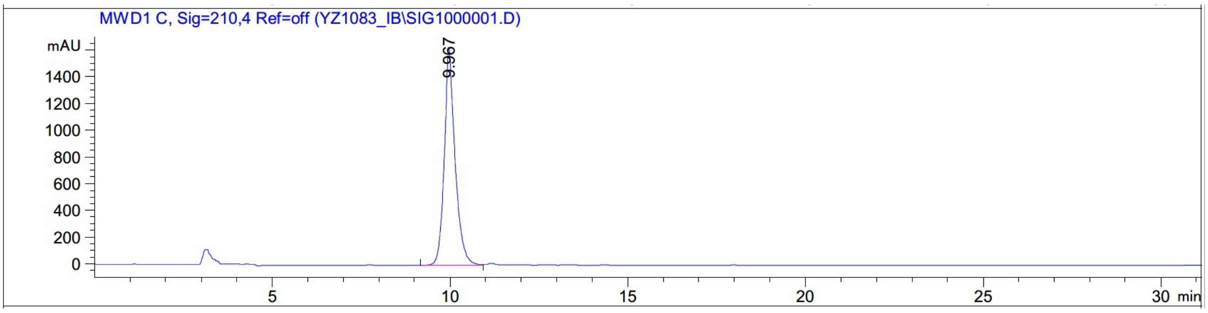


```
Signal 2: MWD1 C, Sig=210,4 Ref=off

Peak RetTime Type  Width     Area      Height     Area
  #    [min]        [min]  [mAU*s]     [mAU]        %
----|-------|----|-------|----------|----------|--------|
   1   9.967 BV    0.2954 3.42879e4 1629.91284 100.0000

Totals :                  3.42879e4  1629.91284
```

1-(2,6-Dimethoxyphenyl)-3-hydroxy-3-phenylpropan-1-one 9b.

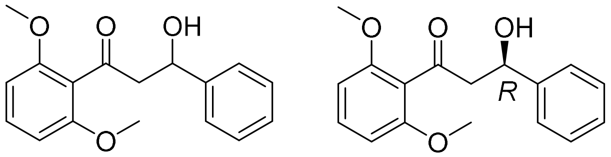

This compound is novel.

Synthesis of a racemic standard: (*R,R*)-3C-Tethered Ru(II)-TsDPEN catalyst (0.44 mg, 0.71 mmol, 0.5 mol%) and (*S,S*)-3C-tethered Ru(II)-TsDPEN catalyst (0.44 mg, 0.71 mmol, 0.5 mol%) were added to FA: TEA (5:2 azeotropic mixture, 0.18 mL) at rt and the mixture was stirred under a nitrogen atmosphere for 15 minutes; after which a solution of 1-(2,6-dimethoxyphenyl)-3-phenylpropane-1,3-dione **9a** (40.0 mg, 0.141 mmol) in DCM (0.25 mL) was added. The reaction mixture was stirred under a nitrogen atmosphere and followed by TLC (3:2 hexane: EtOAc). After 24 h, the reaction was quenched using saturated $NaHCO_3$ solution (20 mL). EtOAc (20 mL) was added and the organic layer was separated. The aqueous layer was extracted with EtOAc (3 x 20 mL) and the combined organic layers were dried ($MgSO_4$) and filtered. The solvent was removed to give the crude product. The product was isolated via flash chromatography on silica eluted with 25-100% EtOAc in hexane to give 1-(2,6-dimethoxyphenyl)-3-hydroxy-3-phenylpropan-1-one **9b** as a colorless oil (29.0 mg, 0.101 mmol, 72%). TLC: Rf ca 0.20 (3:2 hexane: EtOAc), strong UV and $KMnO_4$; HRMS (ESI+) *m/z*: [M+H]+ Calcd for $C_{17}H_{18}NaO_4$ 309.1099; Found 309.1097; -0.5 ppm error; ν_{max} 3460 (br), 3005, 2940, 2838, 1692, 1591, 1470, 1432, 1286, 1250, 1105, 1056, 1016, 983 cm^{-1}; ^1H NMR (500 MHz, $CDCl_3$): δ 7.38 (2H, d, J = 7.5, ArH), 7.30 (2H, t, J = 7.5, ArH), 7.25-7.21 (2H, m, ArH), 6.53 (2H, d, J = 8.4, ArH), 5.27 (1H, dt, J = 9.3, 2.7, ArCH), 3.75 (6H, s, OCH_3), 3.56 (1H, d, J = 2.7, OH), 3.20 (1H, dd, J = 17.5, 2.9, CH_2), 3.12 (1H, dd, J = 17.5, 9.4, CH_2); $^{13}C\{^1H\}$ NMR (125 MHz, $CDCl_3$): δ 205.4 (C), 156.8 (C), 142.9 (C), 131.3 (CH), 128.4 (CH), 127.5 (CH), 125.9 (CH), 119.6 (C), 104.1 (CH), 70.1 (CH), 55.9 (CH_3), 53.4 (CH_2); *m/z* (ES-API+) 309.1 (M^+ + 23, 100%).

Enantiomeric excess and conversion determined by HPLC analysis (Chiralpak IB, 30 cm x 6 mm column, hexane:iPrOH 90:10, 1.0 mL/min, T = 25°C) ketone 10.0 min, *R* and *S* isomers 17.8 min and 20.3 min, configuration assigned by analogy.

(*R*)-1-(2,6-Dimethoxyphenyl)-3-hydroxy-3-phenylpropan-1-one **9b**.

(*R,R*)-3C-tethered Ru(II)-TsDPEN catalyst (0.88 mg, 1.41 mmol, 1 mol%) was added to FA: TEA (5:2 azeotropic mixture, 0.18 mL) at rt and the mixture was stirred under a nitrogen atmosphere for 15 minutes; after which a solution of 1-(2,6-dimethoxyphenyl)-3-phenylpropane-1,3-dione **9a** (40.0 mg, 0.141 mmol) in DCM (0.25 mL) was added. The reaction mixture was stirred under a nitrogen atmosphere and followed by TLC (3:2 hexane: EtOAc). After 24 h, the reaction was quenched using saturated $NaHCO_3$ solution (20 mL). EtOAc (20 mL) was added and the organic layer was separated. The aqueous layer was extracted with EtOAc (3 x 20 mL) and the combined organic layers were dried ($MgSO_4$) and filtered. The solvent was removed to give the crude product. The product was isolated via flash chromatography on silica eluted with 25-100% EtOAc in hexane to give (*R*)-1-(2,6-dimethoxyphenyl)-3-hydroxy-3-phenylpropan-1-one **9b** as a colorless oil (32.0 mg, 0.112 mmol, 77%). The reaction was also followed by HPLC (Chiralpak IB, 30 cm x 6 mm column, hexane:iPrOH 90:10, 1.0 mL/min, T = 25°C): 100% conversion; $[\alpha]_D^{26}$ +31.5 (c 0.143 in $CHCl_3$) 98% ee (*R*).

^{1}H NMR (500 MHz, $CDCl_3$) of 1-(2,6-dimethoxyphenyl)-3-hydroxy-3-phenylpropan-1-one **9b.**

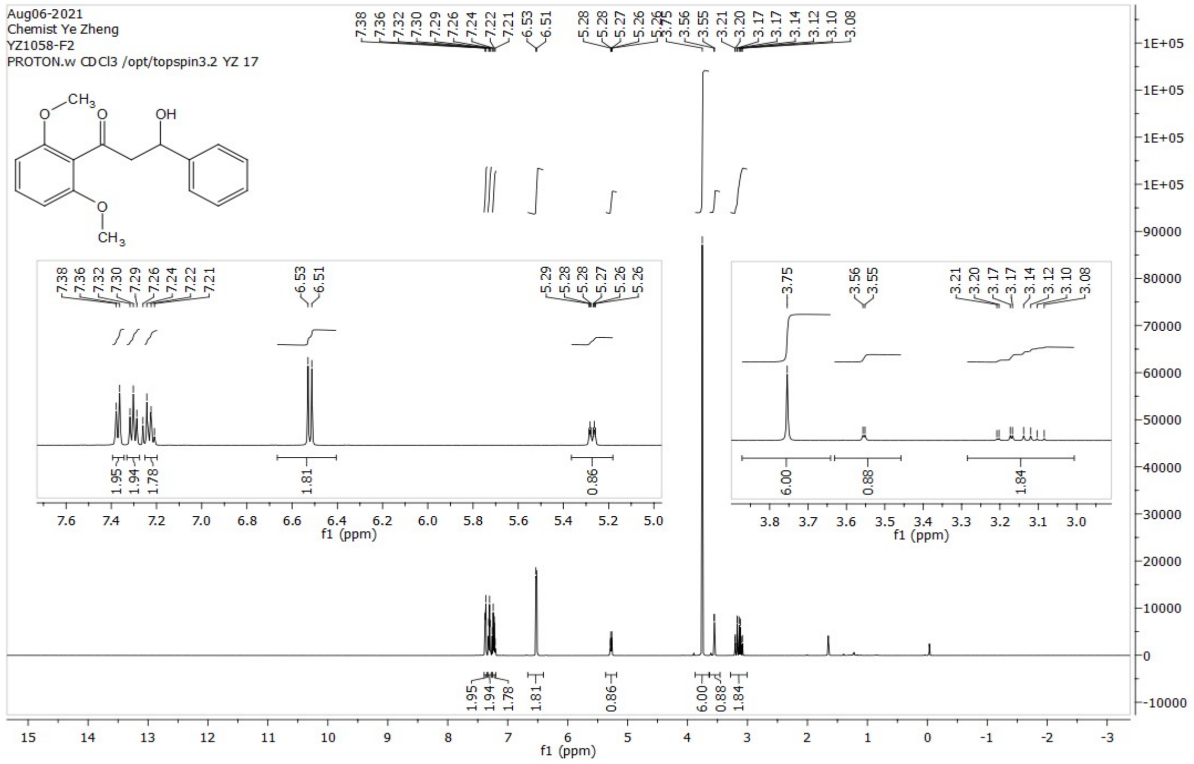

$^{13}C\{^{1}H\}$ NMR (125 MHz, $CDCl_3$) 1-(2,6-dimethoxyphenyl)-3-hydroxy-3-phenylpropan-1-one **9b**

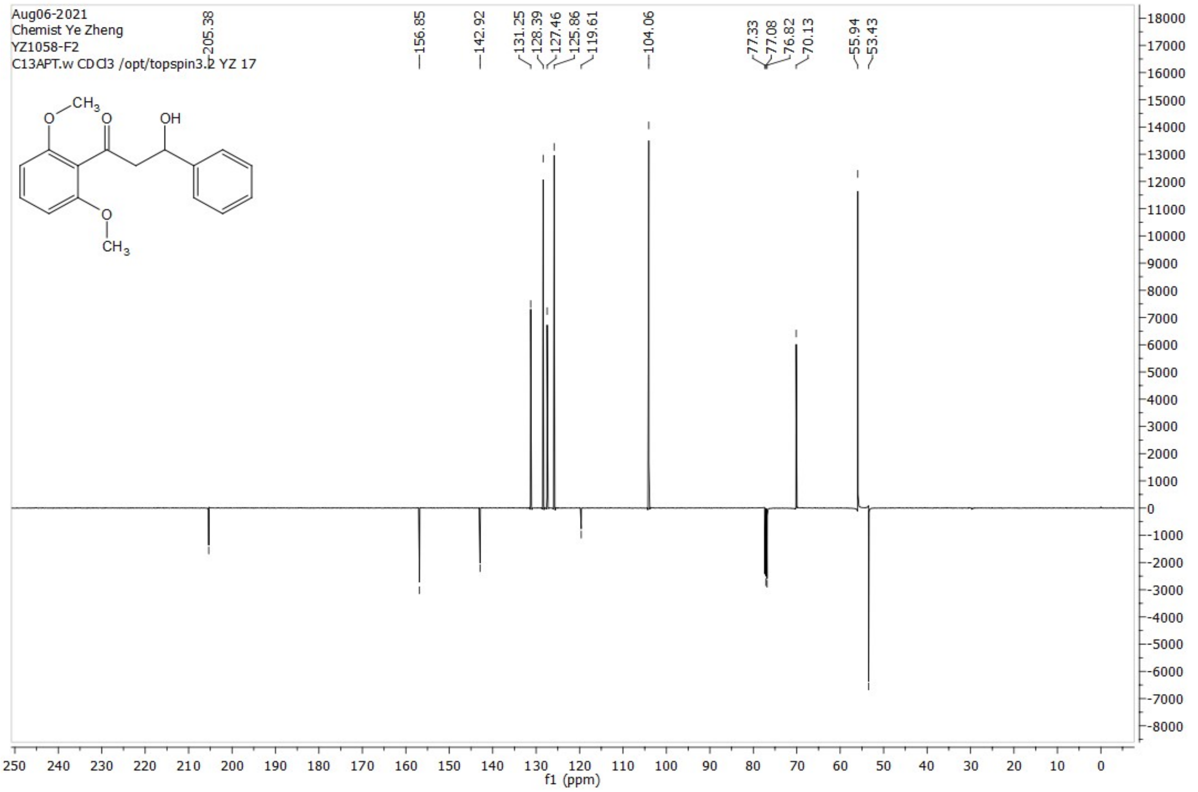

COSY (500 MHz, $CDCl_3$) of 1-(2,6-Dimethoxyphenyl)-3-hydroxy-3-phenylpropan-1-one **9b.**

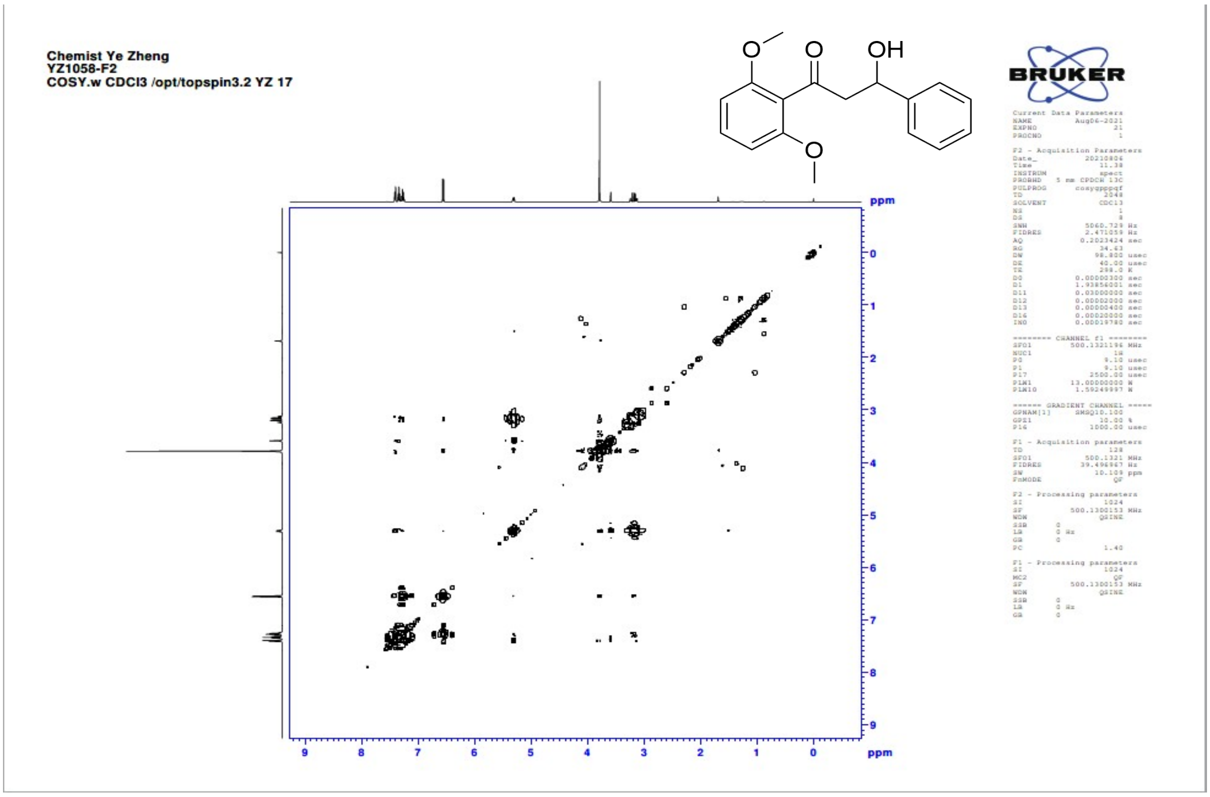

HSQC (500 MHz, $CDCl_3$) of 1-(2,6-dimethoxyphenyl)-3-hydroxy-3-phenylpropan-1-one **9b.**

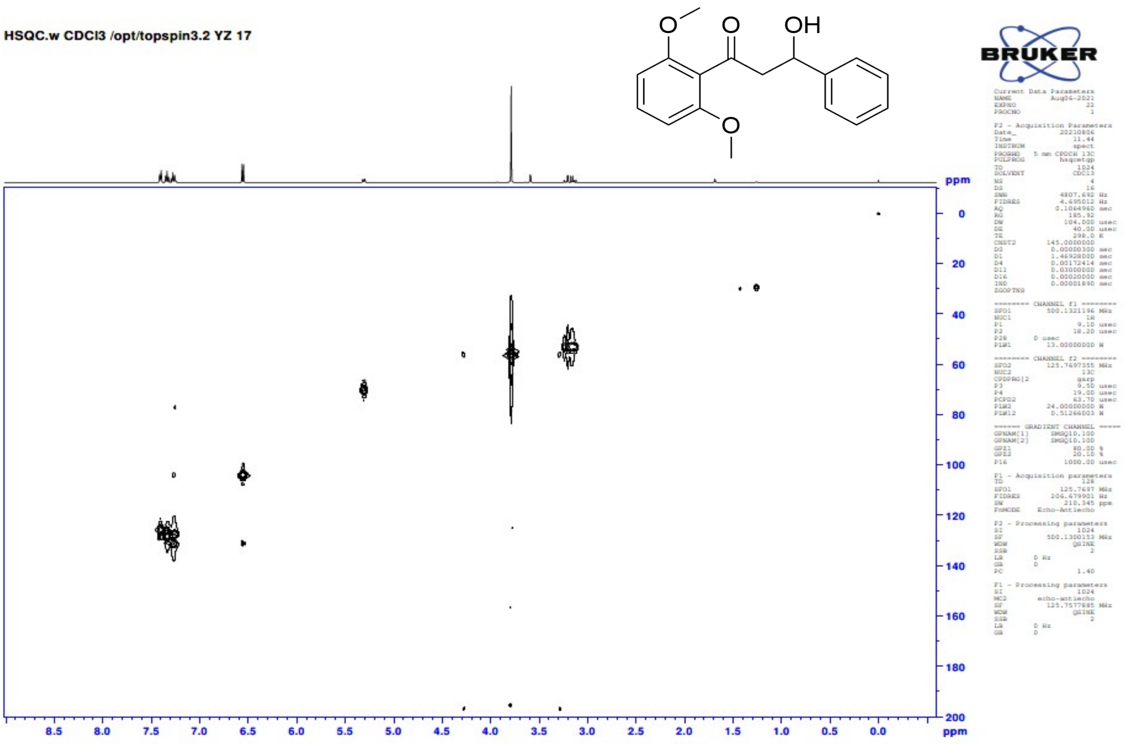

HMBC (500 MHz, $CDCl_3$) of 1-(2,6-dimethoxyphenyl)-3-hydroxy-3-phenylpropan-1-one **9b**.

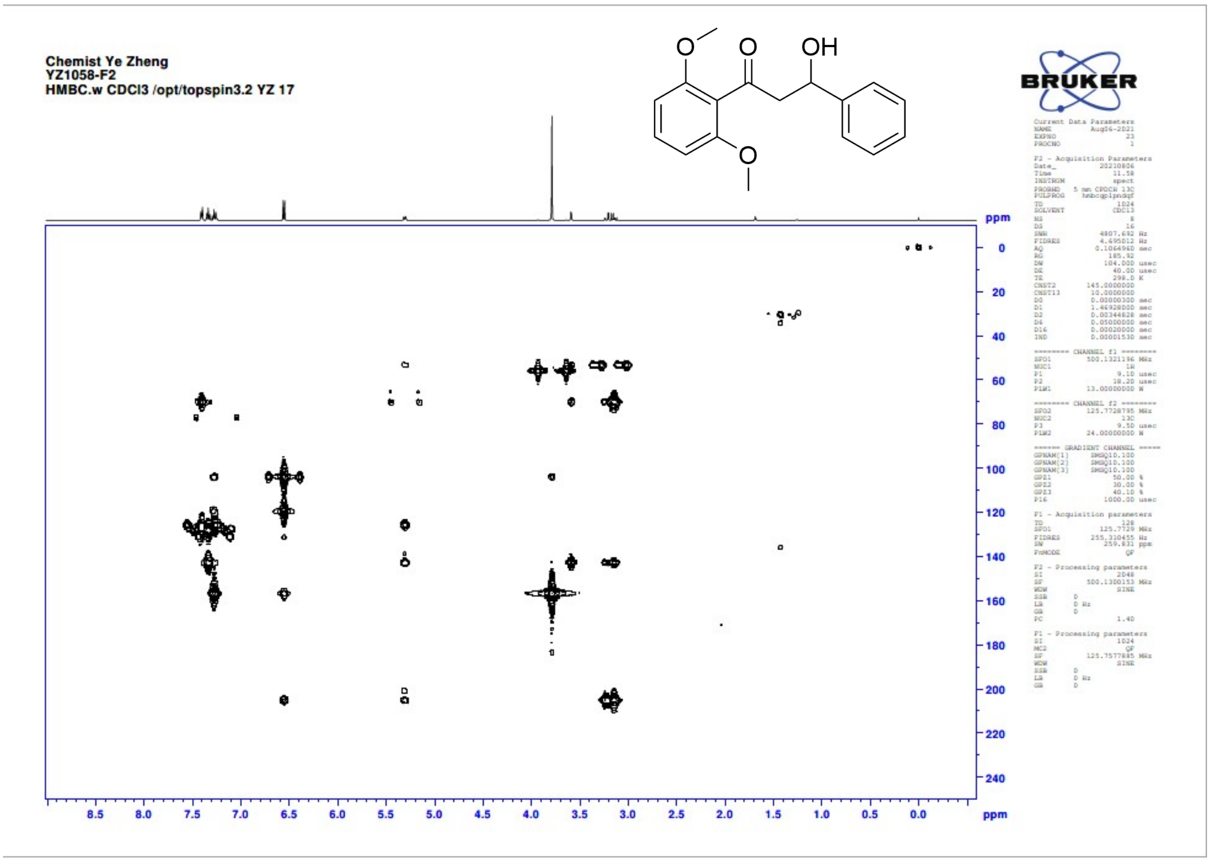

HPLC of racemic 1-(2,6-dimethoxyphenyl)-3-hydroxy-3-phenylpropan-1-one **9b.**

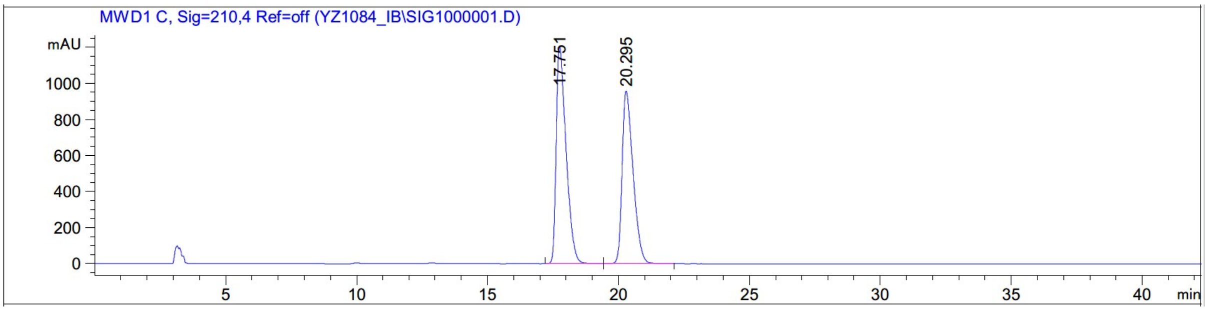

Signal 2: MWD1 C, Sig=210,4 Ref=off

Peak #	RetTime [min]	Type	Width [min]	Area [mAU*s]	Height [mAU]	Area %
1	17.751	BB	0.3974	3.15674e4	1205.85596	53.1998
2	20.295	BB	0.4424	2.77700e4	957.33966	46.8002
Totals :				5.93374e4	2163.19562	

HPLC of (*R*)- 1-(2,6-dimethoxyphenyl)-3-hydroxy-3-phenylpropan-1-one **9b.**

(*R,R*)-3C-Tethered Ru(II)-TsDPEN catalyst (after 24 h, 100% conversion, 98% ee (*R*)).

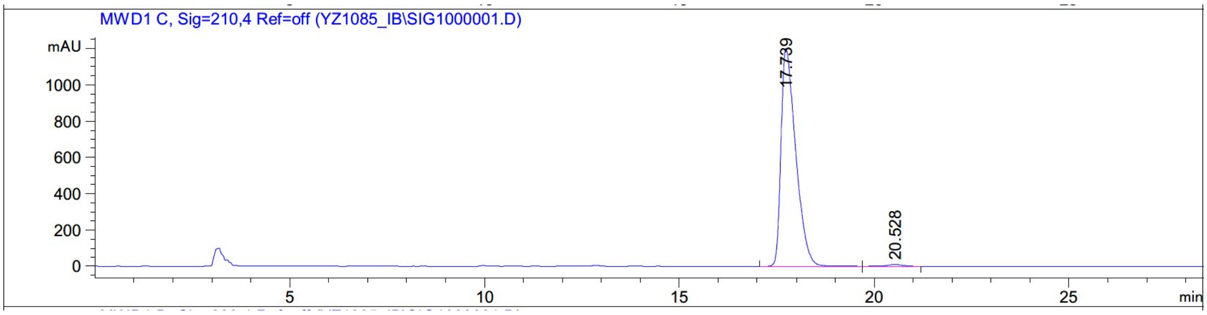

Signal 2: MWD1 C, Sig=210,4 Ref=off

Peak #	RetTime [min]	Type	Width [min]	Area [mAU*s]	Height [mAU]	Area %
1	17.739	BB	0.3940	3.15002e4	1200.90820	99.1412
2	20.528	BB	0.4034	272.85214	10.22391	0.8588

Totals : 3.17731e4 1211.13212

1-(2,6-Dimethoxyphenyl)-3-(4-methoxyphenyl)propane-1,3-dione 10a.

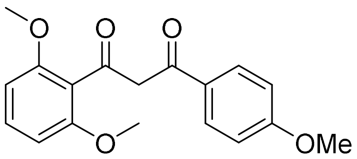

This compound is novel.

To a solution of sodium hydride (468 mg, 60% dispersion in mineral oil, 11.7 mmol) in THF (3 mL) at 0 °C was added dropwise a solution of 1-(2,6-dimethoxyphenyl)ethan-1-one **5** (421 mg, 2.34 mmol) in THF (3 mL). The reaction mixture was stirred under a nitrogen atmosphere at 0 °C for 30 min and then stirred under a nitrogen atmosphere at rt for 30 min, after which a solution of methyl 4-methoxybenzoate (1.95 g, 11.7 mmol) in THF (3 mL) was added dropwise. The reaction mixture was then heated to 65 °C and left stirring under the nitrogen atmosphere overnight. The reaction was followed by TLC (3:2 hexane: EtOAc). The mixture was quenched by 2M HCl solution (20 mL). EtOAc (20 mL) was added and the organic layer was separated. The aqueous layer was extracted with EtOAc (3 × 20 mL), and the combined organic layers were washed with saturated $NaHCO_3$ solution (2 × 20 mL) and brine (20 mL), dried ($MgSO_4$) and filtered. Solvent was removed to give the crude product. The product was isolated via flash chromatography on silica eluted with 25-100% EtOAc in hexane to give 1-(2,6-dimethoxyphenyl)-3-(4-methoxyphenyl)propane-1,3-dione **10a** as a yellow solid (377 mg, 1.20 mmol, 51%). TLC: Rf ca 0.40 (3:2 hexane: EtOAc), strong UV and $KMnO_4$; Mp: 108 °C; HRMS (ESI+) *m/z*: [M+H]+ Calcd for $C_{18}H_{18}NaO_8$ 337.1040; Found 337.1046; 1.9 ppm error; ν_{max} 3006, 2935, 2838, 1707, 1590, 1507, 1469, 1430, 1303, 1248, 1170, 1104, 1022, 987 cm^{-1}; enol: keto = 43:7; ^1H NMR (500 MHz, $CDCl_3$): δ 7.90 (2H, d, J = 8.9, ArH), 7.31 (1H, t, J = 8.4, ArH), 6.94 (2H, d, J = 8.9, ArH), 6.61 (2H, d, J = 8.4, ArH), 6.32 (0.84H, s, CH of enol form), 4.38 (0.28H, s, CH_2 of keto form), 3.86 (3H, s, CH_3), 3.82 (5.16H, s, OCH_3 of enol form), 3.74 (0.84H, s, OCH_3 of keto form); $^{13}C\{^1H\}$ NMR (125 MHz, $CDCl_3$): δ keto form: 197.9 (C), 192.1 (C), 163.6 (C), 156.9 (C), 131.4 (CH), 131.3 (CH), 130.1 (C), 119.1 (C), 113.7 (CH), 104.0 (CH), 55.9 (CH_2), 55.8 (CH_3), 55.5 (CH_3); enol form: 187.2 (C), 182.7 (C), 163.0 (C), 157.7 (C), 131.10 (CH), 129.2 (CH), 127.6 (C), 116.9 (C), 113.9 (CH), 104.1 (CH), 99.7 (CH), 56.1 (CH_3), 55.5 (CH_3); *m/z* (ES-API+) 337.1 (M^+ + 23, 100%).

Enantiomeric excess and conversion determined by HPLC analysis (Chiralpak IB, 30 cm x 6 mm column, hexane:iPrOH 90:10, 1.0 mL/min, T = 25°C) ketone 20.7 min, *R* and *S* isomers 28.5 min and 30.7 min, configuration assigned by analogy.

^{1}H NMR (500 MHz, $CDCl_3$) of 1-(2,6-dimethoxyphenyl)-3-(4-methoxyphenyl)propane-1,3-dione **10a.**

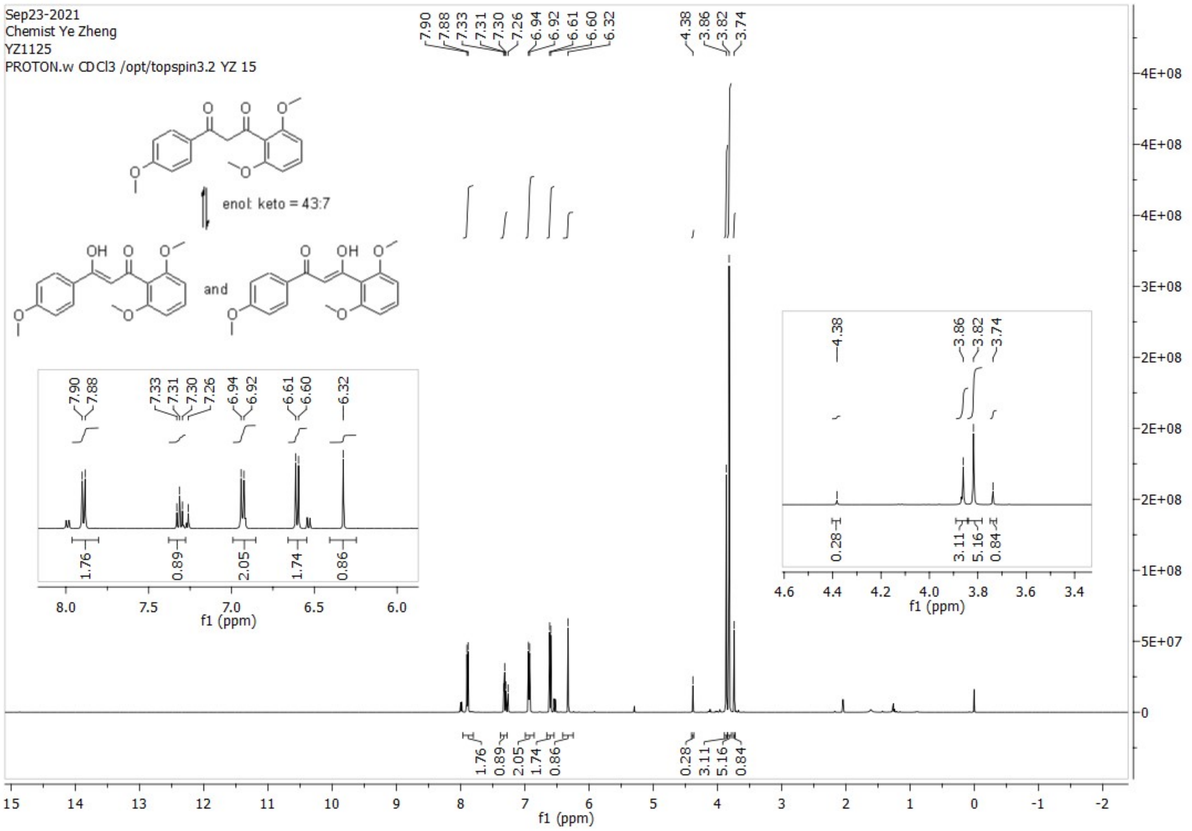

$^{13}C\{^{1}H\}$ NMR (125 MHz, $CDCl_3$) of 1-(2,6-dimethoxyphenyl)-3-(4-methoxyphenyl)propane-1,3-dione **10a.**

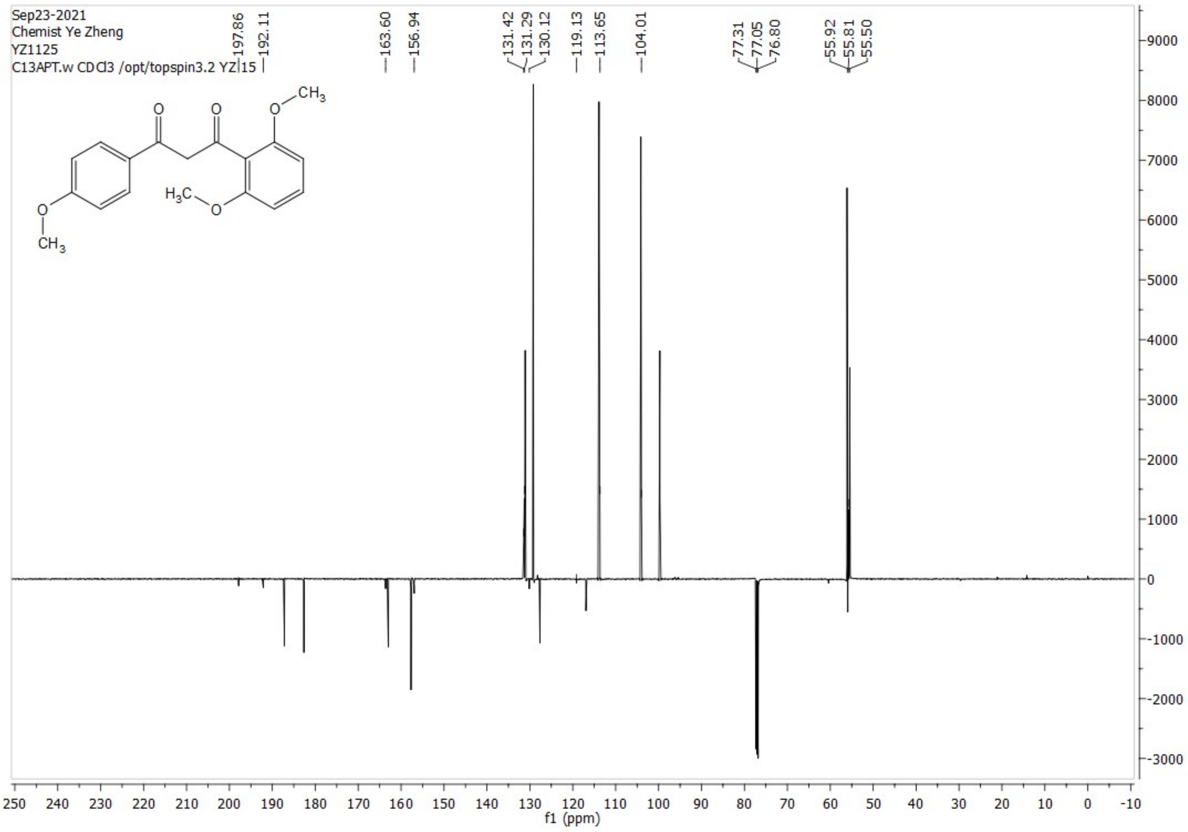

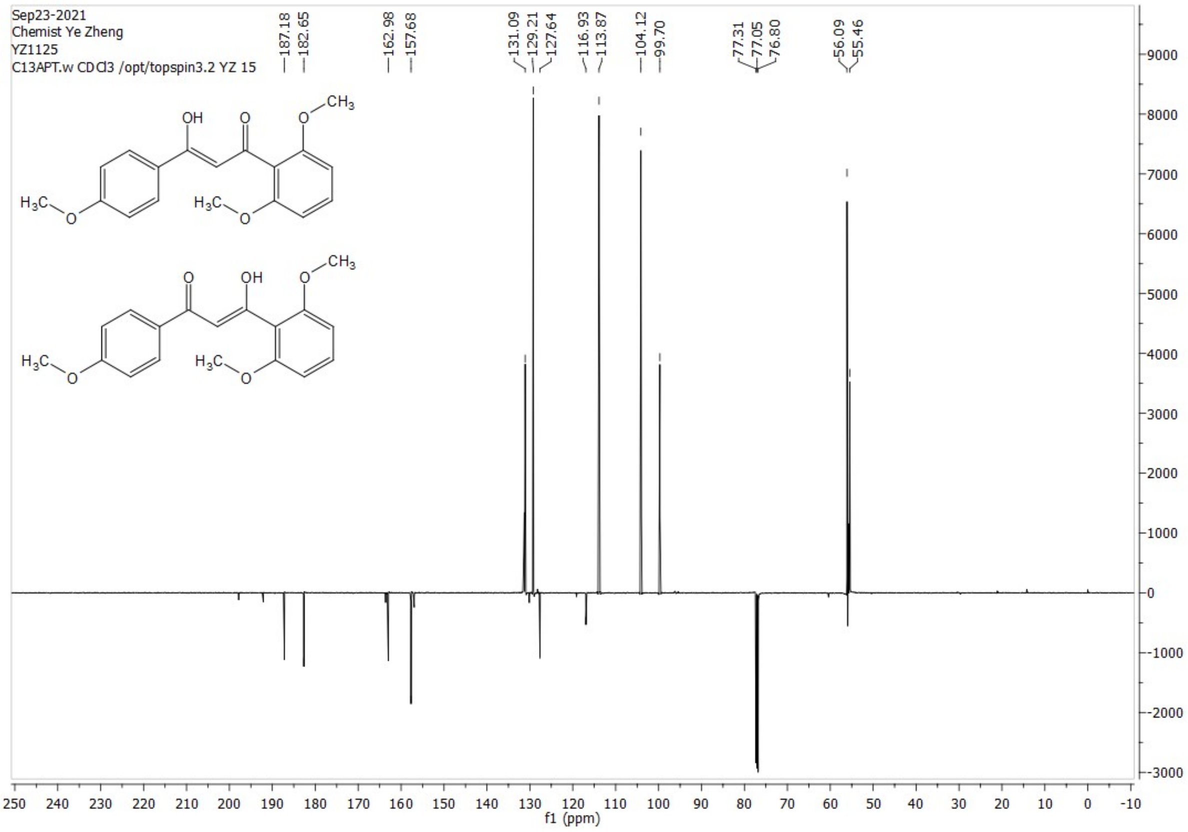

COSY (500 MHz, $CDCl_3$) of 1-(2,6-dimethoxyphenyl)-3-(4-methoxyphenyl)propane-1,3-dione **10a**

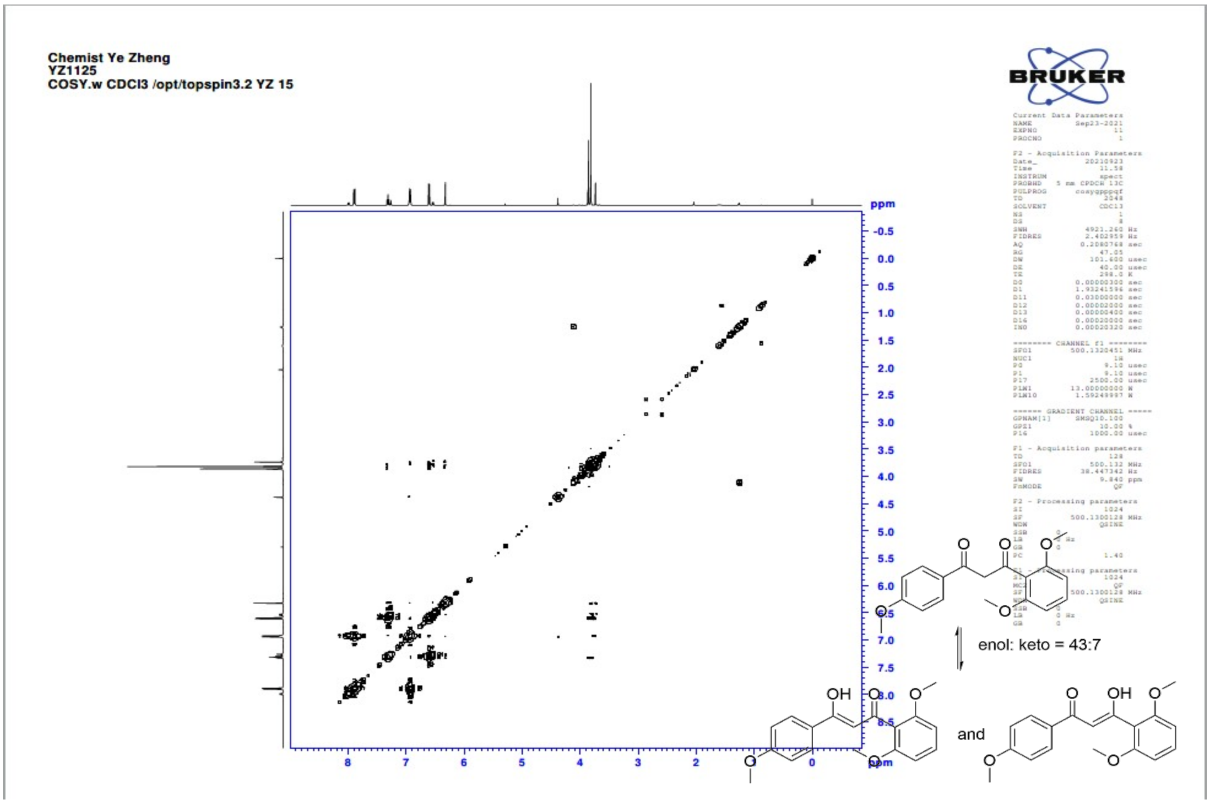

HSQC (500 MHz, $CDCl_3$) of 1-(2,6-dimethoxyphenyl)-3-(4-methoxyphenyl)propane-1,3-dione **10a.**

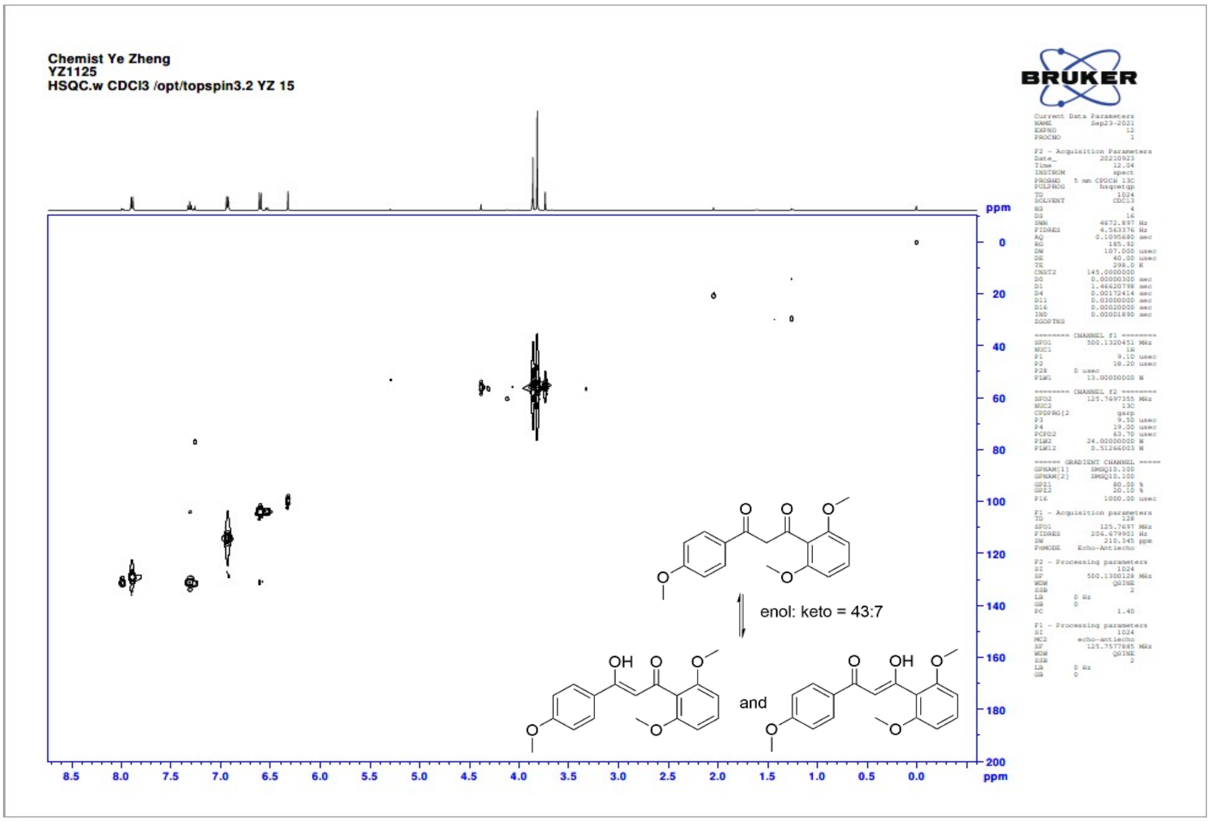

HMBC (500 MHz, $CDCl_3$) of 1-(2,6-dimethoxyphenyl)-3-(4-methoxyphenyl)propane-1,3-dione **10a.**

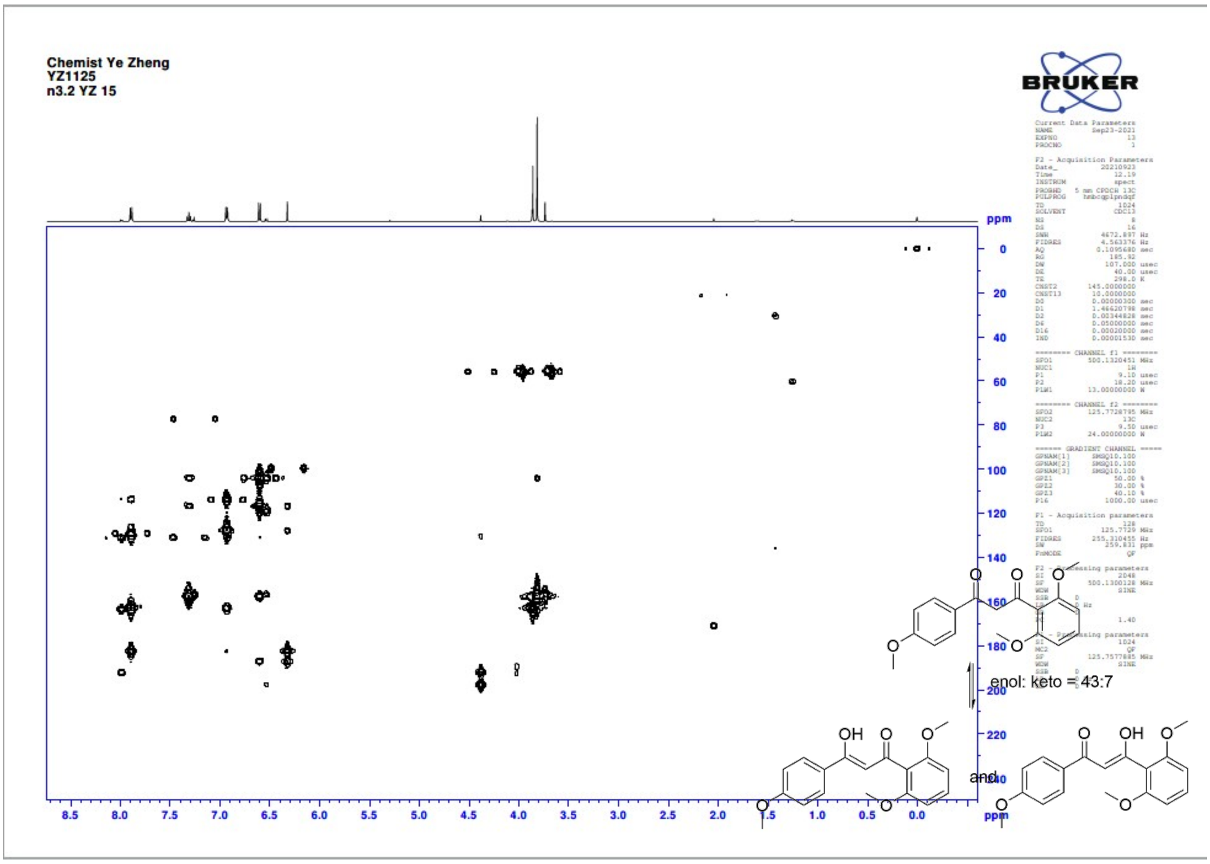

HPLC of 1-(2,6-dimethoxyphenyl)-3-(4-methoxyphenyl)propane-1,3-dione **10a.**

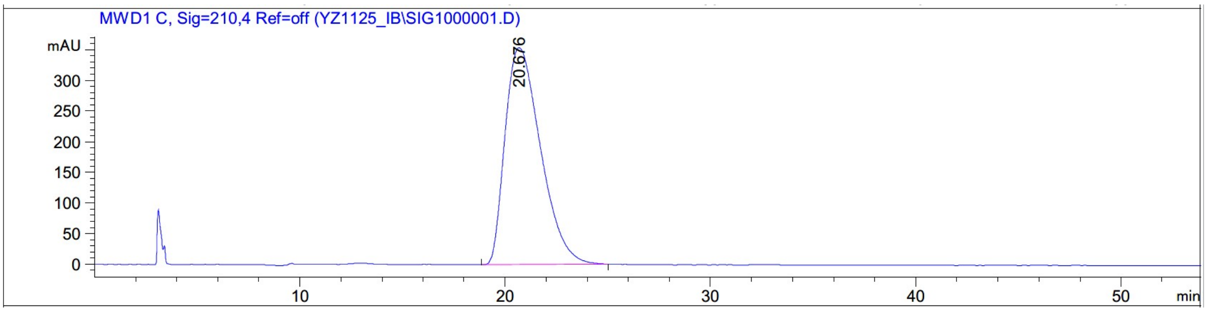

Signal 2: MWD1 C, Sig=210,4 Ref=off

Peak #	RetTime [min]	Type	Width [min]	Area [mAU*s]	Height [mAU]	Area %
1	20.676	BB	1.7902	4.25212e4	353.78183	100.0000

Totals : 4.25212e4 353.78183

1-(2,6-Dimethoxyphenyl)-3-hydroxy-3-(4-methoxyphenyl)propan-1-one 10b.

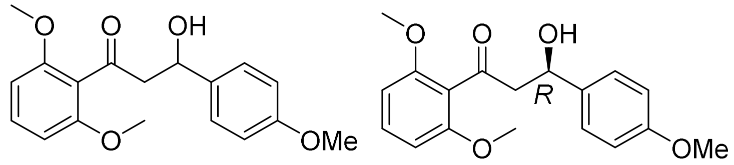

This compound is novel.

Synthesis of a racemic standard: (*R,R*)-3C-Tethered Ru(II)-TsDPEN catalyst (1.0 mg, 1.6 mmol, 0.5 mol%) and (*S,S*)-3C-tethered Ru(II)-TsDPEN catalyst (1.0 mg, 1.6 mmol, 0.5 mol%) were added to FA: TEA (5:2 azeotropic mixture, 0.45 mL) at rt and the mixture was stirred under a nitrogen atmosphere for 15 minutes; after which a solution of 1-(2,6-dimethoxyphenyl)-3-(4-methoxyphenyl)propane-1,3-dione **10a** (100 mg, 0.318 mmol) in DCM (0.63 mL) was added. The reaction mixture was stirred under a nitrogen atmosphere and followed by TLC (3:2 hexane: EtOAc). After 24 h, the reaction was quenched using saturated $NaHCO_3$ solution (20 mL). EtOAc (20 mL) was added and the organic layer was separated. The aqueous layer was extracted with EtOAc (3 x 20 mL) and the combined organic layers were dried ($MgSO_4$) and filtered. The solvent was removed to give the crude product. The product was isolated via flash chromatography on silica eluted with 0-50% EtOAc in hexane to 1-(2,6-dimethoxyphenyl)-3-hydroxy-3-(4-methoxyphenyl)propan-1-one **10b** as a yellow oil (59.0 mg, 0.187 mmol, 59%). TLC: Rf ca 0.30 (3:2 hexane: EtOAc), strong UV and $KMnO_4$; HRMS (ESI+) *m/z*: [M+H]+, Calcd for $C_{18}H_{20}NaO_5$ 339.1199; Found 339.1203; 1.1 ppm error; ν_{max} 3489 (br), 2935, 2837, 1693, 1591, 1511, 1470, 1432, 1302, 1287, 1245, 1172, 1105, 1027, 830, 733 cm^{-1}; ^1H NMR (400 MHz, $CDCl_3$): δ 7.32-7.24 (3H, m, ArH), 6.87 (2H, d, *J* = 8.5, ArH), 6.55 (2H, d, *J* = 8.5, ArH), 5.25 (1H, d, *J* = 8.1, ArCH), 3.77 (9H, s, CH_3), 3.51 (1H, d, *J* = 1.4, OH), 3.21-3.19 (2H, m, CH_2); $^{13}C\{^1H\}$ NMR (100 MHz, $CDCl_3$): δ 205.4 (C), 159.0 (C), 156.8 (C), 135.2 (C), 131.2 (CH), 127.1 (CH), 119.7 (C), 113.8 (CH), 104.1 (CH), 69.7 (CH), 56.0 (CH_3), 55.3 (CH_3), 53.4 (CH_2); *m/z* (ES-API+) 339.1 (M^+ + 23, 100%).

Enantiomeric excess and conversion determined by HPLC analysis (Chiralpak IB, 30 cm x 6 mm column, hexane:iPrOH 90:10, 1.0 mL/min, T = 25°C) ketone 20.7 min, *R* and *S* isomers 28.5 min and 30.7 min, configuration assigned by analogy.

(*R*)-1-(2,6-Dimethoxyphenyl)-3-hydroxy-3-(4-methoxyphenyl)propan-1-one **10b.** (*R,R*)-3C-tethered Ru(II)-TsDPEN catalyst (2.0 mg, 0.32 mmol, 1 mol%) was added to FA: TEA (5:2 azeotropic mixture, 0.45 mL) at rt and the mixture was stirred under a nitrogen atmosphere for 15 minutes, after which a solution of 1-(2,6-dimethoxyphenyl)-3-(4-methoxyphenyl)propane-1,3-dione **10a** (100 mg, 0.318 mmol) in DCM (0.63 mL) was added. The reaction mixture was stirred under a nitrogen atmosphere and followed by TLC (3:2 hexane: EtOAc). After 24 h, the reaction was quenched using saturated $NaHCO_3$ solution (20 mL). EtOAc (20 mL) was added and the organic layer was separated. The aqueous layer was extracted with EtOAc (3 x 20 mL) and the combined organic layers were dried ($MgSO_4$) and filtered. The solvent was removed to give the crude product. The product was isolated via flash chromatography on silica eluted with 0-50% EtOAc in hexane to (*R*)-1-(2,6-dimethoxyphenyl)-3-hydroxy-3-(4-methoxyphenyl)propan-1-one **10b** as a yellow oil (53.0 mg, 0.168 mmol, 53%). The reaction was also followed by HPLC (Chiralpak IB, 30 cm x 6 mm column, hexane:iPrOH 90:10, 1.0 mL/min, T = 25°C): 100% conversion: $[\alpha]_D^{24}$ +25.9 (c 0.212 in $CHCl_3$) 97% ee (*R*).

^{1}H NMR (400 MHz, $CDCl_3$) of (*R*)-1-(2,6-dimethoxyphenyl)-3-hydroxy-3-(4-methoxyphenyl)propan-1-one **10b.**

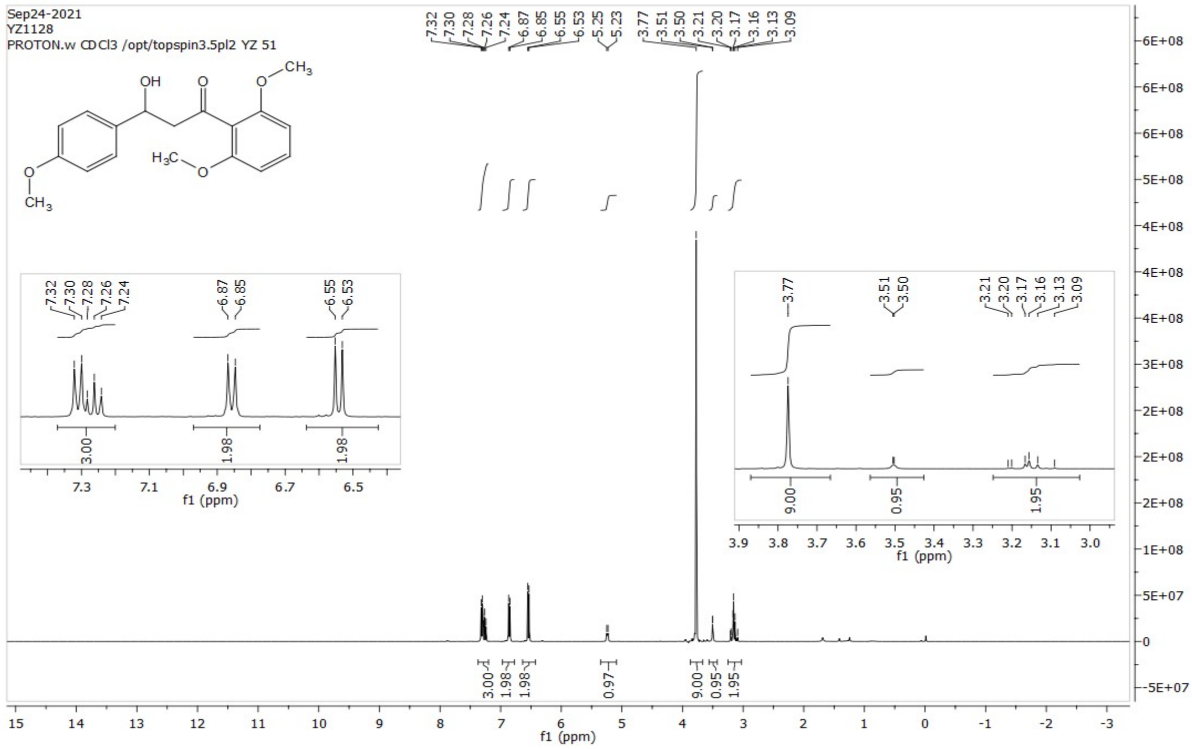

^{13}C{^{1}H} NMR (100 MHz, $CDCl_3$) of (*R*)-1-(2,6-dimethoxyphenyl)-3-hydroxy-3-(4-methoxyphenyl)propan-1-one **10b.**

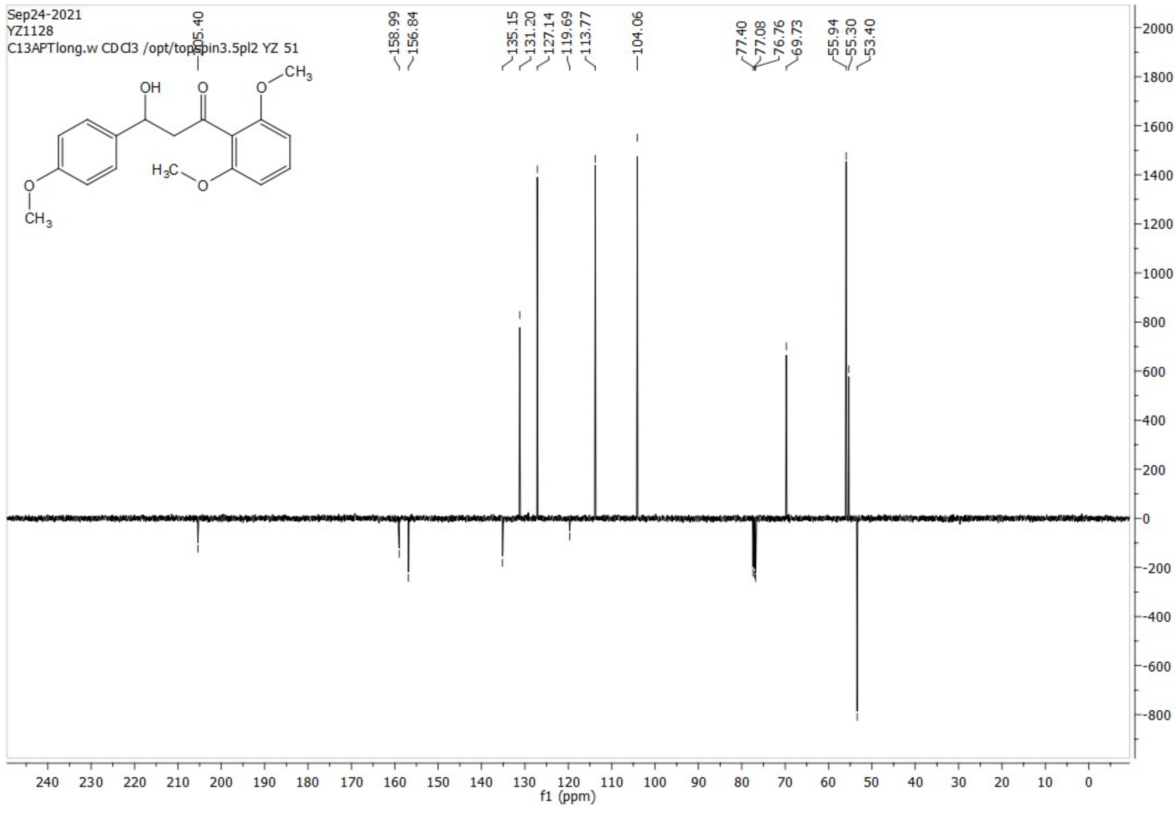

COSY (400 MHz, $CDCl_3$) of (*R*)-1-(2,6-dimethoxyphenyl)-3-hydroxy-3-(4-methoxyphenyl)propan-1-one **10b.**

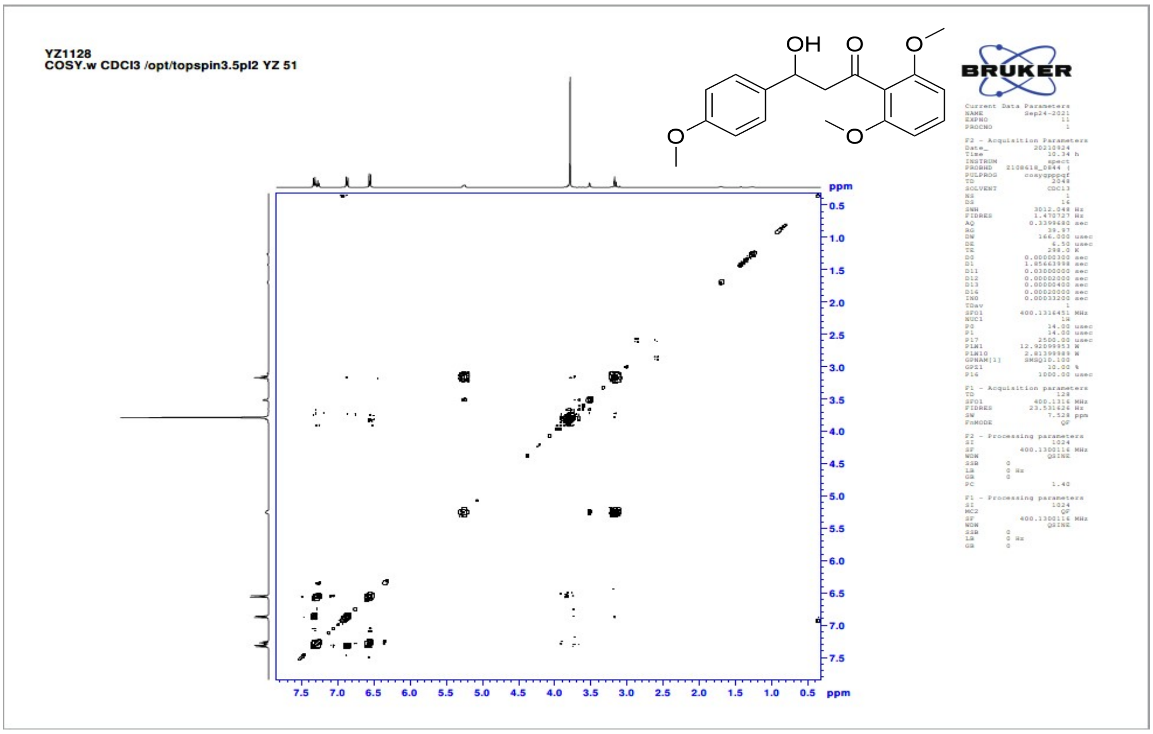

HSQC (400 MHz, $CDCl_3$) of (*R*)-1-(2,6-dimethoxyphenyl)-3-hydroxy-3-(4-methoxyphenyl)propan-1-one **10b.**

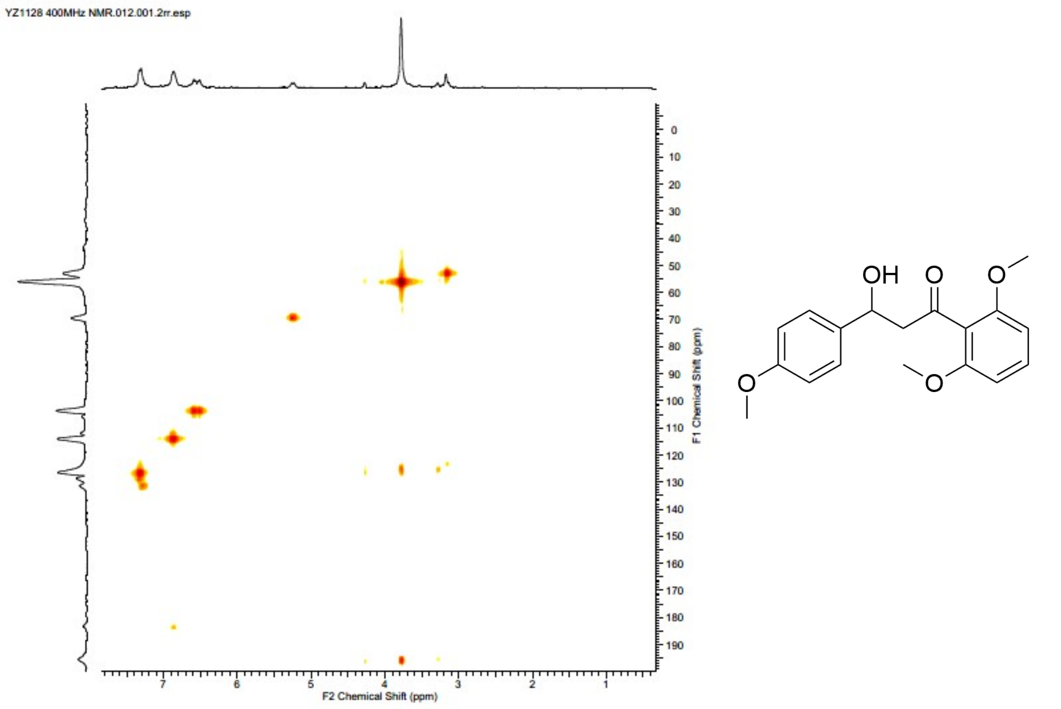

HMBC (400 MHz, $CDCl_3$) of (*R*)-1-(2,6-dimethoxyphenyl)-3-hydroxy-3-(4-methoxyphenyl)propan-1-one **10b**

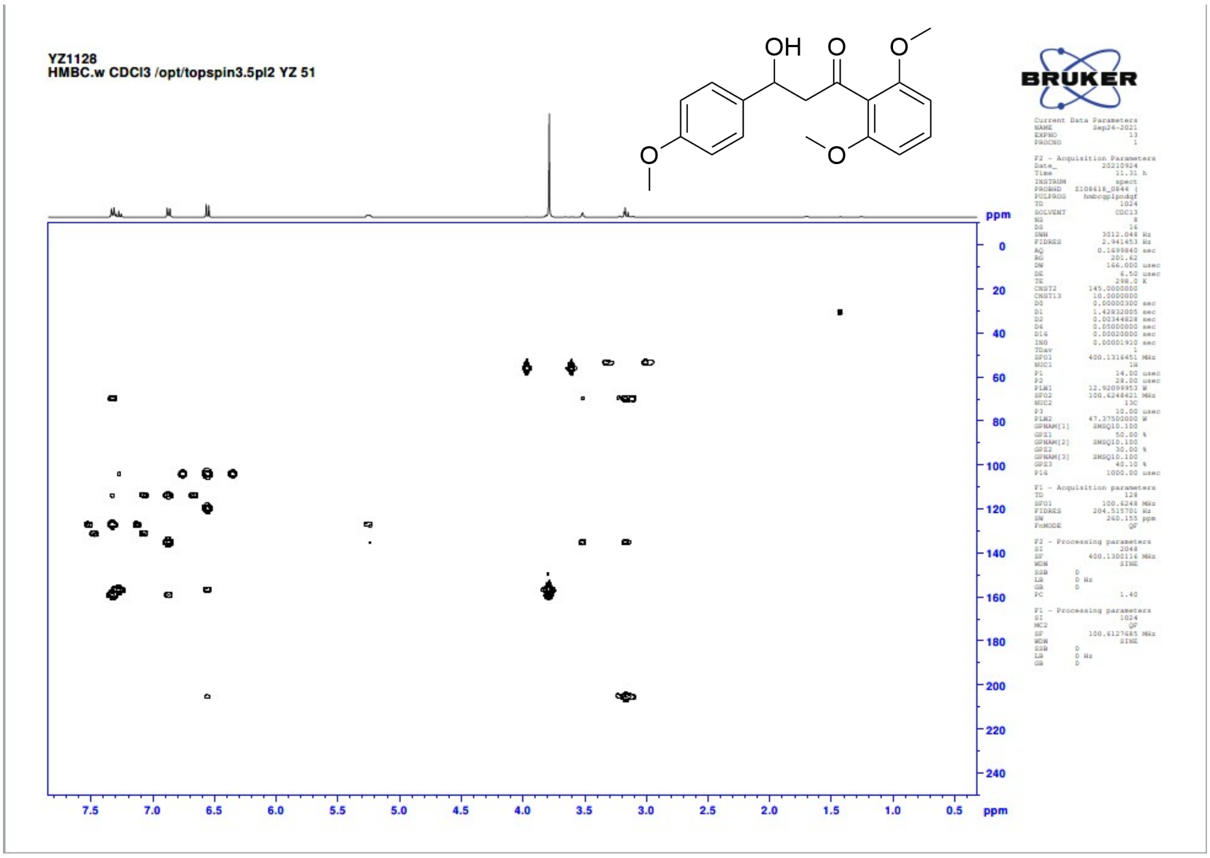

HPLC of racemic 1-(2,6-dimethoxyphenyl)-3-hydroxy-3-(4-methoxyphenyl)propan-1-one **10b.**

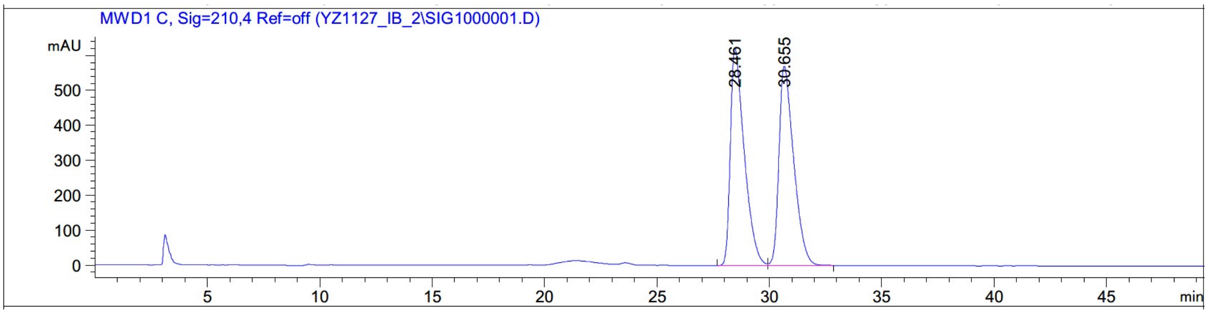

Signal 2: MWD1 C, Sig=210,4 Ref=off

Peak #	RetTime [min]	Type	Width [min]	Area [mAU*s]	Height [mAU]	Area %
1	28.461	BV	0.6298	2.66824e4	622.83954	50.6410
2	30.655	VB	0.6818	2.60070e4	570.59058	49.3590

Totals : 5.26894e4 1193.43011

HPLC of (*R*)-1-(2,6-dimethoxyphenyl)-3-hydroxy-3-(4-methoxyphenyl)propan-1-one **10b.** (*R,R*)-3C-Tethered Ru(II)-TsDPEN catalyst (after 24 h, 100% conversion, 97% ee (*R*))

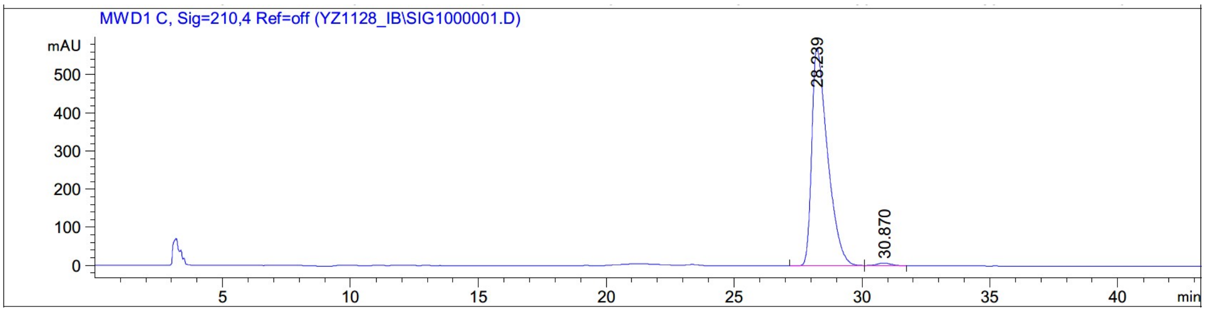

Signal 2: MWD1 C, Sig=210,4 Ref=off

Peak #	RetTime [min]	Type	Width [min]	Area [mAU*s]	Height [mAU]	Area %
1	28.239	BV	0.6344	2.46685e4	572.87982	98.6684
2	30.870	VB	0.5896	332.91327	7.71249	1.3316

Totals : 2.50014e4 580.59231

1-(2-Chlorophenyl)-3-(2,6-dimethoxyphenyl)propane-1,3-dione 11a.

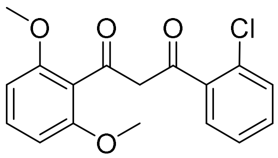

This compound is novel.

To a solution of sodium hydride (583 mg, 60% dispersion in mineral oil, 14.6 mmol) in THF (4 mL) at 0 °C was added dropwise a solution of 1-(2,6-dimethoxyphenyl)ethan-1-one **5** (526 mg, 2.92 mmol) in THF (4 mL). The reaction mixture was stirred under a nitrogen atmosphere at 0 °C for 30 min and then stirred under a nitrogen atmosphere at rt for 30 min, after which a solution of ethyl 2-chlorobenzoate (2.70 g, 14.6 mmol) in THF (4 mL) was added dropwise. The reaction mixture was then heated to 65 °C and left stirring under the nitrogen atmosphere overnight. The reaction was followed by TLC (4:1 hexane: EtOAc). The mixture was quenched by 2M HCl solution (20 mL). EtOAc (20 mL) was added and the organic layer was separated. The aqueous layer was extracted with EtOAc (3 × 20 mL), and the combined organic layers were washed with saturated $NaHCO_3$ solution (2 × 20 mL) and brine (20 mL), dried ($MgSO_4$) and filtered. Solvent was removed to give the crude product. The product was isolated via flash chromatography on silica eluted with 0-50% EtOAc in hexane to give 1-(2-chlorophenyl)-3-(2,6-dimethoxyphenyl)propane-1,3-dione **11a** as a yellow solid (583 mg, 1.83 mmol, 63%). TLC: Rf ca 0.40 (4:1 hexane: EtOAc), strong UV and $KMnO_4$; Mp: 106 °C; HRMS (ESI+) *m/z*: [M+H]+ Calcd for $C_{17}H_{15}{}^{35}ClNaO_4$ 341.0544; Found 341.0551; 2.0 ppm error; ν_{max} 3012, 2962, 2838, 1587, 1470, 1432, 1285, 1249, 1211, 1104, 1058, 1025, 757, 740, 728 cm^{-1}; enol: keto = 49:1; ^1H NMR (400 MHz, $CDCl_3$): δ 15.78 (1H, s, OH of enol form), 7.70 (1H, dd, *J* = 7.2, 2.2, ArH), 7.45-7.30 (4H, m, ArH), 6.61 (2H, d, *J* = 8.4, ArH), 6.32 (0.98H, s, CH of enol form), 4.50 (0.04H, s, CH_2 of keto form), 3.83 (5.88H, s, OCH_3 of enol form), 3.82 (0.12H, s, OCH_3 of keto form); $^{13}C\{^1H\}$ NMR (100 MHz, $CDCl_3$) δ keto form: 188.5 (C), 181.8 (C), 158.0 (C), 135.4 (C), 132.7 (CH), 132.1 (C), 131.8 (CH), 130.9 (CH), 130.3 (CH), 126.8 (CH), 116.5 (C), 105.7 (CH), 64.4 (CH_2), 55.8 (CH_3); enol form: 188.5 (C), 181.8 (C), 158.0 (C), 135.4 (C), 132.1 (C), 131.6 (CH), 131.5 (CH), 130.7 (CH), 130.4 (CH), 126.9 (CH), 116.5 (C), 105.9 (CH), 104.2 (CH), 56.1 (CH_3); *m/z* (ES-API+) 341.1 (M^+ + 23, 100%).

Enantiomeric excess and conversion determined by HPLC analysis (Chiralpak IB, 30 cm x 6 mm column, hexane:iPrOH 95:5, 1.0 mL/min, T = 25°C) ketone 12.1 min, *R* and *S* isomers 20.9 min and 22.3 min, configuration assigned by analogy.

^{1}H NMR (400 MHz, $CDCl_3$) of 1-(2-chlorophenyl)-3-(2,6-dimethoxyphenyl)propane-1,3-dione **11a.**

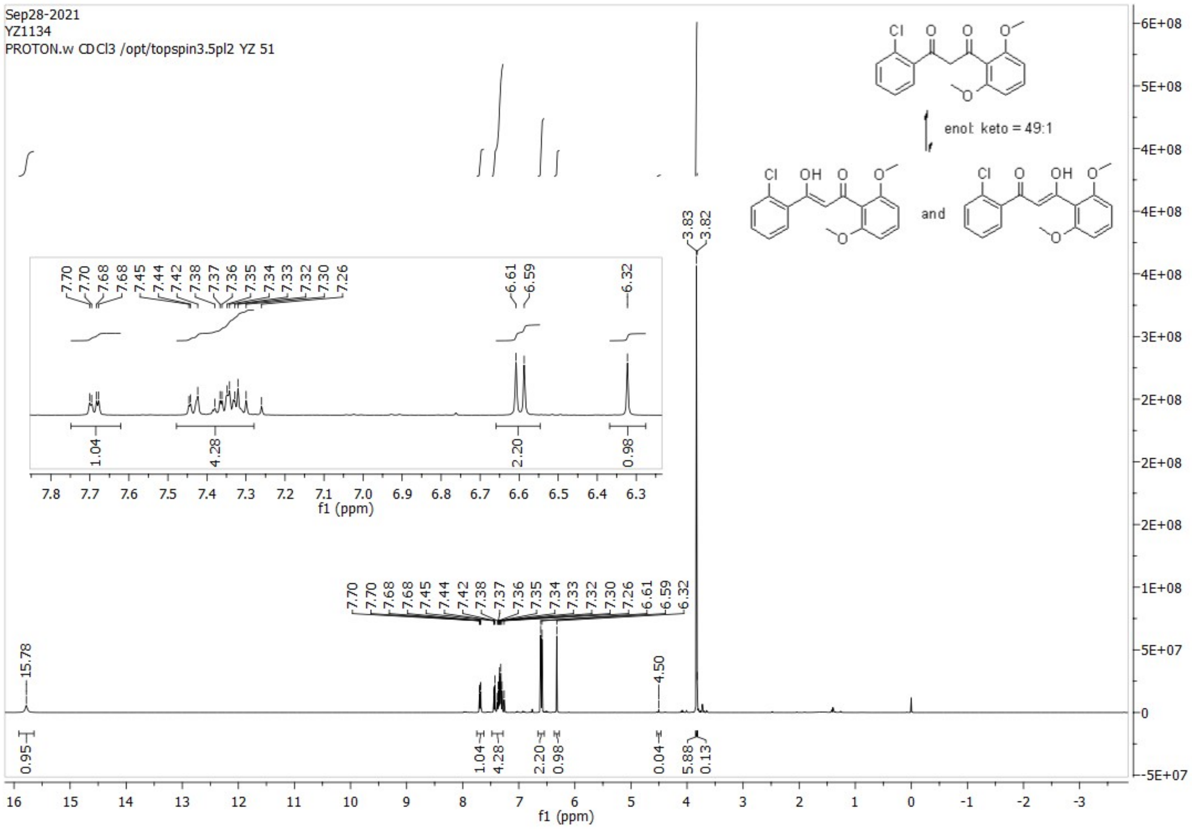

$^{13}C\{^1H\}$ NMR (100 MHz, $CDCl_3$) of 1-(2-chlorophenyl)-3-(2,6-dimethoxyphenyl)propane-1,3-dione **11a.**

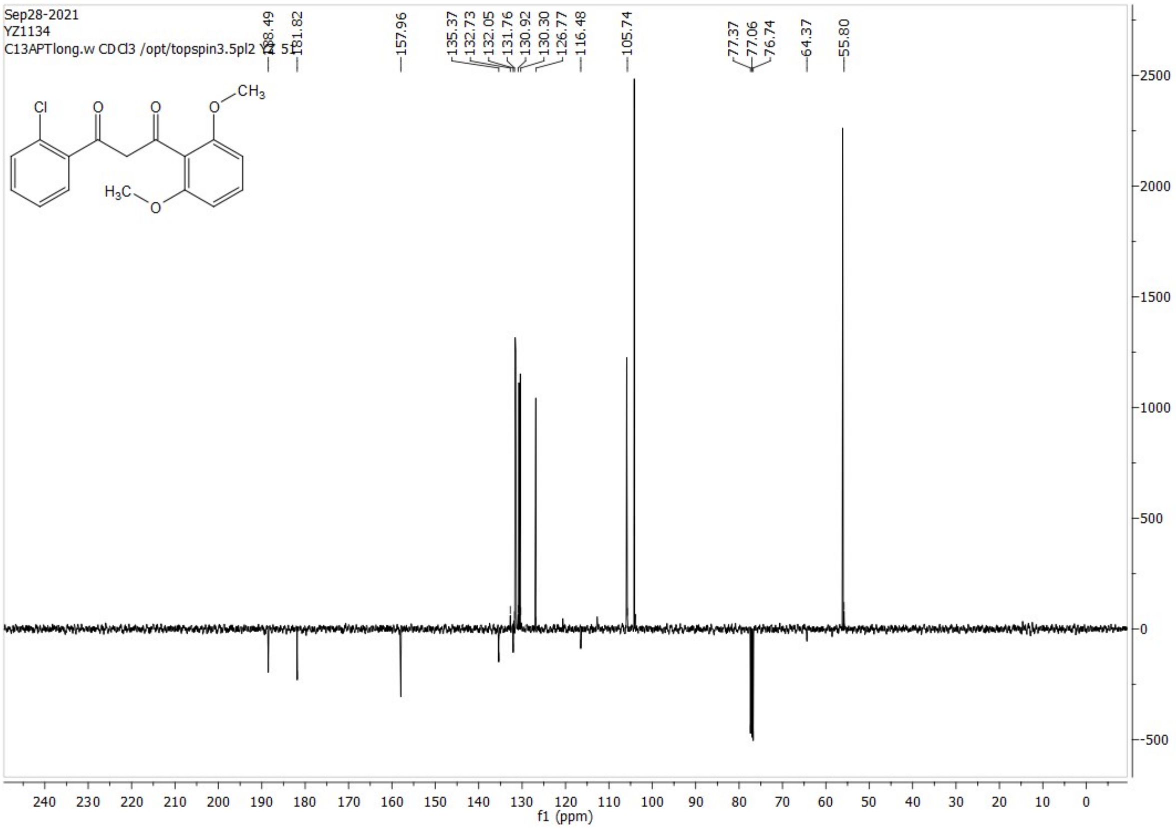

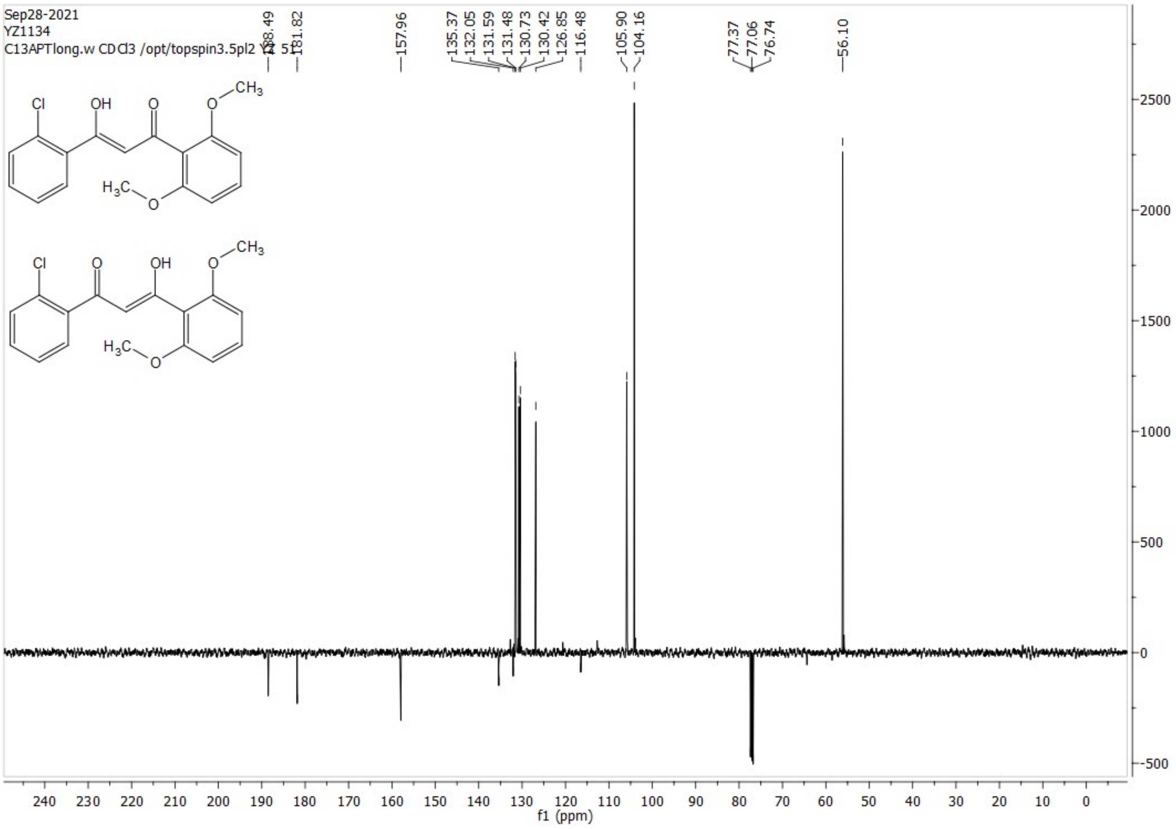

COSY (400 MHz, $CDCl_3$) of 1-(2-chlorophenyl)-3-(2,6-dimethoxyphenyl)propane-1,3-dione **11a.**

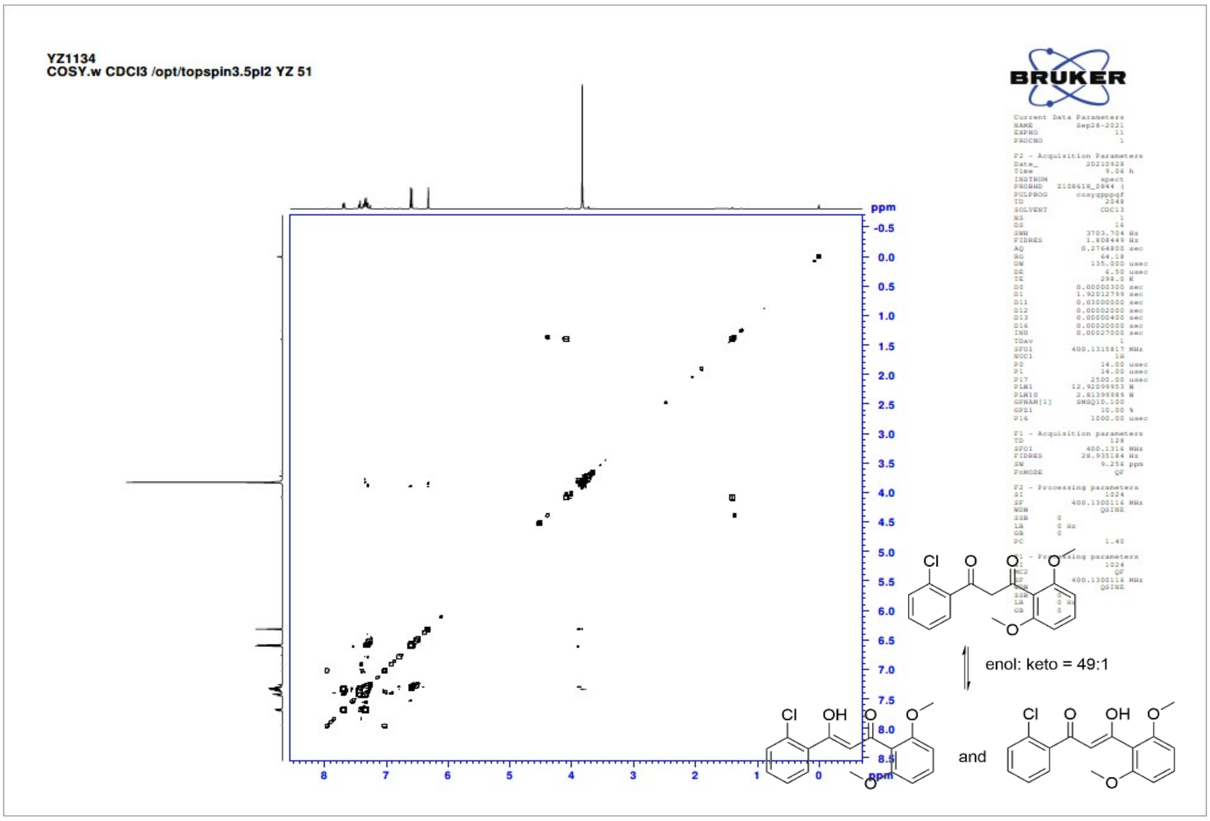

HSQC (400 MHz, $CDCl_3$) of 1-(2-chlorophenyl)-3-(2,6-dimethoxyphenyl)propane-1,3-dione **11a.**

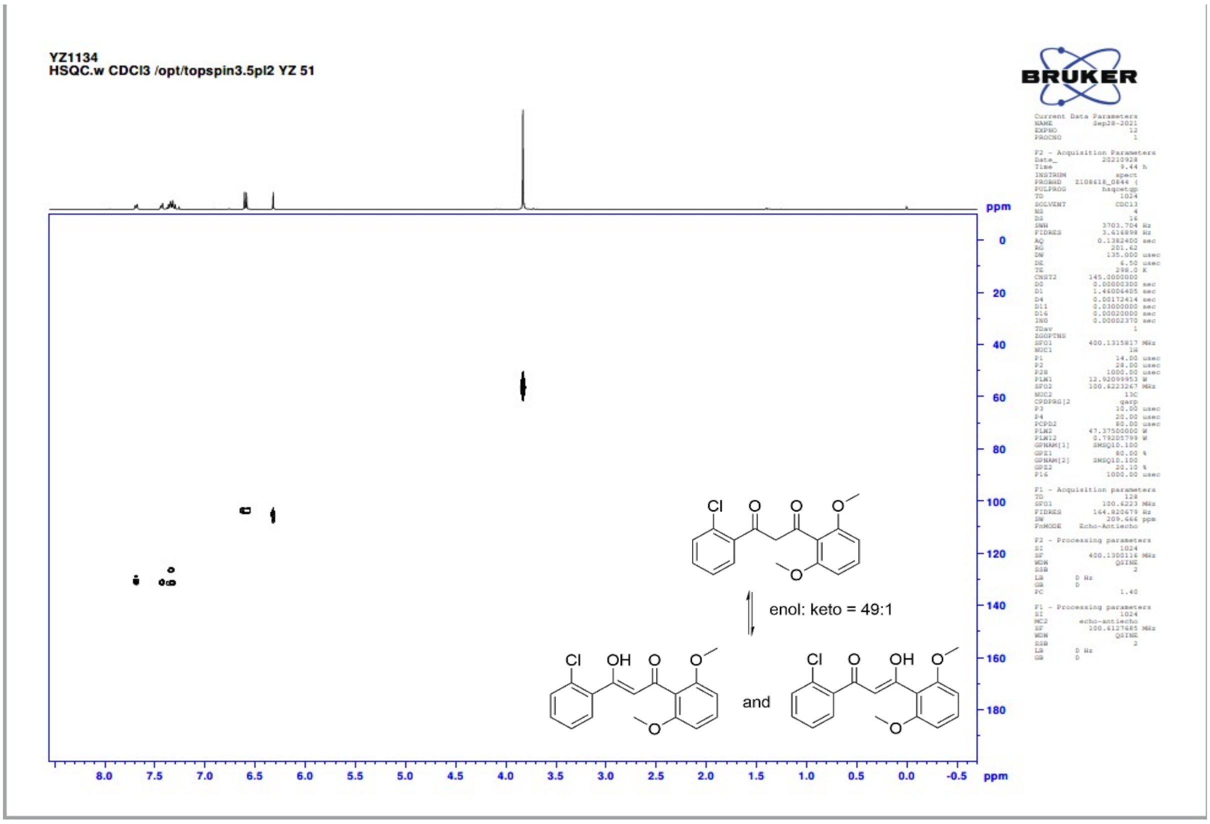

HMBC (400 MHz, $CDCl_3$) of 1-(2-chlorophenyl)-3-(2,6-dimethoxyphenyl)propane-1,3-dione **11a.**

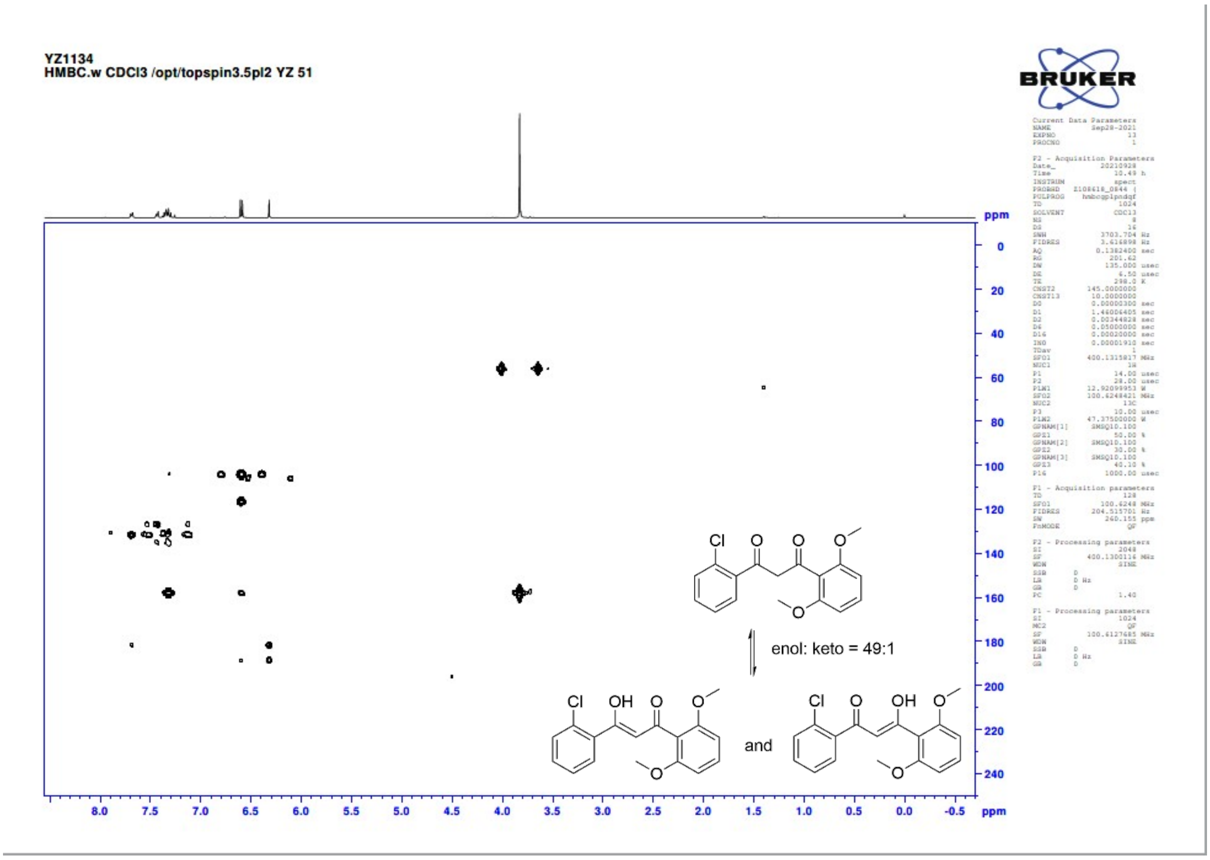

HPLC of 1-(2-chlorophenyl)-3-(2,6-dimethoxyphenyl)propane-1,3-dione **11a.**

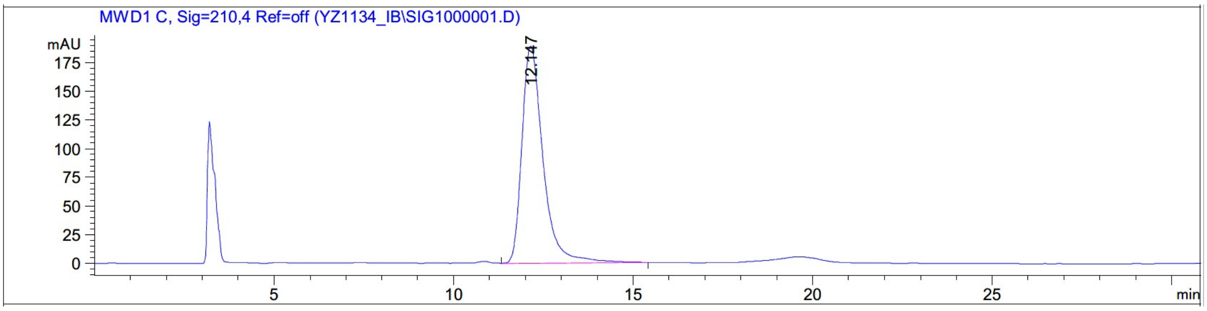

Signal 2: MWD1 C, Sig=210,4 Ref=off

Peak #	RetTime [min]	Type	Width [min]	Area [mAU*s]	Height [mAU]	Area %
1	12.147	BB	0.6239	7742.90967	189.01253	100.0000

Totals : 7742.90967 189.01253

3-(2-Chlorophenyl)-1-(2,6-dimethoxyphenyl)-3-hydroxypropan-1-one 11b.

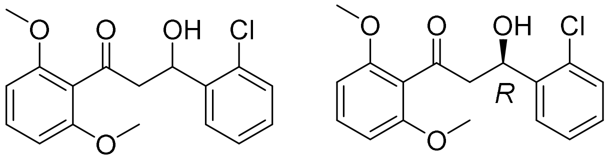

This compound is novel.

Synthesis of a racemic standard: (*R,R*)-3C-Tethered Ru(II)-TsDPEN catalyst (0.78 mg, 1.3 mmol, 0.5 mol%) and (*S,S*)-3C-tethered Ru(II)-TsDPEN catalyst (0.78 mg, 1.3 mmol, 0.5 mol%) were added to FA: TEA (5:2 azeotropic mixture, 0.36 mL) at rt and the mixture was stirred under a nitrogen atmosphere for 15 minutes; after which a solution of 1-(2-chlorophenyl)-3-(2,6-dimethoxyphenyl)propane-1,3-dione **11a** (80.0 mg, 0.252 mmol) in DCM (0.50 mL) was added. The reaction mixture was stirred under a nitrogen atmosphere and followed by TLC (4:1 hexane: EtOAc). After 24 h, the reaction was quenched using saturated $NaHCO_3$ solution (20 mL). EtOAc (20 mL) was added and the organic layer was separated. The aqueous layer was extracted with EtOAc (3 x 20 mL) and the combined organic layers were dried ($MgSO_4$) and filtered. The solvent was removed to give the crude product. The product was isolated via flash chromatography on silica eluted with 0-50% EtOAc in hexane to give 3-(2-chlorophenyl)-1-(2,6-dimethoxyphenyl)-3-hydroxypropan-1-one **11b** as a colorless oil (69.5 mg, 0.217 mmol, 86%). TLC: Rf ca 0.20 (4:1 hexane: EtOAc), strong UV and $KMnO_4$; HRMS (ESI+) *m/z*: [M+H]+ Calcd for $C_{17}H_{17}{}^{35}ClNaO_4$ 343.0701; Found 343.0708; 1.9 ppm error; ν_{max} 3476 (br), 2938, 2838, 1692, 1591, 1470, 1432, 1251, 1106, 1070, 1074, 1017, 986, 780, 755, 734, 721 cm^{-1}; ^1H NMR (500 MHz, $CDCl_3$): δ 7.69 (1H, dt, *J* = 7.7, 1.2, ArH), 7.32-7.28 (3H, m, ArH), 7.19 (1H, td, *J* = 7.7, 1.6, ArH), 6.56 (2H, d, *J* = 8.4, ArH), 5.65 (1H, d, *J* = 9.6, ArCH), 3.88 (1H, d, *J* = 2.8, OH), 3.79 (6H, s, OCH_3), 3.43 (1H, dd, *J* = 17.6, 2.1, CH_2), 2.92 (1H, dd, *J* = 17.6, 9.6, CH_2); $^{13}C\{^1H\}$ NMR (125 MHz, $CDCl_3$): δ 205.6 (C), 156.9 (C), 140.4 (C), 131.3 (CH), 131.3 (C), 129.2 (CH), 128.4 (CH), 127.3 (CH), 127.1 (CH), 119.4 (C), 104.0 (CH), 67.1 (CH), 55.9 (CH_3), 51.4 (CH_2); *m/z* (ES-API+) 343.1 (M^+ + 23, 100%).

Enantiomeric excess and conversion determined by HPLC analysis (Chiralpak IB, 30 cm x 6 mm column, hexane:iPrOH 95:5, 1.0 mL/min, T = 25°C) ketone 12.1 min, *R* and *S* isomers 20.9 min and 22.3 min, configuration assigned by analogy.

(*R*)-3-(2-Chlorophenyl)-1-(2,6-dimethoxyphenyl)-3-hydroxypropan-1-one **11b.**

(*R,R*)-3C-tethered Ru(II)-TsDPEN catalyst (1.6 mg, 2.5 mmol, 1 mol%) was added to FA: TEA (5:2 azeotropic mixture, 0.36 mL) at rt and the mixture was stirred under a nitrogen atmosphere for 15 minutes; after which a solution of 1-(2-chlorophenyl)-3-(2,6-dimethoxyphenyl)propane-1,3-dione **11a** (80.0 mg, 0.252 mmol) in DCM (0.50 mL) was added. The reaction mixture was stirred under a nitrogen atmosphere and followed by TLC (4:1 hexane: EtOAc). After 24 h, the reaction was quenched using saturated $NaHCO_3$ solution (20 mL). EtOAc (20 mL) was added and the organic layer was separated. The aqueous layer was extracted with EtOAc (3 x 20 mL) and the combined organic layers were dried ($MgSO_4$) and filtered. The solvent was removed to give the crude product. The product was isolated via flash chromatography on silica eluted with 0-50% EtOAc in hexane to give (*R*)-3-(2-chlorophenyl)-1-(2,6-dimethoxyphenyl)-3-hydroxypropan-1-one **11b** as a colorless oil (71.0 mg, 0.222 mmol, 88%). The reaction was also followed by HPLC (Chiralpak IB, 30 cm x 6 mm column, hexane:iPrOH 95:5, 1.0 mL/min, T = 25°C): 100% conversion; $[\alpha]_D^{23}$ +71.6 (c 0.488 in $CHCl_3$) 81% ee (*R*).

^{1}H NMR (500 MHz, $CDCl_3$) of 3-(2-chlorophenyl)-1-(2,6-dimethoxyphenyl)-3-hydroxypropan-1-one **11b.**

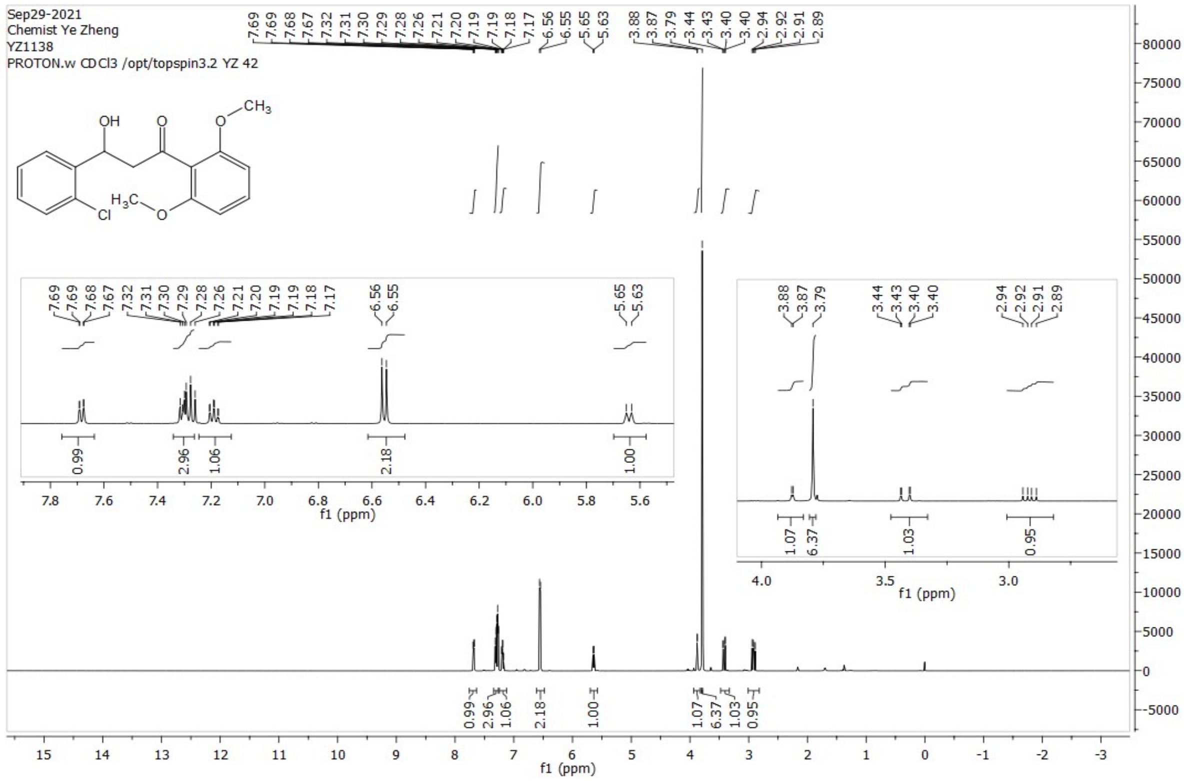

$^{13}C\{^{1}H\}$ NMR (125 MHz, $CDCl_3$) of 3-(2-chlorophenyl)-1-(2,6-dimethoxyphenyl)-3-hydroxypropan-1-one **11b.**

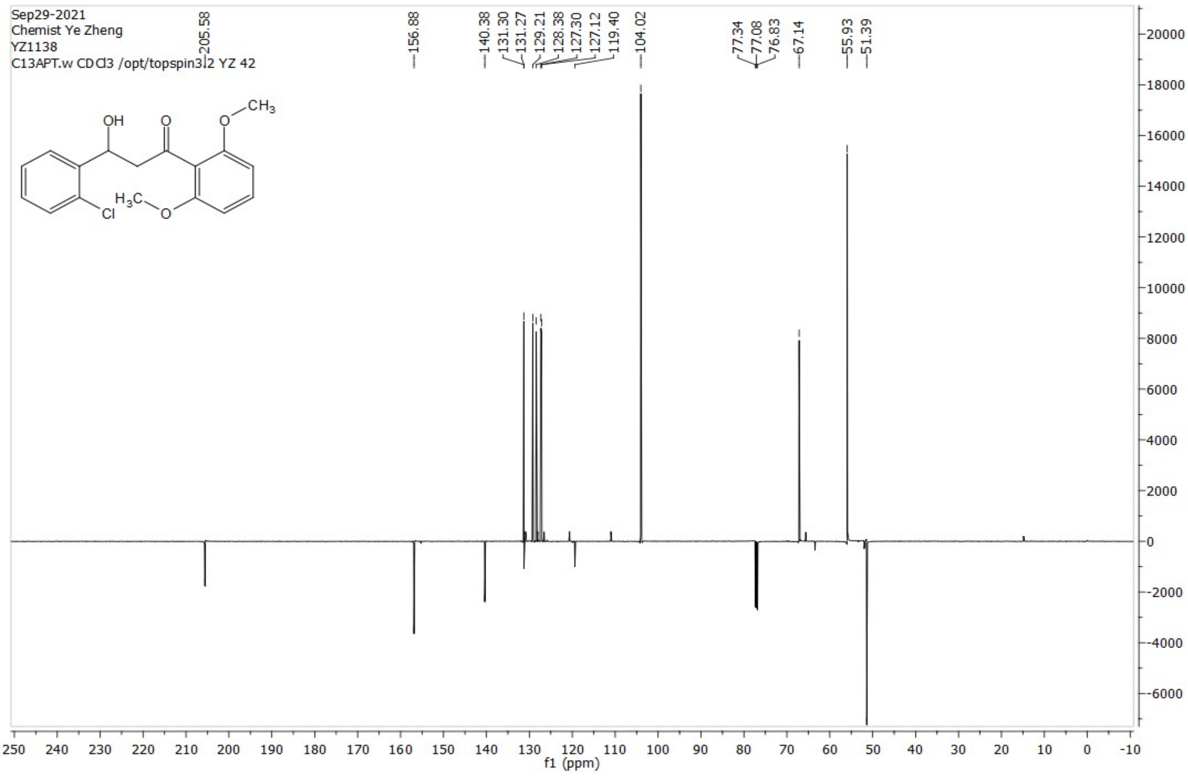

COSY (500 MHz, $CDCl_3$) of 3-(2-chlorophenyl)-1-(2,6-dimethoxyphenyl)-3-hydroxypropan-1-one **11b.**

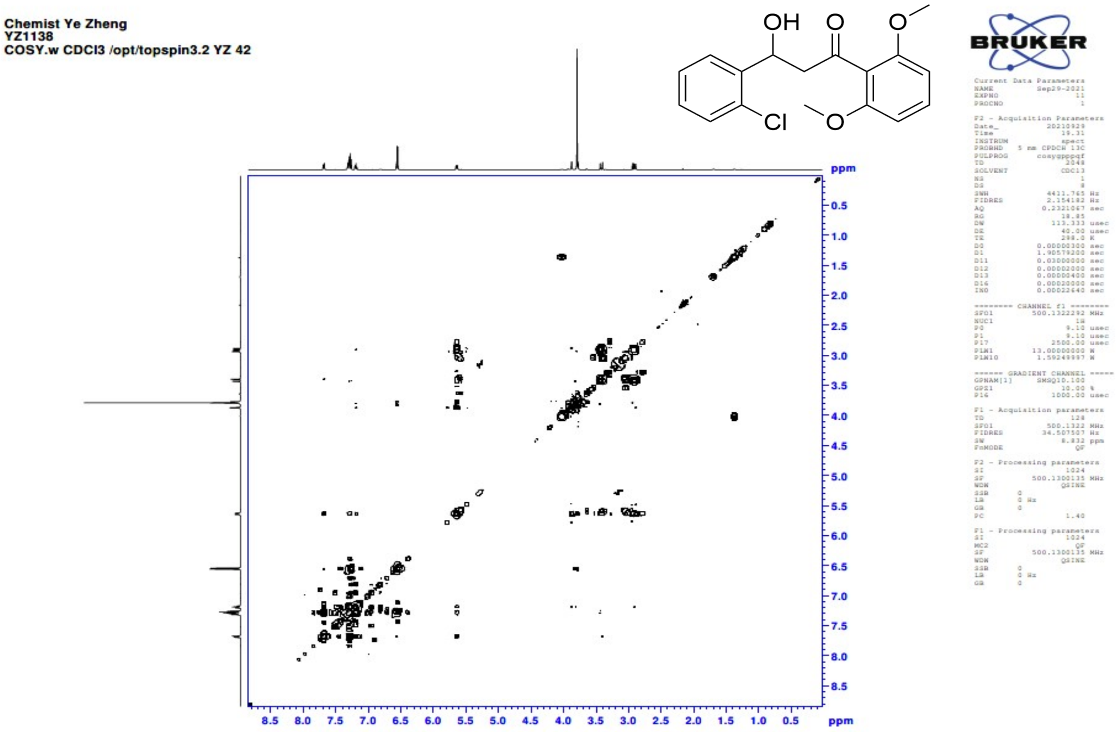

HSQC (500 MHz, $CDCl_3$) of 3-(2-chlorophenyl)-1-(2,6-dimethoxyphenyl)-3-hydroxypropan-1-one **11b.**

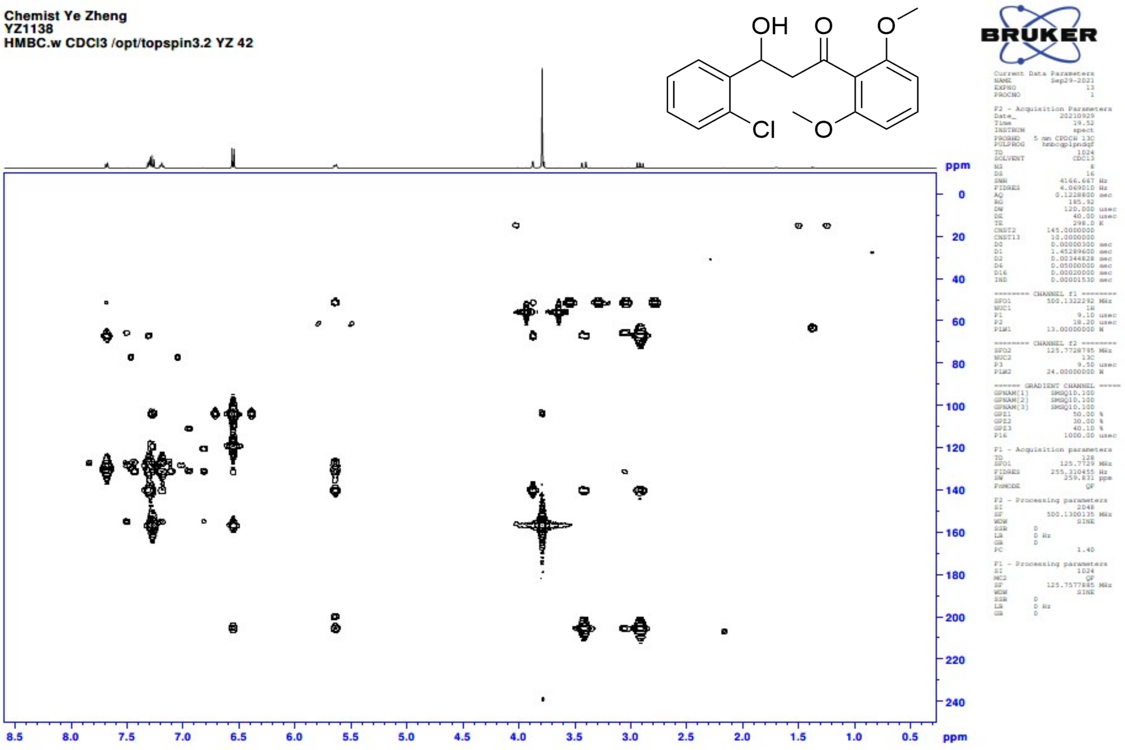

HMBC (500 MHz, $CDCl_3$) of 3-(2-chlorophenyl)-1-(2,6-dimethoxyphenyl)-3-hydroxypropan-1-one **11b.**

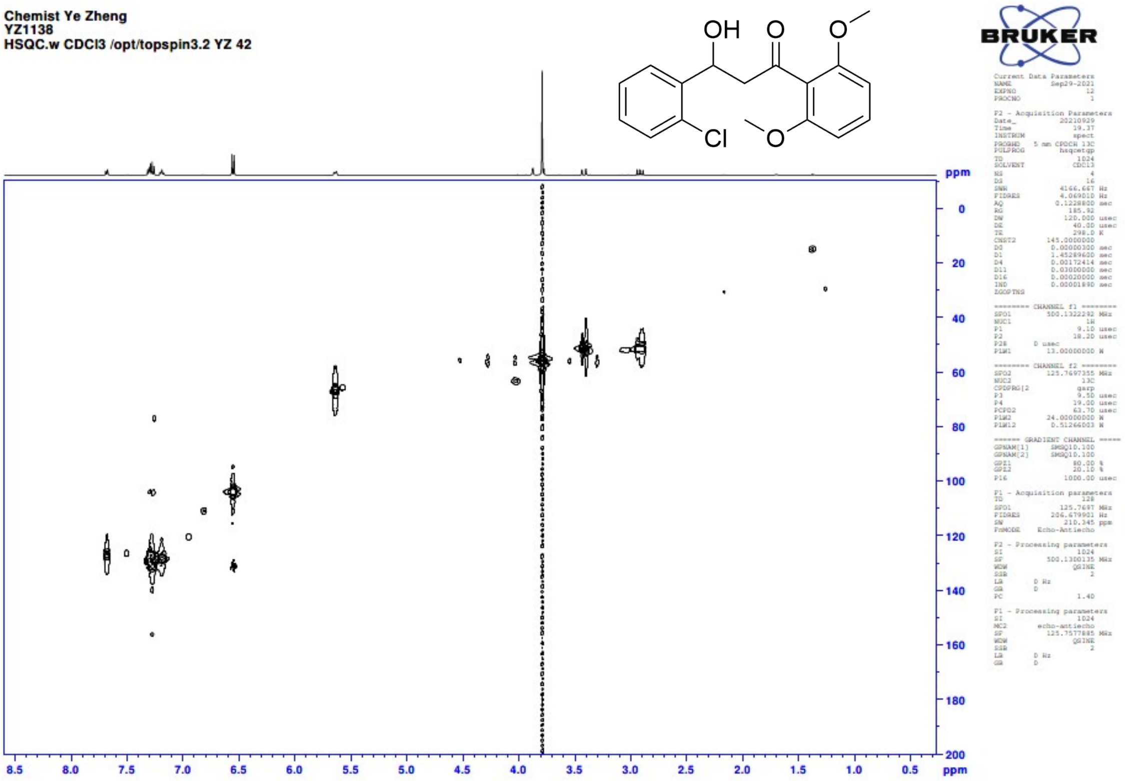

HPLC of racemic 3-(2-chlorophenyl)-1-(2,6-dimethoxyphenyl)-3-hydroxypropan-1-one **11b.**

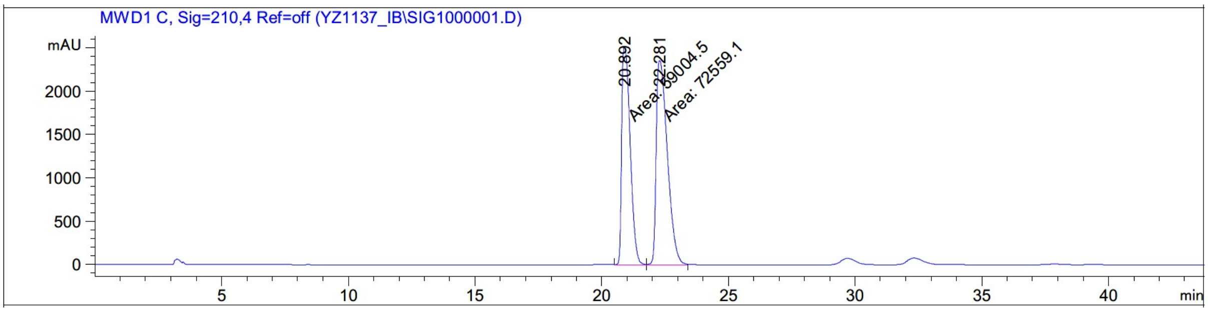

Signal 2: MWD1 C, Sig=210,4 Ref=off

Peak #	RetTime [min]	Type	Width [min]	Area [mAU*s]	Height [mAU]	Area %
1	20.892	MM	0.3905	5.90045e4	2518.52222	44.8486
2	22.281	MM	0.5103	7.25591e4	2369.64087	55.1514
Totals :				1.31564e5	4888.16309	

HPLC of (*R*)-3-(2-chlorophenyl)-1-(2,6-dimethoxyphenyl)-3-hydroxypropan-1-one **11b.** (*R,R*)-3C-Tethered Ru(II)-TsDPEN catalyst (after 24 h, 100% conversion, 81% ee (*R*)).

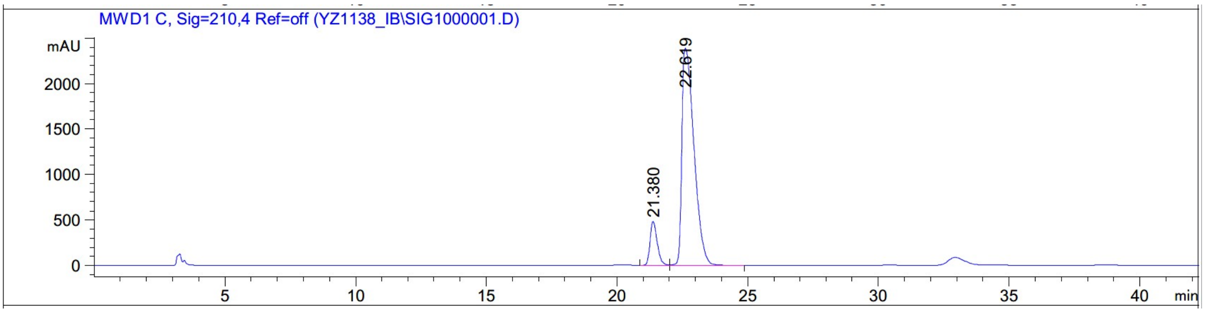

Signal 2: MWD1 C, Sig=210,4 Ref=off

Peak #	RetTime [min]	Type	Width [min]	Area [mAU*s]	Height [mAU]	Area %
1	21.380	VV	0.2924	9214.00684	483.31097	10.7208
2	22.619	VB	0.5009	7.67309e4	2387.60840	89.2792

Totals : 8.59449e4 2870.91937

1-(2,6-Dimethoxyphenyl)-3-(2-methoxyphenyl)propane-1,3-dione 12a.

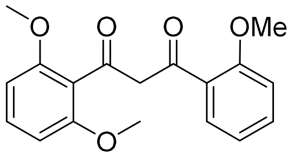

This compound is novel.

To a solution of sodium hydride (408 mg, 60% dispersion in mineral oil, 10.2 mmol) in THF (3 mL) at 0 °C was added dropwise a solution of 1-(2,6-dimethoxyphenyl)ethan-1-one **5** (367 mg, 2.04 mmol) in THF (3 mL). The reaction mixture was stirred under a nitrogen atmosphere at 0 °C for 30 min and then stirred under a nitrogen atmosphere at rt for 30 min, after which a solution of ethyl 2-methoxybenzoate (1.84 g, 10.2 mmol) in THF (3 mL) was added dropwise. The reaction mixture was then heated to 65 °C and left stirring under the nitrogen atmosphere overnight. The reaction was followed by TLC (4:1 hexane: EtOAc). The mixture was quenched by 2M HCl solution (20 mL). EtOAc (20 mL) was added and the organic layer was separated. The aqueous layer was extracted with EtOAc (3 × 20 mL), and the combined organic layers were washed with saturated $NaHCO_3$ solution (2 × 20 mL) and brine (20 mL), dried ($MgSO_4$) and filtered. Solvent was removed to give the crude product. The product was isolated via flash chromatography on silica eluted with 0-50% EtOAc in hexane to give 1-(2,6-dimethoxyphenyl)-3-(2-methoxyphenyl)propane-1,3-dione **12a** as a yellow oil (330 mg, 1.05 mmol, 52%). TLC: Rf ca 0.20 (4:1 hexane: EtOAc), strong UV and $KMnO_4$; HRMS (ESI+) *m/z*: [M+H]+ Calcd for $C_{18}H_{18}NaO_8$ 337.1042; Found 337.1046; 1.3 ppm error; ν_{max} 3010, 2934, 2837, 1584, 1507, 1469, 1431, 1283, 1245, 1105, 1053, 1018 cm^{-1}; enol: keto = 17:3; ^1H NMR (400 MHz, $CDCl_3$): δ 7.98 (1H, dd, J = 7.8, 1.4, ArH), 7.47-7.43 (1H, m, ArH), 7.36-7.29 (1H, m, ArH), 7.06 (1H, t, J = 7.5, ArH),6.99-6.92 (1H, m, ArH), 6.68 (0.85H, s, CH of enol form), 6.64 (2H, d, J = 8.4, ArH), 4.53 (0.30H, s, CH_2 of keto form), 3.89 (3H, s, CH_3), 3.85 (5.1H, s, OCH_3 of enol form), 3.74 (0.9H, s, OCH_3 of keto form); $^{13}C\{^1H\}$ NMR (100 MHz, $CDCl_3$): δ keto form: 190.1 (C), 178.9 (C), 158.6 (C), 157.4 (C), 133.8 (CH), 131.4 (CH), 130.8 (CH), 124.3 (C), 120.5 (CH), 117.6 (C), 111.3 (CH), 105.7 (CH), 59.9 (CH_2), 55.8 (CH_3), 55.3 (CH_3) ppm; enol form: 189.7 (C), 179.9 (C), 158.6 (C), 157.8 (C), 132.8 (CH), 131.0 (CH), 130.3 (CH), 124.3 (C), 120.7 (CH), 117.6 (C), 111.6 (CH), 105.6 (CH), 104.2 (CH), 56.1 (CH_3), 55.7 (CH_3); *m/z* (ES-API+) 337.1 (M^+ + 23, 100%).

Enantiomeric excess and conversion determined by HPLC analysis (Chiralpak IB, 30 cm x 6 mm column, hexane:iPrOH 90:10, 1.0 mL/min, T = 25°C) ketone 15.0 min, *R* and *S* isomers 23.2 min and 25.6 min, configuration assigned by analogy.

^{1}H NMR (400 MHz, $CDCl_3$) of 1-(2,6-dimethoxyphenyl)-3-(2-methoxyphenyl)propane-1,3-dione **12a.**

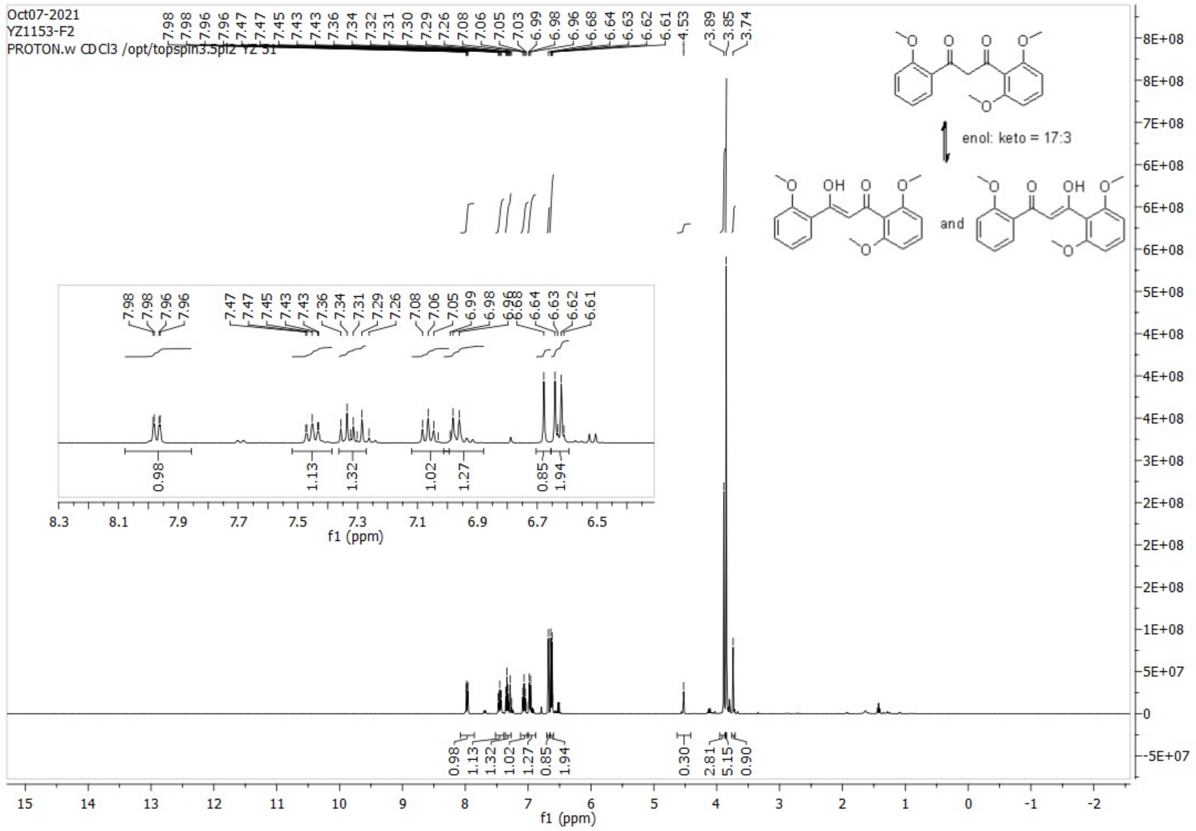

$^{13}C\{^{1}H\}$ NMR (100 MHz, $CDCl_3$) of 1-(2,6-dimethoxyphenyl)-3-(2-methoxyphenyl)propane-1,3-dione **12a.**

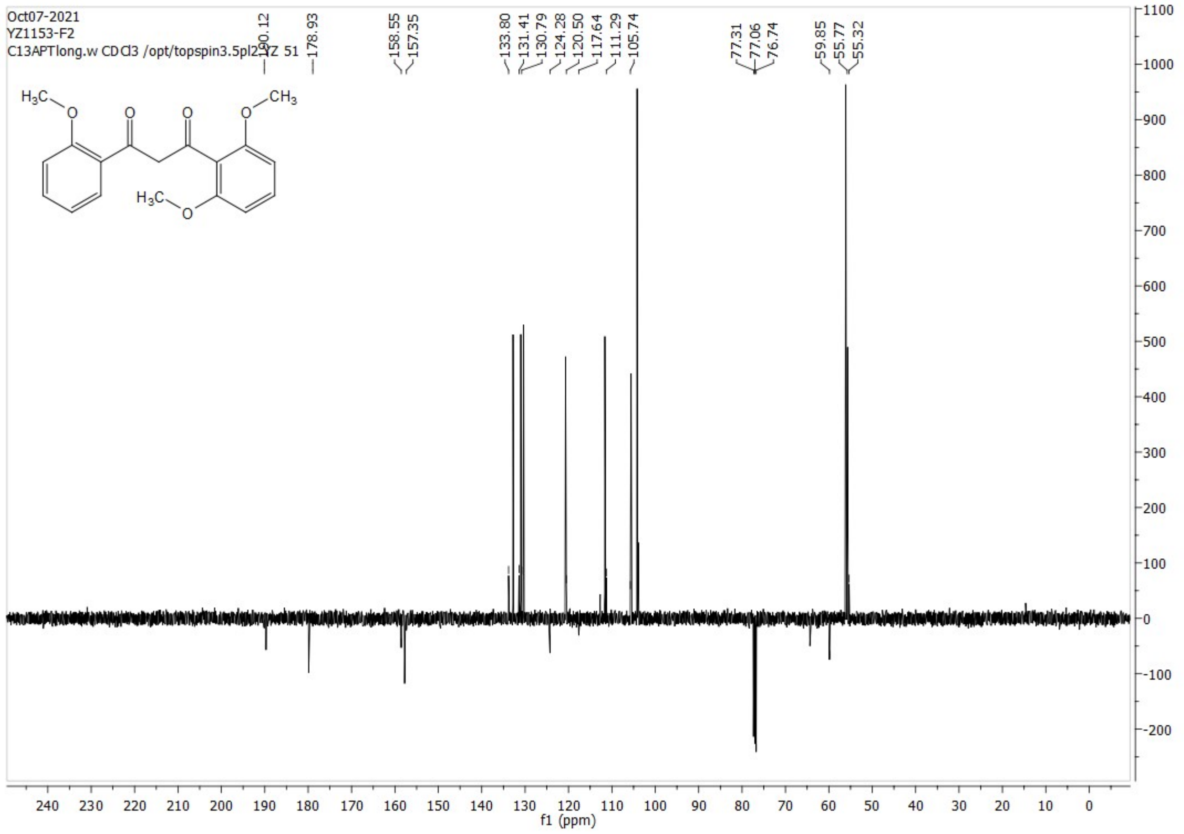

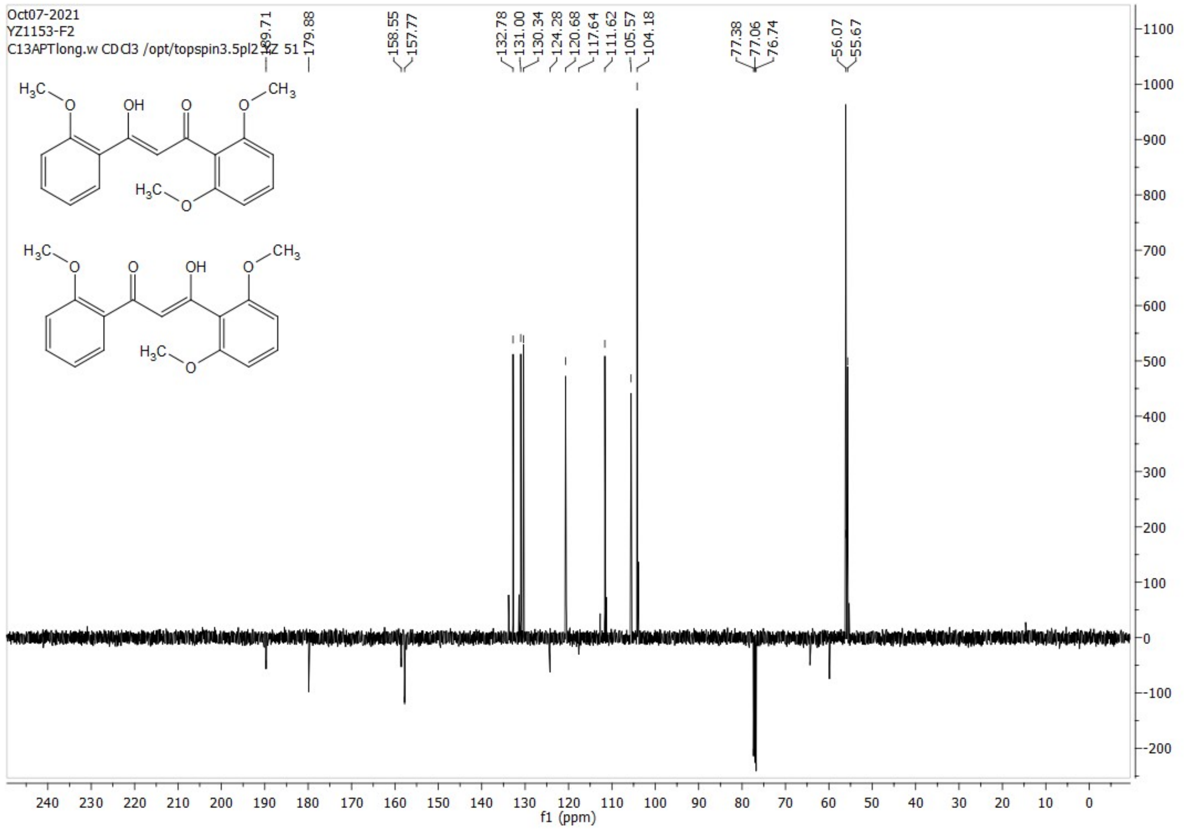

COSY (400 MHz, $CDCl_3$) of 1-(2,6-dimethoxyphenyl)-3-(2-methoxyphenyl)propane-1,3-dione **12a.**

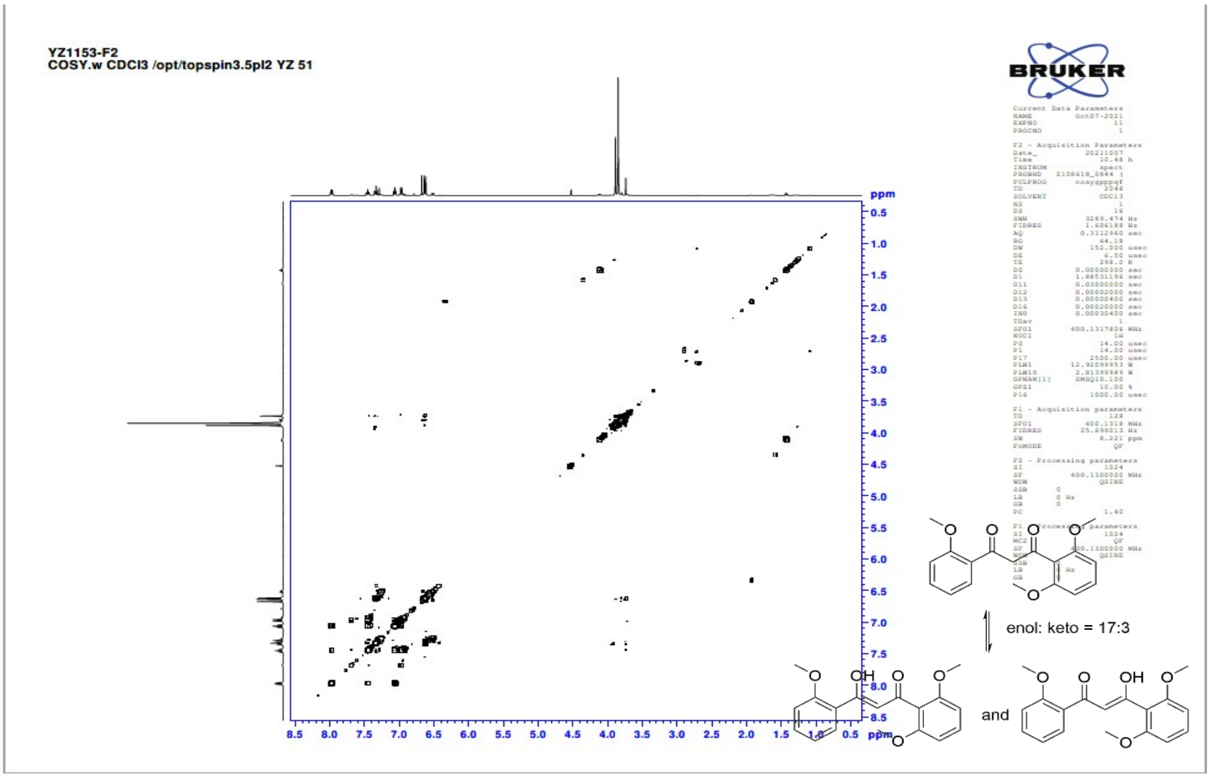

HSQC (400 MHz, $CDCl_3$) of 1-(2,6-dimethoxyphenyl)-3-(2-methoxyphenyl)propane-1,3-dione

12a.

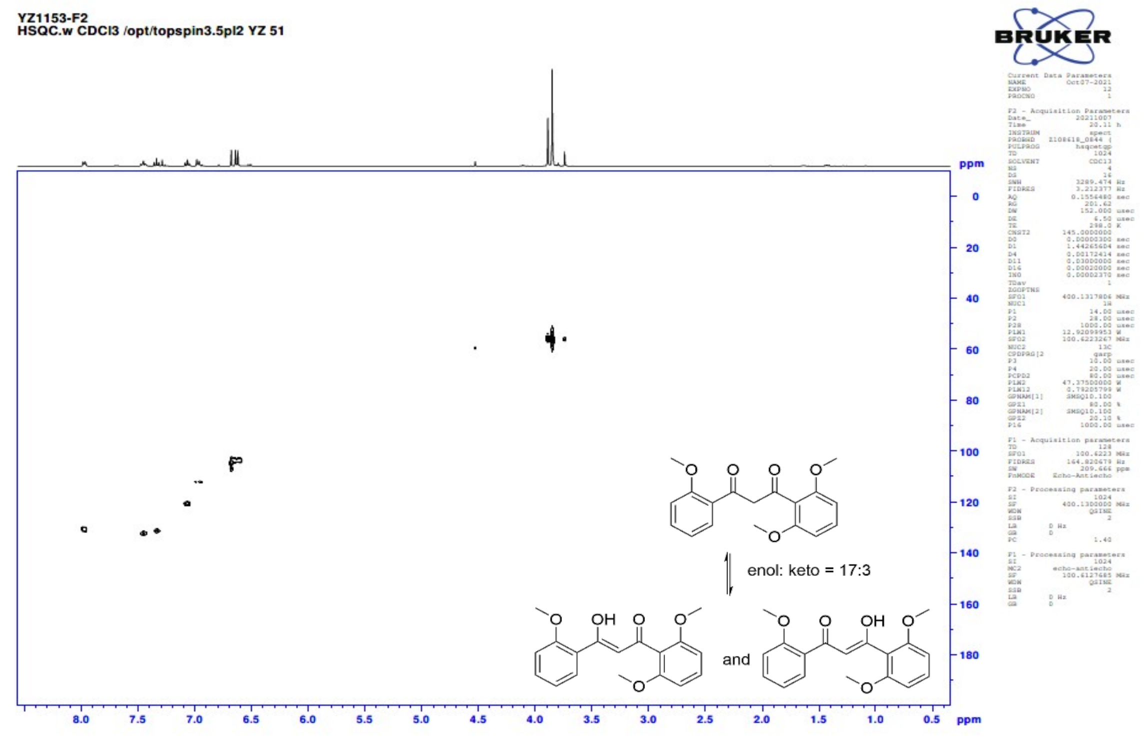

HMBC (400 MHz, $CDCl_3$) of 1-(2,6-dimethoxyphenyl)-3-(2-methoxyphenyl)propane-1,3-dione **12a.**

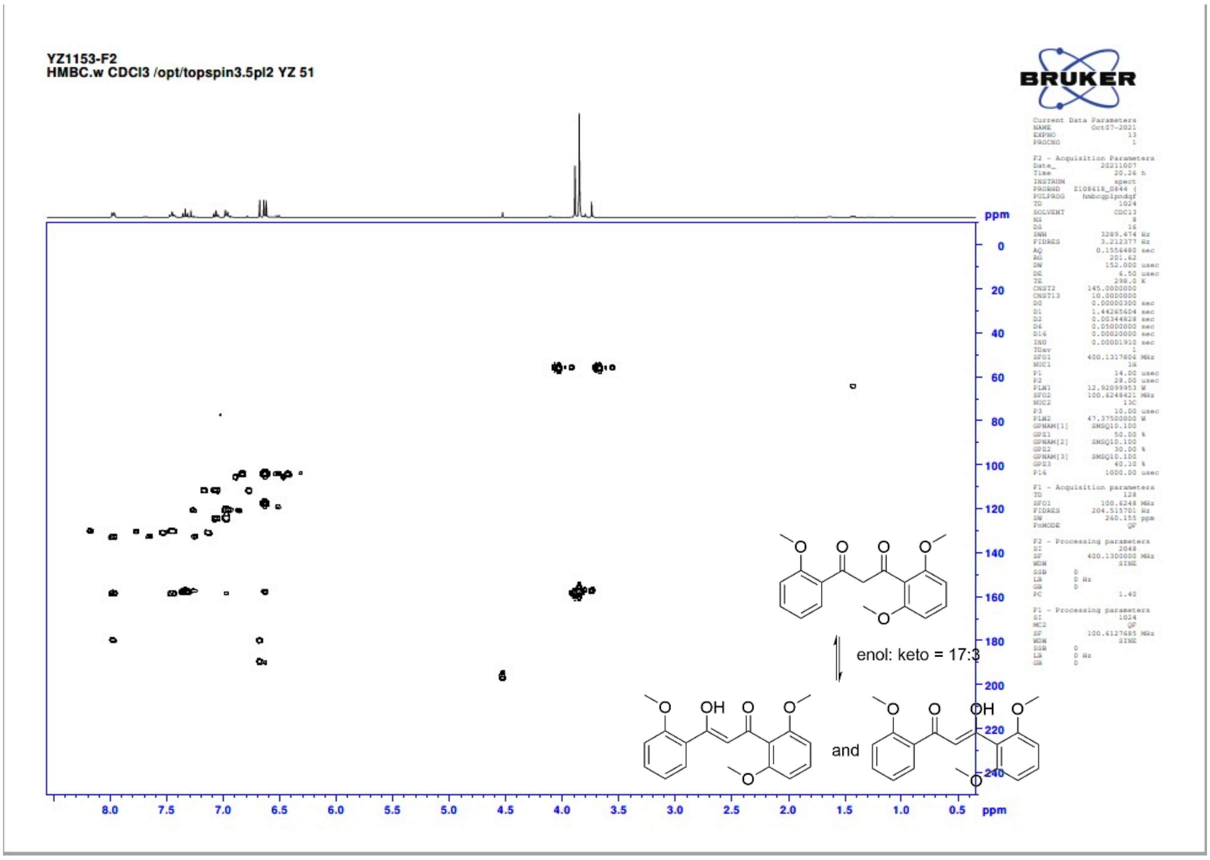

HPLC of 1-(2,6-dimethoxyphenyl)-3-(2-methoxyphenyl)propane-1,3-dione **12a.**

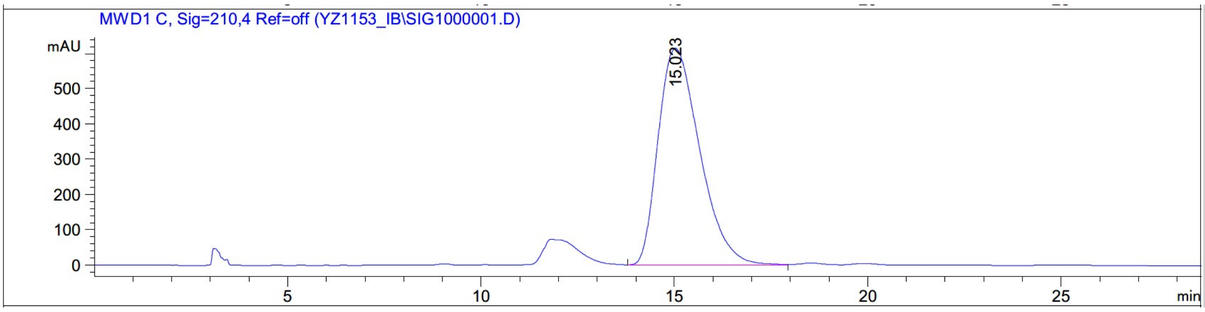

Signal 2: MWD1 C, Sig=210,4 Ref=off

Peak #	RetTime [min]	Type	Width [min]	Area [mAU*s]	Height [mAU]	Area %
1	15.023	VV	1.1558	4.60435e4	614.77106	100.0000

| Totals : | | | | 4.60435e4 | 614.77106 | |

1-(2,6-Dimethoxyphenyl)-3-(2-methoxyphenyl)propane-1,3-dione 12b.

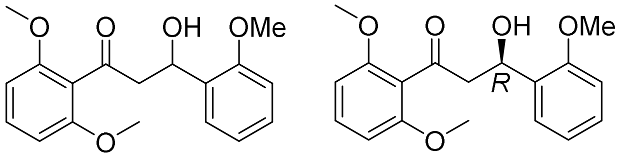

This compound is novel.

Synthesis of a racemic standard: (*R,R*)-3C-Tethered Ru(II)-TsDPEN catalyst (0.80 mg, 1.3 mmol, 0.5 mol%) and (*S,S*)-3C-tethered Ru(II)-TsDPEN catalyst (0.80 mg, 1.3 mmol, 0.5 mol%) were added to FA: TEA (5:2 azeotropic mixture, 0.36 mL) at rt and the mixture was stirred under a nitrogen atmosphere for 15 minutes; after which a solution of 1-(2,6-dimethoxyphenyl)-3-(2-methoxyphenyl)propane-1,3-dione **12a** (80.0 mg, 0.255 mmol) in DCM (0.50 mL) was added. The reaction mixture was stirred under a nitrogen atmosphere and followed by TLC (4:1 hexane: EtOAc). After 24 h, the reaction was quenched using saturated $NaHCO_3$ solution (20 mL). EtOAc (20 mL) was added and the organic layer was separated. The aqueous layer was extracted with EtOAc (3 x 20 mL) and the combined organic layers were dried ($MgSO_4$) and filtered. The solvent was removed to give the crude product. The product was isolated via flash chromatography on silica eluted with 0-50% EtOAc in hexane to 1-(2,6-dimethoxyphenyl)-3-hydroxy-3-(2-methoxyphenyl)propan-1-one **12b** as a colorless oil (48.0 mg, 0.152 mmol, 60%). TLC: Rf ca 0.20 (4:1 hexane: EtOAc), strong UV and $KMnO_4$; HRMS (ESI+) *m/z*: [M+H]+ Calcd for $C_{18}H_{20}NaO_5$ 339.1199; Found 339.1203; 1.2 ppm error; ν_{max} 3485 (br), 2935, 2837, 1694, 1610, 1591, 1511, 1470, 1432, 1302, 1287, 1245, 1172, 1105, 1026, 830, 779 cm^{-1}; ^1H NMR (500 MHz, $CDCl_3$): δ 7.44 (1H, dd, *J* = 7.5, 1.3, ArH), 7.20-7.14 (2H, m, ArH), 6.89 (1H, t, *J* = 7.5, ArH), 6.78 (1H, d, *J* = 8.3, ArH), 6.48 (2H, d, *J* = 8.3, ArH), 5.50 (1H, dt, *J* = 9.1, 3.1, ArCH), 3.74 (3H, s, CH_3), 3.70 (6H, s, CH_3), 3.56 (1H, d, *J* = 3.9, OH), 3.28 (1H, dd, *J* = 17.5, 2.8, CH_2), 3.00 (1H, dd, *J* = 17.5, 9.1, CH_2); $^{13}C\{^1H\}$ NMR (125 MHz, $CDCl_3$): δ 205.6 (C), 156.9 (C), 155.9 (C), 131.2 (C), 131.0 (CH), 128.2 (CH), 126.7 (CH), 120.7 (CH), 119.9 (C), 110.2 (CH), 104.1 (CH), 65.6 (CH), 55.9 (CH_3), 55.3 (CH_3), 51.7 (CH_2); *m/z* (ES-API+) 339.1 (M^+ + 23, 100%).

Enantiomeric excess and conversion determined by HPLC analysis (Chiralpak IB, 30 cm x 6 mm column, hexane:iPrOH 90:10, 1.0 mL/min, T = 25°C) ketone 15.0 min, *R* and *S* isomers 23.2 min and 25.6 min, configuration assigned by analogy.

(*R*)-1-(2,6-Dimethoxyphenyl)-3-(2-methoxyphenyl)propane-1,3-dione **12b.** (*R,R*)-3C-tethered Ru(II)-TsDPEN catalyst (1.6 mg, 2.6 mmol, 1 mol%) was added to FA: TEA (5:2 azeotropic mixture, 0.36 mL) at rt and the mixture was stirred under a nitrogen atmosphere for 15 minutes; after which a solution of 1-(2,6-dimethoxyphenyl)-3-(2-methoxyphenyl)propane-1,3-dione **12a** (80.0 mg, 0.255 mmol) in DCM (0.50 mL) was added. The reaction mixture was stirred under a nitrogen atmosphere and followed by TLC (4:1 hexane: EtOAc). After 24 h, the reaction was quenched using saturated $NaHCO_3$ solution (20 mL). EtOAc (20 mL) was added and the organic layer was separated. The aqueous layer was extracted with EtOAc (3 x 20 mL) and the combined organic layers were dried ($MgSO_4$) and filtered. The solvent was removed to give the crude product. The product was isolated via flash chromatography on silica eluted with 0-50% EtOAc in hexane to give (*R*)-1-(2,6-dimethoxyphenyl)-3-hydroxy-3-(2-methoxyphenyl)propan-1-one **12b** as a colorless oil (52.0 mg, 0.165 mmol, 65%). The reaction was also followed by HPLC (Chiralpak IB, 30 cm x 6 mm column, hexane:iPrOH 90:10, 1.0 mL/min, T = 25°C): 100% conversion; $[\alpha]_D^{23}$ +56.2 (c 0.340 in $CHCl_3$) 83% ee (*R*).

^{1}H NMR (500 MHz, $CDCl_3$) of 1-(2,6-dimethoxyphenyl)-3-(2-methoxyphenyl)propane-1,3-dione **12b.**

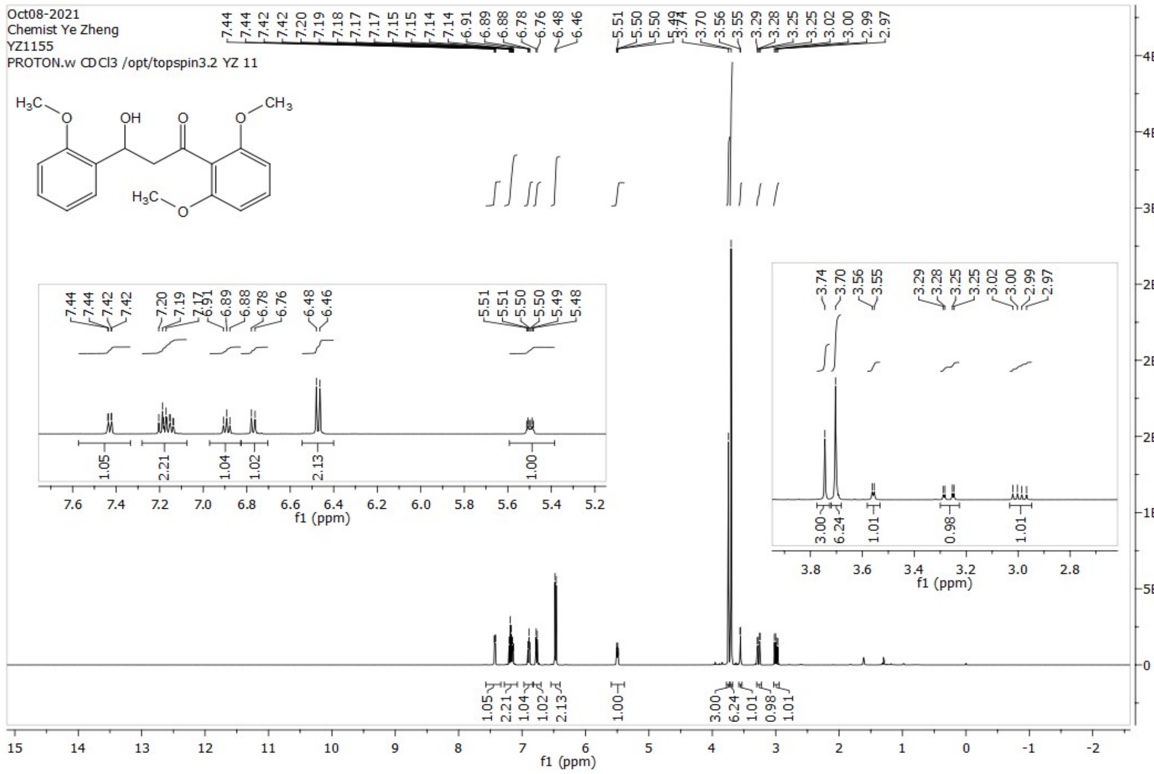

^{13}C{^{1}H} NMR (125 MHz, $CDCl_3$) of 1-(2,6-dimethoxyphenyl)-3-(2-methoxyphenyl)propane-1,3-dione **12b.**

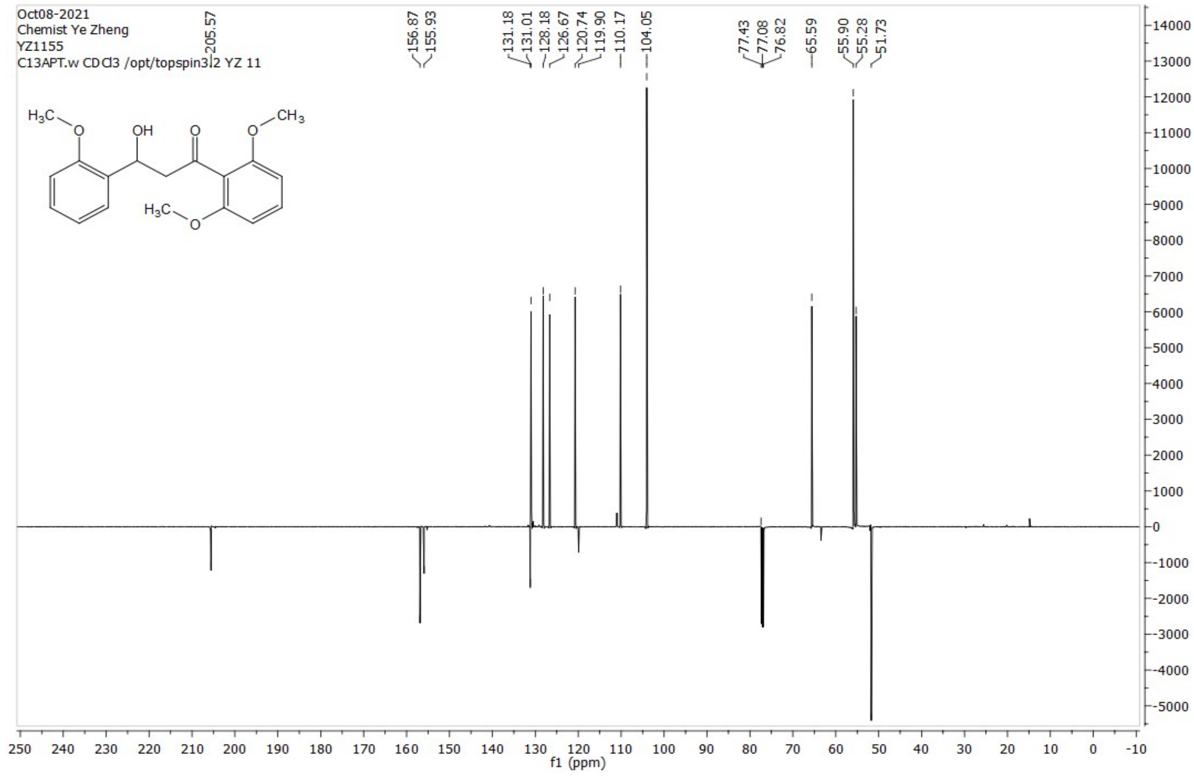

COSY (500 MHz, $CDCl_3$) of 1-(2,6-dimethoxyphenyl)-3-(2-methoxyphenyl)propane-1,3-dione **12b.**

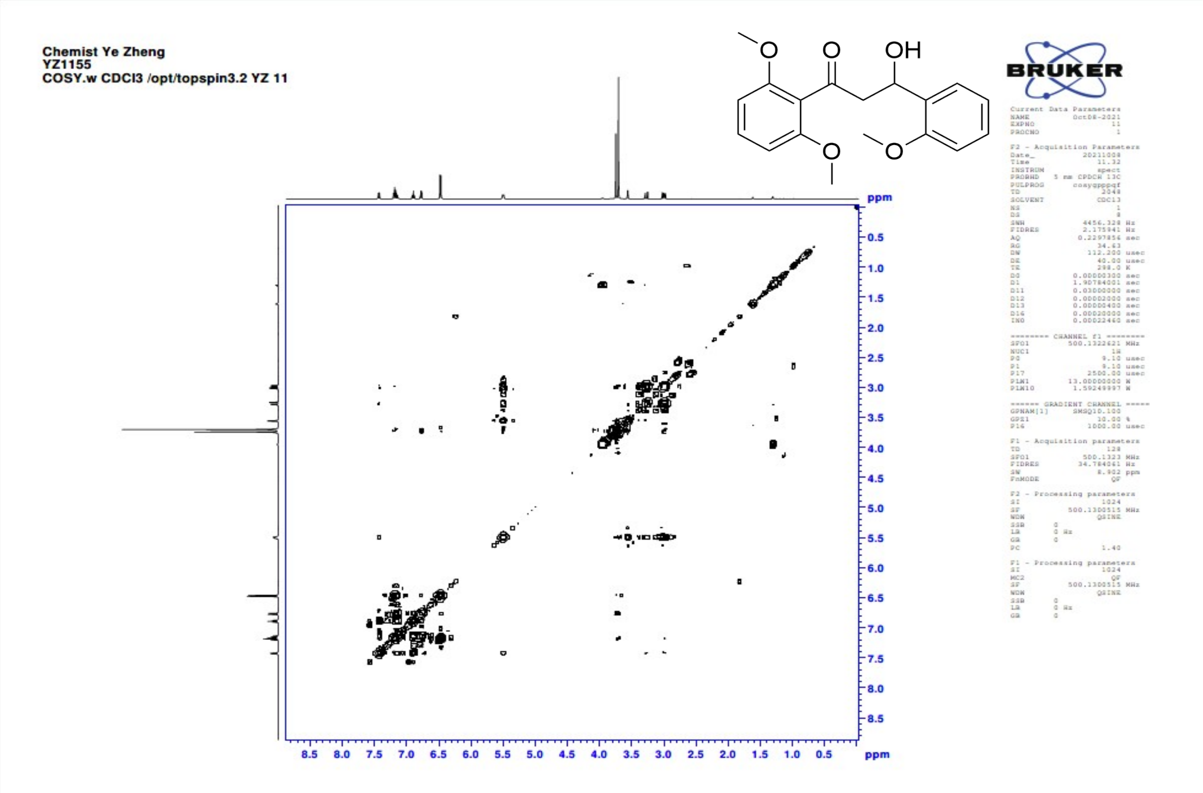

HSQC (500 MHz, $CDCl_3$) of 1-(2,6-dimethoxyphenyl)-3-(2-methoxyphenyl)propane-1,3-dione **12b.**

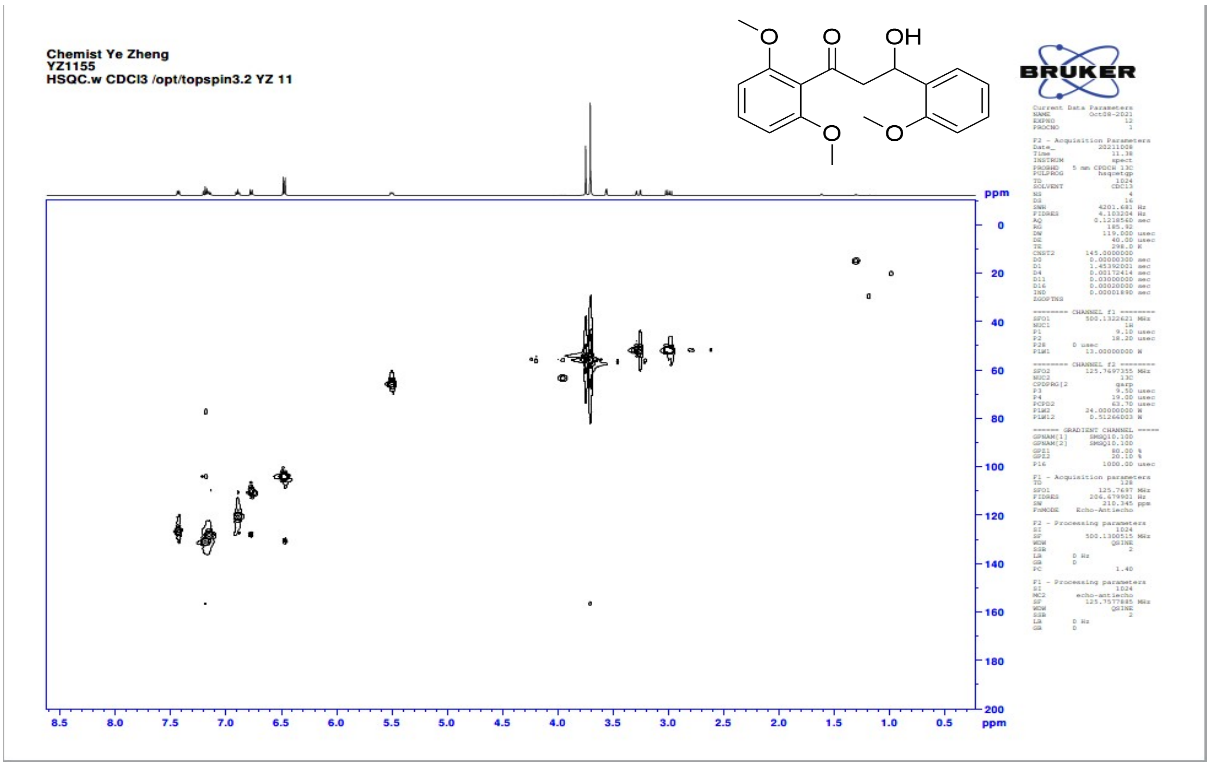

HMBC (500 MHz, $CDCl_3$) of 1-(2,6-dimethoxyphenyl)-3-(2-methoxyphenyl)propane-1,3-dione **12b.**

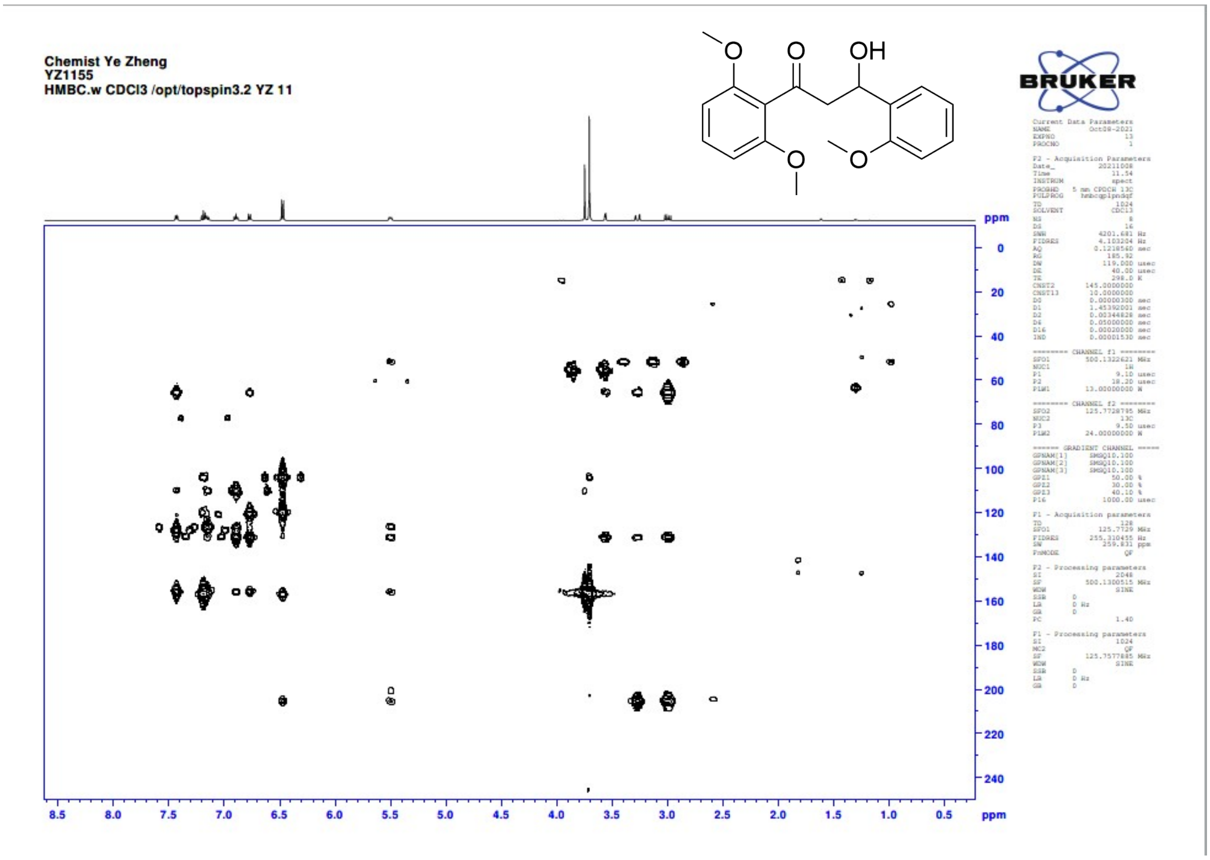

HPLC of racemic 1-(2,6-dimethoxyphenyl)-3-hydroxy-3-(2-methoxyphenyl)propan-1-one **12b.**

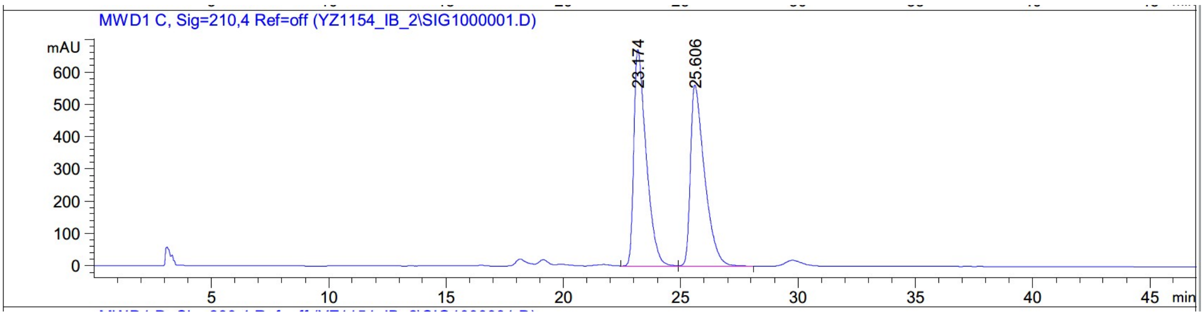

Signal 2: MWD1 C, Sig=210,4 Ref=off

Peak #	RetTime [min]	Type	Width [min]	Area [mAU*s]	Height [mAU]	Area %
1	23.174	BV	0.5422	2.45202e4	671.13196	51.1414
2	25.606	VB	0.6091	2.34257e4	561.04987	48.8586

Totals : 4.79459e4 1232.18182

(*R*)-1-(2,6-Dimethoxyphenyl)-3-(2-methoxyphenyl)propane-1,3-dione **12b.** (*R,R*)-3C-Tethered Ru(II)-TsDPEN catalyst (after 24 h, 100% conversion, 83% ee, (*R*))

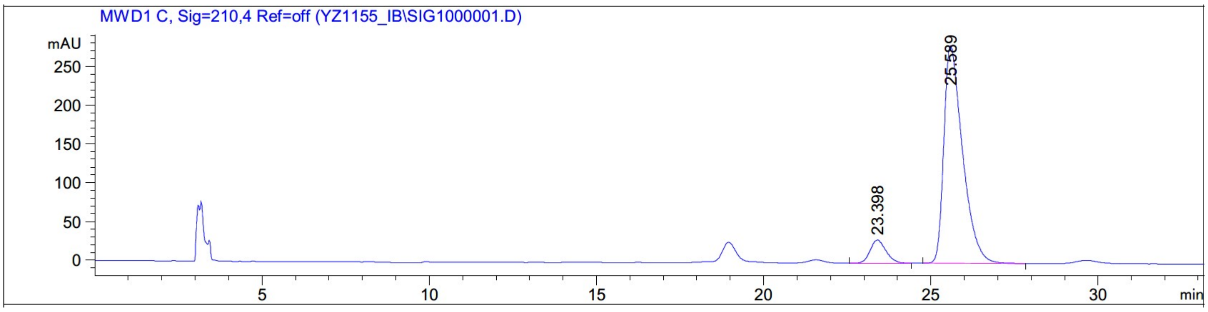

Signal 2: MWD1 C, Sig=210,4 Ref=off

Peak #	RetTime [min]	Type	Width [min]	Area [mAU*s]	Height [mAU]	Area %
1	23.398	BB	0.5064	989.27124	30.02560	8.2983
2	25.589	BB	0.5801	1.09321e4	280.87372	91.7017

Totals : 1.19214e4 310.89932

1-(2,6-Dimethoxyphenyl)-3-(furan-2-yl)propane-1,3-dione 13a.

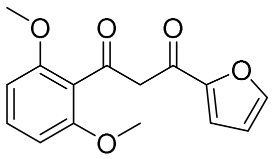

This compound is novel.

To a solution of sodium hydride (200 mg, 60% dispersion in mineral oil, 5.00 mmol) in THF (4 mL) at 0 °C was added dropwise a solution of 1-(2,6-dimethoxyphenyl)ethan-1-one **5** (180 mg, 1.00 mmol) in THF (4 mL). The reaction mixture was stirred under a nitrogen atmosphere at 0 °C for 30 min and then stirred under a nitrogen atmosphere at rt for 30 min, after which ethyl furan-2-carboxylate (700 mg, 5.00 mmol) was added dropwise. The reaction mixture was then heated to 65 °C and left stirring under the nitrogen atmosphere overnight. The reaction was followed by TLC (4:1 hexane: EtOAc). The mixture was quenched by 2M HCl solution (20 mL). EtOAc (20 mL) was added and the organic layer was separated. The aqueous layer was extracted with EtOAc (3 × 20 mL) and the combined organic layers were dried ($MgSO_4$) and filtered. Solvent was removed to give the crude product. The product was isolated via flash chromatography on silica eluted with 0-50% EtOAc in hexane to give 1-(2,6-dimethoxyphenyl)-3-(furan-2-yl)propane-1,3-dione **13a** as a yellow solid (240 mg, 0.876 mmol, 88%). TLC: Rf ca 0.40 (4:1 hexane: EtOAc), strong UV and $KMnO_4$; Mp: 99 °C; HRMS (ESI+) *m/z*: [M+H]+ Calcd for $C_{15}H_{14}NaO_5$ 297.0729; Found 297.0733; 1.6 ppm error; ν_{max} 3151, 2946, 2842, 1588, 1468, 1429, 1248, 1223, 1106, 1087, 1011 cm^{-1}; enol: keto = 83:17; ^1H NMR (400 MHz, $CDCl_3$): δ 15.61 (1H, s, OH of enol form), 7.55 (1H, s, ArH), 7.31 (1H, d, J = 8.4, ArH), 7.16 (1H, d, J = 3.5, ArH), 6.60 (2H, d, J = 8.4, H of furan), 6.54-6.52 (1H, d, J = 8.4, H of furan), 6.28 (0.83H, s, CH of enol form), 4.27 (0.34H, s, CH_2 of keto form), 3.81 (4.98H, s, OCH_3 of enol form), 3.75 (1.02H, s, OCH_3 of keto form); $^{13}C\{^1H\}$ NMR (100 MHz, $CDCl_3$): δ keto form: 196.7 (C), 192.1 (C), 157.0 (C), 150.5 (C), 146.8 (CH), 131.6 (CH), 118.6 (CH), 116.0 (C), 112.4 (CH), 104.0 (CH), 55.9 (CH_3), 55.5 (CH_2); enol form: 185.2 (C), 174.6 (C), 157.8 (C), 150.5 (C), 145.9 (CH), 131.3 (CH), 116.0 (C), 115.5 (CH), 112.5 (CH), 104.1 (CH), 100.1 (CH), 56.1 (CH_3); *m/z* (ES-API+) 297.1 (M^+ + 23, 100%).

Enantiomeric excess and conversion determined by HPLC analysis (Chiralcel ODH, 30 cm x 6 mm column, hexane:iPrOH 90:10, 1.0 mL/min, T = 25°C) ketone 14.7 min, *R* and *S* isomers 29.8 min and 34.1 min, configuration assigned by analogy.

^{1}H NMR (400 MHz, $CDCl_3$) of 1-(2,6-dimethoxyphenyl)-3-(furan-2-yl)propane-1,3-dione **13a.**

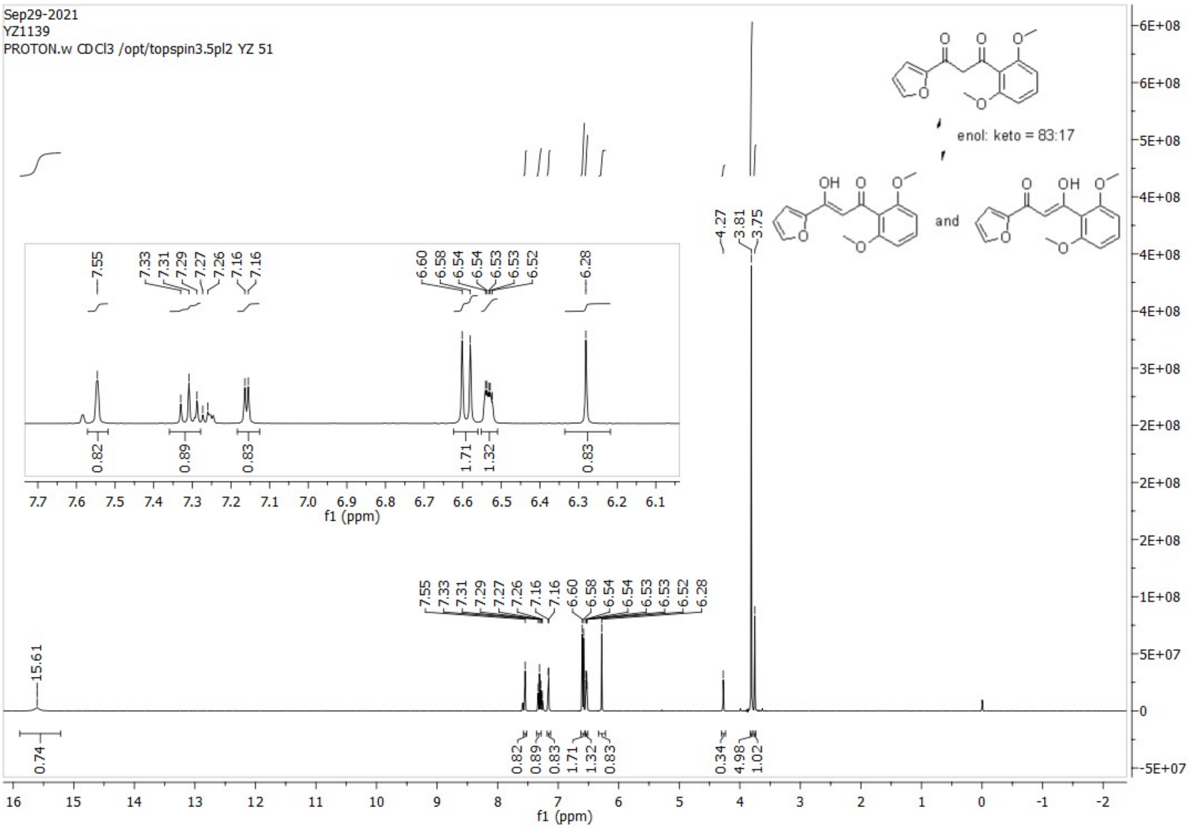

$^{13}C\{^{1}H\}$ NMR (100 MHz, $CDCl_3$) of 1-(2,6-dimethoxyphenyl)-3-(furan-2-yl)propane-1,3-dione **13a.**

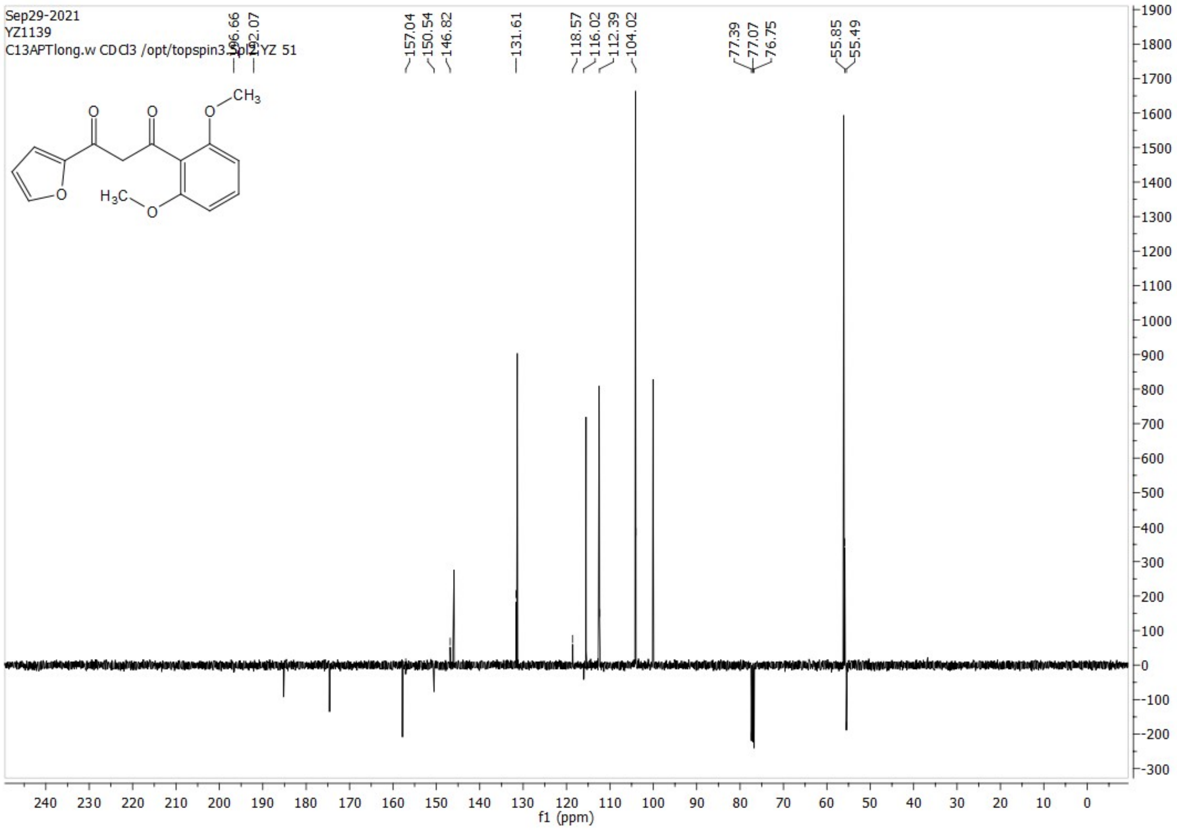

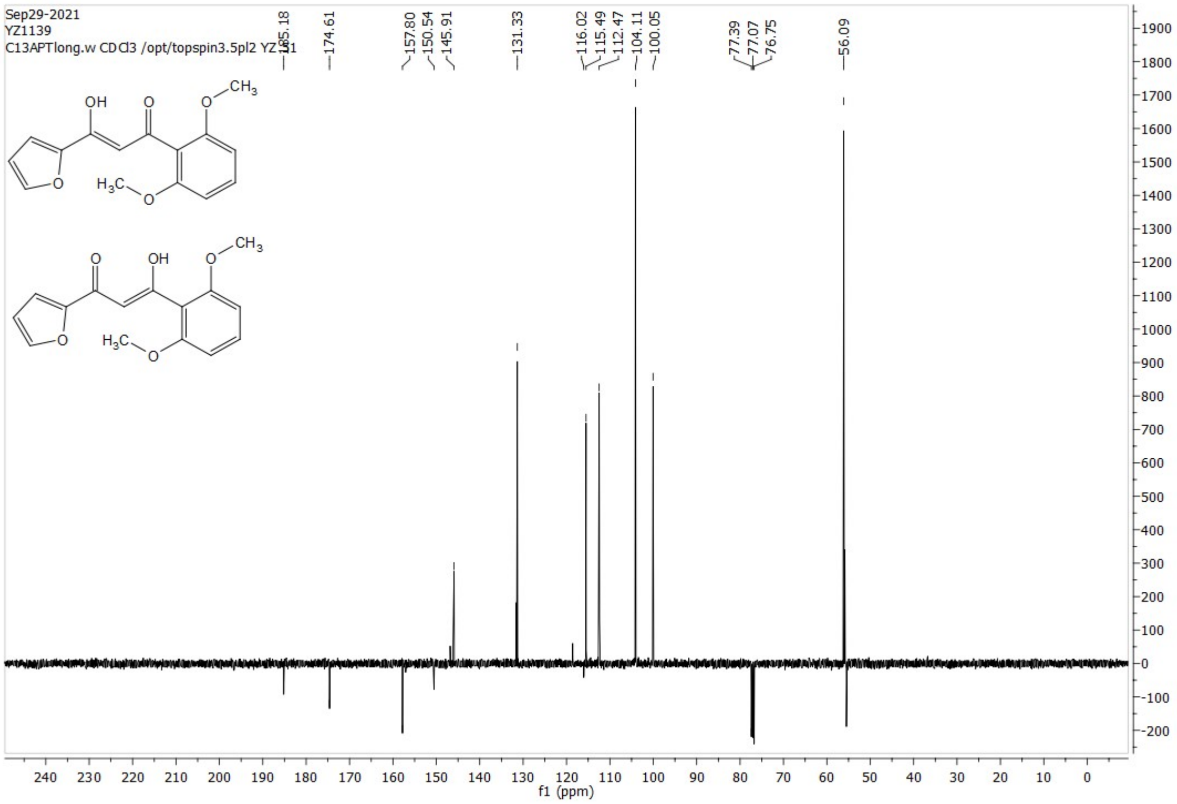

COSY (400 MHz, $CDCl_3$) of 1-(2,6-dimethoxyphenyl)-3-(furan-2-yl)propane-1,3-dione **13a.**

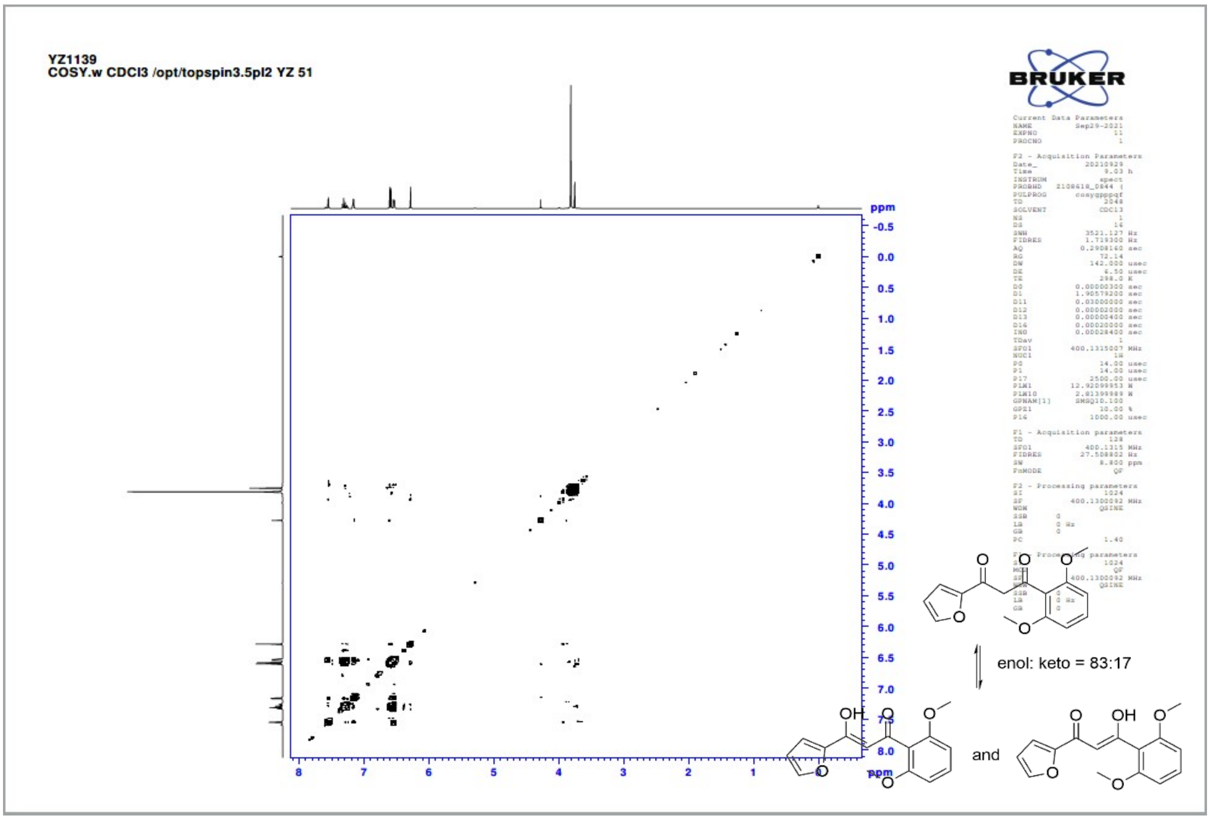

HSQC (400 MHz, $CDCl_3$) of 1-(2,6-dimethoxyphenyl)-3-(furan-2-yl)propane-1,3-dione **13a.**

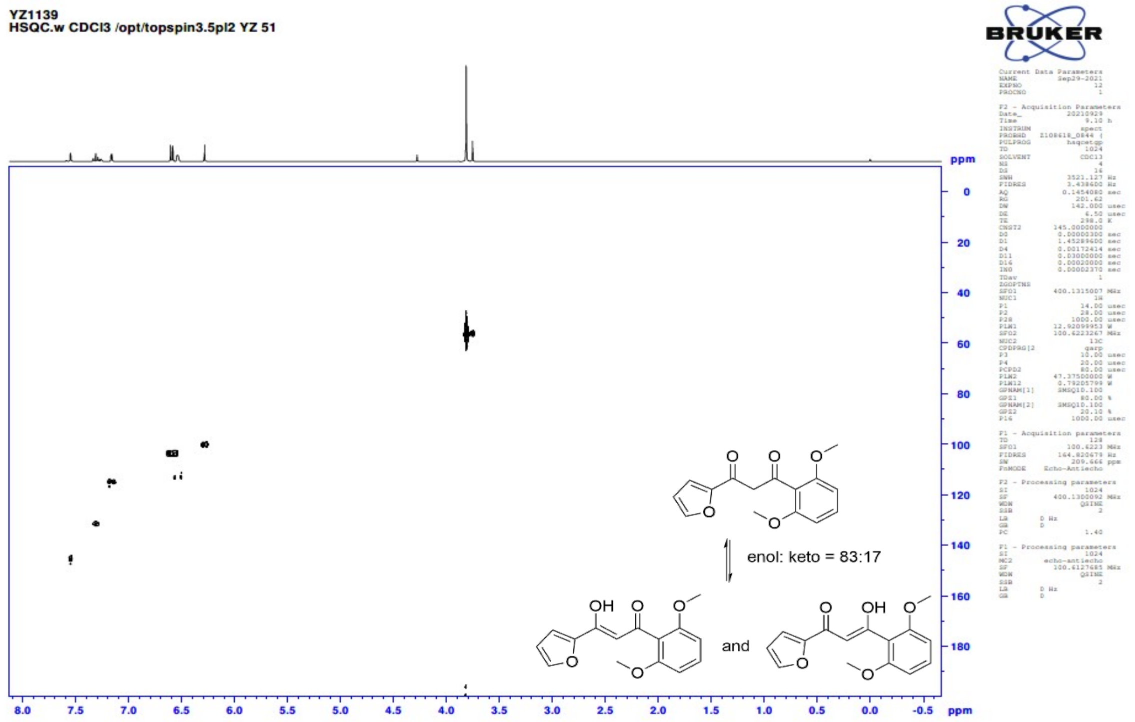

HMBC (400 MHz, $CDCl_3$) of 1-(2,6-dimethoxyphenyl)-3-(furan-2-yl)propane-1,3-dione **13a.**

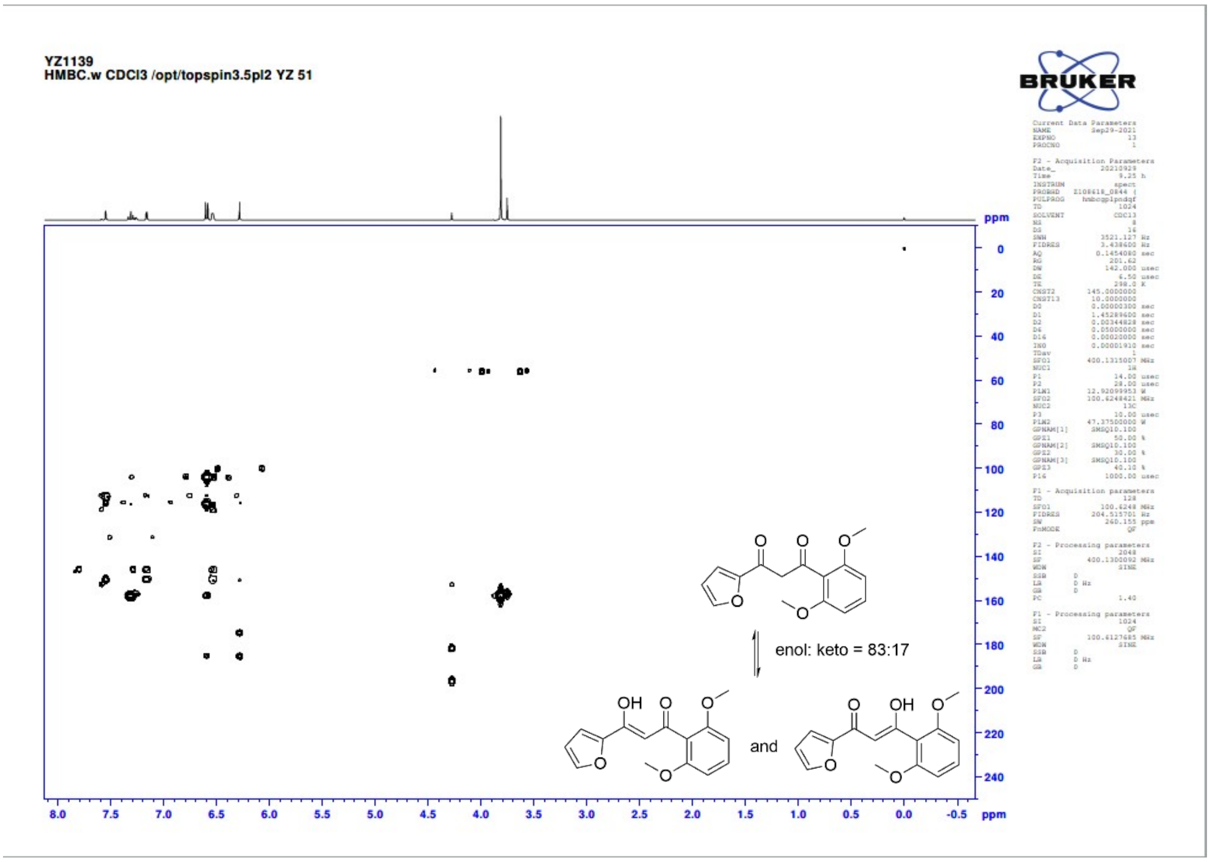

HPLC of 1-(2,6-dimethoxyphenyl)-3-(furan-2-yl)propane-1,3-dione **13a.**

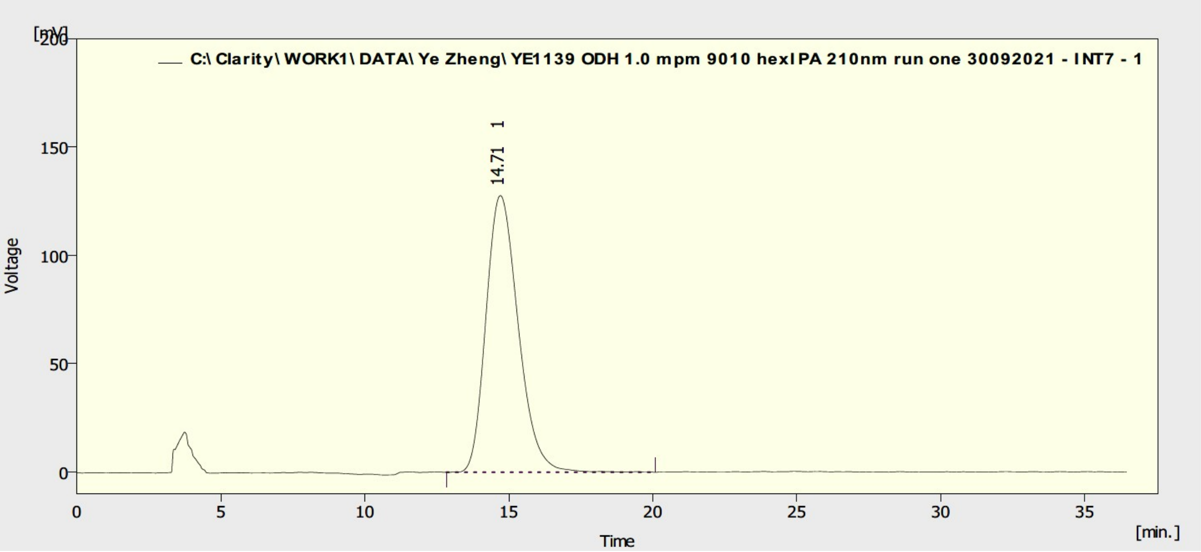

Result Table (Uncal - C:\Clarity\WORK1\DATA\Ye Zheng\YE1139 ODH 1.0 mpm 9010 hexIPA 210nm run one 30092021 - INT7 - 1)

	Reten. Time [min]	Area [mV.s]	Height [mV]	Area [%]	Height [%]	W05 [min]	Compound Name
1	14.713	9973.626	127.739	100.0	100.0	1.20	
	Total	9973.626	127.739	100.0	100.0		

1-(2,6-Dimethoxyphenyl)-3-(furan-2-yl)-3-hydroxypropan-1-one 13b.

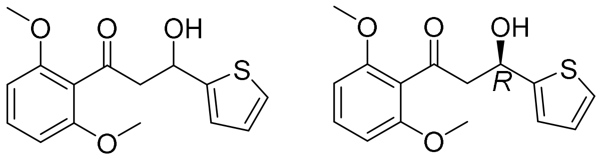

This compound is novel.

Synthesis of a racemic standard: (*R,R*)-3C-Tethered Ru(II)-TsDPEN catalyst (0.90 mg, 1.5 mmol, 0.5 mol%) and (*S,S*)-3C-tethered Ru(II)-TsDPEN catalyst (0.90 mg, 1.5 mmol, 0.5 mol%) were added to FA: TEA (5:2 azeotropic mixture, 0.36 mL) at rt and the mixture was stirred under a nitrogen atmosphere for 15 minutes; after which a solution of 1-(2,6-dimethoxyphenyl)-3-(furan-2-yl)propane-1,3-dione **13a** (80.0 mg, 0.292 mmol) in DCM (0.50 mL) was added. The reaction mixture was stirred under a nitrogen atmosphere and followed by TLC (4:1 hexane: EtOAc). After 24 h, the reaction was quenched using saturated $NaHCO_3$ solution (20 mL). EtOAc (20 mL) was added and the organic layer was separated. The aqueous layer was extracted with EtOAc (3 x 20 mL) and the combined organic layers were washed with saturated $NaHCO_3$ solution (2 × 20 mL), and brine (20 mL), dried ($MgSO_4$) and filtered. The solvent was removed to give the crude product. The product was isolated via flash chromatography on silica eluted with 0-50% EtOAc in hexane to give 1-(2,6-dimethoxyphenyl)-3-(furan-2-yl)-3-hydroxypropan-1-one **13b** as a colorless oil (63.5 mg, 0.230 mmol, 79%). TLC: Rf ca 0.20 (4:1 hexane: EtOAc), strong UV and $KMnO_4$; HRMS found (ESI+) *m/z*: [M+H]+ Calcd for $C_{15}H_{16}NaO_5$ 299.0884; Found 299.0890; 2.1 ppm error; ν_{max} 3431 (br), 2942, 2839, 1696, 1591, 1471, 1432, 1287, 1251, 1105, 1062, 1074, 1008, 984, 779, 729 cm^{-1}; ^1H NMR (500 MHz, $CDCl_3$): δ 7.36 (1H, d, *J* = 0.9, H of furan), 7.28 (1H, t, *J* = 8.4, ArH), 6.57 (2H, d, *J* = 8.4, ArH), 6.33-6.29 (2H, m, H of furan), 5.31 (1H, dt, *J* = 7.8, 3.8, ArCH), 3.79 (6H, s, OCH_3), 3.50 (1H, dt, *J* = 7.8, 3.8, OH), 3.37-3.28 (2H, m, CH_2); $^{13}C\{^1H\}$ NMR (125 MHz, $CDCl_3$): δ 204.6 (C), 156.8 (C), 155.2 (C), 142.0 (CH), 131.3 (CH), 119.4 (C), 110.2 (CH), 106.2 (CH), 104.0 (CH), 67.0 (CH), 55.9 (CH_3), 49.6 (CH_2); *m/z* (ES-API+) 299.1 (M^+ + 23, 100%).

Enantiomeric excess and conversion determined by HPLC analysis (Chiralcel ODH, 30 cm x 6 mm column, hexane:iPrOH 90:10, 1.0 mL/min, T = 25°C) ketone 14.7 min, *R* and *S* isomers 29.8 min and 34.1 min, configuration assigned by analogy.

(*R*)-1-(2,6-Dimethoxyphenyl)-3-(furan-2-yl)-3-hydroxypropan-1-one **13b.**

(*R,R*)-3C-tethered Ru(II)-TsDPEN catalyst (1.8 mg, 2.9 mmol, 1 mol%) was added to FA: TEA (5:2 azeotropic mixture, 0.36 mL) at rt and the mixture was stirred under a nitrogen atmosphere for 15 minutes; after which a solution of 1-(2,6-dimethoxyphenyl)-3-(furan-2-yl)propane-1,3-dione **13a** (80.0 mg, 0.292 mmol) in DCM (0.50 mL) was added. The reaction mixture was stirred under a nitrogen atmosphere and followed by TLC (4:1 hexane: EtOAc). After 24 h, the reaction was quenched using saturated $NaHCO_3$ solution (20 mL). EtOAc (20 mL) was added and the organic layer was separated. The aqueous layer was extracted with EtOAc (3 x 20 mL) and the combined organic layers were dried ($MgSO_4$) and filtered. The solvent was removed to give the crude product. The product was isolated via flash chromatography on silica eluted with 0-50% EtOAc in hexane to give (*R*)-1-(2,6-dimethoxyphenyl)-3-(furan-2-yl)-3-hydroxypropan-1-one **13b** as a colorless oil (65.5 mg, 0.237 mmol, 81%). The reaction was also followed by HPLC (Chiralcel ODH, 30 cm x 6 mm column, hexane:iPrOH 90:10, 1.0 mL/min, T = 25°C): 100% conversion; $[\alpha]_D^{24}$ +10.0 (c 0.444 in $CHCl_3$) 99% ee (*R*).

^{1}H NMR (500 MHz, $CDCl_3$) of 1-(2,6-dimethoxyphenyl)-3-(furan-2-yl)-3-hydroxypropan-1-one **13b**.

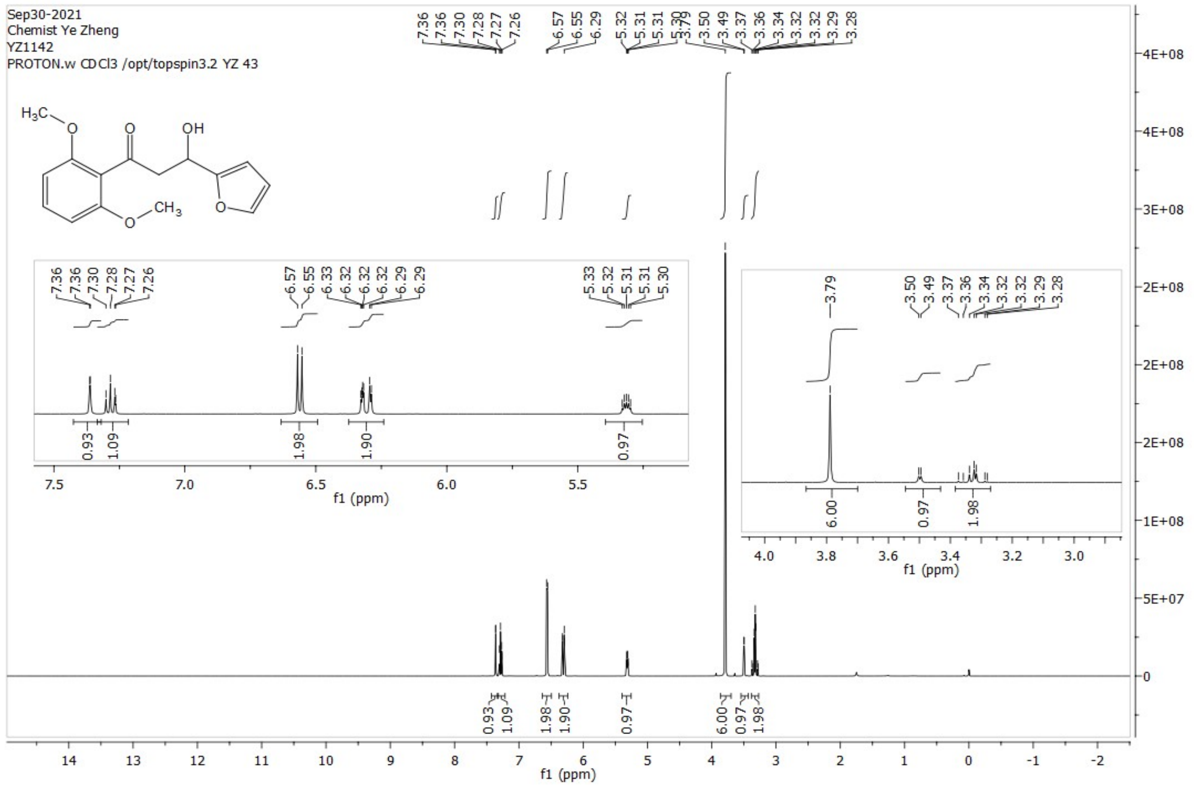

$^{13}C\{^{1}H\}$ NMR (125 MHz, $CDCl_3$) of 1-(2,6-dimethoxyphenyl)-3-(furan-2-yl)-3-hydroxypropan-1-one **13b**.

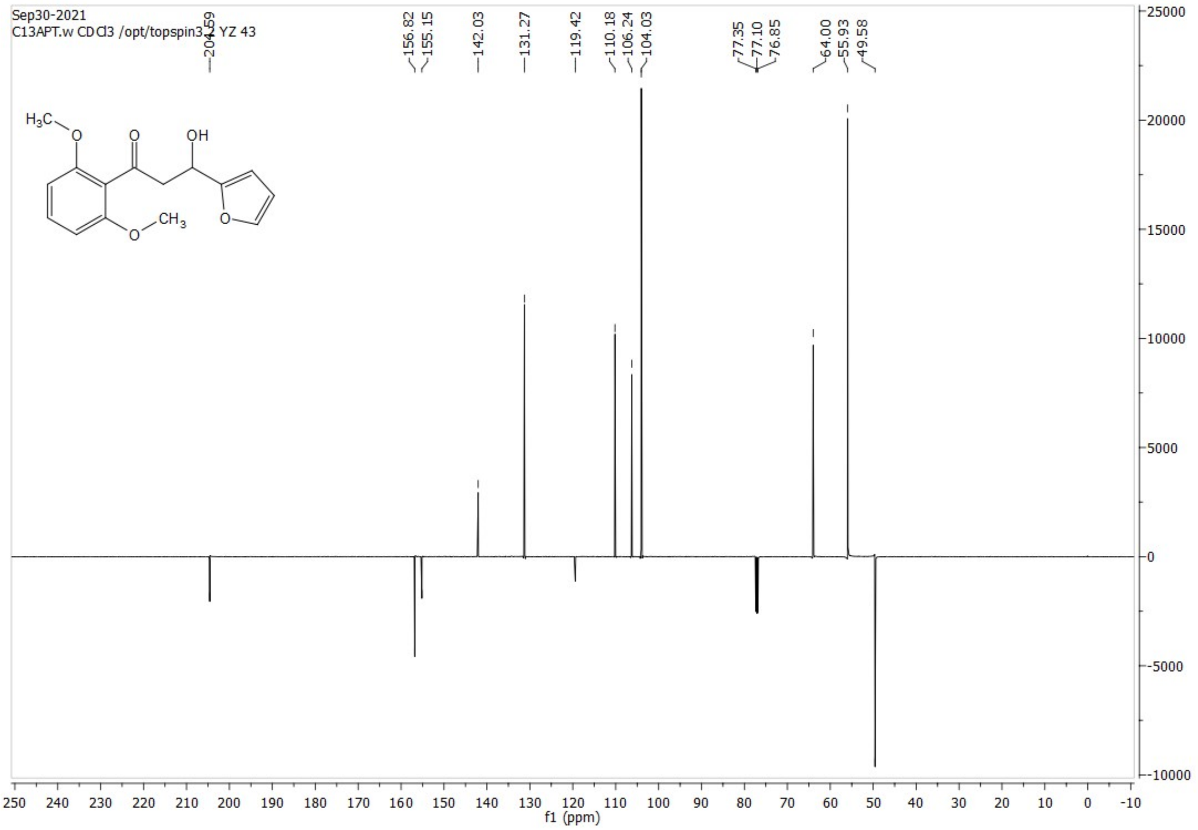

COSY (500 MHz, $CDCl_3$) of 1-(2,6-dimethoxyphenyl)-3-(furan-2-yl)-3-hydroxypropan-1-one **13b**.

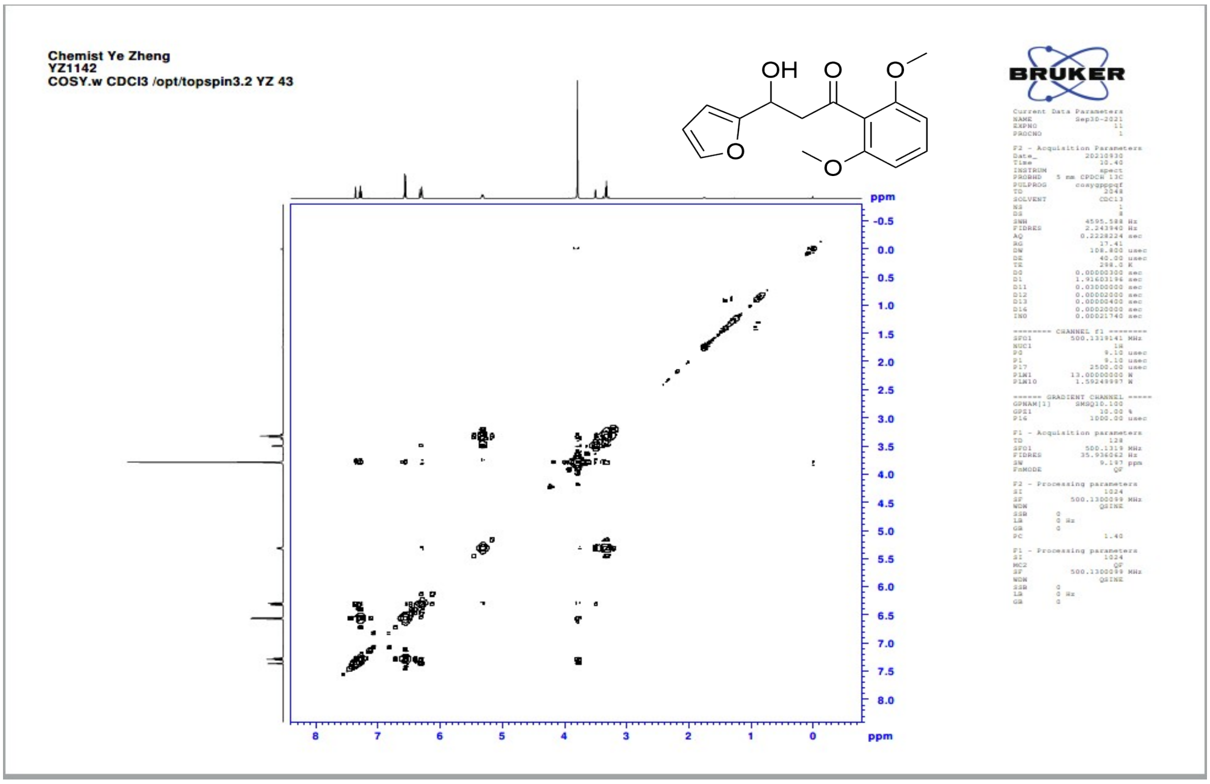

HSQC (500 MHz, $CDCl_3$) of 1-(2,6-dimethoxyphenyl)-3-(furan-2-yl)-3-hydroxypropan-1-one **13b**.

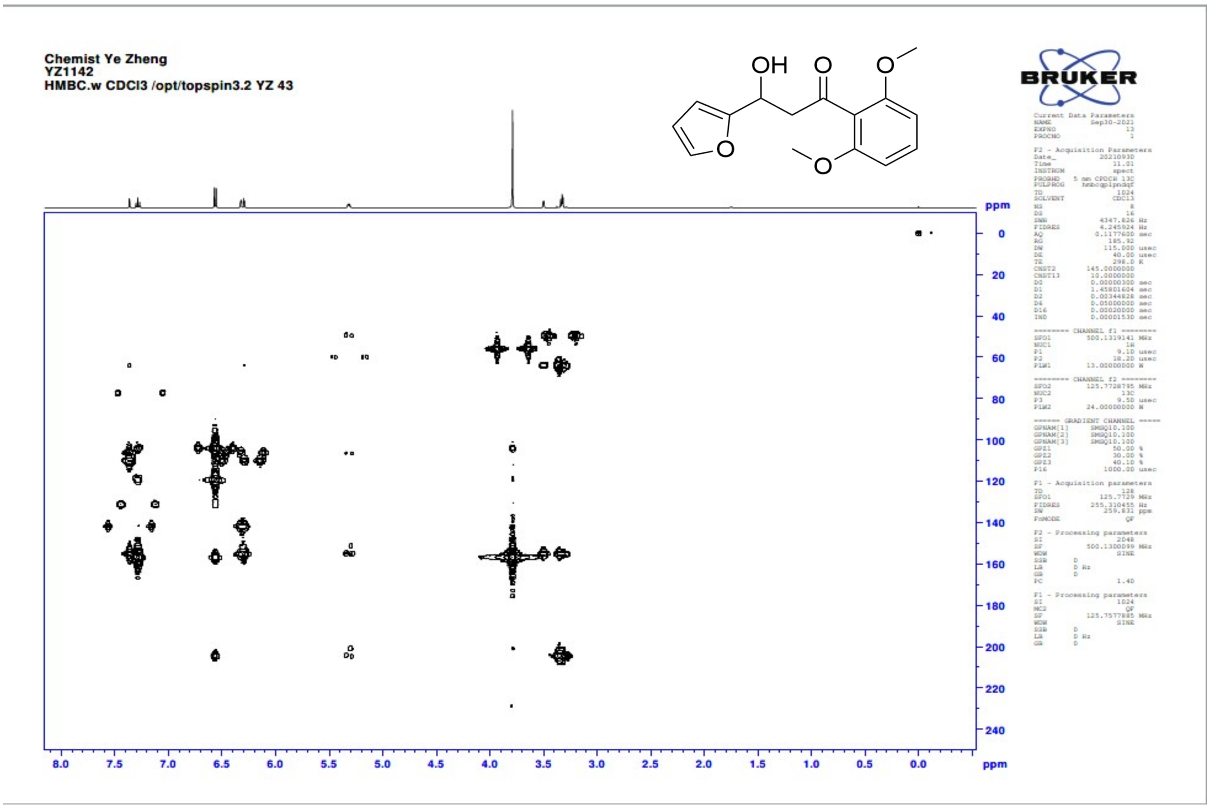

HMBC (500 MHz, $CDCl_3$) of 1-(2,6-dimethoxyphenyl)-3-(furan-2-yl)-3-hydroxypropan-1-one **13b**.

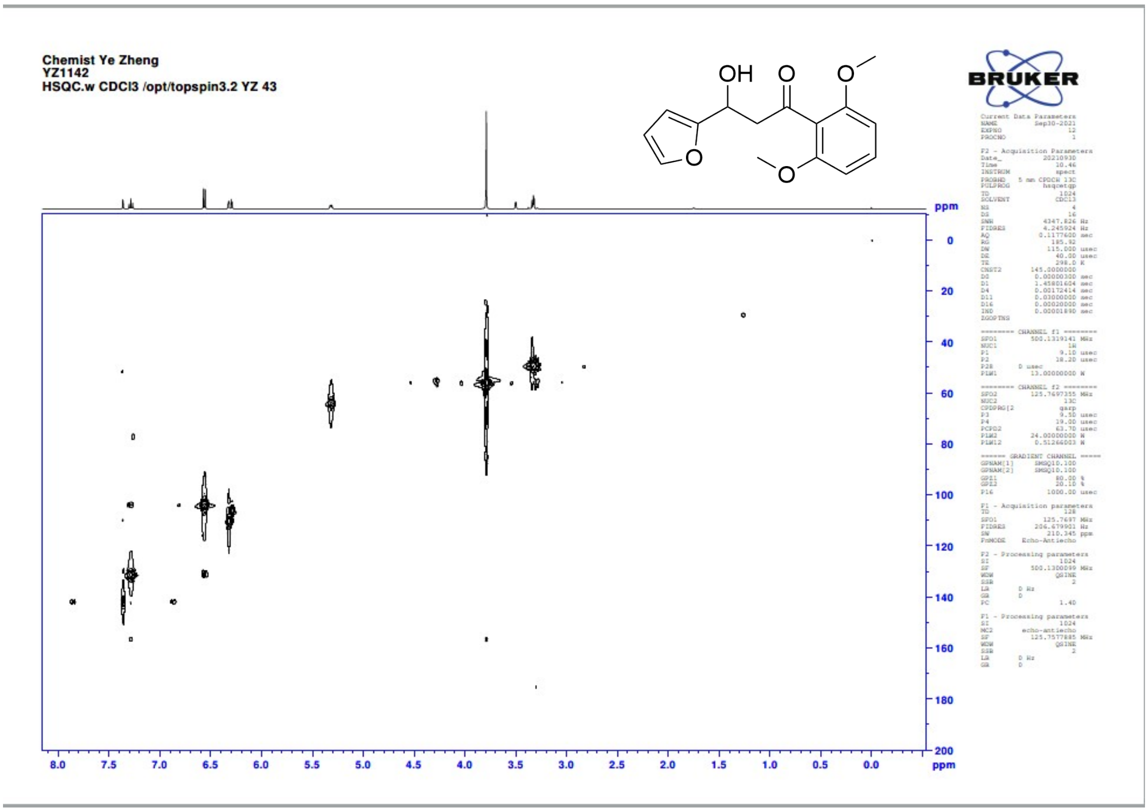

HPLC of racemic 1-(2,6-dimethoxyphenyl)-3-(furan-2-yl)-3-hydroxypropan-1-one **13b.**

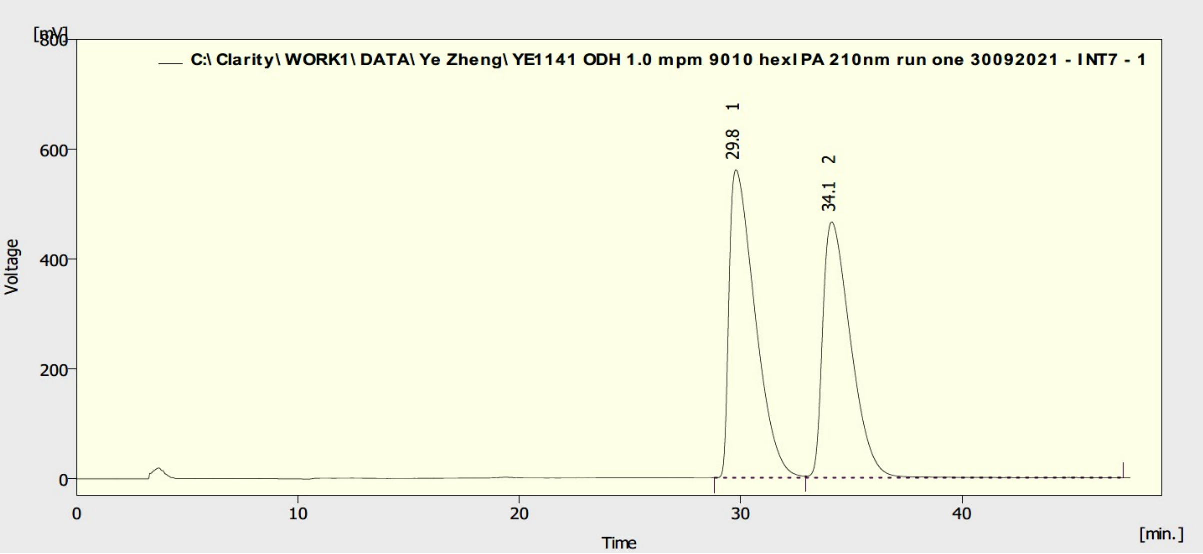

Result Table (Uncal - C:\Clarity\WORK1\DATA\Ye Zheng\YE1141 ODH 1.0 mpm 9010 hexIPA 210nm run one 30092021 - INT7 - 1)

	Reten. Time [min]	Area [mV.s]	Height [mV]	Area [%]	Height [%]	W05 [min]	Compound Name
1	29.783	45608.727	560.440	52.7	54.6	1.27	
2	34.113	40981.689	465.428	47.3	45.4	1.36	
	Total	86590.416	1025.869	100.0	100.0		

HPLC of (*R*)-1-(2,6-dimethoxyphenyl)-3-(furan-2-yl)-3-hydroxypropan-1-one **13b**. (*R,R*)-3C-Tethered Ru(II)-TsDPEN catalyst (after 24 h, 100% conversion, 99% ee (*R*)).

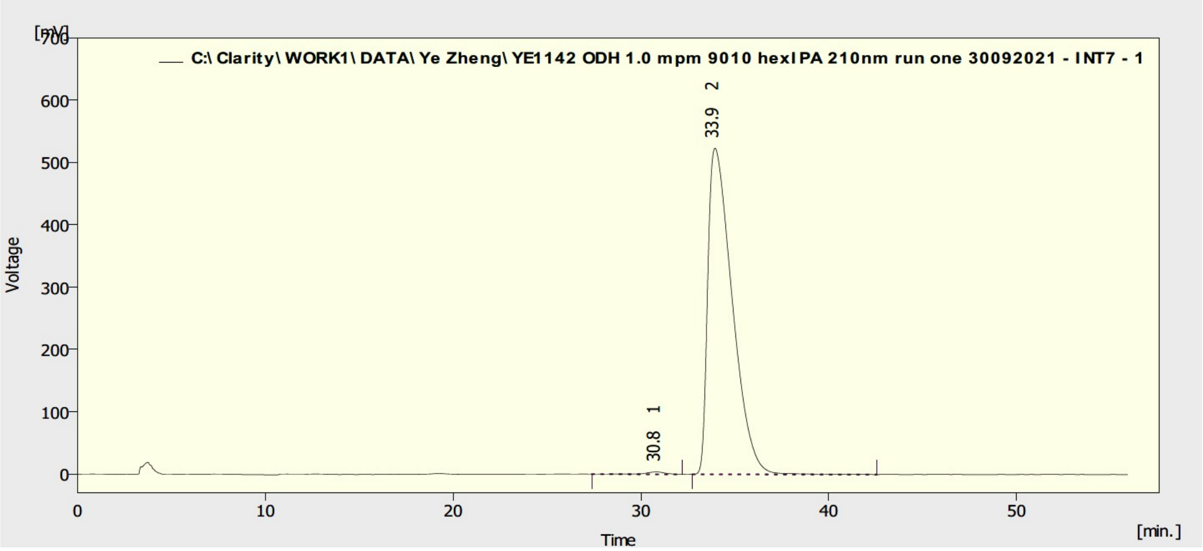

Result Table (Uncal - C:\Clarity\WORK1\DATA\Ye Zheng\YE1142 ODH 1.0 mpm 9010 hexIPA 210nm run one 30092021 - INT7 - 1)

	Reten. Time [min]	Area [mV.s]	Height [mV]	Area [%]	Height [%]	W05 [min]	Compound Name
1	30.827	300.144	3.952	0.6	0.7	1.04	
2	33.943	47300.209	523.438	99.4	99.3	1.41	
	Total	47600.353	527.390	100.0	100.0		

Procedures for 3-(aryl)-3-hydroxy-1-(2,3,4,5,6-pentamethylphenyl)propan-1-ones 14b-17b.

1-(2-Chlorophenyl)-3-(2,3,4,5,6-pentamethylphenyl)propane-1,3-dione 14a.

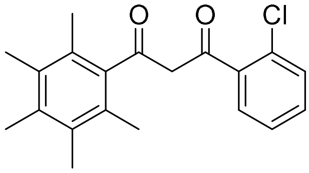

This compound is novel.

To a solution of sodium hydride (400 mg, 60% dispersion in mineral oil, 10.0 mmol) in THF (2 mL) at 0 °C was added dropwise a solution of 1-(2,3,4,5,6-pentamethylphenyl)ethan-1-one **6** (380 mg, 2.00 mmol) in THF (3 mL). The reaction mixture was stirred under a nitrogen atmosphere at 0 °C for 30 min and then stirred under a nitrogen atmosphere at rt for 30 min, after which a solution of ethyl 2-chlorobenzoate (925 mg, 5.00 mmol) in THF (3 mL) was added dropwise. The reaction mixture was heated to 65 °C and left stirring under the nitrogen atmosphere overnight. The reaction was followed by TLC (9:1 hexane: EtOAc). The mixture was quenched by 2M HCl solution (20 mL). EtOAc (20 mL) was added and the organic layer was separated. The aqueous layer was extracted with EtOAc (3 × 20 mL), and the combined organic layers were washed with saturated $NaHCO_3$ solution (2 × 20 mL) and brine (20 mL), dried ($MgSO_4$) and filtered. Solvent was removed to give the crude product. The product was isolated via flash chromatography on silica eluted with 0-10% EtOAc in hexane to give 1-(2-chlorophenyl)-3-(2,3,4,5,6-pentamethylphenyl)propane-1,3-dione **14a** as a yellow solid (398 mg, 1.21 mmol, 61%). TLC: Rf ca 0.80 (9:1 hexane: EtOAc), strong UV and $KMnO_4$; Mp: 100°C; HRMS (ESI+) *m/z*: [M+H]+ Calcd for $C_{20}H_{22}{}^{35}ClO_2$ 329.1304; Found 329.1303; -0.4 ppm error; ν_{max} 2916, 1579, 1510, 1500, 1272, 1254, 1013, 953, 843, 772, 742 cm^{-1}; enol: keto = 100:0; ^1H NMR (400 MHz, $CDCl_3$): δ 15.97 (1H, s, OH of enol form), 7.70 (1H, dd, J = 7.1, 2.3, ArH), 7.47-7.32 (3H, m, ArH), 6.23 (1H, s, CH of enol form), 2.28 (6H, s, CH_3) 2.27 (3H, s, CH_3) 2.23 (6H, s, CH_3); $^{13}C\{^1H\}$ NMR (100 MHz, $CDCl_3$): δ 191.5 (C), 186.8 (C), 136.3 (C), 136.1 (C), 135.4 (C), 132.9 (C), 131.9 (C), 131.8 (CH), 130.79 (CH), 130.2 (CH), 129.7 (C), 127.0 (CH), 104.8 (CH of enol form), 17.6 (CH_3), 16.9 (CH_3), 16.2 (CH_3); *m/z* (ES-API+) 351.1 (M^+ + 23, 100%).

Enantiomeric excess and conversion determined by HPLC analysis (Chiralpak IC, 30 cm x 6 mm column, hexane:iPrOH 95:5, 0.5 mL/min, T = 25°C) ketone 14.0 min, *R* and *S* isomers 18.5 min and 21.1 min, configuration assigned by analogy.

^{1}H NMR (400 MHz, $CDCl_3$) of 1-(2-chlorophenyl)-3-(2,3,4,5,6-pentamethylphenyl)propane-1,3-dione **14a**.

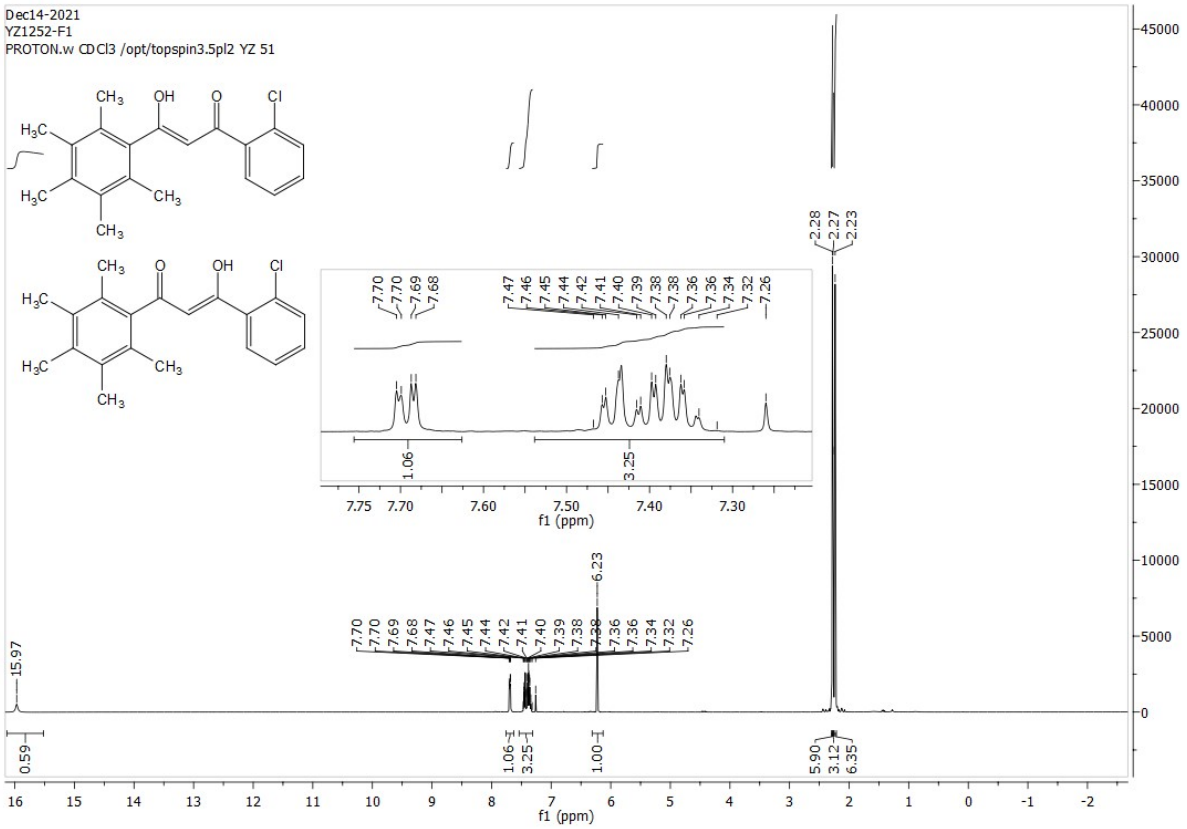

$^{13}C\{^{1}H\}$ NMR (100 MHz, $CDCl_3$) of 1-(2-chlorophenyl)-3-(2,3,4,5,6-pentamethylphenyl)propane-1,3-dione **14a**.

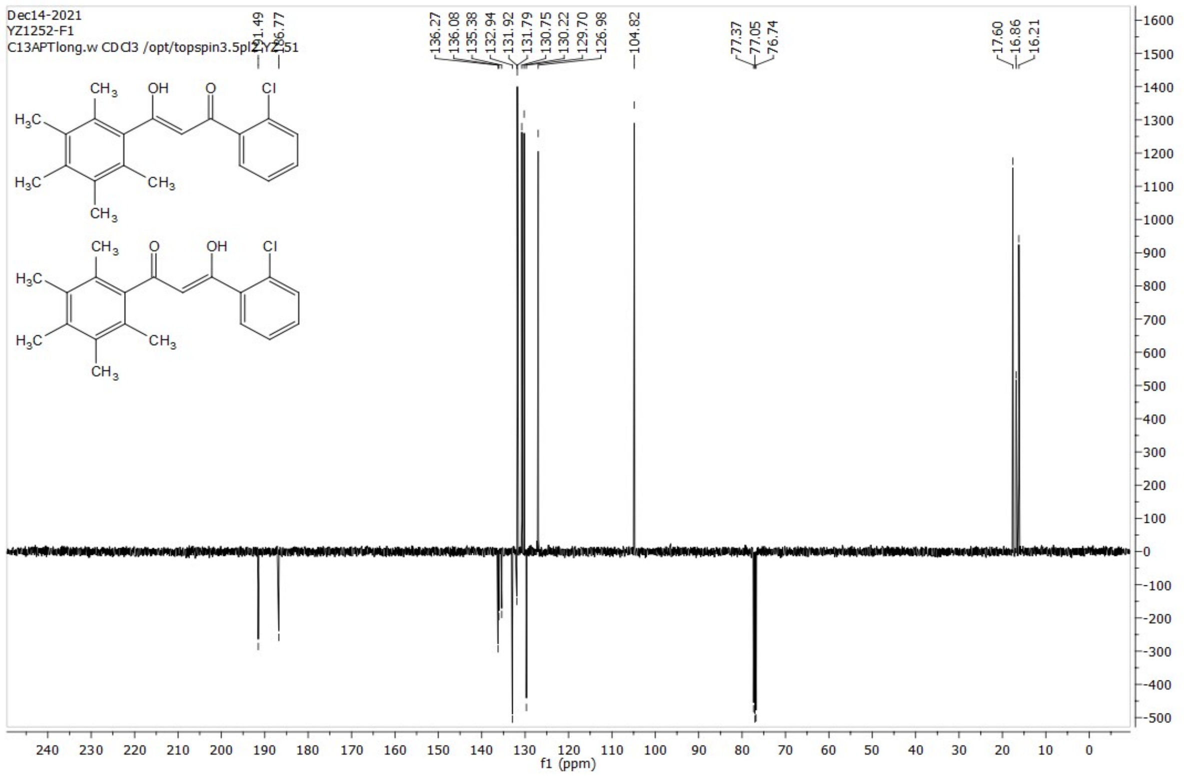

COSY (400 MHz, $CDCl_3$) of 1-(2-chlorophenyl)-3-(2,3,4,5,6-pentamethylphenyl)propane-1,3-dione **14a**.

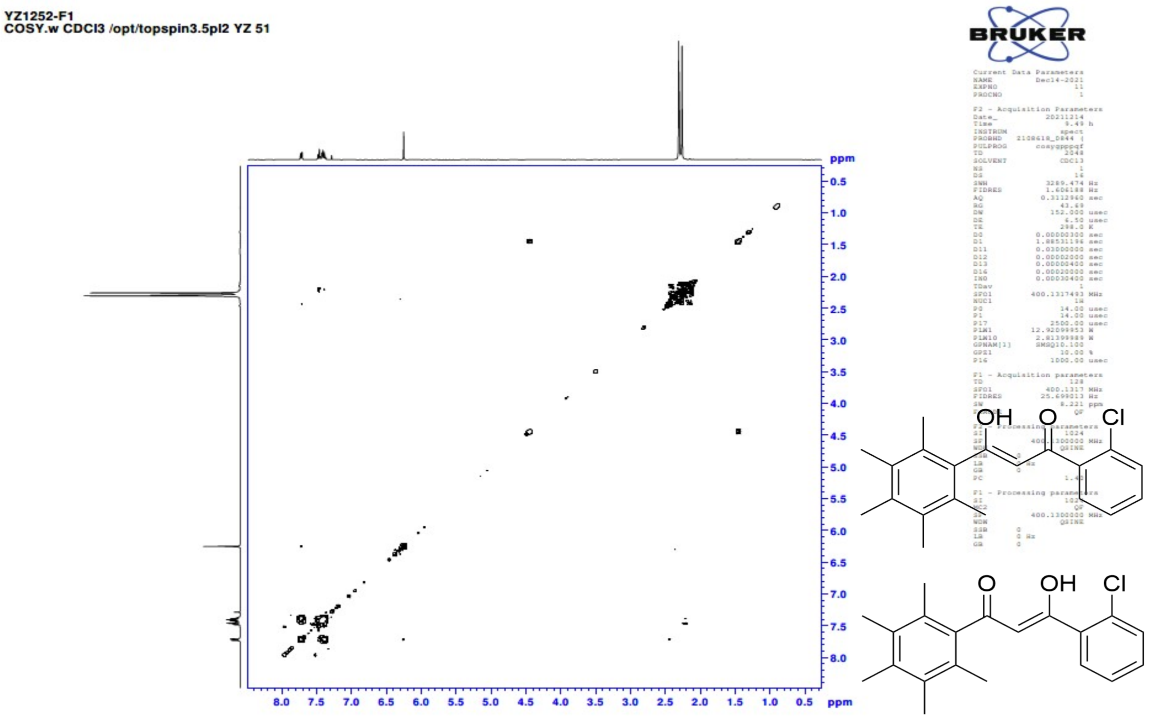

HSQC (400 MHz, $CDCl_3$) of 1-(2-chlorophenyl)-3-(2,3,4,5,6-pentamethylphenyl)propane-1,3-dione **14a**.

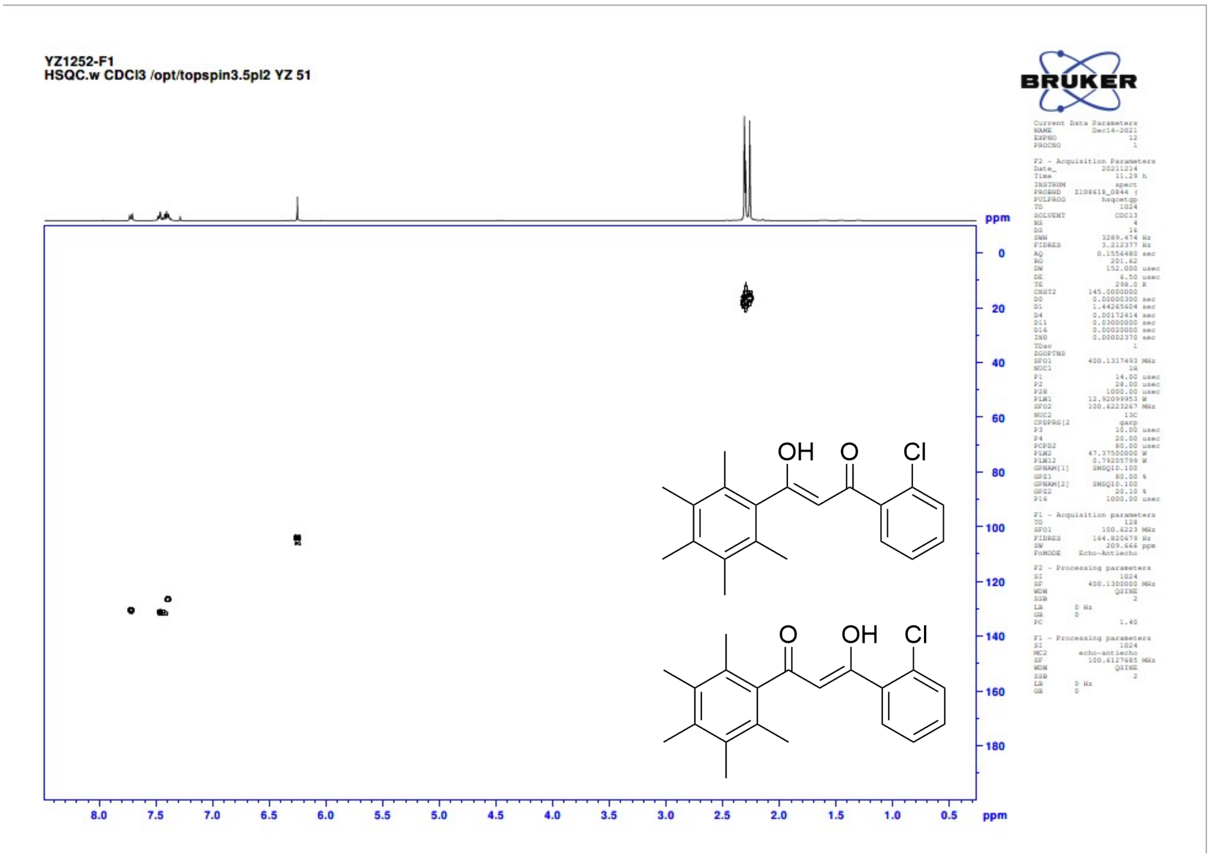

HMBC (400 MHz, $CDCl_3$) of 1-(2-chlorophenyl)-3-(2,3,4,5,6-pentamethylphenyl)propane-1,3-dione **14a**.

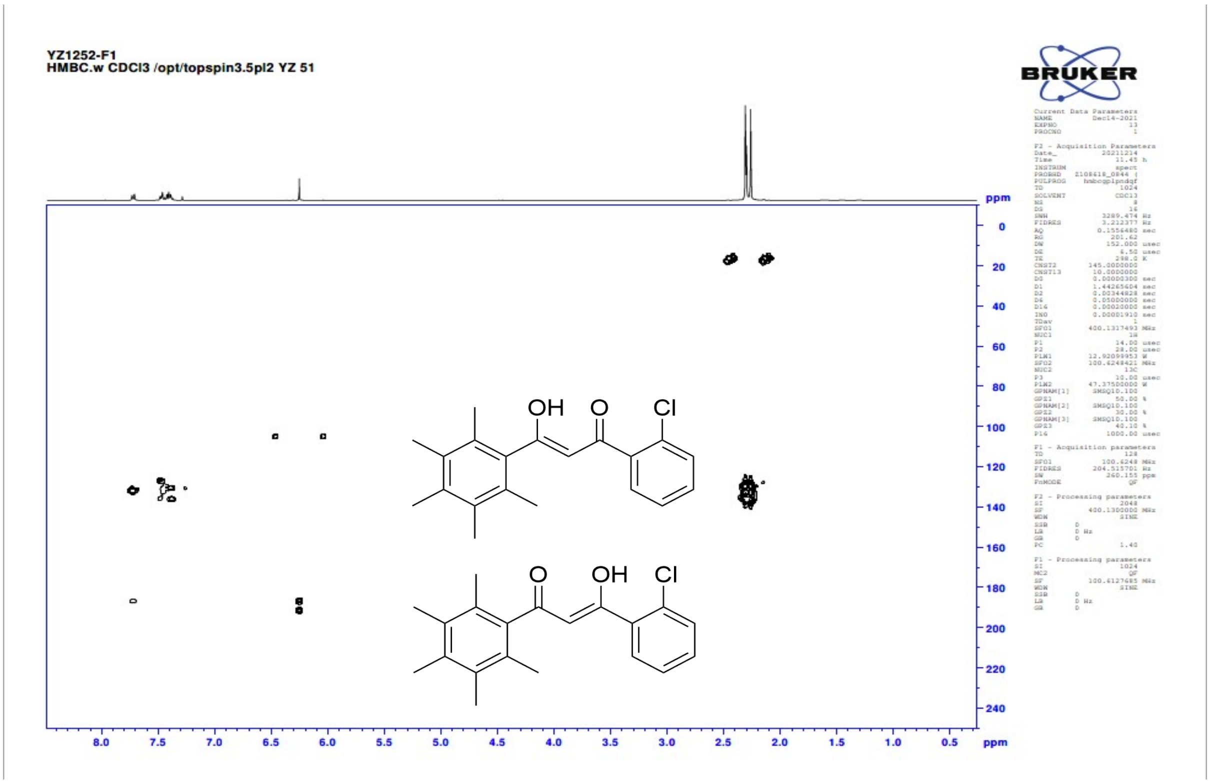

HPLC of 1-(2-chlorophenyl)-3-(2,3,4,5,6-pentamethylphenyl)propane-1,3-dione **14a.**

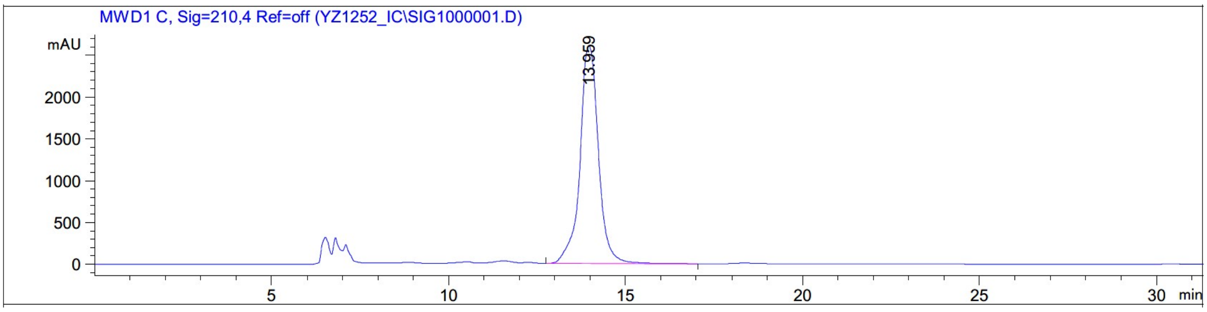

Signal 2: MWD1 C, Sig=210,4 Ref=off

Peak #	RetTime [min]	Type	Width [min]	Area [mAU*s]	Height [mAU]	Area %
1	13.959	BB	0.5495	9.44891e4	2603.00293	100.0000

Totals : 9.44891e4 2603.00293

3-(2-Chlorophenyl)-3-hydroxy-1-(2,3,4,5,6-pentamethylphenyl)propan-1-one 14b.

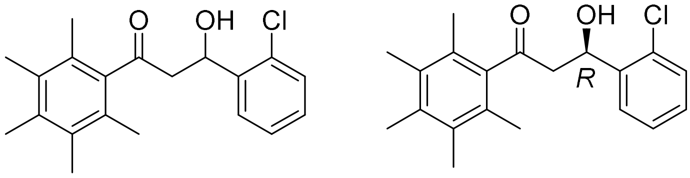

This compound is novel.

Synthesis of a racemic standard: (*R,R*)-3C-Tethered Ru(II)-TsDPEN catalyst (0.76 mg, 1.2 mmol, 0.5 mol%) and (*S,S*)-3C-tethered Ru(II)-TsDPEN catalyst (0.76 mg, 1.2 mmol, 0.5 mol%) were added to FA: TEA (5:2 azeotropic mixture, 0.36 mL) at rt and the mixture was stirred under a nitrogen atmosphere for 15 minutes; after which a solution of 1-(2-chlorophenyl)-3-(2,3,4,5,6-pentamethylphenyl)propane-1,3-dione **14a** (80.0 mg, 0.244 mmol) in DCM (0.50 mL) was added. The reaction mixture was stirred under a nitrogen atmosphere and followed by TLC (9:1 hexane: EtOAc). After 24 h, the reaction was quenched using saturated $NaHCO_3$ solution (20 mL). EtOAc (20 mL) was added and the organic layer was separated. The aqueous layer was extracted with EtOAc (3 x 20 mL) and the combined organic layers were dried ($MgSO_4$) and filtered. The solvent was removed to give the crude product. The product was isolated via flash chromatography on silica eluted with 0-10% EtOAc in hexane to give 3-(2-chlorophenyl)-3-hydroxy-1-(2,3,4,5,6-pentamethylphenyl)propan-1-one **14b** as a white solid (57.0 mg, 0.173 mmol, 71%). TLC: Rf ca 0.20 (9:1 hexane: EtOAc), strong UV and $KMnO_4$; Mp: 131°C; HRMS (ESI+) *m/z*: [M+H]+, Calcd for $C_{20}H_{23}{}^{35}ClNaO_2$ 353.1274; Found 353.1279; 1.4 ppm error; ν_{max} 3372 (br), 3060, 2979, 2873, 1699, 1444, 1381, 1345, 1213, 1108, 1064, 1050, 1033, 1010, 933, 752, 743, 713 cm^{-1}; ^1H NMR (400 MHz, $CDCl_3$): δ 7.71 (1H, d, *J* = 7.4, ArH), 7.35-7.29 (2H, m, ArH), 7.20 (1H, t, *J* = 8.3, ArH), 5.74 (1H, d, *J* = 9.7, ArCH), 3.92 (1H, d, *J* = 3.3, OH), 3.30 (1H, dd, *J* = 18.9, 1.7, CH_2), 2.88 (1H, dd, *J* = 18.9, 9.5, CH_2), 2.23 (3H, s, CH_3), 2.18 (6H, s, CH_3), 2.16 (6H, s, CH_3); $^{13}C\{^1H\}$ NMR (100 MHz, $CDCl_3$): δ 212.9 (C), 140.1 (C), 139.6 (C), 135.9 (C), 133.2 (C), 131.1 (C), 129.3 (CH), 128.5 (CH), 127.3 (C), 127.2 (CH), 127.1 (CH), 66.6 (CH), 52.1 (CH_2), 17.1 (CH_3), 16.7 (CH_3), 15.9 (CH_3); *m/z* (ES-API+) 353.1 (M^+ + 23, 100%).

Enantiomeric excess and conversion determined by HPLC analysis (Chiralpak IC, 30 cm x 6 mm column, hexane:iPrOH 95:5, 0.5 mL/min, T = 25°C) ketone 14.0 min, *R* and *S* isomers 18.5 min and 21.1 min, configuration assigned by analogy.

(*R*)-3-(2-Chlorophenyl)-3-hydroxy-1-(2,3,4,5,6-pentamethylphenyl)propan-1-one **14b**.

(*R,R*)-3C-tethered Ru(II)-TsDPEN catalyst (1.5 mg, 2.4 mmol, 1 mol%) was added to FA: TEA (5:2 azeotropic mixture, 0.36 mL) at rt and the mixture was stirred under a nitrogen atmosphere for 15 minutes; after which a solution of 1-(2-chlorophenyl)-3-(2,3,4,5,6-pentamethylphenyl)propane-1,3-dione **14a** (80.0 mg, 0.244 mmol) in DCM (0.50 mL) was added. The reaction mixture was stirred under a nitrogen atmosphere and followed by TLC (9:1 hexane: EtOAc). After 24 h, the reaction was quenched using saturated $NaHCO_3$ solution (20 mL). EtOAc (20 mL) was added and the organic layer was separated. The aqueous layer was extracted with EtOAc (3 x 20 mL) and the combined organic layers were dried ($MgSO_4$) and filtered. The solvent was removed to give the crude product. The product was isolated via flash chromatography on silica eluted with 0-10% EtOAc in hexane to give (*R*)-3-(2-chlorophenyl)-3-hydroxy-1-(2,3,4,5,6-pentamethylphenyl)propan-1-one **14b** as a white solid (65.0 mg, 0.197 mmol, 81%). The reaction was also followed by HPLC (Chiralpak IC, 30 cm x 6 mm column, hexane:iPrOH 95:5, 0.5 mL/min, T = 25°C): 100% conversion; $[\alpha]_D^{20}$ +79.2 (c 0.440 in $CHCl_3$) 93% ee (*R*).

^{1}H NMR (400 MHz, $CDCl_3$) of (2-chlorophenyl)-3-hydroxy-1-(2,3,4,5,6-pentamethylphenyl)propan-1-one **14b**.

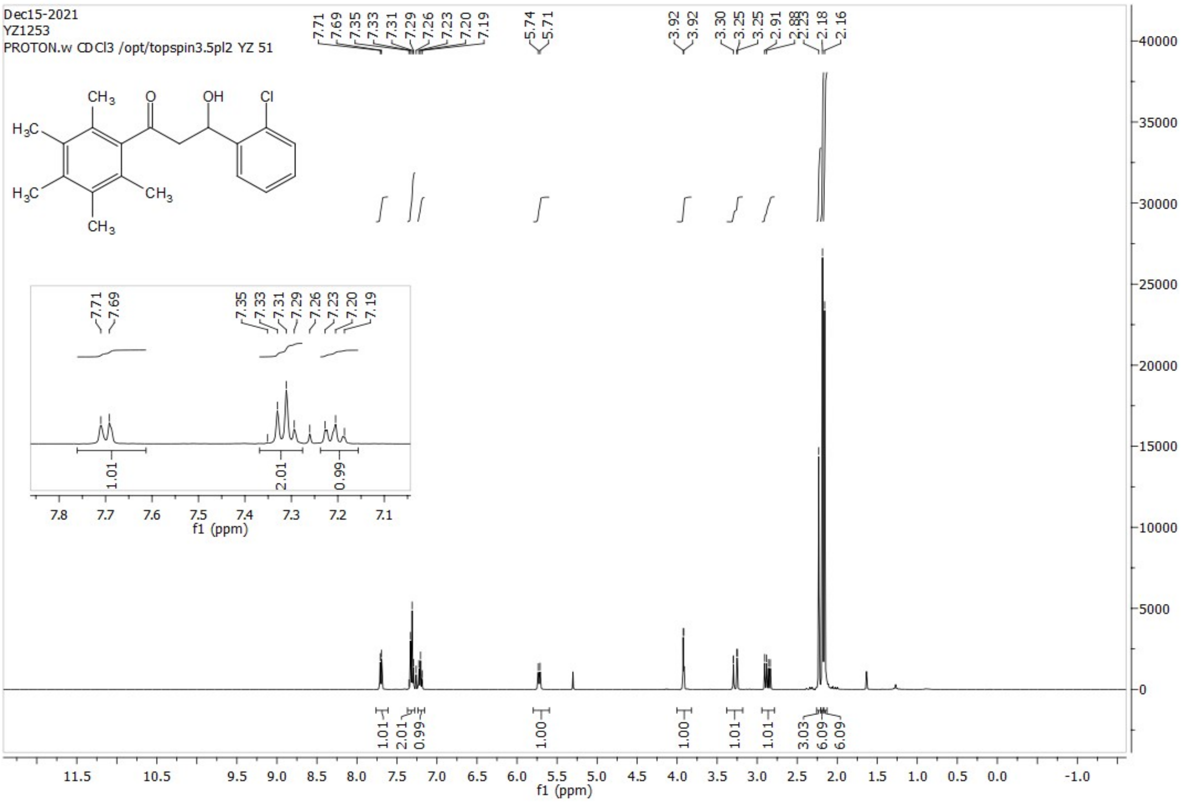

$^{13}C\{^{1}H\}$ NMR (100 MHz, $CDCl_3$) of (2-chlorophenyl)-3-hydroxy-1-(2,3,4,5,6-pentamethylphenyl)propan-1-one **14b**.

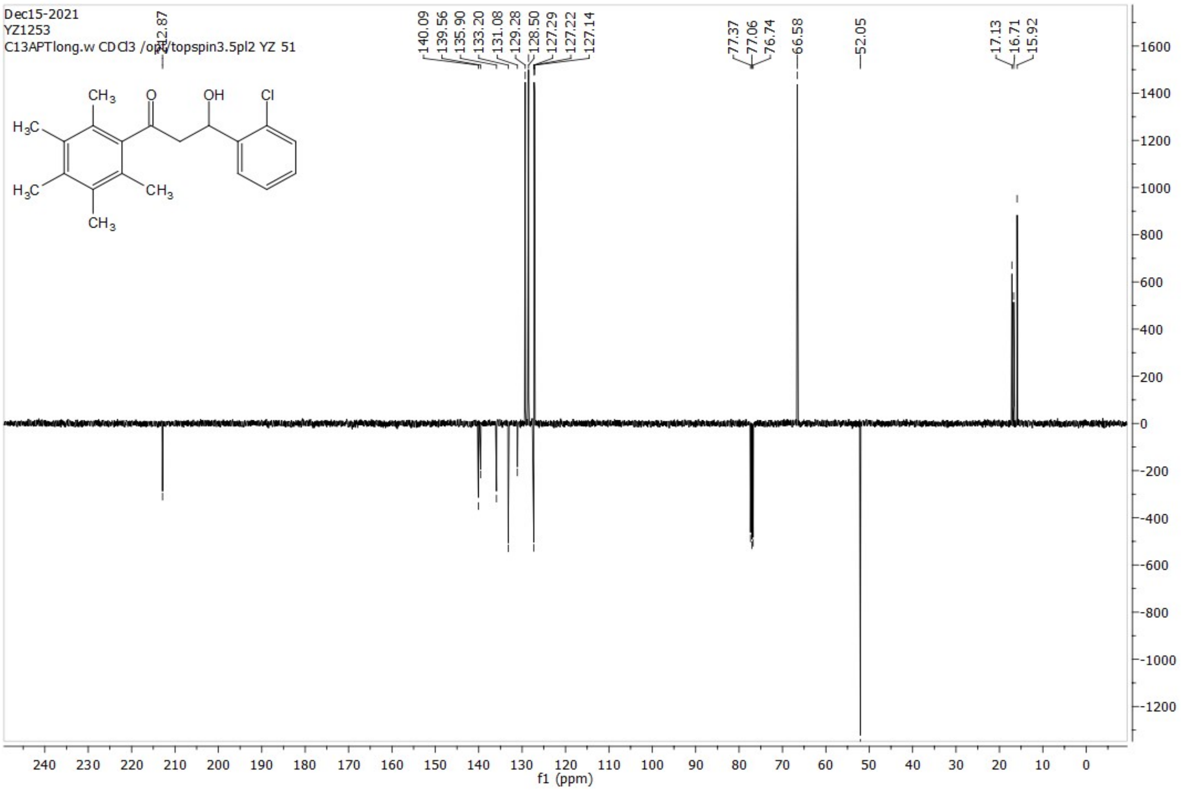

COSY (400 MHz, $CDCl_3$) of (2-chlorophenyl)-3-hydroxy-1-(2,3,4,5,6-pentamethylphenyl)propan-1-one **14b**.

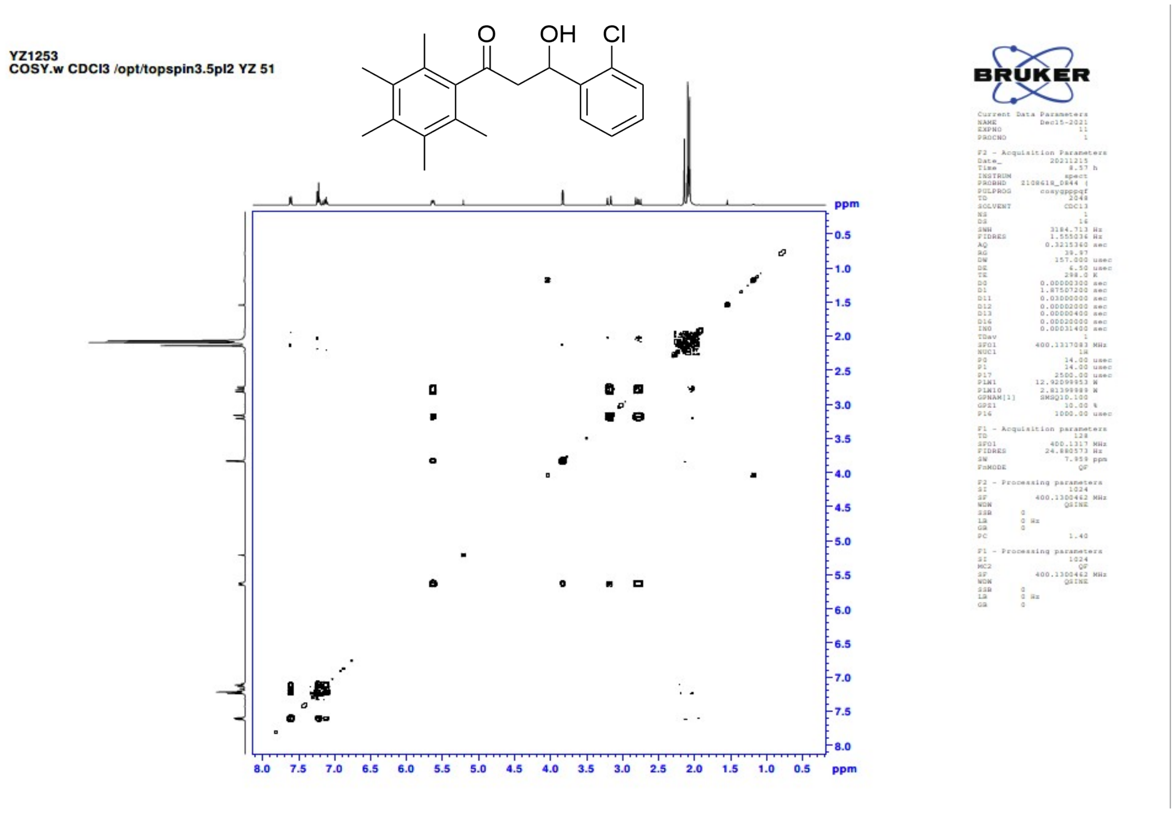

HSQC (400 MHz, $CDCl_3$) of (2-chlorophenyl)-3-hydroxy-1-(2,3,4,5,6-pentamethylphenyl)propan-1-one **14b**.

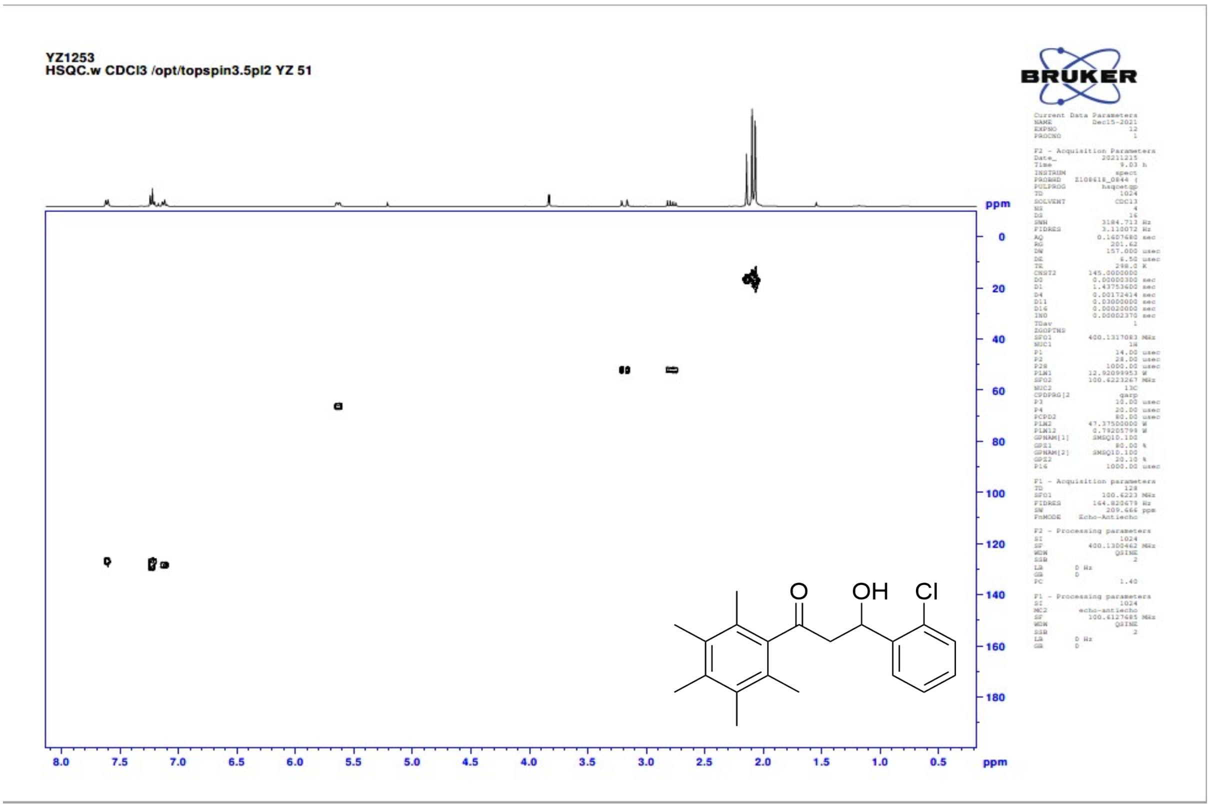

HMBC (400 MHz, $CDCl_3$) of (2-chlorophenyl)-3-hydroxy-1-(2,3,4,5,6-pentamethylphenyl)propan-1-one **14b**.

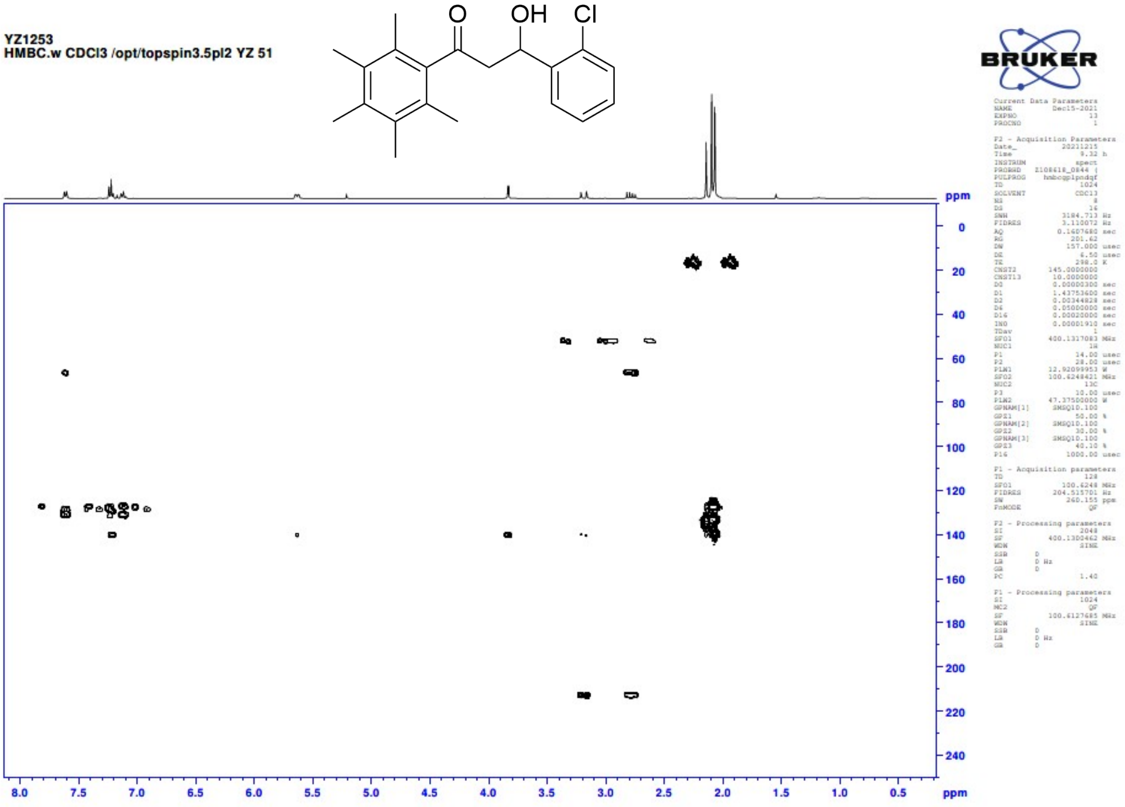

HPLC of racemic 3-(2-chlorophenyl)-3-hydroxy-1-(2,3,4,5,6-pentamethylphenyl)propan-1-one **14b.**

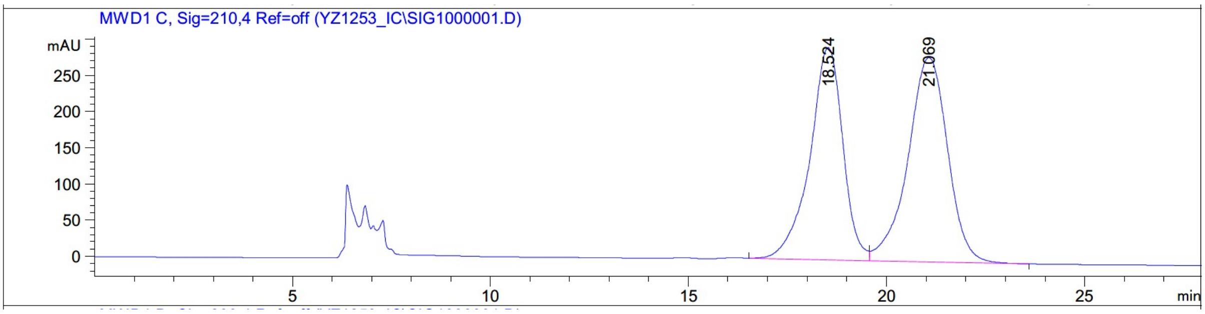

Signal 2: MWD1 C, Sig=210,4 Ref=off

Peak #	RetTime [min]	Type	Width [min]	Area [mAU*s]	Height [mAU]	Area %
1	18.524	BV	0.8775	1.70207e4	293.08728	46.3590
2	21.069	VB	1.0703	1.96942e4	282.16711	53.6410

Totals :				3.67149e4	575.25439	

HPLC of (*R*)-(2-chlorophenyl)-3-hydroxy-1-(2,3,4,5,6-pentamethylphenyl)propan-1-one **14b**.

(*R,R*)-3C-Tethered Ru(II)-TsDPEN catalyst (after 24 h, 100% conversion, 93% ee, (*R*)).

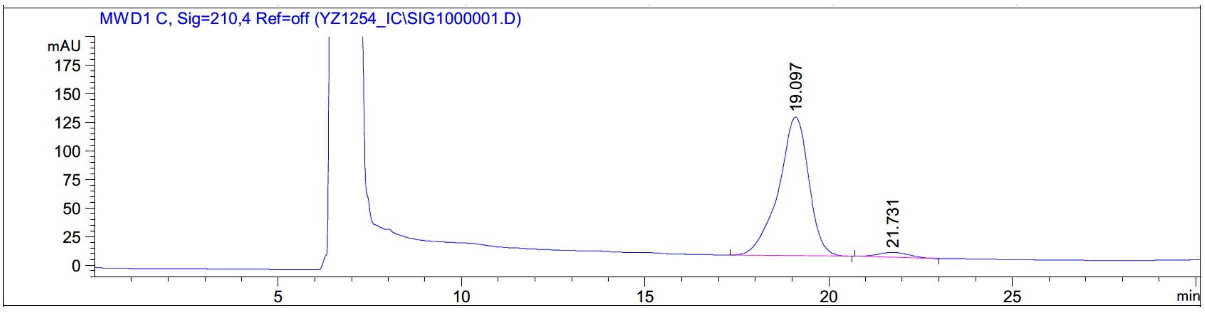

Signal 2: MWD1 C, Sig=210,4 Ref=off

Peak #	RetTime [min]	Type	Width [min]	Area [mAU*s]	Height [mAU]	Area %
1	19.097	BB	0.8462	6933.04102	121.41792	96.5422
2	21.731	BB	0.7079	248.31772	4.18705	3.4578

Totals : 7181.35873 125.60497

1-(4-Chlorophenyl)-3-(2,3,4,5,6-pentamethylphenyl)propane-1,3-dione 15a.

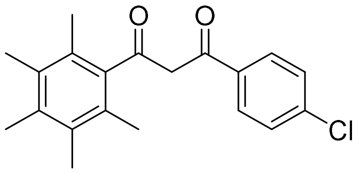

This compound is novel.

To a solution of sodium hydride (300 mg, 60% dispersion in mineral oil, 7.50 mmol) in THF (2 mL) at 0 °C was added dropwise a solution of 1-(2,3,4,5,6-pentamethylphenyl)ethan-1-one **6** (285 mg, 1.50 mmol) in THF (2 mL). The reaction mixture was stirred under a nitrogen atmosphere at 0 °C for 30 min and then stirred under a nitrogen atmosphere at rt for 30 min, after which a solution of ethyl 4-chlorobenzoate (694 mg, 3.75 mmol) in THF (2 mL) was added dropwise. The reaction mixture was heated to 65 °C and left stirring under the nitrogen atmosphere overnight. The reaction was followed by TLC (9:1 hexane: EtOAc). The mixture was quenched by 2M HCl solution (20 mL). EtOAc (20 mL) was added and the organic layer was separated. The aqueous layer was extracted with EtOAc (3 × 20 mL), and the combined organic layers were washed with saturated $NaHCO_3$ solution (2 × 20 mL) and brine (20 mL), dried ($MgSO_4$) and filtered. Solvent was removed to give the crude product. The product was isolated via flash chromatography on silica eluted with 0-10% EtOAc in hexane to give 1-(4-chlorophenyl)-3-(2,3,4,5,6-pentamethylphenyl)propane-1,3-dione **15a** as a white solid (176 mg, 0.537 mmol, 36%). TLC: Rf ca 0.80 (9:1 hexane: EtOAc), strong UV and $KMnO_4$; Mp: 149°C; HRMS (ESI+) *m/z*: [M+H]+ Calcd for $C_{20}H_{22}{}^{35}ClO_2$ 329.1300; Found 329.1303; 0.7 ppm error; ν_{max} 2922, 1589, 1518, 1446, 1276, 1241, 1090, 1012, 965, 838, 780 cm^{-1}; enol: keto = 100:0; ^{1}H NMR (400 MHz, $CDCl_3$): δ 7.88 (2H, d, *J* = 8.6, ArH), 7.45 (2H, t, *J* = 8.6, ArH), 6.29 (1H, s, CH of enol form), 2.28 (3H, s, CH_3), 2.26 (6H, s, CH_3), 2.24 (6H, s, CH_3); $^{13}C\{^1H\}$ NMR (100 MHz, $CDCl_3$): δ 193.2 (C), 183.9 (C), 138.8 (C), 136.2 (C), 135.9 (C), 133.8 (C), 133.0 (C), 129.5 (C), 129.0 (CH), 128.6 (CH), 99.4 (CH of enol form), 17.6 (CH_3), 16.9 (CH_3), 16.2 (CH_3); *m/z* (ES-API+) 351.1 (M^+ + 23, 100%).

Enantiomeric excess and conversion determined by HPLC analysis (Chiralpak IC, 30 cm x 6 mm column, hexane:iPrOH 90:10, 1.0 mL/min, T = 25°C) ketone 5.2 min, *R* isomer 8.8 min and *S* isomer 9.3 min.

^{1}H NMR (400 MHz, $CDCl_3$) of 1-(4-chlorophenyl)-3-(2,3,4,5,6-pentamethylphenyl)propane-1,3-dione **15a**.

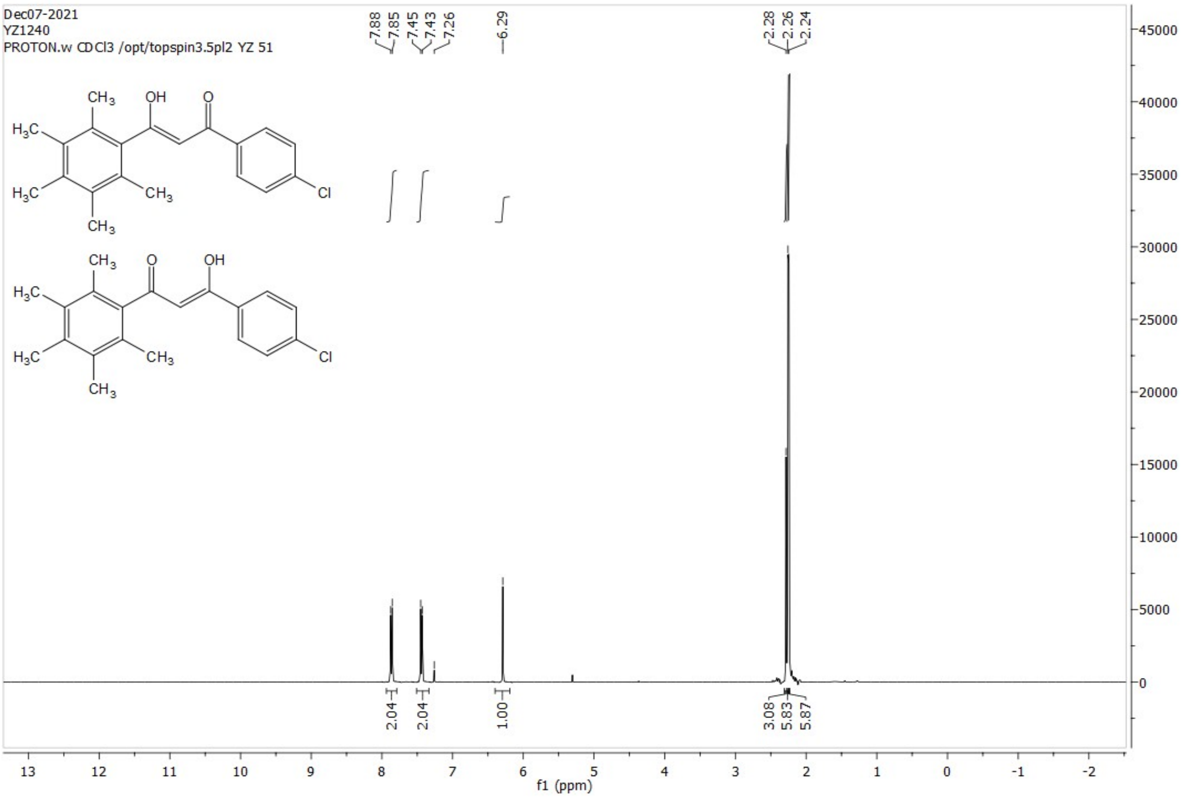

$^{13}C\{^{1}H\}$ NMR (100 MHz, $CDCl_3$) of 1-(4-chlorophenyl)-3-(2,3,4,5,6-pentamethylphenyl)propane-1,3-dione **15a**.

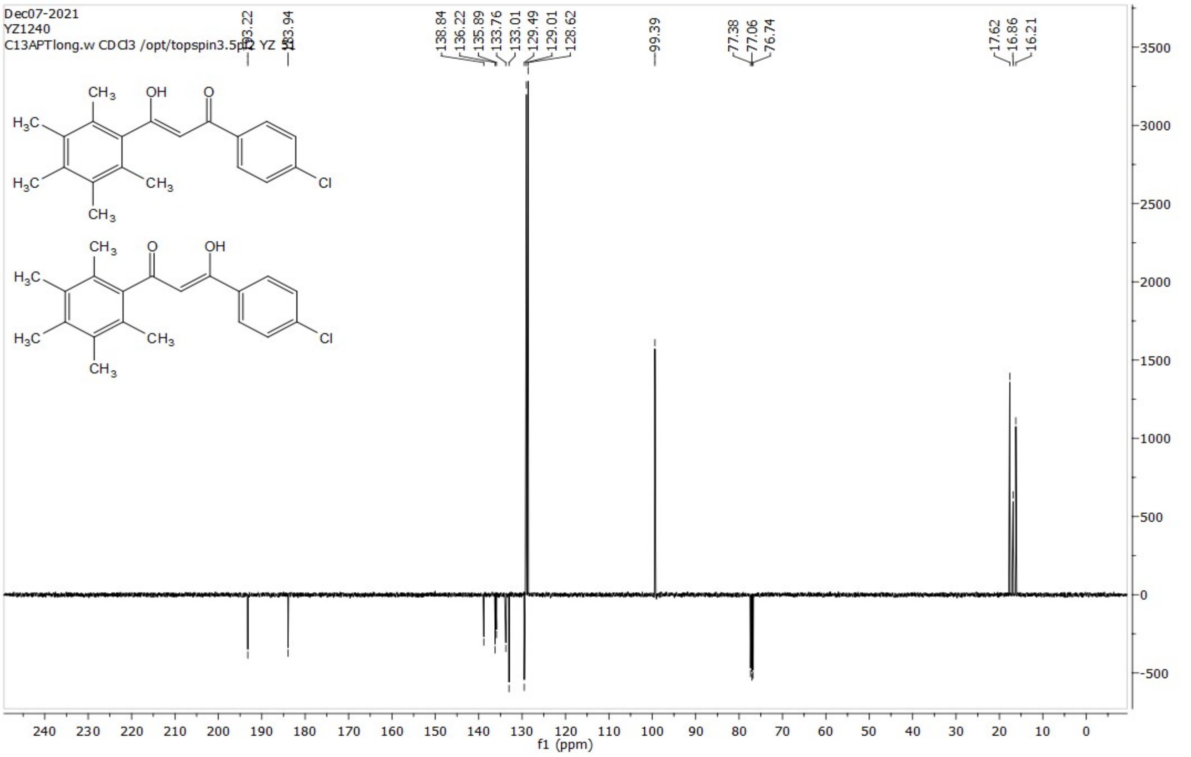

COSY (400 MHz, $CDCl_3$) of 1-(4-chlorophenyl)-3-(2,3,4,5,6-pentamethylphenyl)propane-1,3-dione **15a**.

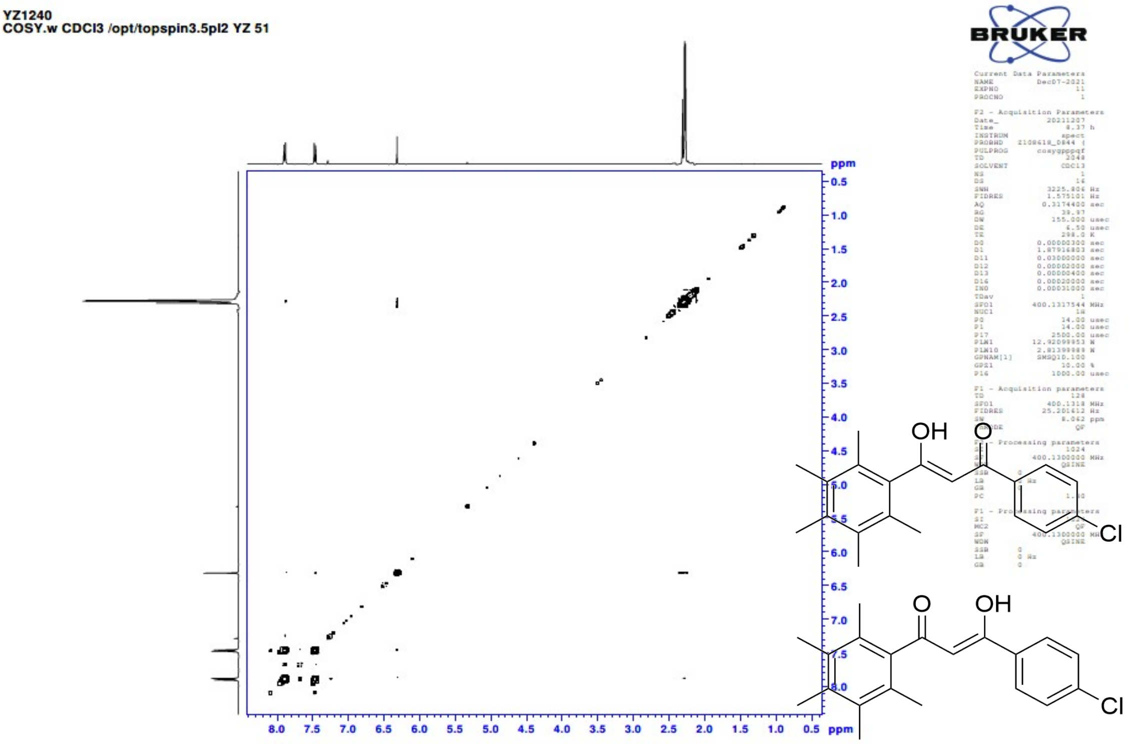

HSQC (400 MHz, $CDCl_3$) of 1-(4-chlorophenyl)-3-(2,3,4,5,6-pentamethylphenyl)propane-1,3-dione **15a**.

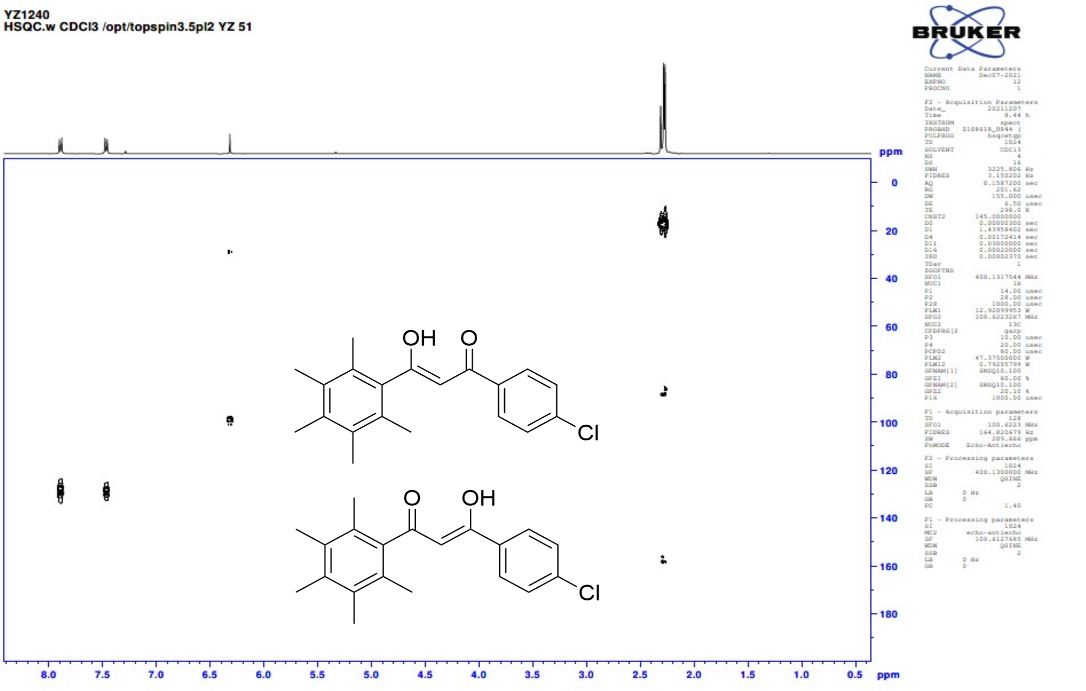

HMBC (400 MHz, $CDCl_3$) of 1-(4-chlorophenyl)-3-(2,3,4,5,6-pentamethylphenyl)propane-1,3-dione **15a**.

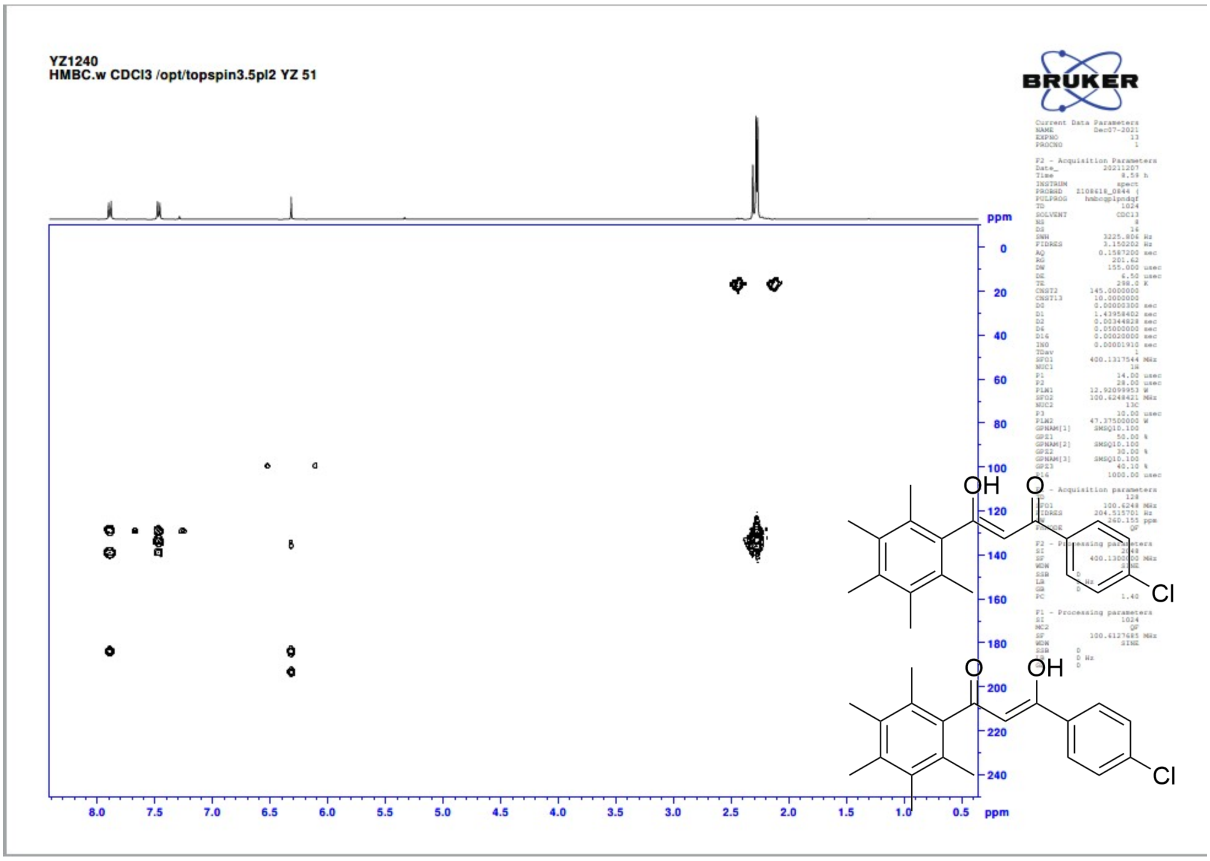

HPLC of 1-(4-chlorophenyl)-3-(2,3,4,5,6-pentamethylphenyl)propane-1,3-dione **15a.**

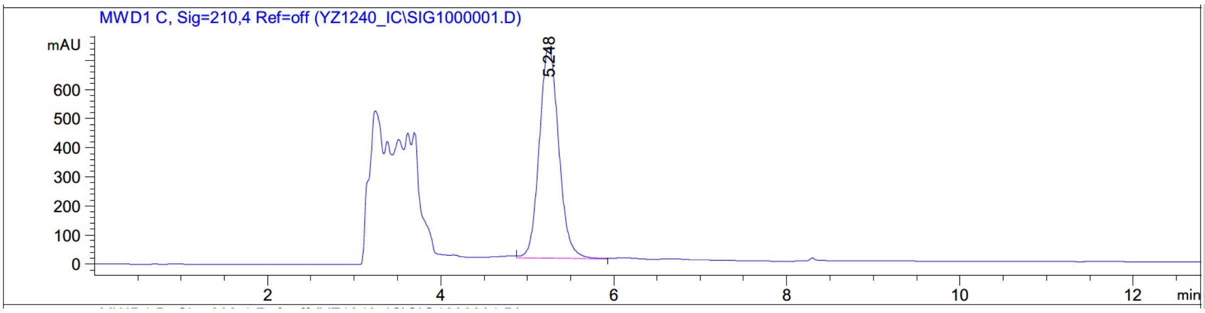

Signal 2: MWD1 C, Sig=210,4 Ref=off

Peak #	RetTime [min]	Type	Width [min]	Area [mAU*s]	Height [mAU]	Area %
1	5.248	VV	0.2435	1.13198e4	727.63397	100.0000
Totals :				1.13198e4	727.63397	

3-(4-Chlorophenyl)-3-hydroxy-1-(2,3,4,5,6-pentamethylphenyl)propan-1-one 15b.

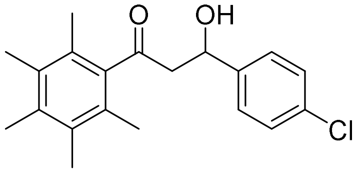

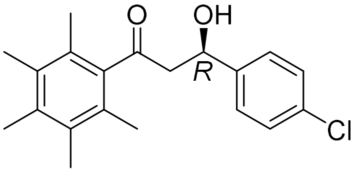

This compound is novel.

Synthesis of a racemic standard: (*R,R*)-3C-Tethered Ru(II)-TsDPEN catalyst (0.76 mg, 1.2 mmol, 0.5 mol%) and (*S,S*)-3C-tethered Ru(II)-TsDPEN catalyst (0.76 mg, 1.2 mmol, 0.5 mol%) were added to FA: TEA (5:2 azeotropic mixture, 0.36 mL) at rt and the mixture was stirred under a nitrogen atmosphere for 15 minutes; after which a solution of 1-(4-chlorophenyl)-3-(2,3,4,5,6-pentamethylphenyl)propane-1,3-dione **15a** (80.0 mg, 0.244 mmol) in DCM (0.50 mL) was added. The reaction mixture was stirred under a nitrogen atmosphere and followed by TLC (9:1 hexane: EtOAc). After 24 h, the reaction was quenched using saturated $NaHCO_3$ solution (20 mL). EtOAc (20 mL) was added and the organic layer was separated. The aqueous layer was extracted with EtOAc (3 x 20 mL) and the combined organic layers were dried ($MgSO_4$) and filtered. The solvent was removed to give the crude product. The product was isolated via flash chromatography on silica eluted with 0-10% EtOAc in hexane to give 3-(4-chlorophenyl)-3-hydroxy-1-(2,3,4,5,6-pentamethylphenyl)propan-1-one **15b** as a white solid (58.0 mg, 0.176 mmol, 72%). TLC: Rf ca 0.20 (9:1 hexane: EtOAc), strong UV and $KMnO_4$; Mp: 139°C; HRMS (ESI+) *m/z*: [M+H]+ Calcd for $C_{20}H_{23}{}^{35}ClNaO_2$ 353.1272; Found 353.1279; 2.0 ppm error; ν_{max} 3440 (br), 2918, 2873, 1691, 1485, 1392, 1356, 1317, 1085, 1010, 814, 781 cm^{-1}; ^1H NMR (400 MHz, $CDCl_3$): δ 7.36-7.30 (4H, m, ArH), 5.34 (1H, dt, *J* = 7.5, 3.9, ArCH), 3.66 (1H, d, *J* = 3.2, OH), 3.07-3.01 (2H, m, CH_2), 2.23 (3H, s, CH_3), 2.18 (6H, s, CH_3), 2.12 (6H, s, CH_3); $^{13}C\{^1H\}$ NMR (100 MHz, $CDCl_3$): δ 212.3 (C), 141.8 (C), 139.5 (C), 136.0 (C), 133.3 (C), 133.3 (C), 128.7 (CH), 127.23 (C), 127.2 (CH), 69.1 (CH), 53.8 (CH_2), 17.2 (CH_3), 16.7 (CH_3), 15.9 (CH_3); *m/z* (ES-API+) 353.1 (M^+ + 23, 100%).

Enantiomeric excess and conversion determined by HPLC analysis (Chiralpak IC, 30 cm x 6 mm column, hexane:iPrOH 90:10, 1.0 mL/min, T = 25°C) ketone 5.2 min, *R* isomer 8.8 min and *S* isomer 9.3 min.

(*R*)-3-(4-Chlorophenyl)-3-hydroxy-1-(2,3,4,5,6-pentamethylphenyl)propan-1-one **15b**.

(*R,R*)-3C-tethered Ru(II)-TsDPEN catalyst (1.5 mg, 2.4 mmol, 1 mol%) was added to FA: TEA (5:2 azeotropic mixture, 0.36 mL) at rt and the mixture was stirred under a nitrogen atmosphere for 15 minutes; after which a solution of 1-(4-chlorophenyl)-3-(2,3,4,5,6-pentamethylphenyl)propane-1,3-dione **15a** (80.0 mg, 0.244 mmol) in DCM (0.50 mL) was added. The reaction mixture was stirred under a nitrogen atmosphere and followed by TLC (9:1 hexane: EtOAc). After 24 h, the reaction was quenched using saturated $NaHCO_3$ solution (20 mL). EtOAc (20 mL) was added and the organic layer was separated. The aqueous layer was extracted with EtOAc (3 x 20 mL) and the combined organic layers were dried ($MgSO_4$) and filtered. The solvent was removed to give the crude product. The product was isolated via flash chromatography on silica eluted with 0-10% EtOAc in hexane to give (*R*)-3-(4-chlorophenyl)-3-hydroxy-1-(2,3,4,5,6-pentamethylphenyl)propan-1-one **15b** as a white solid (65.0 mg, 0.197 mmol, 81%). The reaction was also followed by HPLC (Chiralpak IC, 30 cm x 6 mm column, hexane:iPrOH 90:10, 1.0 mL/min, T = 25°C): 100% conversion; $[\alpha]_D^{20}$ +32.9 (c 0.260 in $CHCl_3$) 98% ee (*R*).

^{1}H NMR (400 MHz, $CDCl_3$) of (*R*)-3-(4-chlorophenyl)-3-hydroxy-1-(2,3,4,5,6-pentamethylphenyl)propan-1-one **15b**.

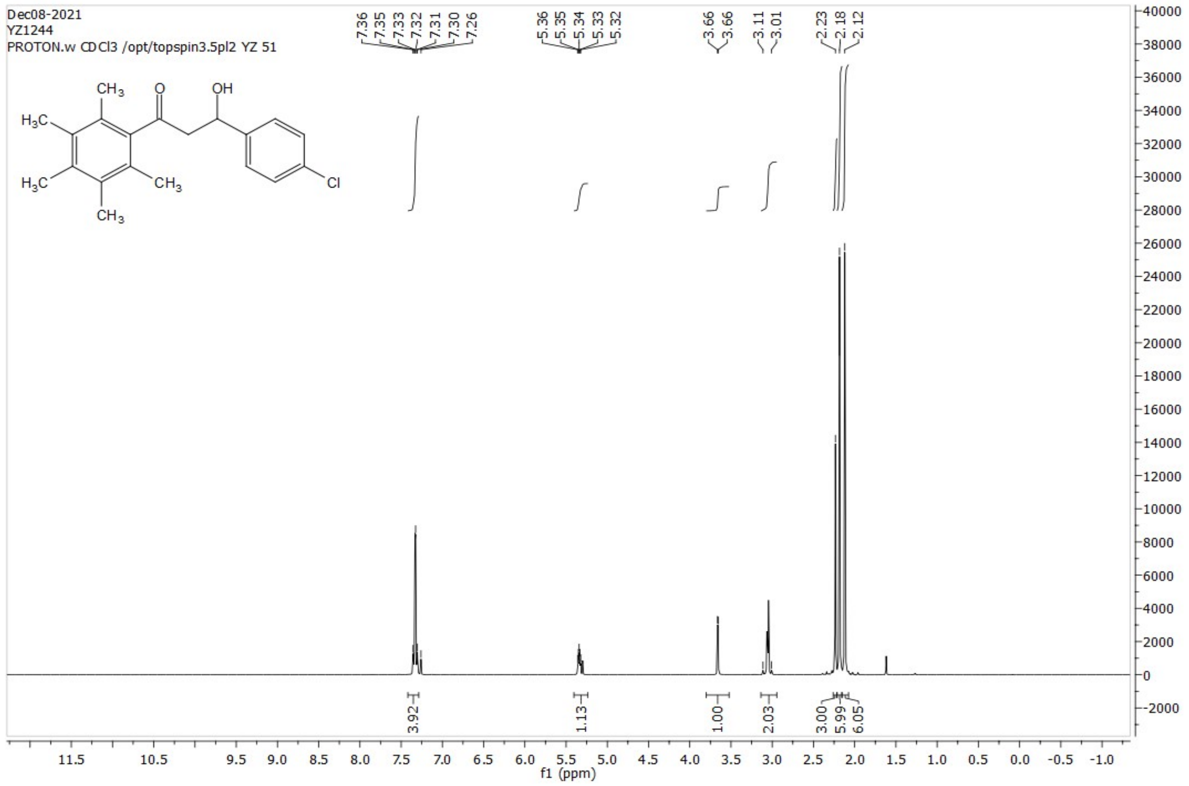

$^{13}C\{^{1}H\}$ NMR (100 MHz, $CDCl_3$) of (*R*)-3-(4-chlorophenyl)-3-hydroxy-1-(2,3,4,5,6-pentamethylphenyl)propan-1-one **15b**.

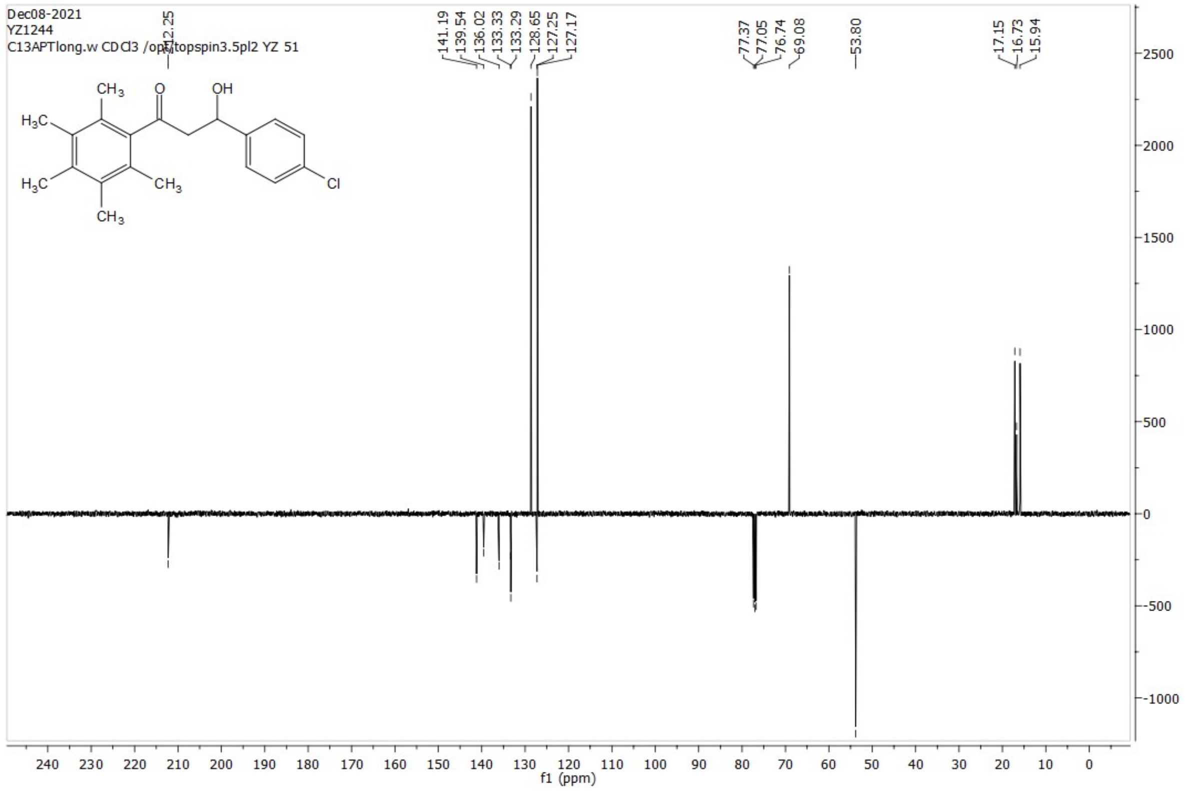

COSY (400 MHz, $CDCl_3$) of (*R*)-3-(4-chlorophenyl)-3-hydroxy-1-(2,3,4,5,6-pentamethylphenyl)propan-1-one **15b**.

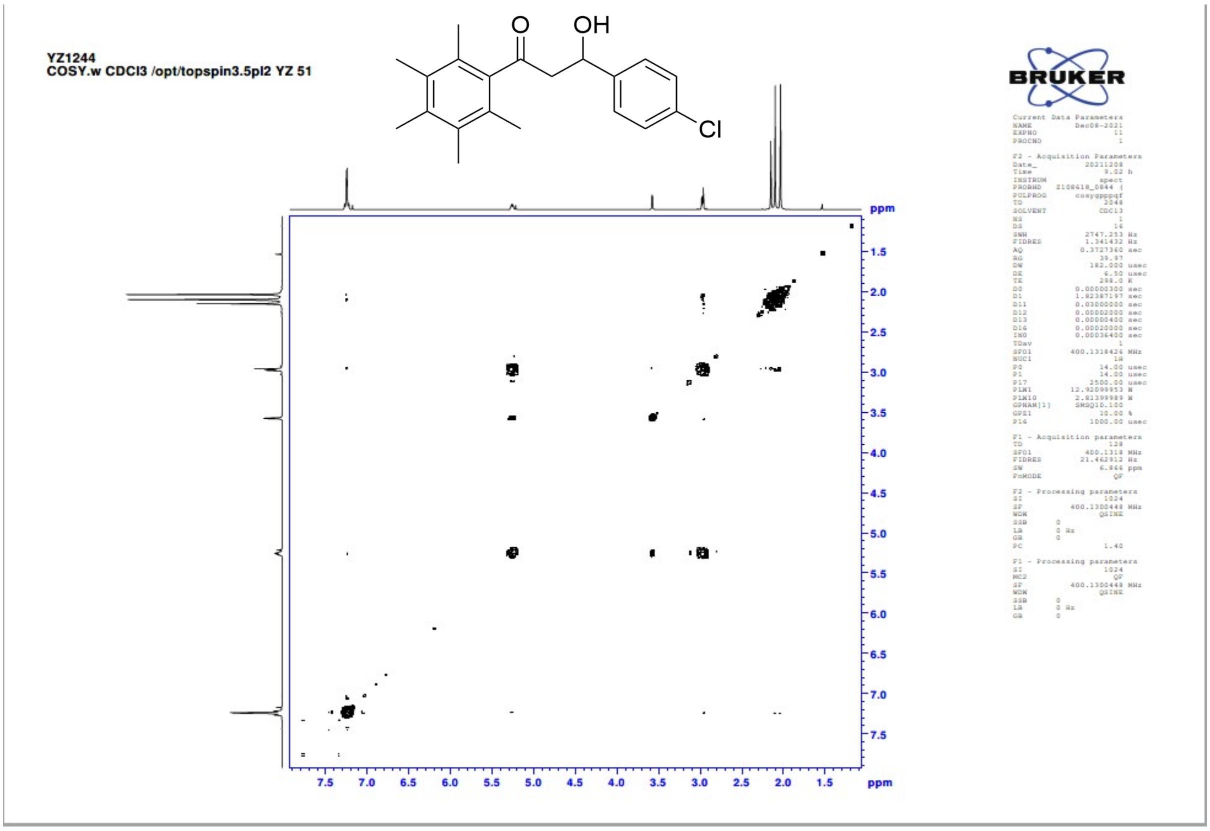

HSQC (400 MHz, $CDCl_3$) of (*R*)-3-(4-chlorophenyl)-3-hydroxy-1-(2,3,4,5,6-pentamethylphenyl)propan-1-one **15b**.

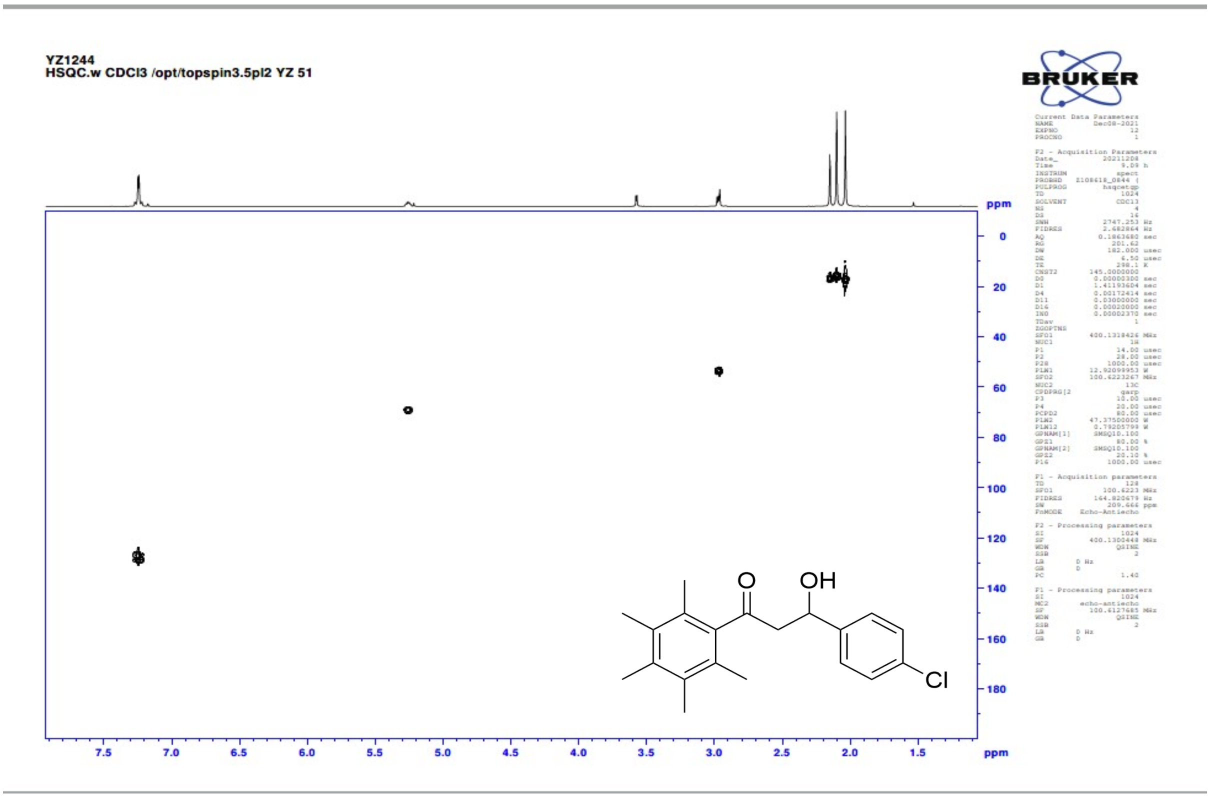

HMBC (400 MHz, $CDCl_3$) of (*R*)-3-(4-chlorophenyl)-3-hydroxy-1-(2,3,4,5,6-pentamethylphenyl)propan-1-one **15b**.

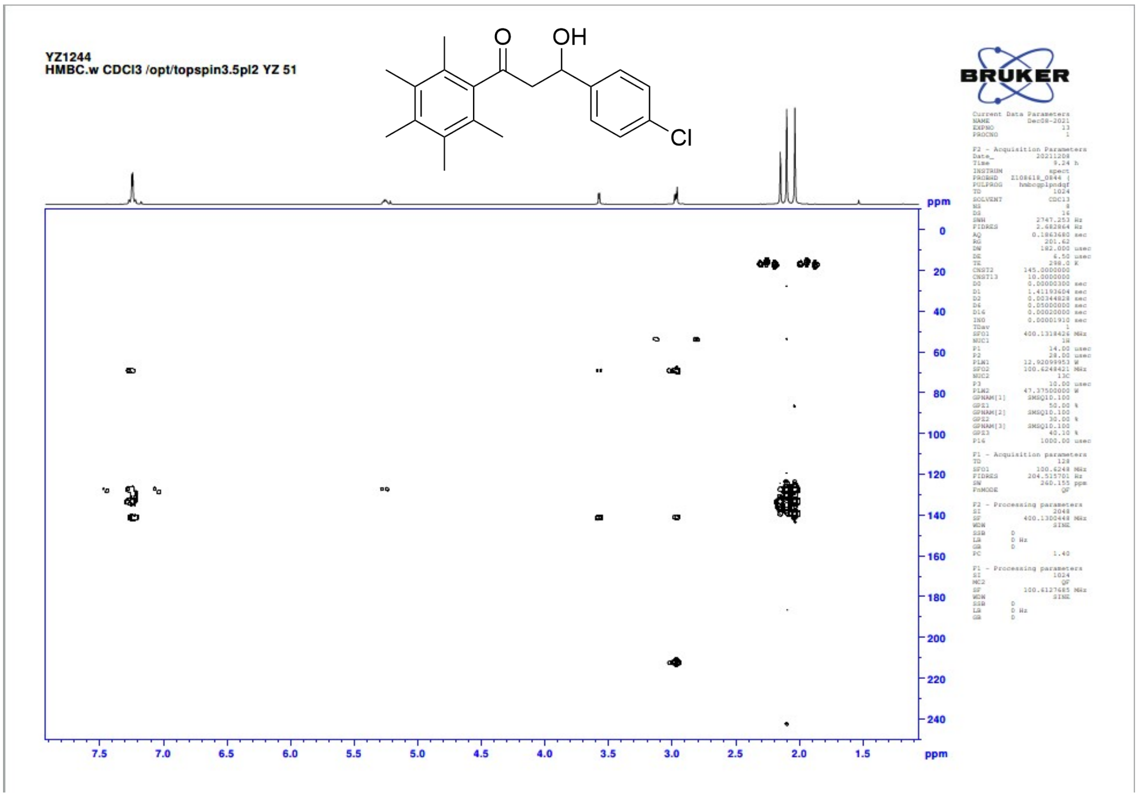

HPLC of racemic 3-(4-chlorophenyl)-3-hydroxy-1-(2,3,4,5,6-pentamethylphenyl)propan-1-one **15b.**

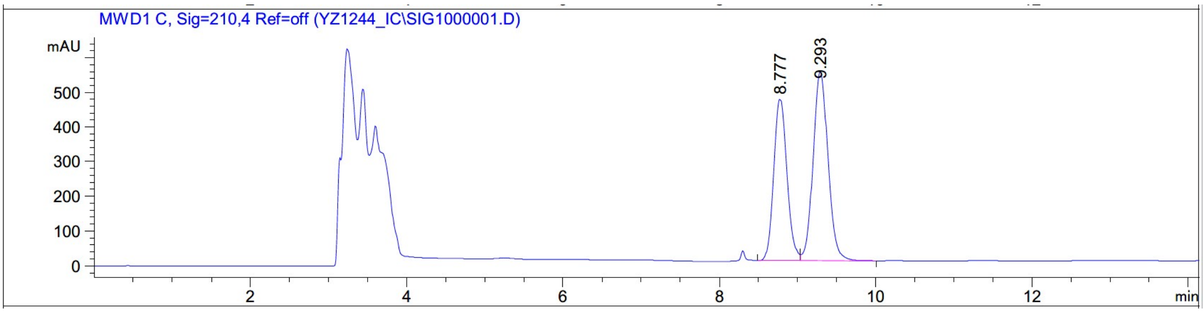

Signal 2: MWD1 C, Sig=210,4 Ref=off

Peak #	RetTime [min]	Type	Width [min]	Area [mAU*s]	Height [mAU]	Area %
1	8.777	BV	0.1830	5467.02637	465.41879	43.0384
2	9.293	VB	0.2051	7235.64795	544.21075	56.9616
Totals :				1.27027e4	1009.62955	

HPLC of (*R*)-3-(4-chlorophenyl)-3-hydroxy-1-(2,3,4,5,6-pentamethylphenyl)propan-1-one **15b..**

(*R,R*)-3C-Tethered Ru(II)-TsDPEN catalyst (after 24 h, 100% conversion, 98% ee, (*R*))

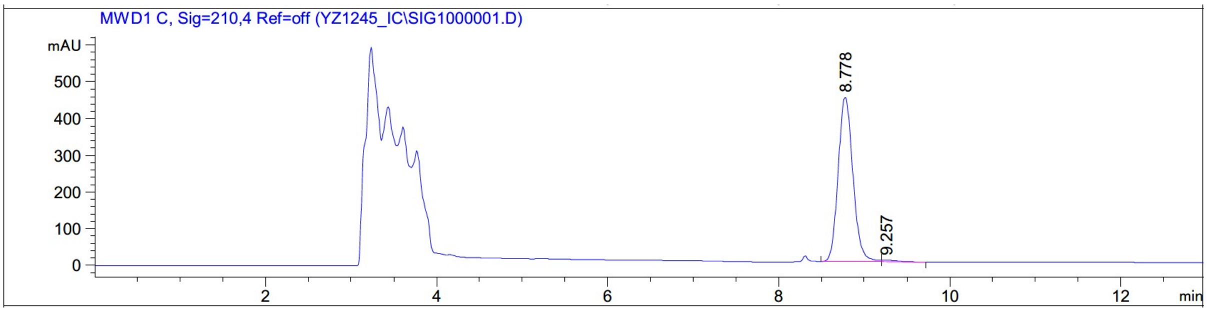

Signal 2: MWD1 C, Sig=210,4 Ref=off

Peak #	RetTime [min]	Type	Width [min]	Area [mAU*s]	Height [mAU]	Area %
1	8.778	BV	0.1859	5371.33643	448.00455	98.8616
2	9.257	VB	0.1972	61.84876	4.71003	1.1384

Totals : 5433.18519 452.71458

X-ray Crystallography Data for (*R*)-15b. CCDC Deposition Number 2276989.

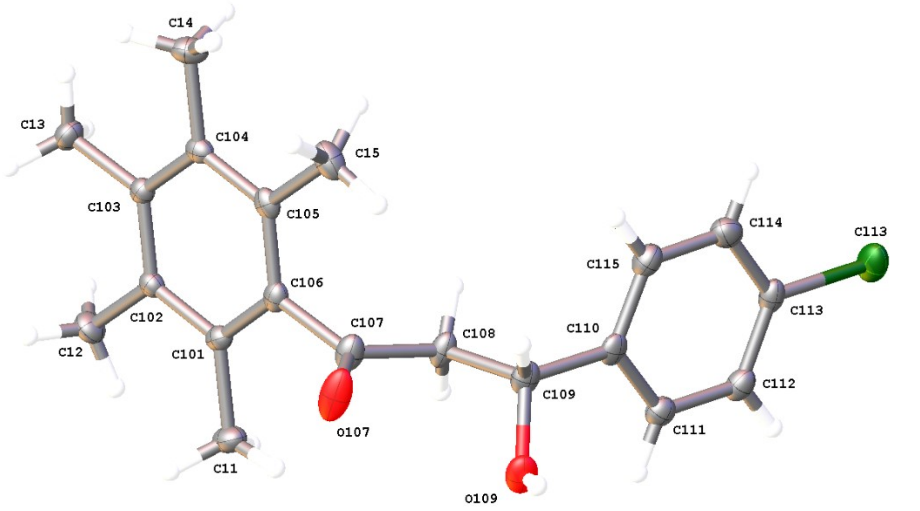

solid state structure of [one of the crystallographically independent but chemically identical molecules in the asymmetric unit] of **15b** with atom labelling and thermal parameters drawn at 50% probability level

Crystal structure determination of 15b.

The asymmetric unit contains two crystallographically independent but chemically identical molecules. Twice this in the unit cell. The OHs was located in a difference map and refined with restraints. O109 forms an intramolecular H bond with the carbonyl O207 of a neighbouring molecule.

Specified hydrogen bonds (with esds except fixed and riding H)

D-H	H...A	D...A	<(DHA)	
0.84	2.11	2.926(3)	162.7	O109-H109...O207

The Flack and associated Hooft y parameter refine to a small value with a small error so you can be confident in the assignment of the stereochemistry of the crystal chosen.

Flack x: 0.011(7) Shelx2018

Hooft y: 0.008(3) Olex 2

Experimental

Single crystals of $C_{20}H_{23}ClO_2$ **(15b)** were grown from DCM/Hexane. A suitable crystal was selected and mounted on a glass fibre with Fomblin oil and placed on a Rigaku Oxford Diffraction SuperNova diffractometer with a dual source (Cu at zero) equipped with an AtlasS2 CCD area detector.

The crystal was kept at 150(2) K during data collection. Using Olex2 [1], the structure was solved with the SHELXT [2] structure solution program using Intrinsic Phasing and refined with the SHELXL [3] refinement package using Least Squares minimisation.

1. Dolomanov, O.V., Bourhis, L.J., Gildea, R.J, Howard, J.A.K. & Puschmann, H. (2009), J. Appl. Cryst. 42, 339-341.
2. Sheldrick, G.M. (2015). Acta Cryst. A71, 3-8.
3. Sheldrick, G.M. (2015). Acta Cryst. C71, 3-8.

Crystal Data for $C_{20}H_{23}ClO_2$ (M =330.83 g/mol): monoclinic, space group $P2_1$ (no. 4), a = 11.57501(5) Å, b = 5.64957(3) Å, c = 26.39873(12) Å, β = 95.2951(4)°, V = 1718.948(15) Å^3, Z = 4, T = 150(2) K, μ(Cu Kα) = 2.015 mm^{-1}, $Dcalc$ = 1.278 g/cm^3, 54046 reflections measured (6.726° ≤ 2Θ ≤ 147.268°), 6635 unique (R_{int} = 0.0338, R_{sigma} = 0.0181) which were used in all calculations. The final R_1 was 0.0408 (I > 2σ(I)) and wR_2 was 0.1113 (all data).

**Table 1 Crystal data and structure refinement for 15b**	
Identification code	yz19
Empirical formula	$C_{20}H_{23}ClO_2$
Formula weight	330.83
Temperature/K	150(2)
Crystal system	monoclinic
Space group	$P2_1$
a/Å	11.57501(5)
b/Å	5.64957(3)
c/Å	26.39873(12)
α/°	90
β/°	95.2951(4)

γ/°	90
Volume/Å^3	1718.948(15)
Z	4
ρ_{calc}mg/mm^3	1.278
μ/mm^{-1}	2.015
F(000)	704.0
Crystal size/mm^3	0.28 × 0.1 × 0.08 colorless block
2Θ range for data collection	6.726 to 147.268°
Index ranges	-14 ≤ h ≤ 14, -7 ≤ k ≤ 6, -32 ≤ l ≤ 32
Reflections collected	54046
Independent reflections	6635[R(int) = 0.0338]
Data/restraints/parameters	6635/4/429
Goodness-of-fit on F^2	1.069
Final R indexes [I>=2σ (I)]	R_1 = 0.0408, wR_2 = 0.1105
Final R indexes [all data]	R_1 = 0.0415, wR_2 = 0.1113
Largest diff. peak/hole / e Å^{-3}	0.57/-0.32
Flack parameter	0.011(7)

1-(2,3,4,5,6-Pentamethylphenyl)-3-phenylpropane-1,3-dione.

.

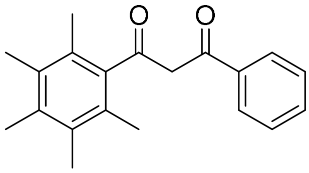

This compound is novel.

To a solution of sodium hydride (283 mg, 60% dispersion in mineral oil, 15.0 mmol) in THF (2 mL) at 0 °C was added dropwise a solution of 1-(2,3,4,5,6-pentamethylphenyl)ethan-1-one **6** (270 mg, 1.42 mmol) in THF (3.7 mL). The reaction mixture was stirred under a nitrogen atmosphere at 0 °C for 30 min and then stirred under a nitrogen atmosphere at rt for 30 min, after which ethyl benzoate (533 mg, 3.55 mmol) was added dropwise. The reaction mixture was heated to 65 °C and left stirring under the nitrogen atmosphere overnight. The reaction was followed by TLC (9:1 hexane: EtOAc). The mixture was quenched by 2M HCl solution (20 mL). EtOAc (20 mL) was added and the organic layer was separated. The aqueous layer was extracted with EtOAc (3 × 20 mL), and the combined organic layers were washed with saturated $NaHCO_3$ solution (2 × 20 mL) and brine (20 mL), dried ($MgSO_4$) and filtered. Solvent was removed to give the crude product. The product was isolated via flash chromatography on silica eluted with 0-10% EtOAc in hexane to give 1-(2,3,4,5,6-pentamethylphenyl)-3-phenylpropane-1,3-dione as a white solid (108 mg, 0.367 mmol, 26%). TLC: Rf ca 0.70 (9:1 hexane: EtOAc), strong UV and $KMnO_4$; Mp: 118°C; HRMS (ESI+) *m/z*: [M+H]+ Calcd for $C_{20}H_{23}O_2$ 295.1684; Found 295.1693; 2.8 ppm error; ν_{max} 3065, 2936, 1715, 1600, 1566, 1450, 1270, 1106, 1027, 1000 cm^{-1}; enol: keto = 47:3; ^{1}H NMR (400 MHz, $CDCl_3$): δ 7.95 (2H, d, *J* = 7.3, ArH), 7.55 (1H, t, *J* = 7.3, ArH), 7.47 (2H, t, *J* = 7.5, ArH), 6.34 (0.94H, s, CH of enol form), 4.41 (0.12H, s, CH_2 of keto form), 2.29 (3H, s, CH_3) 2.27 (5.64H, s, CH_3 of enol form), 2.25 (5.8H, s, CH_3 of enol form), 2.21 (0.36H, s, CH_3 of keto form), 2.15 (0.2H, s, CH_3 of keto form); ^{13}C{^{1}H} NMR (100 MHz, $CDCl_3$): δ 193.2 (C), 185.0 (C), 136.1 (C), 136.1 (C), 135.3 (C), 133.0 (C), 132.6 (CH), 129.5 (C), 128.7 (CH), 127.3 (CH), 99.5 (CH of enol form), 61.0 (CH_2 of keto form), 17.6 (CH_3), 16.9 (CH_3), 16.2 (CH_3) ppm; *m/z* (ES-API+) 317.1 (M^+ + 23, 100%).

^{1}H NMR (400 MHz, $CDCl_3$) of 1-(2,3,4,5,6-Pentamethylphenyl)-3-phenylpropane-1,3-dione.

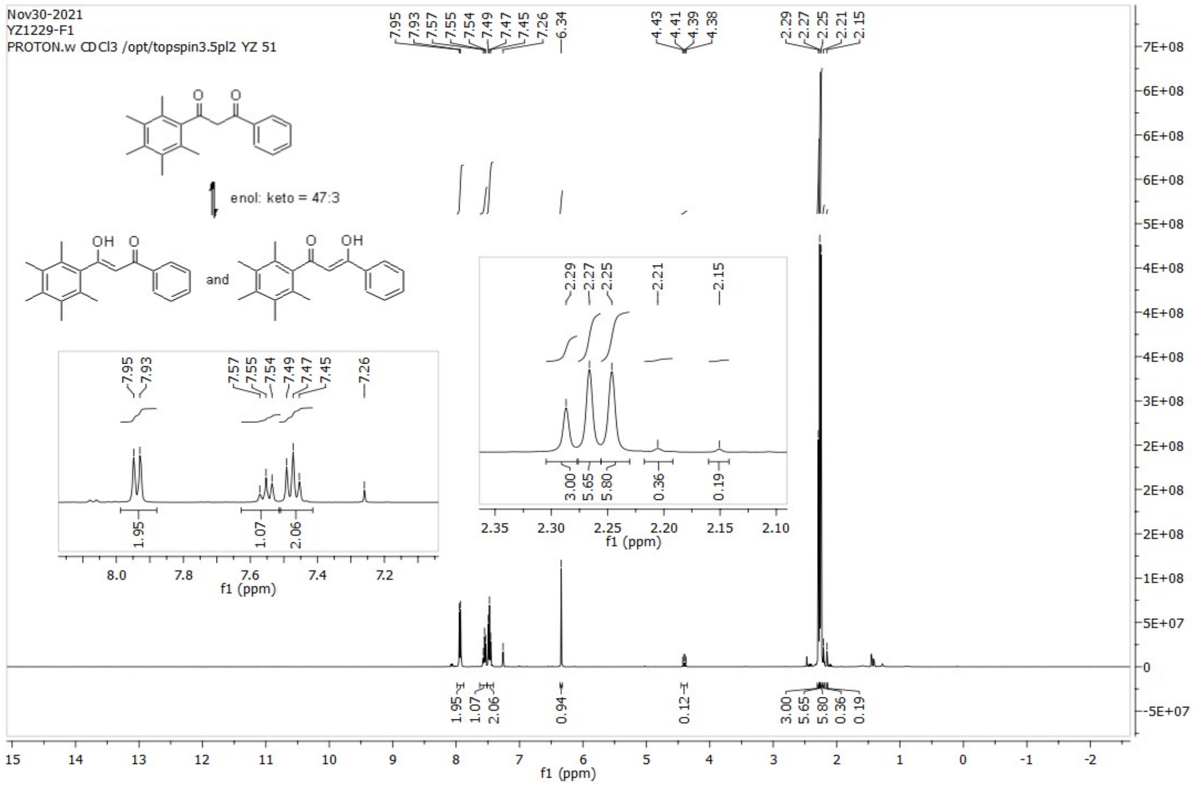

$^{13}C\{^1H\}$ NMR (100 MHz, $CDCl_3$) of 1-(2,3,4,5,6-Pentamethylphenyl)-3-phenylpropane-1,3-dione.

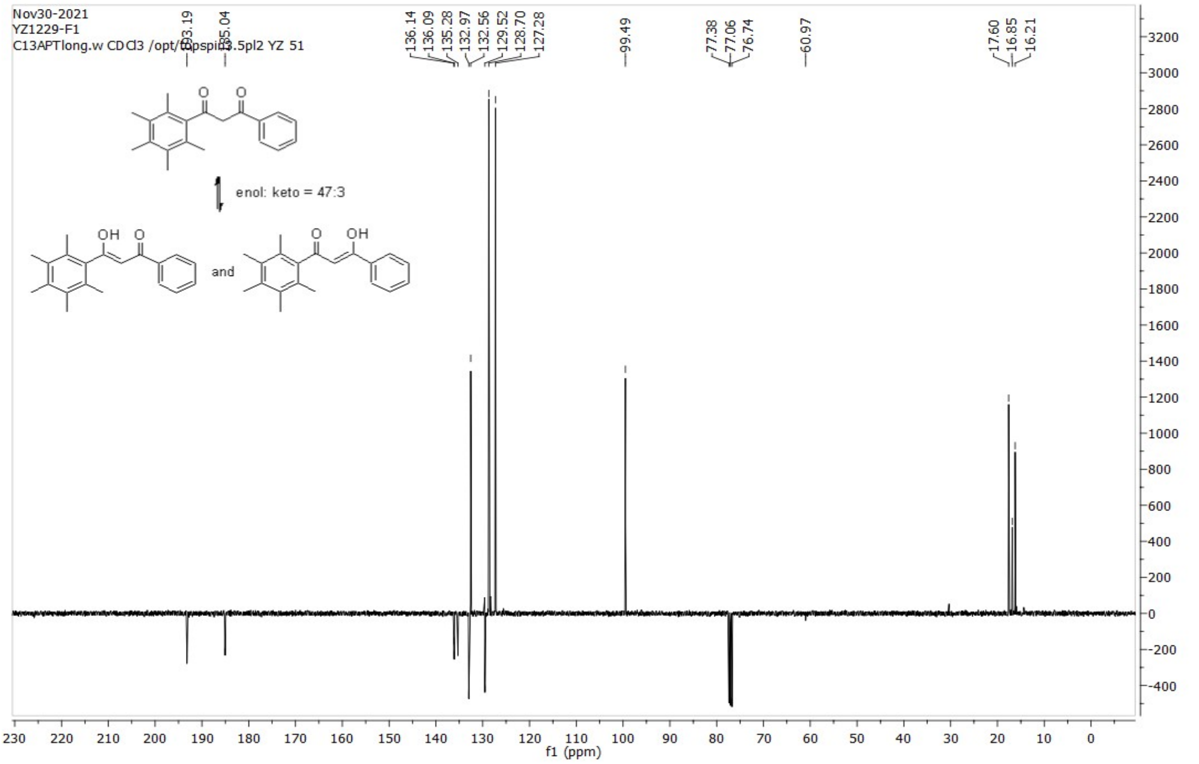

COSY (400 MHz, $CDCl_3$) of 1-(2,3,4,5,6-Pentamethylphenyl)-3-phenylpropane-1,3-dione.

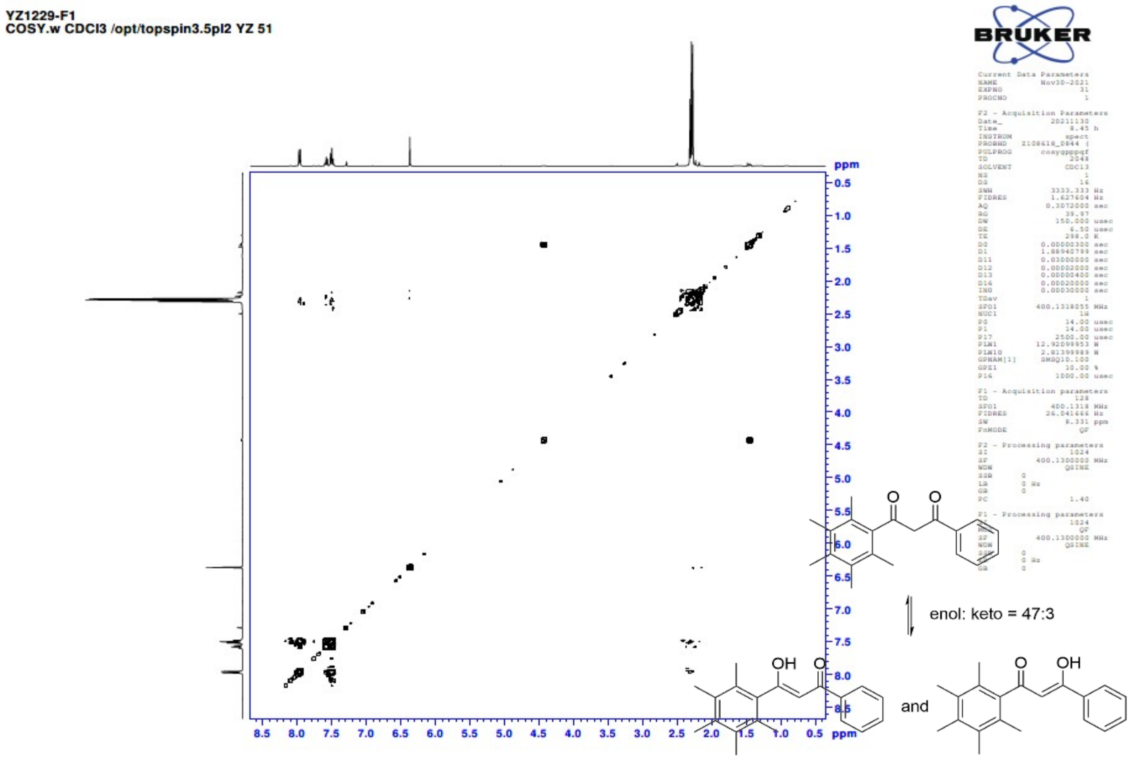

HSQC (400 MHz, $CDCl_3$) of 1-(2,3,4,5,6-Pentamethylphenyl)-3-phenylpropane-1,3-dione.

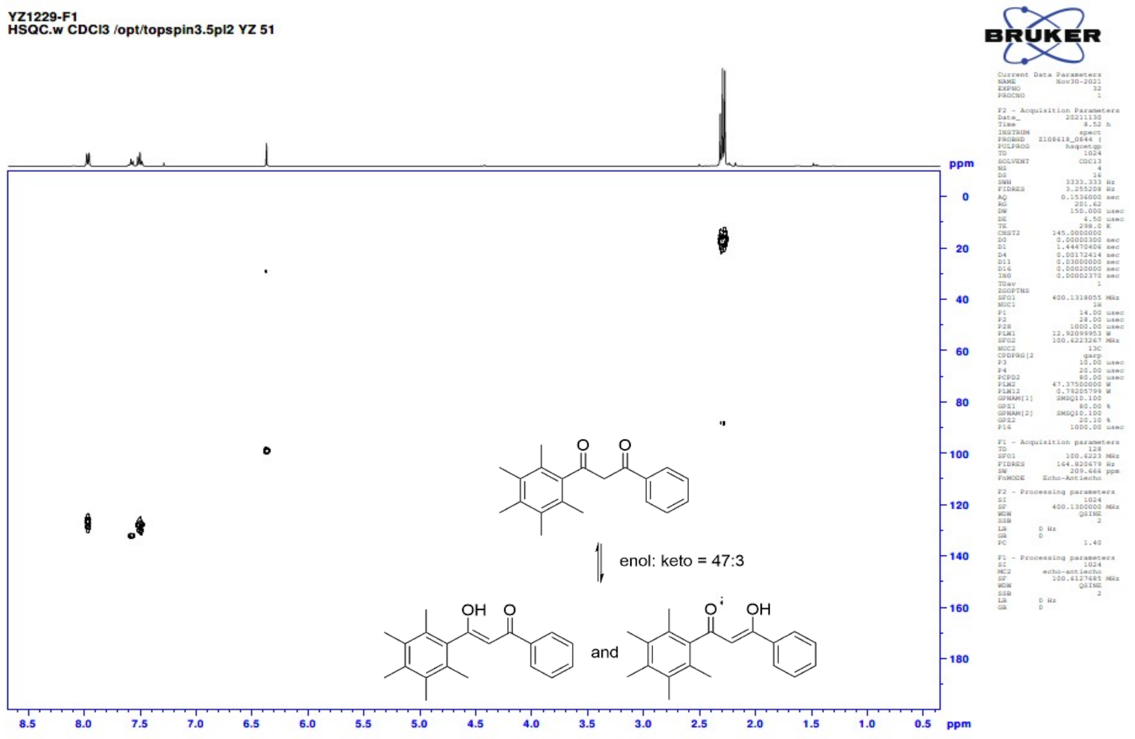

HMBC (400 MHz, $CDCl_3$) of 1-(2,3,4,5,6-Pentamethylphenyl)-3-phenylpropane-1,3-dione.

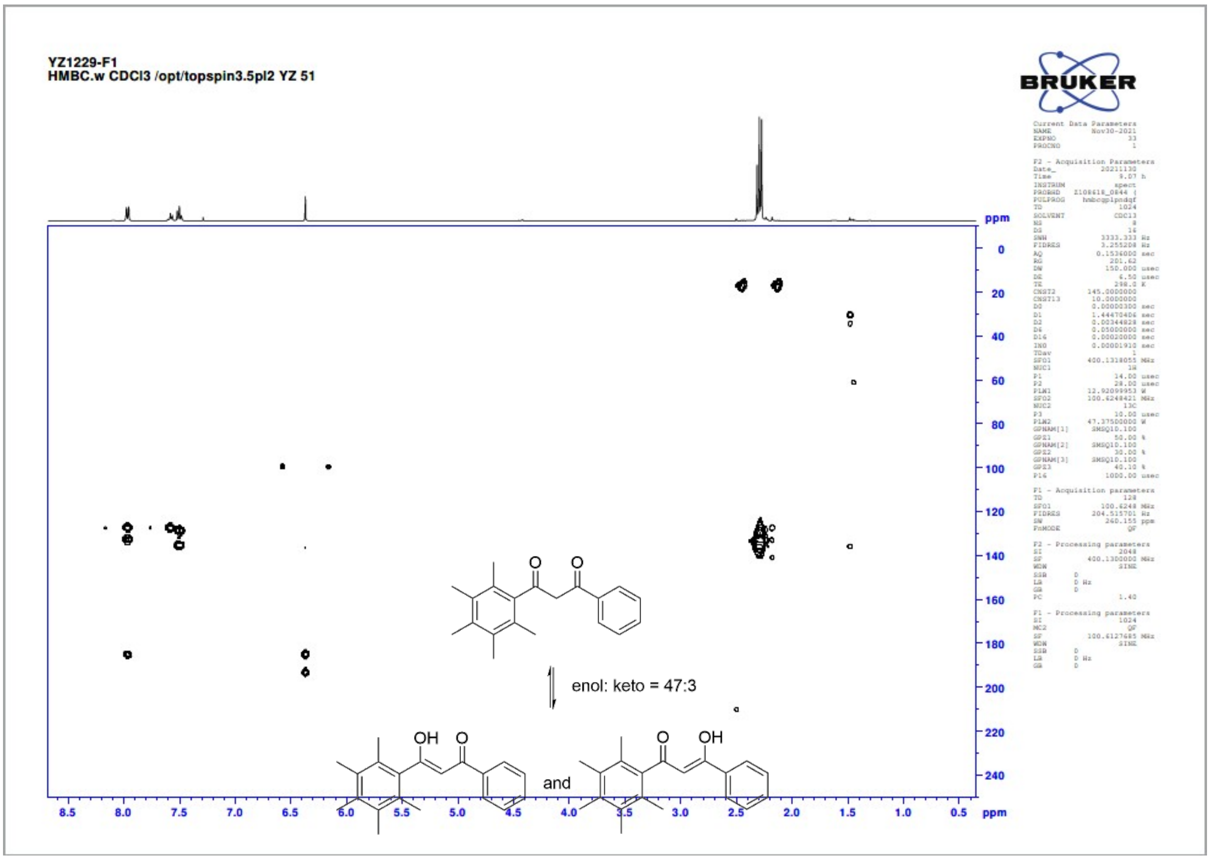

3-Hydroxy-1-(2,3,4,5,6-pentamethylphenyl)-3-phenylpropan-1-one.

This example is not featured in the paper because the enantiomers could not be resolved by HPLC and the ee could not be determined but is reproduced here for information.

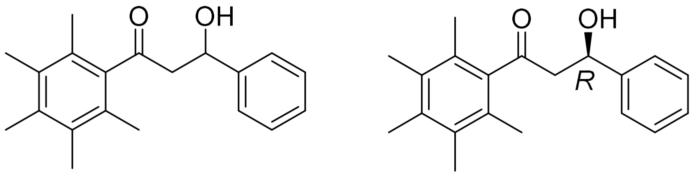

This compound is novel.

Synthesis of a racemic standard: (*R,R*)-3C-Tethered Ru(II)-TsDPEN catalyst (0.8 mg, 1.4 mmol, 0.5 mol%) and (*S,S*)-3C-tethered Ru(II)-TsDPEN catalyst (0.8 mg, 1.4 mmol, 0.5 mol%) were added to FA: TEA (5:2 azeotropic mixture, 0.36 mL) at rt and the mixture was stirred under a nitrogen atmosphere for 15 minutes; after which a solution of 1-(2,3,4,5,6-pentamethylphenyl)-3-phenylpropane-1,3-dione (80.0 mg, 0.272 mmol) in DCM (0.50 mL) was added. The reaction mixture was stirred under a nitrogen atmosphere and followed by TLC (9:1 hexane: EtOAc). After 24 h, the reaction was quenched using saturated $NaHCO_3$ solution (20 mL). EtOAc (20 mL) was added and the organic layer was separated. The aqueous layer was extracted with EtOAc (3 x 20 mL) and the combined organic layers were dried ($MgSO_4$) and filtered. The solvent was removed to give the crude product. The product was isolated via flash chromatography on silica eluted with 0-10% EtOAc in hexane to give 3-hydroxy-1-(2,3,4,5,6-pentamethylphenyl)-3-phenylpropan-1-one as a colorless oil (50.0 mg, 0.169 mmol, 62%). TLC: Rf ca 0.20 (9:1 hexane: EtOAc), strong UV and $KMnO_4$; HRMS (ESI+) *m/z*: [M+H]+ Calcd for $C_{20}H_{24}NaO_2$ 319.1664; Found 319.1669; 1.5 ppm error; ν_{max} 3451 (br), 3028, 2902, 1692, 1451, 1383, 1113, 1056, 936 cm^{-1}; ^1H NMR (400 MHz, $CDCl_3$): δ 7.33-7.16 (5H, m, ArH), 5.29 (1H, dt, *J* = 7.0, 3.1, ArCH), 3.50 (1H, d, *J* = 3.1, OH), 3.11-2.95 (2H, m, CH_2), 2.14 (3H, s, CH_3), 2.09 (6H, s, CH_3), 2.04 (6H, s, CH_3); $^{13}C\{^1H\}$ NMR (100 MHz, $CDCl_3$): δ 212.4 (C), 142.7 (C), 139.7 (C), 135.9 (C), 133.2 (C), 128.5 (CH), 127.7 (CH), 127.3 (C), 125.8 (CH), 69.7 (CH), 53.9 (CH_2), 17.1 (CH_3), 16.7 (CH_3), 15.9 (CH_3) ppm; *m/z* (ES-API+) 319.1 (M^+ + 1, 100%).

(*R*)-3-Hydroxy-1-(2,3,4,5,6-pentamethylphenyl)-3-phenylpropan-1-one.

(*R,R*)-3C-tethered Ru(II)-TsDPEN catalyst (1.9 mg, 3.0 mmol, 1 mol%) was added to FA: TEA (5:2 azeotropic mixture, 0.41 mL) at rt and the mixture was stirred under a nitrogen atmosphere for 15 minutes; after which a solution of 1-(2,3,4,5,6-pentamethylphenyl)-3-phenylpropane-1,3-dione (90.0 mg, 0.306 mmol) in DCM (0.56 mL) was added. The reaction mixture was stirred under a nitrogen atmosphere and followed by TLC (9:1 hexane: EtOAc). After 24 h, the reaction was quenched using saturated $NaHCO_3$ solution (20 mL). EtOAc (20 mL) was added and the organic layer was separated. The aqueous layer was extracted with EtOAc (3 x 20 mL) and the combined organic layers were dried ($MgSO_4$) and filtered. The solvent was removed to give the crude product. The product was isolated via flash chromatography on silica eluted with 0-10% EtOAc in hexane to give (*R*)-3-hydroxy-1-(2,3,4,5,6-pentamethylphenyl)-3-phenylpropan-1-one as a colorless oil (63.0 mg, 0.213 mmol, 70%). The reaction mixture could not be separated in HPLC therefore an ee could not be determined: after 24 h, 100% conversion; $[\alpha]_D^{26}$ +31.5 (c 0.143 in $CHCl_3$). The configuration was assigned by analogy.

^{1}H NMR (400 MHz, $CDCl_3$) of 3-hydroxy-1-(2,3,4,5,6-pentamethylphenyl)-3-phenylpropan-1-one.

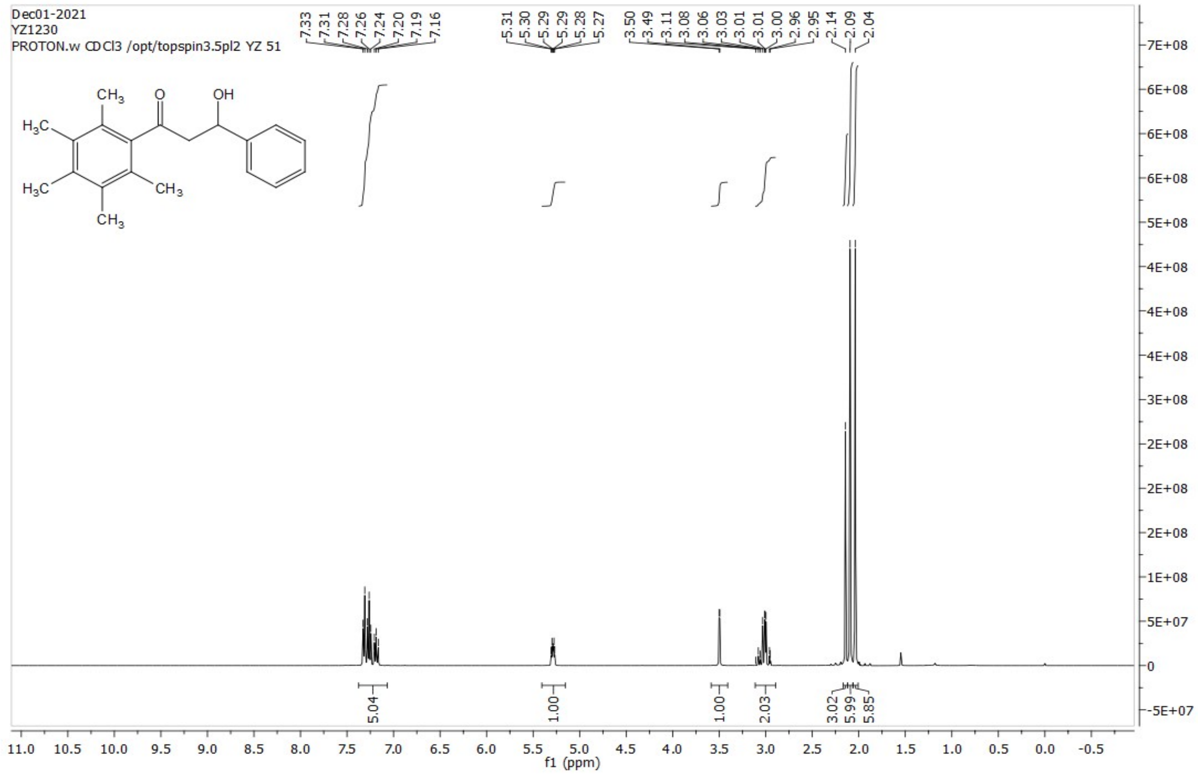

$^{13}C\{^{1}H\}$ NMR (100 MHz, $CDCl_3$) of 3-hydroxy-1-(2,3,4,5,6-pentamethylphenyl)-3-phenylpropan-1-one.

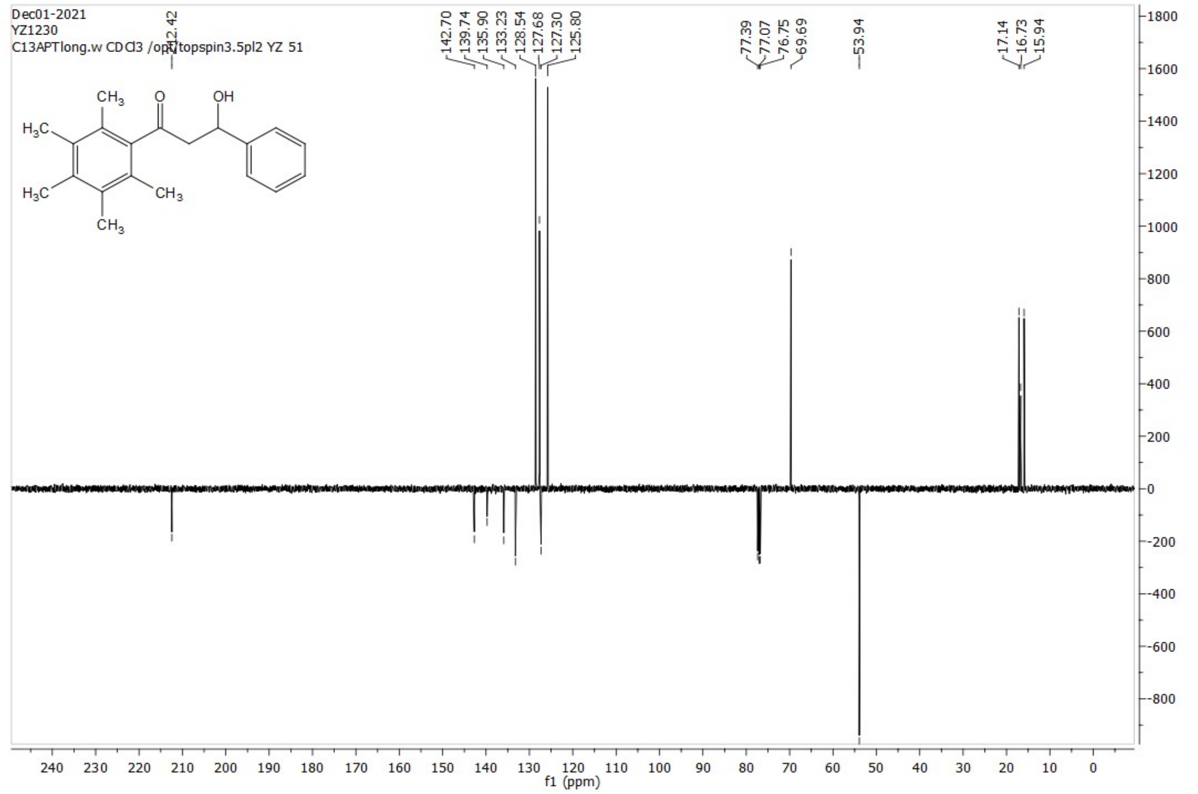

COSY (400 MHz, $CDCl_3$) of 3-hydroxy-1-(2,3,4,5,6-pentamethylphenyl)-3-phenylpropan-1-one.

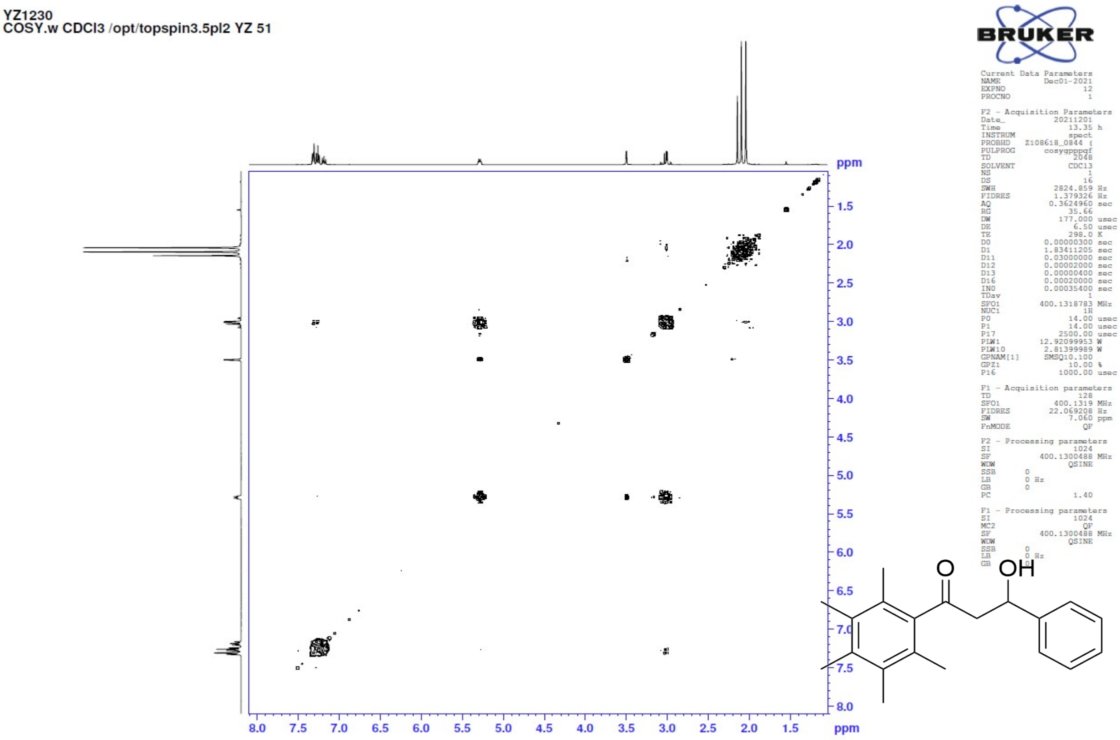

HSQC (400 MHz, $CDCl_3$) of 3-hydroxy-1-(2,3,4,5,6-pentamethylphenyl)-3-phenylpropan-1-one.

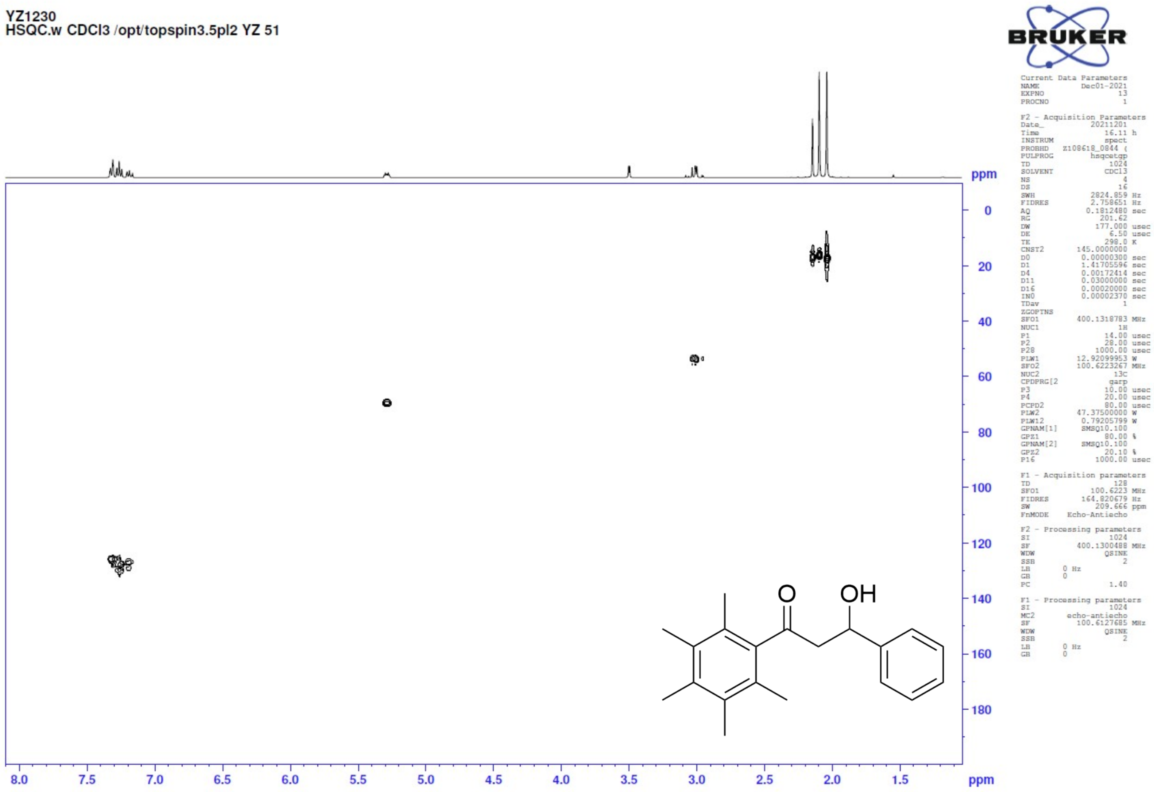

HMBC (400 MHz, $CDCl_3$) of 3-hydroxy-1-(2,3,4,5,6-pentamethylphenyl)-3-phenylpropan-1-one.

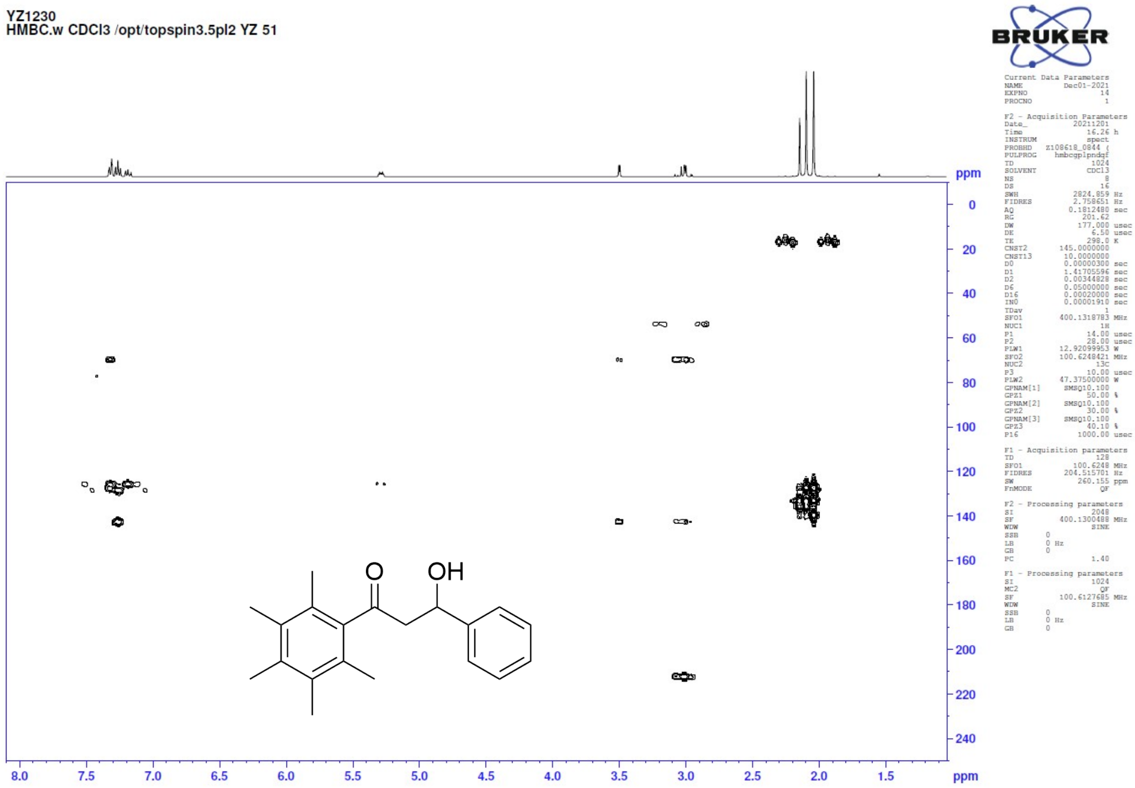

1-(2-Methoxyphenyl)-3-(2,3,4,5,6-pentamethylphenyl)propane-1,3-dione 16a.

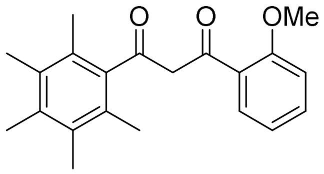

This compound is novel.

To a solution of sodium hydride (272 mg, 60% dispersion in mineral oil, 6.78 mmol) in THF (1.4 mL) at 0 °C was added dropwise a solution of 1-(2,3,4,5,6-pentamethylphenyl)ethan-1-one **6** (258 mg, 1.36 mmol) in THF (2 mL). The reaction mixture was stirred under a nitrogen atmosphere at 0 °C for 30 min and then stirred under a nitrogen atmosphere at rt for 30 min, after which a solution of ethyl 2-methoxybenzoate (1.22 g, 6.78 mmol) in THF (2 mL) was added dropwise. The reaction mixture was heated to 65 °C and left stirring under the nitrogen atmosphere overnight. The reaction was followed by TLC (9:1 hexane: EtOAc). The mixture was quenched by 2M HCl solution (20 mL). EtOAc (20 mL) was added and the organic layer was separated. The aqueous layer was extracted with EtOAc (3 × 20 mL), and the combined organic layers were washed with saturated $NaHCO_3$ solution (2 × 20 mL) and brine (20 mL), dried ($MgSO_4$) and filtered. Solvent was removed to give the crude product. The product was isolated via flash chromatography on silica eluted with 3% EtOAc in hexane to give 1-(2-methoxyphenyl)-3-(2,3,4,5,6-pentamethylphenyl)propane-1,3-dione **16a** as a white solid (128 mg, 0.395 mmol, 29%). TLC: Rf ca 0.60 (9:1 hexane: EtOAc), strong UV and $KMnO_4$; Mp: 153°C; HRMS (ESI+) *m/z*: [M+H]+ Calcd for $C_{21}H_{24}NaO_3$ 347.1612; Found 347.1618; 1.6 ppm error; ν_{max} 2922, 1600, 1488, 1453, 1248, 1226, 1178, 1163, 1017, 809 cm^{-1}; enol: keto = 100:0; ^{1}H NMR (400 MHz, $CDCl_3$): δ 8.00 (1H, dd, *J* = 7.8, 1.6, ArH), 7.49-7.45 (1H, m, ArH), 7.08 (1H, t, *J* = 7.5, ArH), 6.98 (1H, d, *J* = 8.4, ArH), 6.61 (1H, s, CH of enol form), 3.86 (3H, s, OCH_3), 2.29 (9H, s, CH_3), 2.25 (6H, s, CH_3); $^{13}C\{^1H\}$ NMR (100 MHz, $CDCl_3$): δ 193.1 (C), 183.8 (C), 158.7 (C), 136.6 (C), 135.9 (C), 133.2 (CH), 132.9 (C), 130.5 (CH), 129.7 (C), 124.8 (C), 120.8 (CH), 111.6 (CH), 104.9 (CH of enol form), 55.6 (OCH_3), 17.6 (CH_3), 16.9 (CH_3), 16.2 (CH_3); *m/z* (ES-API+) 347.1 (M^+ + 23, 100%).

Enantiomeric excess and conversion determined by HPLC analysis (Chiralpak IC, 30 cm x 6 mm column, hexane:iPrOH 90:10, 1.0 mL/min, T = 25°C) ketone 11.5 min, *R* and *S* isomers 18.8 min and 20.2 min, configuration assigned by analogy.

^{1}H NMR (400 MHz, $CDCl_3$) of 1-(2-methoxyphenyl)-3-(2,3,4,5,6-pentamethylphenyl)propane-1,3-dione **16a**.

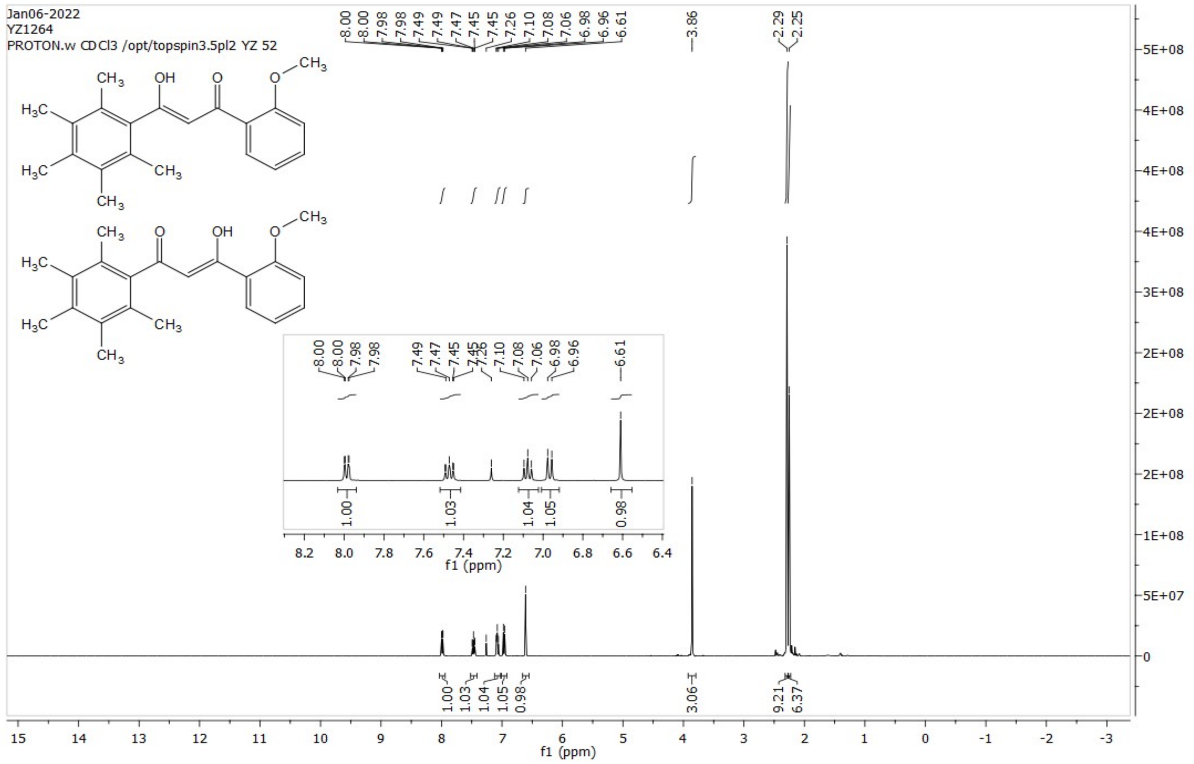

^{13}C{^{1}H} NMR (100 MHz, $CDCl_3$) of 1-(2-methoxyphenyl)-3-(2,3,4,5,6-pentamethylphenyl)propane-1,3-dione **16a**.

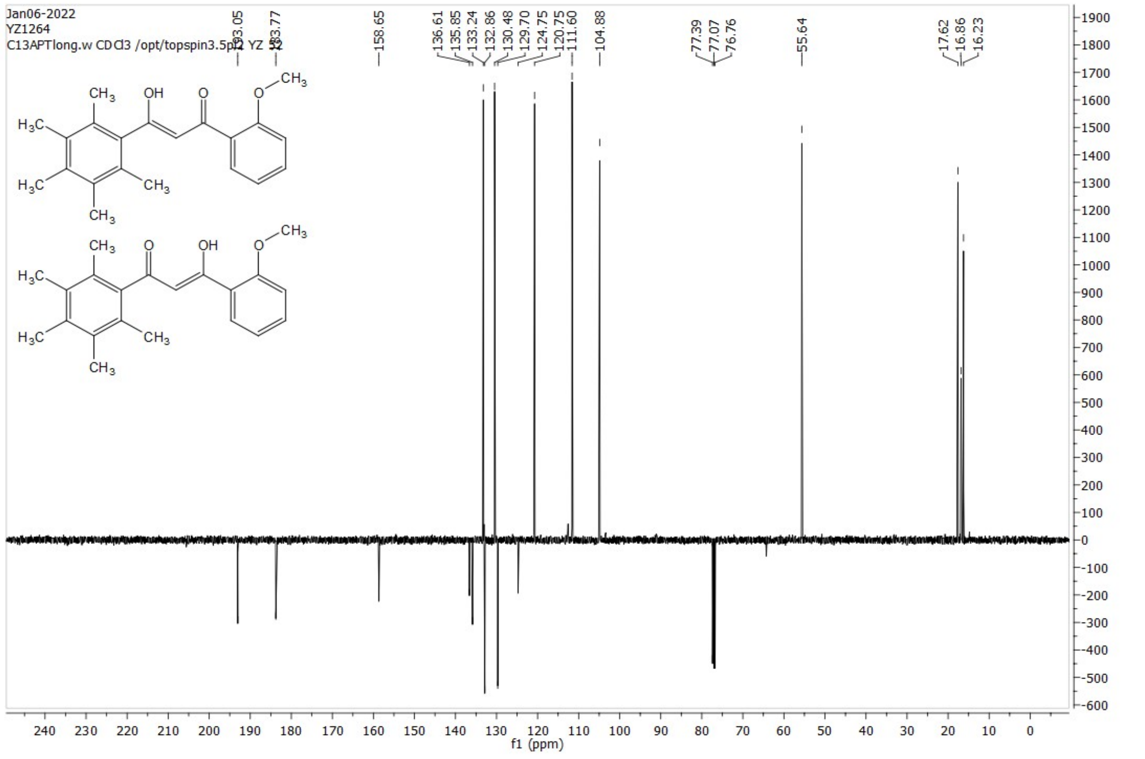

COSY (400 MHz, $CDCl_3$) of 1-(2-methoxyphenyl)-3-(2,3,4,5,6-pentamethylphenyl)propane-1,3-dione **16a**.

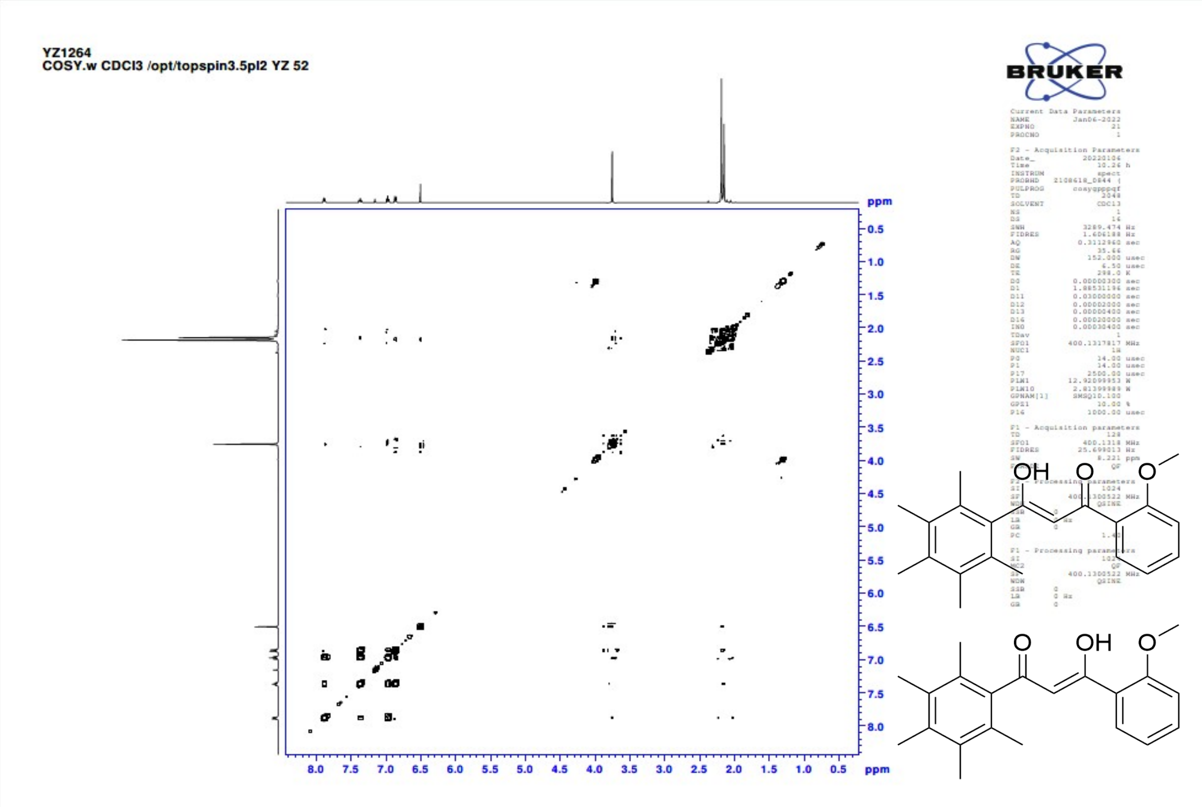

HSQC (400 MHz, $CDCl_3$) of 1-(2-Methoxyphenyl)-3-(2,3,4,5,6-pentamethylphenyl)propane-1,3-dione **16a**.

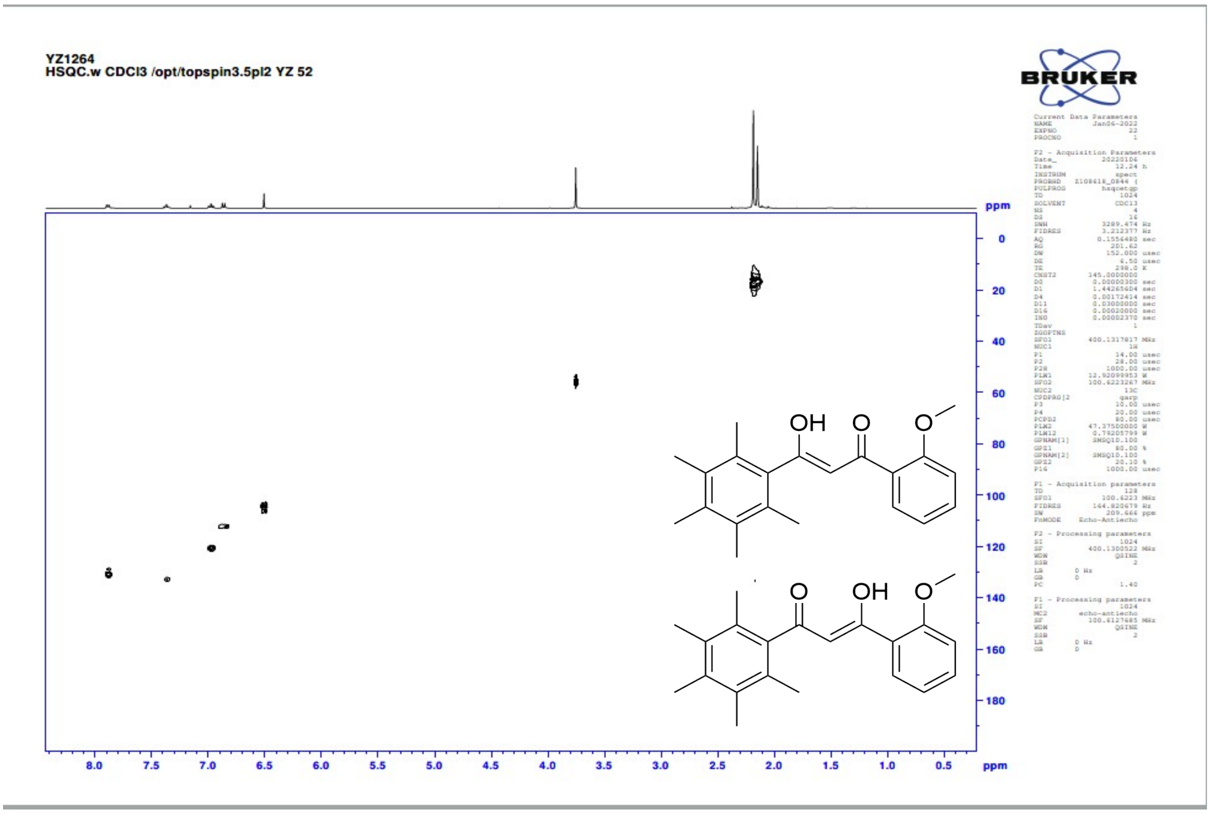

HMBC (400 MHz, $CDCl_3$) of 1-(2-methoxyphenyl)-3-(2,3,4,5,6-pentamethylphenyl)propane-1,3-dione **16a**.

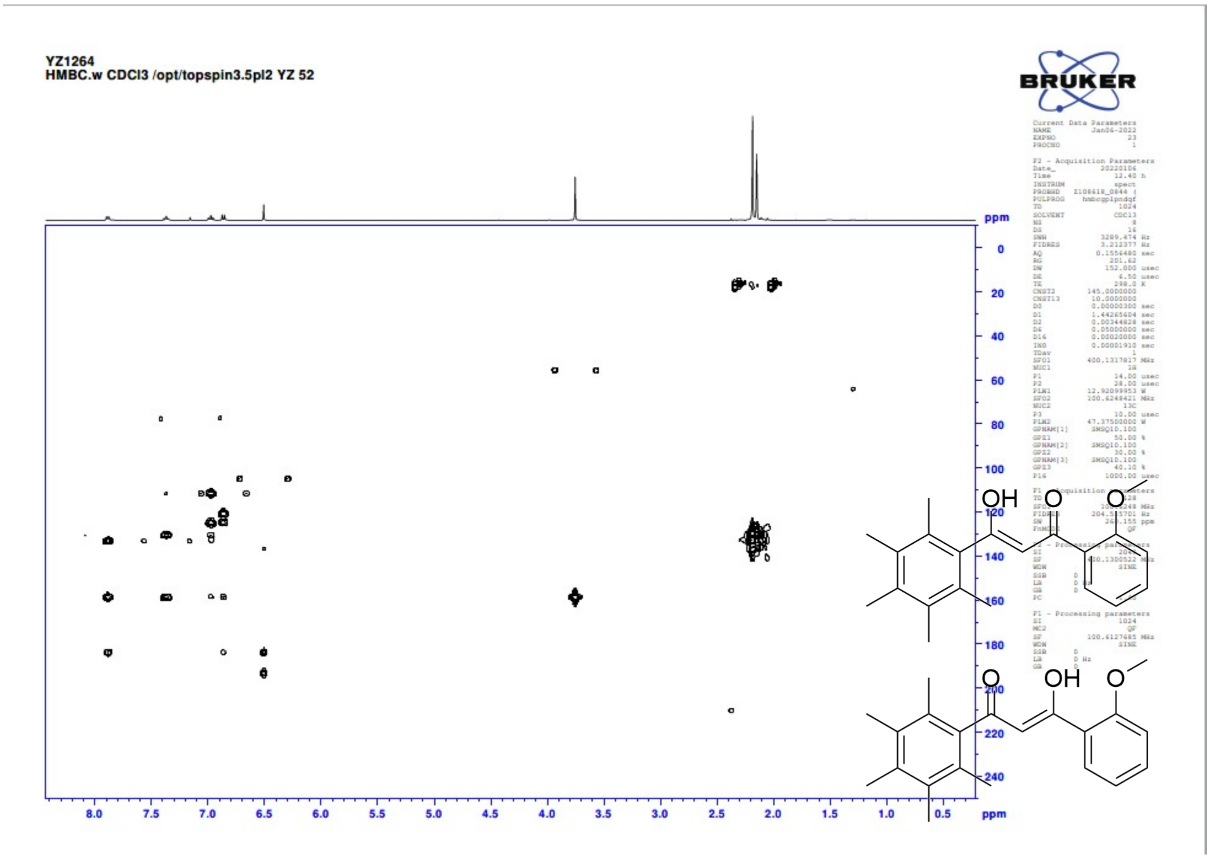

HPLC of 1-(2-methoxyphenyl)-3-(2,3,4,5,6-pentamethylphenyl)propane-1,3-dione **16a.**

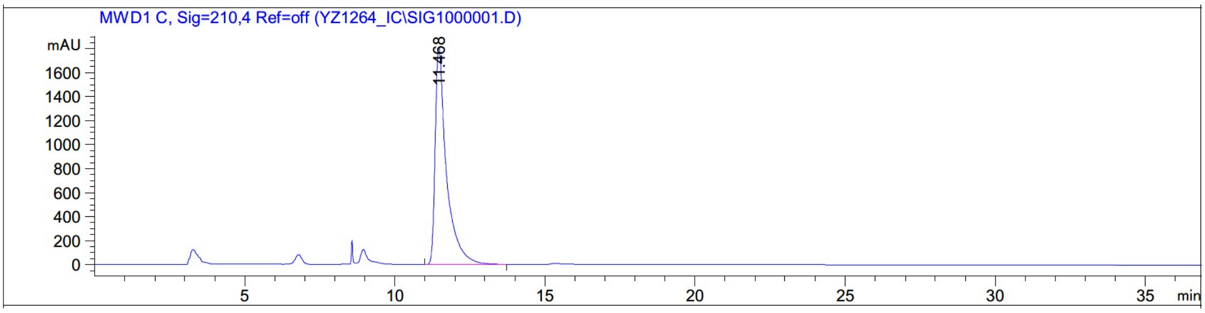

Signal 2: MWD1 C, Sig=210,4 Ref=off

Peak #	RetTime [min]	Type	Width [min]	Area [mAU*s]	Height [mAU]	Area %
1	11.468	BV	0.3716	4.71793e4	1813.25635	100.0000
Totals :				4.71793e4	1813.25635	

3-Hydroxy-3-(2-methoxyphenyl)-1-(2,3,4,5,6-pentamethylphenyl)propan-1-one 16b.

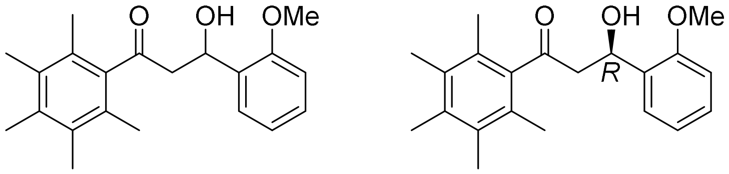

This compound is novel.

Synthesis of a racemic standard:

(*R,R*)-3C-Tethered Ru(II)-TsDPEN catalyst (0.8 mg, 1.2 mmol, 0.5 mol%) and (*S,S*)-3C-tethered Ru(II)-TsDPEN catalyst (0.8 mg, 1.2 mmol, 0.5 mol%) were added to FA: TEA (5:2 azeotropic mixture, 0.36 mL) at rt and the mixture was stirred under a nitrogen atmosphere for 15 minutes; after which a solution of 1-(2-methoxyphenyl)-3-(2,3,4,5,6-pentamethylphenyl)propane-1,3-dione **16a** (80.0 mg, 0.247 mmol) in DCM (0.50 mL) was added. The reaction mixture was stirred under a nitrogen atmosphere and followed by TLC (9:1 hexane: EtOAc). After 24 h, the reaction was quenched using saturated $NaHCO_3$ solution (20 mL). EtOAc (20 mL) was added and the organic layer was separated. The aqueous layer was extracted with EtOAc (3 x 20 mL) and the combined organic layers were dried ($MgSO_4$) and filtered. The solvent was removed to give the crude product. The product was isolated via flash chromatography on silica eluted with 0-20% EtOAc in hexane to 3-hydroxy-3-(2-methoxyphenyl)-1-(2,3,4,5,6-pentamethylphenyl)propan-1-one **16b** as a white solid (50.0 mg, 0.123 mmol, 50%). TLC: Rf ca 0.20 (9:1 hexane: EtOAc), strong UV and $KMnO_4$; Mp: 120 °C; HRMS (ESI+) *m/z*: [M+H]+ Calcd for $C_{21}H_{26}NaO_3$ 349.1768; Found 349.1774; 1.7 ppm error; ν_{max} 3557 (br), 3001, 2921, 2834, 1697, 1488, 1460, 1437, 1353, 1280, 1235, 1110, 1059, 1047, 1025, 1010, 752 cm^{-1}; ^1H NMR (400 MHz, $CDCl_3$): δ 7.45 (1H, d, *J* = 7.3, ArH), 7.17-7.13 (1H, m, ArH), 6.90 (1H, t, *J* = 7.3, ArH), 6.77 (1H, d, *J* = 8.2, ArH), 5.54 (1H, dd, *J* = 5.4, 3.4, ArCH), 3.73 (3H, s, OCH_3), 3.68 (1H, d, *J* = 4.3, OH), 3.19 (1H, dd, *J* = 18.7, 2.3, CH_2), 2.90 (1H, dd, *J* = 18.7, 9.0, CH_2), 2.14 (3H, s, CH_3), 2.09 (6H, s, CH_3), 2.04 (6H, s, CH_3); $^{13}C\{^1H\}$ NMR (100 MHz, $CDCl_3$): δ 213.1 (C), 155.7 (C), 140.0 (C), 135.7 (C), 133.1 (C), 130.9 (C), 128.3 (CH), 127.4 (C), 126.4 (CH), 120.8 (CH), 110.1 (CH), 65.4 (CH), 55.2 (CH_3), 52.3 (CH_2), 17.0 (CH_3), 16.7 (CH_3), 15.9 (CH_3); *m/z* (ES-API+) 349.2 (M^+ + 23, 100%).

Enantiomeric excess and conversion determined by HPLC analysis (Chiralpak IC, 30 cm x 6 mm column, hexane:iPrOH 90:10, 1.0 mL/min, T = 25°C) ketone 11.5 min, *R* and *S* isomers 18.8 min and 20.2 min, configuration assigned by analogy.

(R)-3-Hydroxy-3-(2-methoxyphenyl)-1-(2,3,4,5,6-pentamethylphenyl)propan-1-one **16b**.

(*R,R*)-3C-tethered Ru(II)-TsDPEN catalyst (1.5 mg, 2.5 mmol, 1 mol%) was added to FA: TEA (5:2 azeotropic mixture, 0.36 mL) at rt and the mixture was stirred under a nitrogen atmosphere for 15 minutes; after which a solution of 1-(2-methoxyphenyl)-3-(2,3,4,5,6-pentamethylphenyl)propane-1,3-dione **16a** (80.0 mg, 0.247 mmol) in DCM (0.50 mL) was added. The reaction mixture was stirred under a nitrogen atmosphere and followed by TLC (9:1 hexane: EtOAc). After 24 h, the reaction was quenched using saturated $NaHCO_3$ solution (20 mL). EtOAc (20 mL) was added and the organic layer was separated. The aqueous layer was extracted with EtOAc (3 x 20 mL) and the combined organic layers were dried ($MgSO_4$) and filtered. The solvent was removed to give the crude product. The product was isolated via flash chromatography on silica eluted with 0-20% EtOAc in hexane to give (*R*)-3-hydroxy-3-(2-methoxyphenyl)-1-(2,3,4,5,6-pentamethylphenyl)propan-1-one **16b** as a white solid (60.0 mg, 0.184 mmol, 75%). The reaction was also followed by HPLC (Chiralpak IC, 30 cm x 6 mm column, hexane:iPrOH 90:10, 1.0 mL/min, T = 25°C): 100% conversion; $[\alpha]_D^{26}$ +37 (c 0.350 in $CHCl_3$) 94% ee (*R*).

^{1}H NMR (400 MHz, $CDCl_3$) of *(R)*-3-hydroxy-3-(2-methoxyphenyl)-1-(2,3,4,5,6-pentamethylphenyl)propan-1-one **16b**.

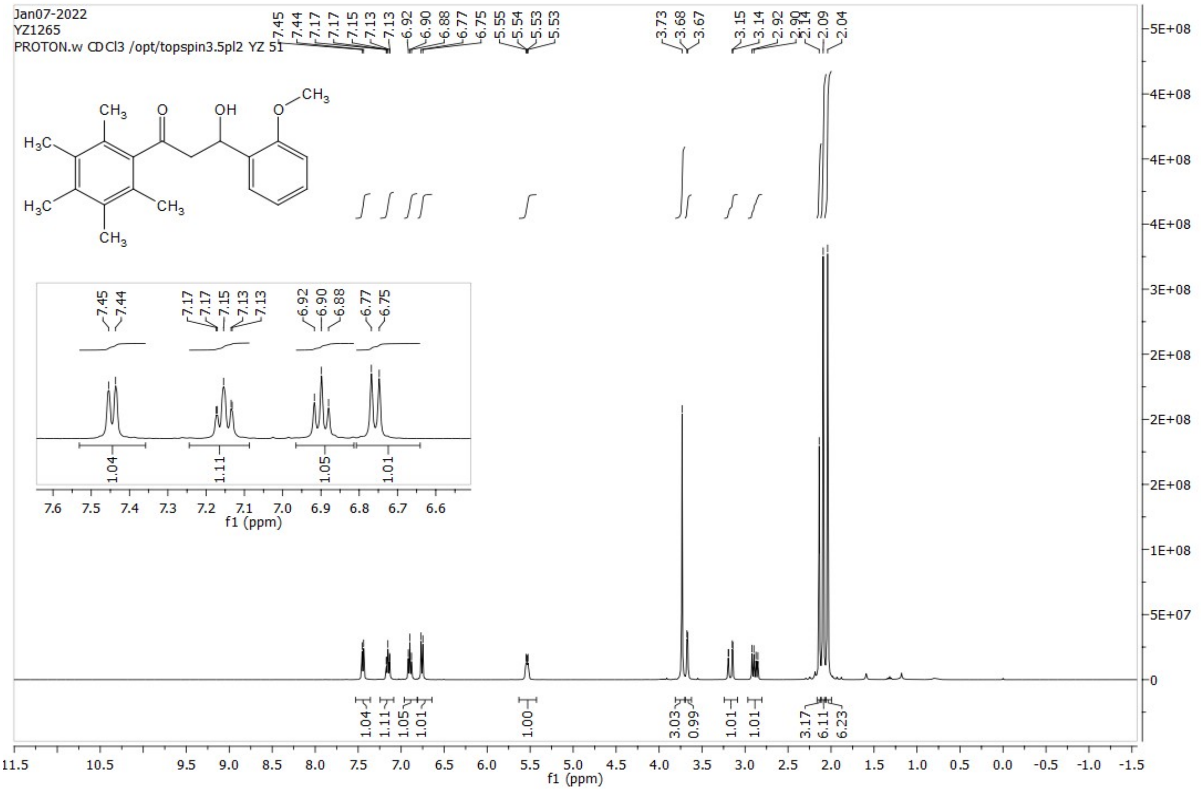

$^{13}C\{^{1}H\}$ NMR (100 MHz, $CDCl_3$) of *(R)*-3-hydroxy-3-(2-methoxyphenyl)-1-(2,3,4,5,6-pentamethylphenyl)propan-1-one **16b**.

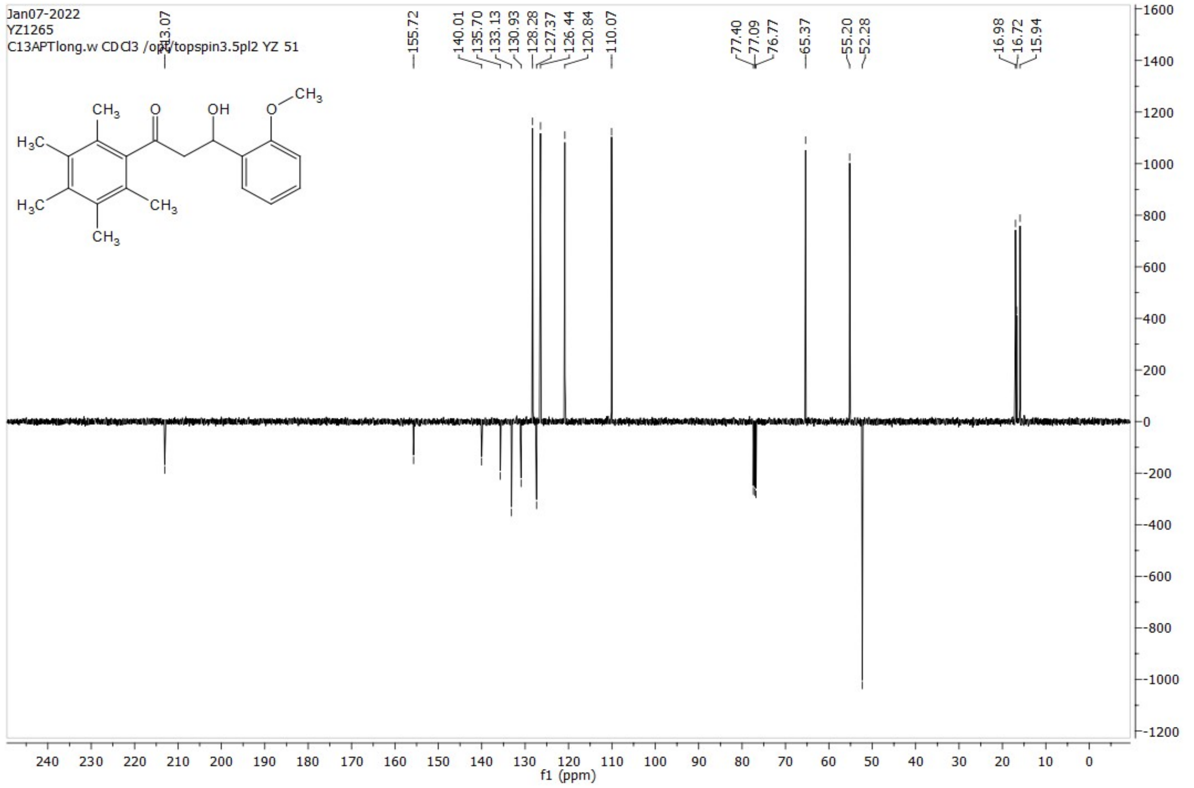

COSY (400 MHz, $CDCl_3$) of (*R*)-3-hydroxy-3-(2-methoxyphenyl)-1-(2,3,4,5,6-pentamethylphenyl)propan-1-one **16b**.

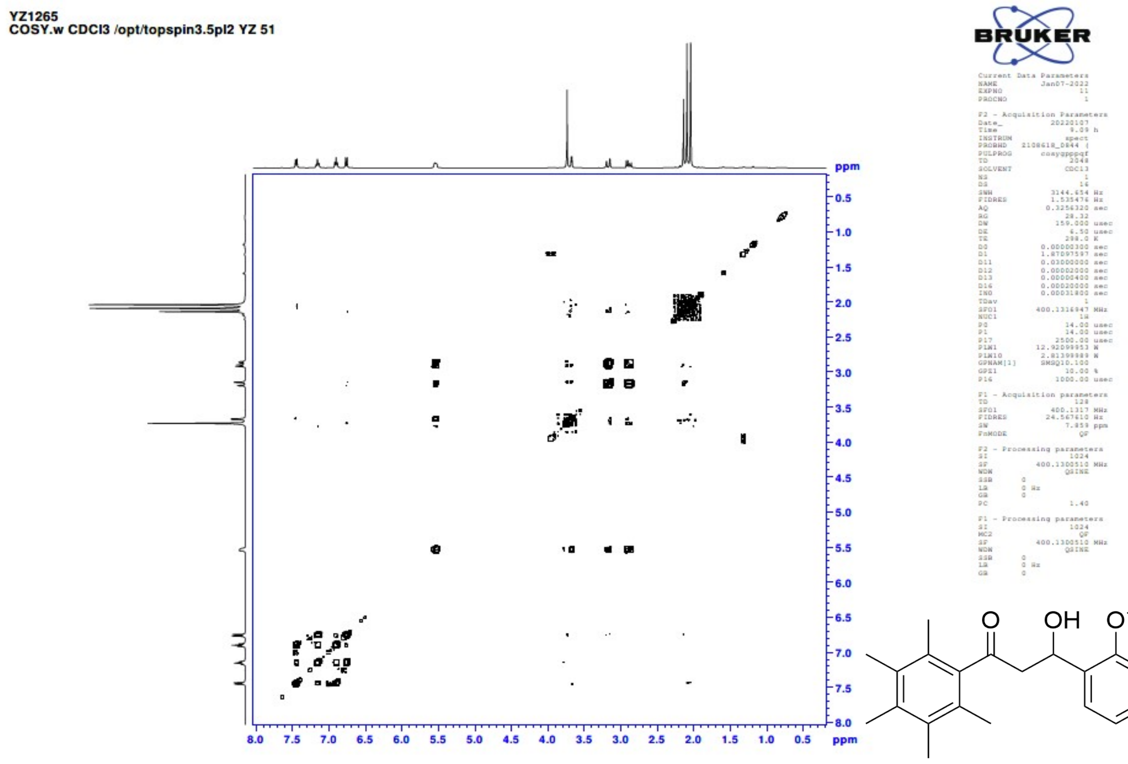

HSQC (400 MHz, $CDCl_3$) of (*R*)-3-hydroxy-3-(2-methoxyphenyl)-1-(2,3,4,5,6-pentamethylphenyl)propan-1-one **16b**.

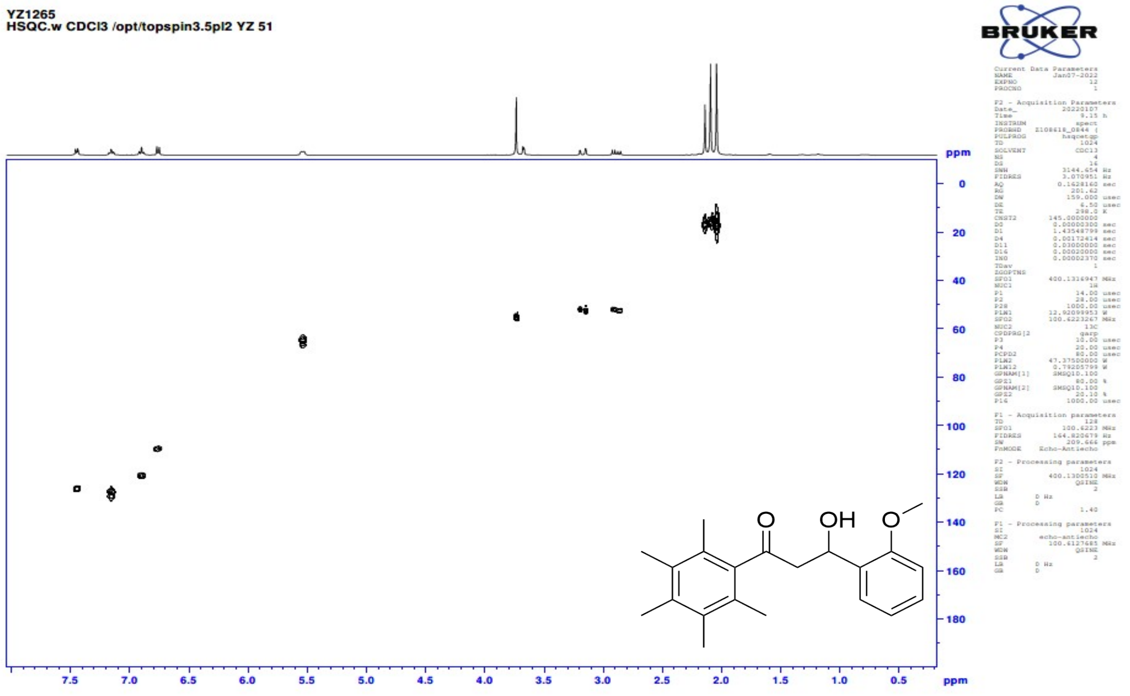

HMBC (400 MHz, $CDCl_3$) of (*R*)-3-hydroxy-3-(2-methoxyphenyl)-1-(2,3,4,5,6-pentamethylphenyl)propan-1-one **16b**.

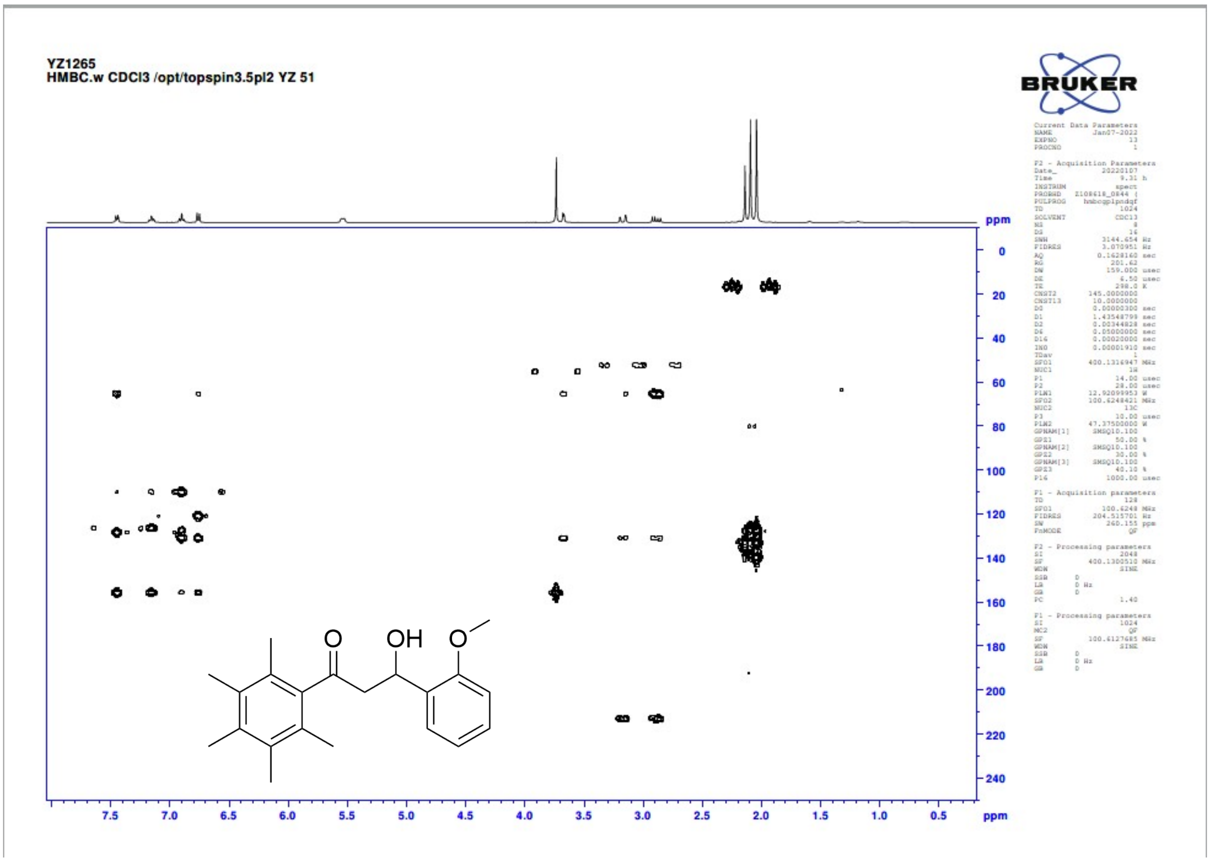

HPLC of racemic 3-hydroxy-3-(2-methoxyphenyl)-1-(2,3,4,5,6-pentamethylphenyl)propan-1-one **16b**.

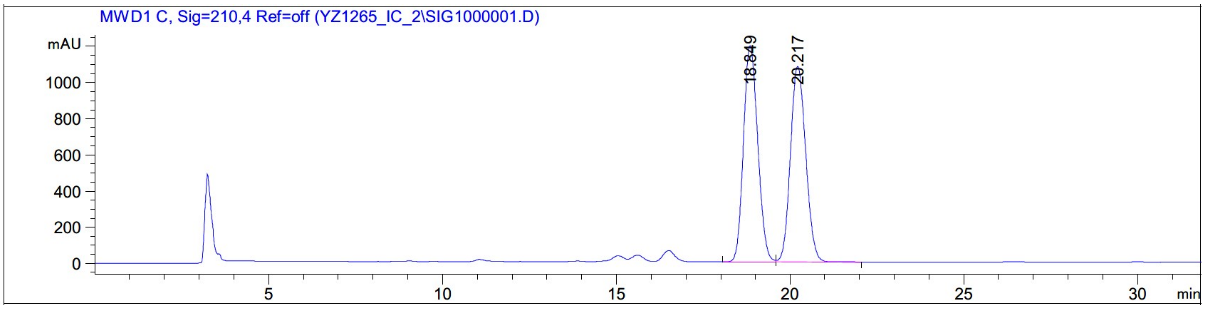

Signal 2: MWD1 C, Sig=210,4 Ref=off

Peak #	RetTime [min]	Type	Width [min]	Area [mAU*s]	Height [mAU]	Area %
1	18.849	BV	0.4492	3.41184e4	1194.59412	50.7774
2	20.217	VB	0.4783	3.30737e4	1082.84473	49.2226

Totals : 6.71921e4 2277.43884

HPLC of (*R*)-3-hydroxy-3-(2-methoxyphenyl)-1-(2,3,4,5,6-pentamethylphenyl)propan-1-one **16b**. (*R,R*)-3C-Tethered Ru(II)-TsDPEN catalyst (after 24 h, 100% conversion, 94% ee (*R*)).

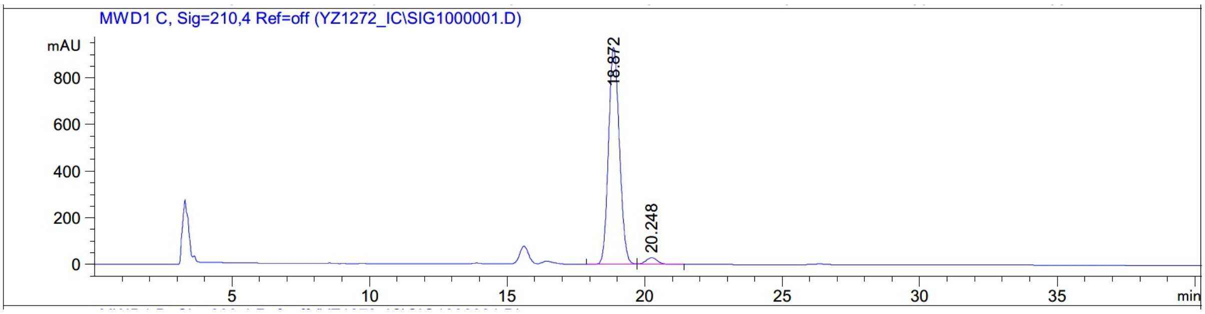

Signal 2: MWD1 C, Sig=210,4 Ref=off

Peak #	RetTime [min]	Type	Width [min]	Area [mAU*s]	Height [mAU]	Area %
1	18.872	BV	0.4297	2.56353e4	929.69446	96.8011
2	20.248	VB	0.4624	847.15283	28.37109	3.1989

Totals :				2.64825e4	958.06554	

1-(Furan-2-yl)-3-(2,3,4,5,6-pentamethylphenyl)propane-1,3-dione 17a.

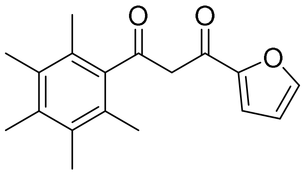

This compound is novel.

To a solution of sodium hydride (300 mg, 60% dispersion in mineral oil, 7.50 mmol) in THF (2 mL) at 0 °C was added dropwise a solution of 1-(2,3,4,5,6-pentamethylphenyl)ethan-1-one **6** (285 mg, 1.50 mmol) in THF (2 mL). The reaction mixture was stirred under a nitrogen atmosphere at 0 °C for 30 min and then stirred under a nitrogen atmosphere at rt for 30 min, after which a solution of ethyl 2-furoate (525 mg, 3.75 mmol) in THF (2 mL) was added dropwise. The reaction mixture was heated to 65 °C and left stirring under the nitrogen atmosphere overnight. The reaction was followed by TLC (9:1 hexane: EtOAc). The mixture was quenched by 2M HCl solution (20 mL). EtOAc (20 mL) was added and the organic layer was separated. The aqueous layer was extracted with EtOAc (3 × 20 mL), and the combined organic layers were washed with saturated $NaHCO_3$ solution (2 × 20 mL) and brine (20 mL), dried ($MgSO_4$) and filtered. Solvent was removed to give the crude product. The product was isolated via flash chromatography on silica eluted with 4% EtOAc in hexane to give 1-(furan-2-yl)-3-(2,3,4,5,6-pentamethylphenyl)propane-1,3-dione **17a** as a white solid (284 mg, 1.00 mmol, 67%). TLC: Rf ca 0.80 (9:1 hexane: EtOAc), strong UV and $KMnO_4$; Mp: 127 °C; HRMS (ESI+) *m/z*: [M+H]+ Calcd for $C_{18}H_{20}NaO_3$ 307.1301; Found 307.1305; 1.3 ppm error; ν_{max} 3127, 2911, 1597, 1577, 1464, 1381, 1254, 1080, 1028, 931, 758 cm^{-1}; enol: keto = 100:0; ^1H NMR (400 MHz, $CDCl_3$): δ 15.72 (1H, s, OH), 7.59 (1H, s, H of furan), 7.21 (1H, d, *J* = 3.5, H of furan), 6.57 (1H, dd, *J* = 3.5, 1.3, H of furan), 6.20 (1H, s, CH of enol form), 2.28-2.27 (9H, m, CH_3), 2.24 (6H, s, CH_3); $^{13}C\{^1H\}$ NMR (100 MHz, $CDCl_3$): δ 188.4 (C), 177.8 (C), 151.0 (C), 146.3 (CH), 136.2 (C), 134.9 (C), 136.2 (C), 129.9 (C), 116.1 (CH), 112.6 (CH), 99.3 (CH of enol form), 17.6 (CH_3), 16.9 (CH_3), 16.2 (CH_3); *m/z* (ES-API+) 307.1 (M^+ + 23, 100%).

Enantiomeric excess and conversion determined by HPLC analysis (Chiralcel OJ, 30 cm x 6 mm column, hexane:iPrOH 90:10, 1.0 mL/min, T = 25°C) ketone 8.8 min, *R* and *S* isomers 8.8 min and 11.3 min, configuration assigned by analogy.

^{1}H NMR (400 MHz, $CDCl_3$) of 1-(furan-2-yl)-3-(2,3,4,5,6-pentamethylphenyl)propane-1,3-dione **17a**.

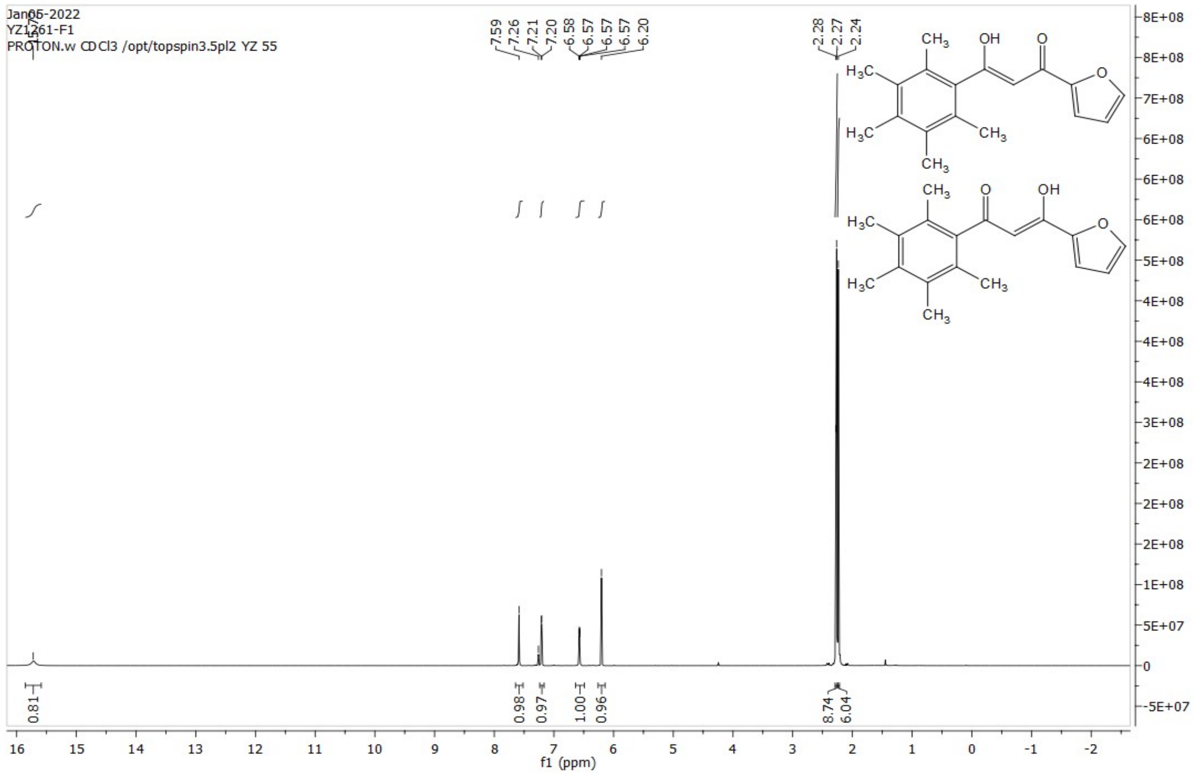

^{13}C{^{1}H} NMR (100 MHz, $CDCl_3$) of 1-(furan-2-yl)-3-(2,3,4,5,6-pentamethylphenyl)propane-1,3-dione **17a**.

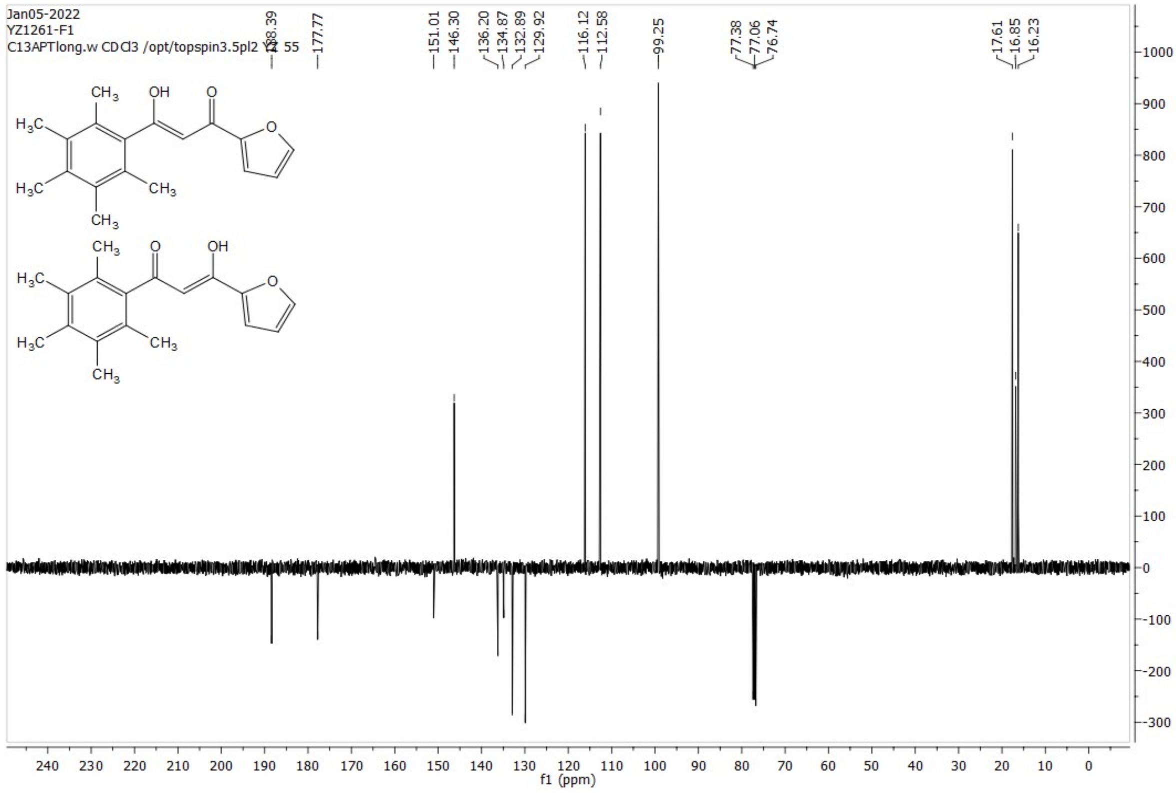

COSY (400 MHz, $CDCl_3$) of 1-(furan-2-yl)-3-(2,3,4,5,6-pentamethylphenyl)propane-1,3-dione **17a**.

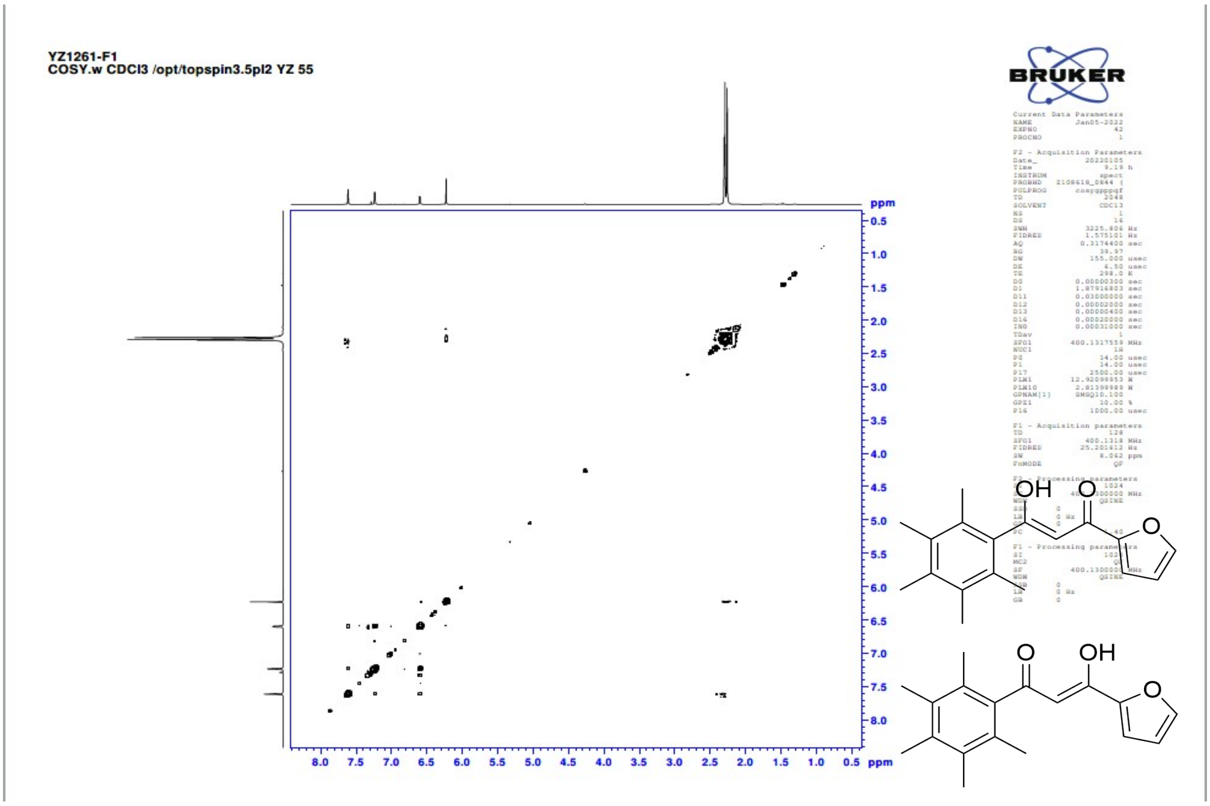

HSQC (400 MHz, $CDCl_3$) of 1-(furan-2-yl)-3-(2,3,4,5,6-pentamethylphenyl)propane-1,3-dione **17a**.

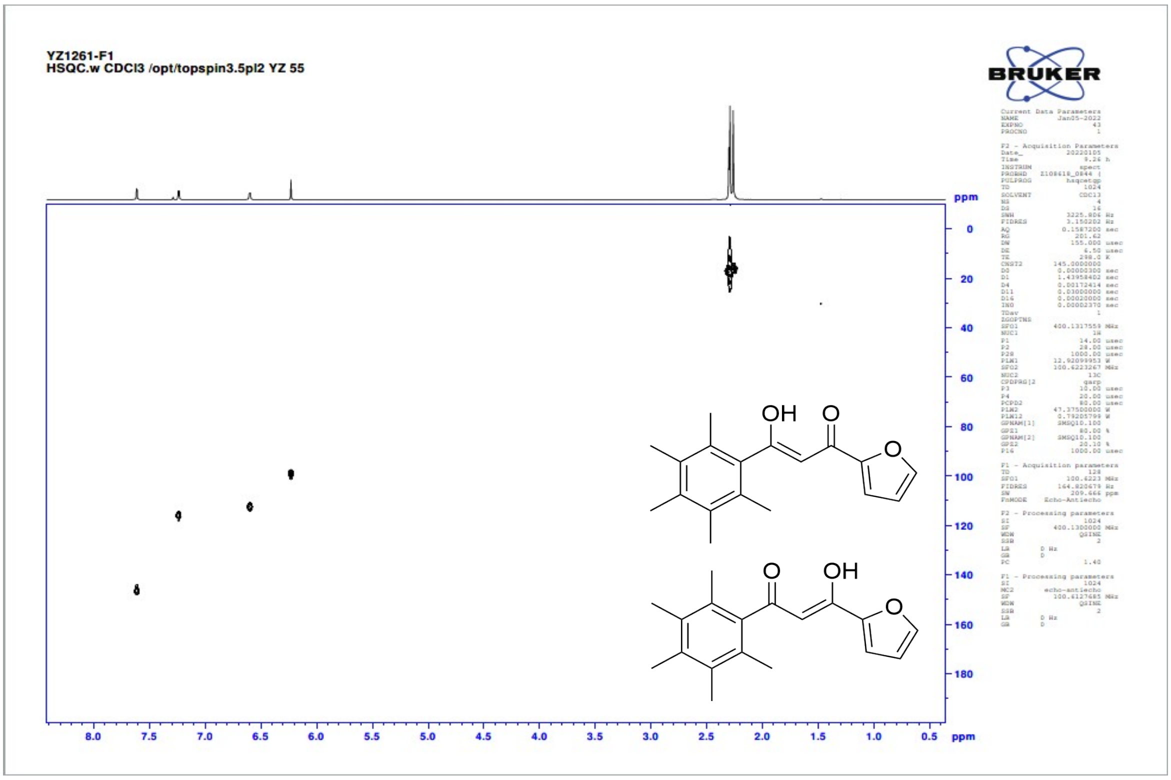

HMBC (400 MHz, $CDCl_3$) of 1-(furan-2-yl)-3-(2,3,4,5,6-pentamethylphenyl)propane-1,3-dione **17a**.

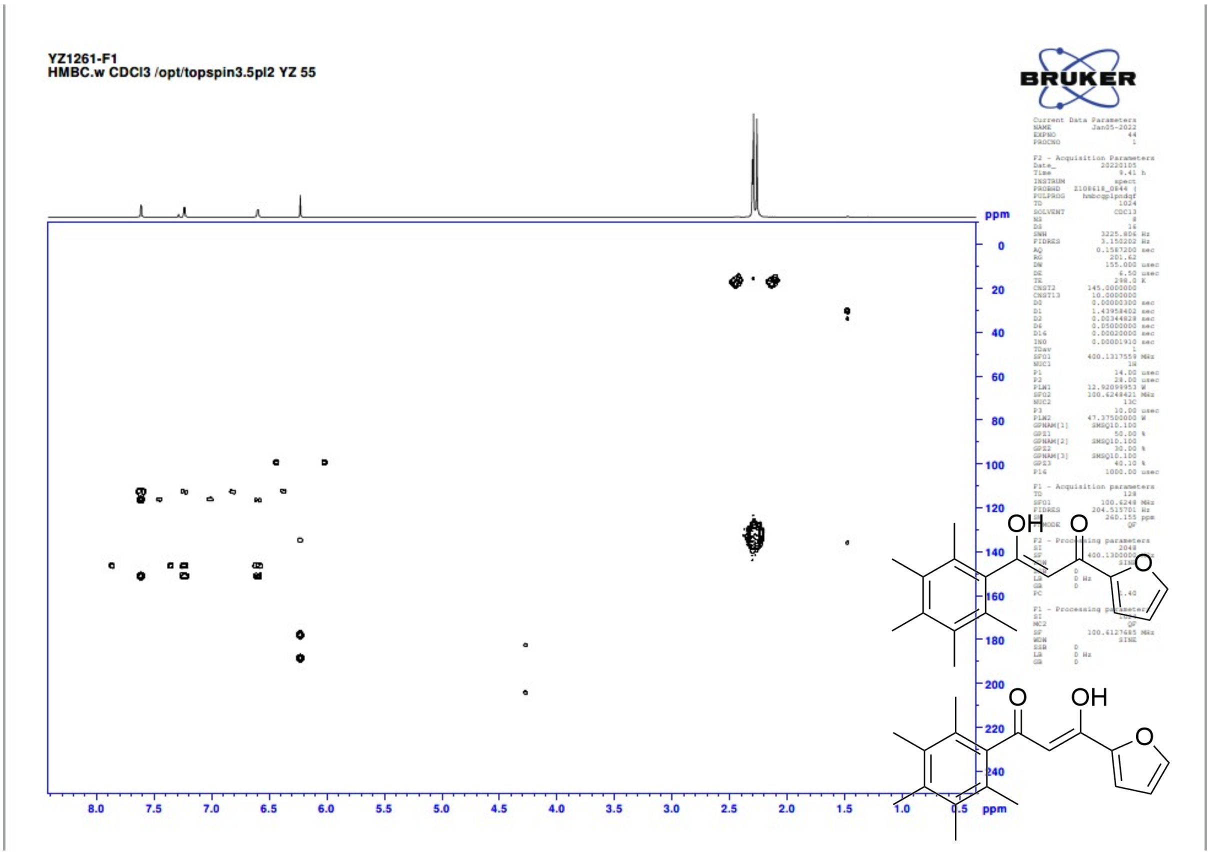

HPLC of 1-(furan-2-yl)-3-(2,3,4,5,6-pentamethylphenyl)propane-1,3-dione **17a.**

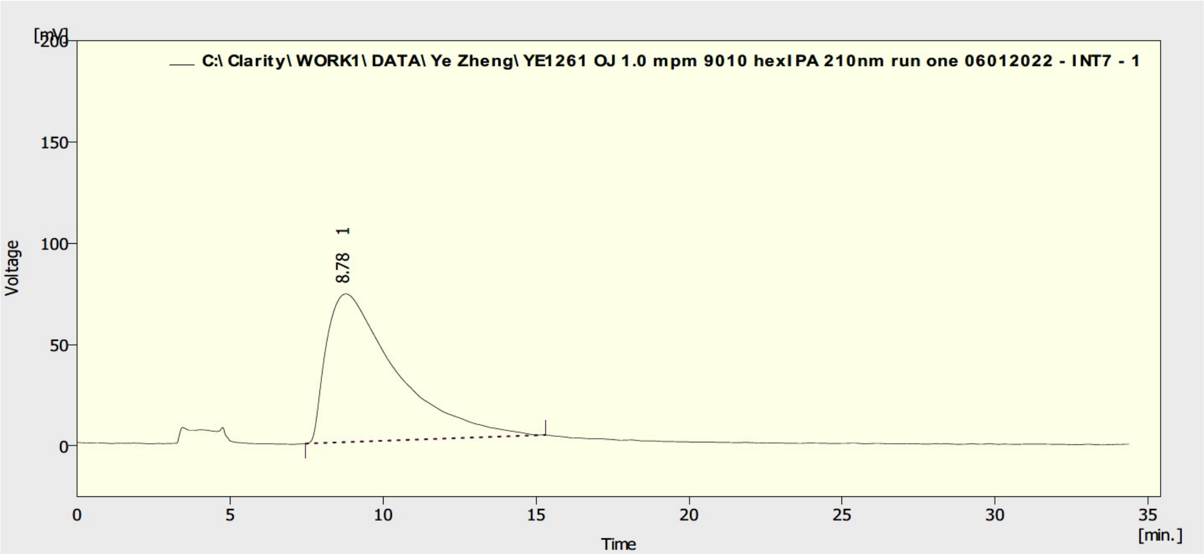

Result Table (Uncal - C:\Clarity\WORK1\DATA\Ye Zheng\YE1261 OJ 1.0 mpm 9010 hexIPA 210nm run one 06012022 - INT7 - 1)

	Reten. Time [min]	Area [mV.s]	Height [mV]	Area [%]	Height [%]	W05 [min]	Compound Name
1	8.780	11634.569	73.133	100.0	100.0	2.32	
	Total	11634.569	73.133	100.0	100.0		

3-(Furan-2-yl)-3-hydroxy-1-(2,3,4,5,6-pentamethylphenyl)propan-1-one 17b.

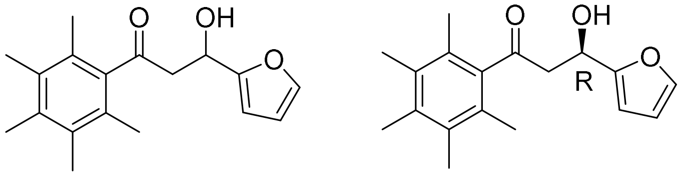

This compound is novel.

Synthesis of a racemic standard: (*R,R*)-3C-Tethered Ru(II)-TsDPEN catalyst (0.9 mg, 1.4 mmol, 0.5 mol%) and (*S,S*)-3C-tethered Ru(II)-TsDPEN catalyst (0.9 mg, 1.4 mmol, 0.5 mol%) were added to FA: TEA (5:2 azeotropic mixture, 0.36 mL) at rt and the mixture was stirred under a nitrogen atmosphere for 15 minutes; after which a solution of 1-(furan-2-yl)-3-(2,3,4,5,6-pentamethylphenyl)propane-1,3-dione **17a** (80.0 mg, 0.282 mmol) in DCM (0.50 mL) was added. The reaction mixture was stirred under a nitrogen atmosphere and followed by TLC (9:1 hexane: EtOAc). After 24 h, the reaction was quenched using saturated $NaHCO_3$ solution (20 mL). EtOAc (20 mL) was added and the organic layer was separated. The aqueous layer was extracted with EtOAc (3 x 20 mL) and the combined organic layers were washed with saturated $NaHCO_3$ solution (2 × 20 mL), and brine (20 mL), dried ($MgSO_4$) and filtered. The solvent was removed to give the crude product. The product was isolated via flash chromatography on silica eluted with 0-20% EtOAc in hexane to give 3-(furan-2-yl)-3-hydroxy-1-(2,3,4,5,6-pentamethylphenyl)propan-1-one **17b** as a white solid (56.0 mg, 0.196 mmol, 70%). TLC: Rf ca 0.20 (9:1 hexane: EtOAc), strong UV and $KMnO_4$; Mp: 113 °C; HRMS (ESI+) *m/z*: [M+H]+ Calcd for $C_{18}H_{22}NaO_3$ 309,1456; Found 309.1461; 1.6 ppm error; ν_{max} 3247 (br), 2920, 1700, 1381, 1357, 1084, 1065, 1004, 933, 924, 730 cm^{-1}; ^{1}H NMR (400 MHz, $CDCl_3$): δ 7.38 (1H, s, H of furan), 6.36-6.31 (2H, m, H of furan), 5.41-5.37 (1H, m, ArCH), 3.53 (1H, d, *J* = 4.3, OH), 3.32 (1H, dd, *J* = 18.8, 8.9, CH_2), 3.20 (1H, dd, *J* = 18.8, 3.1, CH_2), 2.25 (3H, s, CH_3), 2.20 (6H, s, CH_3), 2.13 (6H, s, CH_3); $^{13}C\{^1H\}$ NMR (100 MHz, $CDCl_3$): δ 211.6 (C), 154.9 (C), 142.2 (CH), 139.6 (C), 136.0 (C), 133.3 (C), 127.4 (C), 110.3 (CH), 106.5 (CH), 63.6 (CH), 50.2 (CH_2), 17.1 (CH_3), 16.7 (CH_3), 15.9 (CH_3); *m/z* (ES-API+) 309.1 (M^+ + 23, 100%).

Enantiomeric excess and conversion determined by HPLC analysis (Chiralcel OJ, 30 cm x 6 mm column, hexane:iPrOH 90:10, 1.0 mL/min, T = 25°C) ketone 8.8 min, *R* and *S* isomers 8.8 min and 11.3 min, configuration assigned by analogy.

(*R*)-3-(Furan-2-yl)-3-hydroxy-1-(2,3,4,5,6-pentamethylphenyl)propan-1-one **17b..** (*R,R*)-3C-tethered Ru(II)-TsDPEN catalyst (1.8 mg, 2.8 mmol, 1 mol%) was added to FA: TEA (5:2 azeotropic mixture, 0.36 mL) at rt and the mixture was stirred under a nitrogen atmosphere for 15 minutes; after which a solution of 1-(furan-2-yl)-3-(2,3,4,5,6-pentamethylphenyl)propane-1,3-dione **17a** (80.0 mg, 0.282 mmol) in DCM (0.50 mL) was added. The reaction mixture was stirred under a nitrogen atmosphere and followed by TLC (9:1 hexane: EtOAc). After 24 h, the reaction was quenched using saturated $NaHCO_3$ solution (20 mL). EtOAc (20 mL) was added and the organic layer was separated. The aqueous layer was extracted with EtOAc (3 x 20 mL) and the combined organic layers were dried ($MgSO_4$) and filtered. The solvent was removed to give the crude product. The product was isolated via flash chromatography on silica eluted with 0-20% EtOAc in hexane to give (*R*)-3-(furan-2-yl)-3-hydroxy-1-(2,3,4,5,6-pentamethylphenyl)propan-1-one **17b** as a white solid (60.0 mg, 0.210 mmol, 74%). The reaction was also followed by HPLC (Chiralcel OJ, 30 cm x 6 mm column, hexane:iPrOH 90:10, 1.0 mL/min, T = 25°C): 100% conversion; $[\alpha]_D^{25}$ +23.6 (c 0.250 in $CHCl_3$) 98% ee (*R*).

^{1}H NMR (400 MHz, $CDCl_3$) of 3-(furan-2-yl)-3-hydroxy-1-(2,3,4,5,6-pentamethylphenyl)propan-1-one **17b**.

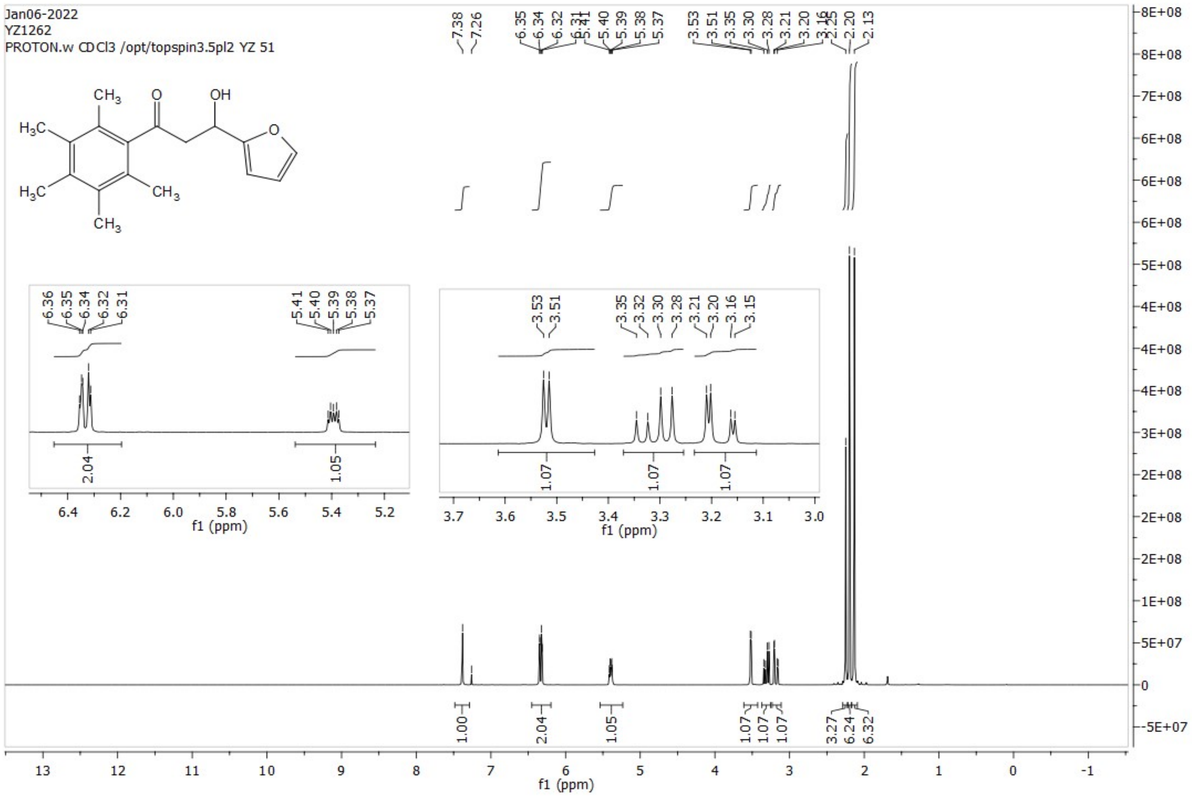

$^{13}C\{^{1}H\}$ NMR (100 MHz, $CDCl_3$) of 3-(furan-2-yl)-3-hydroxy-1-(2,3,4,5,6-pentamethylphenyl)propan-1-one **17b**.

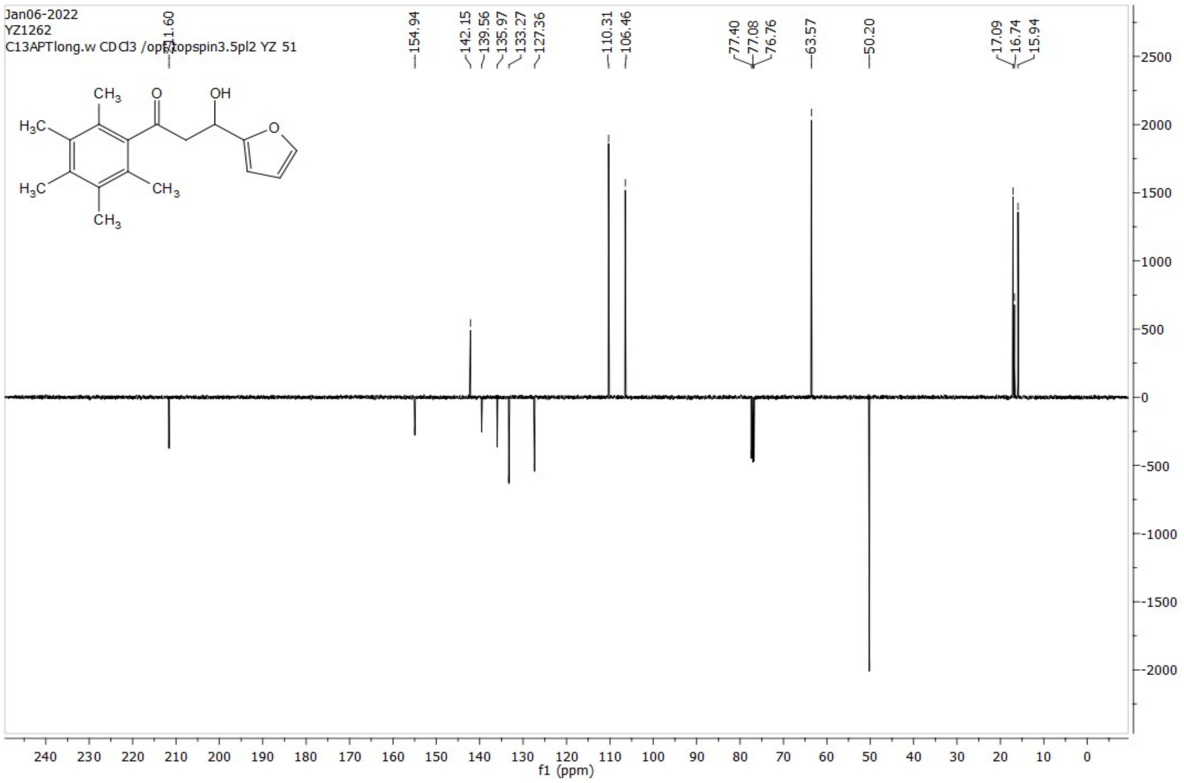

COSY (400 MHz, $CDCl_3$) of 3-(furan-2-yl)-3-hydroxy-1-(2,3,4,5,6-pentamethylphenyl)propan-1-one **17b**.

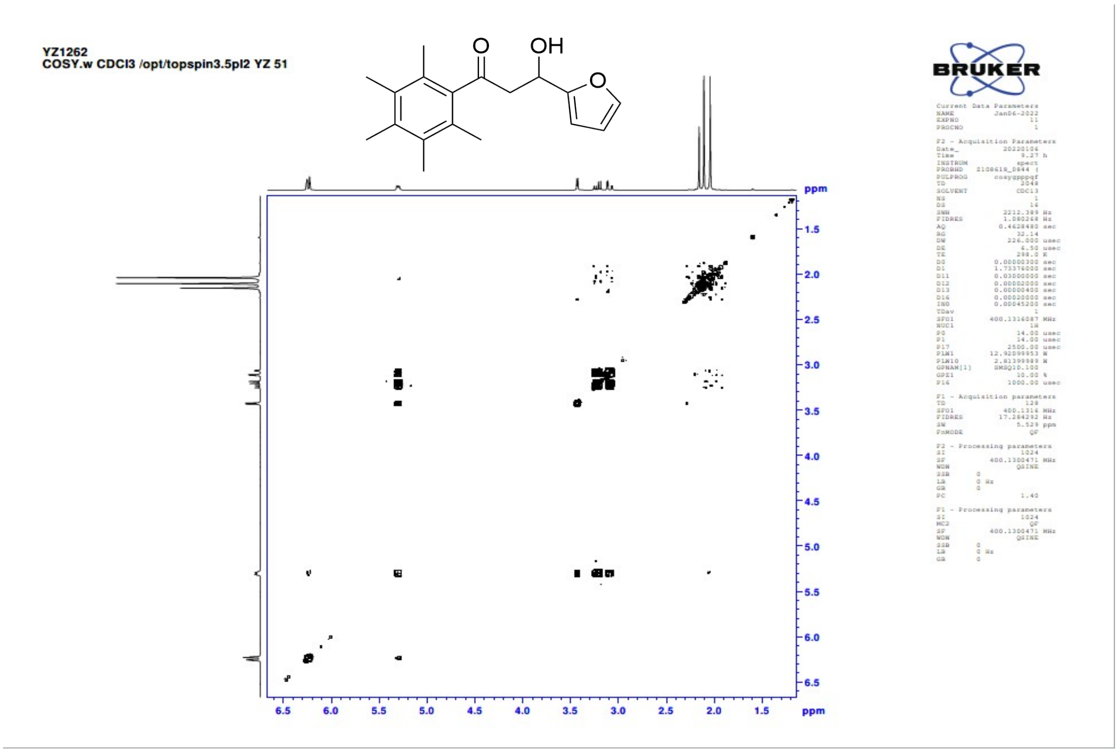

HSQC (400 MHz, $CDCl_3$) of 3-(furan-2-yl)-3-hydroxy-1-(2,3,4,5,6-pentamethylphenyl)propan-1-one **17b**.

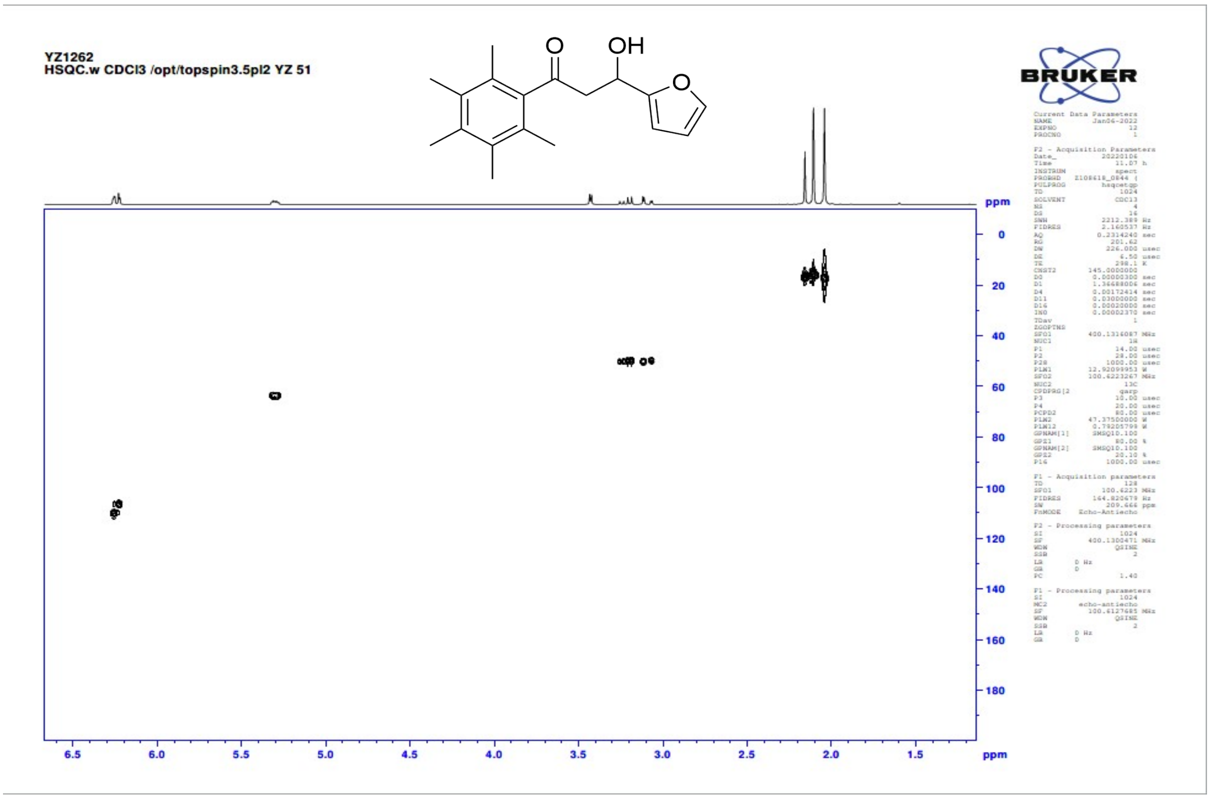

HMBC (400 MHz, $CDCl_3$) of 3-(furan-2-yl)-3-hydroxy-1-(2,3,4,5,6-pentamethylphenyl)propan-1-one **17b**.

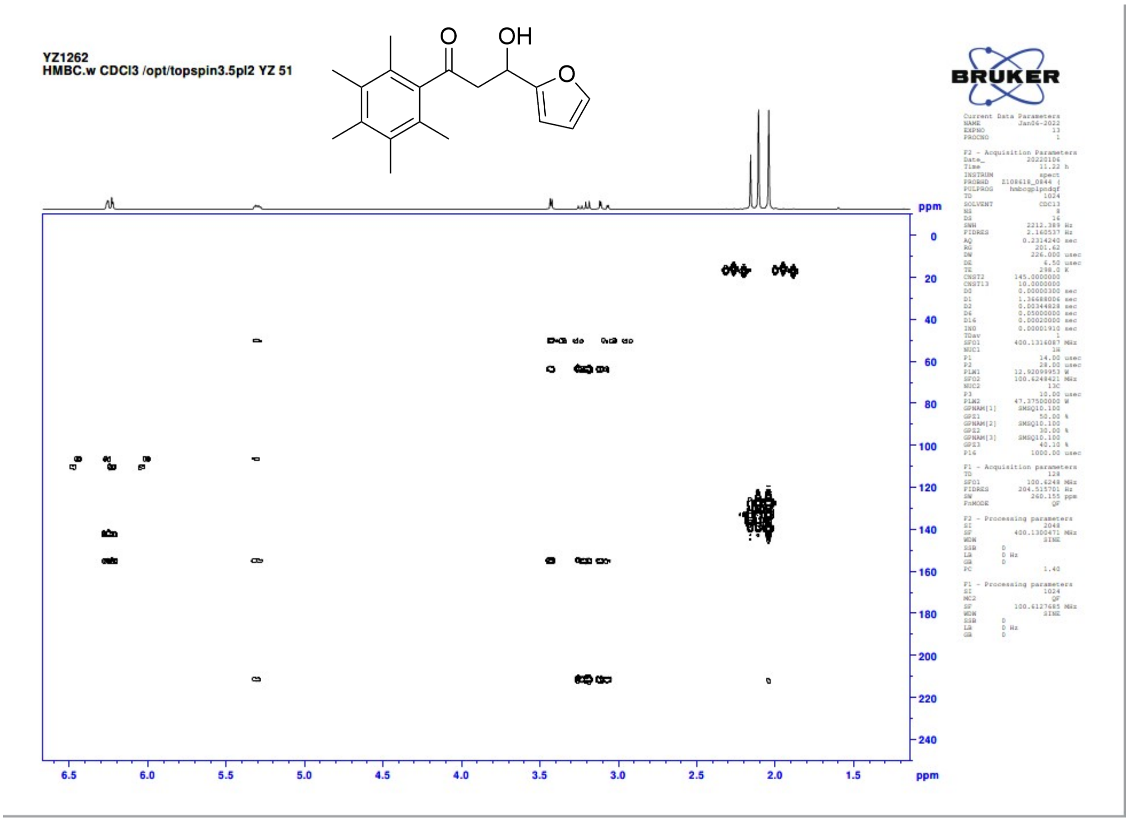

HPLC of racemic 3-(furan-2-yl)-3-hydroxy-1-(2,3,4,5,6-pentamethylphenyl)propan-1-one **17b.**

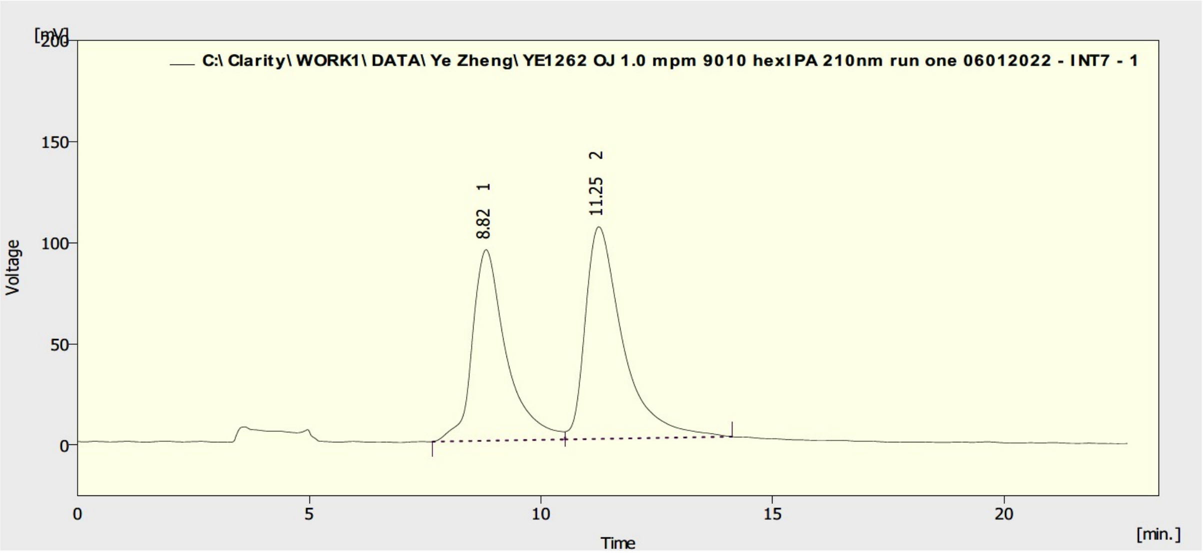

Result Table (Uncal - C:\Clarity\WORK1\DATA\Ye Zheng\YE1262 OJ 1.0 mpm 9010 hexIPA 210nm run one 06012022 - INT7 - 1)

	Reten. Time [min]	Area [mV.s]	Height [mV]	Area [%]	Height [%]	W05 [min]	Compound Name
1	8.820	4829.686	94.420	44.9	47.4	0.71	
2	11.253	5929.440	104.831	55.1	52.6	0.80	
	Total	10759.126	199.251	100.0	100.0		

HPLC of 3-(furan-2-yl)-3-hydroxy-1-(2,3,4,5,6-pentamethylphenyl)propan-1-one **17b**.. (*R,R*)-3C-Tethered Ru(II)-TsDPEN catalyst (after 24 h, 100% conversion, 98% ee (*R*))

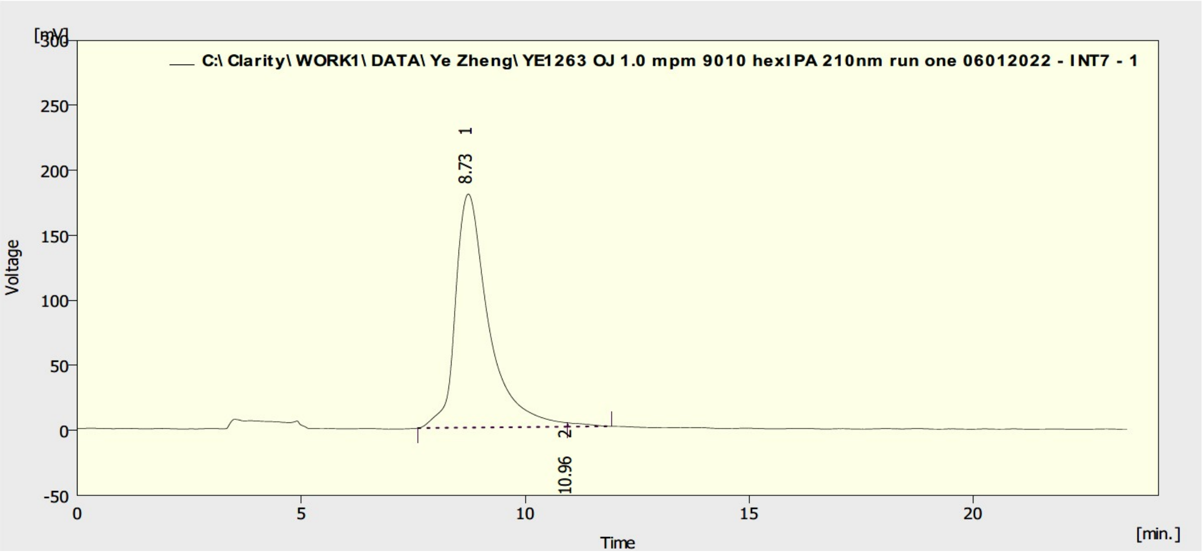

Result Table (Uncal - C:\Clarity\WORK1\DATA\Ye Zheng\YE1263 OJ 1.0 mpm 9010 hexIPA 210nm run one 06012022 - INT7 - 1)

	Reten. Time [min]	Area [mV.s]	Height [mV]	Area [%]	Height [%]	W05 [min]	Compound Name
1	8.730	9309.279	179.716	99.1	98.4	0.71	
2	10.957	82.227	2.923	0.9	1.6	0.53	
	Total	9391.506	182.638	100.0	100.0		

Procedures for 3-hydroxy-3-aryl-1-(2,3,5,6-tetramethylphenyl)propan-1-ones 18b-24b.

1-Phenyl-3-(2,3,5,6-tetramethylphenyl)propane-1,3-dione 18a.

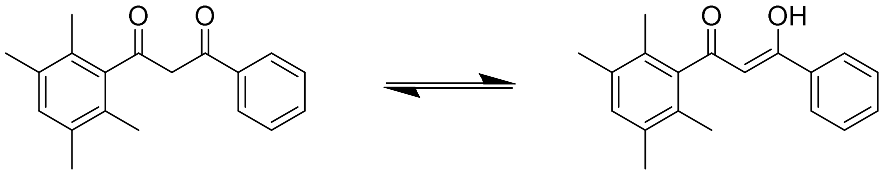

This compound is novel.

To a solution of sodium hydride (227 mg, 60% dispersion in mineral oil, 5.7 mmol) in THF (3 mL) at 0 °C was added dropwise a solution of 1-(2,3,5,6-tetramethylphenyl) ethan-1-one **7** (200 mg, 1.13 mmol) in THF (2 mL). The reaction mixture was stirred under a nitrogen atmosphere at 0 °C for 30 min and then stirred under a nitrogen atmosphere at rt for 30 min, after which ethyl benzoate (836 mg, 5.7 mmol) was added dropwise at 0°C. The reaction mixture was refluxed at 66 °C and left stirring under the nitrogen atmosphere overnight. The reaction was followed by TLC (4:1 hexane: EtOAc). The mixture was quenched by 2M HCl solution (20 mL). EtOAc (20 mL) was added, and the organic layer was separated. The aqueous layer was extracted with EtOAc (3 × 20 mL), and the combined organic layers were washed with saturated $NaHCO_3$ solution (2 × 20 mL) and brine (20 mL), dried ($MgSO_4$) and filtered. Solvent was removed to give the crude product. The product was isolated via flash chromatography on silica eluted with 1% EtOAc in hexane to give 1-phenyl-3-(2,3,5,6-tetramethylphenyl)propane-1,3-dione **18a** as a white solid (73.8 mg, 2.50 mmol, 23%). TLC: Rf ca 0.46 (9:1 hexane: EtOAc), strong UV and $KMnO_4$; Mp: 93.8 °C; HRMS: (found (ESI+): [M+Na]+, Calcd for $C_{19}H_{20}NaO_2$ 303.1356; Found 303.1346; 3.1 ppm error; ν_{max} 2941, 2925, 1596, 1561, 1468 cm^{-1}; enol: keto = 49:1; ^1H NMR (500 MHz, $CDCl_3$): δ 7.96 (2H, d, J = 7.8, CH of Ar), 7.58 (1H, t, J = 7.3, CH of Ar), 7.50 (2H, t, J = 7.5, CH of Ar), 7.05 (1H, s, CH of Ar), 6.35 (1H, s, CH of enol form), 4.43 (2H, s, CH_2 of keto form), 2.40 (6H, s, CH_3 of keto form), 2.36 (6H, s, CH_3 of keto form), 2.28 (6H, s, CH_3 of enol form), 2.24 (6H, s, CH_3 of enol form) (due to the predominance of the enol form, fractional integrals are not listed); $^{13}C\{^1H\}$ NMR (126 MHz, $CDCl_3$): δ 192.9 (C), 185.0 (C), 138.2 (C), 135.2 (C), 134.2 (C), 132.6 (CH), 132.1 (CH), 130.1 (C), 128.7 (CH), 127.3 (CH), 99.3 (CH), 55.4 (CH_2), 19.7 (CH_3), 16.4 (CH_3); *m/z* (ES-API+) 303.1 (M^+ + Na, 100%); Enantiomeric excess

and conversion determined by HPLC analysis (Chiralpak OJ, 30 cm x 6 mm column, hexane:iPrOH 90:10, 1.0 mL/min, T = 25°C) ketone 6.2 min, *R* and *S* isomer 8.3 min and 11.2 min. Configuration assigned by analogy.

^{1}H NMR (500 MHz, $CDCl_3$) of 1-phenyl-3-(2,3,5,6-tetramethylphenyl)propane-1,3-dione **18a**.

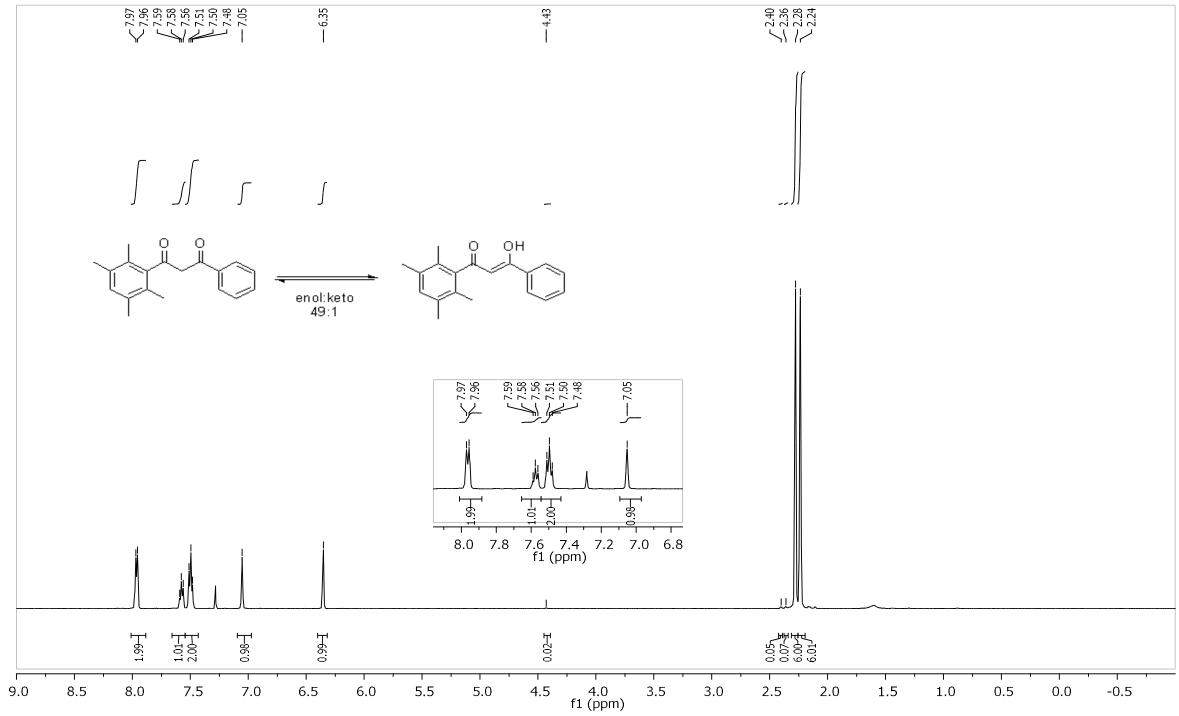

COSY (500 MHz, $CDCl_3$) of 1-phenyl-3-(2,3,5,6-tetramethylphenyl)propane-1,3-dione **18a**.

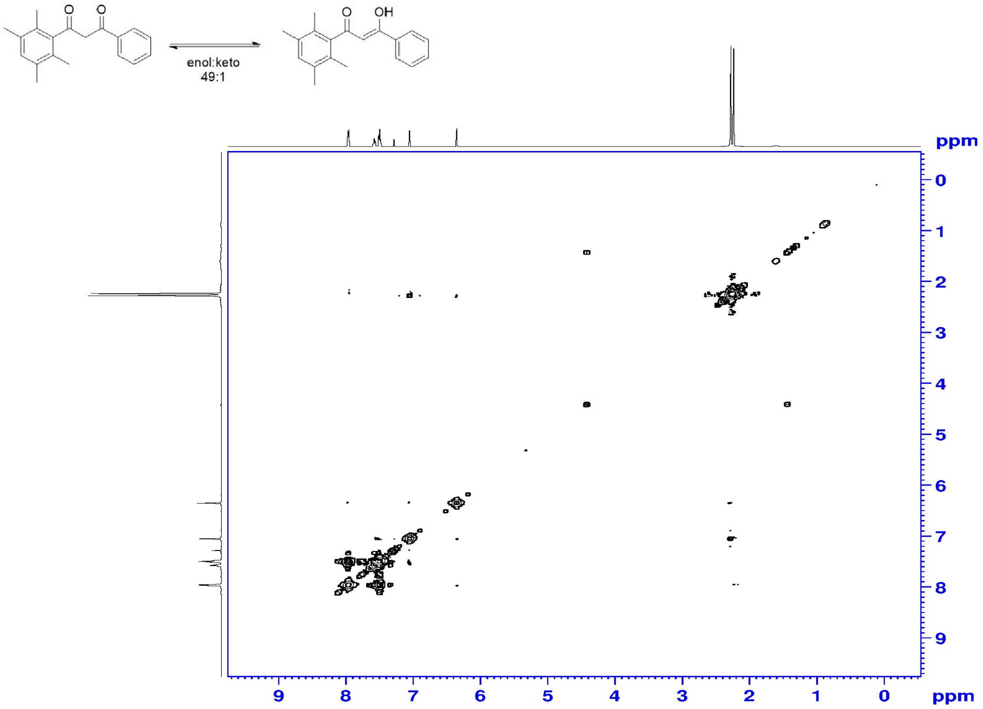

HSQC (126 MHz, $CDCl_3$) of 1-phenyl-3-(2,3,5,6-tetramethylphenyl)propane-1,3-dione **18a**.

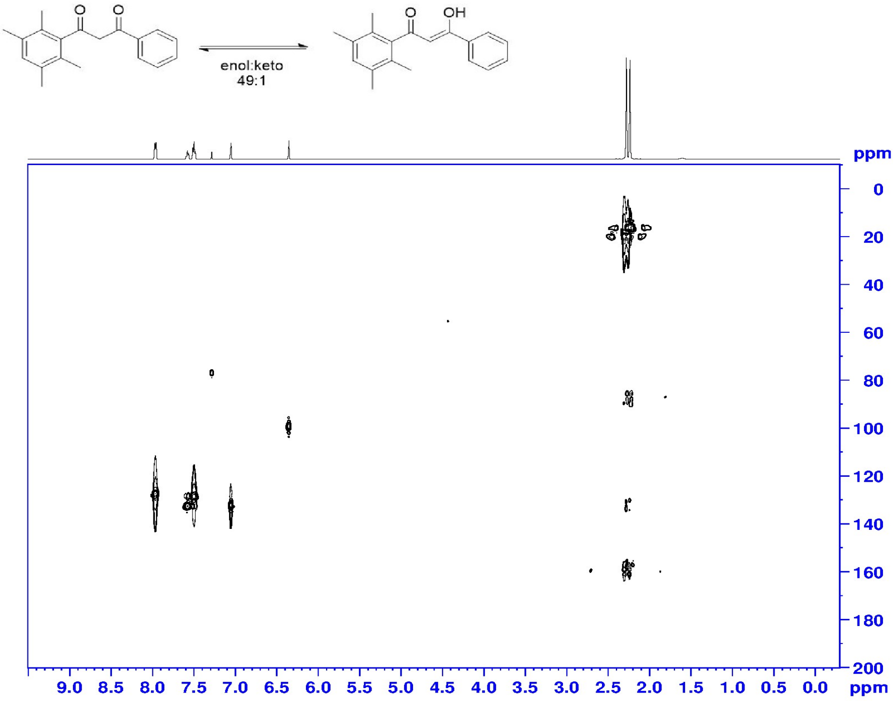

HMBC (126 MHz, $CDCl_3$) of 1-phenyl-3-(2,3,5,6-tetramethylphenyl)propane-1,3-dione **18a**.

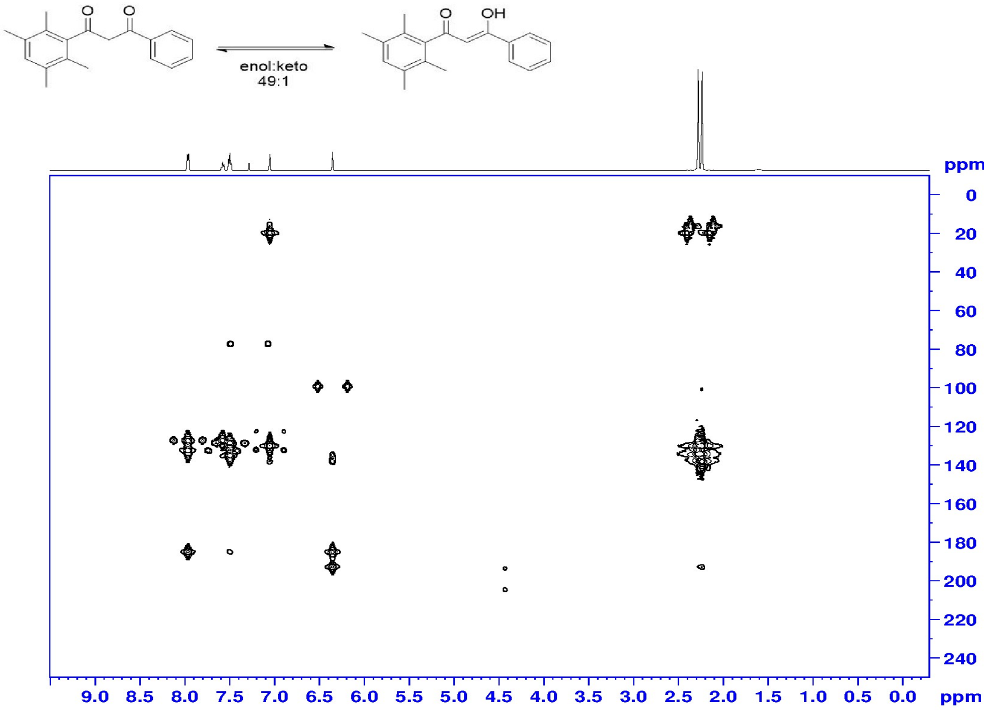

$^{13}C\{^1H\}$ NMR (126 MHz, $CDCl_3$) of 1-phenyl-3-(2,3,5,6-tetramethylphenyl)propane-1,3-dione **18a**.

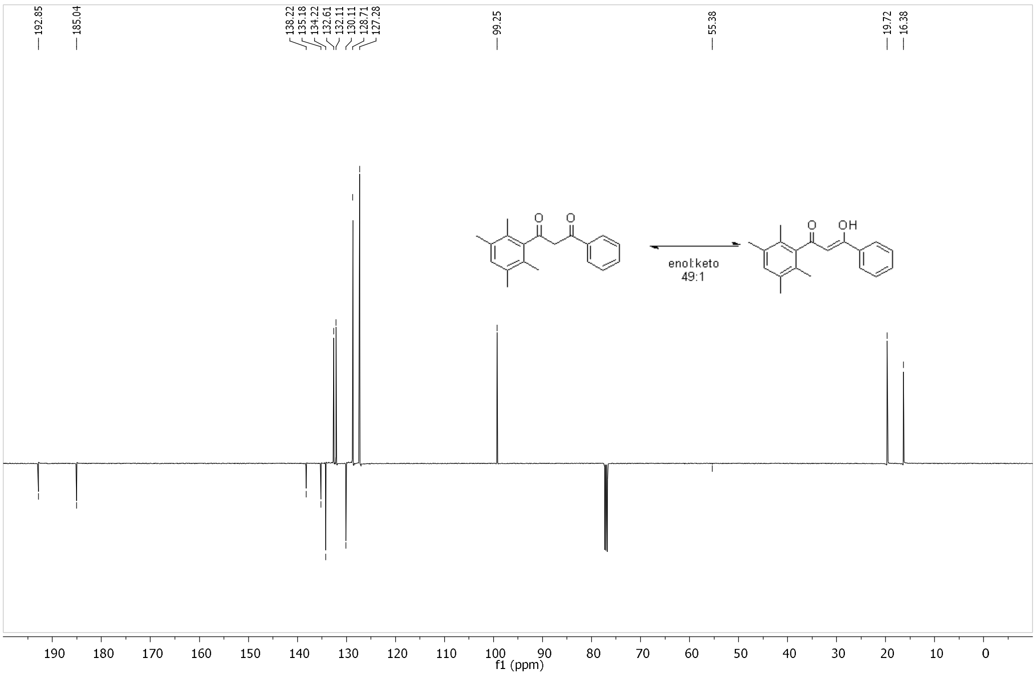

HPLC of 1-phenyl-3-(2,3,5,6-tetramethylphenyl)propane-1,3-dione **18a**.

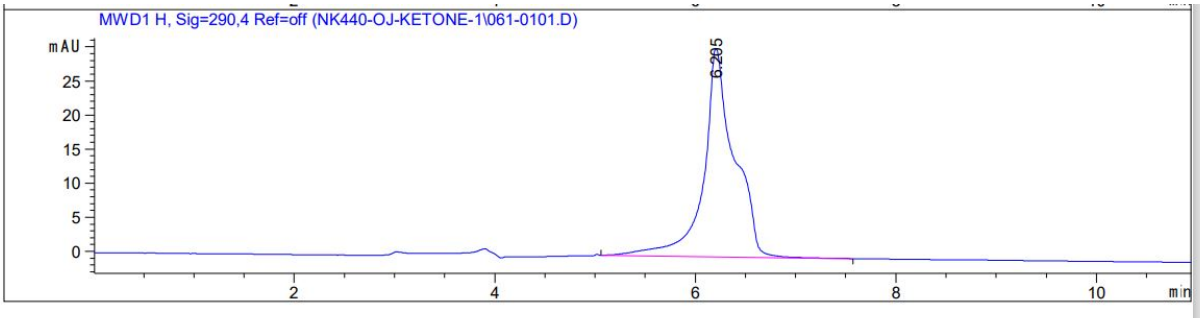

Signal 8: MWD1 H, Sig=290,4 Ref=off

Peak #	RetTime [min]	Type	Width [min]	Area [mAU*s]	Height [mAU]	Area %
1	6.205	BB	0.2823	655.91547	30.57329	100.0000
Totals :				655.91547	30.57329	

3-Hydroxy-3-phenyl-1-(2,3,5,6-tetramethylphenyl)propan-1-one 18b.

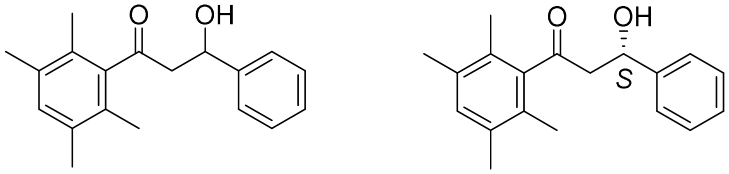

This compound has been reported and fully characterized in racemic form. Cheong, C. B.; Frost, J. R.; Donohoe, T. J. Pentamethylphenyl (Ph*) and related derivatives as useful acyl protecting groups for organic synthesis: a preliminary study. *Synlett* **2020,** *31*, 1828-1832.

Synthesis of a racemic standard: (*R,R*)-3C-Tethered Ru(II)-TsDPEN catalyst (1.1 mg, 1.8 mmol, 0.5 mol%) and (*S,S*)-3C-tethered Ru(II)-TsDPEN catalyst (1.1 mg, 1.8 mmol, 0.5 mol%) were added to FA: TEA (5:2 azeotropic mixture, 0.36 mL) at rt and the mixture was stirred under a nitrogen atmosphere for 15 minutes, after which 1-phenyl-3-(2,3,5,6-tetramethylphenyl)propane-1,3-dione (100 mg, 0.36 mmol) was added. The reaction mixture was stirred under a nitrogen atmosphere and followed by TLC (5:1 hexane: EtOAc). After 24 h, the reaction was quenched using saturated $NaHCO_3$ solution (20 mL). EtOAc (20 mL) was added, and the organic layer was separated. The aqueous layer was extracted with EtOAc (3 x 20 mL) and the combined organic layers were dried ($MgSO_4$) and filtered. The solvent was removed to give the crude product. The product was isolated via flash chromatography on silica eluted with 0-50% EtOAc in petroleum ether to give 3-hydroxy-3-phenyl-1-(2,3,5,6-tetramethylphenyl)propan-1-one **18b** as a white solid (88.4 mg, 0.313 mmol, 88%). TLC: Rf ca 0.30 (5:1 hexane: EtOAc), strong UV and $KMnO_4$; HRMS (ESI+) *m/z*: [M+Na]+ Calcd for $C_{19}H_{22}NaO_2$ 305.1512; Found 305.1510 1; 0.8 ppm error; ^{1}H NMR (500 MHz, $CDCl_3$): δ 7.43 (2H, d, *J* = 7.5, ArH), 7.38 (2H, t, *J* = 7.5, ArH), 7.30 (1H, dd, *J* = 12.4, 5.2, ArH), 6.99 (1H, s, ArH), 5.45 – 5.36 (1H, m, ArCH), 3.54 (1H, t, *J* 2.7, OH), 3.15 (1H, dd, *J* = 18.7, 6.1, CH_2), 3.10 (1H, dd, *J* = 18.7, 6.1, CH_2), 2.22 (6H, s, CH_3), 2.11 (6H, s, CH_3); ^{13}C{^{1}H} NMR (126 MHz, $CDCl_3$): δ 212.1 (C), 142.7 (C), 141.89 (C), 134.5 (C), 131.9 (CH), 128.6 (CH), 127.9 (C), 127.7 (CH), 125.8 (CH), 69.7 (CH), 53.7 (CH_2), 19.4 (CH_3), 15.9 (CH_3); *m/z* (ES-API+) 305.1 (M^+ + Na, 100%); Enantiomeric excess and conversion determined by HPLC analysis (Chiralpak OJ, 30 cm x 6 mm column, hexane:iPrOH 90:10, 1.0 mL/min, T =

25°C) ketone 6.2 min, *R* and *S* isomer 8.3 min and 11.2 min. Configuration assigned by analogy. The data matched that reported.

(*S*)-3-Hydroxy-3-phenyl-1-(2,3,5,6-tetramethylphenyl)propan-1-one **18b.**
(*S,S*)-3C-tethered Ru(II)-TsDPEN catalyst (6.2 mg, 0.01 mmol, 1 mol%) was added to FA: TEA (5:2 azeotropic mixture, 0.50 mL) at rt and the mixture was stirred under a nitrogen atmosphere for 10-15 minutes; after which 1-phenyl-3-(2,3,5,6-tetramethylphenyl)propane-1,3-dione (280 mg, 1.00 mmol) was added. The reaction mixture was stirred under a nitrogen atmosphere overnight. The reaction was followed by TLC (5:1 hexane: EtOAc). After 70 h, the reaction was quenched using saturated $NaHCO_3$ solution (20 mL). EtOAc (20 mL) was added, and the organic layer was separated. The aqueous layer was extracted with EtOAc (3 x 20 mL), and the combined organic layers were dried ($MgSO_4$) and filtered. The solvent was removed to give the crude product. The product was isolated via flash chromatography on silica eluted with 0-60% EtOAc in petroleum ether to give (*S*)-3-hydroxy-3-phenyl-1-(2,3,5,6-tetramethylphenyl)propan-1-one **18b** as a white solid (222 mg, 0.79 mmol, 79%). The reaction was also followed by HPLC (Chiralpak OJ, 30 cm x 6 mm column, hexane:iPrOH 90:10, 1.0 mL/min, T = 25°C); $[\alpha]_D^{23}$ – 41.2 (c 0.146 in $CHCl_3$); (after 70 h, 100% conversion, >99% ee (*S*)).

^{1}H NMR (500 MHz, $CDCl_3$) of 3-hydroxy-3-phenyl-1-(2,3,5,6-tetramethylphenyl)propan-1-one **18b.**

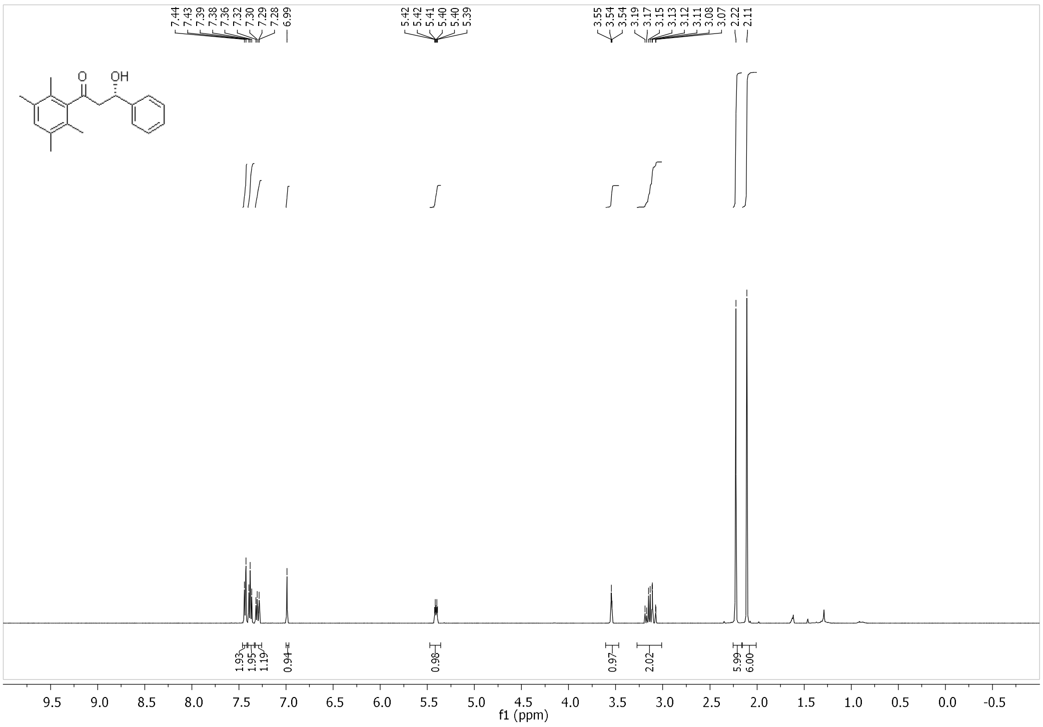

$^{13}C\{^{1}H\}$ NMR (126 MHz, $CDCl_3$) of 3-hydroxy-3-phenyl-1-(2,3,5,6-tetramethylphenyl)propan-1-one **18b.**

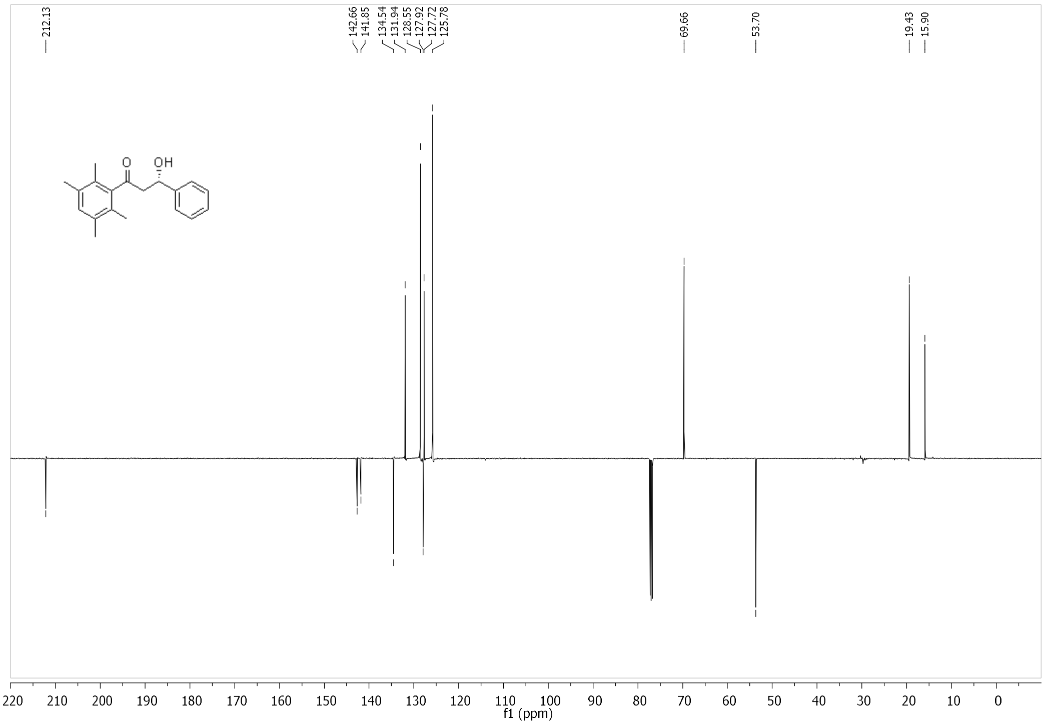

COSY of 3-hydroxy-3-phenyl-1-(2,3,5,6-tetramethylphenyl)propan-1-one **18b.**

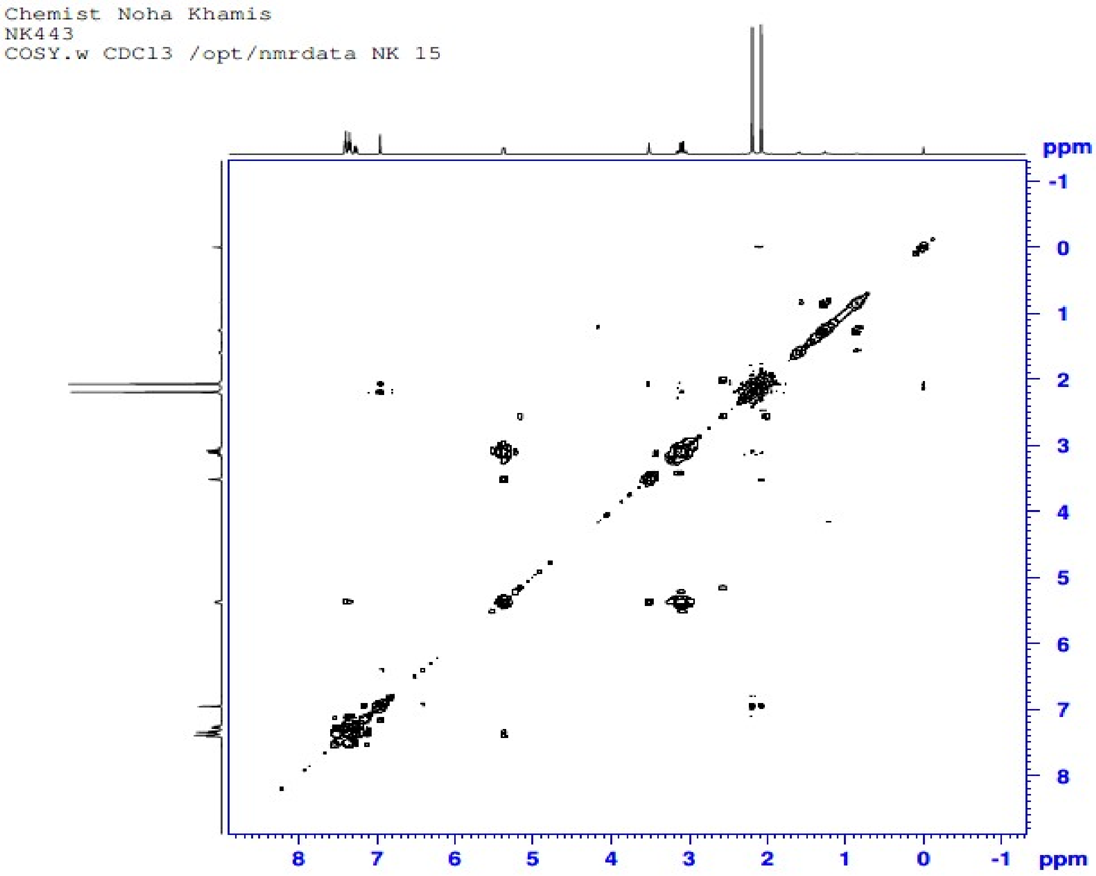

HSQC of 3-hydroxy-3-phenyl-1-(2,3,5,6-tetramethylphenyl)propan-1-one **18b.**

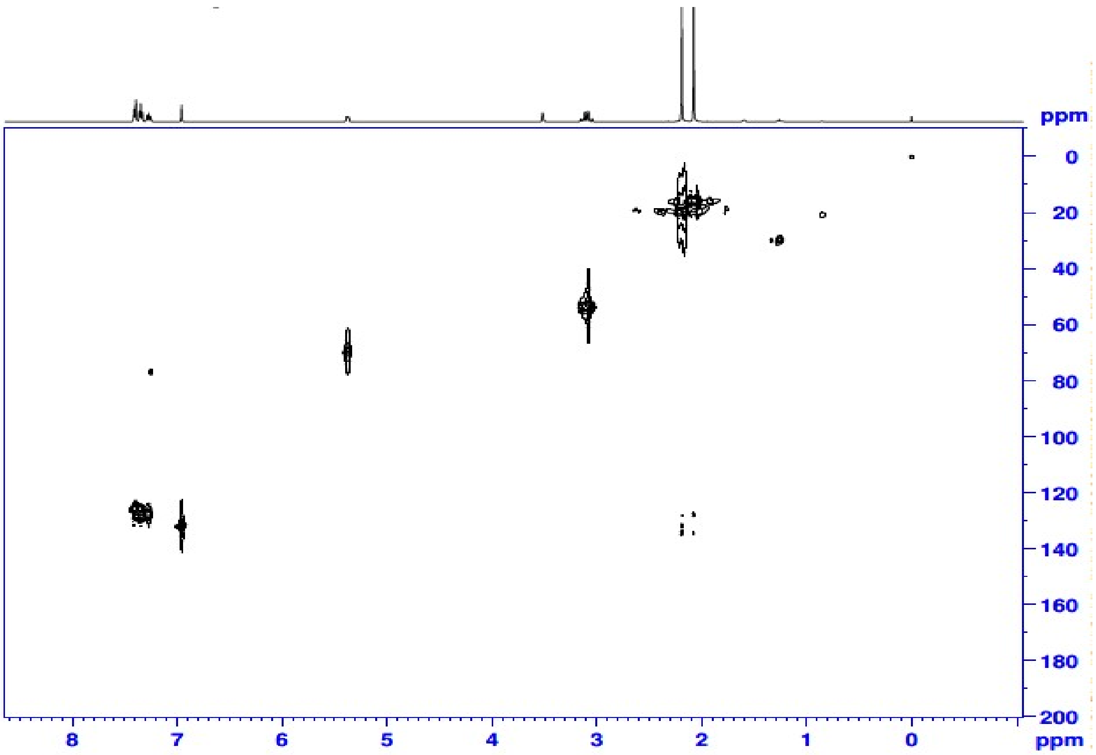

HMBC of 3-hydroxy-3-phenyl-1-(2,3,5,6-tetramethylphenyl)propan-1-one **18b.**

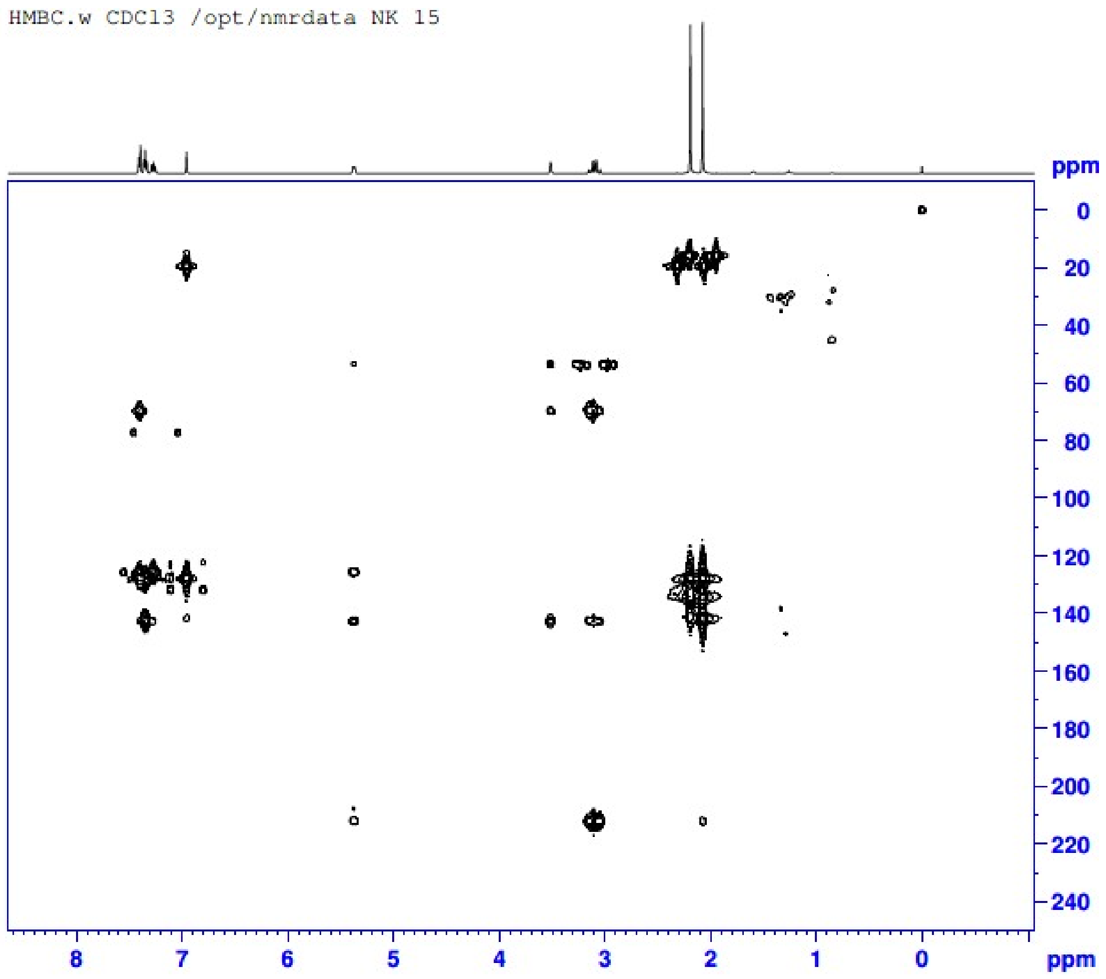

HPLC ofracemic 3-hydroxy-3-phenyl-1-(2,3,5,6-tetramethylphenyl)propan-1-one **18b.**

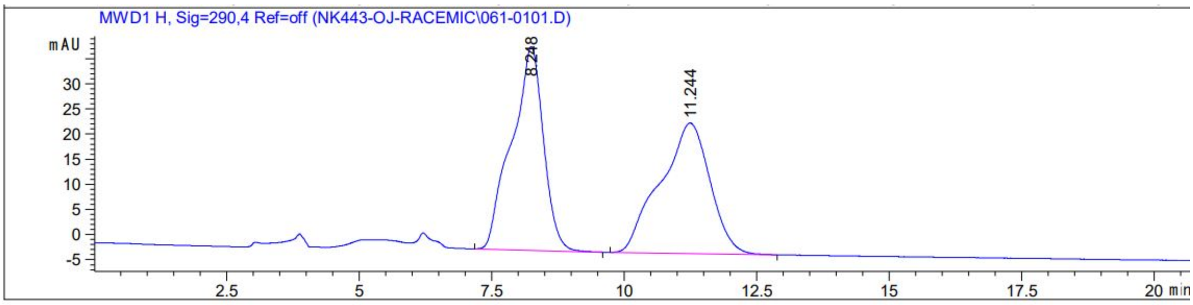

Signal 8: MWD1 H, Sig=290,4 Ref=off

Peak #	RetTime [min]	Type	Width [min]	Area [mAU*s]	Height [mAU]	Area %
1	8.248	BB	0.5814	1693.29895	40.52917	50.0273
2	11.244	BB	0.9016	1691.44788	25.97773	49.9727
Totals :				3384.74683	66.50690	

HPLC of (*R*)-3-hydroxy-3-phenyl-1-(2,3,5,6-tetramethylphenyl)propan-1-one **18b.**

(*S,S*)-3C-tethered Ru(II)-TsDPEN catalyst (after 24 h, 100% conversion, >99% ee (*S*)).

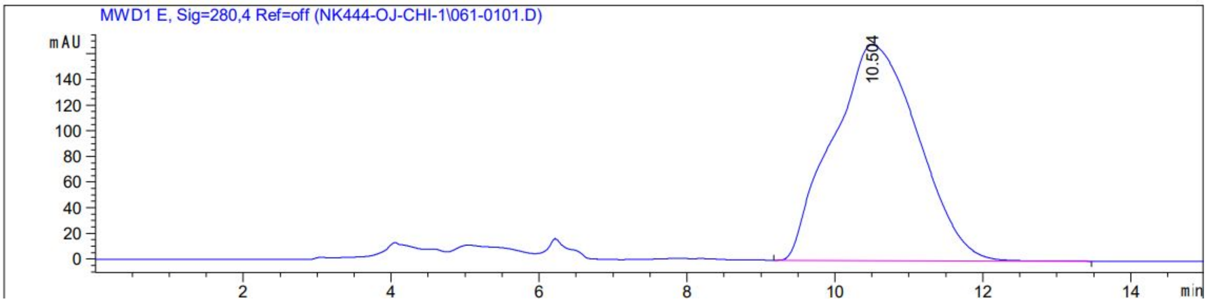

Signal 5: MWD1 E, Sig=280,4 Ref=off

Peak #	RetTime [min]	Type	Width [min]	Area [mAU*s]	Height [mAU]	Area %
1	10.504	BB	1.1895	1.34817e4	168.12970	100.0000
Totals :				1.34817e4	168.12970	

Benzo[d] [1,2]dioxine-1,4-dione (phthaloyl peroxide).

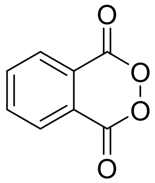

This compound has been reported and fully characterized. Alvi, S.; Singleton, D. A., Energy Read-out as a Probe of Kinetically Hidden Transition States. *Org Lett* **2021,** *23*, 2174-2177.

To a vigorously stirred suspension of $Na_2CO_3 \cdot 1.5H_2O_2$ (2.4 g, 15.5 mmol) in CH_2Cl_2 (50 mL) was added phthaloyl chloride (1.5 mL, 10.0 mmol) at RT and stirred for 3 h. The reaction mixture was filtered under suction through a pad of Celite®, eluting with CH_2Cl_2 (50 mL), and the eluent concentrated in vacuo, ensuring that the water bath temperature does not exceed 30 °C to avoid the danger of explosion. The residue was re-dissolved in a minimum amount of warm benzene that heated using oil bath at 60 ºC, then poured into ice-cold pentane (50 mL). The white solid precipitated immediately and collected by Büchner filtration and washed by cold pentane to afford phthaloyl peroxide (729 mg, 44%) as a white solid. The product should be kept in a freezer because it slowly decomposes at r.t; ^{1}H NMR (500 MHz, $CDCl_3$): δ 8.35 – 8.29 (1H, m, ArH), 8.08 – 8.02 (2H, m, ArH); $^{13}C\{^1H\}$ NMR (126 MHz, $CDCl_3$): δ 162.0 (C), 136.5 (CH), 130.2 (CH), 123.7 (C). The data matched that reported.

^1H NMR (500 MHz, $CDCl_3$) of benzo[d] [1,2]dioxine-1,4-dione.

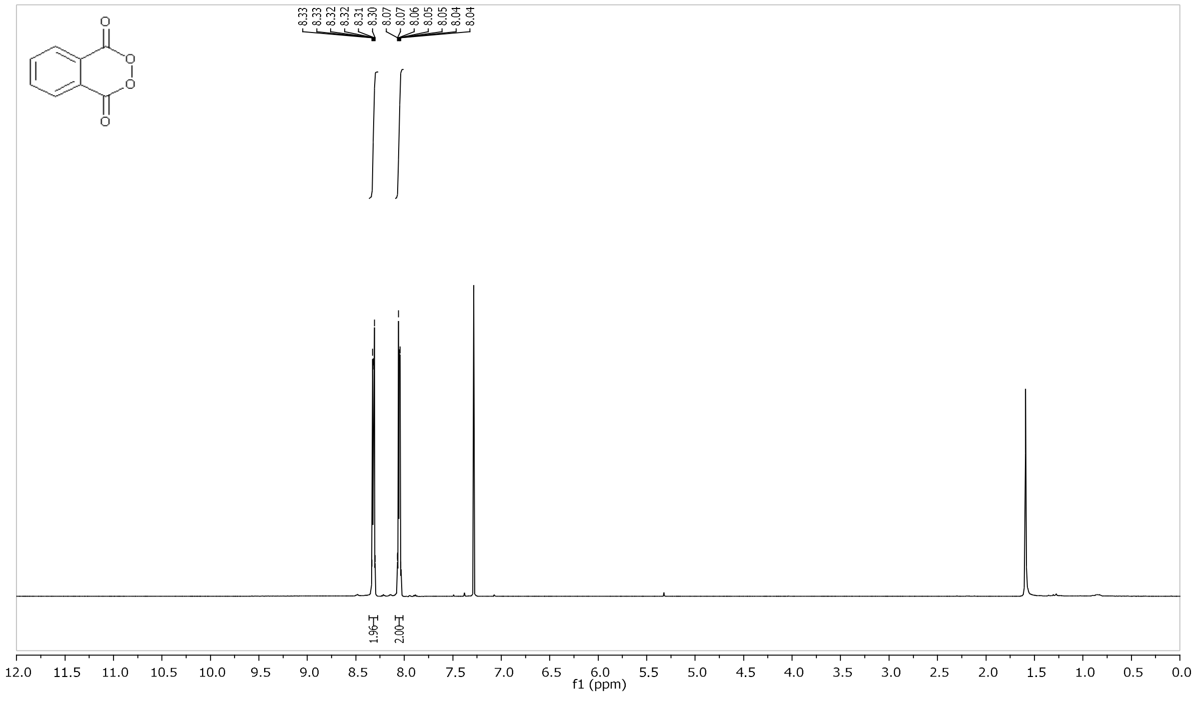

$^{13}C\{^1H\}$ NMR (126 MHz, $CDCl_3$) of benzo[d] [1,2]dioxine-1,4-dione.

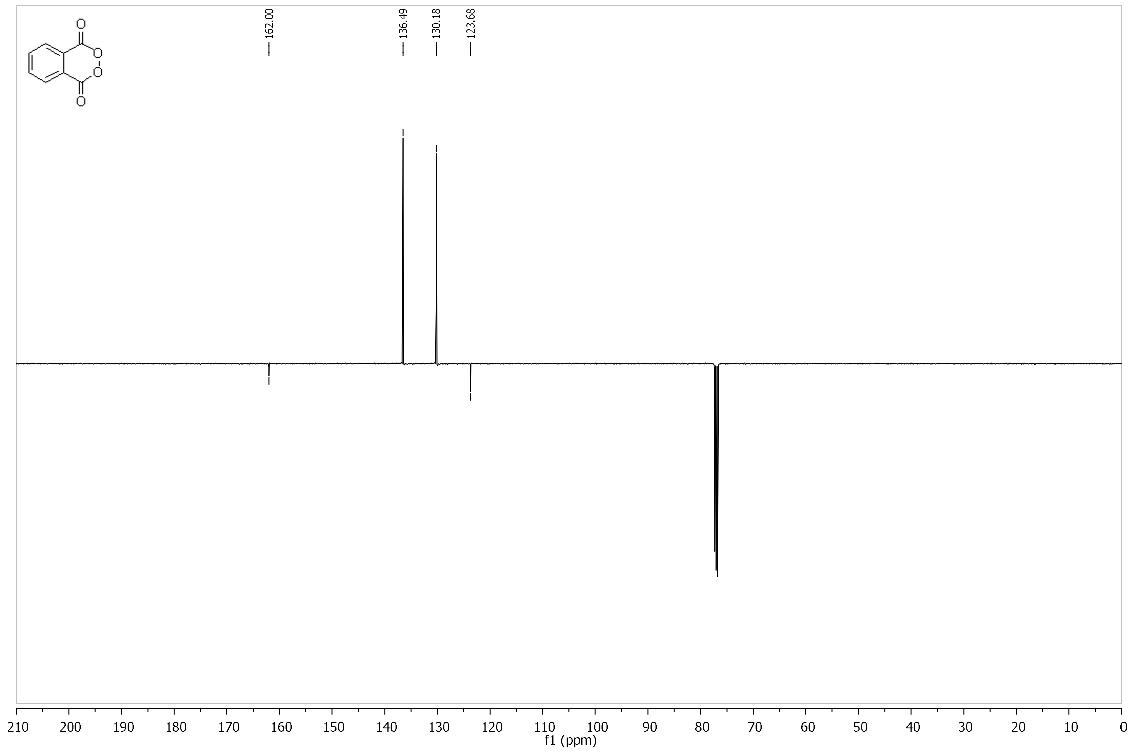

3-Hydroxy-1-(4-hydroxy-2,3,5,6-tetramethylphenyl)-3-phenylpropan-1-one 25.

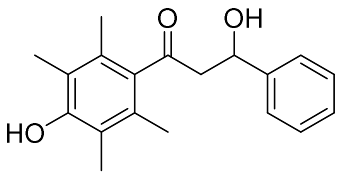

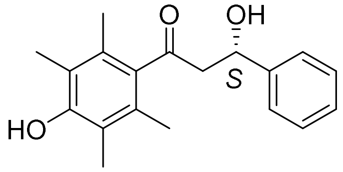

This compound has been reported and fully characterized in racemic form. Cheong, C. B.; Frost, J. R.; Donohoe, T. J. Pentamethylphenyl (Ph*) and related derivatives as useful acyl protecting groups for organic synthesis: a preliminary study. *Synlett* **2020,** *31*, 1828-1832.

Synthesis of a racemic standard: To a solution of 3-hydroxy-3-phenyl-1-(2,3,5,6-tetramethylphenyl)propan-1-one **18b** (86.1 mg, 0.305 mmol) in HFIP (3.1 mL), Phthaloyl peroxide (81.3mg, 0.495 mmol) was added at r.t and the solution warmed to 40°C and left for 24 h. After this time, the reaction was concentrated under vacuum at 23°C, then 2.95:0.35 MeOH : sat.$NaHCO_3$ was added and warmed to 40°C and left for 48 h. After completion, the reaction mixture was diluted with brine (20 mL) and DCM (20 mL) and extracted using EtOAc (3 x 10 mL) then dried using $MgSO_4$. Solvent was removed to give the crude product. The product was isolated via flash chromatography on silica eluted with 5-50% EtOAc in petroleum ether to give 3-hydroxy-1-(4-hydroxy-2,3,5,6-tetramethylphenyl)-3-phenylpropan-1-one **25** as a yellow solid (50.9 mg, 0.171 mmol, 56%). TLC: Rf ca 0.9 (1:1 petroleum ether: EtOAc), weak UV and PMA active; Mp: 136.5°C; HRMS (ESI+) *m/z*: [M+Na]+ Calcd for $C_{19}H_{22}NaO_3$ 321.1461; Found 321.1456; 1.5 ppm error; ν_{max} 3563, 3377, 2920, 2866 cm^{-1}; ^1H NMR (500 MHz, $CDCl_3$): δ 7.42 (2H, d, *J* = 7.4, ArH), 7.37 (2H, t, *J* = 7.5, ArH), 7.32 – 7.27 (1H, m, ArH), 5.38 (1H, dd, *J* = 8.9, 2.7, ArCH), 5.10 (1H, s, OH), 3.65 (1H, s, OH), 3.23 – 3.00 (2H, m, CH_2), 2.14 (6H, s, CH_3), 2.12 (6H, s, CH_3); $^{13}C\{^1H\}$ NMR (126 MHz, $CDCl_3$): δ 212.3 (C=O), 152.2 (COH), 142.7 (C), 134.9 (C), 129.0 (C), 128.5 (CH), 127.7 (CH), 125.8 (CH), 120.1 (C), 69.8 (CH), 54.1 (CH_2), 16.6 (CH_3), 11.7 (CH_3); *m/z* (ES-API+) 321.1 (M^+ + Na, 100%); Enantiomeric excess and conversion determined by HPLC analysis (Chiralpak OJ, 30 cm x 6 mm column, hexane:iPrOH 90:10, 1.0 mL/min, T = 25°C) *R* and *S* isomer 35.00 min and 39.65 min. The data matched that reported.

(*S*)-3-Hydroxy-1-(4-hydroxy-2,3,5,6-tetramethylphenyl)-3-phenylpropan-1-one **25.:** To a solution of (*S*)-3-hydroxy-3-phenyl-1-(2,3,5,6-tetramethylphenyl)propan-1-one **18b** (86.6 mg, 0.307 mmol) was in HFIP (3.1 mL), phthaloyl peroxide (75.4 mg, 0.459 mmol) was added at r.t and the solution warmed to 40 ºC and left for 24 h, then concentrated under vacuum at 23 ºC, then 2.8:0.29 MeOH : sat.$NaHCO_3$ was added and the reaction was warmed to 40 ºC and left for 48 h after completion and the reaction mixture diluted with brine (20 mL) and DCM (20 mL) and extracted using DCM (3 x 10 mL) then dried using $MgSO_4$. Solvent was removed to give the crude product. The product was isolated via flash chromatography on silica eluted with 5-50% EtOAc in petroleum ether to give (*S*)-3-hydroxy-1-(4-hydroxy-2,3,5,6-tetramethylphenyl)-3-phenylpropan-1-one **25** as a yellow solid (72.6 mg, 0.243 mmol, 77%). The reaction was also followed by HPLC (Chiralpak OJ, 30 cm x 6 mm column, hexane:iPrOH 90:10, 1.0 mL/min, T = 25 °C); $[\alpha]_D^{29}$ – 32.2 (c 0.03 in $CHCl_3$); (>99% ee, (*S*)).

^1H NMR (500 MHz, $CDCl_3$) of 3-hydroxy-1-(4-hydroxy-2,3,5,6-tetramethylphenyl)-3-phenylpropan-1-one.

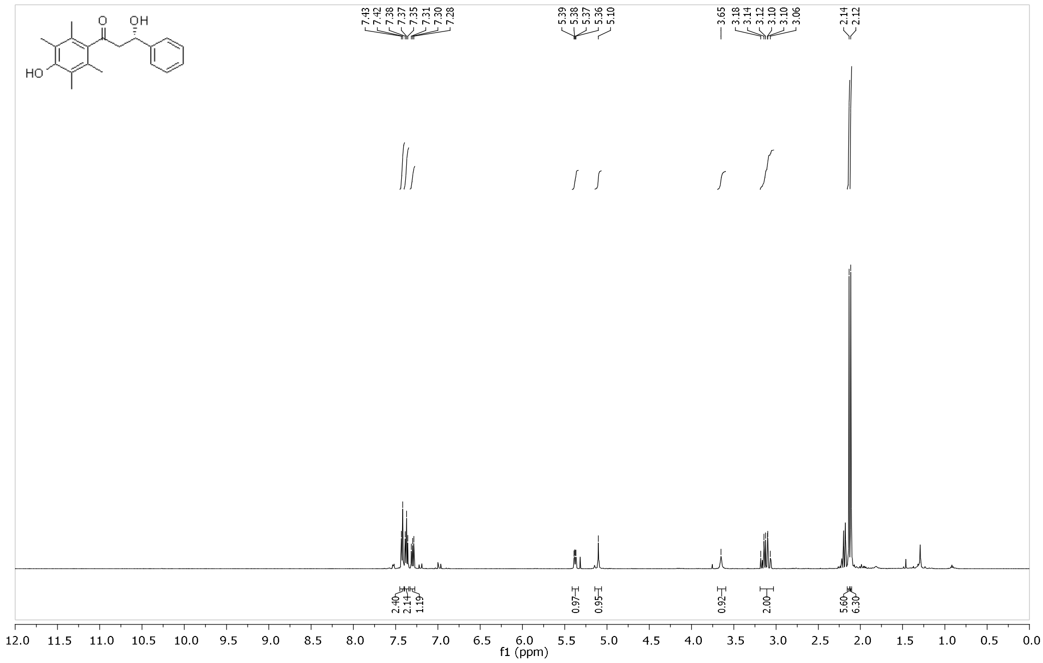

$^{13}C\{^{1}H\}$ NMR (126 MHz, $CDCl_3$) of (*S*)-3-hydroxy-1-(4-hydroxy-2,3,5,6-tetramethylphenyl)-3-phenylpropan-1-one.

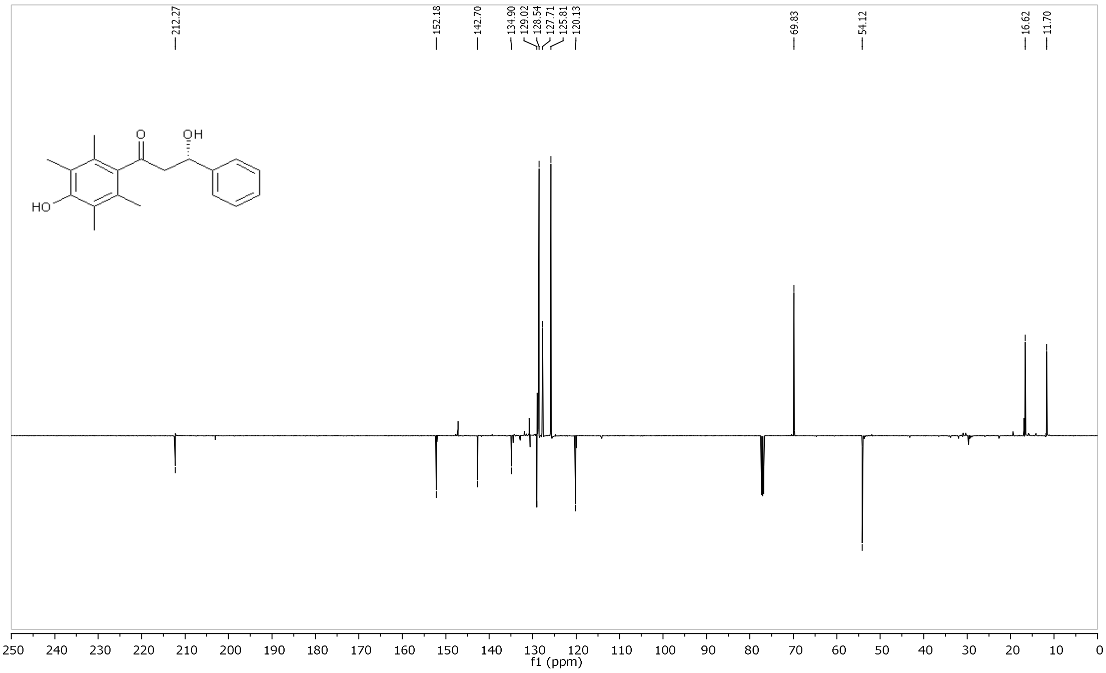

COSY (500 MHz, $CDCl_3$) of (*S*)-3-hydroxy-1-(4-hydroxy-2,3,5,6-tetramethylphenyl)-3-phenylpropan-1-one.

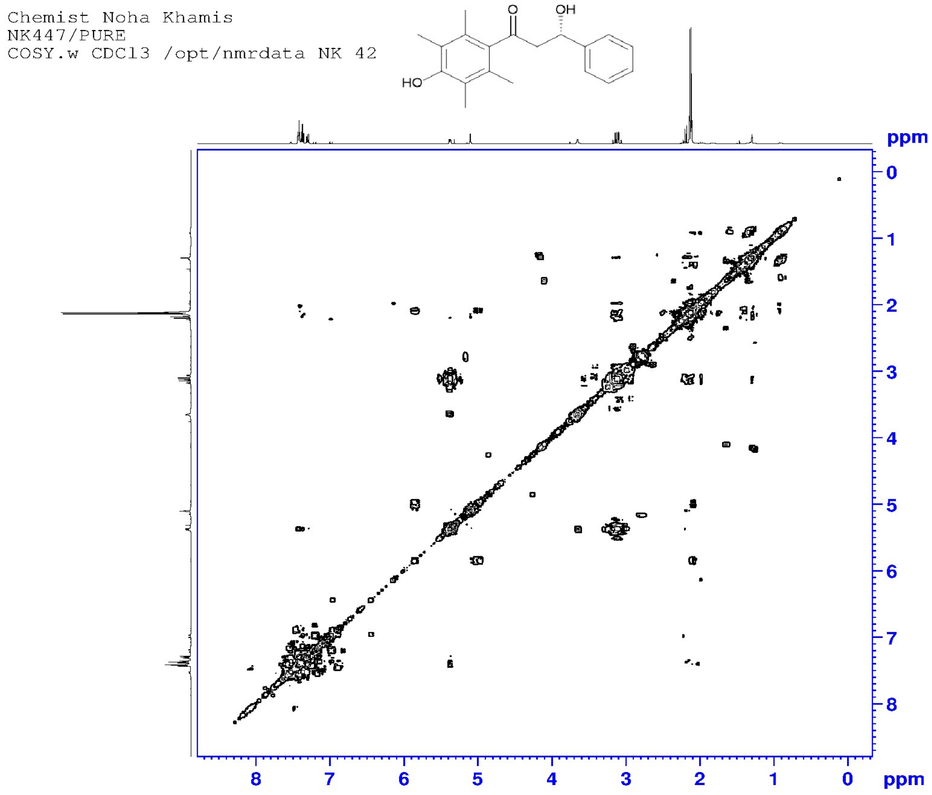

HSQC (126 MHz, $CDCl_3$) of (*S*)-3-hydroxy-1-(4-hydroxy-2,3,5,6-tetramethylphenyl)-3-phenylpropan-1-one

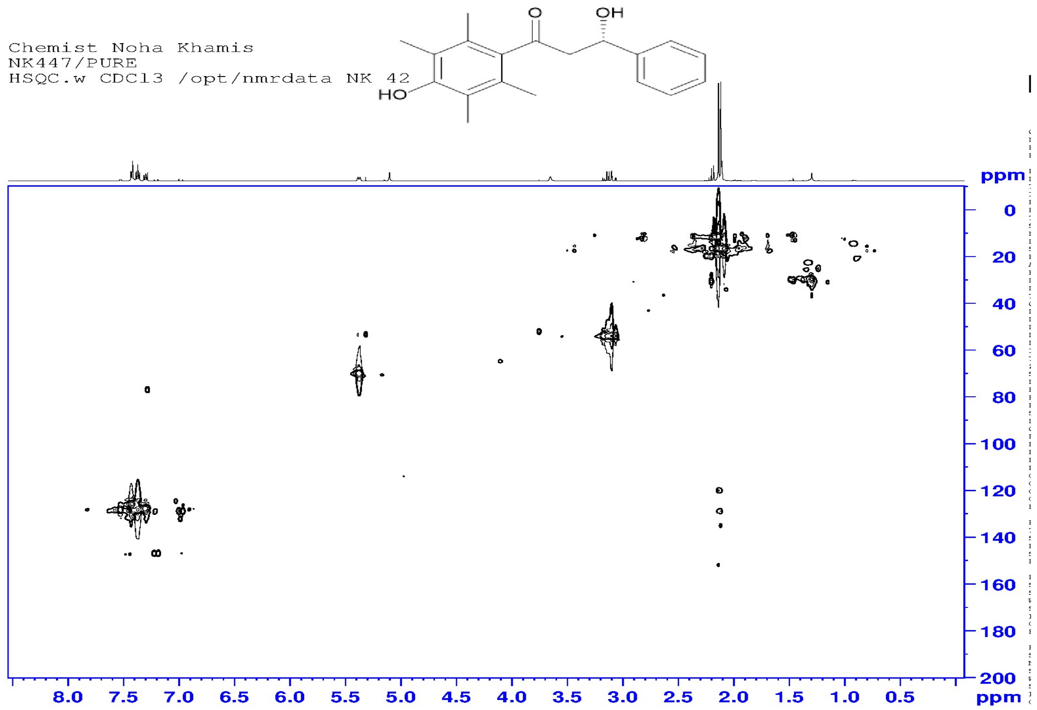

HMBC (126 MHz, $CDCl_3$)

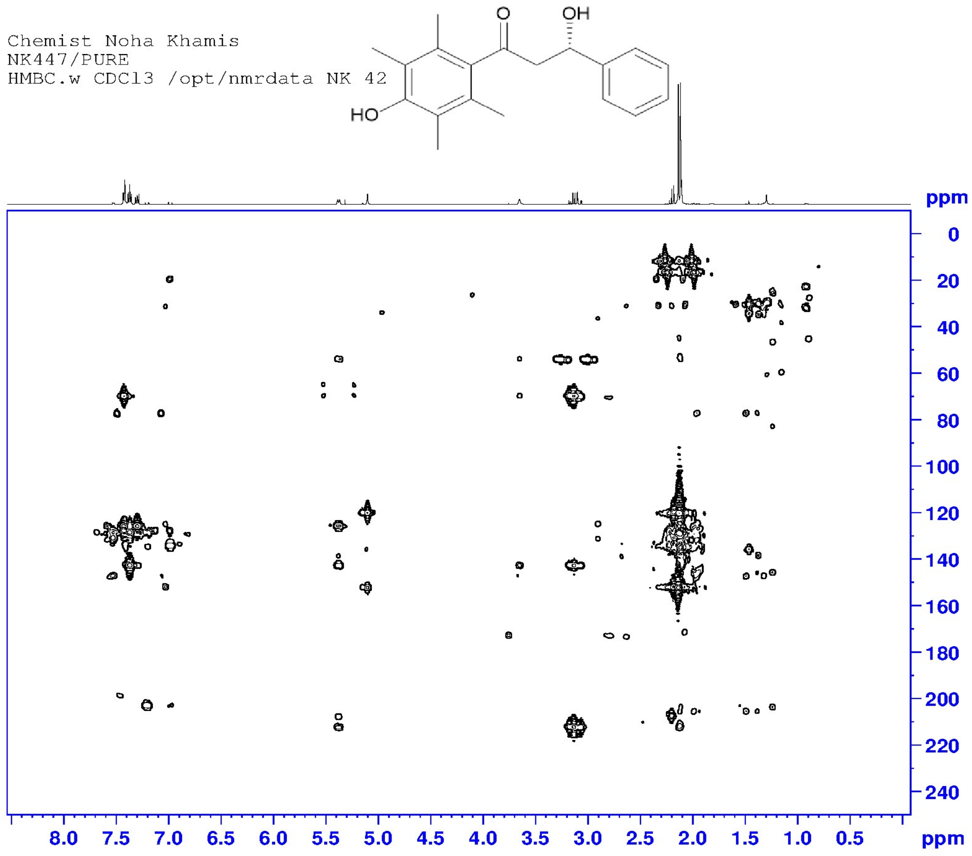

HPLC of racemic (*S*)-3-Hydroxy-1-(4-hydroxy-2,3,5,6-tetramethylphenyl)-3-phenylpropan-1-one.

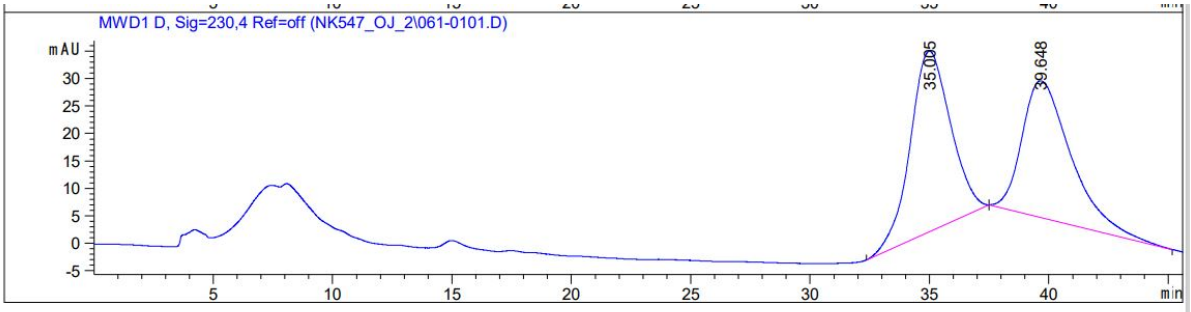

Signal 4: MWD1 D, Sig=230,4 Ref=off

Peak #	RetTime [min]	Type	Width [min]	Area [mAU*s]	Height [mAU]	Area %
1	35.005	BB	1.4833	3857.44336	32.86555	51.9614
2	39.648	BBA	1.6890	3566.22925	24.86937	48.0386

Totals : 7423.67261 57.73492

HPLC of (*S*)-3-Hydroxy-1-(4-hydroxy-2,3,5,6-tetramethylphenyl)-3-phenylpropan-1-one :

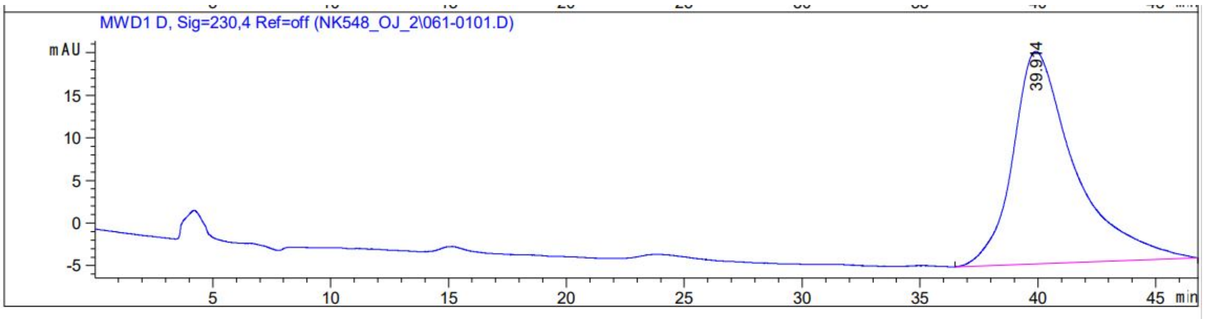

Signal 4: MWD1 D, Sig=230,4 Ref=off

Peak #	RetTime [min]	Type	Width [min]	Area [mAU*s]	Height [mAU]	Area %
1	39.914	BBA	2.1429	4555.77100	24.94275	100.0000

Totals : 4555.77100 24.94275

(*S*)-Methyl 3-hydroxy-3-phenylpropanoate 26.

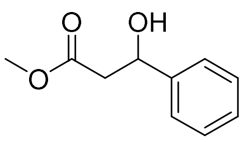

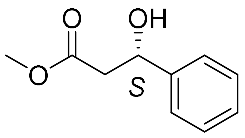

This compound has been reported and fully characterized. Cheong, C. B.; Frost, J. R.; Donohoe, T. J. Pentamethylphenyl (Ph*) and related derivatives as useful acyl protecting groups for organic synthesis: a preliminary study. *Synlett* **2020,** *31*, 1828-1832.

Synthesis of a racemic standard:

To a solution of 3-hydroxy-1-(4-hydroxy-2,3,5,6-tetramethylphenyl)-3-phenylpropan-1-one **25** (50.9 mg, 0.17 mmol) in MeOH (4 mL) was titrated a solution of CAN (411 mg, 0.75 mmol) in MeOH (1.8 mL) at RT in the open atmosphere until the color of the CAN solution persisted (orange color). The reaction mixture was diluted with H_2O (7 mL) and stirred for 5–10 minutes then further diluted with DCM (4 mL) and saturated with NaCl. The layers were separated, and the aqueous layer extracted with DCM (5 × 3 mL), and the combined organics dried (Na_2SO_4) and concentrated in vacuo. The product was isolated via flash chromatography on silica eluted with 10-50% EtOAc in petroleum ether to afford methyl 3-hydroxy-3-phenylpropanoate **26** (23.1 mg, 0.19 mmol, 75%) as a yellow oil; TLC: Rf ca 0.1 (1:1 petroleum ether: EtOAc), PMA active; ^{1}H NMR (400 MHz, $CDCl_3$): δ 7.35 – 7.25 (4H, m, ArH), 7.24 – 7.17 (1H, m, ArH), 5.07 (1H, dd, *J* = 8.8, 3.9, CH), 3.66 (2H, s, OCH_3), 3.13 (1H, br. s, OH), 2.74 – 2.65 (2H, m, CH_2); $^{13}C\{^1H\}$ NMR (101 MHz, $CDCl_3$): δ 172.8 (C), 142.5 (C), 128.6 (CH), 127.9 (CH), 125.7 (CH), 70.3 (CH_2), 51.9 (CH_2), 43.2 (CH_3); *m/z* (ES-API+) 203.1 (M^+ + Na, 100%). Enantiomeric excess and conversion determined by HPLC analysis (Chiralpak OD-H, 30 cm x 6 mm column, hexane:iPrOH 95:05, 0.5 mL/min, T = 25°C) *R* and *S* isomer 34.8 min and 28.2 min. Data matched that reported.

(*S*)-Methyl 3-hydroxy-3-phenylpropanoate **26**. To a solution of (*S*)-3-hydroxy-1-(4-hydroxy-2,3,5,6-tetramethylphenyl)-3-phenylpropan-1-one (44.8 mg, 0.15 mmol) in MeOH (3.5 mL) was titrated a solution of CAN (411 mg, 0.75 mmol) in MeOH (1.8 mL) at RT in the open atmosphere until the color of the CAN solution persisted (orange color). The reaction mixture was diluted with H_2O (6 mL) and stirred for 5–

10 minutes then further diluted with DCM (4 mL) and saturated with NaCl. The layers were separated, and the aqueous layer extracted with DCM (5 × 3 mL), and the combined organics dried (Na_2SO_4) and concentrated in vacuo. The product was isolated via flash chromatography on silica eluted with 10-50% EtOAc in petroleum ether to afford methyl (*S*)-3-hydroxy-3-phenylpropanoate (16.1 mg, 0.09 mmol, 59%) as a yellow oil; TLC: Rf ca 0.1 (1:1 petroleum ether: EtOAc), PMA active; ^{1}H NMR (400 MHz, $CDCl_3$) δ 7.35 – 7.25 (4H, m, ArH), 7.24 – 7.17 (1H, m, ArH), 5.07 (1H, dd, *J* = 8.8, 3.9, CH), 3.66 (2H, s, OCH_3), 3.13 (1H, br. s, OH), 2.74 – 2.65 (2H, m, CH_2); δ_C $^{13}C\{^1H\}$ NMR (101 MHz, $CDCl_3$): δ 172.8 (C), 142.5 (C), 128.6 (CH), 127.9 (CH), 125.7 (CH), 70.3 (CH_2), 51.9 (CH_2), 43.2 (CH_3); *m/z* (ES-API+) 203.1 (M^+ + Na, 100%). The reaction was also followed by HPLC (Chiralpak OD-H, 30 cm x 6 mm column, hexane:iPrOH 95:05, 0.5 mL/min, T = 25°C); $[\alpha]_D^{20}$ – 40.8 (c 0.04 in $CHCl_3$); (98% ee (*S*)).

^{1}H NMR (400 MHz, $CDCl_3$) of (*S*)-methyl 3-hydroxy-3-phenylpropanoate **26**.

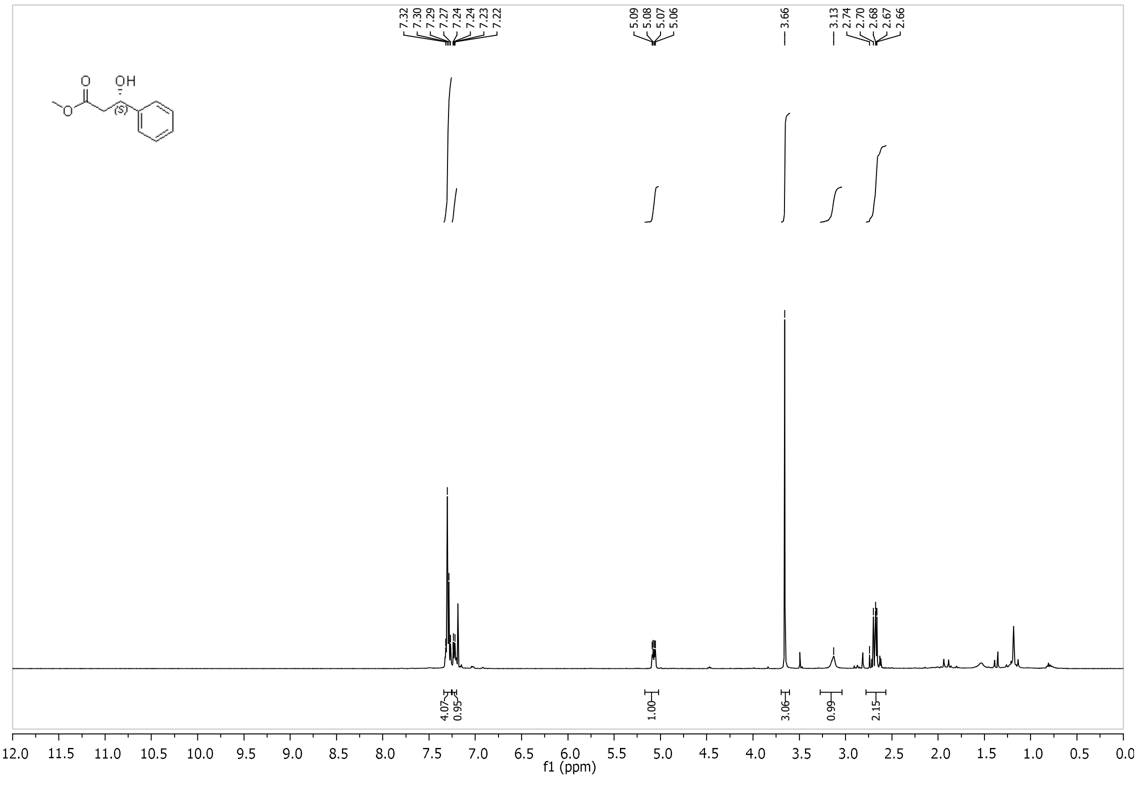

$^{13}C\{^{1}H\}$ NMR (101 MHz, $CDCl_3$) of (*S*)-methyl 3-hydroxy-3-phenylpropanoate **26**.

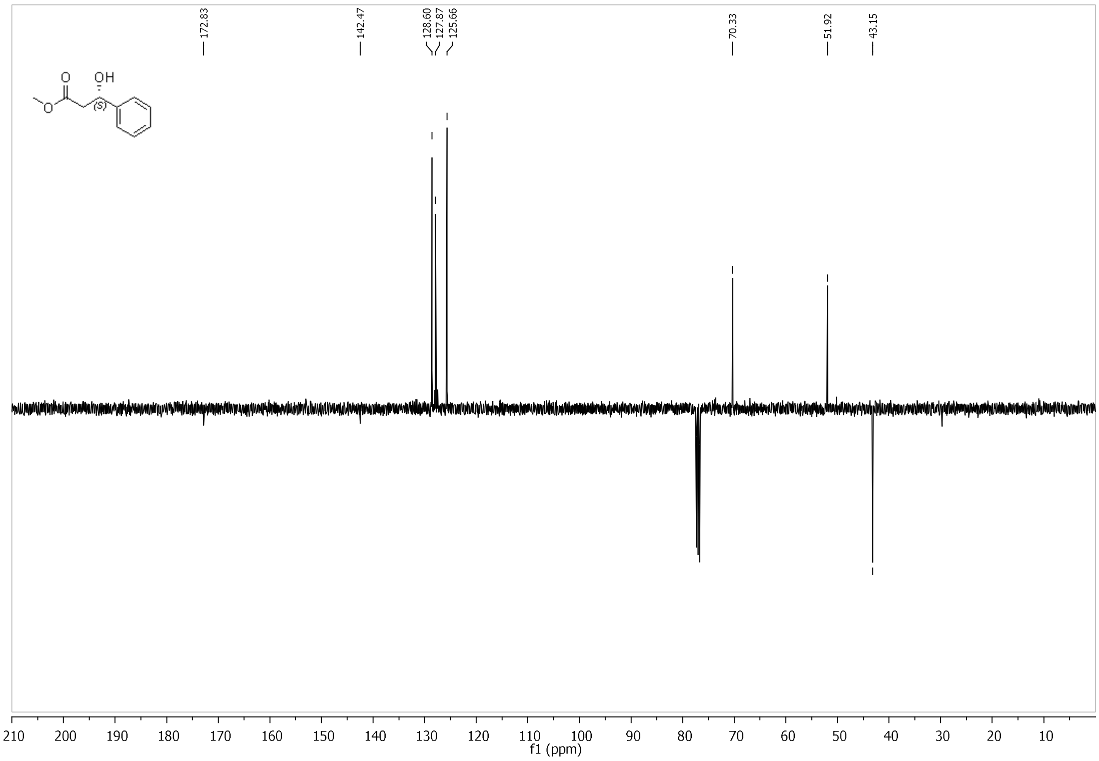

HPLC of racemic methyl 3-hydroxy-3-phenylpropanoate **26**.

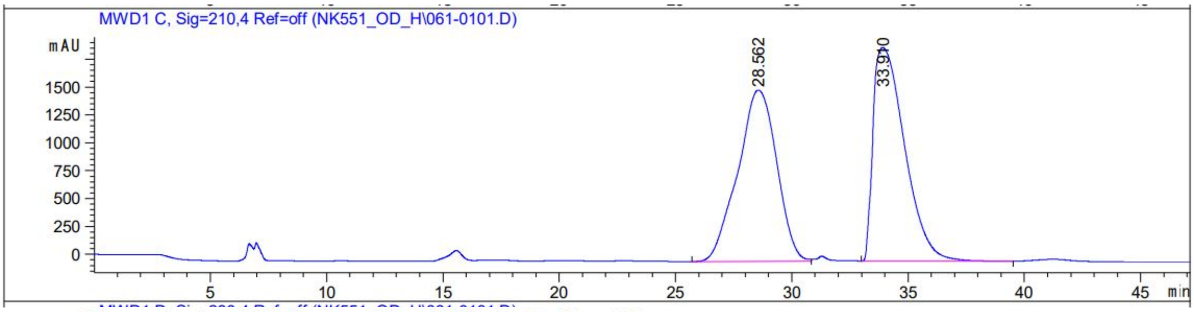

Signal 3: MWD1 C, Sig=210,4 Ref=off

Peak #	RetTime [min]	Type	Width [min]	Area [mAU*s]	Height [mAU]	Area %
1	28.562	BV	1.5641	1.78841e5	1536.31641	49.6763
2	33.910	VB	1.1248	1.81172e5	1916.59631	50.3237
Totals :				3.60013e5	3452.91272	

HPLC of (*S*)-methyl 3-hydroxy-3-phenylpropanoate **26**.

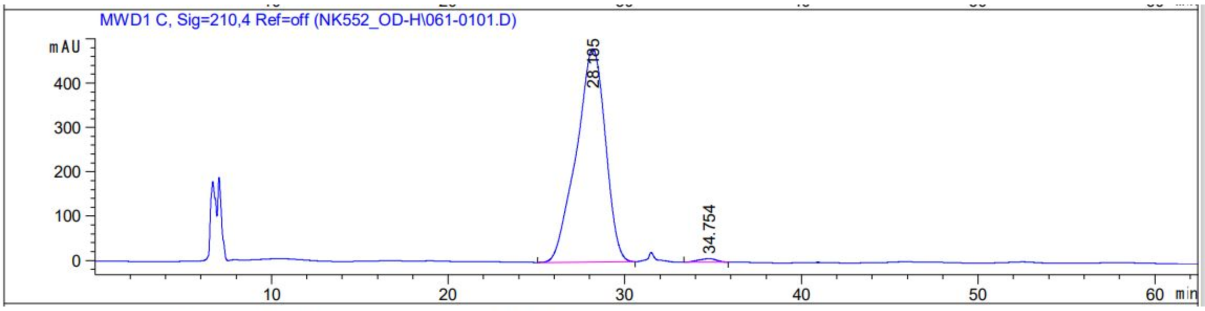

Signal 3: MWD1 C, Sig=210,4 Ref=off

Peak #	RetTime [min]	Type	Width [min]	Area [mAU*s]	Height [mAU]	Area %
1	28.185	BB	1.6758	5.49004e4	480.94586	98.9708
2	34.754	BB	0.8418	570.92139	8.10476	1.0292

Totals :				5.54714e4	489.05063	

1-(4-Chlorophenyl)-3-(2,3,5,6-tetramethylphenyl)propane-1,3-dione 19a.

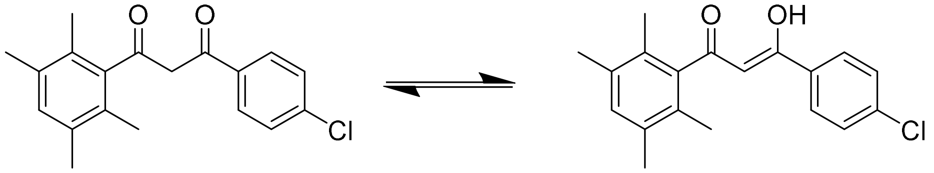

This compound is novel.

To a solution of sodium hydride (397 mg, 60% dispersion in mineral oil, 9.93 mmol) in THF (4 mL) at 0 °C was added dropwise a solution of 1-(2,3,5,6-tetramethylphenyl)ethan-1-one **7** (350 mg, 1.98 mmol) in THF (4 mL). The reaction mixture was stirred under a nitrogen atmosphere at 0 °C for 30 min and then stirred under a nitrogen atmosphere at rt for 30 min, after which ethyl 4-chlorobenzoate (1.83 g, 9.9 mmol) was added dropwise at 0 °C. The reaction mixture was refluxed at 66 °C and left stirring under the nitrogen atmosphere overnight. The reaction was followed by TLC (4:1 hexane: EtOAc). The mixture was quenched by 2M HCl solution (20 mL). EtOAc (20 mL) was added, and the organic layer was separated. The aqueous layer was extracted with EtOAc (3 × 20 mL) and the combined organic layers were washed with saturated $NaHCO_3$ solution (2 × 20 mL) and brine (20 mL), dried ($MgSO_4$) and filtered. Solvent was removed to give the crude product. The product was isolated via flash chromatography on silica eluted with 1% EtOAc in hexane to give 1-(4-chlorophenyl)-3-(2,3,5,6-tetramethylphenyl)propane-1,3-dione **19a** as a white solid (538.3 mg, 1.71 mmol, 86%). TLC: Rf ca 0.31 (9:1 hexane: EtOAc), strong UV and $KMnO_4$; Mp: 115.8 °C; HRMS (ESI+) *m/z*: [M+Na]+, Calcd for $C_{19}H_{19}ClNaO_2$ 337.0966; Found 337.0955; 3.2 ppm error; ν_{max} 2962, 2922, 2860, 1719, 1590 cm^{-1}; enol: keto = 50:1; ^1H NMR (500 MHz, $CDCl_3$): δ 7.89 (2H, d, J = 8.6, CH of Ar), 7.46 (2 H, d, J = 8.6, CH of ArH), 7.05 (1H, s, ArH), 6.29 (1H, s, CH of enol form), 4.15 (2H, m, CH_2 of keto form), 2.39 (6H, s, CH_3 of keto form), 2.34 (6H, s, CH_3 of keto form), 2.27 (6H, s, CH_3 of enol form), 2.22 (6H, s, CH_3 of enol form) ppm (fractional integrals not listed due to predominance of enol form); $^{13}C\{^1H\}$ NMR (126 MHz, $CDCl_3$): δ 192.9 (C), 183.9 (C), 138.9 (C), 138.0 (C), 134.3 (C), 133.7 (C), 132.2 (CH),130.1 (C), 129.0 (CH), 128.6 (CH), 99.1 (CH), 55.4 (CH_2), 19.7 (CH_3), 16.4 (CH_3); *m/z* (ES-API+) 337.1 (M^+ + Na, 100%); Enantiomeric excess and conversion determined by HPLC analysis (Chiralpak OJ, 30 cm x 6 mm column,

hexane:iPrOH 90:10, 1.0 mL/min, T = 25°C) ketone 6.6 min, *S* and *R* isomer 7.1 min and 8.4 min.

^{1}H NMR (500 MHz, $CDCl_3$) of 1-(4-chlorophenyl)-3-(2,3,5,6-tetramethylphenyl)propane-1,3-dione **19a**.

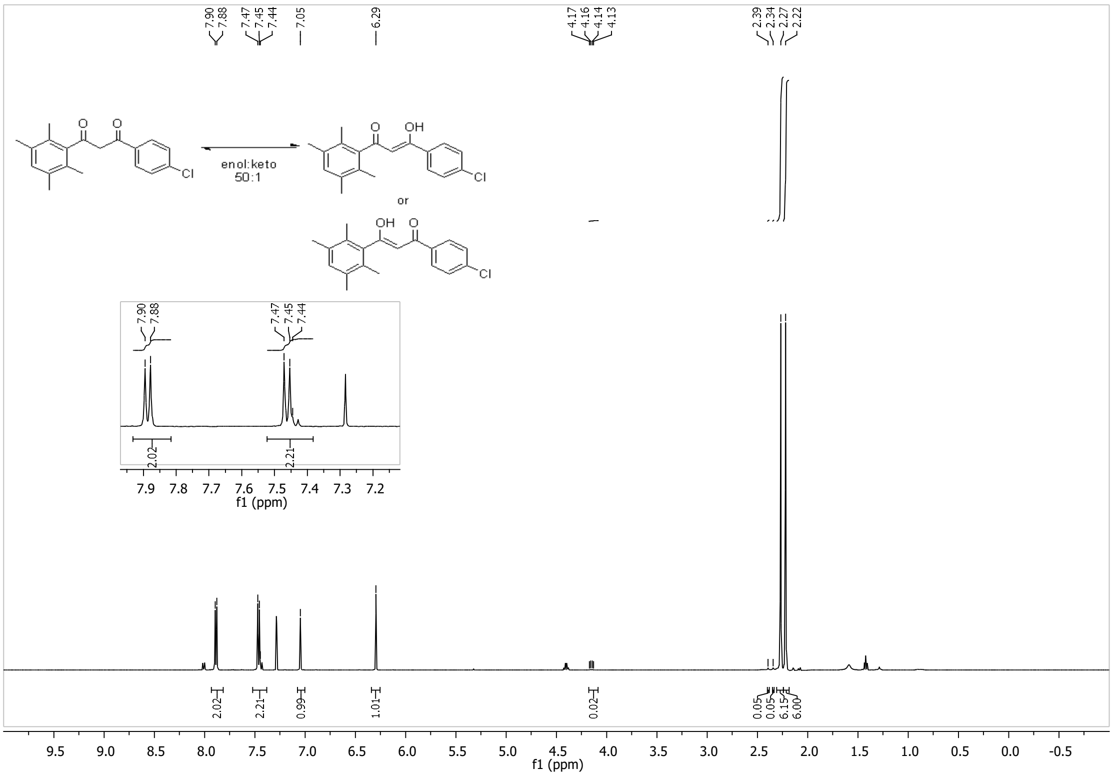

COSY (500 MHz, $CDCl_3$) of 1-(4-chlorophenyl)-3-(2,3,5,6-tetramethylphenyl)propane-1,3-dione **19a**.

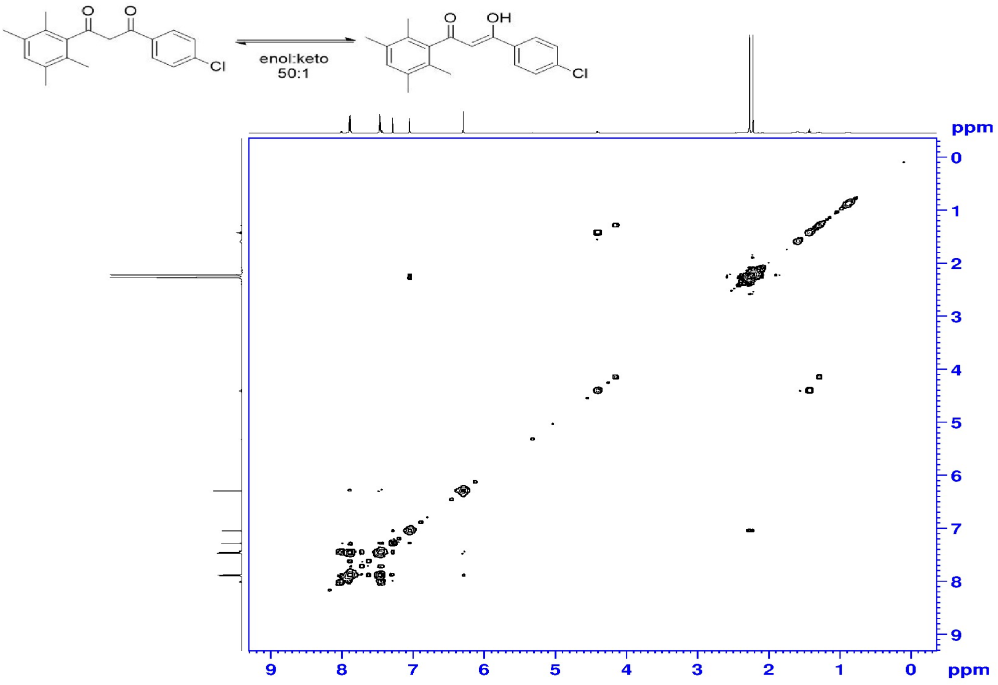

HSQC (126 MHz, $CDCl_3$) of 1-(4-chlorophenyl)-3-(2,3,5,6-tetramethylphenyl)propane-1,3-dione **19a**.

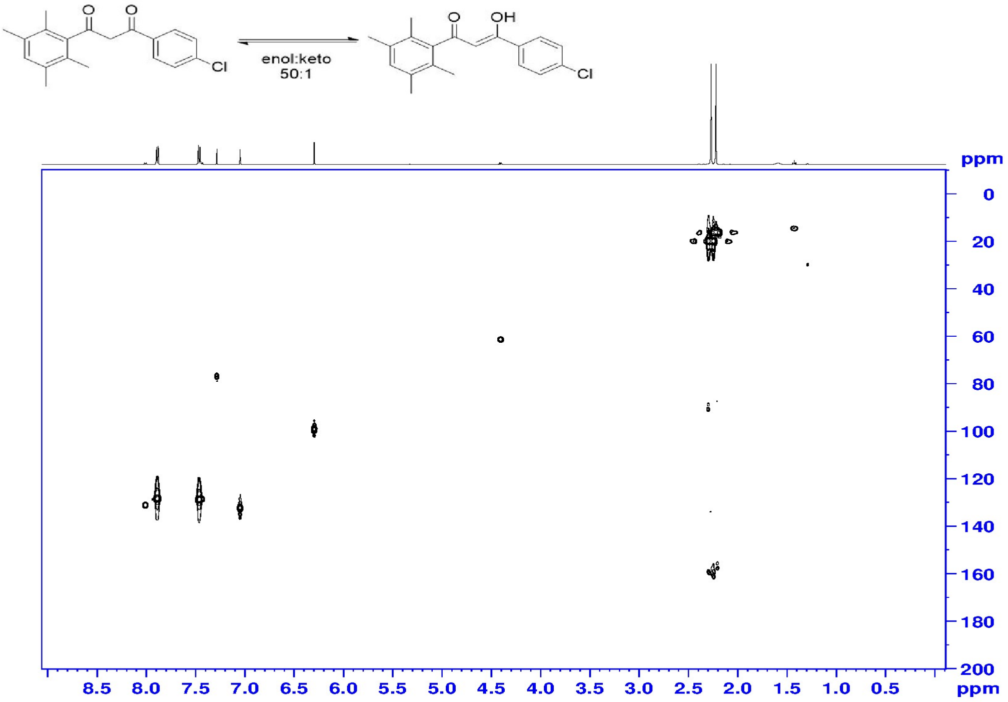

HMBC (126 MHz, $CDCl_3$) of 1-(4-chlorophenyl)-3-(2,3,5,6-tetramethylphenyl)propane-1,3-dione **19a**.

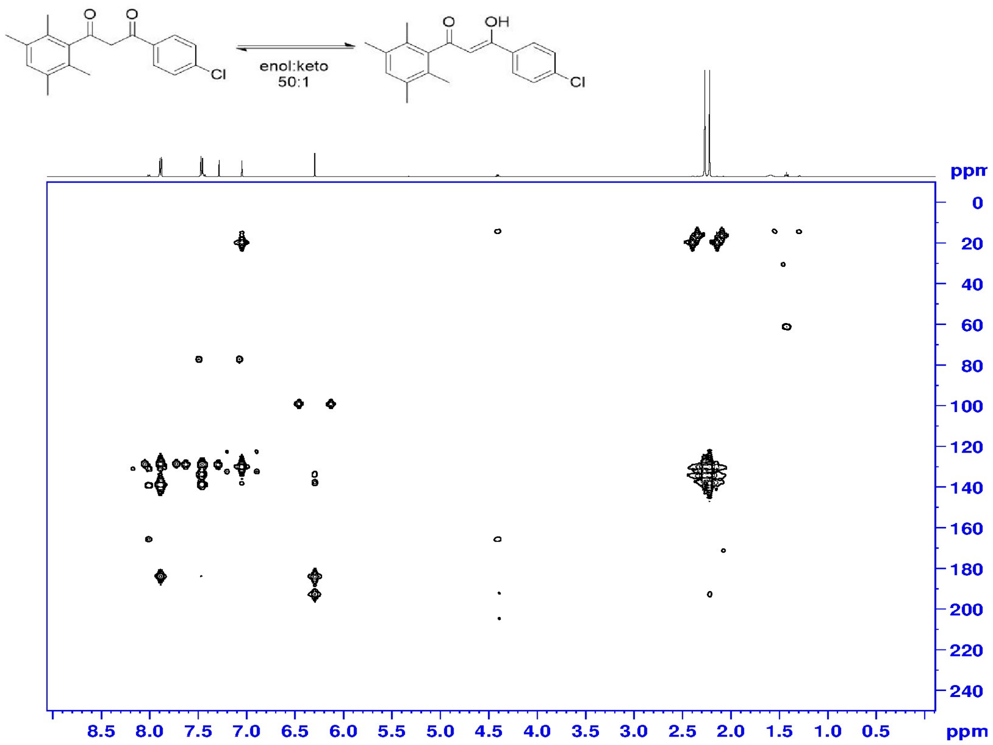

$^{13}C\{^{1}H\}$ NMR (126 MHz, $CDCl_3$) of 1-(4-chlorophenyl)-3-(2,3,5,6-tetramethylphenyl)propane-1,3-dione **19a**.

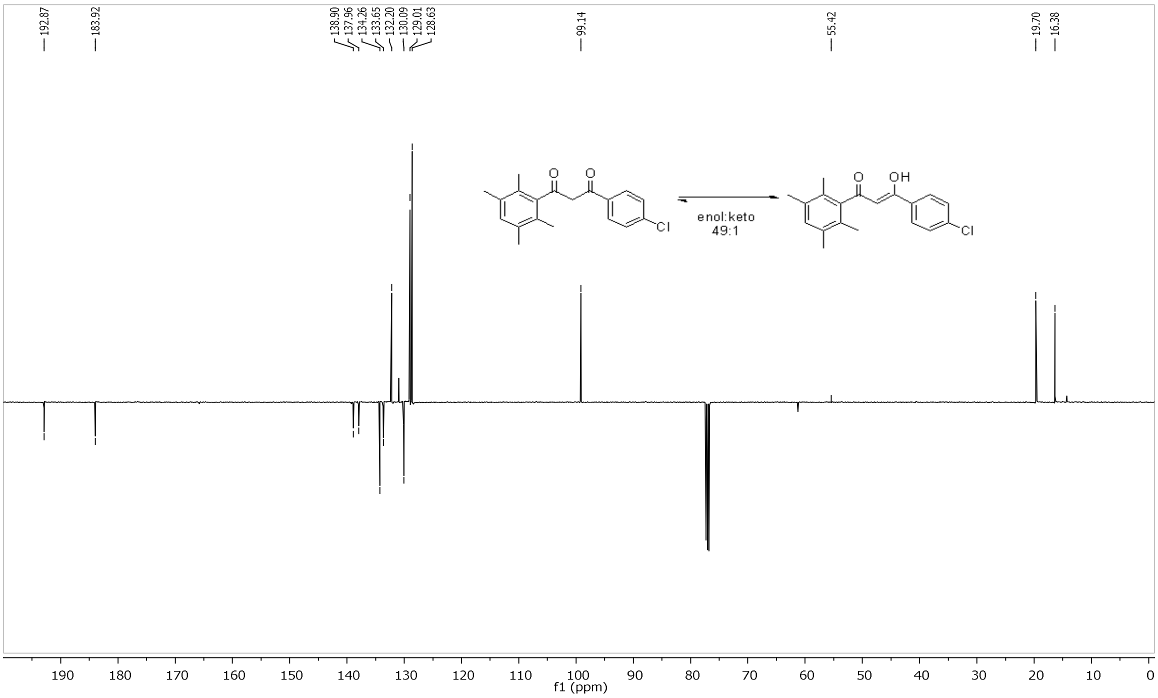

HPLC of 1-(4-chlorophenyl)-3-(2,3,5,6-tetramethylphenyl)propane-1,3-dione **19a**.

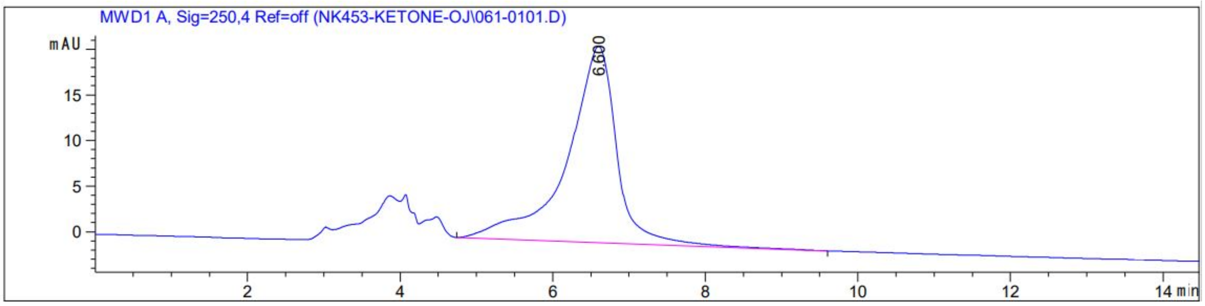

Signal 1: MWD1 A, Sig=250,4 Ref=off

Peak #	RetTime [min]	Type	Width [min]	Area [mAU*s]	Height [mAU]	Area %
1	6.600	BB	0.6397	977.23236	21.52334	100.0000
Totals :				977.23236	21.52334	

3-(4-Chlorophenyl)-3-hydroxy-1-(2,3,5,6-tetramethylphenyl)propan-1-one 19b.

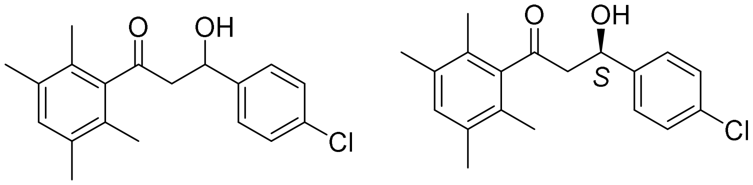

This compound is novel.

Synthesis of a racemic standard: (*R,R*)-3C-Tethered Ru(II)-TsDPEN catalyst (0.8mg, 0.0013 mmol, 0.5 mol%) and (*S,S*)-3C-tethered Ru(II)-TsDPEN catalyst (0.8 mg, 0.0013 mmol, 0.5 mol%) were added to FA: TEA (5:2 azeotropic mixture, 0.384 mL) at rt and the mixture was stirred under a nitrogen atmosphere for 15 minutes; after which 1-(4-chlorophenyl)-3-(2,3,5,6-tetramethylphenyl)propane-1,3-dione (80 mg, 0.25 mmol) was added. The reaction mixture was stirred under a nitrogen atmosphere and followed by TLC (5:1 hexane: EtOAc). After 48 h, the reaction was quenched using saturated $NaHCO_3$ solution (20 mL). EtOAc (20 mL) was added, and the organic layer was separated. The aqueous layer was extracted with EtOAc (3 x 20 mL) and the combined organic layers were dried ($MgSO_4$) and filtered. The solvent was removed to give the crude product. The product was isolated via flash chromatography on silica eluted with 0-50% EtOAc in petroleum ether to give 3-(4-chlorophenyl)-3-hydroxy-1-(2,3,5,6-tetramethylphenyl)propan-1-one **19b** as a white solid (72.6 mg, 0.229 mmol, 90%); ν_{max} 3376, 2961, 2922, 1905 cm^{-1}; TLC: Rf ca 0.25 (4:1 hexane: EtOAc), strong UV and $KMnO_4$; Mp: 113.8°C; HRMS (ESI+) *m/z*: [M+Na]+ Calcd for $C_{19}H_{21}ClNaO_2$ 339.1122; Found 339.1114; 2.5 ppm error; ^1H NMR (400 MHz, $CDCl_3$): δ 7.44 – 7.31 (4H, m, ArH), 7.00 (1H, s, ArH), 5.37 (1H, m, *J* = 3.8, ArCH), 3.64 (1H, s, OH), 3.12-3.08 (2H, m, CH_2), 2.23 (6H, s, CH_3), 2.10 (6H, s, CH_3); $^{13}C\{^1H\}$ NMR (101 MHz, $CDCl_3$): δ 211.9 (C), 141.7 (C), 141.2 (C), 134.6 (C), 133.4 (C), 132.0 (CH), 128.7 (CH), 127.9 (C), 127.2 (CH), 69.0 (CH), 53.6 (CH_2), 19.4 (CH_3), 15.9 (CH_3); *m/z* (ES-API+) 339.1 (M^+ + Na, 100%); Enantiomeric excess and conversion determined by HPLC analysis (Chiralpak OJ, 30 cm x 6 mm column, hexane:iPrOH 90:10, 1.0 mL/min, T = 25 °C) ketone 6.6 min, *R* and *S* isomer 7.1 min and 8.5 min. Configuration assigned by analogy.

(R)-3-(4-Chlorophenyl)-3-hydroxy-1-(2,3,5,6-tetramethylphenyl)propan-1-one **19b.**

(R,R)-3C-tethered Ru(II)-TsDPEN catalyst (1.96 mg, 0.0032 mmol, 1 mol%) was added to FA: TEA (5:2 azeotropic mixture, 0.64 mL) at rt and the mixture was stirred under a nitrogen atmosphere for 10-15 minutes; after which 1-(4-chlorophenyl)-3-(2,3,5,6-tetramethylphenyl)propane-1,3-dione **19a** (100 mg, 0.317 mmol) was added. The reaction mixture was stirred under a nitrogen atmosphere for 48 h. The reaction was followed by TLC (9:1 hexane: EtOAc). After 48 h, the reaction was quenched using saturated $NaHCO_3$ solution (20 mL). EtOAc (20 mL) was added, and the organic layer was separated. The aqueous layer was extracted with EtOAc (3 x 20 mL) and the combined organic layers were dried ($MgSO_4$) and filtered. The solvent was removed to give the crude product. The product was isolated via flash chromatography on silica eluted with 0-50% EtOAc in petroleum ether to give (R)-3-(4-chlorophenyl)-3-hydroxy-1-(2,3,5,6-tetramethylphenyl)propan-1-one **19b** as a white solid (70.4 mg, 0.22 mmol, 70%). The reaction was also followed by HPLC (Chiralpak OJ, 30 cm x 6 mm column, hexane:iPrOH 90:10, 1.0 mL/min, T = 25°C); $[\alpha]_D^{26}$ + 35.8 (c 0.06 in $CHCl_3$); (after 48 h, 100% conversion, >99% ee (R)).

^{1}H NMR (400 MHz, $CDCl_3$) of 3-(4-chlorophenyl)-3-hydroxy-1-(2,3,5,6-tetramethylphenyl)propan-1-one **19b.**

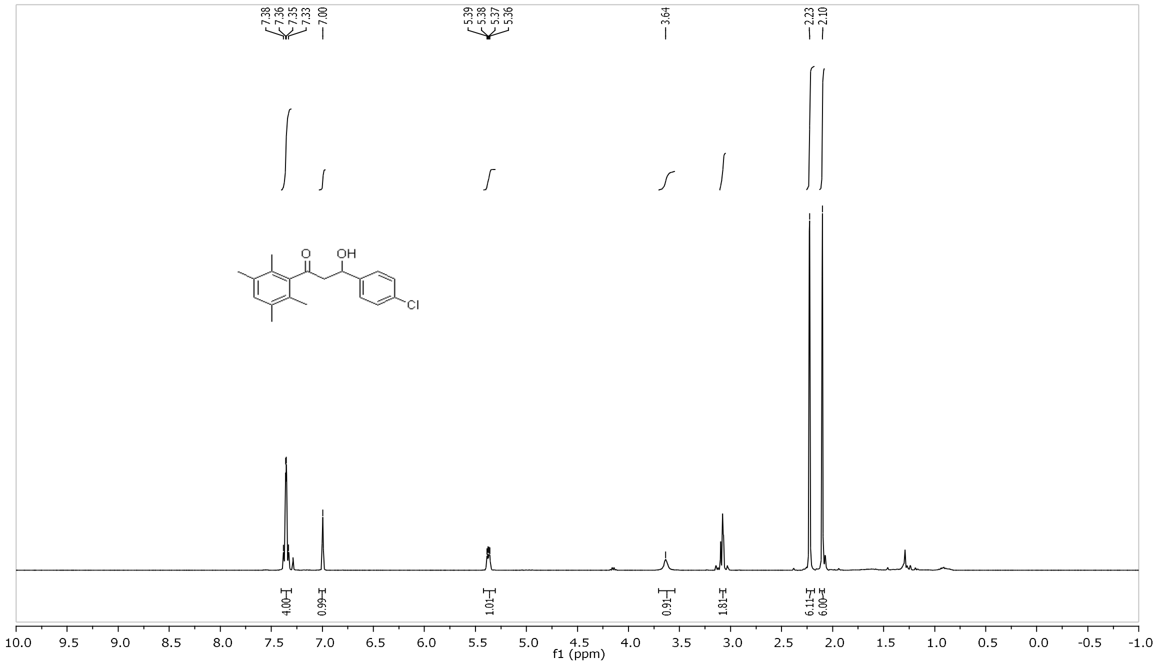

COSY (400 MHz, $CDCl_3$) of 3-(4-chlorophenyl)-3-hydroxy-1-(2,3,5,6-tetramethylphenyl)propan-1-one **19b.**

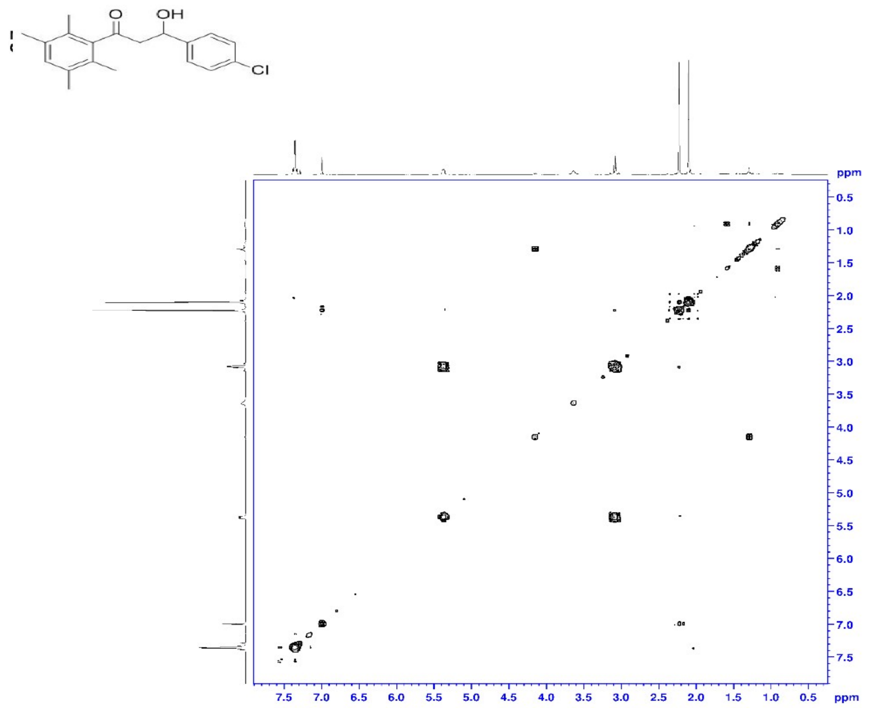

HSQC (101 MHz, $CDCl_3$) of 3-(4-chlorophenyl)-3-hydroxy-1-(2,3,5,6-tetramethylphenyl)propan-1-one **19b.**

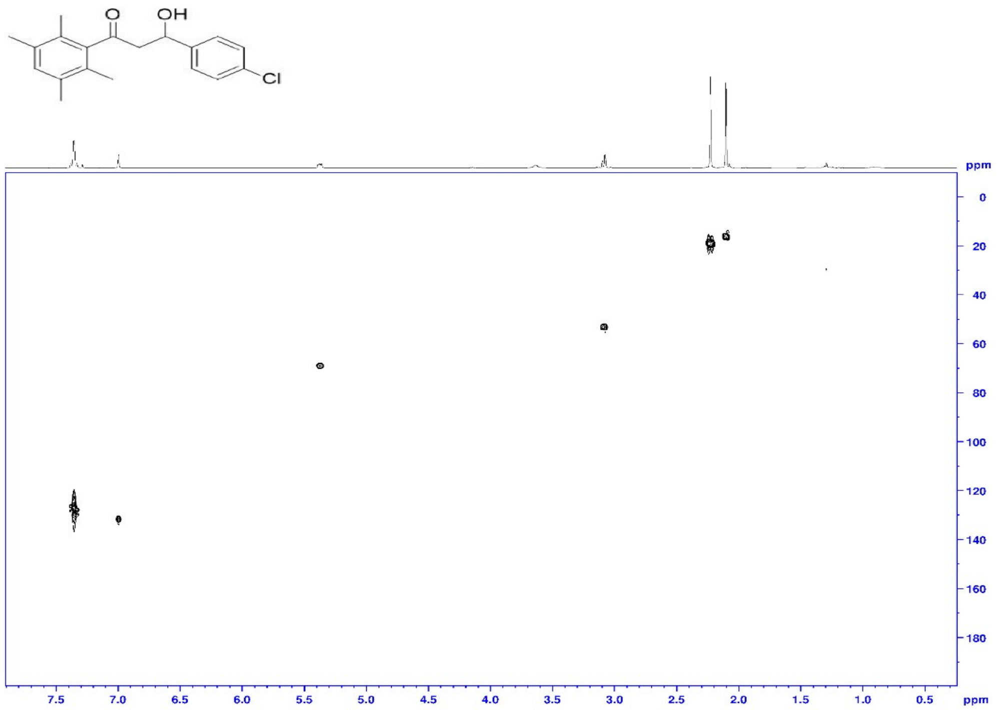

HMBC (101 MHz, $CDCl_3$) of 3-(4-chlorophenyl)-3-hydroxy-1-(2,3,5,6-tetramethylphenyl)propan-1-one **19b.**

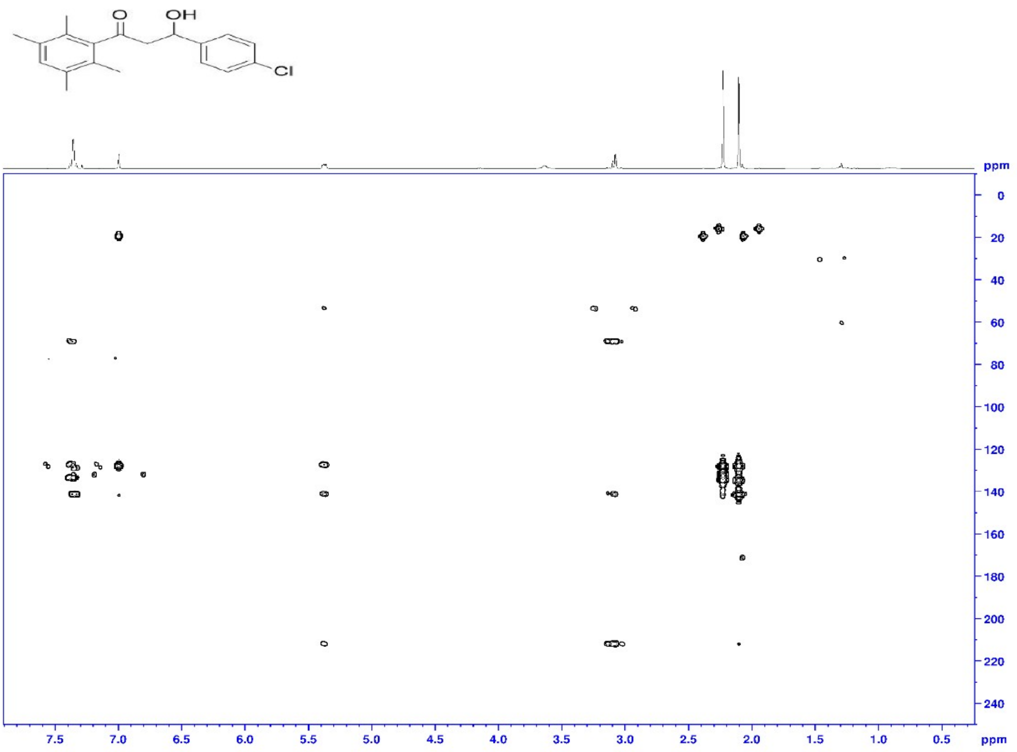

$^{13}C\{^1H\}$ NMR (101 MHz, $CDCl_3$) of 3-(4-chlorophenyl)-3-hydroxy-1-(2,3,5,6-tetramethylphenyl)propan-1-one **19b.**

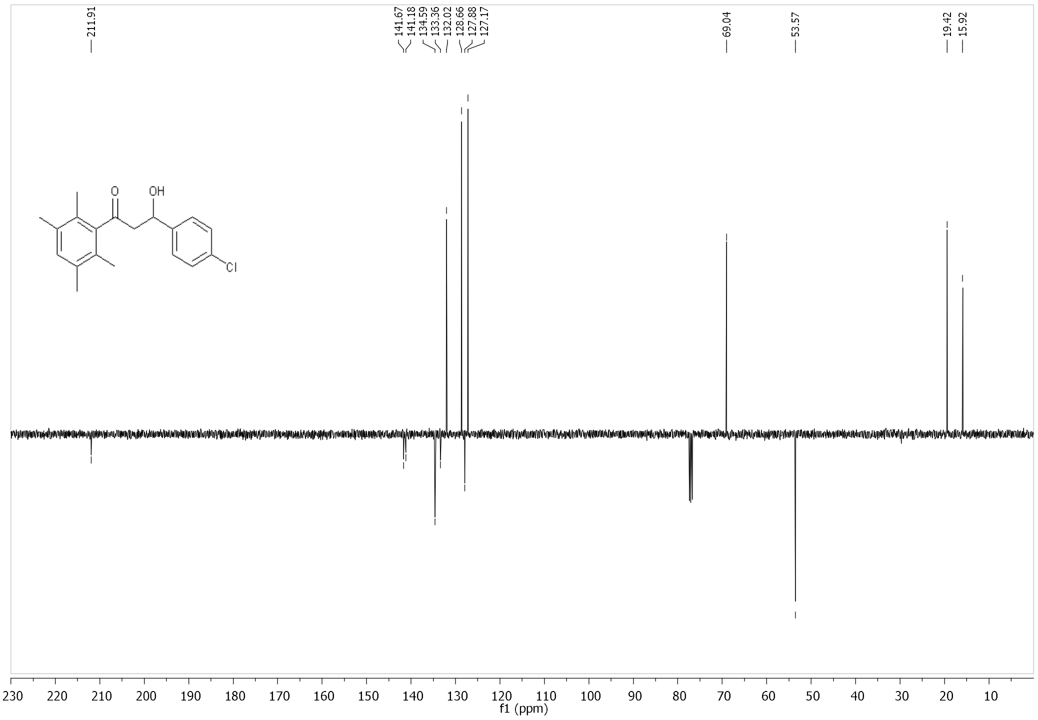

Racemic of 3-(4-chlorophenyl)-3-hydroxy-1-(2,3,5,6-tetramethylphenyl)propan-1-one **19b**.:

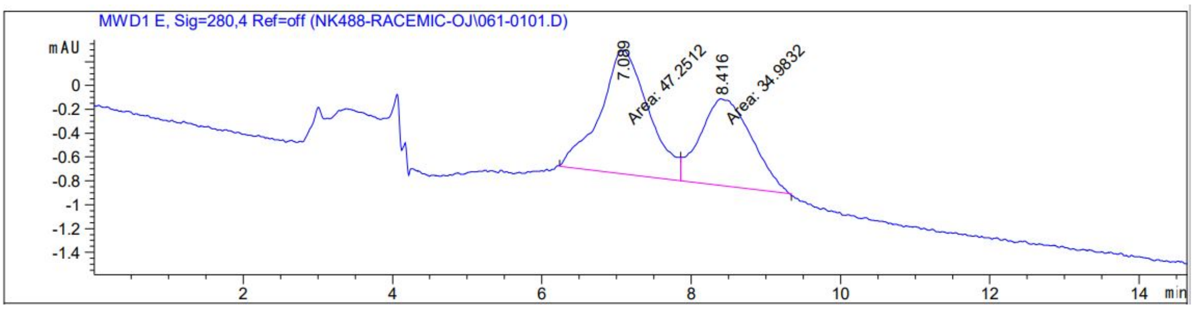

Signal 1: MWD1 A, Sig=250,4 Ref=off

Peak #	RetTime [min]	Type	Width [min]	Area [mAU*s]	Height [mAU]	Area %
1	7.088	BB	0.5473	92.99154	2.16422	62.7721
2	8.463	MM	0.7572	55.14995	1.21386	37.2279
Totals :				148.14149	3.37808	

HPLC of 3-(4-chlorophenyl)-3-hydroxy-1-(2,3,5,6-tetramethylphenyl)propan-1-one **19b.**

(*R,R*)-3C-tethered Ru(II)-TsDPEN catalyst (after 48 h, 100% conversion, >99% ee (*R*)).

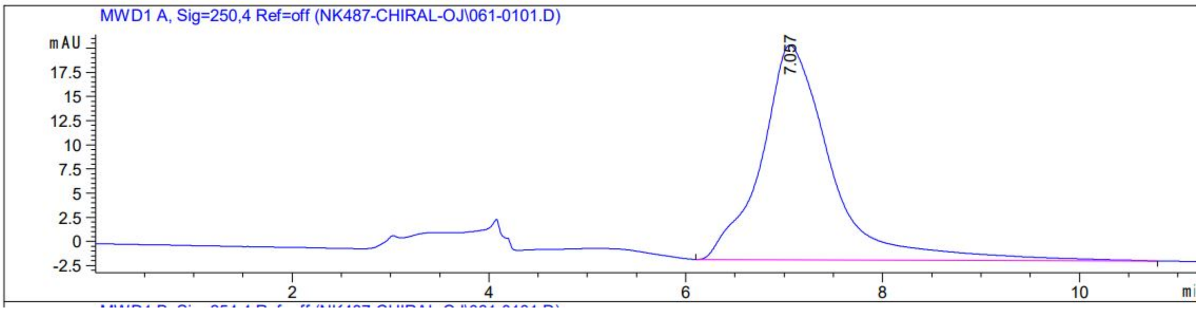

Signal 1: MWD1 A, Sig=250,4 Ref=off

Peak #	RetTime [min]	Type	Width [min]	Area [mAU*s]	Height [mAU]	Area %
1	7.057	BB	0.7327	1099.06458	22.23444	100.0000
Totals :				1099.06458	22.23444	

1-(2-Chlorophenyl)-3-(2,3,5,6-tetramethylphenyl)propane-1,3-dione 20a.

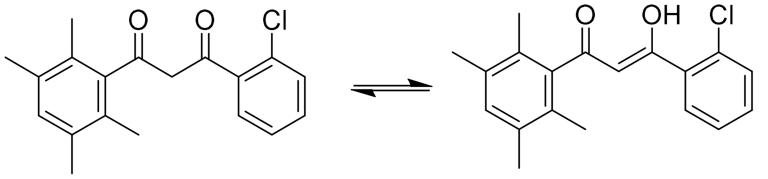

This compound is novel. To a solution of sodium hydride (455 mg, 60% dispersion in mineral oil, 11.35 mmol) in THF (4 mL) at 0 °C was added dropwise a solution of 1-(2,3,5,6-tetramethylphenyl)ethan-1-one **7** (350 mg, 1.98 mmol) in THF (4 mL). The reaction mixture was stirred under a nitrogen atmosphere at 0 °C for 30 min and then stirred under a nitrogen atmosphere at rt for 30 min, after which ethyl 2-chlorobenzoate (2.09 g, 11.3 mmol) was added dropwise at 0 °C. The reaction mixture was refluxed at 66 °C and left stirring under the nitrogen atmosphere overnight. The reaction was followed by TLC (4:1 hexane: EtOAc). The mixture was quenched by 2M HCl solution (20 mL). EtOAc (20 mL) was added, and the organic layer was separated. The aqueous layer was extracted with EtOAc (3 × 20 mL), and the combined organic layers were washed with saturated $NaHCO_3$ solution (2 × 20 mL) and brine (20 mL), dried ($MgSO_4$) and filtered. Solvent was removed to give the crude product. The product was isolated via flash chromatography on silica eluted with 0.5% EtOAc in hexane to give 1-(2-chlorophenyl)-3-(2,3,5,6-tetramethylphenyl)propane-1,3-dione **20a** as a yellow solid (526 mg, 1.67 mmol, 74%). TLC: Rf ca 0.31 (9:1 hexane: EtOAc), strong UV and $KMnO_4$; Mp: 75 °C; HRMS (ESI+) *m/z*: [M+Na]+ Calcd for $C_{19}H_{19}ClNaO_2$ 337.0966; Found 337.0959; 2.0 ppm error; ν_{max} 2991, 2967, 2922, 2859, 1730 cm^{-1}; enol: keto = 80:20; ^1H NMR (500 MHz, $CDCl_3$): δ 15.98 (0.8H, s, OH), 7.72 (1H, dd, *J* = 7.4, 1.4, ArH), 7.49 – 7.45 (1H, m, ArH), 7.44 – 7.36 (2H, m, CH_2), 7.04 (1H, s, ArH), 6.24 (0.8H, br. S, CH of enol form), 4.12-4.10 (0.4H, m, CH_2 of keto form), 2.39 (1.2H, s, CH_3 of keto form), 2.38 (1.2H, s, CH_3 of keto form), 2.27 (4.8H, s, CH_3 of enol form), 2.25 (4.8H, s, CH_3 of enol form); $^{13}C\{^1H\}$ NMR (126 MHz, $CDCl_3$): δ 191.2 (C), 186.7 (C), 137.5 (C), 136.0 (C), 134.2 (C), 132.3 (CH), 131.9 (C), 131.8 (CH), 130.8 (CH), 130.3 (C), 130.2 (CH), 127.0 (CH), 104.6 (CH), 64.3 (CH_2), 19.7 (CH_3), 16.4 (CH_3); *m/z* (ES-API+) 337.1 (M^+ + Na, 100%); Enantiomeric excess and conversion determined by HPLC analysis (Chiralpak OD-H, 30 cm x 6 mm column,

hexane:iPrOH 95:05, 0.6 mL/min, T = 25°C) ketone 6.6 min, *S* and *R* isomer 10.03 min and 9.04 min.

^{1}H NMR (500 MHz, $CDCl_3$) of 1-(2-chlorophenyl)-3-(2,3,5,6-tetramethylphenyl)propane-1,3-dione **20a.**

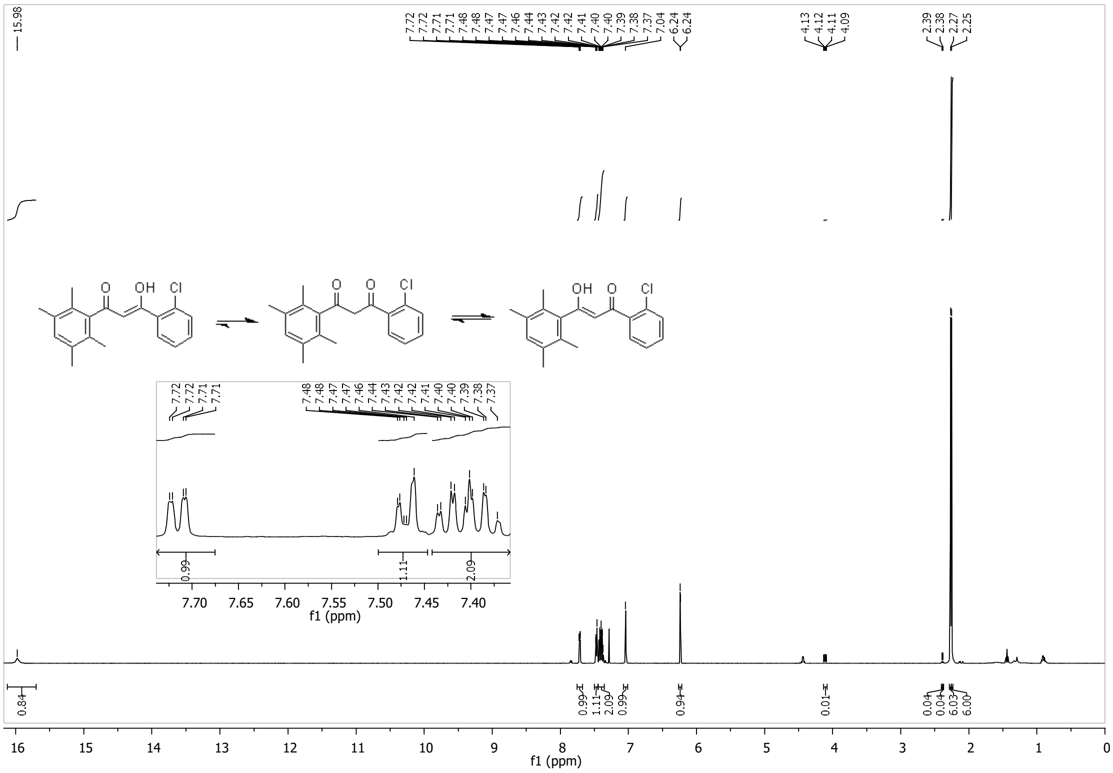

COSY (500 MHz, $CDCl_3$) of 1-(2-chlorophenyl)-3-(2,3,5,6-tetramethylphenyl)propane-1,3-dione **20a.**

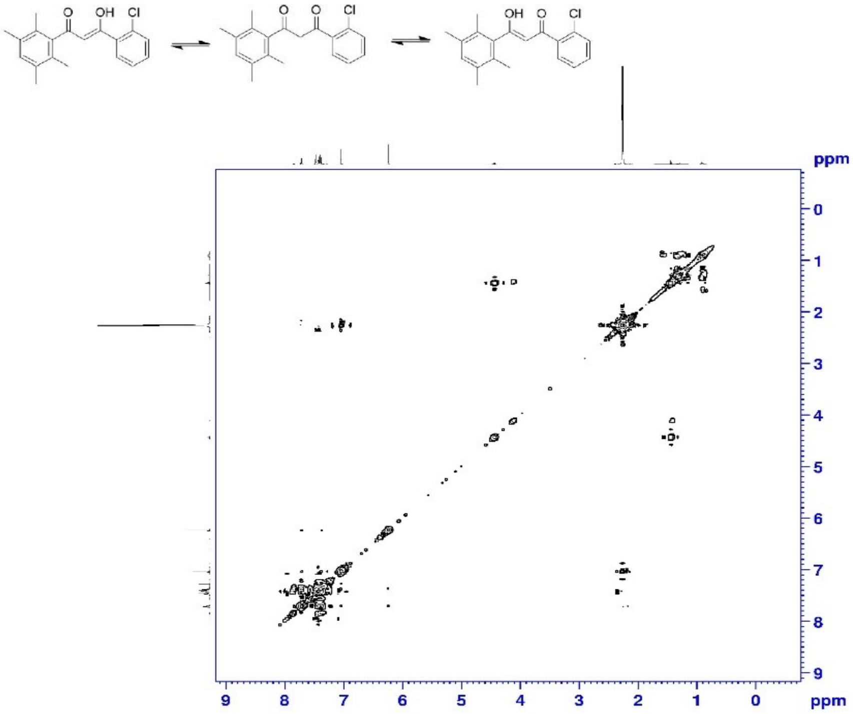

HMBC (126 MHz, $CDCl_3$) of 1-(2-chlorophenyl)-3-(2,3,5,6-tetramethylphenyl)propane-1,3-dione **20a.**

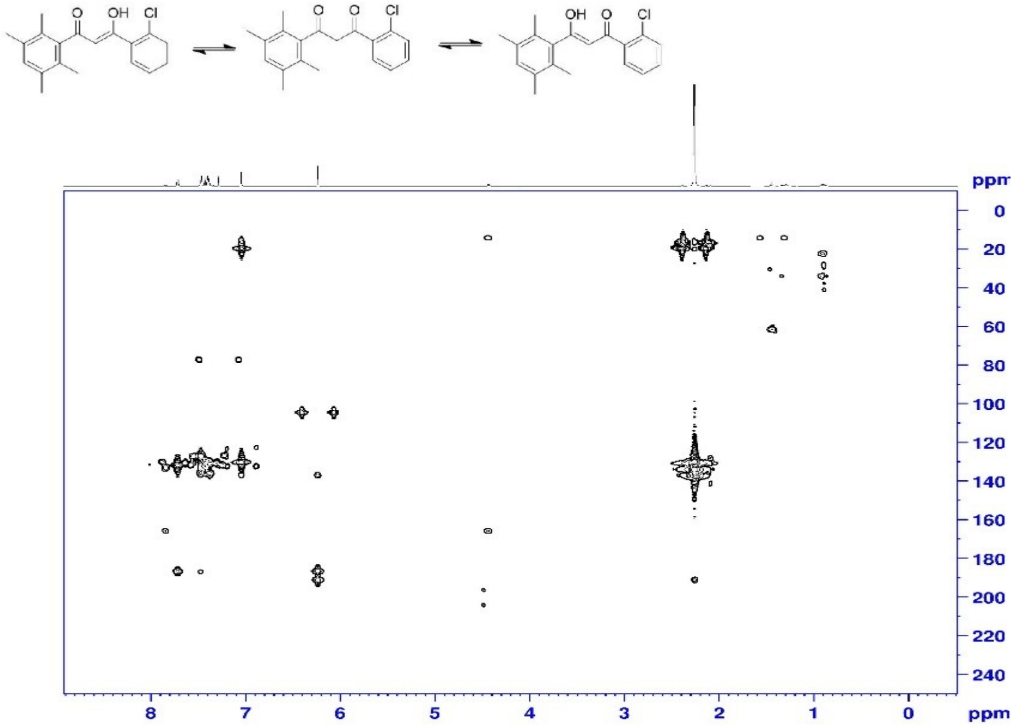

HSQC (126 MHz, $CDCl_3$) of 1-(2-chlorophenyl)-3-(2,3,5,6-tetramethylphenyl)propane-1,3-dione **20a.**

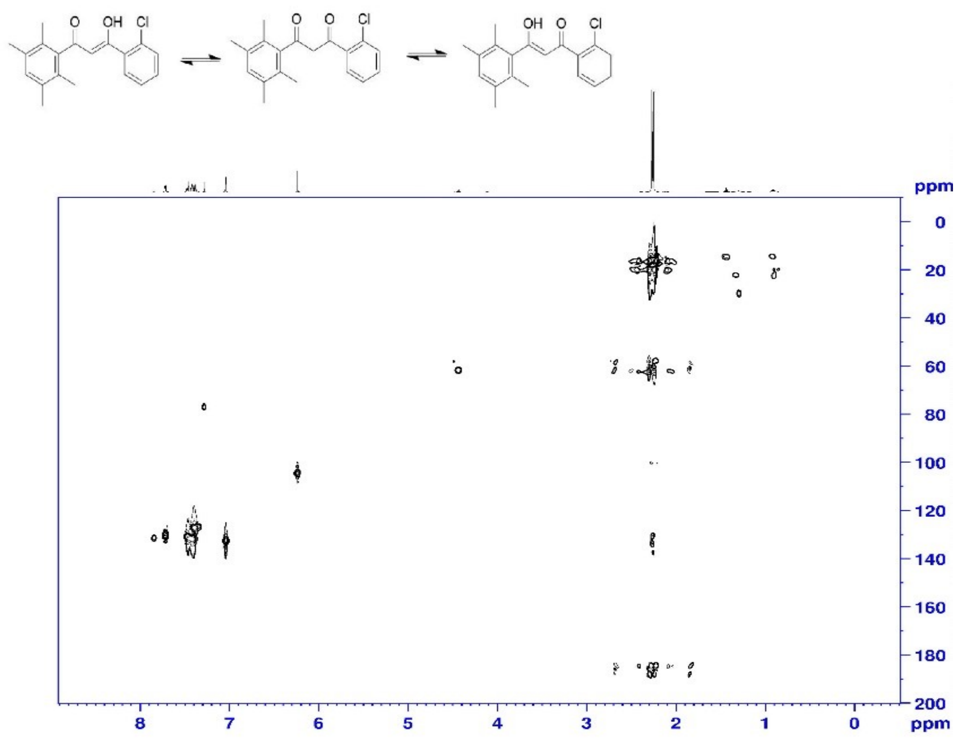

$^{13}C\{^1H\}$ NMR (126 MHz, $CDCl_3$) of 1-(2-chlorophenyl)-3-(2,3,5,6-tetramethylphenyl)propane-1,3-dione **20a.**

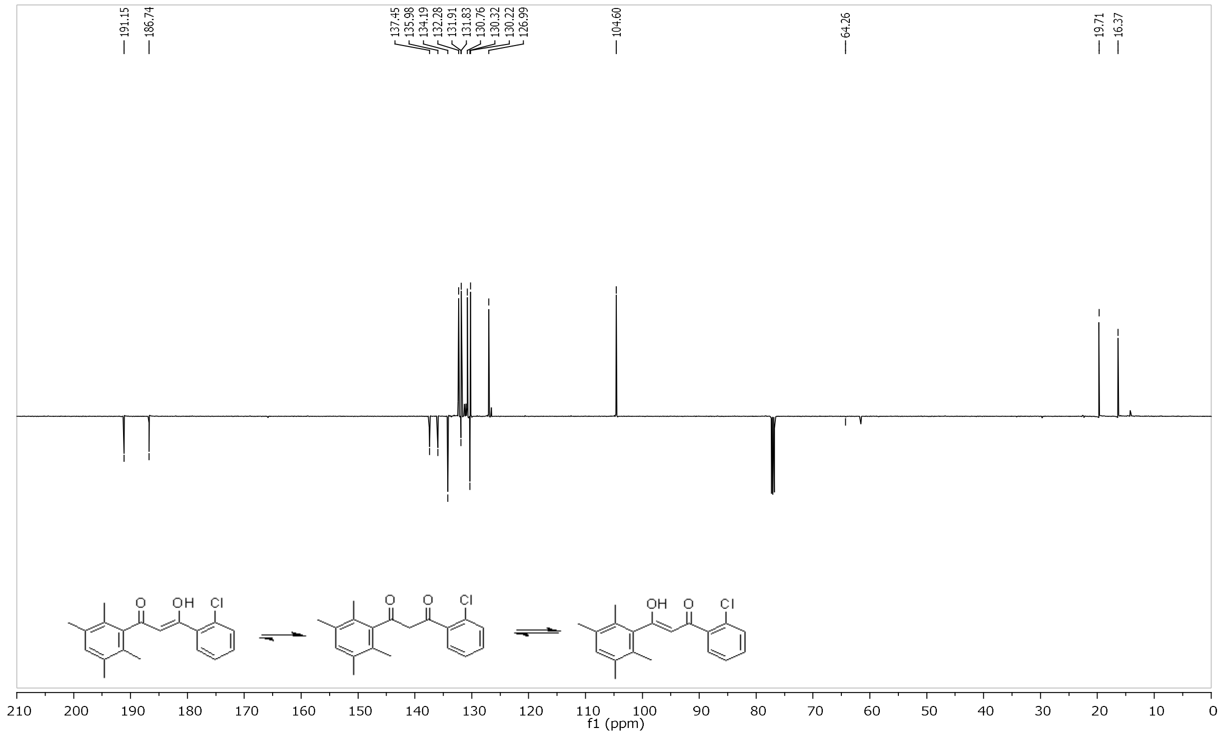

HPLC of 1-(2-chlorophenyl)-3-(2,3,5,6-tetramethylphenyl)propane-1,3-dione **20a.**

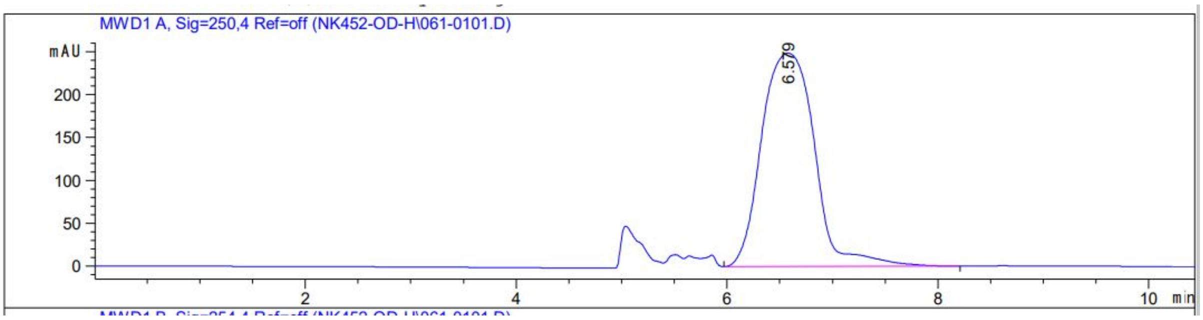

Signal 1: MWD1 A, Sig=250,4 Ref=off

Peak #	RetTime [min]	Type	Width [min]	Area [mAU*s]	Height [mAU]	Area %
1	6.579	BB	0.5699	8695.70898	248.69838	100.0000

Totals : 8695.70898 248.69838

3-(2-Chlorophenyl)-3-hydroxy-1-(2,3,5,6-tetramethylphenyl)propan-1-one 20b.

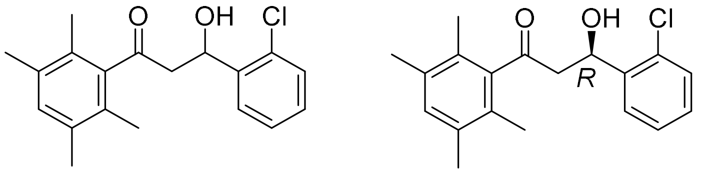

This compound is novel.

Synthesis of a racemic standard: (*R,R*)-3C-Tethered Ru(II)-TsDPEN catalyst (0.8 mg, 0.0013 mmol, 0.5 mol%) and (*S,S*)-3C-tethered Ru(II)-TsDPEN catalyst (0.8 mg, 0.0013 mmol, 0.5 mol%) were added to FA:TEA (5:2 azeotropic mixture, 0.13 mL) at rt and the mixture was stirred under a nitrogen atmosphere for 15 minutes; after which 1-(2-chlorophenyl)-3-(2,3,5,6-tetramethylphenyl)propane-1,3-dione **20a** (80 mg, 0.25 mmol) was added after dissolving in DCM (0.2 mL). The reaction mixture was stirred under a nitrogen atmosphere and followed by TLC (5:1 hexane: EtOAc). After 48 h, the reaction was quenched using saturated $NaHCO_3$ solution (20 mL). EtOAc (20 mL) was added, and the organic layer was separated. The aqueous layer was extracted with EtOAc (3 x 20 mL) and the combined organic layers were dried ($MgSO_4$) and filtered. The solvent was removed to give the crude product. The product was isolated via flash chromatography on silica eluted with 0-20% EtOAc in petroleum ether to give 3-(2-chlorophenyl)-3-hydroxy-1-(2,3,5,6-tetramethylphenyl)propan-1-one **20b** as a white solid (63.1 mg, 0.199 mmol, 78%). TLC: Rf ca 0.3 (5:1 hexane: EtOAc), strong UV and PMA active; Mp: 110.3 °C; HRMS (ESI+) *m/z*: [M+Na]+ Calcd for $C_{19}H_{21}ClNaO_2$ 339.1122; Found 339.1119; 1.1 ppm error; *m/z* (ES-API+) 339.1 (M^+ + Na, 100%); ν_{max} 3355, 2967, 2916, 2857 cm^{-1}; ^1H NMR (400 MHz, $CDCl_3$): δ 7.62 (1H, d, *J* = 7.9, ArH), 7.24 (2H, t, *J* = 7.4, ArH), 7.13 (1H, t, *J* = 7.6, ArH), 6.88 (1H, s, ArH), 5.65 (1H, d, *J* = 9.4, ArCH), 3.76 (1H, s, OH), 3.19 (1H, d, *J* = 18.9, C1$H_A$$H_B$), 2.80 (1H, dd, *J* = 18.9, 9.6, C1$H_A$$H_B$), 2.12 (6H, s, CH_3), 2.03 (6H, s, CH_3); $^{13}C\{^1H\}$ NMR (101 MHz, $CDCl_3$): δ 212.6 (C), 140.0 (C), 134.5 (C), 131.9 (CH), 131.1 (C), 129.3 (CH), 128.5 (CH), 127.9 (C), 127.2 (CH), 127.1 (CH), 66.5 (CH), 51.8 (CH_2), 19.4 (CH_3), 15.9 (CH_3); Enantiomeric excess and conversion determined by HPLC analysis (Chiralpak OD_H, 30 cm x 6 mm column, hexane:iPrOH 95:05, 0.6 mL/min, T = 25°C) ketone 6.58 min, *R* and *S* isomer 6.04 min and 10.26 min.

(*R*)-3-(2-Chlorophenyl)-3-hydroxy-1-(2,3,5,6-tetramethylphenyl)propan-1-one **20b..** (*R,R*)-3C-tethered Ru(II)-TsDPEN catalyst (2.0 mg, 0.0032 mmol, 1 mol%) was added to FA: TEA (5:2 azeotropic mixture, 0.16 mL) at rt and the mixture was stirred under a nitrogen atmosphere for 10-15 minutes; after which 1-(2-chlorophenyl)-3-(2,3,5,6-tetramethylphenyl)propane-1,3-dione **20a** (100 mg, 0.32 mmol) was in DCM (0.16 mL). The reaction mixture was stirred under a nitrogen atmosphere for 48 h. The reaction was followed by TLC (5:1 hexane: EtOAc). After 48 h, the reaction was quenched using saturated $NaHCO_3$ solution (20 mL). EtOAc (20 mL) was added, and the organic layer was separated. The aqueous layer was extracted with EtOAc (3 x 20 mL) and the combined organic layers were dried ($MgSO_4$) and filtered. The solvent was removed to give the crude product. The product was isolated via flash chromatography on silica eluted with 0-20% EtOAc in petroleum ether to give (*R*)-3-(2-chlorophenyl)-3-hydroxy-1-(2,3,5,6-tetramethylphenyl)propan-1-one **20b** as a white solid (77.6 mg, 0.24 mmol, 77%). The reaction was also followed by HPLC (Chiralpak OD_H, 30 cm x 6 mm column, hexane:iPrOH 95:5, 0.6 mL/min, T = 25°C); $[\alpha]_D^{26}$ +93.8 (c 0.056 in $CHCl_3$); (after 48 h, 100% conversion, 98% ee (*S*)).

^1H NMR (400 MHz, $CDCl_3$) of 3-(2-chlorophenyl)-3-hydroxy-1-(2,3,5,6-tetramethylphenyl)propan-1-one **20b.**

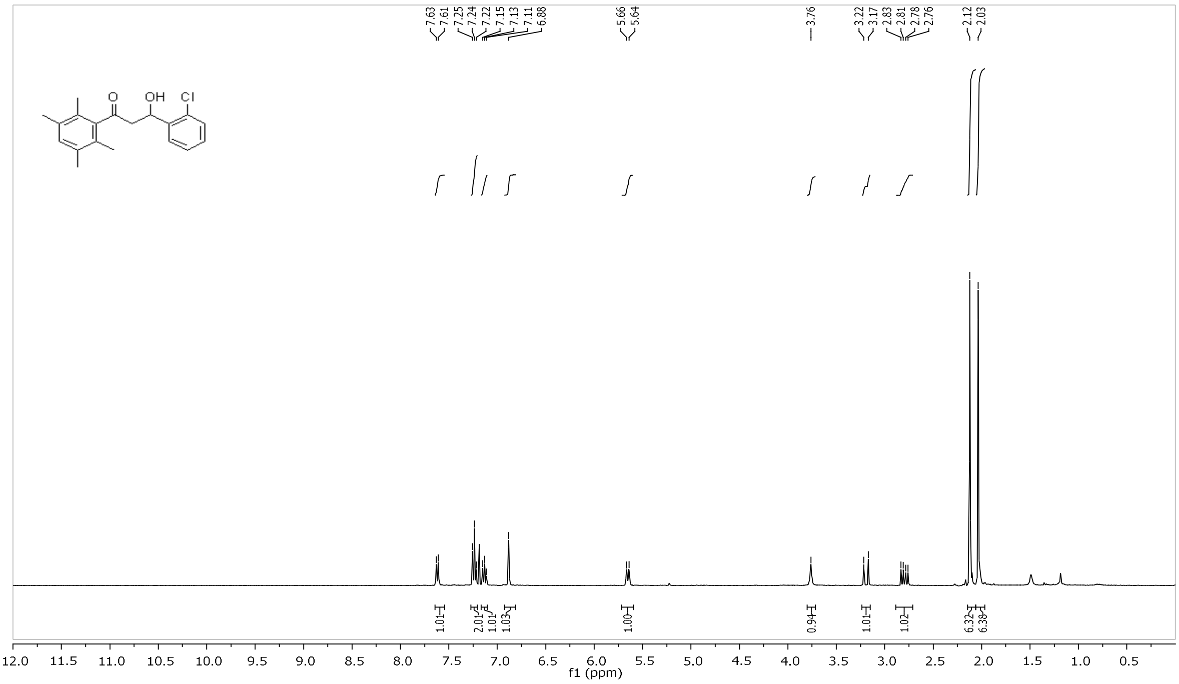

COSY (400 MHz, $CDCl_3$) of 3-(2-chlorophenyl)-3-hydroxy-1-(2,3,5,6-tetramethylphenyl)propan-1-one **20b.**

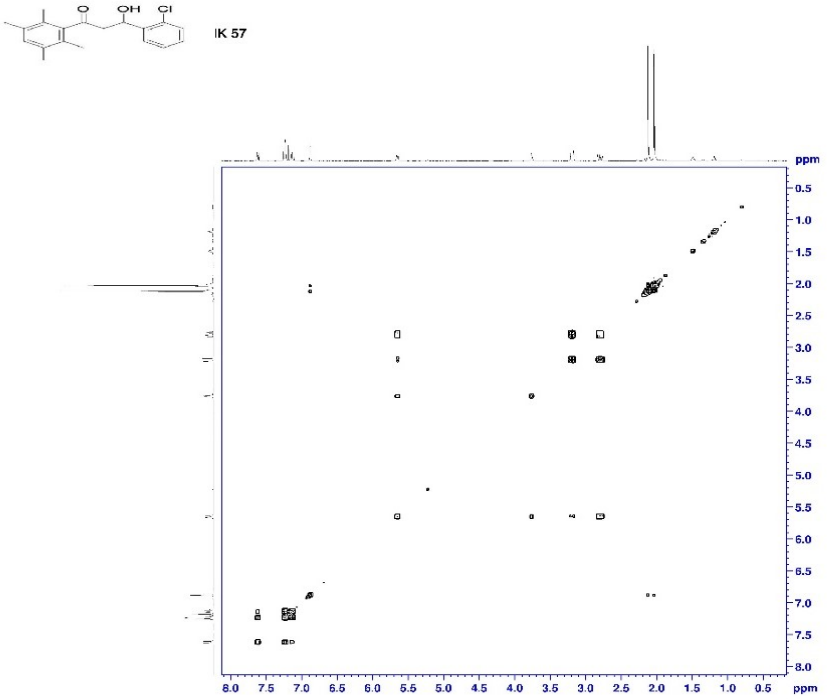

HSQC (101 MHz, $CDCl_3$) of 3-(2-chlorophenyl)-3-hydroxy-1-(2,3,5,6-tetramethylphenyl)propan-1-one **20b.**

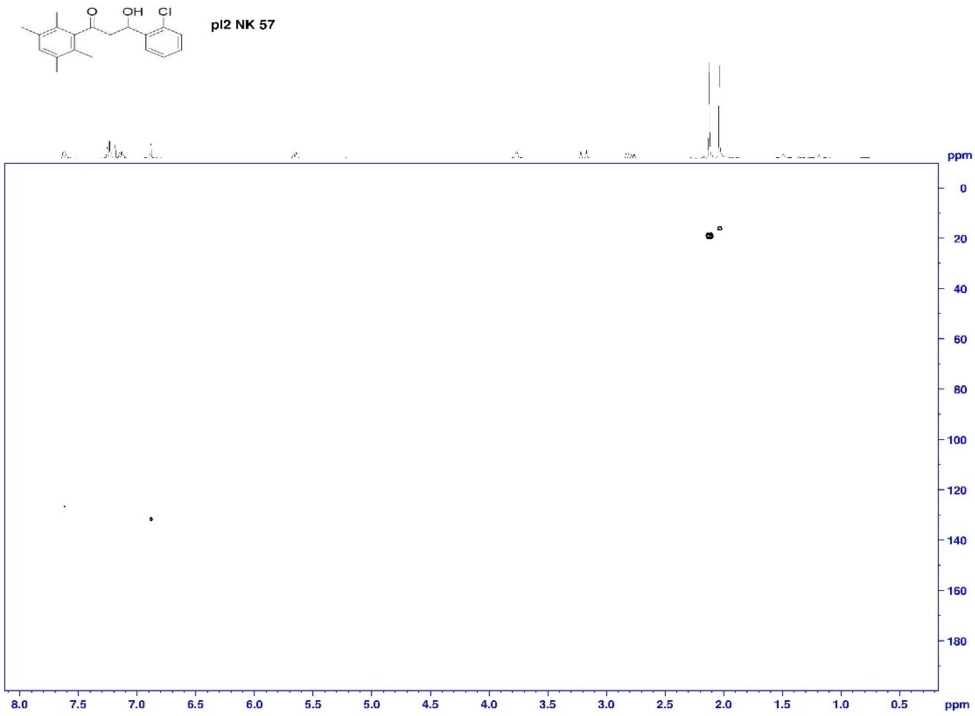

HMBC (101 MHz, $CDCl_3$) of 3-(2-chlorophenyl)-3-hydroxy-1-(2,3,5,6-tetramethylphenyl)propan-1-one **20b.**

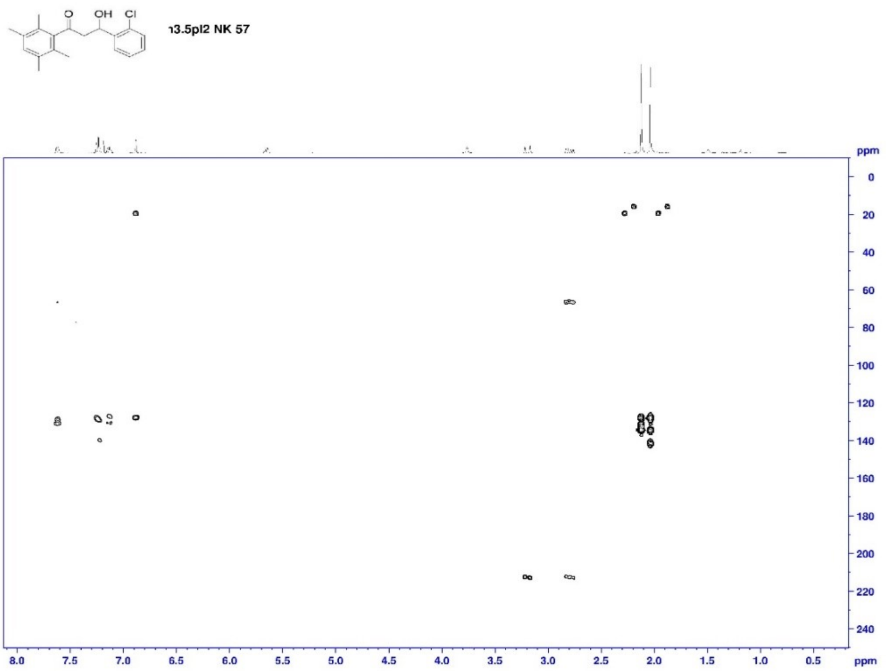

$^{13}C\{^1H\}$ (101 MHz, $CDCl_3$) of 3-(2-chlorophenyl)-3-hydroxy-1-(2,3,5,6-tetramethylphenyl)propan-1-one **20b.**

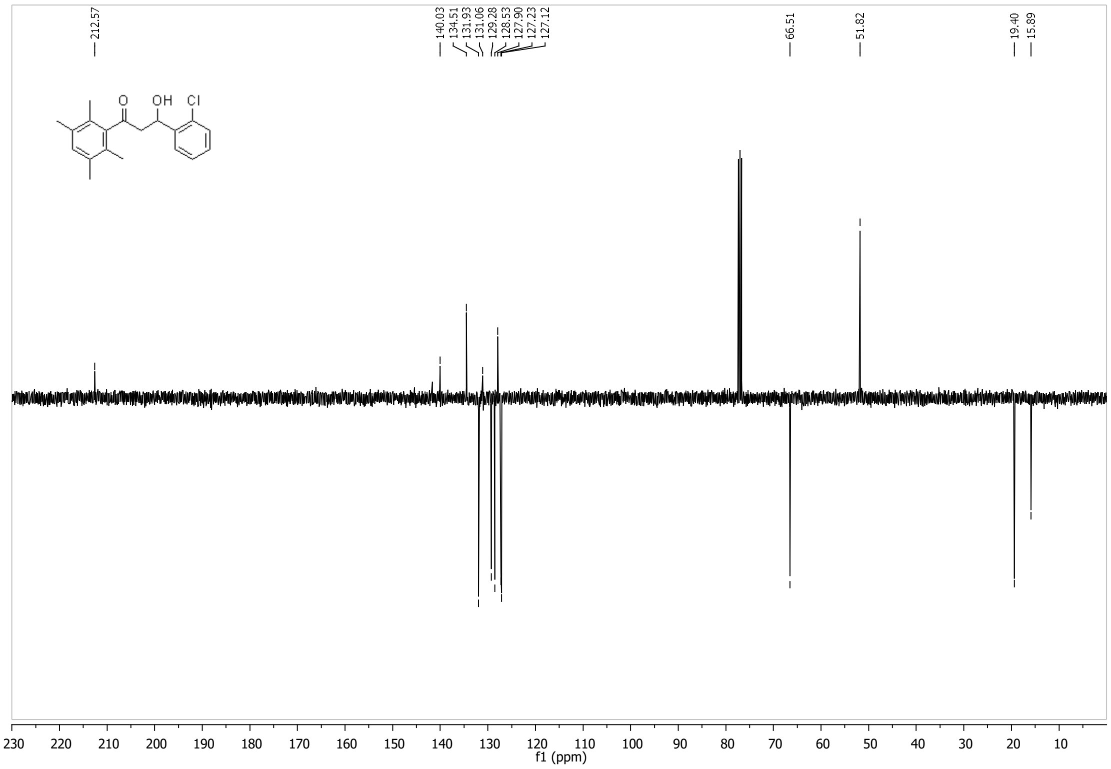

HPLC of racemic 3-(2-chlorophenyl)-3-hydroxy-1-(2,3,5,6-tetramethylphenyl)propan-1-one **20b.**

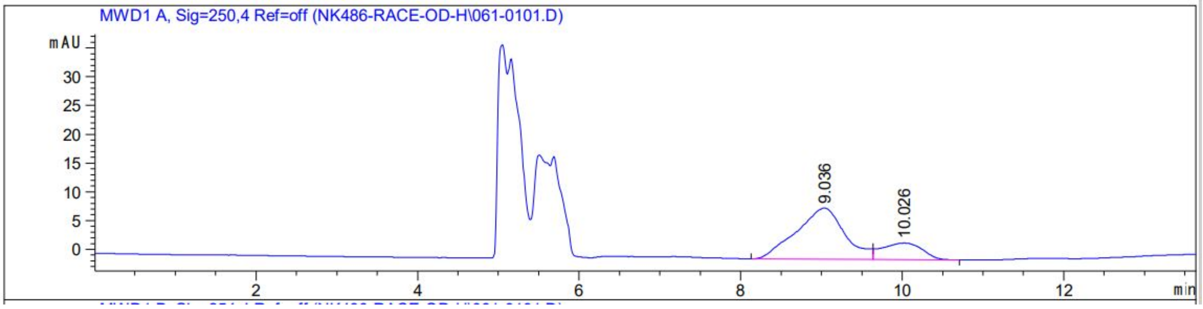

Signal 1: MWD1 A, Sig=250,4 Ref=off

Peak #	RetTime [min]	Type	Width [min]	Area [mAU*s]	Height [mAU]	Area %
1	9.036	BV	0.5693	372.09338	8.90926	78.7699
2	10.026	VB	0.4413	100.28658	2.90121	21.2301

Totals : 472.37996 11.81047

HPLC of 3-(2-chlorophenyl)-3-hydroxy-1-(2,3,5,6-tetramethylphenyl)propan-1-one **20b.**

(*R,R*)-3C-tethered Ru(II)-TsDPEN catalyst (after 48 h, 100% conversion, 98% ee (*S*)).

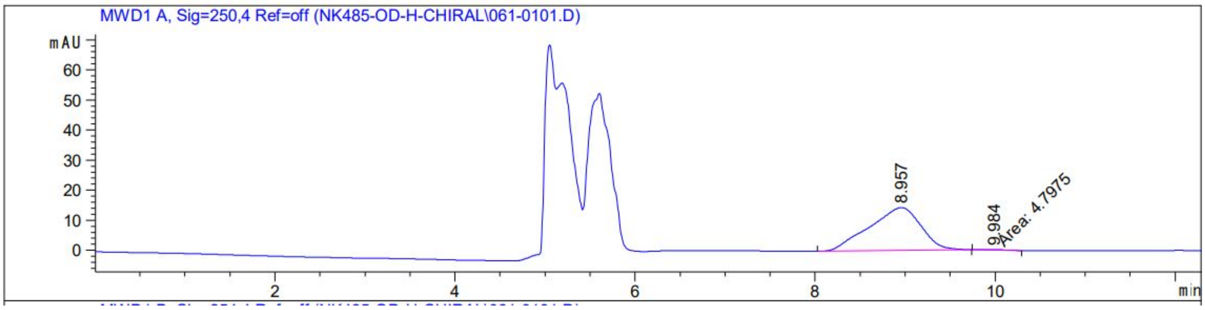

Signal 1: MWD1 A, Sig=250,4 Ref=off

Peak #	RetTime [min]	Type	Width [min]	Area [mAU*s]	Height [mAU]	Area %
1	8.957	BB	0.5655	568.41876	14.24456	99.1631
2	9.984	MM	0.4071	4.79750	1.96393e-1	0.8369

Totals : 573.21627 14.44095

X-ray Crystallography Data for (*R*)-20b – CCDC Deposition Number 2276986.

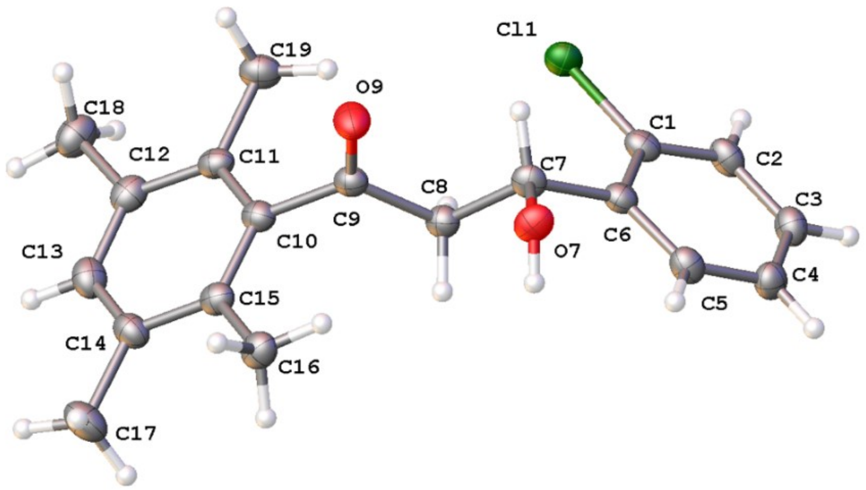

Solid state structure of **20b** with atom labelling and thermal ellipsoids drawn at 50% probability level.

Crystal structure determination of 20b.

The asymmetric unit contains the alcohol. There are two molecules in the unit cell.

The OH was located in a difference map and refined with restraints.

It forms a short contact with the OH of a symmetry related alcohol tabulated below.

Specified hydrogen bonds (with esds except fixed and riding H)

D-H H...A D...A <(DHA)

0.84 2.06 2.8146(19) 150.1 O7-H7...O7_$1

Symmetry operator used to define symmetry related atoms in above contact was.

$1 2-X,-0.5+Y,1-Z

The Flack parameter and the associated Parson's q and Hooft y parameter as a measure of the confidence you can have in the refinement of the handedness of this crystal were.

Flack x: 0.01(2) Shelx2018

Parson's q: 0.005(8) Shelx2018

Hooft y: 0.004(6) Olex2

These are small with a small error so you can have confidence that this is the handedness of this crystal.

Experimental

Single crystals of $C_{19}H_{21}ClO_2$ **20b** were grown from DCM. A suitable crystal was selected and mounted on a Mitegen head with Fomblin oil and placed on a

Rigaku Oxford Diffraction Synergy-S diffractometer with a dual source equipped with a Hybrid pixel array detector. The crystal was kept at 100(2) K during data collection. Using Olex2 [1], the structure was solved with the SHELXT [2] structure solution program using Intrinsic Phasing and refined with the SHELXL [3] refinement package using Least Squares minimisation.

1. Dolomanov, O.V., Bourhis, L.J., Gildea, R.J, Howard, J.A.K. & Puschmann, H. (2009), J. Appl. Cryst. 42, 339-341.
2. Sheldrick, G.M. (2015). Acta Cryst. A71, 3-8.
3. Sheldrick, G.M. (2015). Acta Cryst. C71, 3-8.

Crystal Data for $C_{19}H_{21}ClO_2$ (M =316.81 g/mol): monoclinic, space group P2$_1$ (no. 4), a = 13.4910(3) Å, b = 4.94580(10) Å, c = 13.7194(3) Å, β = 116.013(3)°, V = 822.67(4) Å^3, Z = 2, T = 100(2) K, μ(Cu Kα) = 2.083 mm^{-1}, $Dcalc$ = 1.279 g/cm^3, 21455 reflections measured (7.17° ≤ 2Θ ≤ 159.82°), 3526 unique (R_{int} = 0.0555, R_{sigma} = 0.0314) which were used in all calculations. The final R_1 was 0.0391 (I > 2σ(I)) and wR_2 was 0.1060 (all data).

Table 1 Crystal data and structure refinement for 20b.

Identification code (local)	nk10
Empirical formula	$C_{19}H_{21}ClO_2$
Formula weight	316.81
Temperature/K	100(2)
Crystal system	Monoclinic
Space group	P2$_1$
a/Å	13.4910(3)
b/Å	4.94580(10)
c/Å	13.7194(3)
α/°	90
β/°	116.013(3)
γ/°	90
Volume/Å^3	822.67(4)
Z	2

ρ_{calc}g/cm^3	1.279
μ/mm^{-1}	2.083
F(000)	336.0
Crystal size/mm^3	0.08 × 0.08 × 0.005. colorless block
Radiation	Cu Kα (λ = 1.54184)
2Θ range for data collection/°	7.17 to 159.82
Index ranges	-16 ≤ h ≤ 17, -6 ≤ k ≤ 6, -16 ≤ l ≤ 17
Reflections collected	21455
Independent reflections	3526 [R_{int} = 0.0555, R_{sigma} = 0.0314]
Data/restraints/parameters	3526/1/204
Goodness-of-fit on F^2	1.083
Final R indexes [I>=2σ (I)]	R_1 = 0.0391, wR_2 = 0.1034
Final R indexes [all data]	R_1 = 0.0418, wR_2 = 0.1060
Largest diff. peak/hole / e Å^{-3}	0.36/-0.34
Flack parameter	0.005(8)

1-(4-Bromophenyl)-3-(2,3,5,6-tetramethylphenyl)propane-1,3-dione 21a.

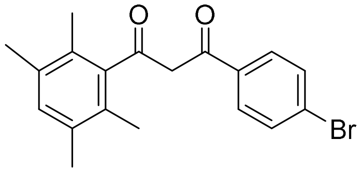

This compound is novel. To a solution of sodium hydride (453 mg, 60% dispersion in mineral oil, 11.4 mmol) in THF (3 mL) at 0 °C was added dropwise a solution of 1-(2,3,5,6-tetramethylphenyl)ethan-1-one **7** (400 mg, 2.3 mmol) in THF (2 mL). The reaction mixture was stirred under a nitrogen atmosphere at 0 °C for 30 min and then stirred under a nitrogen atmosphere at rt for 30 min, after which ethyl 4-bromobenzoate (2.6 g, 11.3 mmol) was added dropwise at 0 °C. The reaction mixture was refluxed at 66 °C and left stirring under the nitrogen atmosphere overnight. The reaction was followed by TLC (4:1 hexane: EtOAc). The mixture was quenched by 2M HCl solution (20 mL). EtOAc (20 mL) was added, and the organic layer was separated. The aqueous layer was extracted with EtOAc (3 × 20 mL), and the combined organic layers were washed with saturated $NaHCO_3$ solution (2 × 20 mL) and brine (20 mL), dried ($MgSO_4$) and filtered. Solvent was removed to give the crude product. The product was isolated via flash chromatography on silica eluted with 1-2% EtOAc in hexane to give 1-(4-bromophenyl)-3-(2,3,5,6-tetramethylphenyl)propane-1,3-dione as a white solid (397 mg, 1.11 mmol, 49%). TLC: Rf ca 0.31 (9:1 hexane: EtOAc), strong UV and $KMnO_4$; Mp: 118.6 °C; HRMS (ESI+) *m/z*: [M+Na]+ Calcd for $C_{19}H_{19}BrNaO_2$ 381.0461; Found 381.0451; 2.6 ppm error; ν_{max} 2963, 2970, 2859, 1715, 1585 cm^{-1}; enol: keto = 100:0; ^1H NMR (500 MHz, $CDCl_3$): δ 7.81 (2H, d, *J* = 8.5, ArH), 7.63 (2H, d, *J* = 8.5, ArH), 7.05 (1H, s, ArH), 6.30 (1H, d, *J* 1.1, CH of enol form), 2.27 (6H, s, CH_3), 2.22 (6H, s, CH_3); $^{13}C\{^1H\}$ NMR (126 MHz, $CDCl_3$): δ 193.0 (C), 183.9 (C), 138.0 (C), 134.3 (C), 134.1 (C), 132.2 (CH), 132.0 (CH), 130.1 (C), 128.8 (CH), 127.5 (C), 99.1 (CH), 19.7 (CH_3), 16.4 (CH_3); *m/z* (ES-API+) 381.0 (M^+ + Na, 100%); Enantiomeric excess and conversion determined by HPLC analysis (Chiralpak OD_H, 30 cm x 6 mm column, hexane:iPrOH 90:10, 1.0 mL/min, T = 25°C) ketone 3.80 min, *R* and *S* isomer 6.96 min and 7.38 min.

^{1}H NMR (500 MHz, $CDCl_3$) of 1-(4-bromophenyl)-3-(2,3,5,6-tetramethylphenyl)propane-1,3-dione **21a**.

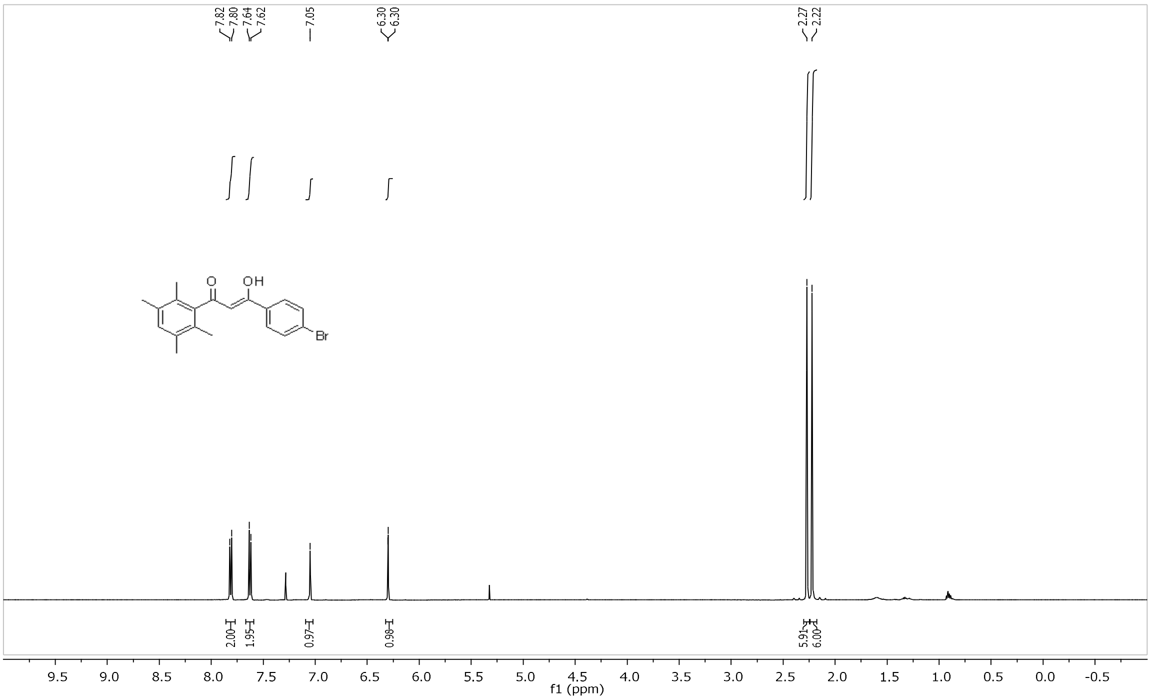

COSY (500 MHz, $CDCl_3$) of 1-(4-bromophenyl)-3-(2,3,5,6-tetramethylphenyl)propane-1,3-dione **21a**.

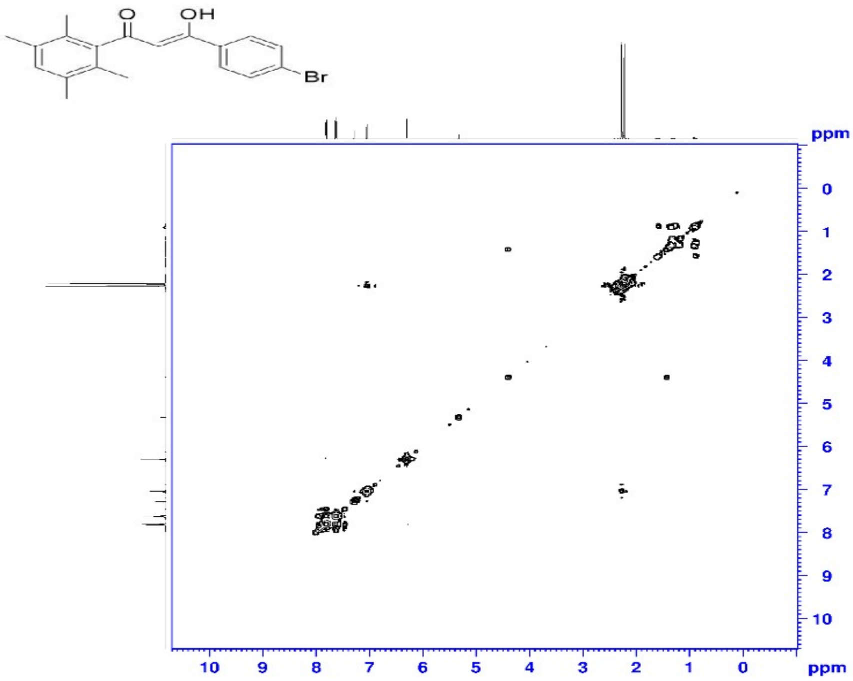

HSQC (126MHz, $CDCl_3$) of 1-(4-bromophenyl)-3-(2,3,5,6-tetramethylphenyl)propane-1,3-dione **21a**.

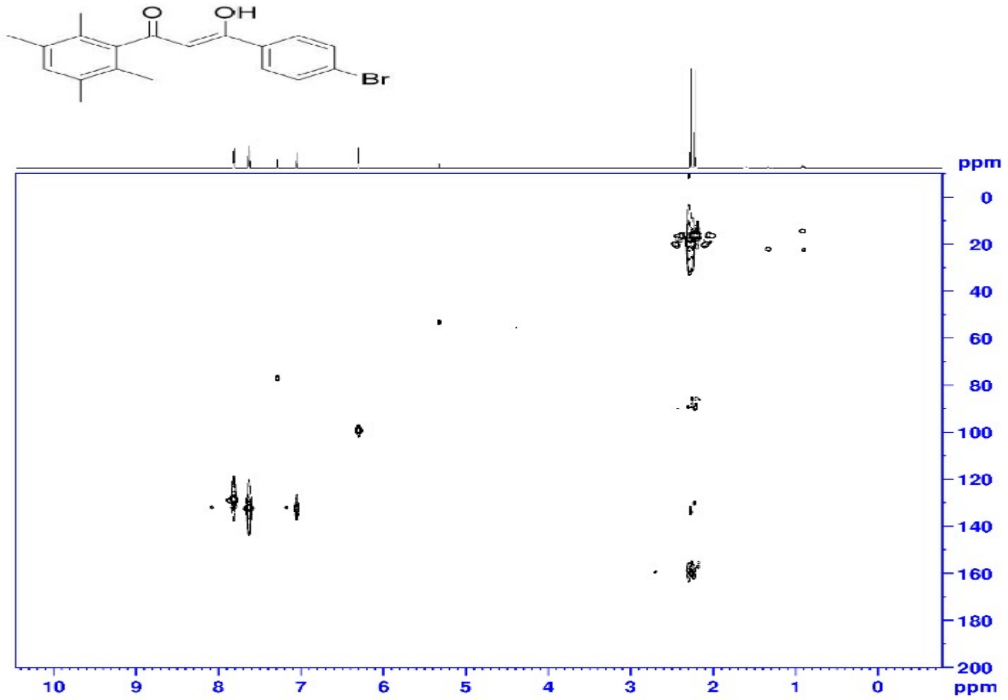

HMBC (126 MHz, $CDCl_3$) of 1-(4-bromophenyl)-3-(2,3,5,6-tetramethylphenyl)propane-1,3-dione **21a**.

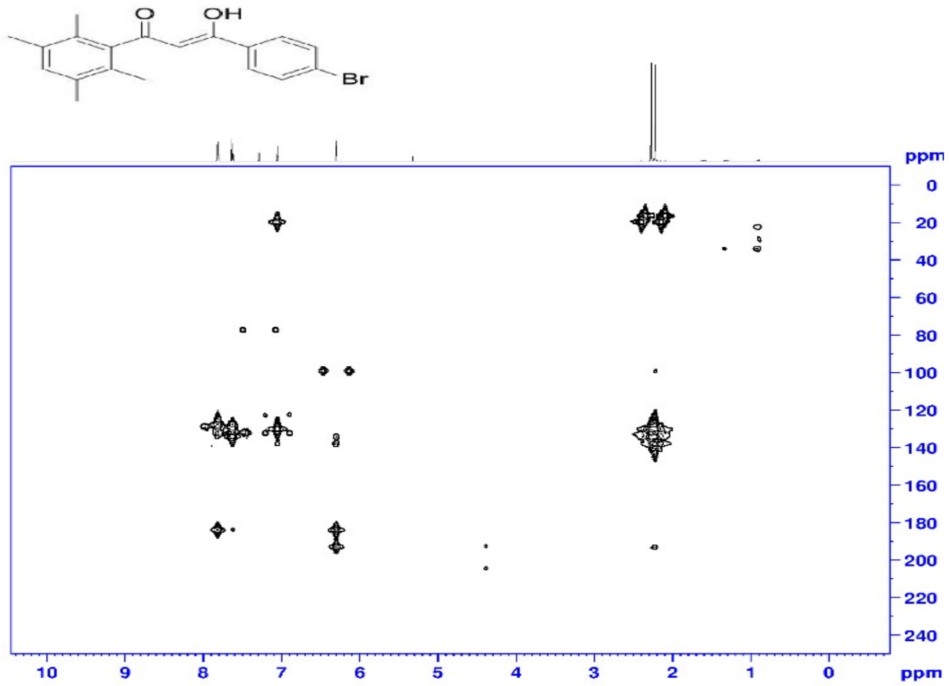

$^{13}C\{^1H\}$ NMR (126 MHz, $CDCl_3$) of 1-(4-bromophenyl)-3-(2,3,5,6-tetramethylphenyl)propane-1,3-dione **21a**.

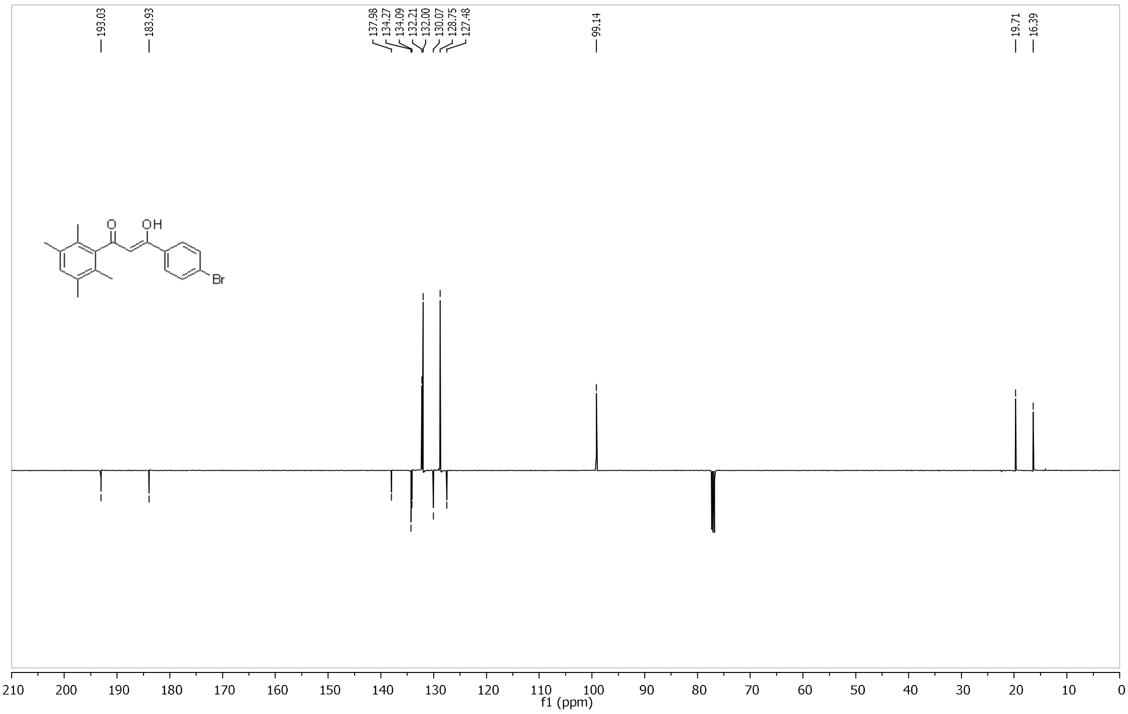

HPLC of racemic 1-(4-bromophenyl)-3-(2,3,5,6-tetramethylphenyl)propane-1,3-dione **21a**.

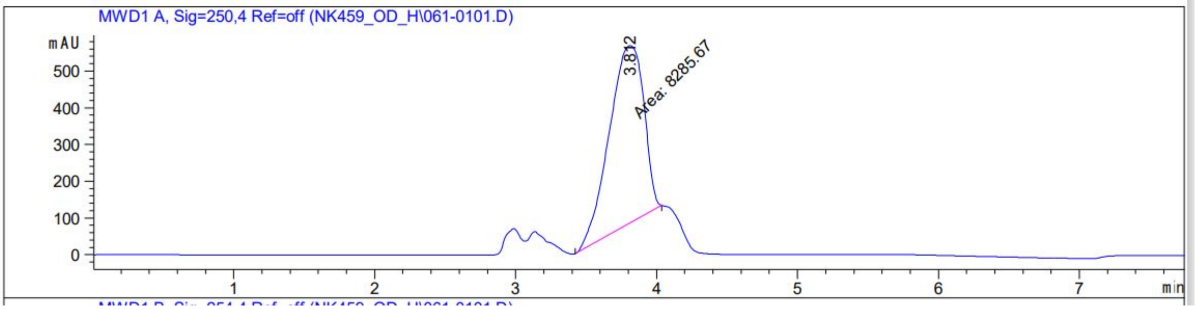

Signal 1: MWD1 A, Sig=250,4 Ref=off

Peak #	RetTime [min]	Type	Width [min]	Area [mAU*s]	Height [mAU]	Area %
1	3.812	MM	0.2858	8285.67480	483.21396	100.0000
Totals :				8285.67480	483.21396	

3-(4-Bromophenyl)-3-hydroxy-1-(2,3,5,6-tetramethylphenyl)propan-1-one 21b.

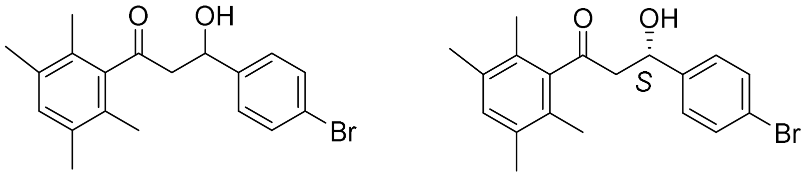

This compound is novel. **Synthesis of a racemic standard**: (*R,R*)-3C-Tethered Ru(II)-TsDPEN catalyst (0.7 mg, 0.0011 mmol, 0.5 mol%) and (*S,S*)-3C-tethered Ru(II)-TsDPEN catalyst (0.7 mg, 0.0011 mmol, 0.5 mol%) were added to FA: TEA (5:2 azeotropic mixture, 0.11 mL) at rt and the mixture was stirred under a nitrogen atmosphere for 15 minutes; after which 1-(4-bomophenyl)-3-(2,3,5,6-tetramethylphenyl)propane-1,3-dione **21a** (80 mg, 0.22 mmol) was added in DCM (0.2 mL). The reaction mixture was stirred under a nitrogen atmosphere and followed by TLC (5:1 hexane: EtOAc). After 48 h, the reaction was quenched using saturated $NaHCO_3$ solution (20 mL). EtOAc (20 mL) was added, and the organic layer was separated. The aqueous layer was extracted with EtOAc (3 x 20 mL) and the combined organic layers were dried ($MgSO_4$) and filtered. The solvent was removed to give the crude product. The product was isolated via flash chromatography on silica eluted with 0-25% EtOAc in petroleum ether to give 3-(4-bromophenyl)-3-hydroxy-1-(2,3,5,6-tetramethylphenyl)propan-1-one **21b** as a yellow solid (63.7 mg, 0.176 mmol, 79%). TLC: Rf ca 0.3 (4:1 hexane: EtOAc), strong UV and PMA; Mp: 108.6 °C; HRMS (ESI+) *m/z*: [M+Na]+ Calcd for $C_{19}H_{21}BrNaO_2$ 383.0617; Found 383.0614; 0.7 ppm error; ν_{max} 3494, 2966, 2921, 2858, 1678 cm^{-1}; ^1H NMR (400 MHz, $CDCl_3$): δ 7.40 (2H, d, *J* = 7.6, ArH), 7.24 – 7.15 (2H, m, ArH), 6.89 (1H, s, ArH), 5.26 (1H, m, ArCH), 3.51 (1H, br. s, OH), 3.00 – 2.95 (2H, m, CH_2), 2.12 (6H, s, CH_3), 2.00 (6H, s, CH_3); $^{13}C\{^1H\}$ NMR (101 MHz, $CDCl_3$): δ 211.9 (C), 141.6 (C), 134.6 (C), 132.0 (CH), 131.6 (CH), 127.9 (C), 127.5 (CH), 121.5 (C), 69.1 (CH), 53.5 (CH_2), 19.4 (CH_3), 15.9 (CH_3); *m/z* (ES-API+) 383.1 (M^+ + Na, 100%); Enantiomeric excess and conversion determined by HPLC analysis (Chiralpak OD_H, 30 cm x 6 mm column, hexane:iPrOH 90:10, 1.0 mL/min, T = 25°C) ketone 3.8 min, *R* and *S* isomer 6.96 min and 7.38 min.

(*S*)-3-(4-Bromophenyl)-3-hydroxy-1-(2,3,5,6-tetramethylphenyl)propan-1-one **21b.**
(*S,S*)-3C-tethered Ru(II)-TsDPEN catalyst (1.7 mg, 0.0028 mmol, 1 mol%) was added to FA: TEA (5:2 azeotropic mixture, 0.14 mL) at rt and the mixture was stirred under

a nitrogen atmosphere for 10-15 minutes; after which 1-(4-bromophenyl)-3-(2,3,5,6-tetramethylphenyl)propane-1,3-dione **21a** (100.0 mg, 0.28 mmol) was added in DCM (0.24 mL). The reaction mixture was stirred under a nitrogen atmosphere for 48 h. The reaction was followed by TLC (5:1 hexane: EtOAc). After 48 h, the reaction was quenched using saturated $NaHCO_3$ solution (20 mL). EtOAc (20 mL) was added, and the organic layer was separated. The aqueous layer was extracted with EtOAc (3 x 20 mL) and the combined organic layers were dried ($MgSO_4$) and filtered. The solvent was removed to give the crude product. The product was isolated via flash chromatography on silica eluted with 0-25% EtOAc in petroleum ether to give (S)-3-(4-bromophenyl)-3-hydroxy-1-(2,3,5,6-tetramethylphenyl)propan-1-one give as a yellow solid (73.7 mg, 0.20 mmol, 73%). The reaction was also followed by HPLC (Chiralpak OD_H, 30 cm x 6 mm column, hexane:iPrOH 90:10, 1.0 mL/min, T = 25°C); $[\alpha]_D^{29}$ – 31.05 (c 0.146 in $CHCl_3$); (after 48 h, 100% conversion, >99% ee (*S*)).

^1H NMR (400 MHz, $CDCl_3$) of 3-(4-bromophenyl)-3-hydroxy-1-(2,3,5,6-tetramethylphenyl)propan-1-one **21b.**

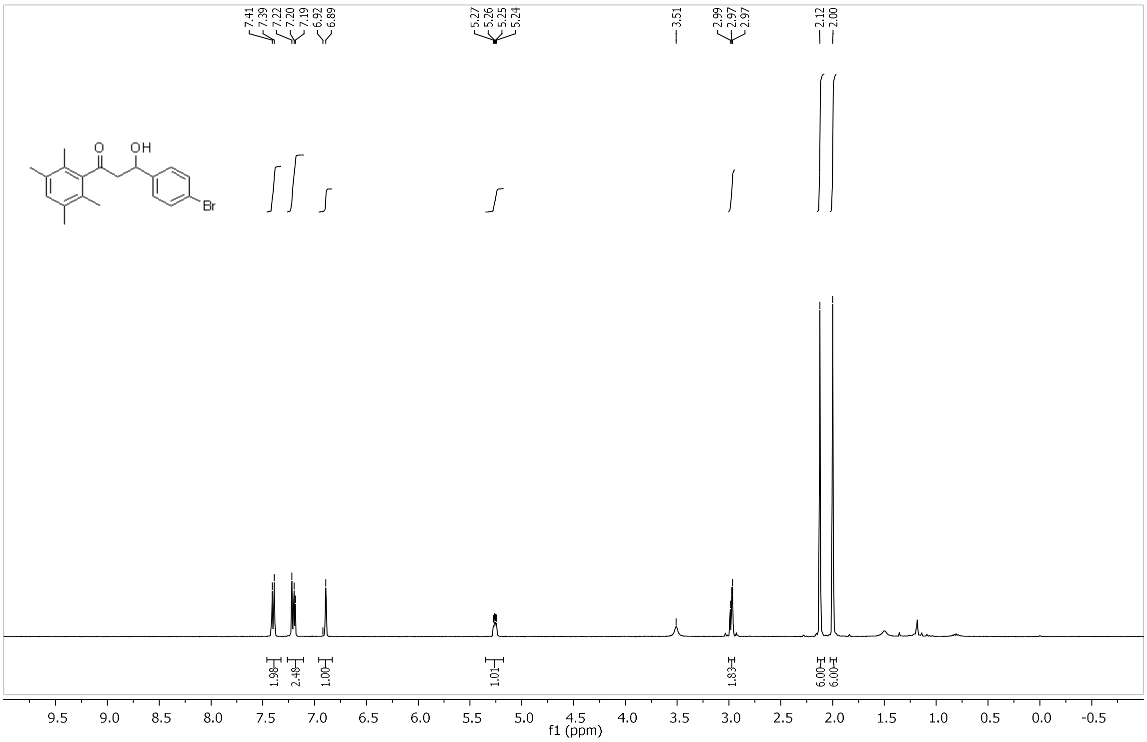

COSY (400 MHz, $CDCl_3$) of 3-(4-bromophenyl)-3-hydroxy-1-(2,3,5,6-tetramethylphenyl)propan-1-one **21b.**

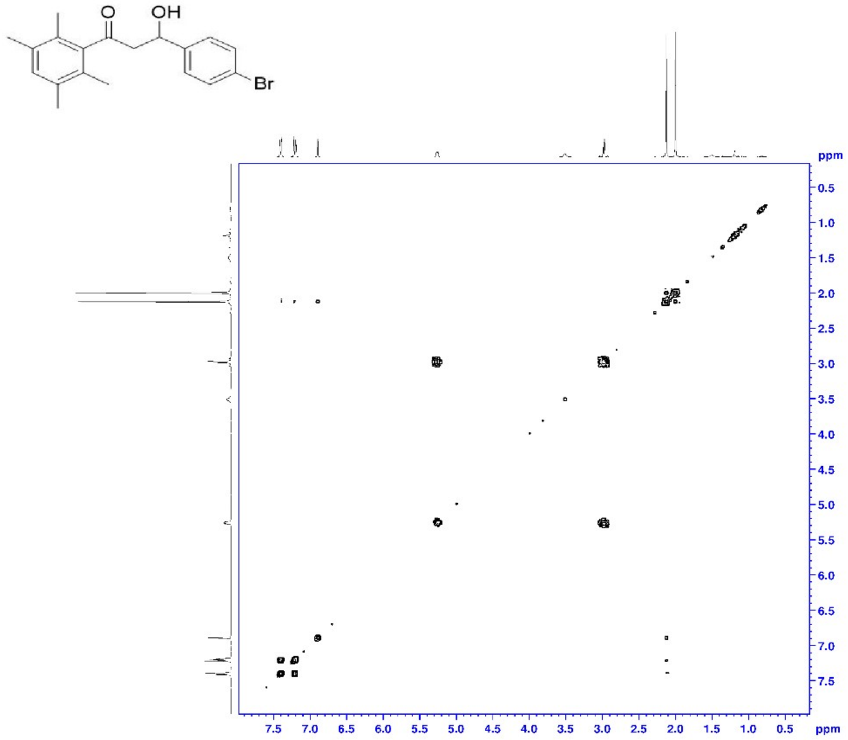

HSQC (101 Hz, $CDCl_3$) of 3-(4-bromophenyl)-3-hydroxy-1-(2,3,5,6-tetramethylphenyl)propan-1-one **21b.**

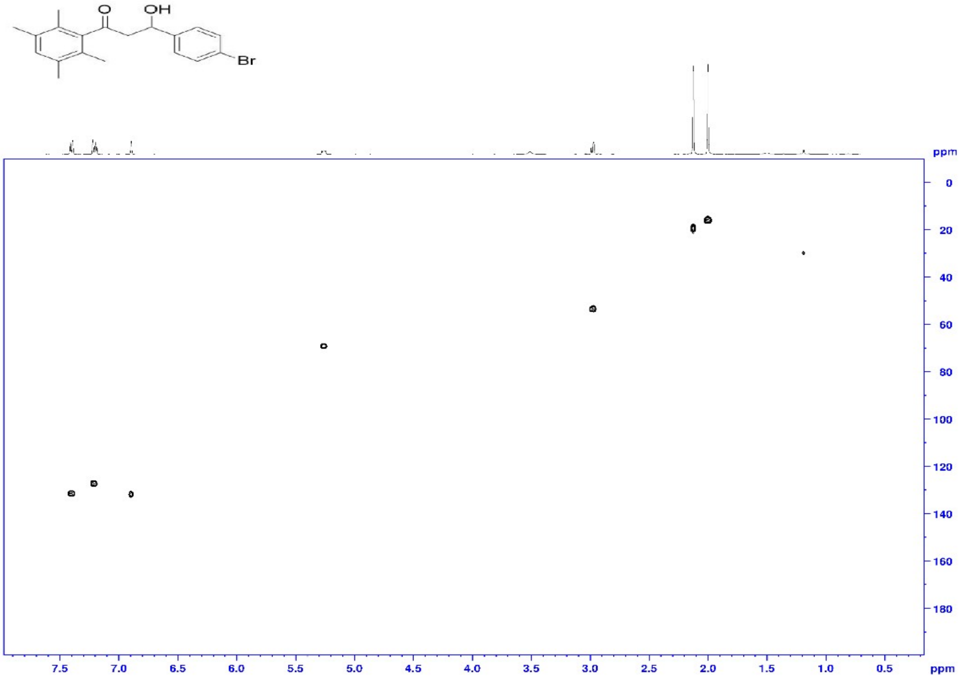

HMBC (101 Hz, $CDCl_3$) of 3-(4-bromophenyl)-3-hydroxy-1-(2,3,5,6-tetramethylphenyl)propan-1-one **21b.**

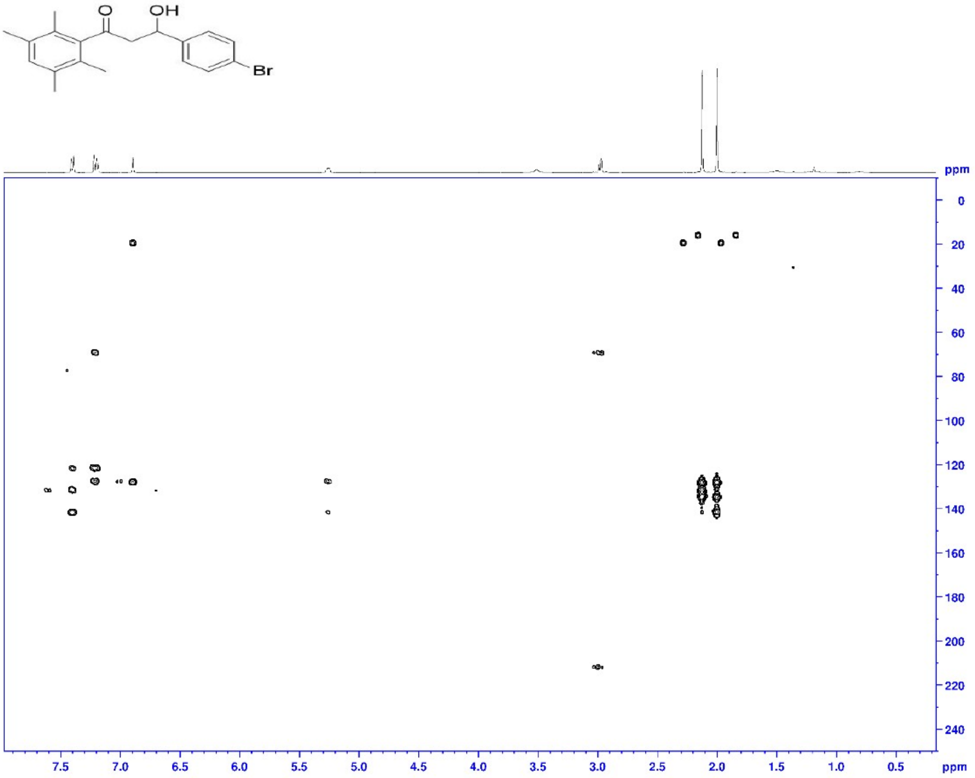

$^{13}C\{^1H\}$ NMR (101 Hz, $CDCl_3$) of 3-(4-bromophenyl)-3-hydroxy-1-(2,3,5,6-tetramethylphenyl)propan-1-one **21b.**

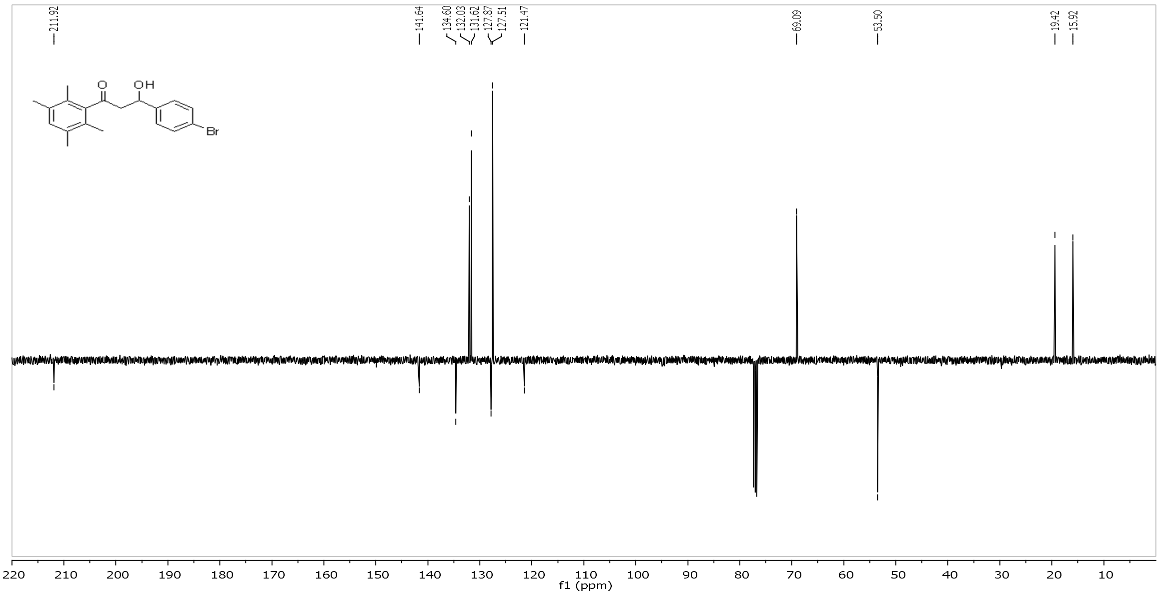

HPLC of racemic 3-(4-bromophenyl)-3-hydroxy-1-(2,3,5,6-tetramethylphenyl)propan-1-one **21b.**

:

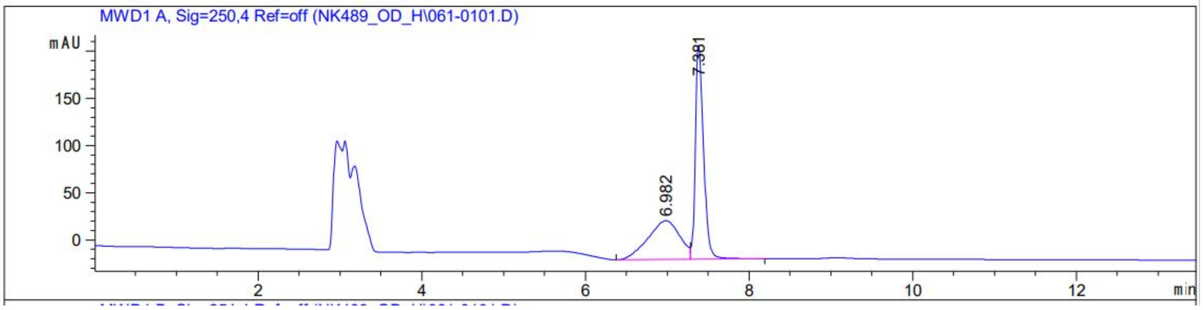

Signal 1: MWD1 A, Sig=250,4 Ref=off

Peak #	RetTime [min]	Type	Width [min]	Area [mAU*s]	Height [mAU]	Area %
1	6.982	BV	0.4100	1115.67688	41.18533	41.3496
2	7.381	VB	0.1093	1582.47693	227.02834	58.6504

Totals : 2698.15381 268.21367

HPLC of (*S*)-3-(4-bromophenyl)-3-hydroxy-1-(2,3,5,6-tetramethylphenyl)propan-1-one **21b.**

(*S,S*)-3C-tethered Ru(II)-TsDPEN catalyst (after 48 h, 100% conversion, >99% ee (*S*)).

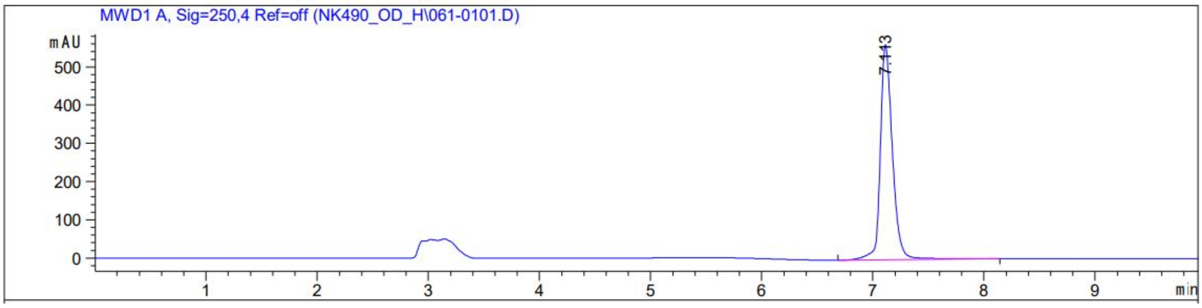

Signal 1: MWD1 A, Sig=250,4 Ref=off

Peak #	RetTime [min]	Type	Width [min]	Area [mAU*s]	Height [mAU]	Area %
1	7.113	BB	0.1183	4262.38818	562.97461	100.0000

Totals : 4262.38818 562.97461

1-(2-Methoxyphenyl)-3-(2,3,5,6-tetramethylphenyl)propane-1,3-dione 22a.

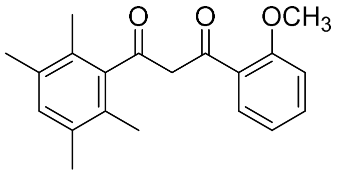

This compound is novel. To a solution of sodium hydride (288 mg, 60% dispersion in mineral oil, 7.2 mmol) in THF (3 mL) at 0 °C was added dropwise a solution of 1-(2,3,5,6-tetramethylphenyl)ethan-1-one **7** (255 mg, 1.40 mmol) in THF (2 mL). The reaction mixture was stirred under a nitrogen atmosphere at 0 °C for 30 min and then stirred under a nitrogen atmosphere at rt for 30 min, after which ethyl 2-methoxybenzoate (1.3 g, 7.2 mmol) was added dropwise at 0 °C. The reaction mixture was refluxed at 66 °C and left stirring under the nitrogen atmosphere overnight. The reaction was followed by TLC (4:1 hexane: EtOAc). The mixture was quenched by 2M HCl solution (20 mL). EtOAc (20 mL) was added, and the organic layer was separated. The aqueous layer was extracted with EtOAc (3 × 20 mL), and the combined organic layers were washed with saturated $NaHCO_3$ solution (2 × 20 mL) and brine (20 mL), dried ($MgSO_4$) and filtered. Solvent was removed to give the crude product. The product was isolated via flash chromatography on silica eluted with 0-5% EtOAc in hexane to give 1-(2-methoxyphenyl)-3-(2,3,5,6-tetramethylphenyl)propane-1,3-dione **22a** as a yellow solid (364 mg, 1.17 mmol, 84%). TLC: Rf ca 0.19 (9:1 hexane: EtOAc), strong UV and $KMnO_4$; Mp: 146 °C; HRMS (ESI+) *m/z*: [M+Na]+ Calcd for $C_{20}H_{22}NaO_3$ 333.1461; Found 333.1452; 2.8 ppm error; ν_{max} 2943, 2921, 2856, 1599, 1577 cm^{-1}; enol: keto = 100:0; ^1H NMR (500 MHz, $CDCl_3$): δ 8.00 (1H, d, *J* = 7.8, ArH), 7.49 (1H, t, *J* = 7.8, ArH), 7.10 (1H, t, *J* = 7.6, ArH), 7.04 (1H, s, ArH), 6.99 (1H, d, *J* 8.3, ArH), 6.61 (1H, s, ArCH), 3.88 (3H, s, CH_3), 2.28 (6H, s, CH_3), 2.25 (6H, s, CH_3); $^{13}C\{^1H\}$ NMR (126 MHz, $CDCl_3$): δ 192.7 (C), 183.7 (C), 158.7 (C), 138.7 (C), 134.1 (C), 1333 (CH), 131.9 (CH), 130.5 (CH), 130.3 (C), 124.6 (C), 120.8 (CH), 111.6 (CH), 104.6 (CH), 55.7 (CH_3), 19.8 (CH_3), 16.4 (CH_3); *m/z* (ES-API+) 333.1 (M^+ + Na, 100%); Enantiomeric excess and conversion determined by HPLC analysis (Chiralpak IC, 30 cm x 6 mm column, hexane:iPrOH 90:10, 1.0 mL/min, T = 25°C) ketone 8.3 min, *R* and *S* isomer 12.7 min and 13.3 min.

^{1}H NMR (500 MHz, $CDCl_3$) of 1-(2-methoxyphenyl)-3-(2,3,5,6-tetramethylphenyl)propane-1,3-dione **22a**.

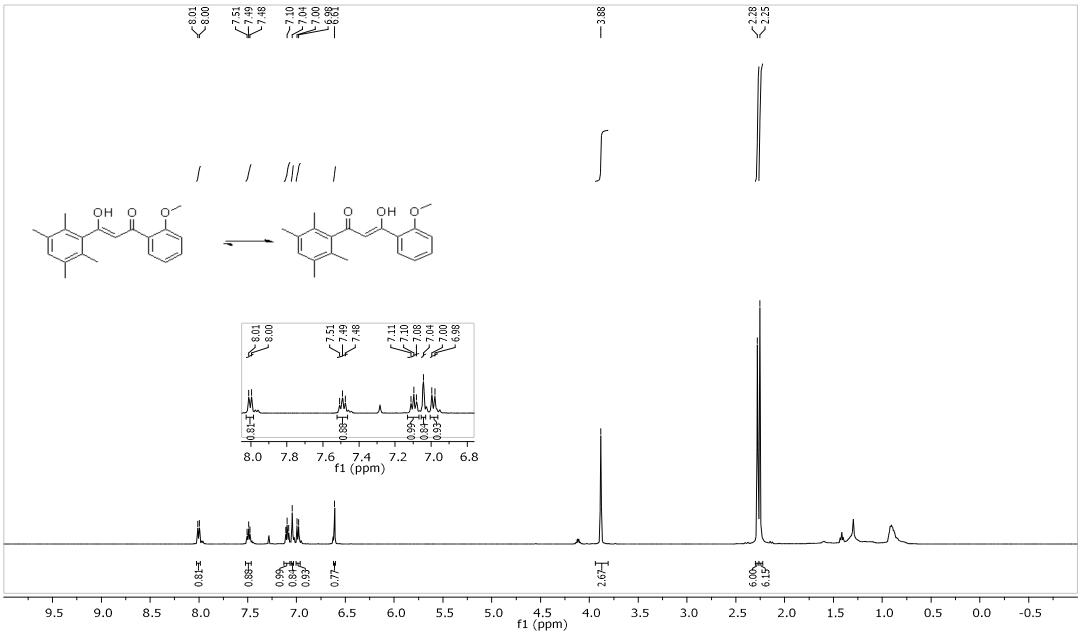

COSY (500 MHz, $CDCl_3$) of 1-(2-methoxyphenyl)-3-(2,3,5,6-tetramethylphenyl)propane-1,3-dione **22a**.

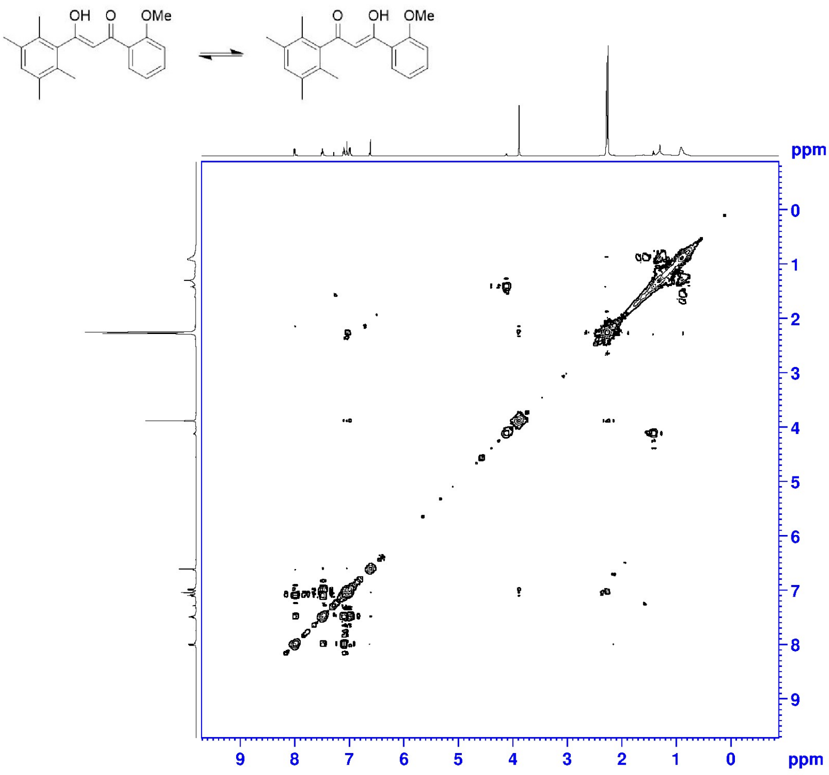

HSQC (126 Hz, $CDCl_3$) of 1-(2-methoxyphenyl)-3-(2,3,5,6-tetramethylphenyl)propane-1,3-dione **22a**.

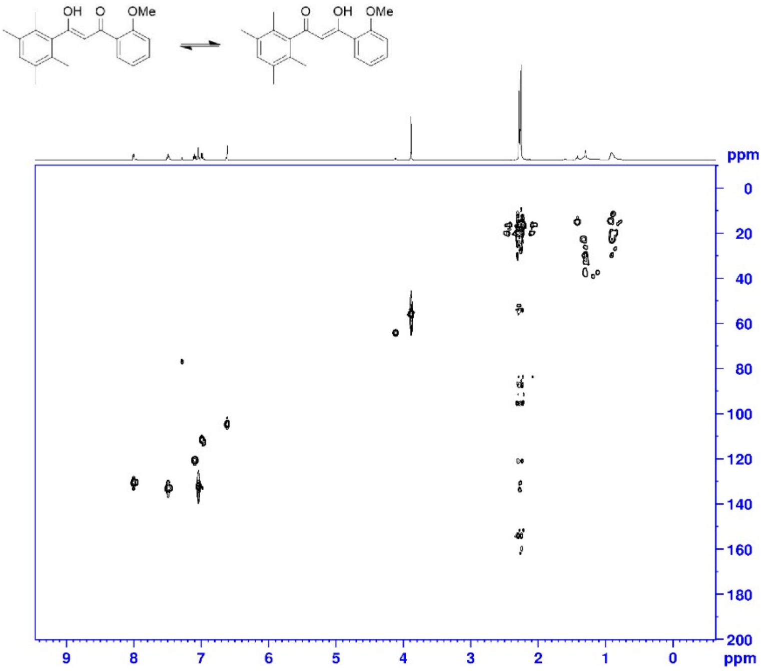

HMBC (126 Hz, $CDCl_3$) of 1-(2-methoxyphenyl)-3-(2,3,5,6-tetramethylphenyl)propane-1,3-dione **22a**.

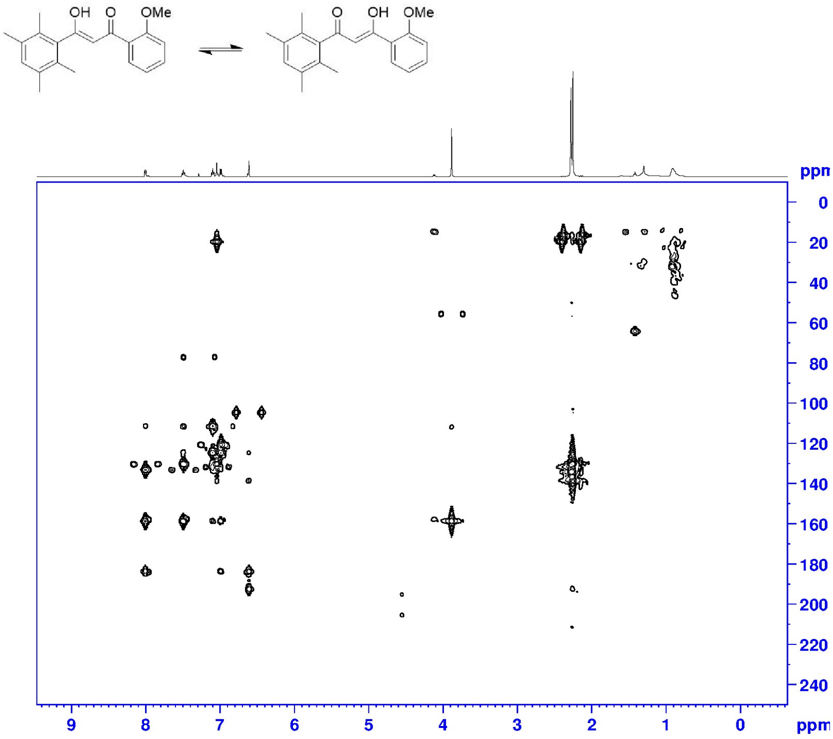

^{13}C (126 Hz, $CDCl_3$) of 1-(2-methoxyphenyl)-3-(2,3,5,6-tetramethylphenyl)propane-1,3-dione **22a**.

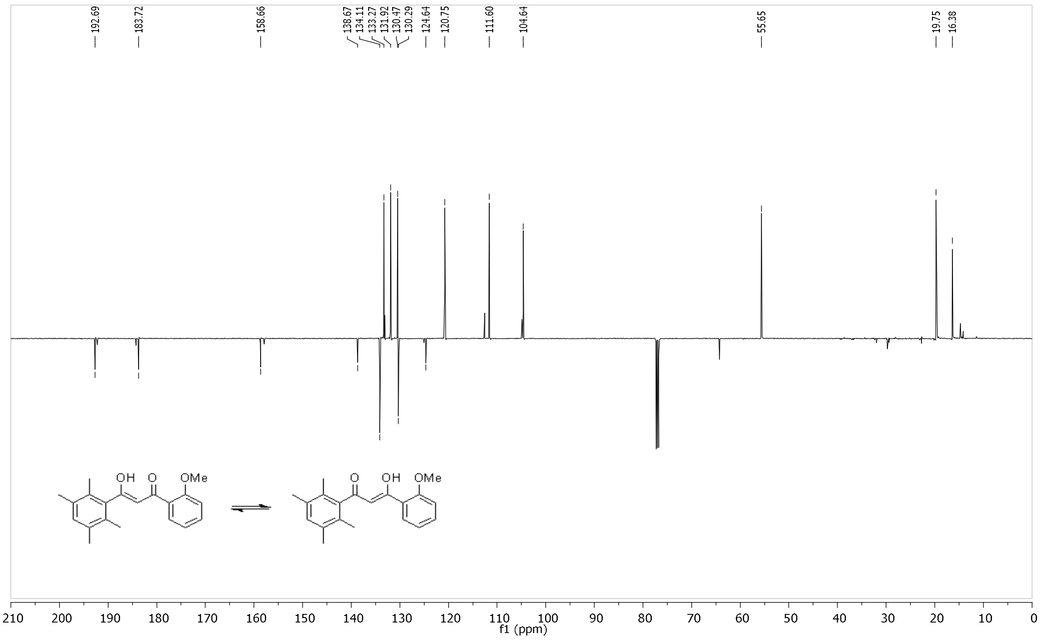

HPLC of 1-(2-methoxyphenyl)-3-(2,3,5,6-tetramethylphenyl)propane-1,3-dione **22a**.

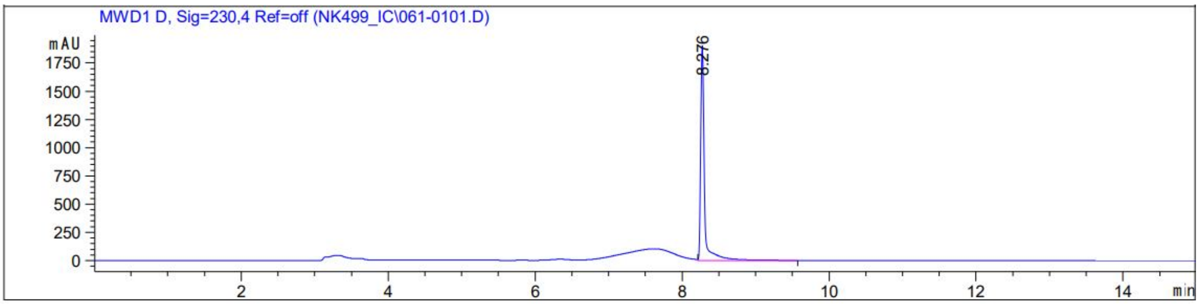

Signal 4: MWD1 D, Sig=230,4 Ref=off

Peak #	RetTime [min]	Type	Width [min]	Area [mAU*s]	Height [mAU]	Area %
1	8.276	VB	0.0530	6705.94336	1906.51001	100.0000
Totals :				6705.94336	1906.51001	

3-Hydroxy-3-(2-methoxyphenyl)-1-(2,3,5,6-tetramethylphenyl)propan-1-one 22b.

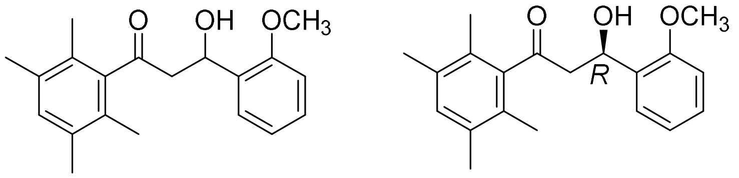

This compound is novel. **Synthesis of a racemic standard**: (*R,R*)-3C-Tethered Ru(II)-TsDPEN catalyst (0.4 mg, 0.0006 mmol, 0.5 mol%) and (*S,S*)-3C-tethered Ru(II)-TsDPEN catalyst (0.4 mg, 0.0006 mmol, 0.5 mol%) were added to FA: TEA (5:2 azeotropic mixture, 0.1 mL) at rt and the mixture was stirred under a nitrogen atmosphere for 15 minutes; after which 1-(2-methoxyphenyl)-3-(2,3,5,6-tetramethylphenyl)propane-1,3-dione **7** (40 mg, 0.13 mmol) was added in DCM (0.2 mL). The reaction mixture was stirred under a nitrogen atmosphere and followed by TLC (5:1 hexane: EtOAc). After 48 h, the reaction was quenched using saturated $NaHCO_3$ solution (20 mL). EtOAc (20 mL) was added, and the organic layer was separated. The aqueous layer was extracted with EtOAc (3 x 20 mL), and the combined organic layers were dried ($MgSO_4$) and filtered. The solvent was removed to give the crude product. The product was isolated via flash chromatography on silica eluted with 0-20% EtOAc in petroleum ether to give 3-hydroxy-3-(2-methoxyphenyl)-1-(2,3,5,6-tetramethylphenyl)propan-1-one **22b** as a white solid (11.6 mg, 0.04 mmol, 29%). TLC: Rf ca 0.1 (4:1 hexane: EtOAc), strong UV and PMA; Mp: 101.9 °C; HRMS (ESI+) *m/z*: [M+Na]+ Calcd for $C_{20}H_{24}NaO_3$ 335.1618; Found 335.1607; 3.3 ppm error; ν_{max} 3562, 3007, 2858, 1701 cm^{-1}; ^1H NMR (500 MHz, $CDCl_3$): δ 7.56 (1H, d, J = 7.5, ArH), 7.27 (1H, m, ArH), 7.02 (1H, t, J = 7.5, ArH), 6.99 (1H, s, ArH), 6.88 (1H, d, J = 8.2, ArH), 5.70 – 5.63 (1H, m, ArCH), 3.86 (3H, s, CH_3), 3.73 (1H, br.s , OH), 3.34 – 3.24 (1H, m, CH_2), 3.01 (1H, m, CH_2), 2.23 (6H, s, CH_3), 2.12 (6H, s, CH_3); $^{13}C\{^1H\}$ NMR (126 MHz, $CDCl_3$): δ 212.8 (C), 155.7 (C), 142.1 (C), 134.4 (C), 131.8 (CH), 130.9 (C), 128.3 (CH), 128.0 (C), 126.4 (CH), 120.9 (CH), 110.1 (CH), 65.3 (CH), 55.2 (CH_3), 52.1 (CH_2), 19.4 (CH_3), 15.8 (CH_3); *m/z* (ES-API+) 335.2 (M^+ + Na, 100%); Enantiomeric excess and conversion determined by HPLC analysis (Chiralpak IC, 30 cm x 6 mm column, hexane:iPrOH 90:10, 1.0 mL/min, T = 25°C) ketone 8.3 min, *R* and *S* isomer 12.7 min and 13.3 min.

(*R*)-3-Hydroxy-3-(2-methoxyphenyl)-1-(2,3,5,6-tetramethylphenyl)propan-1-one **22b..**

(*R,R*)-3C-tethered Ru(II)-TsDPEN catalyst (0.37 mg, 0.0006 mmol, 1 mol%) was added to FA: TEA (5:2 azeotropic mixture, 0.04 mL) at rt and the mixture was stirred under a nitrogen atmosphere for 10-15 minutes; after which 1-(2-methoxyphenyl)-3-(2,3,5,6-tetramethylphenyl)propane-1,3-dione **22a** (20.0 mg, 0.064 mmol) was added in DCM (0.65 mL). The reaction mixture was stirred under a nitrogen atmosphere for 70 h. The reaction was followed by TLC (5:1 hexane: EtOAc). After 70 h, the reaction was quenched using saturated $NaHCO_3$ solution (20 mL). EtOAc (20 mL) was added, and the organic layer was separated. The aqueous layer was extracted with EtOAc (3 x 20 mL) and the combined organic layers were dried ($MgSO_4$) and filtered. The solvent was removed to give the crude product. The product was isolated via flash chromatography on silica eluted with 0-20% EtOAc in petroleum ether to give (*R*)-3-hydroxy-3-(2-methoxyphenyl)-1-(2,3,5,6-tetramethylphenyl)propan-1-one **22b** as a white solid (14.5 mg, 0.046 mmol, 73%).). The reaction was also followed by HPLC (Chiralpak IC, 30 cm x 6 mm column, hexane:iPrOH 90:10, 1.0 mL/min, T = 25°C); $[\alpha]_D^{29}$ + 17.8 (c 0.234 in $CHCl_3$); (after 48 h, 100% conversion, 94% ee (*R*)).

^1H NMR (500 MHz, $CDCl_3$) of 3-hydroxy-3-(2-methoxyphenyl)-1-(2,3,5,6-tetramethylphenyl)propan-1-one **22b.**

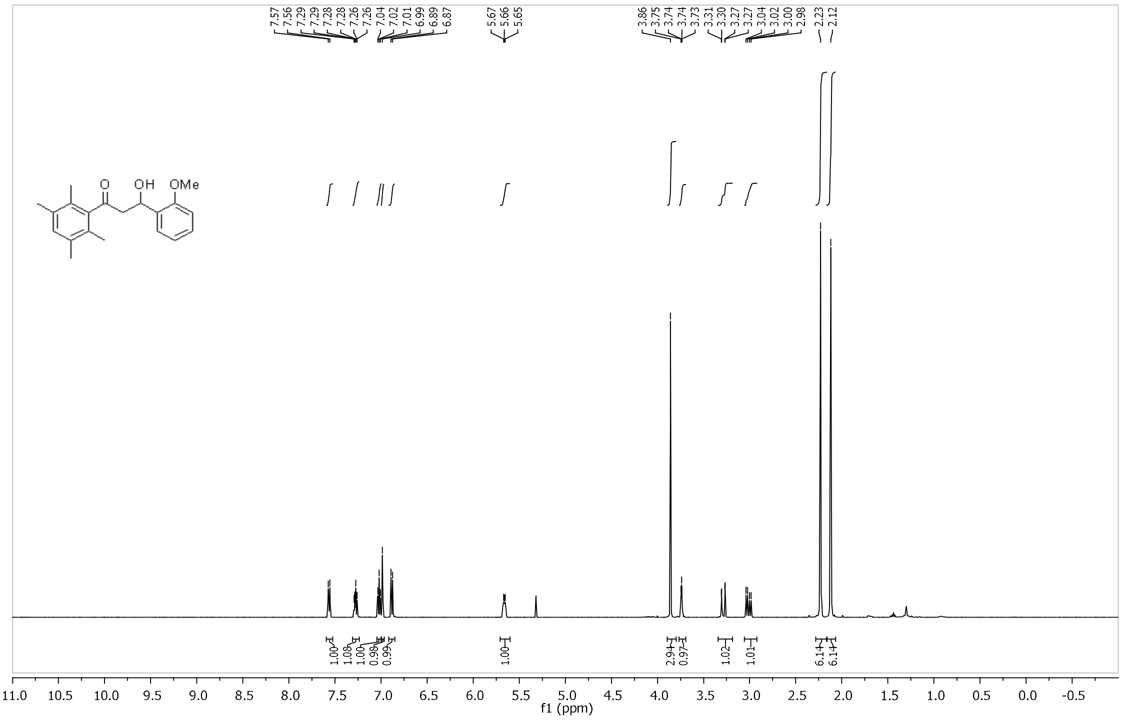

COSY (500 MHz, $CDCl_3$) of 3-hydroxy-3-(2-methoxyphenyl)-1-(2,3,5,6-tetramethylphenyl)propan-1-one **22b.**

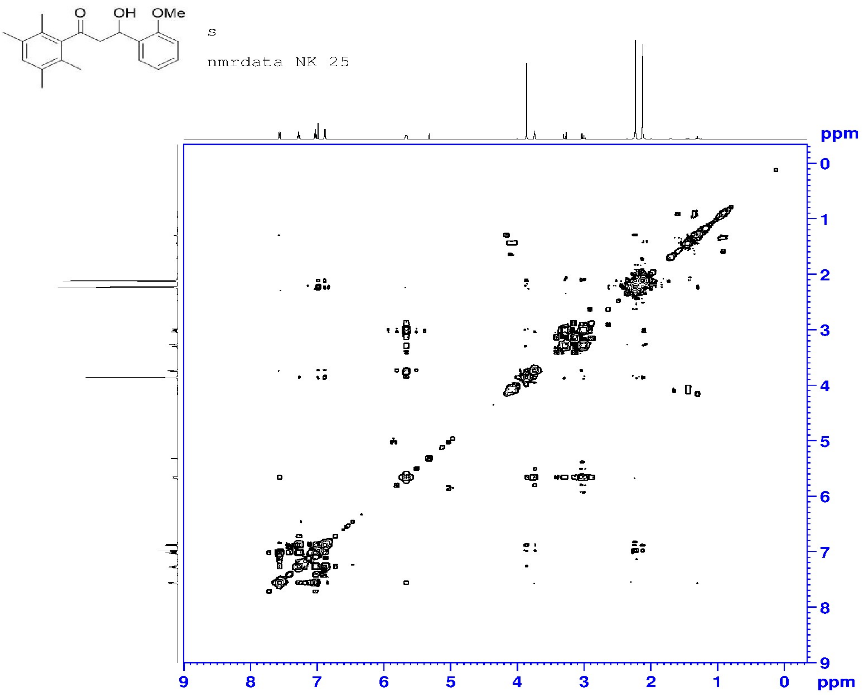

HSQC (126 MHz, $CDCl_3$) of 3-hydroxy-3-(2-methoxyphenyl)-1-(2,3,5,6-tetramethylphenyl)propan-1-one **22b.**

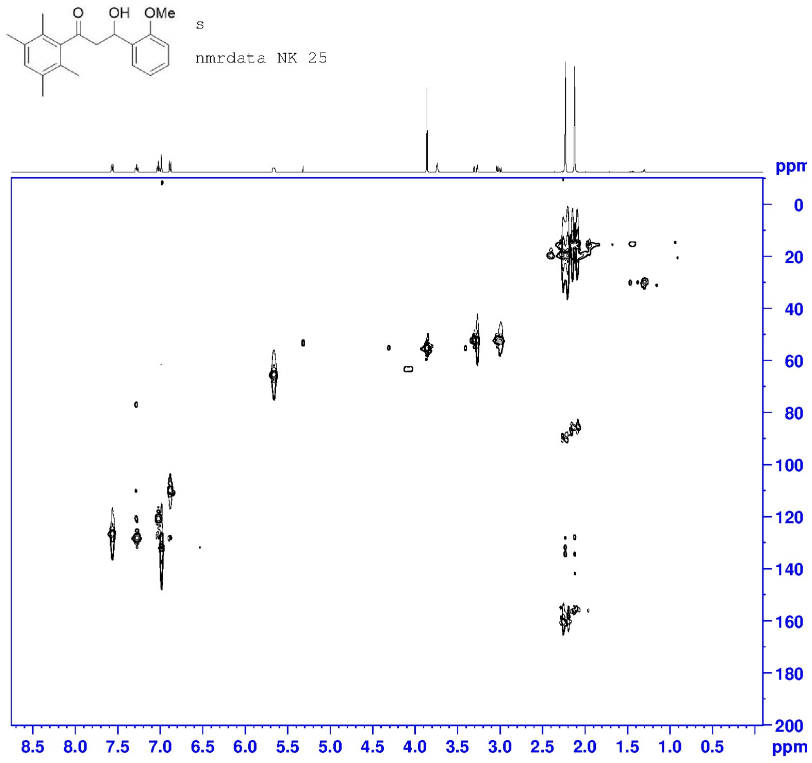

HMBC (126 MHz, $CDCl_3$) of 3-hydroxy-3-(2-methoxyphenyl)-1-(2,3,5,6-tetramethylphenyl)propan-1-one **22b.**

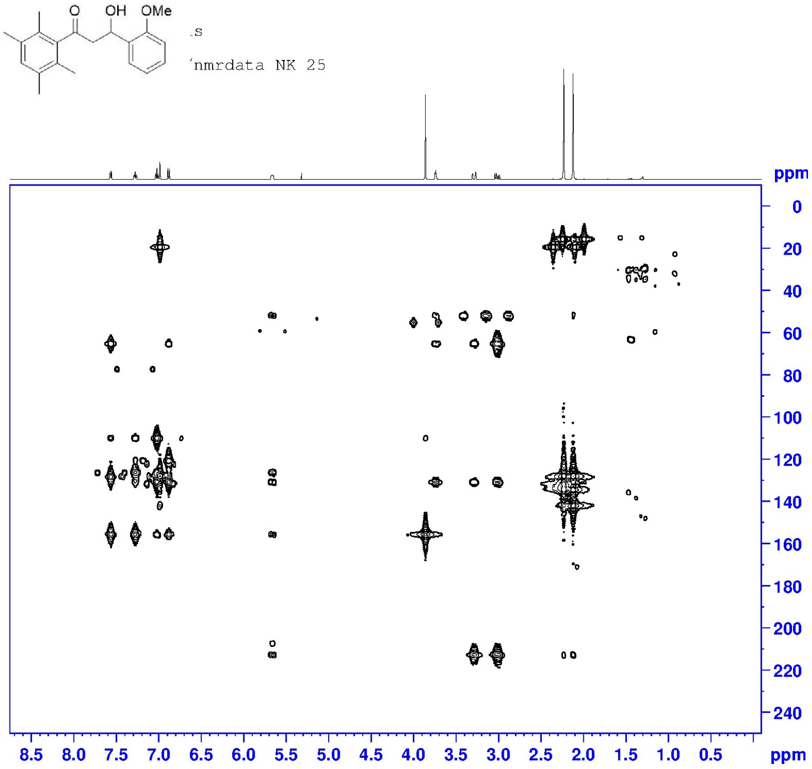

$^{13}C\{^1H\}$ NMR (126 Hz, $CDCl_3$) of 3-hydroxy-3-(2-methoxyphenyl)-1-(2,3,5,6-tetramethylphenyl)propan-1-one **22b.**

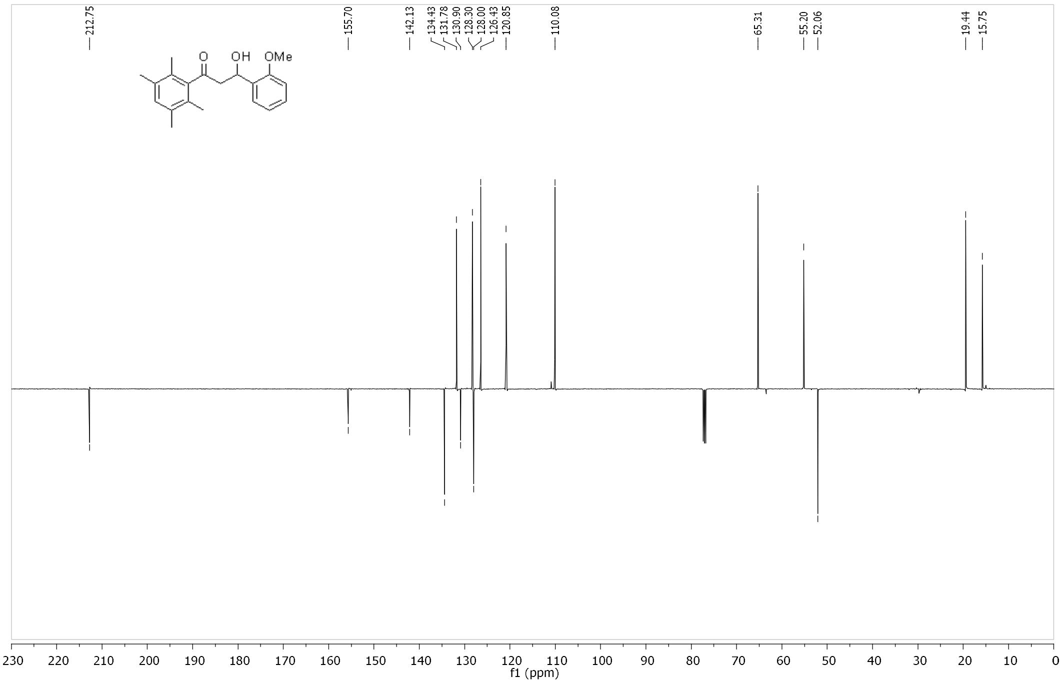

HPLC of racemic of 3-hydroxy-3-(2-methoxyphenyl)-1-(2,3,5,6-tetramethylphenyl)propan-1-one **22b.**

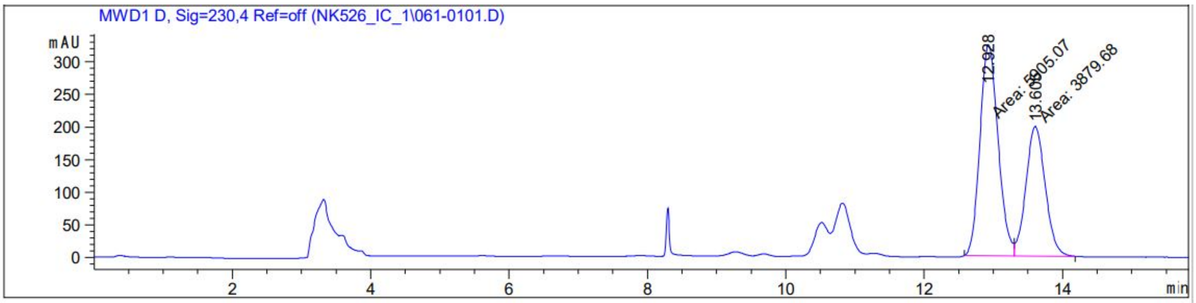

Signal 4: MWD1 D, Sig=230,4 Ref=off

Peak #	RetTime [min]	Type	Width [min]	Area [mAU*s]	Height [mAU]	Area %
1	12.928	MF	0.3036	5905.06836	324.14697	60.3497
2	13.605	FM	0.3246	3879.68433	199.17630	39.6503
Totals :				9784.75269	523.32327	

HPLC of (*R*)-3-hydroxy-3-(2-methoxyphenyl)-1-(2,3,5,6-tetramethylphenyl)propan-1-one **22b.**

(*R,R*)-3C-tethered Ru(II)-TsDPEN catalyst (after 48 h, 100% conversion, 94% ee (*R*)).

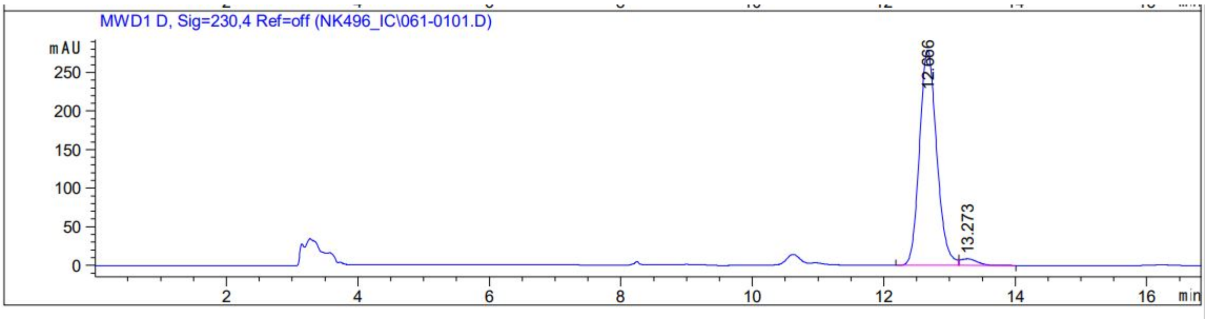

Signal 4: MWD1 D, Sig=230,4 Ref=off

Peak #	RetTime [min]	Type	Width [min]	Area [mAU*s]	Height [mAU]	Area %
1	12.666	BV	0.2818	5063.86035	278.92484	97.0985
2	13.273	VB	0.2697	151.31770	8.49978	2.9015
Totals :				5215.17805	287.42461	

1-(2,3,5,6-Tetramethylphenyl)-3-(thiophen-2-yl)propane-1,3-dione 23a.

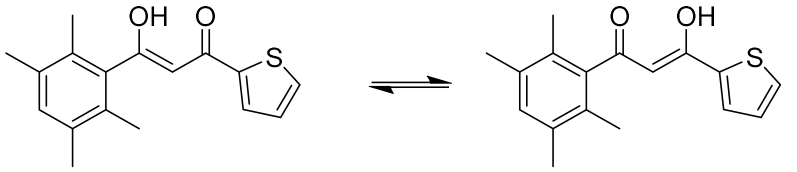

This compound is novel. To a solution of sodium hydride (454 mg, 60% dispersion in mineral oil, 11.3 mmol) in THF (3 mL) at 0 °C was added dropwise a solution of 1-(2,3,5,6-tetramethylphenyl)ethan-1-one **7** (400 mg, 2.3 mmol) in THF (2 mL). The reaction mixture was stirred under a nitrogen atmosphere at 0 °C for 30 min and then stirred under a nitrogen atmosphere at rt for 30 min, after which ethyl thiophene-2-carboxylate (1.8 g, 11.3 mmol) was added dropwise at 0 °C. The reaction mixture was refluxed at 66 °C and left stirring under the nitrogen atmosphere overnight. The reaction was followed by TLC (4:1 hexane: EtOAc). The mixture was quenched by 2M HCl solution (20 mL). EtOAc (20 mL) was added, and the organic layer was separated. The aqueous layer was extracted with EtOAc (3 × 20 mL), and the combined organic layers were washed with saturated $NaHCO_3$ solution (2 × 20 mL) and brine (20 mL), dried ($MgSO_4$) and filtered. Solvent was removed to give the crude product. The product was isolated via flash chromatography on silica eluted with 0-1.5% EtOAc in hexane to give 1-(2,3,5,6-tetramethylphenyl)-3-(thiophen-2-yl)propane-1,3-dione **23a** as a yellow solid (311 mg, 1.10 mmol, 48%). TLC: Rf ca 0.26 (9:1 hexane: EtOAc), strong UV and $KMnO_4$; Mp: 132.2 °C; HRMS (ESI+) *m/z*: [M+Na]+ Calcd for $C_{17}H_{18}NaO_2S$ 309.0920; Found 309.0916; 1.2 ppm error; ν_{max} 3106, 2935, 2918, 2859, 1596 cm^{-1}; enol: keto = 100:0; ^1H NMR (500 MHz, $CDCl_3$): δ 15.83 (1H, s, OH), 7.72 (1H, dd, *J* = 3.8, 0.9, ArH), 7.66 (1H, dd, *J* = 4.9, 0.9,ArH), 7.17 (1H, m, ArH), 7.05 (1H, s, ArH), 6.17 (1H, s, CH), 2.27 (6H, s, CH_3), 2.24 (6H, s, CH_3); $^{13}C\{^1H\}$ NMR (126 MHz, $CDCl_3$): δ 186.0 (C), 183.0 (C), 142.3 (C), 136.6 (C), 134.2 (C), 133.0 (CH), 132.3 (C), 130.7 (CH), 128.4 (CH), 99.3 (CH), 19.8 (CH_3), 16.4 (CH_3); *m/z* (ES-API+) 309.1 (M^+ + Na, 100%); Enantiomeric excess and conversion determined by HPLC analysis (Chiralpak IC, 30 cm x 6 mm column, hexane:iPrOH 90:10, 1.0 mL/min, T = 25°C) ketone 7.4 min, *R* and *S* isomer 11.2 min and 12.7 min.

^{1}H NMR (500 MHz, $CDCl_3$) of 1-(2,3,5,6-tetramethylphenyl)-3-(thiophen-2-yl)propane-1,3-dione **23a**.

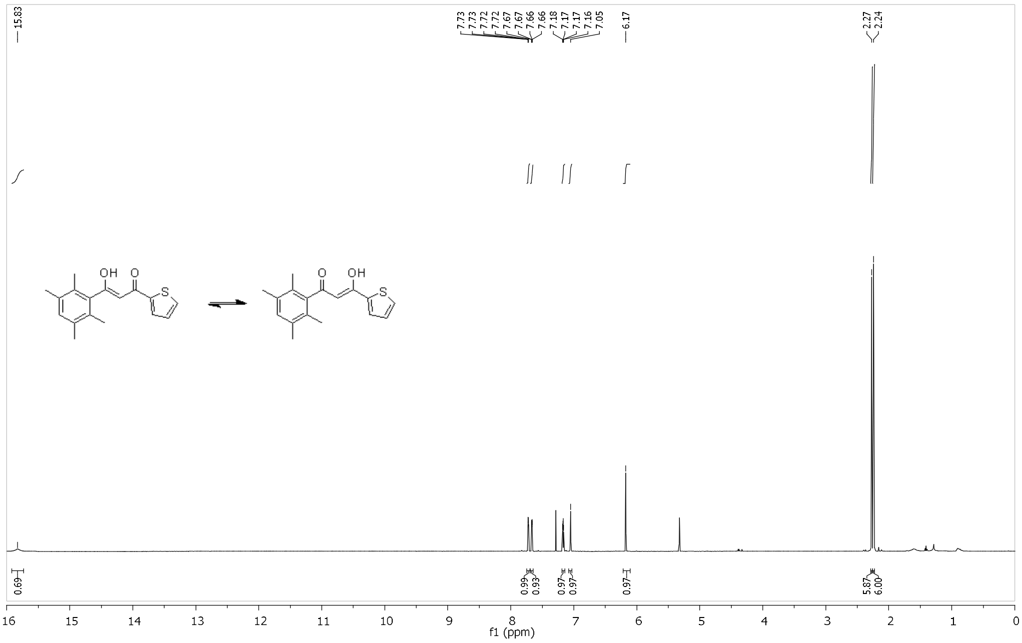

COSY (500 MHz, $CDCl_3$) of 1-(2,3,5,6-tetramethylphenyl)-3-(thiophen-2-yl)propane-1,3-dione **23a**.

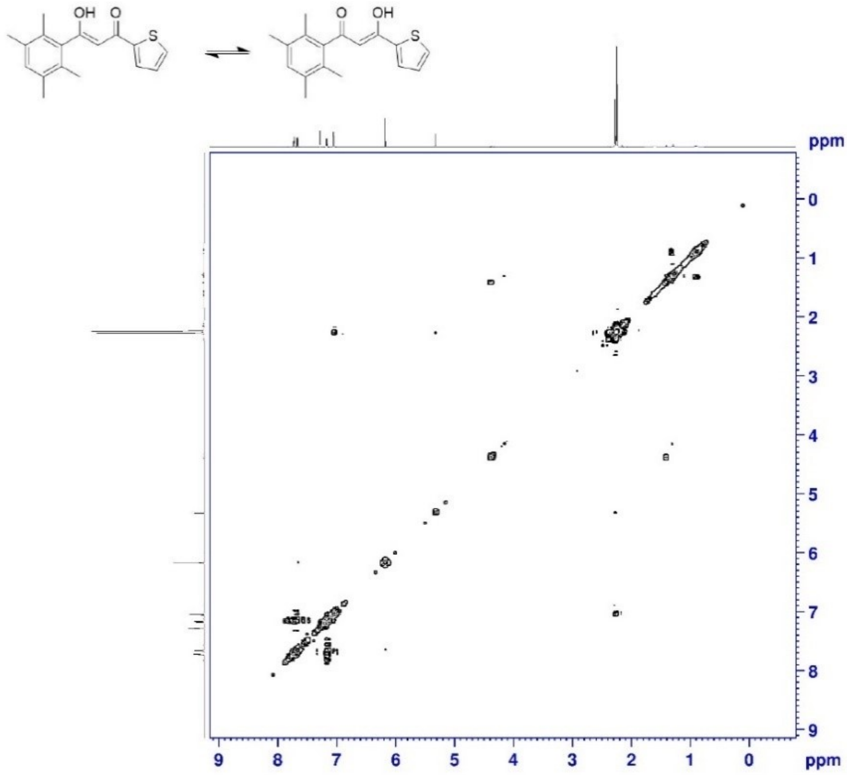

HSQC (126 MHz, $CDCl_3$) of 1-(2,3,5,6-tetramethylphenyl)-3-(thiophen-2-yl)propane-1,3-dione **23a**.

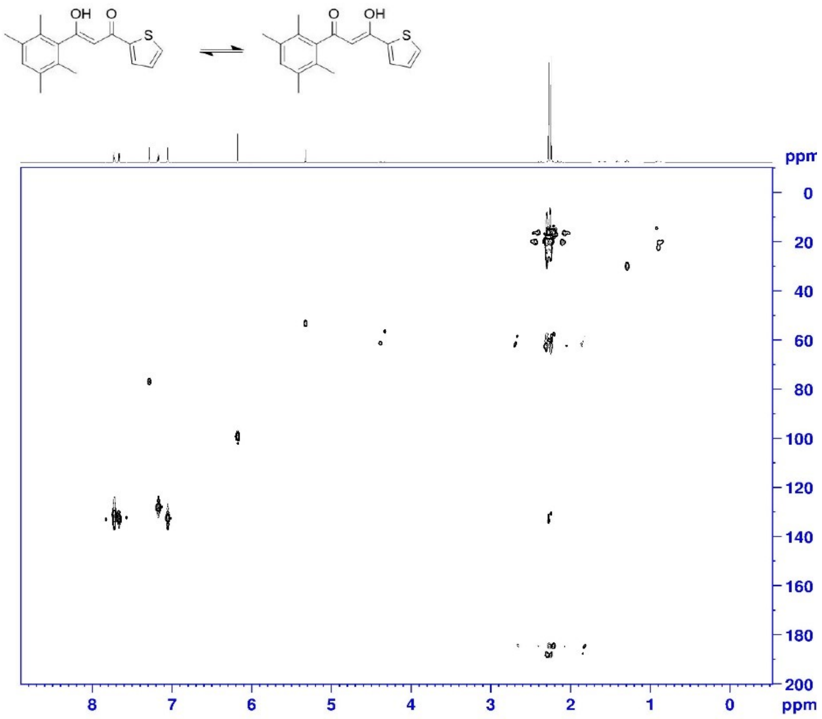

HMBC (126 MHz, $CDCl_3$) of 1-(2,3,5,6-tetramethylphenyl)-3-(thiophen-2-yl)propane-1,3-dione **23a**.

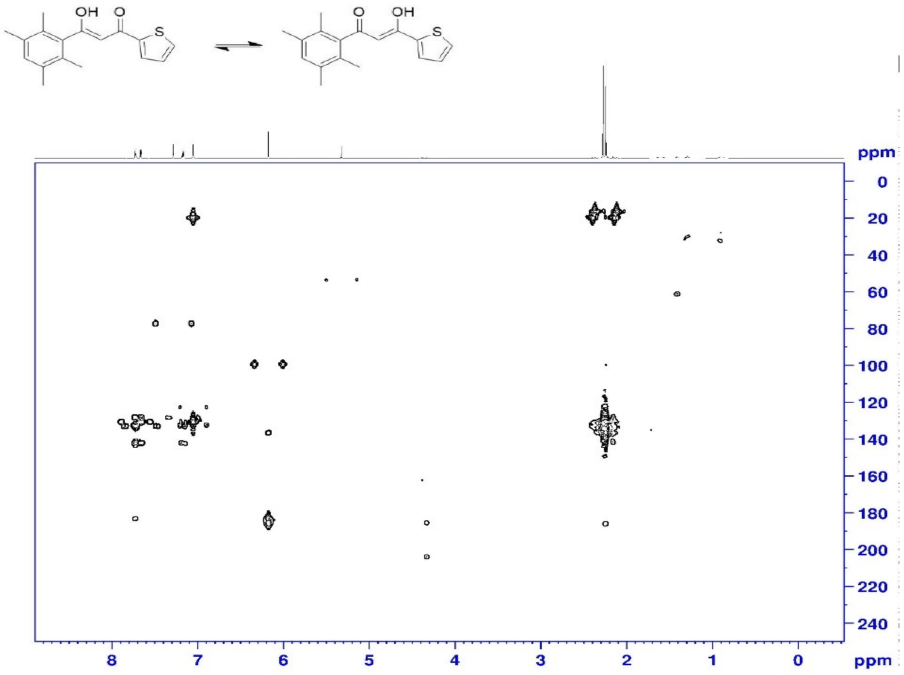

$^{13}C\{^{1}H\}$ NMR (126 MHz, $CDCl_3$) of 1-(2,3,5,6-tetramethylphenyl)-3-(thiophen-2-yl)propane-1,3-dione **23a**.

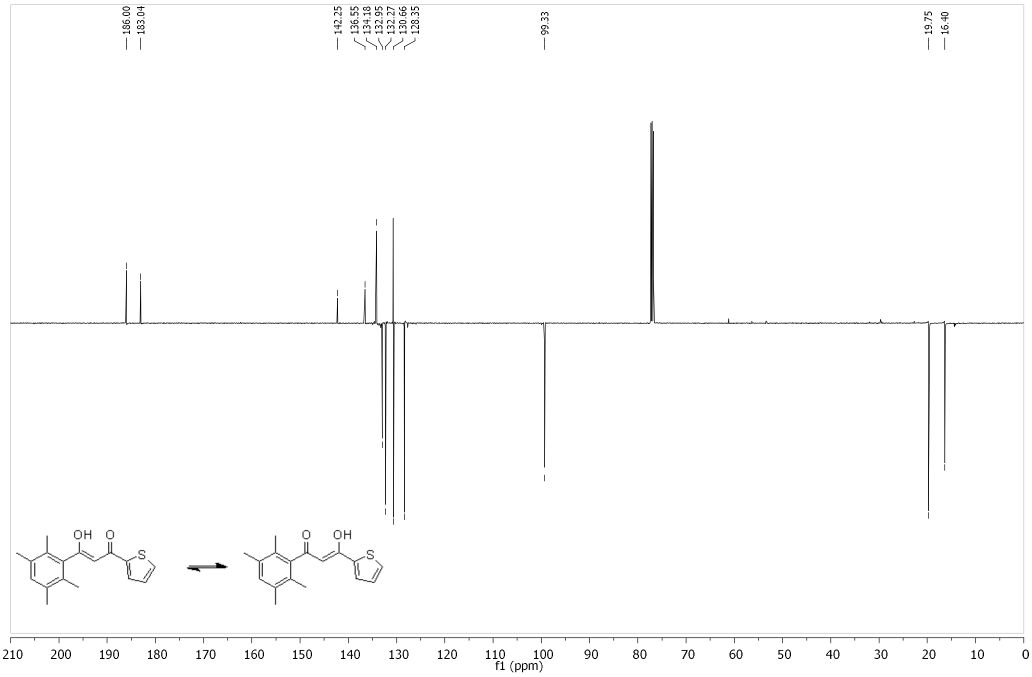

HPLC of racemic of 1-(2,3,5,6-tetramethylphenyl)-3-(thiophen-2-yl)propane-1,3-dione **23a**.

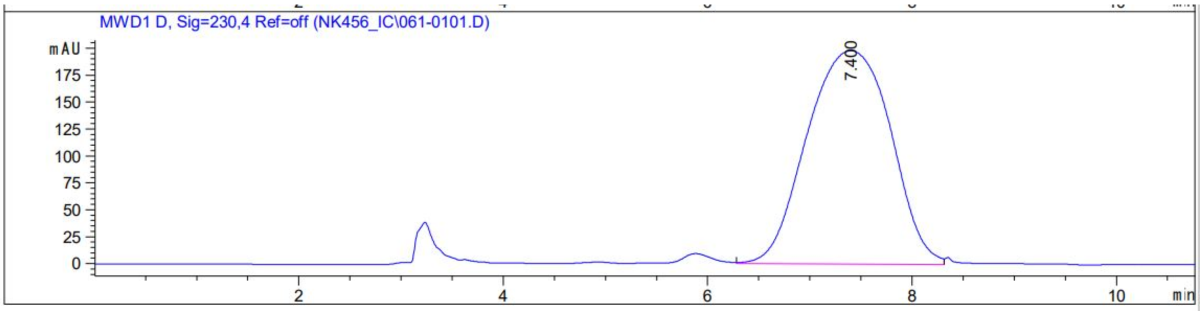

Signal 4: MWD1 D, Sig=230,4 Ref=off

Peak #	RetTime [min]	Type	Width [min]	Area [mAU*s]	Height [mAU]	Area %
1	7.400	VV	0.9534	1.12981e4	198.09972	100.0000
Totals :				1.12981e4	198.09972	

3-Hydroxy-1-(2,3,5,6-tetramethylphenyl)-3-(thiophen-2-yl)propan-1-one 23b.

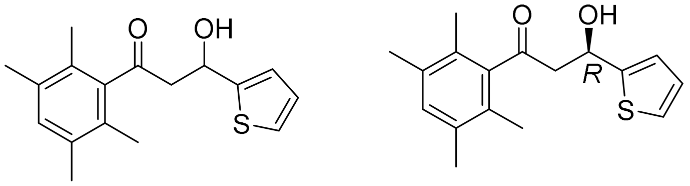

This compound is novel.

Synthesis of a racemic standard: (*R,R*)-3C-Tethered Ru(II)-TsDPEN catalyst (0.4 mg, 0.0007 mmol, 0.5 mol%) and (*S,S*)-3C-tethered Ru(II)-TsDPEN catalyst (0.4 mg, 0.0007 mmol, 0.5 mol%) were added to FA: TEA (5:2 azeotropic mixture, 0.07 mL) at rt and the mixture was stirred under a nitrogen atmosphere for 15 minutes; after which 1-(2,3,5,6-tetramethylphenyl)-3-(thiophen-2-yl)propane-1,3-dione **23a** (40 mg, 0.14 mmol) was added in DCM (0.1 mL). The reaction mixture was stirred under a nitrogen atmosphere and followed by TLC (5:1 hexane: EtOAc). After 48 h, the reaction was quenched using saturated $NaHCO_3$ solution (20 mL). EtOAc (20 mL) was added, and the organic layer was separated. The aqueous layer was extracted with EtOAc (3 x 20 mL) and the combined organic layers were dried ($MgSO_4$) and filtered. The solvent was removed to give the crude product. The product was isolated via flash chromatography on silica eluted with 0-50% EtOAc in petroleum ether to give 3-hydroxy-1-(2,3,5,6-tetramethylphenyl)-3-(thiophen-2-yl)propan-1-one **23b** as a white solid (13.1 mg, 0.05 mmol, 33%). TLC: Rf ca 0.3 (4:1 hexane: EtOAc), strong UV and PMA active; Mp: 92.3°C; HRMS (ESI+) *m/z*: [M+Na]+ Calcd for $C_{17}H_{20}NaO_2S$ 311.1076; Found 311.1068; 2.6 ppm error; ν_{max} 3443, 2919, 2858, 1699 cm^{-1}; ^1H NMR (500 MHz, $CDCl_3$): δ 7.32 – 7.24 (1H, m, ArH), 7.04 (1H, d, *J* = 3.3, ArH), 7.02 – 6.97 (2H, m, ArH), 5.69 – 5.61 (1H, m, ArCH), 3.63 (1H, br. S, OH), 3.33 – 3.17 (2H, m, CH_2), 2.23 (6H, s, CH_3), 2.11 (6H, s, CH_3); $^{13}C\{^1H\}$ NMR (126 MHz, $CDCl_3$): δ 211.5 (C), 146.3 (C), 141.6 (C), 134.6 (C), 132.0 (CH), 128.0 (C), 126.7 (CH), 124.9 (CH), 123.7 (CH), 65.9 (CH), 53.5 (CH_2), 19.4 (CH_3), 15.9 (CH_3); *m/z* (ES-API+) 311.1 (M^+ + Na, 100%); Enantiomeric excess and conversion determined by HPLC analysis (Chiralpak IC, 30 cm x 6 mm column, hexane:iPrOH 90:10, 1.0 mL/min, T = 25°C) ketone 7.4 min, *R* and *S* isomer 11.2 min and 12.7 min.

(*R*)-3-Hydroxy-1-(2,3,5,6-tetramethylphenyl)-3-(thiophen-2-yl)propan-1-one **23b**. (*R,R*)-3C-tethered Ru(II)-TsDPEN catalyst (1.3 mg, 0.0021 mmol, 1 mol%) was added to FA: TEA (5:2 azeotropic mixture, 0.10 mL) at rt and the mixture was stirred

under a nitrogen atmosphere for 10-15 minutes, after which 1-(2,3,5,6-tetramethylphenyl)-3-(thiophen-2-yl)propane-1,3-dione **23a** (60 mg, 0.21 mmol) was added in DCM (0.7 mL). The reaction mixture was stirred under a nitrogen atmosphere for 92 h. The reaction was followed by TLC (9:1 hexane: EtOAc). After 48 h, the reaction was quenched using saturated $NaHCO_3$ solution (20 mL). EtOAc (20 mL) was added, and the organic layer was separated. The aqueous layer was extracted with EtOAc (3 x 20 mL) and the combined organic layers were dried ($MgSO_4$) and filtered. The solvent was removed to give the crude product. The product was isolated via flash chromatography on silica eluted with 0-50% EtOAc in petroleum ether to give (*R*)-3-hydroxy-1-(2,3,5,6-tetramethylphenyl)-3-(thiophen-2-yl)propan-1-one as a white solid (39 mg, 0.14 mmol, 67%). The reaction was also followed by HPLC (Chiralpak IC, 30 cm x 6 mm column, hexane:iPrOH 90:10, 1.0 mL/min, T = 25°C); $[\alpha]_D^{29}$ + 19.1 (c 0.04 in $CHCl_3$); (after 92 h, >99% ee (*R*))

^{1}H NMR (500 MHz, $CDCl_3$) of 3-hydroxy-1-(2,3,5,6-tetramethylphenyl)-3-(thiophen-2-yl)propan-1-one **23b**.

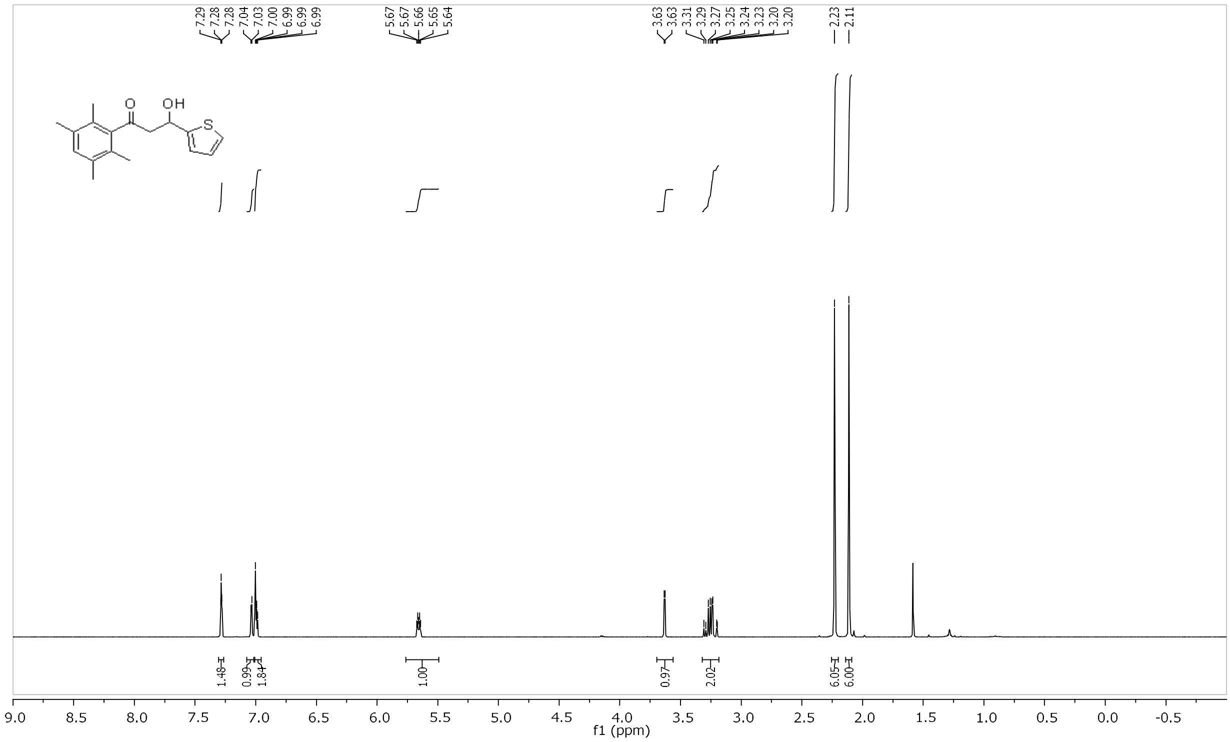

COSY (500 MHz, CDCl$_3$) of 3-hydroxy-1-(2,3,5,6-tetramethylphenyl)-3-(thiophen-2-yl)propan-1-one **23b**.

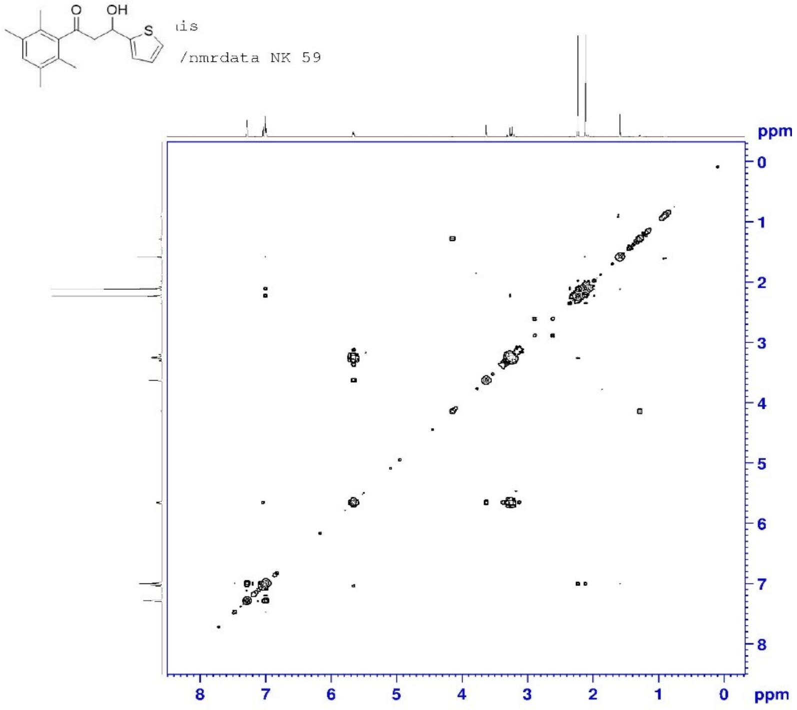

HSQC (126 MHz, CDCl$_3$) of 3-hydroxy-1-(2,3,5,6-tetramethylphenyl)-3-(thiophen-2-yl)propan-1-one **23b**.

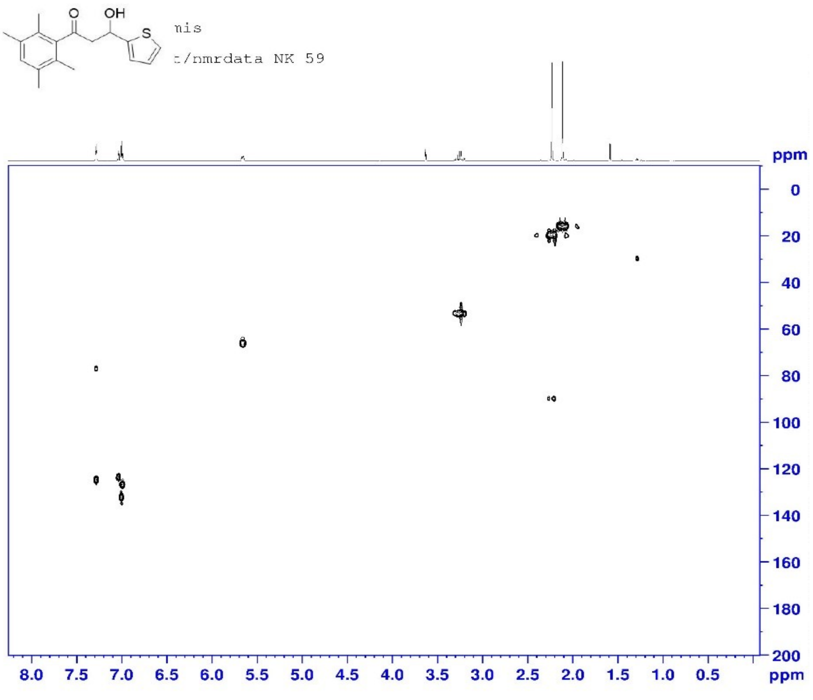

HMBC (126 MHz, $CDCl_3$) of 3-hydroxy-1-(2,3,5,6-tetramethylphenyl)-3-(thiophen-2-yl)propan-1-one **23b**.

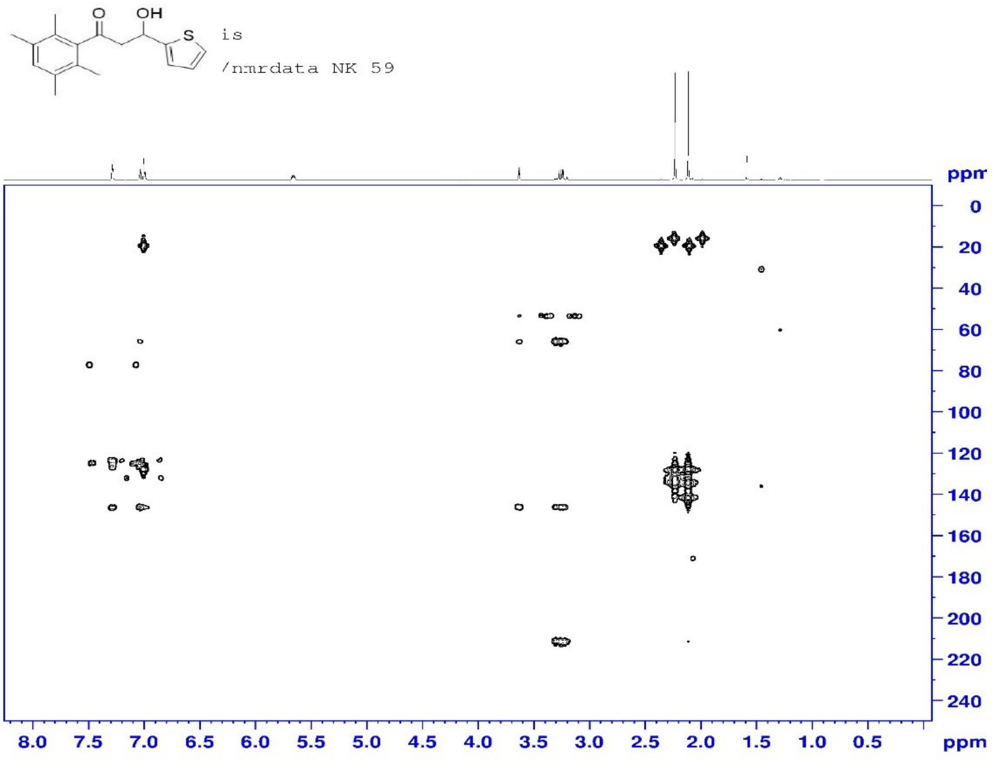

$^{13}C\{^1H\}$ NMR (126 MHz, $CDCl_3$) of 3-hydroxy-1-(2,3,5,6-tetramethylphenyl)-3-(thiophen-2-yl)propan-1-one **23b**.

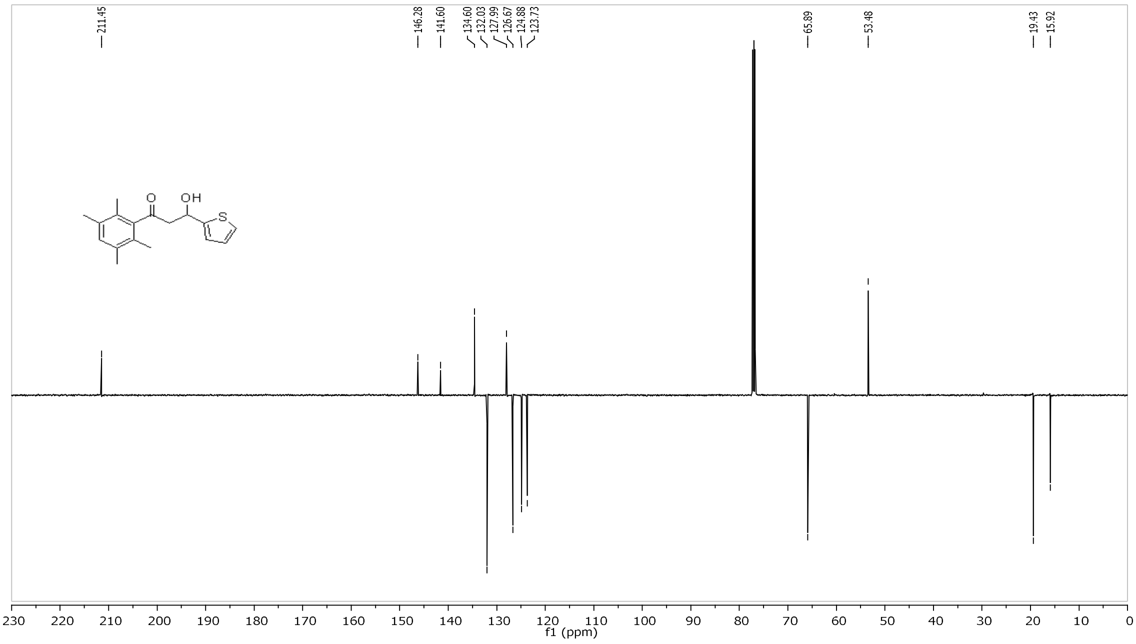

HPLC of racemic of 3-hydroxy-1-(2,3,5,6-tetramethylphenyl)-3-(thiophen-2-yl)propan-1-one **23b**.

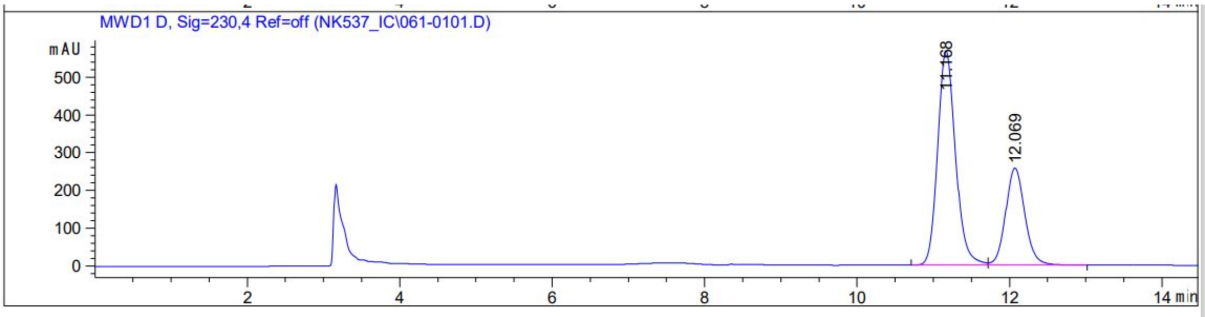

Signal 4: MWD1 D, Sig=230,4 Ref=off

Peak #	RetTime [min]	Type	Width [min]	Area [mAU*s]	Height [mAU]	Area %
1	11.168	BV	0.2526	9263.11719	567.02039	67.3415
2	12.069	VB	0.2677	4492.32764	257.24426	32.6585
Totals :				1.37554e4	824.26465	

HPLC of (*R*)-3-hydroxy-1-(2,3,5,6-tetramethylphenyl)-3-(thiophen-2-yl)propan-1-one **23b**.

(*R,R*)-3C-tethered Ru(II)-TsDPEN catalyst (after 48 h, >99% ee (*R*))

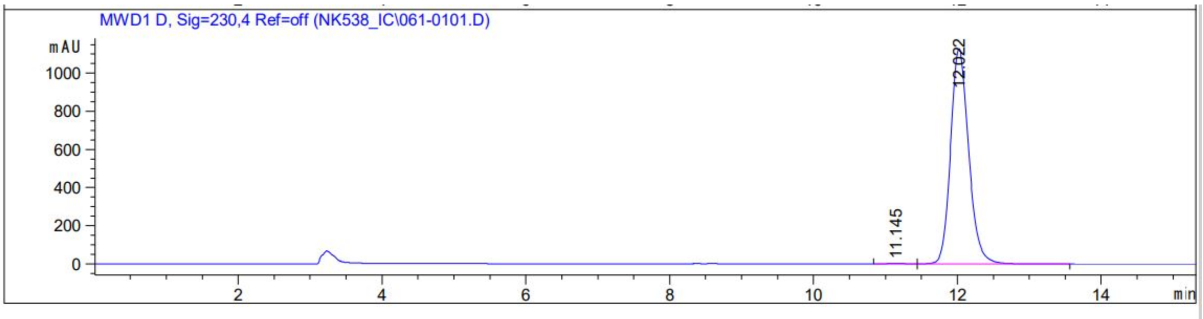

Signal 4: MWD1 D, Sig=230,4 Ref=off

Peak #	RetTime [min]	Type	Width [min]	Area [mAU*s]	Height [mAU]	Area %
1	11.145	BV	0.2365	29.17353	1.92831	0.1481
2	12.022	VB	0.2675	1.96759e4	1128.25769	99.8519
Totals :				1.97051e4	1130.18600	

X-ray Crystallography Data for (*R*)-23b. CCDC Deposition Number 2276987

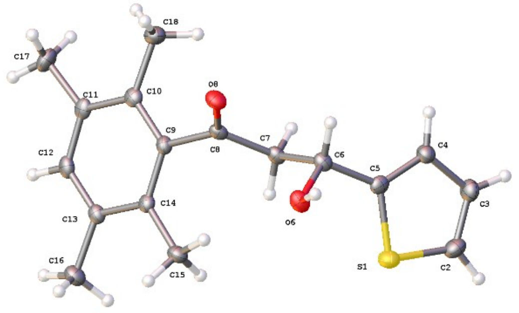

Solid state structure of **23b** with atom labelling and thermal ellipsoids drawn at 50% probability level.

Crystal structure determination of 23b

The asymmetric unit contained the alcohol, there are two molecules in the unit cell related by a crystallographic 2(1) axis.

The OH was detected in a difference map and refined with restraints. It forms short contacts tabulated below.

Specified hydrogen bonds (with esds except fixed and riding H)

D-H	H...A	D...A	<(DHA)	
0.84	2.00	2.836(2)	171.6	O6-H6A...O8

Symmetry operator used to generate symmetry equivalent atom discussed in above contact was $1 1-X,-0.5+Y,2-Z

The Flack parameter and associated Hooft y parameter were.

Hooft y: -0.001(4) Olex2

Flack x: 0.017(9) Shelx2019 (Parsons'method used as lower error)

[Flack x = 0.006(19) by classical fit to all intensities

0.017(9) from 1211 selected quotients (Parsons' method)

Hooft y: -0.001(4) Olex2]

These are small with a small error so you can be confident in the assignment of the handedness of the crystal chosen.

Experimental

Single crystals of $C_{17}H_{20}O_2S$ **23b** were grown from a diisopropyl ether solution saturated with pet ether. A suitable crystal was selected and mounted on a glass fibre with Fomblin oil and placed on a Rigaku Oxford Diffraction SuperNova diffractometer with a dual source microfocus (Cu at zero) equipped with an Hypix-6000HE Hybrid pixel array detector.

The crystal was kept at 100(2) K during data collection. Using Olex2 [1], the structure was solved with the SHELXT [2] structure solution program using Intrinsic Phasing and refined with the SHELXL [3] refinement package using Least Squares minimisation.

1. Dolomanov, O.V., Bourhis, L.J., Gildea, R.J, Howard, J.A.K. & Puschmann, H. (2009), J. Appl. Cryst. 42, 339-341.
2. Sheldrick, G.M. (2015). Acta Cryst. A71, 3-8.
3. Sheldrick, G.M. (2015). Acta Cryst. C71, 3-8.

Crystal Data for $C_{17}H_{20}O_2S$ (M =288.39 g/mol): monoclinic, space group $P2_1$ (no. 4), a = 11.06745(7) Å, b = 5.39352(5) Å, c = 12.36808(8) Å, β = 92.4822(6)°, V = 737.590(9) Å^3, Z = 2, T = 100(2) K, μ(Cu Kα) = 1.930 mm^{-1}, $Dcalc$ = 1.299 g/cm^3, 22897 reflections measured (7.154° ≤ 2Θ ≤ 156.39°), 2970 unique (R_{int} = 0.0472, R_{sigma} = 0.0195) which were used in all calculations. The final R_1 was 0.0311 (I > 2σ(I)) and wR_2 was 0.0797 (all data).

**Table 1 Crystal data and structure refinement for 23b.**	
Identification code (local)	nk11
Empirical formula	$C_{17}H_{20}O_2S$
Formula weight	288.39
Temperature/K	100(2)
Crystal system	Monoclinic
Space group	$P2_1$
a/Å	11.06745(7)

b/Å	5.39352(5)
c/Å	12.36808(8)
α/°	90
β/°	92.4822(6)
γ/°	90
Volume/Å^3	737.590(9)
Z	2
ρ_{calc}g/cm^3	1.299
μ/mm^{-1}	1.930
F(000)	308.0
Crystal size/mm^3	0.25 × 0.2 × 0.1
Radiation	Cu Kα (λ = 1.54184)
2Θ range for data collection/°	7.154 to 156.39
Index ranges	-13 ≤ h ≤ 14, -6 ≤ k ≤ 6, -15 ≤ l ≤ 15
Reflections collected	22897
Independent reflections	2970 [R_{int} = 0.0472, R_{sigma} = 0.0195]
Data/restraints/parameters	2970/1/186
Goodness-of-fit on F^2	1.045
Final R indexes [I>=2σ (I)]	R_1 = 0.0311, wR_2 = 0.0797
Final R indexes [all data]	R_1 = 0.0311, wR_2 = 0.0797
Largest diff. peak/hole / e Å^{-3}	0.19/-0.27
Flack parameter	0.017(9)

1-(Furan-2-yl)-3-(2,3,5,6-tetramethylphenyl)propane-1,3-dione 24a.

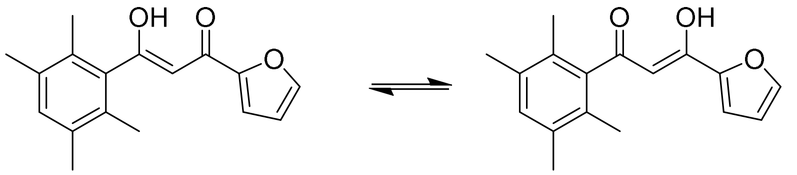

This compound is novel. To a solution of sodium hydride (454 mg, 60% dispersion in mineral oil, 11.3 mmol) in THF (3 mL) at 0 °C was added dropwise a solution of 1-(2,3,5,6-tetramethylphenyl)ethan-1-one **7** (400 mg, 2.3 mmol) in THF (2 mL). The reaction mixture was stirred under a nitrogen atmosphere at 0 °C for 30 min and then stirred under a nitrogen atmosphere at rt for 30 min, after which ethyl furan-2-carboxylate (1.60 g, 11.3 mmol) was added dropwise at 0 °C. The reaction mixture was refluxed at 66 °C and left stirring under the nitrogen atmosphere overnight. The reaction was followed by TLC (4:1 hexane: EtOAc). The mixture was quenched by 2M HCl solution (20 mL). EtOAc (20 mL) was added, and the organic layer was separated. The aqueous layer was extracted with EtOAc (3 × 20 mL), and the combined organic layers were washed with saturated $NaHCO_3$ solution (2 × 20 mL) and brine (20 mL), dried ($MgSO_4$) and filtered. Solvent was removed to give the crude product. The product was isolated via flash chromatography on silica eluted with 0-1.5% EtOAc in hexane to give 1-(furan-2-yl)-3-(2,3,5,6-tetramethylphenyl)propane-1,3-dione **24a** as a yellow solid (467 mg, 1.73 mmol, 76%). TLC: Rf ca 0.19 (9:1 hexane: EtOAc), strong UV and $KMnO_4$; Mp: 106.3 °C; HRMS (ESI+) *m/z*: [M+Na]+ Calcd for $C_{17}H_{18}NaO_3$ 293.1148; Found 293.1141; 2.3 ppm error; ν_{max} 3127, 2997, 2964, 2921 cm^{-1}; enol: keto = 100:0; ^{1}H NMR (500 MHz, $CDCl_3$): δ 7.61 (1H, s, CH), 7.24 (1H, d, *J* = 3.6, ArH), 7.03 (1H, s, ArH), 6.60 (1H, m, ArH), 6.22 (1H, s, CH of enol form), 2.27 (6H, s, CH_3), 2.23 (6H, s, CH_3); $^{13}C\{^1H\}$ NMR (126 MHz, $CDCl_3$): δ 188.0 (C), 177.7 (C), 150.9 (C), 146.3 (CH), 137.0 (C), 134.1 (C), 132.2 (CH), 130.5 (C), 116.2 (CH), 112.6 (CH), 99.0 (CH), 19.7 (CH_3), 16.4 (CH_3); *m/z* (ES-API+) 293.1 (M^+ + Na, 100%); Enantiomeric excess and conversion determined by HPLC analysis (Chiralpak OJ, 30 cm x 6 mm column, hexane:iPrOH 90:10, 1.0 mL/min, T = 25 °C) ketone 7.6 min, *R* and *S* isomer 8.8 min and 13.3 min.

^{1}H NMR (500 MHz, $CDCl_3$) of 1-(furan-2-yl)-3-(2,3,5,6-tetramethylphenyl)propane-1,3-dione **24a**.

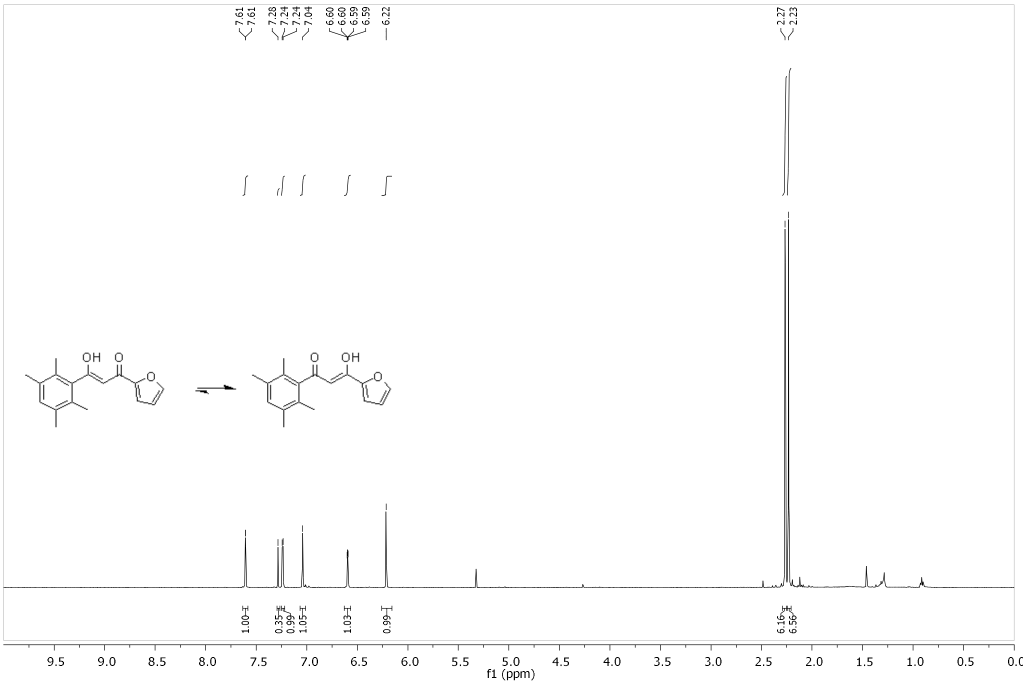

COSY (500 MHz, $CDCl_3$) of 1-(furan-2-yl)-3-(2,3,5,6-tetramethylphenyl)propane-1,3-dione **24a**.

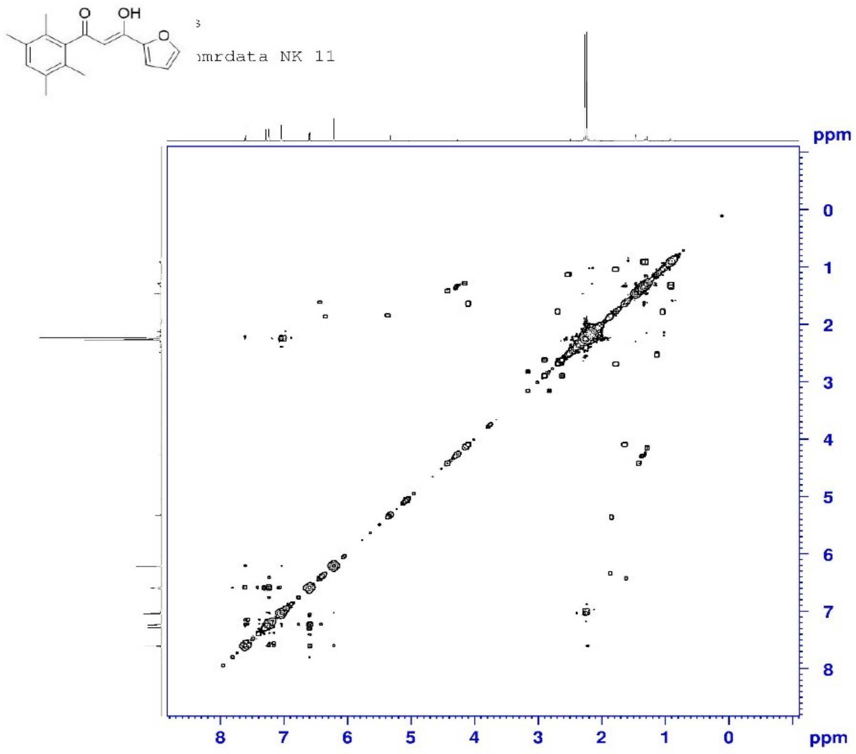

HMBC (126 MHz, $CDCl_3$) of 1-(furan-2-yl)-3-(2,3,5,6-tetramethylphenyl)propane-1,3-dione **24a**.

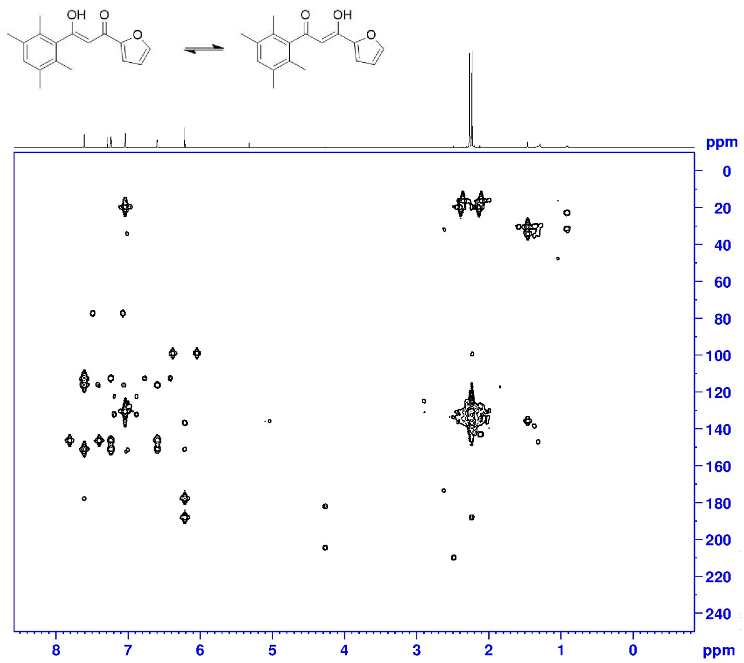

HSQC (126 MHz, $CDCl_3$) of 1-(furan-2-yl)-3-(2,3,5,6-tetramethylphenyl)propane-1,3-dione **24a**.

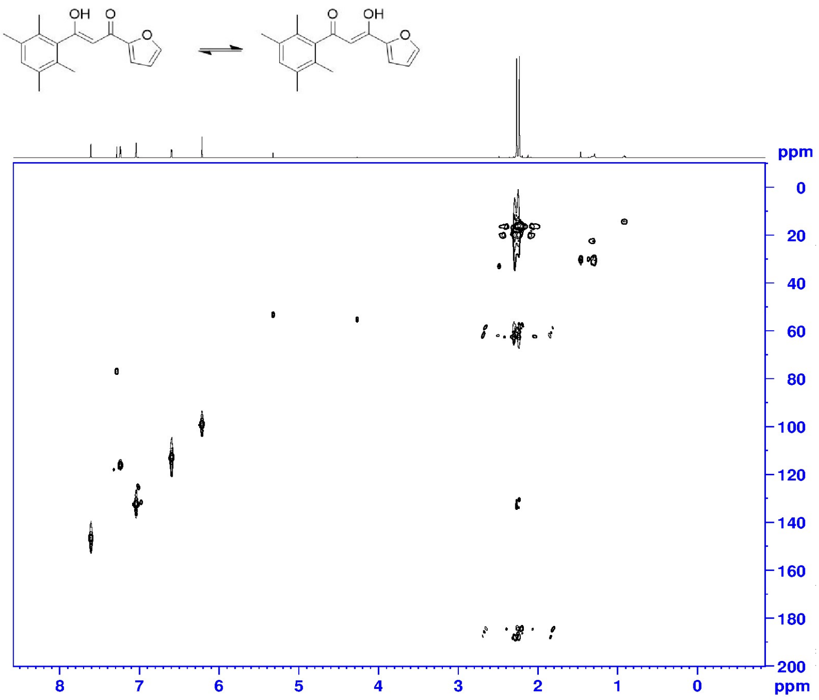

$^{13}C\{^1H\}$ NMR (126 MHz, $CDCl_3$) of 1-(furan-2-yl)-3-(2,3,5,6-tetramethylphenyl)propane-1,3-dione **24a**.

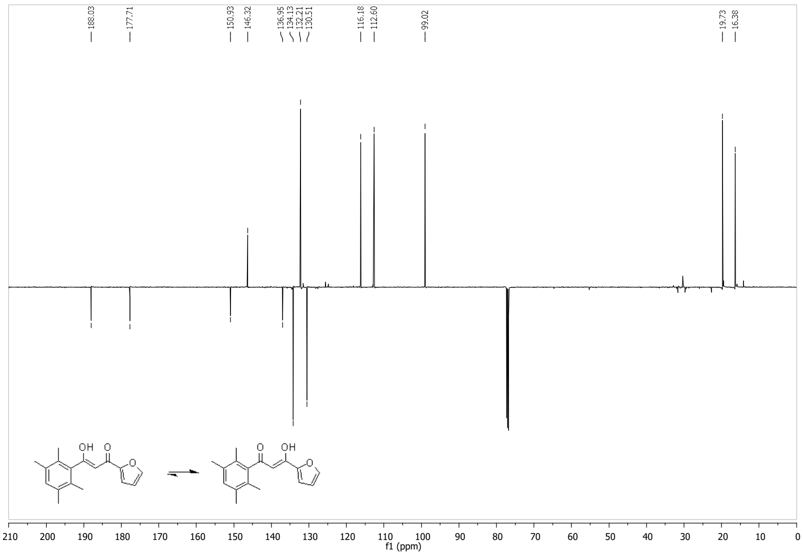

HPLC of of 1-(furan-2-yl)-3-(2,3,5,6-tetramethylphenyl)propane-1,3-dione **24a**.

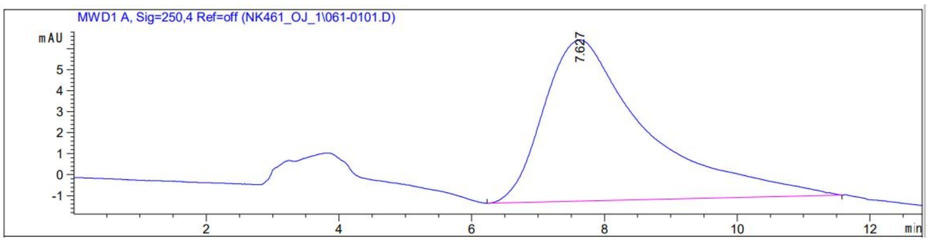

Signal 1: MWD1 A, Sig=250,4 Ref=off

Peak #	RetTime [min]	Type	Width [min]	Area [mAU*s]	Height [mAU]	Area %
1	7.627	BB	1.2813	830.39667	7.69302	100.0000

Totals : 830.39667 7.69302

3-(Furan-2-yl)-3-hydroxy-1-(2,3,5,6-tetramethylphenyl)propan-1-one 24b.

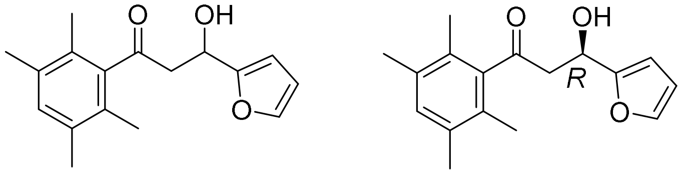

This compound is novel. **Synthesis of a racemic standard**: (*R,R*)-3C-Tethered Ru(II)-TsDPEN catalyst (1.1 mg, 0.002 mmol, 0.5 mol%) and (*S,S*)-3C-tethered Ru(II)-TsDPEN catalyst (1.1 mg, 0.002 mmol, 0.5 mol%) were added to FA: TEA (5:2 azeotropic mixture, 0.18 mL) at rt and the mixture was stirred under a nitrogen atmosphere for 15 minutes; after which 1-(furan-2-yl)-3-(2,3,5,6-tetramethylphenyl)propane-1,3-dione **24a** (100 mg, 0.37 mmol) was added in DCM (0.3 mL). The reaction mixture was stirred under a nitrogen atmosphere and followed by TLC (5:1 hexane: EtOAc). After 78 h, the reaction was quenched using saturated $NaHCO_3$ solution (20 mL). EtOAc (20 mL) was added, and the organic layer was separated. The aqueous layer was extracted with EtOAc (3 x 20 mL) and the combined organic layers were dried ($MgSO_4$) and filtered. The solvent was removed to give the crude product. The product was isolated via flash chromatography on silica eluted with 0-50% EtOAc in petroleum ether to give 3-(furan-2-yl)-3-hydroxy-1-(2,3,5,6-tetramethylphenyl)propan-1-one **24b** as a yellow solid (27.6 mg, 0.100 mmol, 28%). TLC: Rf ca 0.15 (4:1 hexane: EtOAc), strong UV and PMA; Mp: 76.6°C; HRMS (ESI+) *m/z*: [M+Na]+ Calcd for $C_{17}H_{20}NaO_3$ 295.1305; Found 295.1301; 1.2 ppm error; ν_{max} 3538, 3127, 2940, 1759 cm^{-1}; ^1H NMR (500 MHz, $CDCl_3$): δ 7.40 (1H, d, *J* = 0.9, ArH), 7.00 (1H, s, ArH), 6.41 – 6.29 (2H, m, ArH), 5.41 (1H, d, *J* = 8.9, ArCH), 3.44 (1H, d, *J* = 3.5, OH), 3.33 (1H, dd, *J* = 18.8, 9.0, CHA*HB*), 3.20 (1H, dd, *J* = 18.8, 3.0, C*HA*HB), 2.23 (6H, s, CH_3), 2.10 (6H, s, CH_3); $^{13}C\{^1H\}$ NMR (126 MHz, $CDCl_3$): δ 211.4 (C), 154.8 (C), 142.2 (CH), 141.7 (C), 134.6 (C), 132.0 (CH), 128.0 (C), 110.3 (CH), 106.5 (CH), 63.6 (CH), 49.9 (CH_2), 19.4 (CH_3), 15.9 (CH_3); *m/z* (ES-API+) 295.1 (M^+ + Na, 100%); Enantiomeric excess and conversion determined by HPLC analysis (Chiralpak OJ, 30 cm x 6 mm column, hexane:iPrOH 90:10, 1.0 mL/min, T = 25°C) ketone 7.6 min, *R* and *S* isomer 8.8 min and 13.3 min.

(*R*)-3-(Furan-2-yl)-3-hydroxy-1-(2,3,5,6-tetramethylphenyl)propan-1-one **24b.**

(*R,R*)-3C-tethered Ru(II)-TsDPEN catalyst (3.4 mg, 0.0055 mmol, 1 mol%) was added to FA: TEA (5:2 azeotropic mixture, 0.3 mL) at rt and the mixture was stirred

under a nitrogen atmosphere for 10-15 minutes; after which 1-(furan-2-yl)-3-(2,3,5,6-tetramethylphenyl)propane-1,3-dione **24a** (150 mg, 0.55 mmol) was added in DCM (1.2 mL). The reaction mixture was stirred under a nitrogen atmosphere for 92 h then quenched using saturated $NaHCO_3$ solution (20 mL). EtOAc (20 mL) was added, and the organic layer was separated. The aqueous layer was extracted with EtOAc (3 x 20 mL) and the combined organic layers were dried ($MgSO_4$) and filtered. The solvent was removed to give the crude product. The product was isolated via flash chromatography on silica eluted with 0-50% EtOAc in petroleum ether to give (*R*)-3-(furan-2-yl)-3-hydroxy-1-(2,3,5,6-tetramethylphenyl)propan-1-one **24b** as a yellow solid (95.5 mg, 0.35 mmol, 64%). The reaction was also followed by HPLC (Chiralpak OJ, 30 cm x 6 mm column, hexane:iPrOH 90:10, 1.0 mL/min, T = 25°C); $[\alpha]_D^{29}$ + 14.3 (c 0.042 in $CHCl_3$); (after 78 h, >99% ee (*R*)).

^1H NMR (500 MHz, $CDCl_3$) of 3-(furan-2-yl)-3-hydroxy-1-(2,3,5,6-tetramethylphenyl)propan-1-one **24b.**

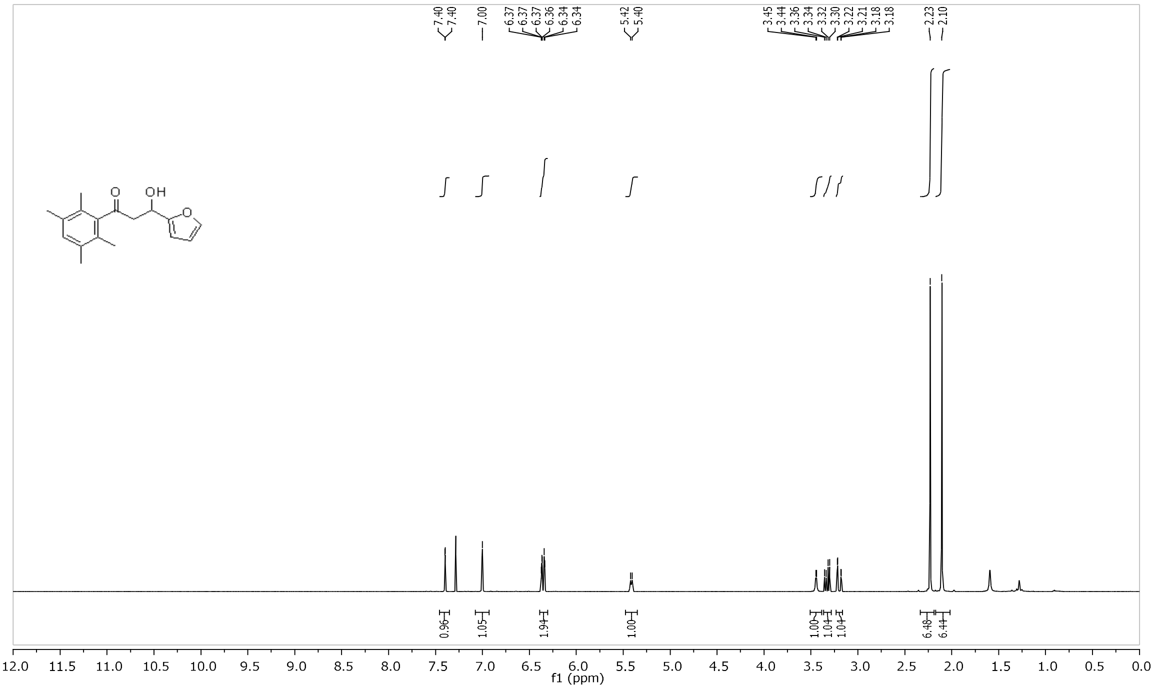

COSY (500 MHz, $CDCl_3$) of 3-(furan-2-yl)-3-hydroxy-1-(2,3,5,6-tetramethylphenyl)propan-1-one **24b.**

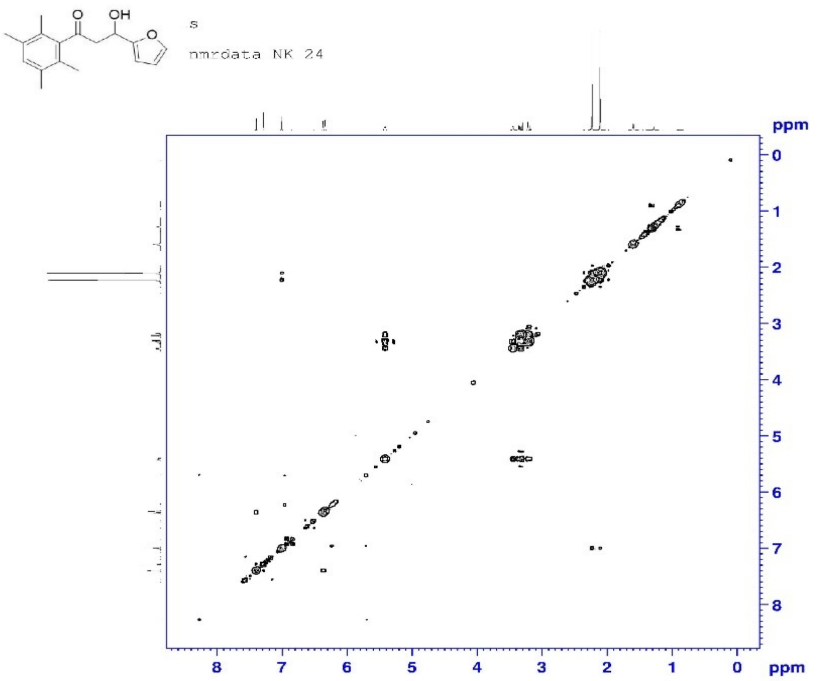

HSQC (126 MHz, $CDCl_3$) of 3-(furan-2-yl)-3-hydroxy-1-(2,3,5,6-tetramethylphenyl)propan-1-one **24b.**

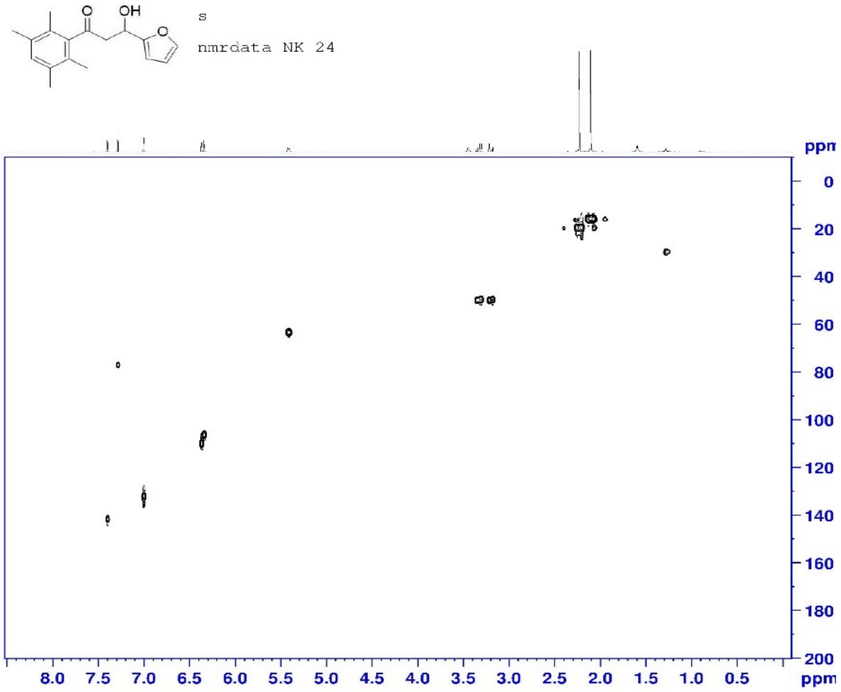

HMBC (126 MHz, $CDCl_3$) of 3-(furan-2-yl)-3-hydroxy-1-(2,3,5,6-tetramethylphenyl)propan-1-one **24b.**

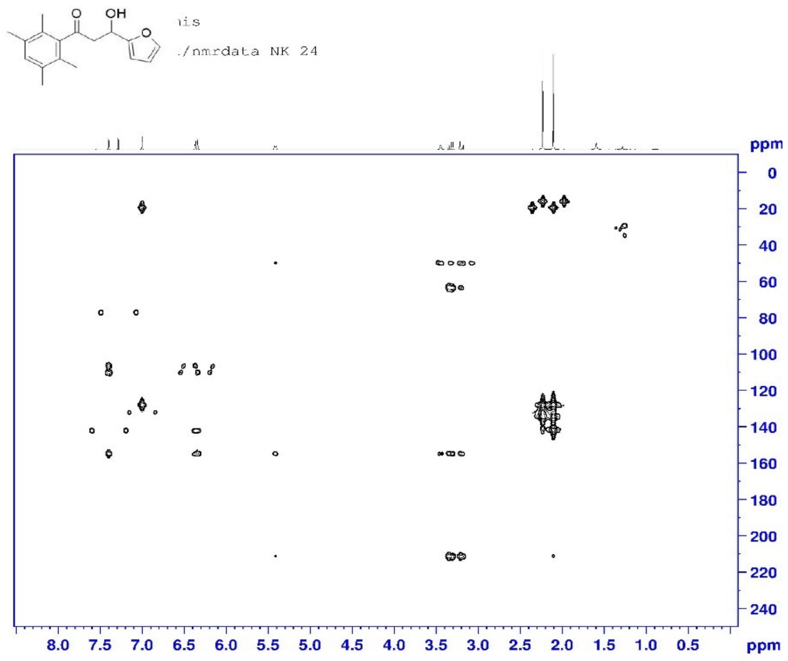

$^{13}C\{^{1}H\}$ NMR (126 MHz, $CDCl_3$) of 3-(furan-2-yl)-3-hydroxy-1-(2,3,5,6-tetramethylphenyl)propan-1-one **24b.**

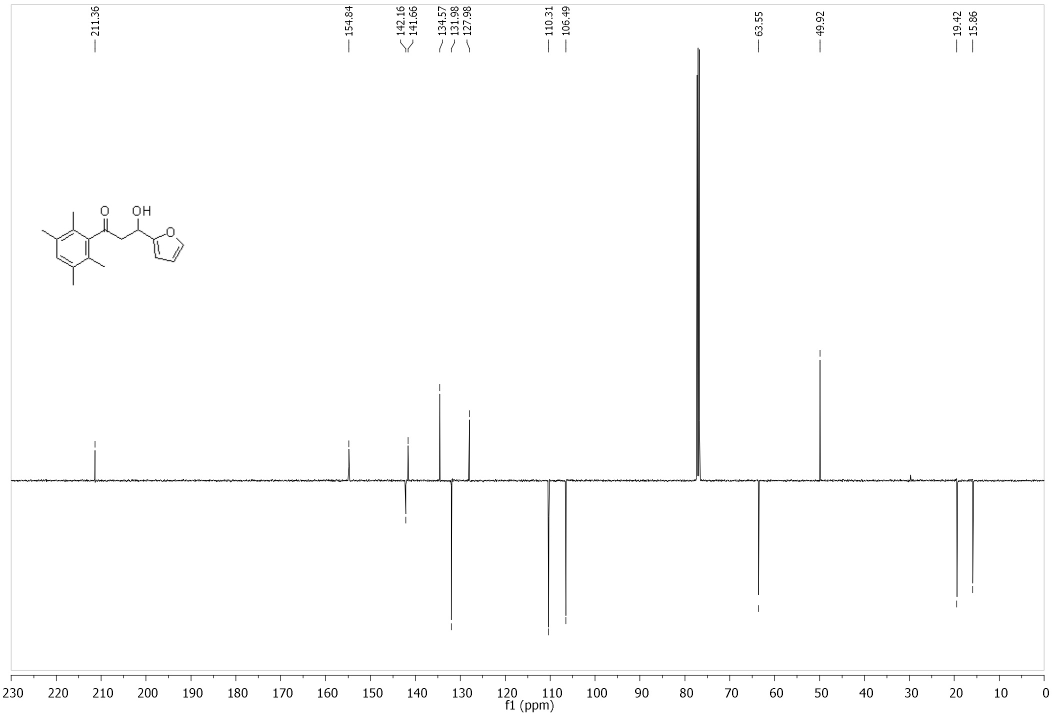

HPLC of racemic of 3-(furan-2-yl)-3-hydroxy-1-(2,3,5,6-tetramethylphenyl)propan-1-one **24b.**

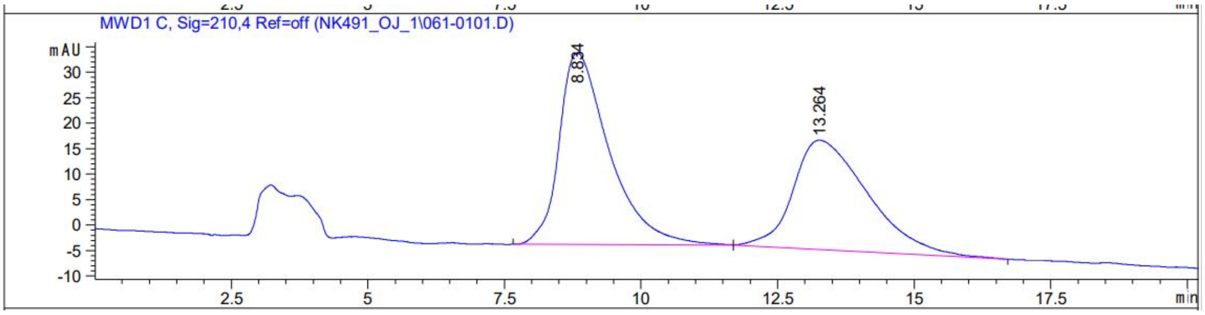

Signal 3: MWD1 C, Sig=210,4 Ref=off

Peak #	RetTime [min]	Type	Width [min]	Area [mAU*s]	Height [mAU]	Area %
1	8.834	BB	0.9119	2403.19116	37.58863	54.0763
2	13.264	BB	1.1516	2040.88745	21.41895	45.9237

Totals : 4444.07861 59.00758

HPLC of (*R*)- of 3-(furan-2-yl)-3-hydroxy-1-(2,3,5,6-tetramethylphenyl)propan-1-one **24b.**

(*R,R*)-3C-tethered Ru(II)-TsDPEN catalyst (after 78 h, >99% ee (*R*)).

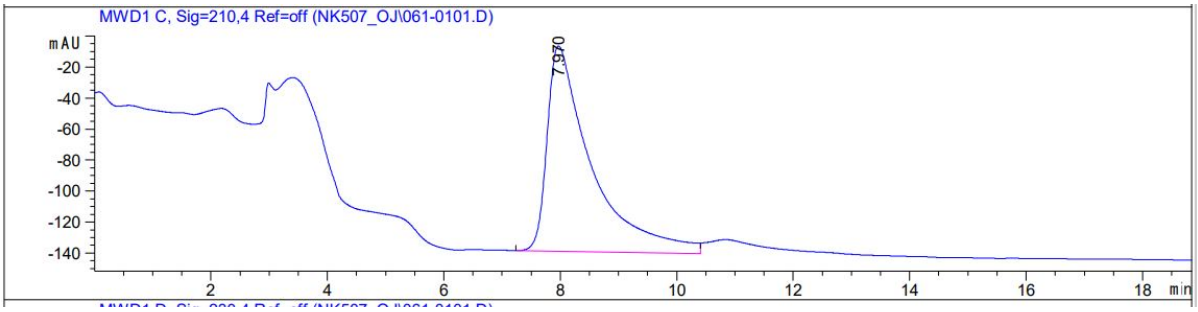

Signal 3: MWD1 C, Sig=210,4 Ref=off

Peak #	RetTime [min]	Type	Width [min]	Area [mAU*s]	Height [mAU]	Area %
1	7.970	BV	0.7270	7005.91992	132.46776	100.0000

Totals : 7005.91992 132.46776

Procedures for 4-hydroxy-4-aryl-1-(2,3,5,6-tetramethylphenyl)butan-1-ones 27b-30b.

Synthesis of 1,4-diketones, overall procedure:

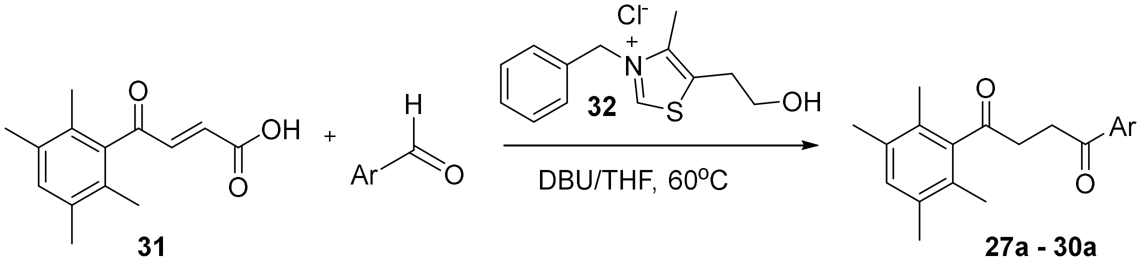

(*E*)-4-Oxo-4-(2,3,5,6tetramethylphenyl)but-2-enoic acid 31.

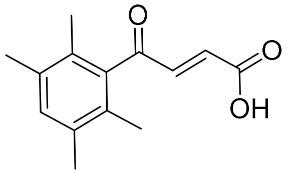

This compound has been reported but not fully characterized. Vitorović-Todorović, M. D.; Erić-Nikolić, A.; Kolundžija, B.; Hamel, E.; Ristić, S.; Juranić, I. O.; Drakulić, B. J., (E)-4-Aryl-4-oxo-2-butenoic acid amides, chalcone–aroylacrylic acid chimeras: Design, antiproliferative activity and inhibition of tubulin polymerization. *Eur. J. Med. Chem.* **2013,** *62*, 40-50.

To a solution of Durene (1.00 g, 7.5 mmol) in DCM (30 mL) and maleic Anhydride (735 mg, 7.5 mmol), $AlCl_3$ (2.00 g, 15 mmol) added portionwise over 10 minutes. The reaction mixture was warmed to rt and left stirring under the nitrogen atmosphere overnight. The mixture was poured into ice water (30 mL). DCM (50 mL) was added, and the organic layer was separated. The aqueous layer was extracted with DCM (3 × 50 mL), and the combined organic layers were dried ($MgSO_4$) and filtered. Solvent was removed to give the crude product. The crude product was dissolved using saturated $NaHCO_3$ solution, then 6M HCl was added until pH=2, and the solution was filtered using Büchner filtration to give the product **31** as a yellow solid (691 mg, 2.98 mmol, 40%); Mp: 167.5°C; HRMS (ESI-) *m/z*: [M-H]- Calcd for $C_{14}H_{15}O_3$ 231.1027; Found 231.1017; 4.1 ppm error; ν_{max} 2913, 1727, 1630, 1467 cm^{-1}; ^{1}H NMR (500 MHz, $CDCl_3$): δ 11.37 (1H, s, OH), 7.31 (1H, d, J = 16.0, CH), 7.04 (1H, s, ArH), 6.37 (1H, d, J = 16.0, CH), 2.23 (6H, s, CH_3), 2.05 (6H, s, CH_3); $^{13}C\{^1H\}$ NMR (126 MHz, $CDCl_3$): δ 201.7 (C), 170.9 (C), 143.0 (CH), 138.7 (C), 134.6 (C), 133.2 (CH),

132.6 (CH), 129.5 (C), 19.4 (CH_3), 16.3 (CH_3) ; *m/z* (ES-API-) 231.1 ((M – H)-, 100%). The data matched that reported.

^{1}H NMR (500 MHz, $CDCl_3$) of **(*E*)**-4-oxo-4-(2,3,5,6-tetramethylphenyl)but-2-enoic acid **31**.

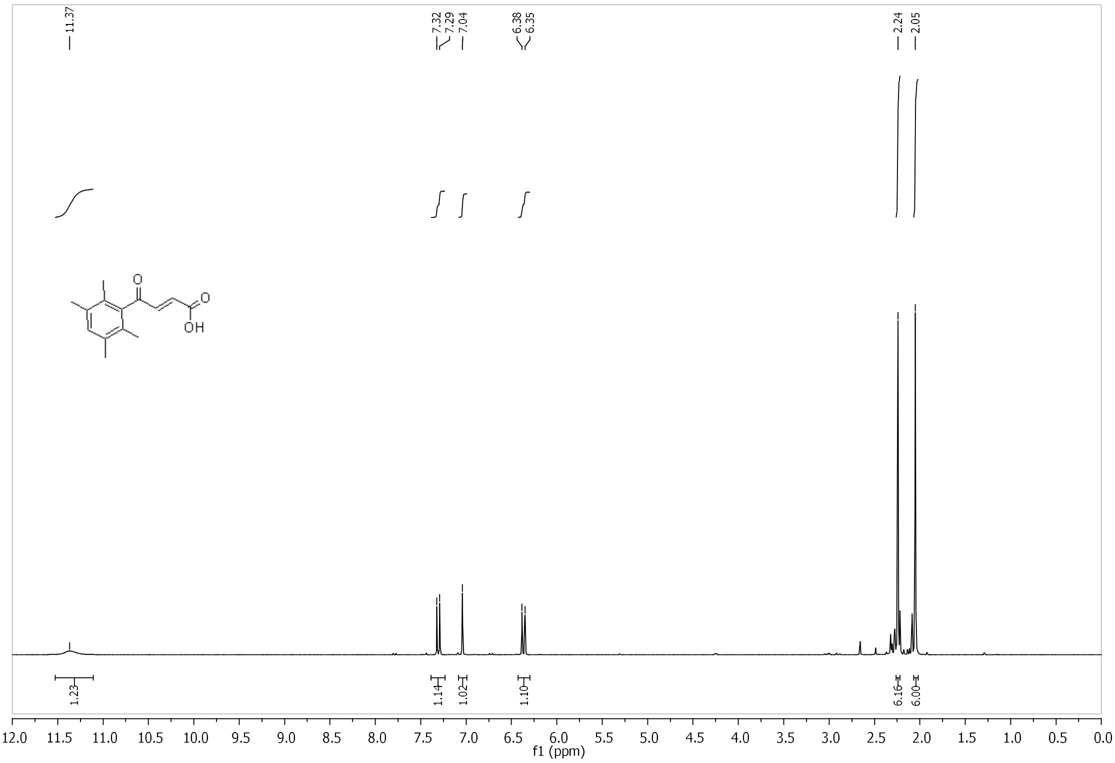

COSY (500 MHz, $CDCl_3$) of (*E*)-4-oxo-4-(2,3,5,6-tetramethylphenyl)but-2-enoic acid **31**.

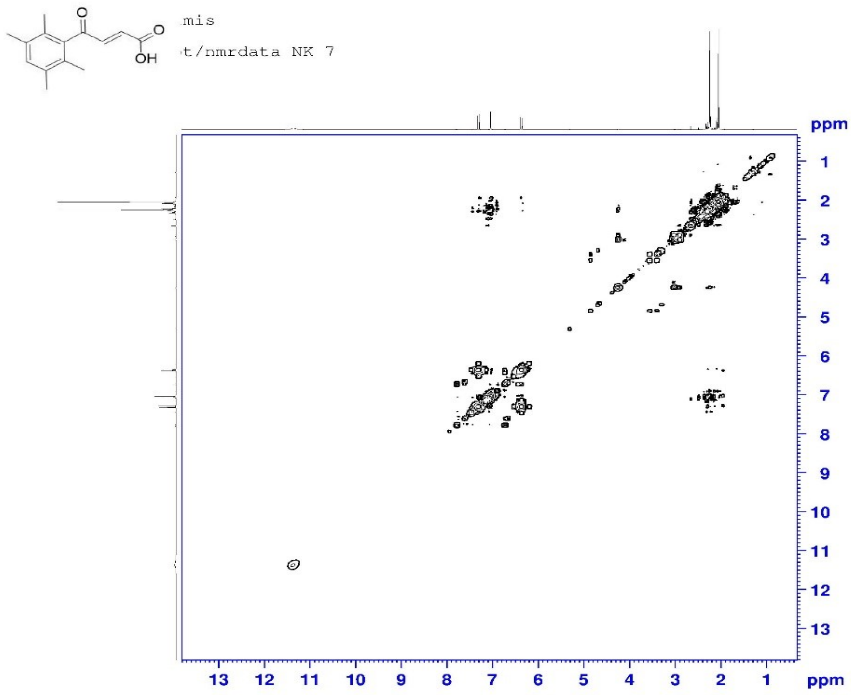

HSQC (126 MHz, $CDCl_3$) of (*E*)-4-oxo-4-(2,3,5,6tetramethylphenyl)but-2-enoic acid **31**.

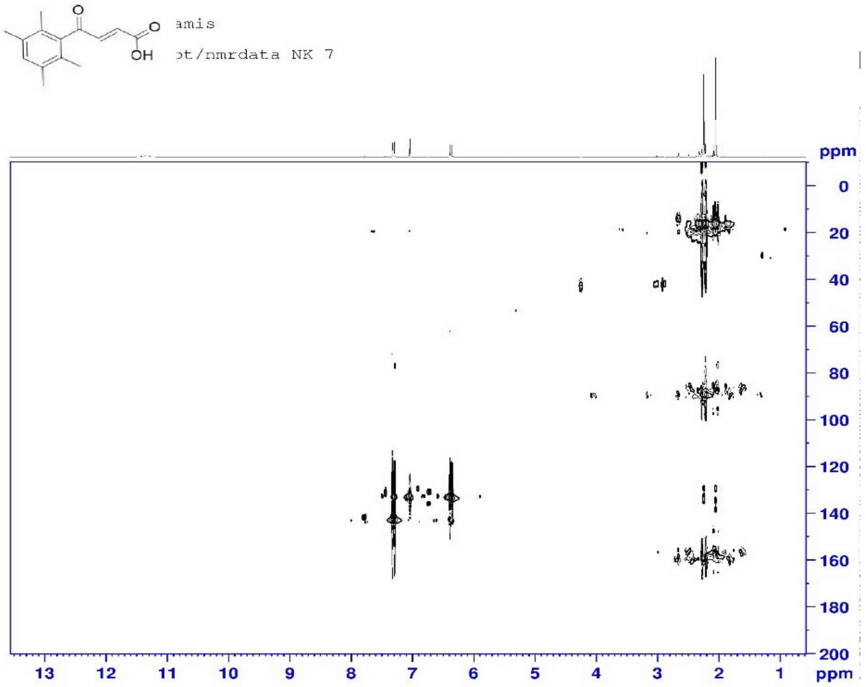

HMBC (126 MHz, $CDCl_3$) of (*E*)-4-oxo-4-(2,3,5,6tetramethylphenyl)but-2-enoic acid **31**.

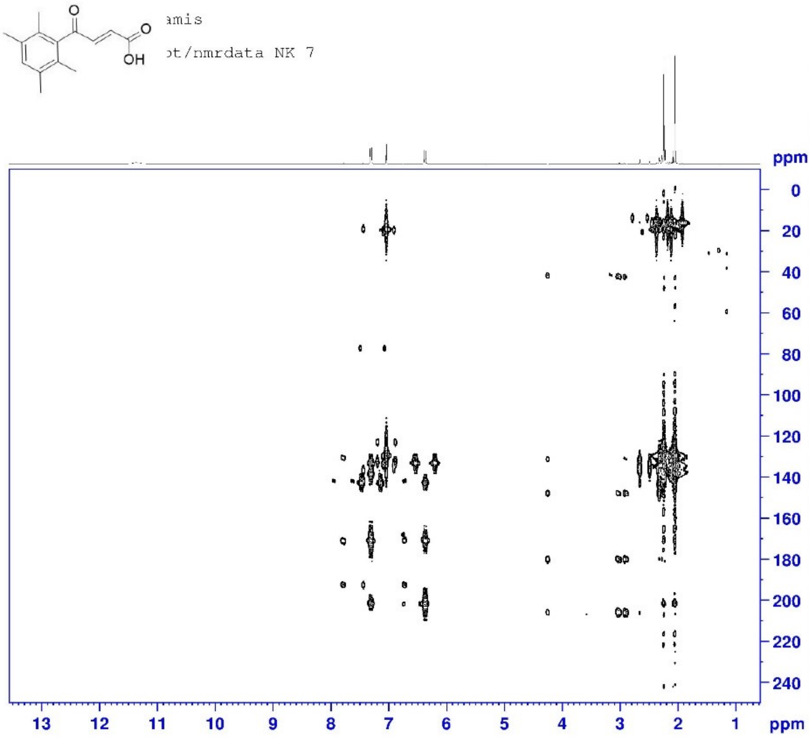

$^{13}C\{^1H\}$ NMR (126 MHz, $CDCl_3$) of (*E*)-4-oxo-4-(2,3,5,6tetramethylphenyl)but-2-enoic acid **31**.

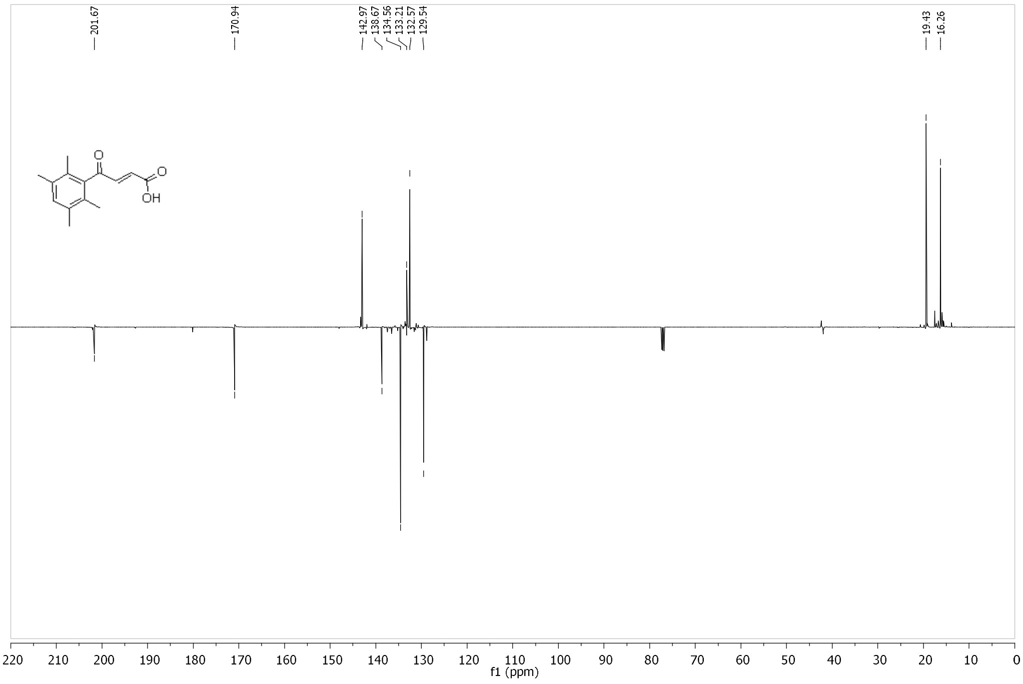

1-Phenyl-4-(2,3,5,6-tetramethylphenyl)butane-1,4-dione 27a.

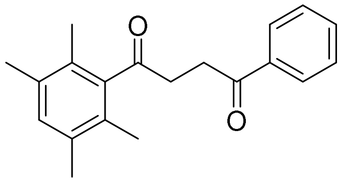

This compound is novel. DBU (105 mg, 0.68 mmol, 0.4 equiv) was added to the stirred solution of benzaldehyde (366 mg, 3.45 mmol, 2 equiv) and thiazolium salt (92.8 mg, 0.34 mmol, 0.2 equiv) in THF (6 mL). The resulting reaction mixture was stirred in room temperature for 10-15 minutes. After that (E)-4-oxo-4-(2,3,5,6-tetramethylphenyl)but-2-enoic acid **31** (400 mg, 1.72 mmol, 1.0 equiv) was added at 60 °C overnight and monitored by TLC. After completion of the reaction, the reaction system was cooled to room temperature and washed with saturated sodium bicarbonate (30 mL) and extracted with EtOAc (3 x 30 mL), the organic layer was dried over $MgSO_4$ and concentrated under reduced pressure. The product was isolated via flash chromatography on silica eluted with 0-6% EtOAc in petroleum ether to give 1-phenyl-4-(2,3,5,6-tetramethylphenyl)butane-1,4-dione **27a** as a white solid (50.4 mg, 0.17 mmol, 14%). TLC: Rf ca 0.1 (9:1 hexane: EtOAc), strong UV and weak $KMnO_4$; Mp: 158.7 °C; HRMS (ESI+) *m/z*: [M+Na]+ Calcd for $C_{20}H_{22}NaO_2$ 317.1512; Found 317.1501; 3.4 ppm error; ν_{max} 2995, 2968, 2878, 1697, 1681 cm^{-1}; ^{1}H NMR (500 MHz, $CDCl_3$): δ 8.08 (2H, d, *J* = 7.2, ArH), 7.52 (3H, t, *J* = 7.5, ArH), 7.00 (1H, s, ArH), 3.44 (2H, t, *J* = 6.2, CH_2), 3.22 (2H, t, *J* = 6.2, CH_2), 2.25 (6H, s, CH_3), 2.16 (6H, s, CH_3); $^{13}C\{^1H\}$ NMR (126 MHz, $CDCl_3$): δ 209.9 (C), 198.4 (C), 142.2 (C), 136.9 (C), 134.4 (C), 133.2 (CH), 131.7 (CH), 128.6 (CH), 128.2 (C), 128.1 (CH), 39.2 (CH_2), 31.7 (CH_2), 19.5 (CH_3), 15.9 (CH_3); *m/z* (ES-API+) 317.2 (M^+ + Na, 100%); Enantiomeric excess and conversion determined by HPLC analysis (Chiralpak IC, 30 cm x 6 mm column, hexane:iPrOH 90:10, 1.0 mL/min, T = 25°C) ketone 6.9 min, *R* and *S* isomer 17.4 min and 14.7 min.

^{1}H NMR (500 MHz, $CDCl_3$) of 1-phenyl-4-(2,3,5,6-tetramethylphenyl)butane-1,4-dione **27a**.

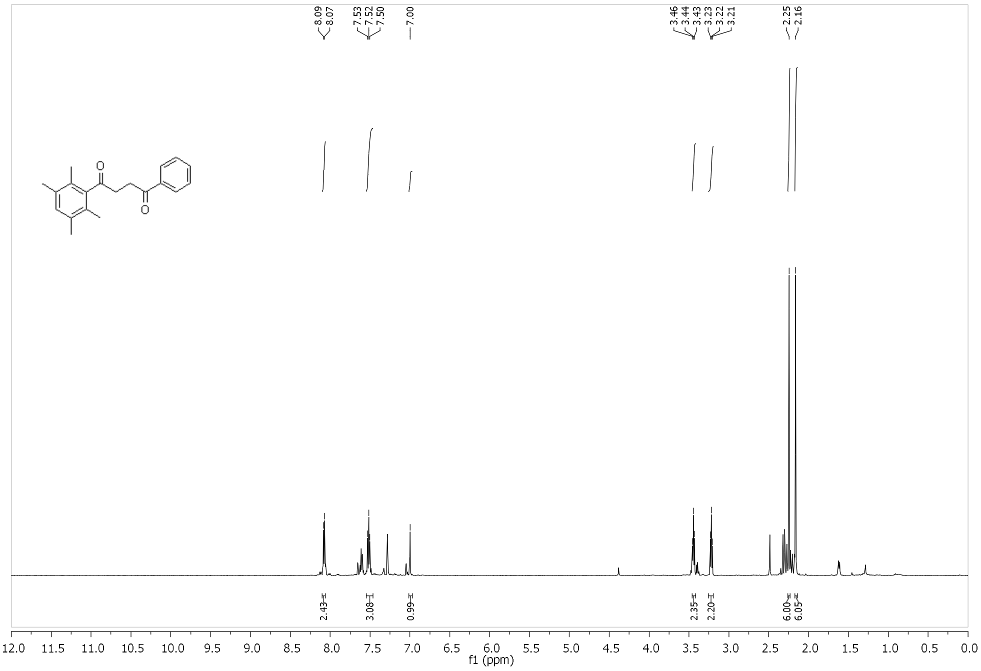

COSY (500 MHz, $CDCl_3$) of 1-phenyl-4-(2,3,5,6-tetramethylphenyl)butane-1,4-dione **27a**.

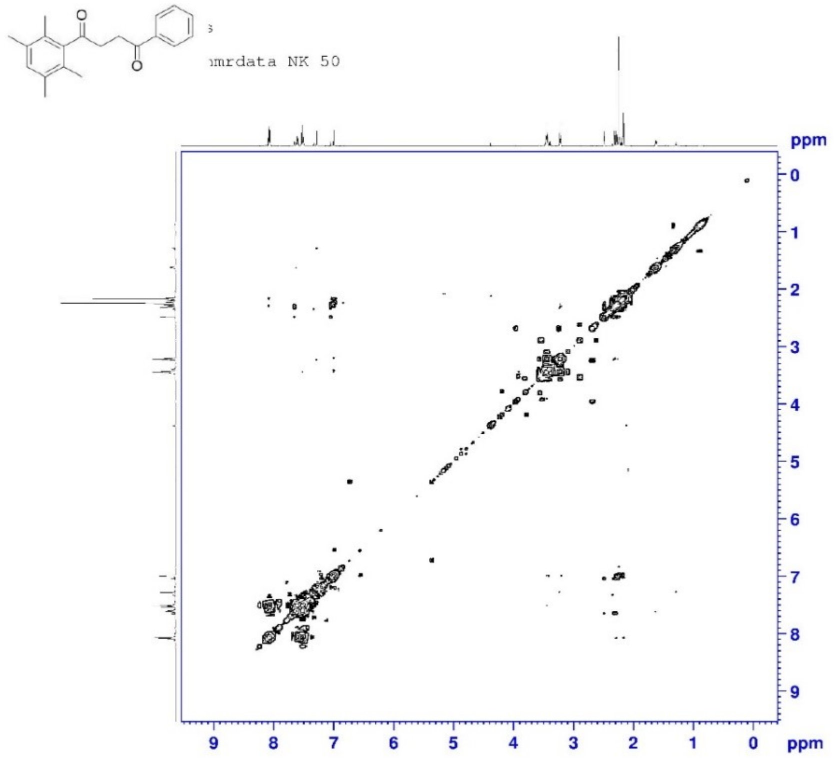

HSQC (126 MHz, $CDCl_3$) of 1-phenyl-4-(2,3,5,6-tetramethylphenyl)butane-1,4-dione **27a**.

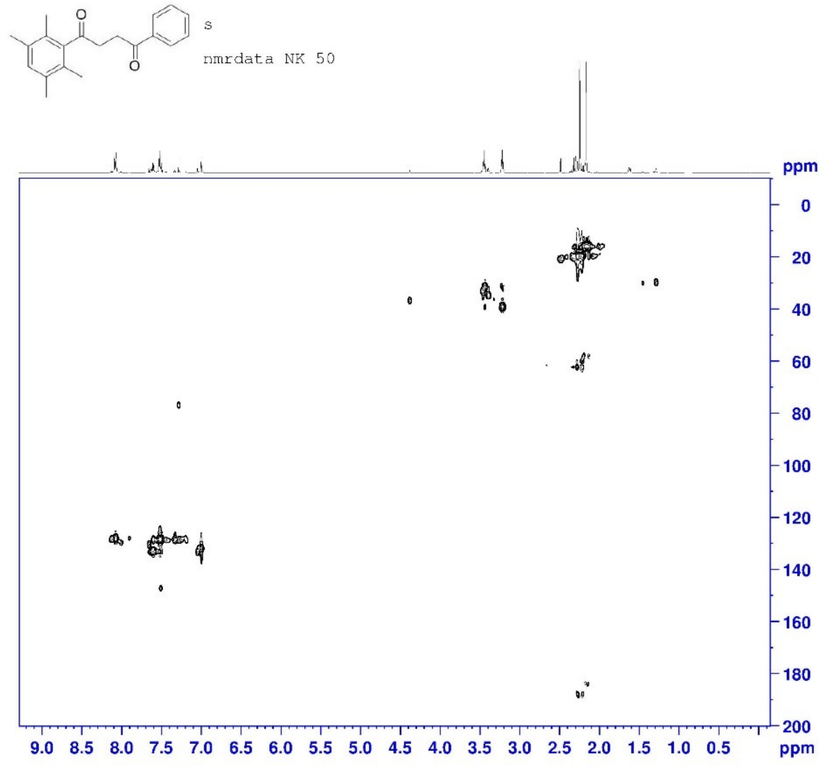

HMBC (126 MHz, $CDCl_3$) of 1-phenyl-4-(2,3,5,6-tetramethylphenyl)butane-1,4-dione **27a**.

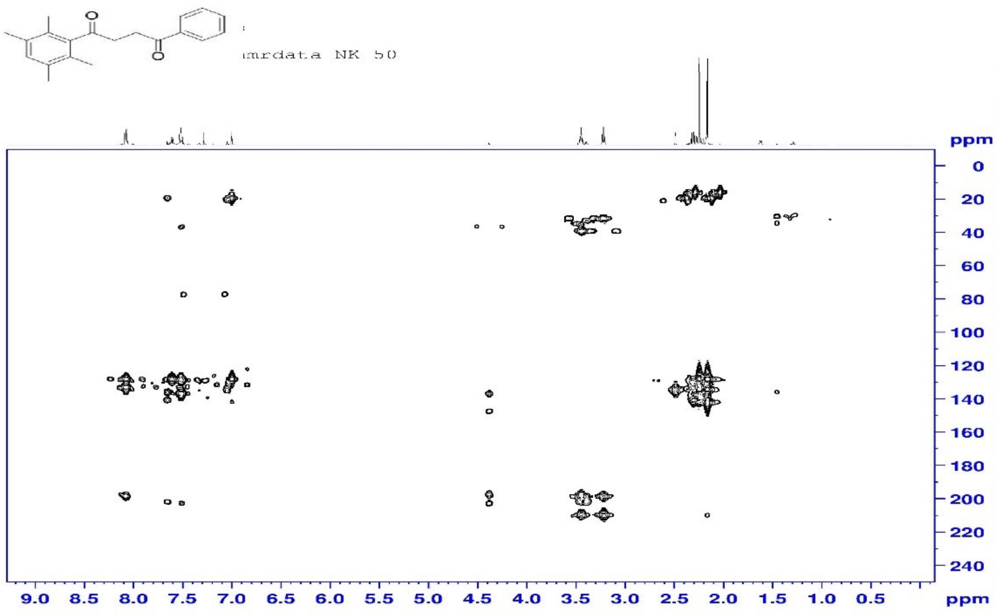

$^{13}C\{^{1}H\}$ NMR (126 MHz, $CDCl_3$) of 1-phenyl-4-(2,3,5,6-tetramethylphenyl)butane-1,4-dione **27a**.

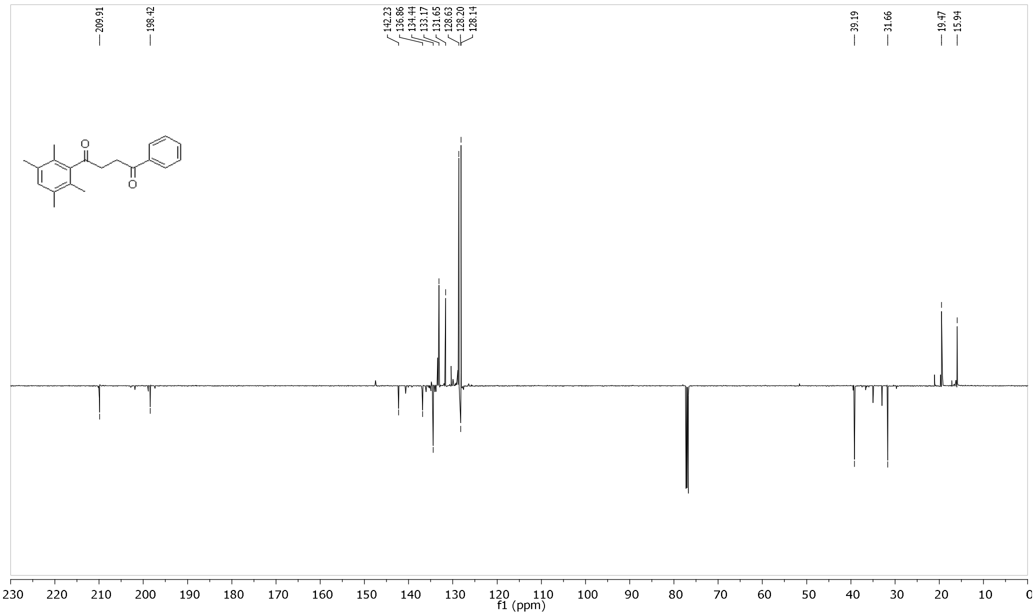

HPLC of 1-phenyl-4-(2,3,5,6-tetramethylphenyl)butane-1,4-dione **27a**.

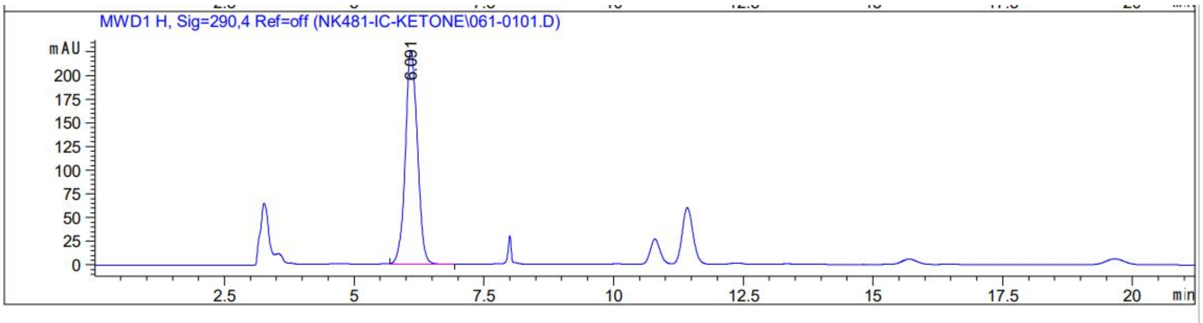

Signal 8: MWD1 H, Sig=290,4 Ref=off

Peak #	RetTime [min]	Type	Width [min]	Area [mAU*s]	Height [mAU]	Area %
1	6.091	VB	0.2560	3636.24707	225.63739	100.0000
Totals :				3636.24707	225.63739	

4-Hydroxy-4-phenyl-1-(2,3,5,6-tetramethylphenyl)butan-1-one 27b.

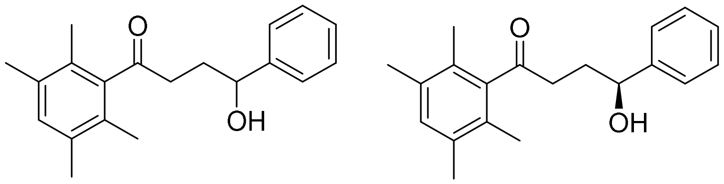

This compound is novel.

Synthesis of a racemic standard: (*R,R*)-3C-Tethered Ru(II)-TsDPEN catalyst (0.22 mg, 0.0004 mmol, 0.5 mol%) and (*S,S*)-3C-tethered Ru(II)-TsDPEN catalyst (0.22 mg, 0.0004 mmol, 0.5 mol%) were added to FA: TEA (5:2 azeotropic mixture, 0.04 mL) at rt and the mixture was stirred under a nitrogen atmosphere for 15 minutes, after which 1-phenyl-4-(2,3,5,6-tetramethylphenyl)butane-1,4- **27a** (20 mg, 0.07 mmol) was added. The reaction mixture was stirred under a nitrogen atmosphere and followed by TLC (5:1 hexane: EtOAc). After 48 h, the reaction was quenched using saturated $NaHCO_3$ solution (20 mL). EtOAc (20 mL) was added, and the organic layer was separated. The aqueous layer was extracted with EtOAc (3 x 20 mL) and the combined organic layers were dried ($MgSO_4$) and filtered. The solvent was removed to give the crude product. The product was isolated via flash chromatography on silica eluted with 0-50% EtOAc in petroleum ether to give 4-hydroxy-4-phenyl-1-(2,3,5,6-tetramethylphenyl)butan-1-one **27b** as a white solid (14.8 mg, 0.05 mmol, 73%). TLC: Rf ca 0.16 (4:1 hexane: EtOAc), strong UV and $KMnO_4$; Mp: 114.4°C; HRMS (ESI+) *m/z*: [M+Na]+ Calcd for $C_{20}H_{24}NaO_2$ 319.1669; Found 319.1658; 3.4 ppm error; ^{1}H NMR (500 MHz, $CDCl_3$): δ 7.42-7.38 (4H, m, ArH), 7.32-7.30 (1H, m, ArH), 6.97 (1H, s, ArH), 4.89 (1H, m, ArCH), 2.83 (2H, t, J = 6.9, CH_2), 2.44 (1H, s, OH), 2.21 (8H, s, CH_3 + CH_2), 2.05 (6H, s, CH_3); $^{13}C\{^1H\}$ NMR (126 MHz, $CDCl_3$): δ 212.3 (C), 144.2 (C), 142.6 (C), 134.4 (C), 131.6 (CH), 128.5 (CH), 127.9 (C), 127.6 (CH), 125.8 (CH), 73.5 (CH), 41.5 (CH_2), 32.2 (CH_2), 19.4 (CH_3), 15.9 (CH_3); *m/z* (ES-API+) 319.2 (M^+ + Na, 100%); Enantiomeric excess and conversion determined by HPLC analysis (Chiralpak IC, 30 cm x 6 mm column, hexane:iPrOH 90:10, 1.0 mL/min, T = 25°C) ketone 6.9 min, *R* and *S* isomer 17.4 min and 14.7 min.

(*S*)-4-Hydroxy-4-phenyl-1-(2,3,5,6-tetramethylphenyl)butan-1-one **27b**.

(*S,S*)-3C-tethered Ru(II)-TsDPEN catalyst (0.74 mg, 0.0012 mmol, 1 mol%) was added to FA: TEA (5:2 azeotropic mixture, 0.1 mL) at rt and the mixture was stirred under a nitrogen atmosphere for 10-15 minutes, after which 1-phenyl-4-(2,3,5,6-tetramethylphenyl)butane-1,4-dione **27a** (35 mg, 0.119 mmol) was added. The reaction mixture was stirred under a nitrogen atmosphere for 48 h. The reaction was followed by TLC (5:1 hexane: EtOAc). After 48 h, the reaction was quenched using saturated $NaHCO_3$ solution (20 mL). EtOAc (20 mL) was added and the organic layer was separated. The aqueous layer was extracted with EtOAc (3 x 20 mL) and the combined organic layers were dried ($MgSO_4$) and filtered. The solvent was removed to give the crude product. The product was isolated via flash chromatography on silica eluted with 0-50% EtOAc in petroleum ether to give (*S*)-4-hydroxy-4-phenyl-1-(2,3,5,6-tetramethylphenyl)butan-1-one **27a** as a white solid (21.4 mg, 0.07 mmol, 61%). The reaction was also followed by HPLC (Chiralpak IC, 30 cm x 6 mm column, hexane:iPrOH 90:10, 1.0 mL/min, T = 25°C); $[\alpha]_D^{18}$ – 16.7 (c 0.05 in $CHCl_3$); (after 48 h, 100% conversion, 94% ee (*S*)).

^1H NMR (500 MHz, $CDCl_3$) of 4-hydroxy-4-phenyl-1-(2,3,5,6-tetramethylphenyl)butan-1-one **27b**.

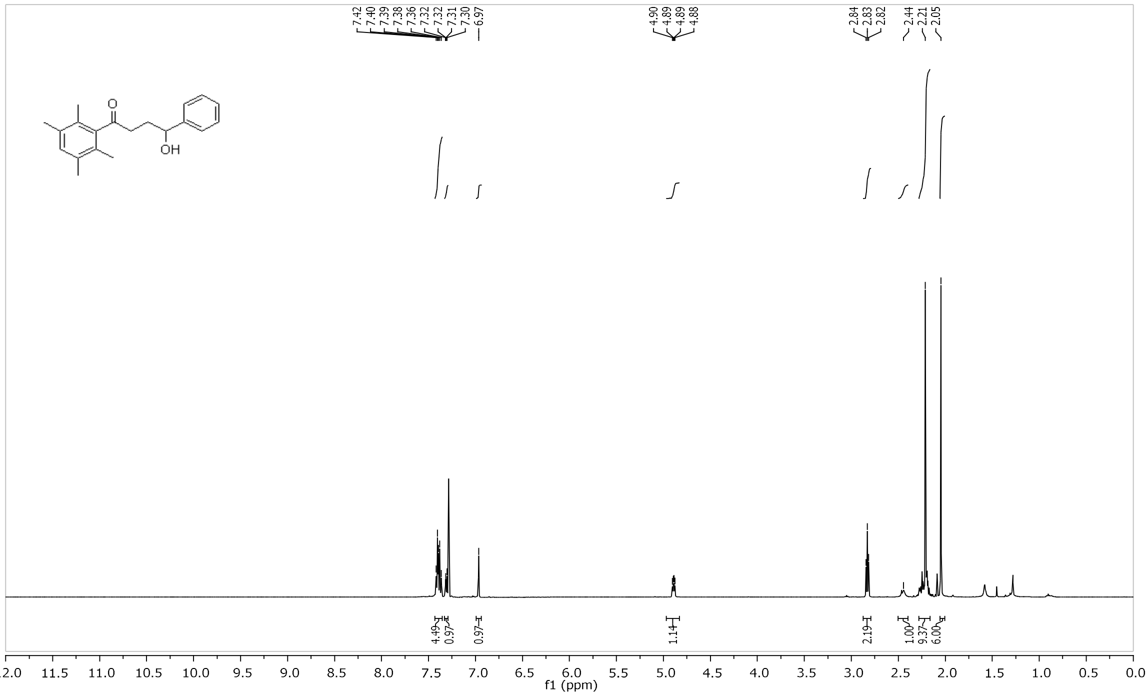

COSY (500 MHz, $CDCl_3$) of 4-hydroxy-4-phenyl-1-(2,3,5,6-tetramethylphenyl)butan-1-one **27b**.

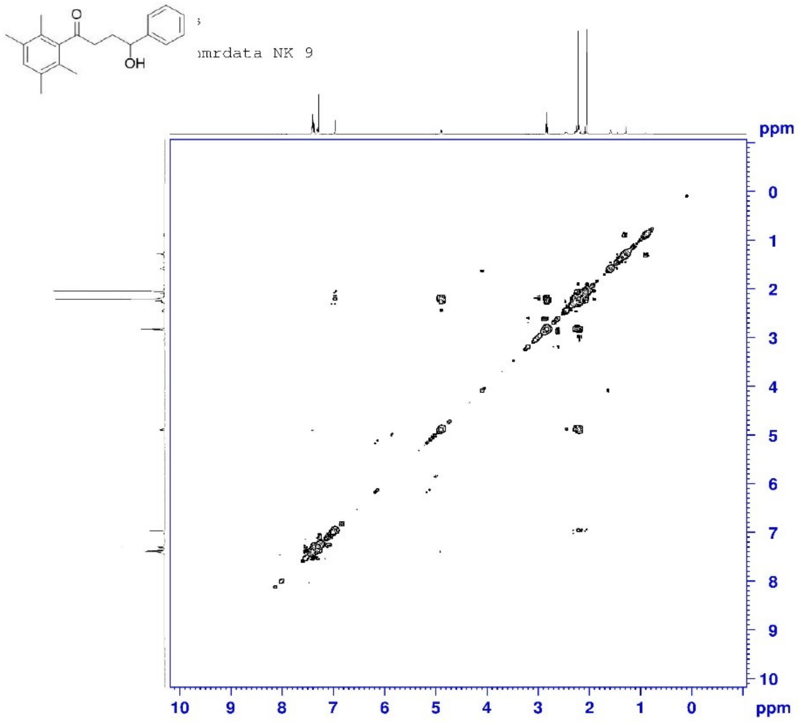

HSQC (126 MHz, $CDCl_3$) of 4-hydroxy-4-phenyl-1-(2,3,5,6-tetramethylphenyl)butan-1-one **27b**.

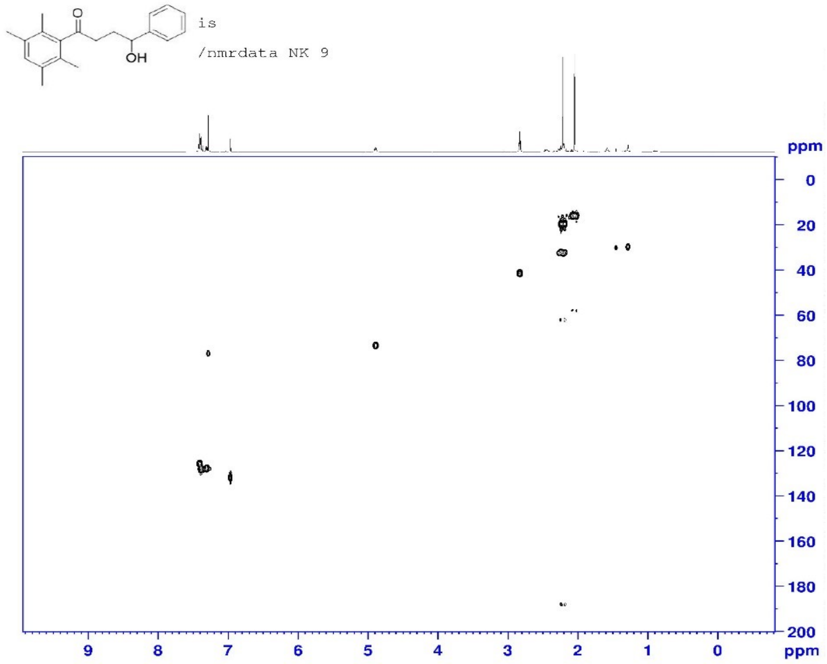

HMBC (126 MHz, $CDCl_3$) of 4-hydroxy-4-phenyl-1-(2,3,5,6-tetramethylphenyl)butan-1-one **27b**.

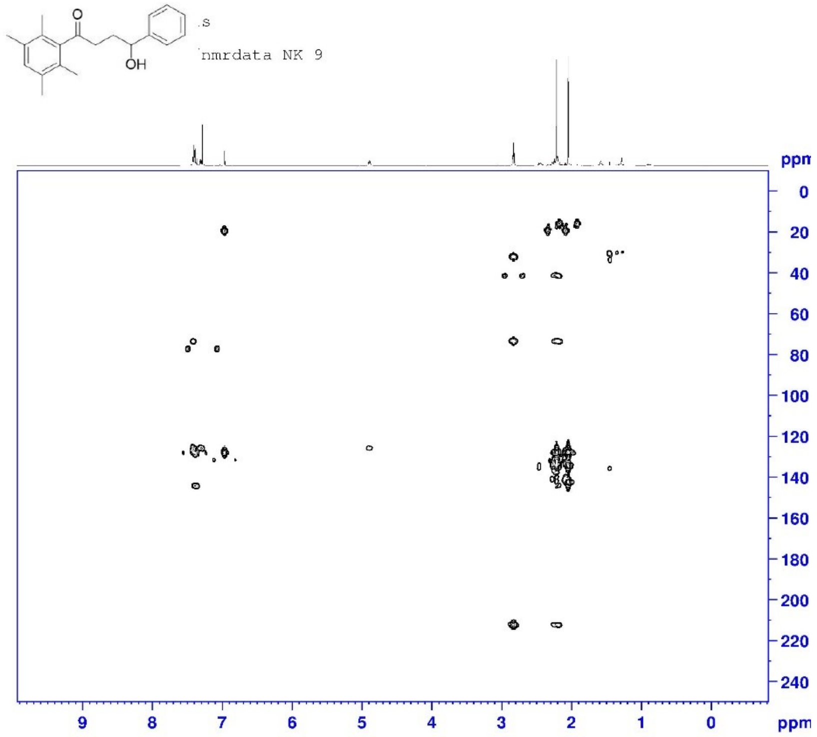

$^{13}C\{^1H\}$ NMR (126 MHz, $CDCl_3$) of 4-hydroxy-4-phenyl-1-(2,3,5,6-tetramethylphenyl)butan-1-one **27b**.

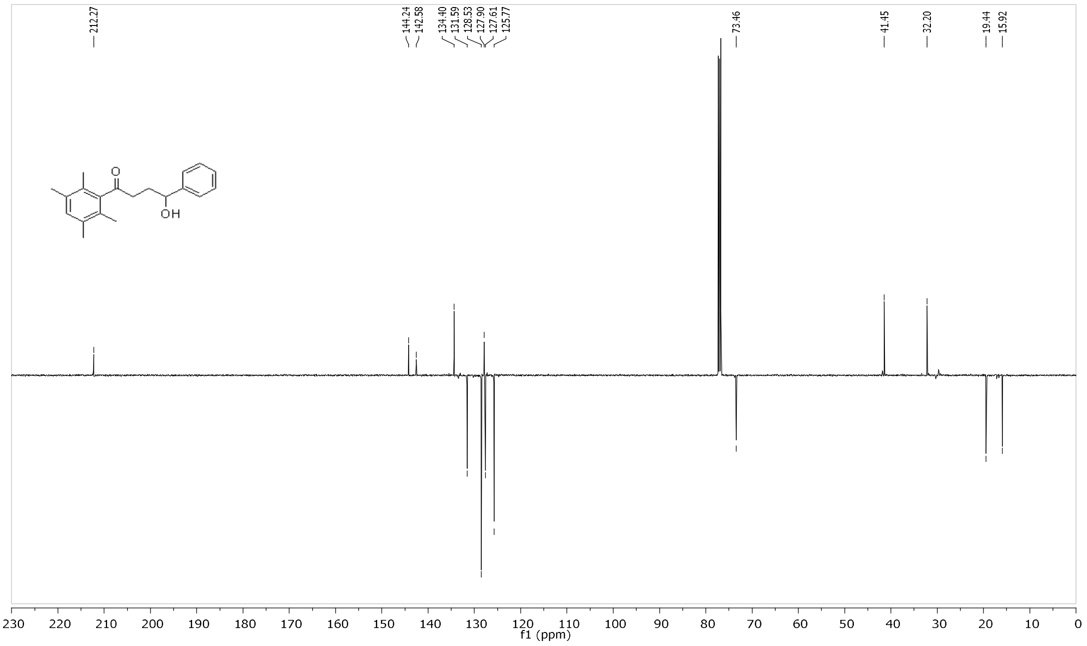

HPLC of racemic 4-hydroxy-4-phenyl-1-(2,3,5,6-tetramethylphenyl)butan-1-one **27b**.

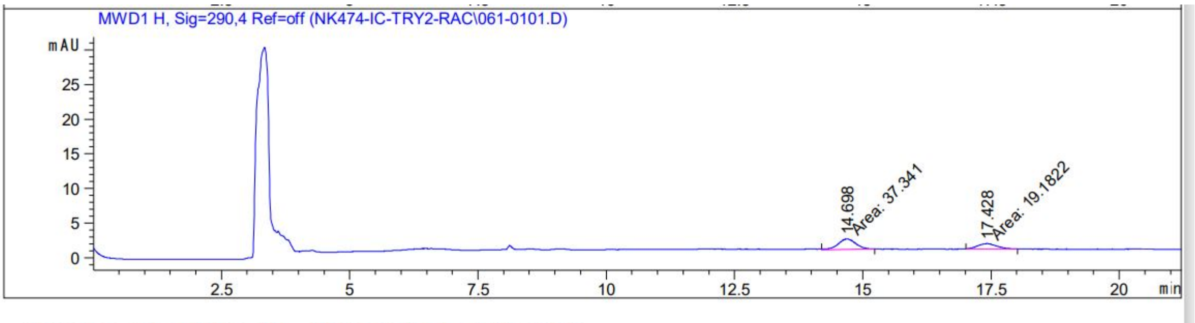

Signal 8: MWD1 H, Sig=290,4 Ref=off

Peak #	RetTime [min]	Type	Width [min]	Area [mAU*s]	Height [mAU]	Area %
1	14.698	MM T	0.4089	37.34096	1.52188	66.0631
2	17.428	MM T	0.4233	19.18221	7.55189e-1	33.9369
Totals :				56.52317	2.27707	

HPLC of (*R*)-4-Hydroxy-4-phenyl-1-(2,3,5,6-tetramethylphenyl)butan-1-one **27b**.

(*R,R*)-3C-tethered Ru(II)-TsDPEN catalyst (after 48 h, 100% conversion, 94% ee (*S*)).

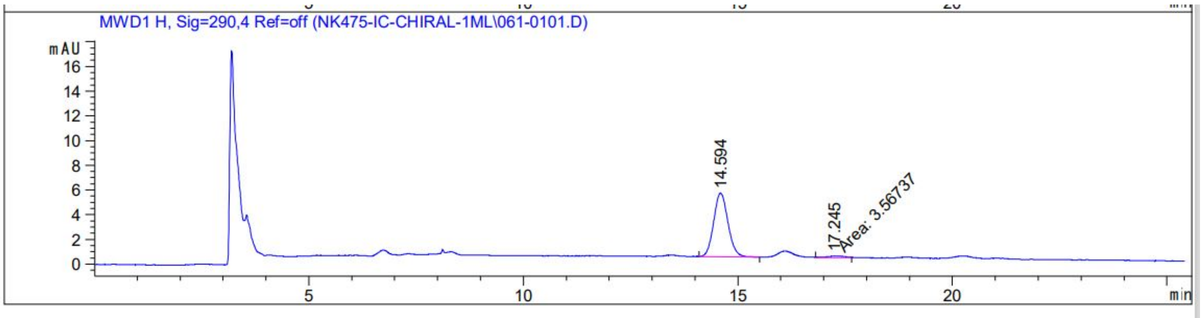

Signal 8: MWD1 H, Sig=290,4 Ref=off

Peak #	RetTime [min]	Type	Width [min]	Area [mAU*s]	Height [mAU]	Area %
1	14.594	BB	0.3428	119.54066	5.15011	97.1022
2	17.245	MM T	0.4884	3.56737	1.21747e-1	2.8978
Totals :				123.10803	5.27185	

4-Hydroxy-1-(4-hydroxy-2,3,5,6-tetramethylphenyl)-4-phenylbutan-1-one.

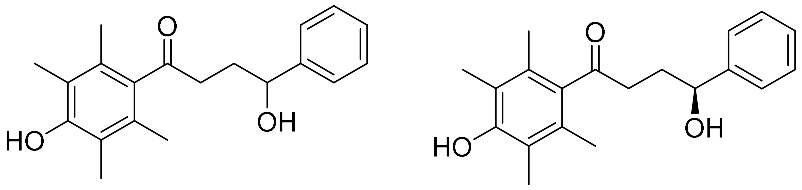

This compound is novel. **Synthesis of a racemic standard**: To a solution of 4-hydroxy-4-phenyl-1-(2,3,5,6-tetramethylphenyl)butan-1-one **27b** (101 mg, 0.34 mmol) was in HFIP (4.3 mL), phthaloyl peroxide (106 mg, 0.645 mol) was added at r.t and the solution warmed to 40 °C and left for 24 h. The solution was concentrated under vacuum at 23 °C, then 3.85:0.45 MeOH : sat.$NaHCO_3$ was added and warmed to 40 °C and left for 24 h after completion. The reaction mixture was then diluted with brine (20 mL) and DCM (20mL) and extracted using DCM (3 x 10mL) then dried using $MgSO_4$. Solvent was removed to give the crude product. The product was isolated via flash chromatography on silica eluted with 5-50% EtOAc in petroleum ether to give 4-hydroxy-1-(4-hydroxy-2,3,5,6-tetramethylphenyl)-4-phenylbutan-1-one as a yellow solid (55.3 mg, 0.177 mmol, 52%). TLC: Rf ca 0.6 (1:1 petroleum ether: EtOAc), weak UV and PMA active; Mp: 92.8 °C; HRMS (ESI+) *m/z*: [M+Na]+ Calcd for $C_{20}H_{24}NaO_3$ 335.1618; Found 335.1610; 2.3 ppm error; ν_{max} 3516, 2930, 1728, 1687 cm^{-1}; ^{1}H NMR (500 MHz, $CDCl_3$) δ 7.62 (1H, br.s, ArH), 7.37 (4H, m, ArH), 7.30 – 7.25 (1H, m, ArH), 4.91 – 4.79 (1H, m, CH), 2.78 (2H, t, *J* = 7.0, CH_2), 2.23 – 2.13 (3H, m, CH_2 + OH), 2.11 (6H, s, CH_3), 2.03 (6H, s, CH_3); $^{13}C\{^1H\}$ NMR (126 MHz, $CDCl_3$): δ 212.4 (C), 151.9 (C), 144.2 (C), 135.6 (C), 128.9 (C), 128.5 (CH), 127.6 (CH), 125.8 (CH), 119.8 (C), 73.5 (CH), 42.0 (CH_2), 32.4 (CH_2), 16.6 (CH_3), 11.7 (CH_3); *m/z* (ES-API+) 335.2 (M^+ + Na, 100%); Enantiomeric excess and conversion determined by HPLC analysis (Chiralpak IC, 30 cm x 6 mm column, hexane:iPrOH 90:10, 1.0 mL/min, T = 25 °C) *R* and *S* isomer 24.3 min and 21.8 min.

Synthesis of a chiral product:

To a solution of (*S*)-4-hydroxy-4-phenyl-1-(2,3,5,6-tetramethylphenyl)butan-1-one (134 mg, 0.45 mmol) in HFIP (4.6 mL), phthaloyl peroxide (112 mg, 0.68 mmol) was added at r.t and the solution warmed to 40 °C and left for 24 h, then concentrated under vacuum at 23 °C. A mixture of 4.1:0.48 MeOH : sat.$NaHCO_3$ were added and the reaction warmed to 40 °C and left for 24 h after completion and the reaction mixture was diluted with brine (20 mL) and DCM (20 mL) and extracted using DCM

(3 x10 mL) then dried using $MgSO_4$. Solvent was removed to give the crude product. The product was isolated via flash chromatography on silica eluted with 5-50% EtOAc in petroleum ether to give (*S*)-4-hydroxy-1-(4-hydroxy-2,3,5,6-tetramethylphenyl)-4-phenylbutan-1-one as a yellow solid (24.8 mg, 0.08 mmol, 18%). The reaction was also followed by HPLC (Chiralpak IC, 30 cm x 6 mm column, hexane:iPrOH 90:10, 1.0 mL/min, T = 25°C); $[\alpha]_D^{29}$ + 13.3 (c 0.01 in $CHCl_3$); (73% ee, (*S*)). Although the ee appears to have reduced, in the subsequent reaction the product ee is 99%. This may be due to an impurity under the minor peak in the HPLC for this compound, or an unintended enrichment in the ee of the subsequent product **38**.

^1H NMR (500 MHz, $CDCl_3$) of 4-hydroxy-1-(4-hydroxy-2,3,5,6-tetramethylphenyl)-4-phenylbutan-1-one.

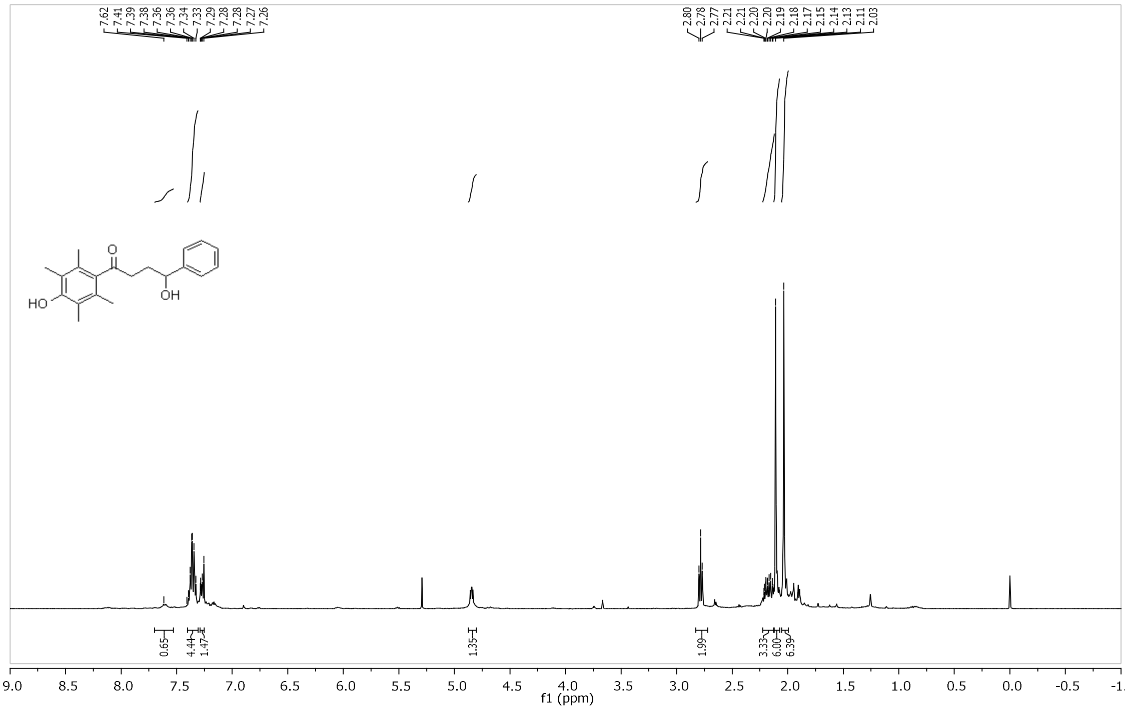

COSY (500 MHz, $CDCl_3$) of 4-hydroxy-1-(4-hydroxy-2,3,5,6-tetramethylphenyl)-4-phenylbutan-1-one.

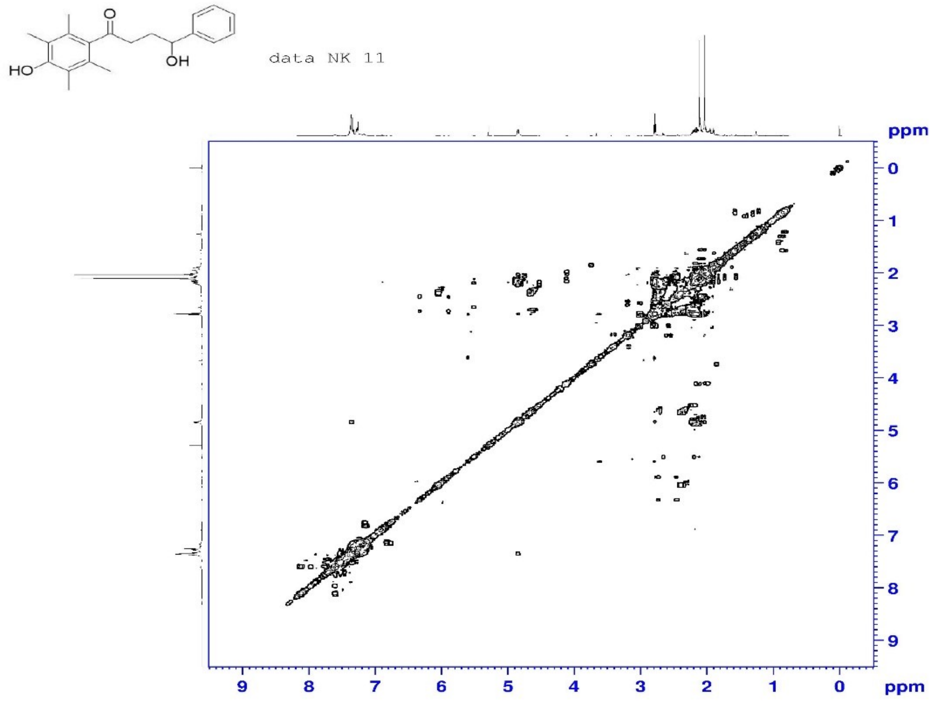

HSQC (126 MHz, $CDCl_3$) of 4-hydroxy-1-(4-hydroxy-2,3,5,6-tetramethylphenyl)-4-phenylbutan-1-one.

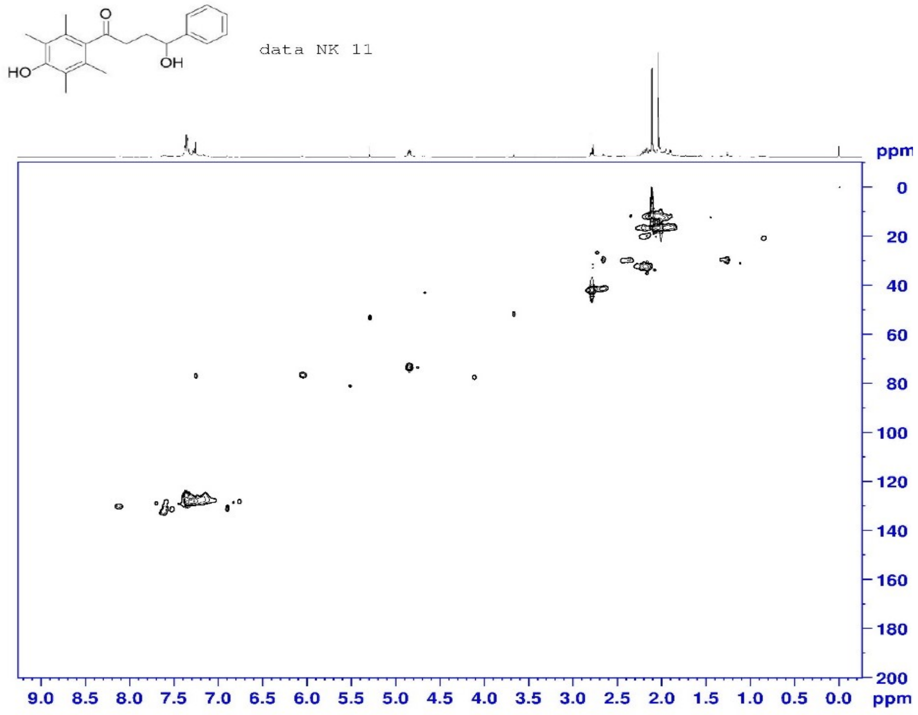

HMBC (126 MHz, $CDCl_3$) of 4-hydroxy-1-(4-hydroxy-2,3,5,6-tetramethylphenyl)-4-phenylbutan-1-one.

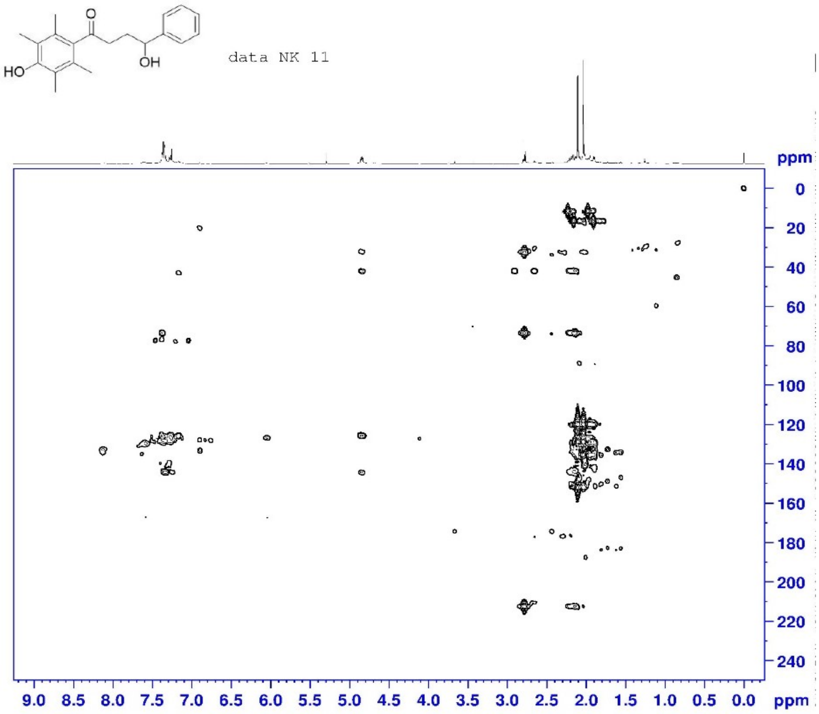

$^{13}C\{^{1}H\}$ NMR (126 MHz, $CDCl_3$) of 4-hydroxy-1-(4-hydroxy-2,3,5,6-tetramethylphenyl)-4-phenylbutan-1-one.

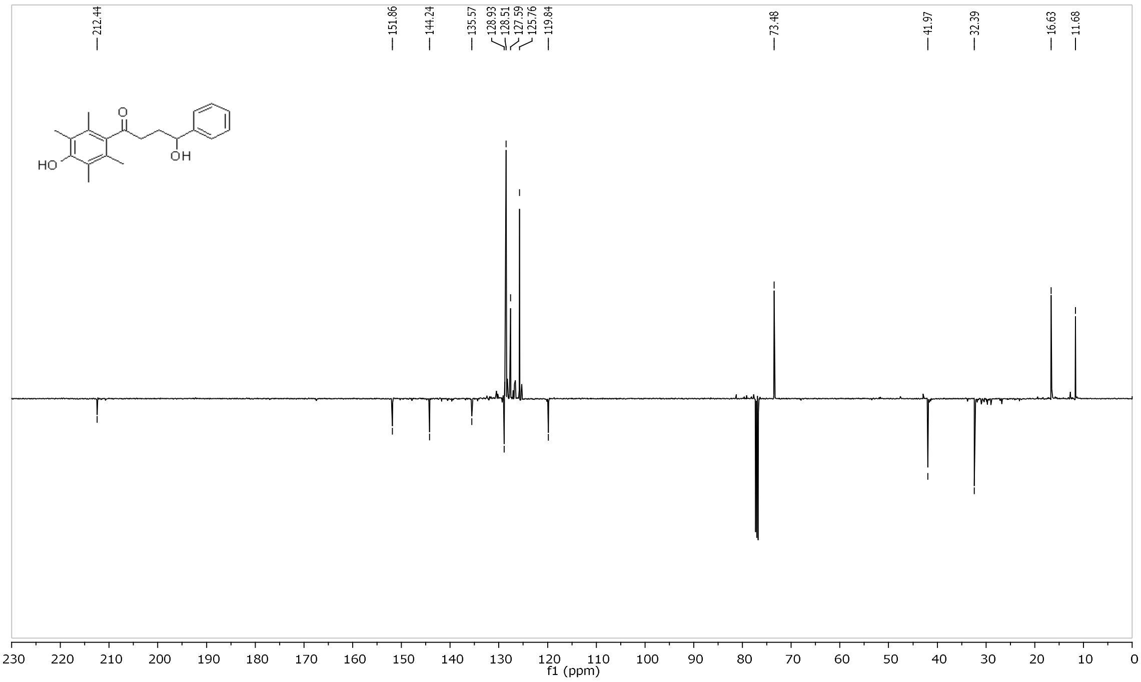

HPLC of racemic 4-hydroxy-1-(4-hydroxy-2,3,5,6-tetramethylphenyl)-4-phenylbutan-1-one.

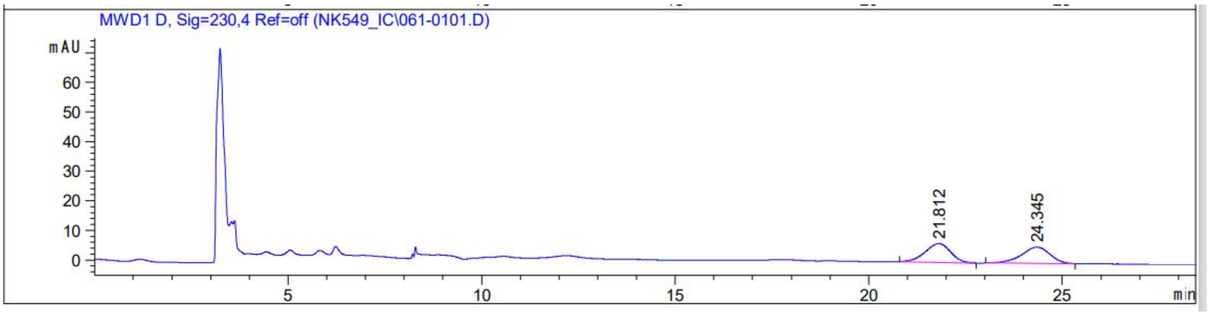

Signal 4: MWD1 D, Sig=230,4 Ref=off

Peak #	RetTime [min]	Type	Width [min]	Area [mAU*s]	Height [mAU]	Area %
1	21.812	BB	0.6263	288.03738	6.33645	50.7204
2	24.345	BB	0.6362	279.85468	5.45799	49.2796
Totals :				567.89206	11.79444	

HPLC of (*R*)- of 4-hydroxy-1-(4-hydroxy-2,3,5,6-tetramethylphenyl)-4-phenylbutan-1-one.

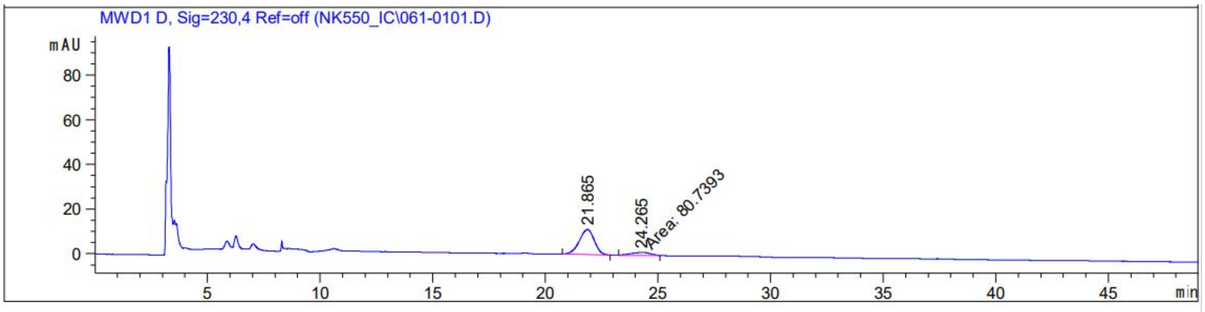

Signal 4: MWD1 D, Sig=230,4 Ref=off

Peak #	RetTime [min]	Type	Width [min]	Area [mAU*s]	Height [mAU]	Area %
1	21.865	BB	0.6947	521.64337	11.25506	86.5967
2	24.265	MM	1.0210	80.73929	1.31792	13.4033
Totals :				602.38266	12.57298	

5-Phenyldihydrofuran-2(3H)-one 38.

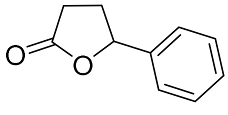

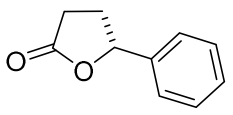

This compound is known and fully characterized. Brown, H. C.; Kulkarni, S. V.; Racheria, U. S. Chiral Synthesis via Organoboranes. 39. A Facile Synthesis of γ-Substituted-γ-butyrolactones in Exceptionally High Enantiomeric Purity. *J. Org. Chem.* **1994**, *59*, 365-369.

Synthesis of a racemic standard: To a solution of 4-hydroxy-1-(4-hydroxy-2,3,5,6-tetramethylphenyl)-4-phenylbutan-1-one (55.3 mg, 0.177 mmol) in MeOH (4.1 mL) was titrated a solution of CAN (411 mg, 0.75 mmol) in MeOH (1.8 mL) at RT in the open atmosphere until the color of the CAN solution persisted (orange color). The reaction mixture was diluted with H_2O (7 mL) and stirred for 5–10 minutes then further diluted with DCM (4 mL) and saturated with NaCl. The layers were separated, and the aqueous layer extracted with DCM (5 × 3 mL), and the combined organics dried (Na_2SO_4) and concentrated in vacuo. The product was isolated via flash chromatography on silica eluted with 10-50% EtOAc in petroleum ether to afford methyl 4-hydroxy-4-phenylbutanoate with 5-phenyldihydrofuran-2(3H)-one **38**. To fully convert the lactone, the purified mixture was diluted in DCM (0.5 mL) and one drop of TFA was added and left at r.t for 4 h, then $NaHCO_3$ (10 mL) was added and extracted with DCM (3 x 10 mL), washed with brine (10 mL) and the combined organics dried (Na_2SO_4) and concentrated in vacuo to give 5-phenyldihydrofuran-2(3H)-one **38** as a yellow oil (8 mg, 0.05 mmol, 28%) as a yellow oil; TLC: Rf ca 0.7 (9:1 DCM: EtOAc), weak UV and PMA active; ^{1}H NMR (500 MHz, $CDCl_3$): δ 7.43 – 7.37 (2H, m, ArH), 7.35 (3H, m, ArH), 5.53 (1H, m, CH), 2.71 – 2.61 (3H, m, CH_2), 2.28 – 2.14 (1H, m, CH_2); $^{13}C\{^1H\}$ NMR (126 MHz, $CDCl_3$): δ 176.9 (C), 139.4 (C), 128.8 (CH), 128.5 (CH), 125.3 (CH), 81.2 (CH), 31.0 (CH_2), 29.0 (CH_2); *m/z* (ES-API+) 185.0 (M^+ + Na, 100%); GC analysis (CP-CHIRALSIL-DEX-CB, 25 m x 0.25 mm x 0.25 μm, gas H_2, T = 140 °C, P = 18 psi, FID temp 250 °C, injector temp 220 °C, *R* isomer 10.49 min., *S* isomer 9.67 min.)

(*S*)-5-Phenyldihydrofuran-2(3H)-one **38.**

To a solution of (*S*)-4-hydroxy-1-(4-hydroxy-2,3,5,6-tetramethylphenyl)-4-phenylbutan-1-one (24 mg, 0.077 mmol) in MeOH (1.8 mL) was titrated a solution of CAN (411 mg, 0.75 mmol) in MeOH (1.8 mL) at RT in the open atmosphere until the color of the CAN solution persisted (orange color). The reaction mixture was diluted with H_2O (6 mL) and stirred for 5–10 minutes then further diluted with DCM (4 mL) and saturated with NaCl. The layers were separated, and the aqueous layer extracted with DCM (5 × 3 mL), and the combined organics dried (Na_2SO_4) and concentrated in vacuo The product was isolated via flash chromatography on silica eluted with 10-50% EtOAc in petroleum ether to afford methyl 4-hydroxy-4-phenylbutanoate with 5-phenyldihydrofuran-2(3H)-one **38**. To fully convert the lactone, the purified mixture was diluted in 0.5 mL DCM and one drop of TFA was added and left at r.t for 4 h, then $NaHCO_3$ (10 mL) was added and extracted with DCM (3 x 10 mL), washed with brine (10 mL) and the combined organics dried (Na_2SO_4) and concentrated in vacuo to give 5-phenyldihydrofuran-2(3H)-one **38** (9.3 mg, 0.06 mmol, 74%) as a yellow oil; $[\alpha]_D^{29}$ – 11.7 (c 0.186 in $CHCl_3$); (99% ee (*S*)).

^{1}H NMR (500 MHz, $CDCl_3$) of (*S*)-5-phenyldihydrofuran-2(3H)-one **38.**

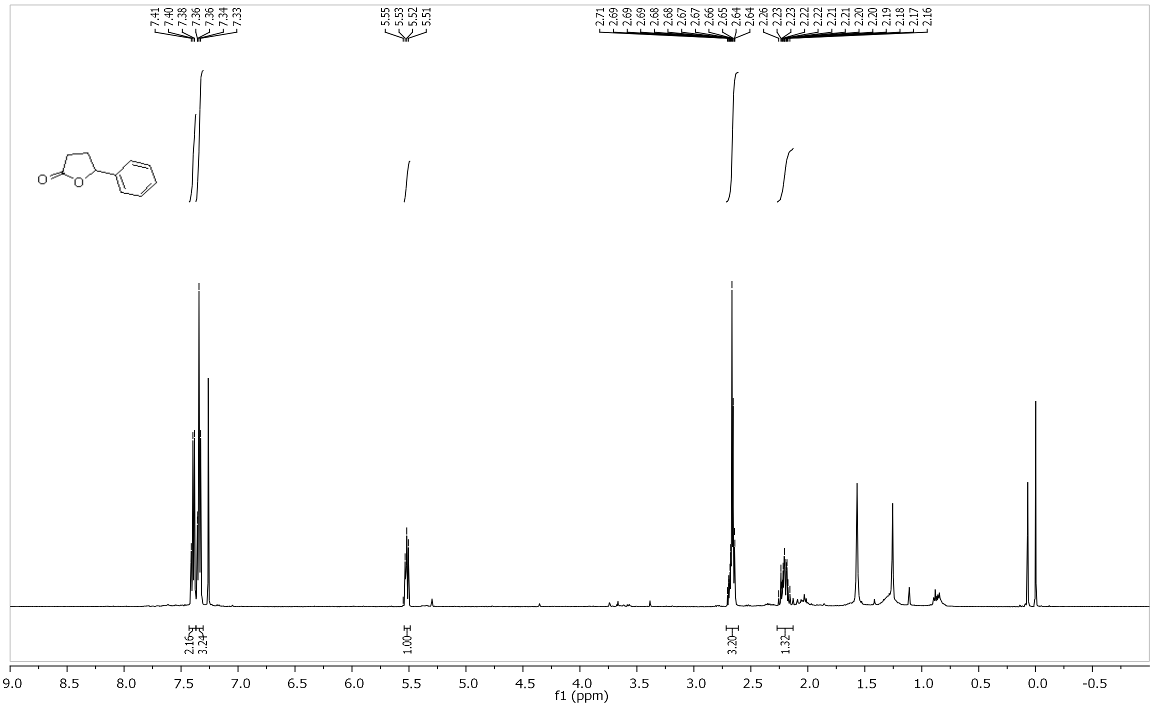

$^{13}C\{^1H\}$ NMR (126 MHz, $CDCl_3$) of (*S*)-5-phenyldihydrofuran-2(3H)-one **38.**

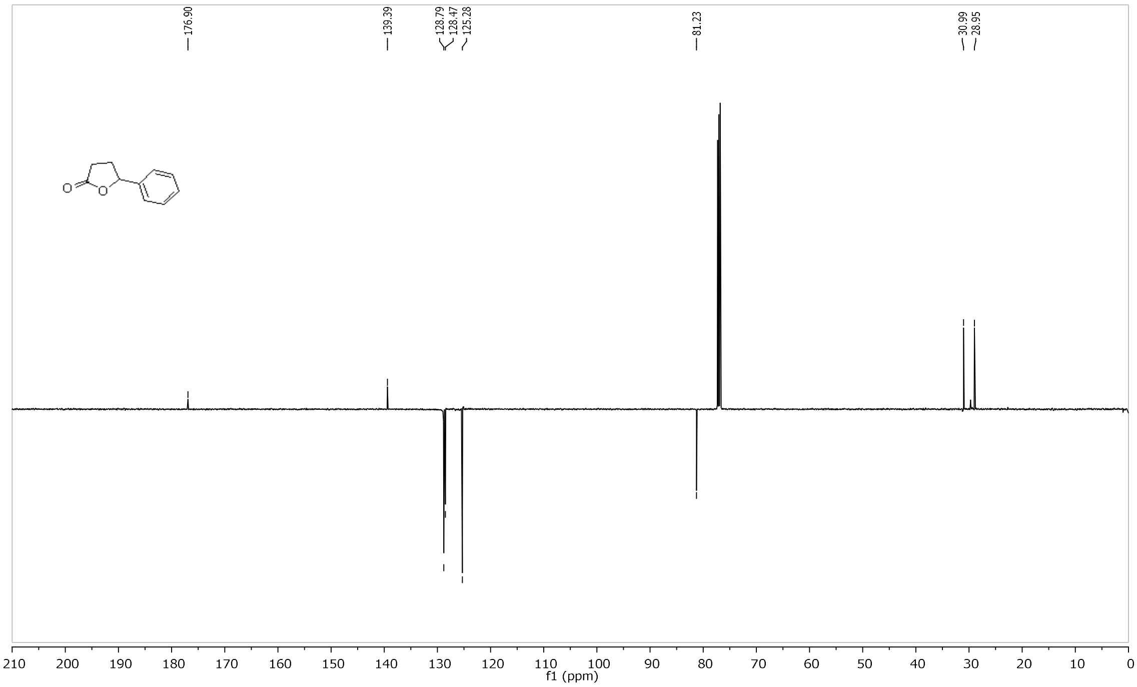

HPLC of Racemic (*S*)-5-phenyldihydrofuran-2(3H)-one **38.**

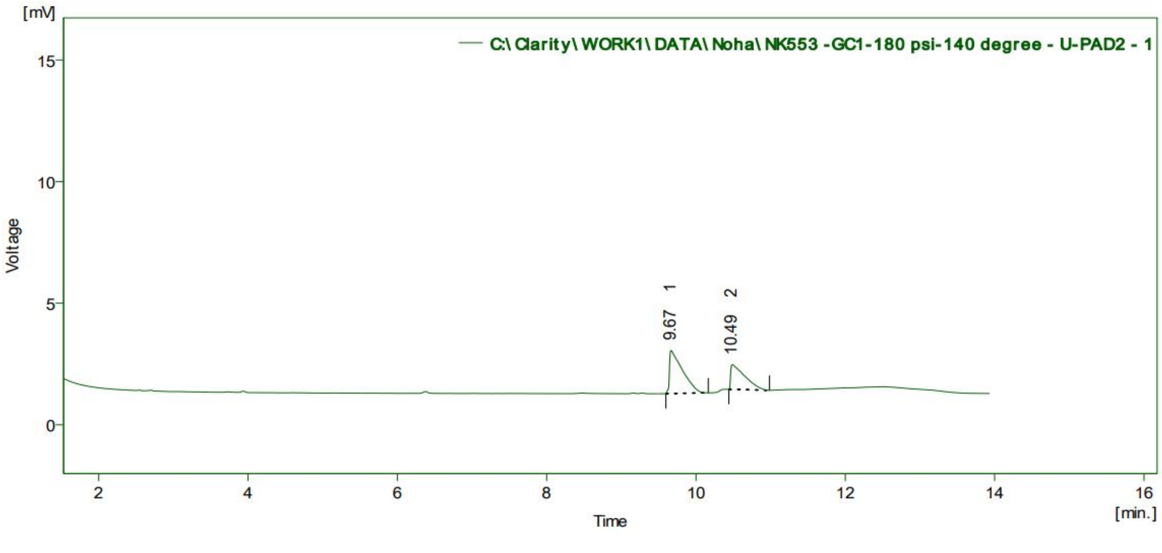

Result Table (Uncal -

	Reten. Time [min]	Start Time [min]	End Time [min]	Start Value [mV]	End Value [mV]	Area [mV.s]	Height [mV]	Area [%]	Height [%]	W05 [min]
1	9.668	9.596	10.164	2.570	2.630	42.794	3.528	61.4	63.4	0.19
2	10.488	10.440	10.984	2.916	2.839	26.937	2.036	38.6	36.6	0.21
	Total					69.731	5.563	100.0	100.0	

HPLC of ((*S*)-5-phenyldihydrofuran-2(3H)-one **38.**:

99% ee assigned as the minor isomer was not detected.

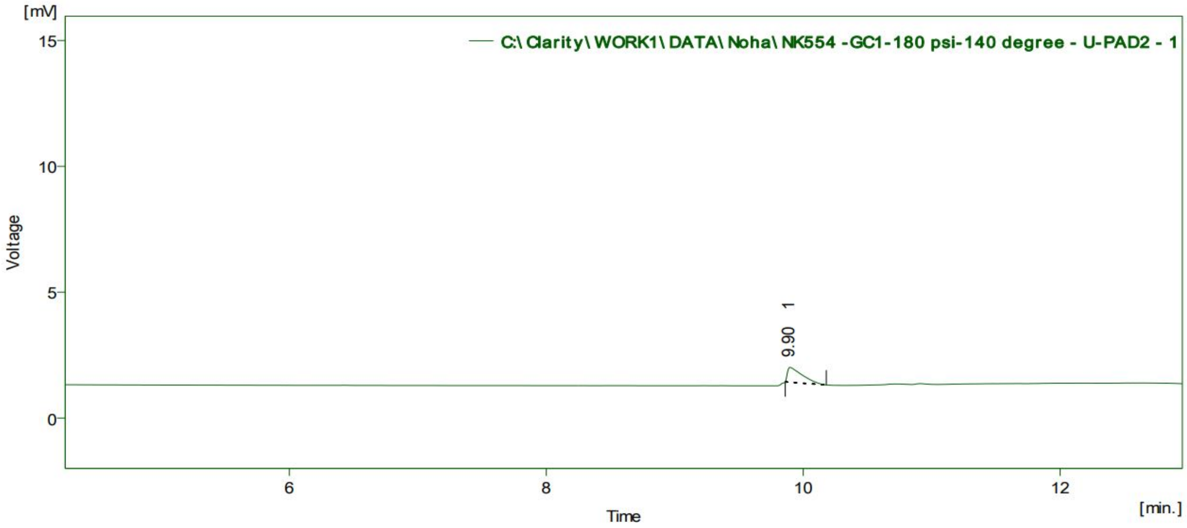

Result Table (Uncal -

	Reten. Time [min]	Start Time [min]	End Time [min]	Start Value [mV]	End Value [mV]	Area [mV.s]	Height [mV]	Area [%]	Height [%]	W05 [min]
1	9.896	9.860	10.180	2.868	2.631	9.600	1.178	100.0	100.0	0.12
	Total					9.600	1.178	100.0	100.0	

1-(4-Chlorophenyl)-4-(2,3,5,6-tetramethylphenyl)butane-1,4-dione 28a.

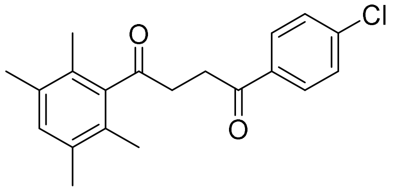

This compound is novel.

DBU (131 mg, 0.86 mmol, 0.4 equiv) was added to the stirred solution of 4-chloro benzaldehyde (454 mg, 3.23 mmol, 1.5 equiv) and thiazolium salt **32** (116 mg, 0.43 mmol, 0.2 equiv) in THF. The resulting reaction mixture was stirred in room temperature for 10-15 minutes. Then (E)-4-oxo-4-(2,3,5,6-tetramethylphenyl)but-2-enoic acid **31** (500 mg, 2.154 mmol, 1.0 equiv) was added at 60 °C and reacted for 2 days and monitored by TLC. After completion of the reaction, reaction system was cooled to room temperature and washed with saturated solution of sodium bicarbonate and extracted with EtOAc (3 x 30 mL), the organic layer was dried over sodium sulphate and concentrated under reduced pressure. The product was isolated via flash chromatography on silica eluted with 0-10% EtOAc in petroleum ether to give1-(4-chlorophenyl)-4-(2,3,5,6-tetramethylphenyl)butane-1,4-dione **28a** as a white solid (74.7 mg, 0.23 mmol, 11%). TLC: Rf ca 0.43 (5:2 hexane: EtOAc), strong UV and weak $KMnO_4$; Mp: 149.0 °C; HRMS (ESI+) *m/z*: [M+Na]+ Calcd for $C_{20}H_{21}ClNaO_2$ 351.1122; Found 351.1113; 2.7 ppm error; ν_{max} 2966, 2939, 2915, 2860 cm^{-1}; ^1H NMR (400 MHz, $CDCl_3$): δ 7.92 (2H, d, *J* = 7.7, ArH), 7.39 (2H, d, *J* = 7.3, ArH), 6.90 (1H, s, ArH), 3.30 (2H, t, *J* = 5.9, CH_2), 3.11 (2H, t, *J* = 6.0, CH_2), 2.14 (6H, s, CH_3), 2.05 (6H, s, CH_3); $^{13}C\{^1H\}$ NMR (126 MHz, $CDCl_3$): δ 209.8 (C), 197.2 (C), 142.1 (C), 139.6 (C), 135.2 (C), 134.5 (C), 131.7 (CH), 129.6 (CH), 129.0 (CH), 128.2 (C), 39.1 (CH_2), 31.6 (CH_2), 19.5 (CH_3), 15.9 (CH_3). *m/z* (ES-API+) 351.1 (M^+ + Na, 100%); Enantiomeric excess and conversion determined by HPLC analysis (Chiralpak IC, 30 cm x 6 mm column, hexane:iPrOH 90:10, 1.0 mL/min, T = 25 °C) ketone 8.2 min, *R* and *S* isomer 10.6 min and 9.96 min.

^{1}H NMR (500 MHz, $CDCl_3$) of 1-(4-chlorophenyl)-4-(2,3,5,6-tetramethylphenyl)butane-1,4-dione **28a**.

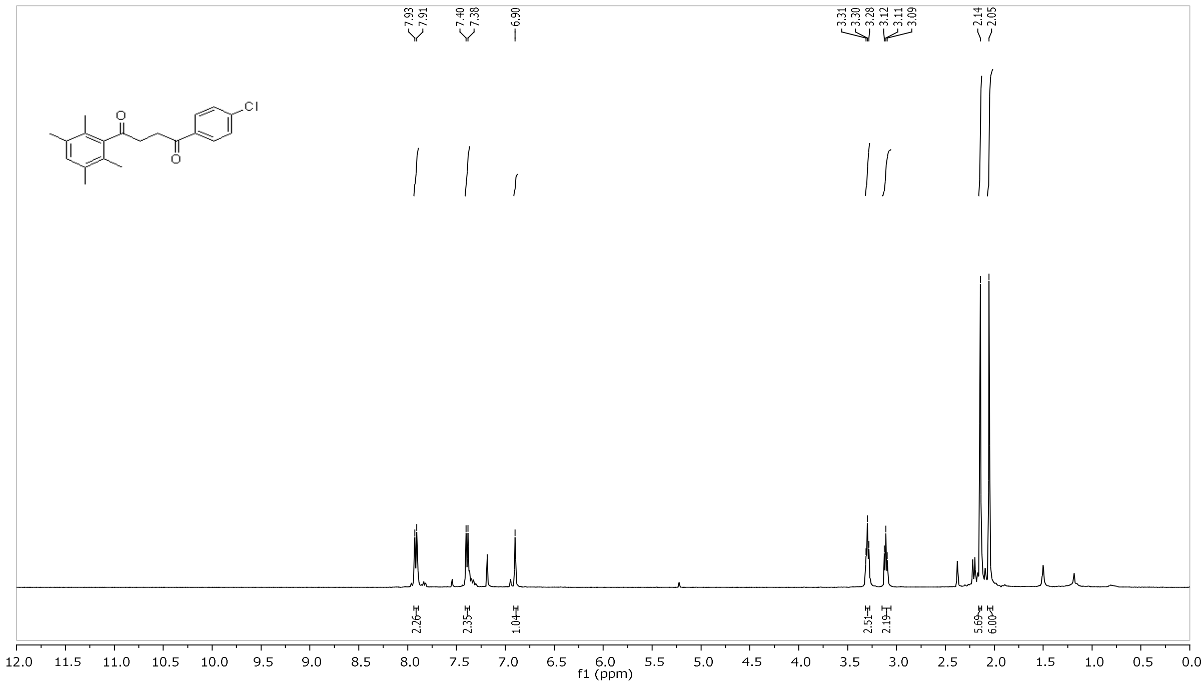

COSY (500 MHz, $CDCl_3$) of 1-(4-chlorophenyl)-4-(2,3,5,6-tetramethylphenyl)butane-1,4-dione **28a**.

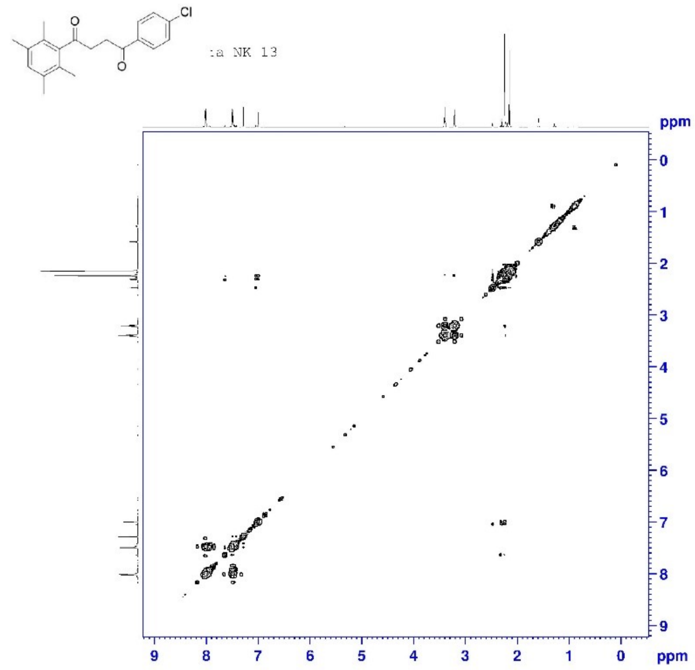

HMBC (126 MHz, $CDCl_3$) of 1-(4-chlorophenyl)-4-(2,3,5,6-tetramethylphenyl)butane-1,4-dione **28a**.

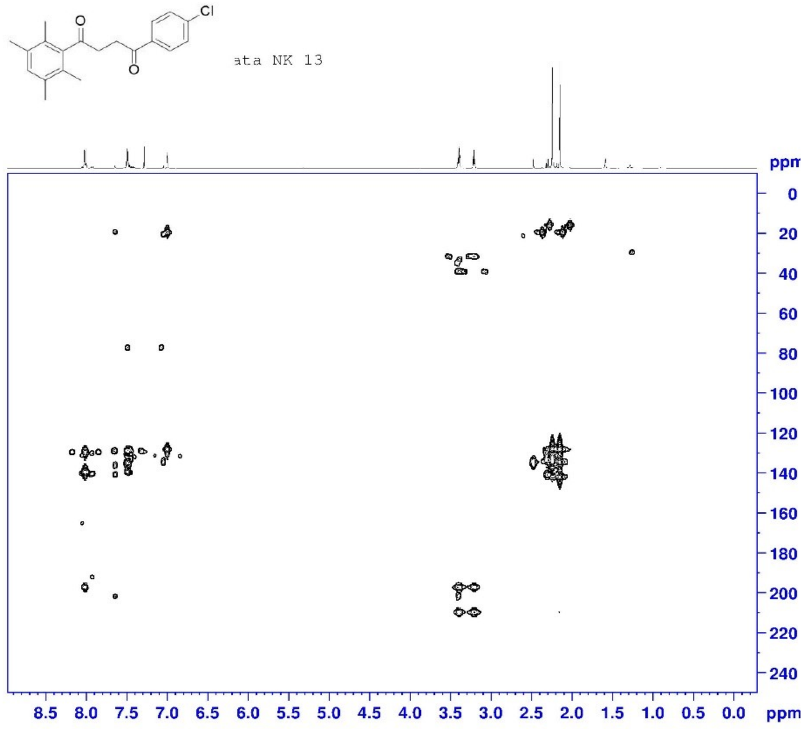

HSQC (126 MHz, $CDCl_3$) of 1-(4-chlorophenyl)-4-(2,3,5,6-tetramethylphenyl)butane-1,4-dione **28a**.

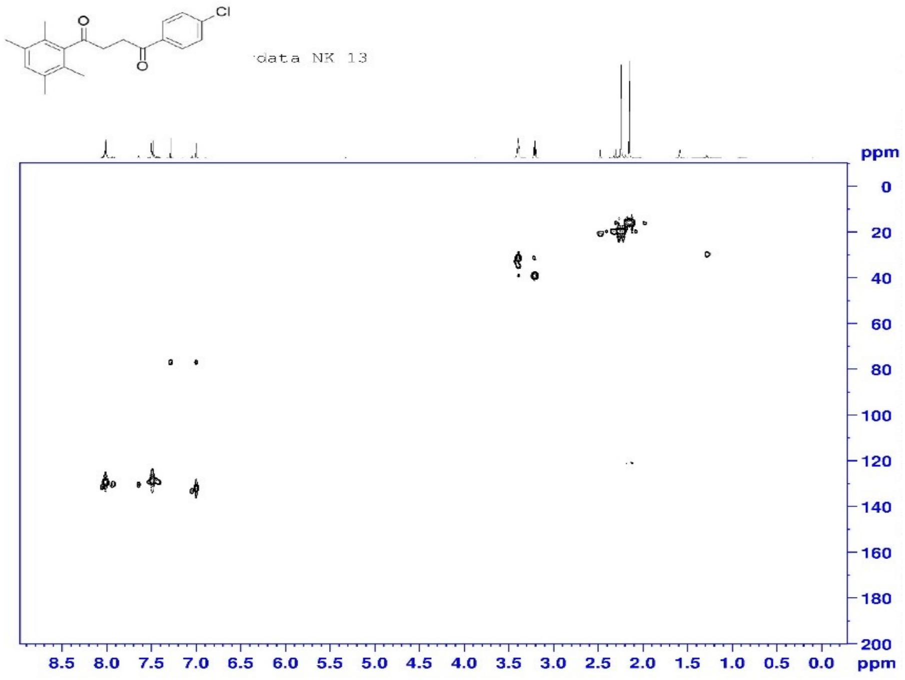

$^{13}C\{^{1}H\}$ NMR (126 MHz, $CDCl_3$) of 1-(4-chlorophenyl)-4-(2,3,5,6-tetramethylphenyl)butane-1,4-dione **28a**.

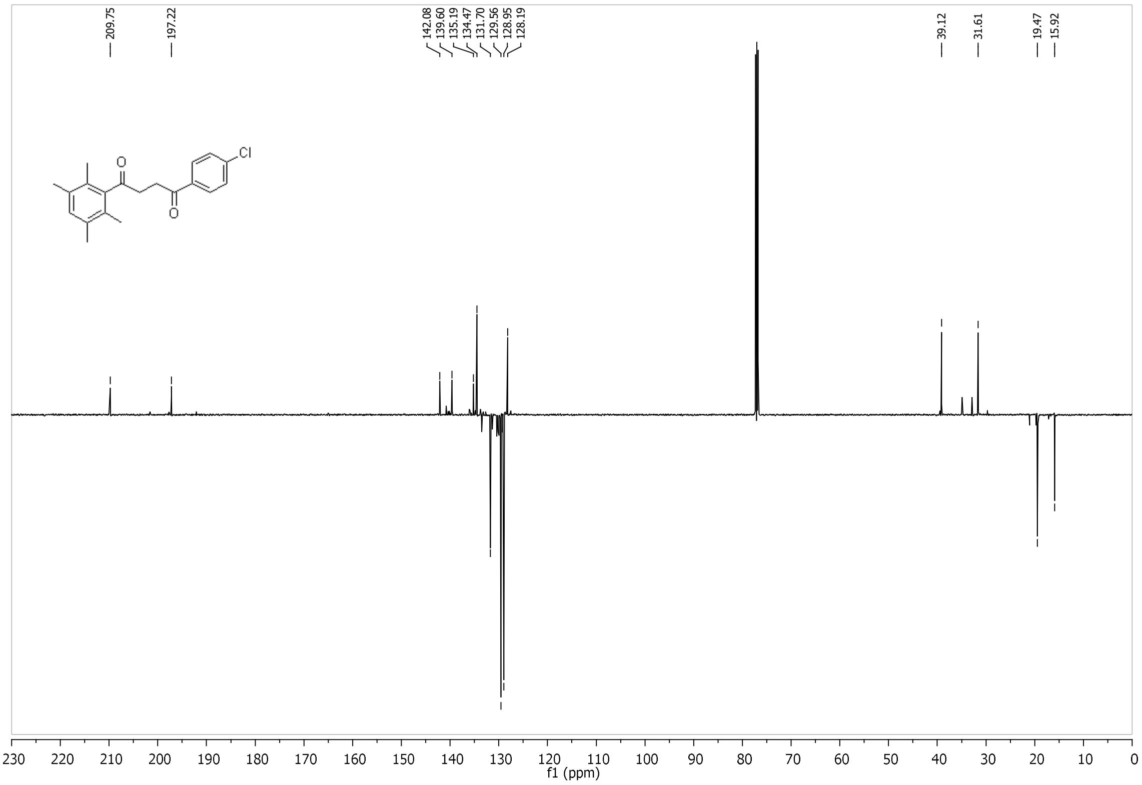

HPLC of 1-(4-chlorophenyl)-4-(2,3,5,6-tetramethylphenyl)butane-1,4-dione **28a**.

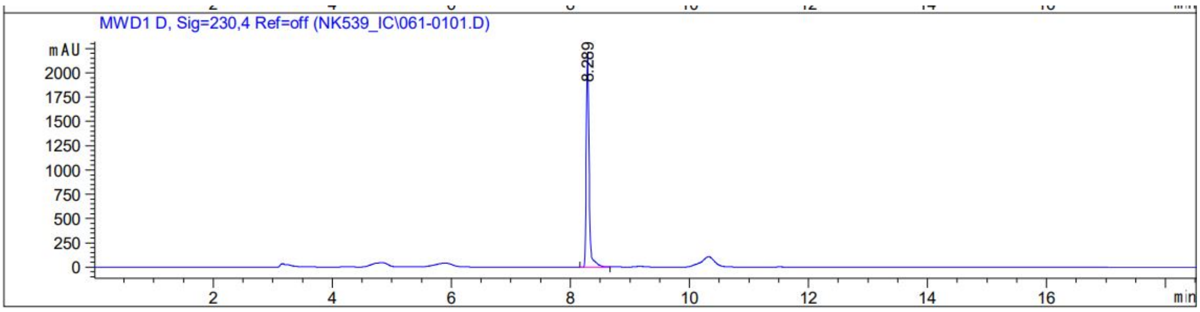

Signal 4: MWD1 D, Sig=230,4 Ref=off

Peak #	RetTime [min]	Type	Width [min]	Area [mAU*s]	Height [mAU]	Area %
1	8.289	BV	0.0519	7605.59424	2224.50269	100.0000
Totals :				7605.59424	2224.50269	

4-(4-Chlorophenyl)-4-hydroxy-1-(2,3,5,6-tetramethylphenyl)butan-1-one 28b.

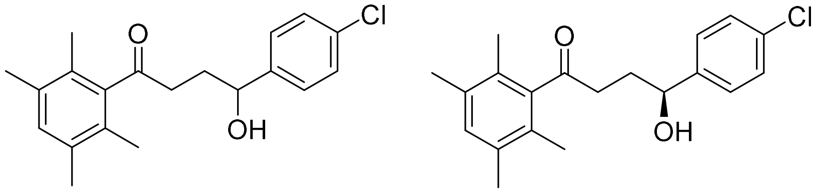

This compound is novel.

Synthesis of a racemic standard: (*R,R*)-3C-Tethered Ru(II)-TsDPEN catalyst (0.14 mg, 0.0002 mmol, 0.5 mol%) and (*S,S*)-3C-tethered Ru(II)-TsDPEN catalyst (0.14 mg, 0.0002 mmol, 0.5 mol%) were added to FA: TEA (5:2 azeotropic mixture, 0.02 mL) at rt and the mixture was stirred under a nitrogen atmosphere for 15 minutes; after which 1-(4-chlorophenyl)-4-(2,3,5,6-tetramethylphenyl)butane-1,4-dione **28a** (14.5 mg, 0.044 mmol) was added in DCM (0.02 mL). The reaction mixture was stirred under a nitrogen atmosphere and followed by TLC (5:1 hexane: EtOAc). After 48 h, the reaction was quenched using saturated $NaHCO_3$ solution (20 mL). EtOAc (20 mL) was added, and the organic layer was separated. The aqueous layer was extracted with EtOAc (3 x 20 mL) and the combined organic layers were dried ($MgSO_4$) and filtered. The solvent was removed to give the crude product. The product was isolated via flash chromatography on silica eluted with 0-50% EtOAc in petroleum ether to give 4-(4-chlorophenyl)-4-hydroxy-1-(2,3,5,6-tetramethylphenyl)butan-1-one **28b** as a white solid (12.8 mg, 0.038 mmol, 87%); ν_{max} 3353, 2953, 2923, 1594, 1491 cm^{-1}; TLC: Rf ca 0.2 (4:1 hexane: EtOAc), strong UV and PMA active; Mp: 106.2°C; HRMS (ESI+) *m/z*: [M+Na]+ Calcd for $C_{20}H_{23}ClNaO_2$ 353.1279; Found 353.1273; 1.7 ppm error; ^1H NMR (500 MHz, $CDCl_3$): δ 7.38 – 7.31 (4H, m, CH), 6.97 (1H, s, CH), 4.94 – 4.85 (1H, m, CH), 2.89 – 2.78 (2H, m, CH_2), 2.59 (1H, s, OH), 2.29 – 2.19 (8 H, m, CH_3 + CH_2), 2.05 (6H, s, CH_3); $^{13}C\{^1H\}$ NMR (126 MHz, $CDCl_3$): δ 212.3 (C), 142.8 (C), 142.4 (C), 134.5 (C), 133.7 (C), 131.7 (CH), 128.6 (CH), 127.9 (C), 127.1 (CH), 72.8 (CH), 41.3 (CH_2), 32.2 (CH_2), 19.4 (CH_3), 15.9 (CH_3); *m/z* (ES-API+) 353.1 (M^+ + Na, 100%); Enantiomeric excess and conversion determined by HPLC analysis (Chiralpak IC, 30 cm x 6 mm column, hexane:iPrOH 90:10, 1.0 mL/min, T = 25°C) ketone 8.2 min, *R* and *S* isomer 10.6 min and 9.96 min.

(*S*)-4-(4-Chlorophenyl)-4-hydroxy-1-(2,3,5,6-tetramethylphenyl)butan-1-one **28b.** (*S,S*)-3C-Tethered Ru(II)-TsDPEN catalyst (0.18 mg, 0.00030 mmol, 1 mol%) was added to FA: TEA (5:2 azeotropic mixture, 0.02 mL) at rt and the mixture was stirred under a nitrogen atmosphere for 10-15 minutes; after which 1-(4-chlorophenyl)-4-(2,3,5,6-tetramethylphenyl)butane-1,4-dione (10 mg, 0.030 mmol) was added in DCM (0.1 mL). The reaction mixture was stirred under a nitrogen atmosphere for 48 h. The reaction was followed by TLC (9:1 hexane: EtOAc). After 48 h, the reaction was quenched using saturated $NaHCO_3$ solution (20 mL). EtOAc (20 mL) was added, and the organic layer was separated. The aqueous layer was extracted with EtOAc (3 x 20 mL) and the combined organic layers were dried ($MgSO_4$) and filtered. The solvent was removed to give the crude product. The product was isolated via flash chromatography on silica eluted with 0-50% EtOAc in petroleum ether to (*S*)-4-(4-chlorophenyl)-4-hydroxy-1-(2,3,5,6-tetramethylphenyl)butan-1-one give as a white solid (4 mg, 0.012 mmol, 39%). The reaction was also followed by HPLC (Chiralpak IC, 30 cm x 6 mm column, hexane:iPrOH 90:10, 1.0 mL/min, T = 25 °C); $[\alpha]_D^{29}$ – 5.8 (c 0.08 in $CHCl_3$); (after 48 h, 100% conversion, 85% ee (*S*)).

^{1}H NMR (500 MHz, $CDCl_3$) of 4-(4-chlorophenyl)-4-hydroxy-1-(2,3,5,6-tetramethylphenyl)butan-1-one **28b.**

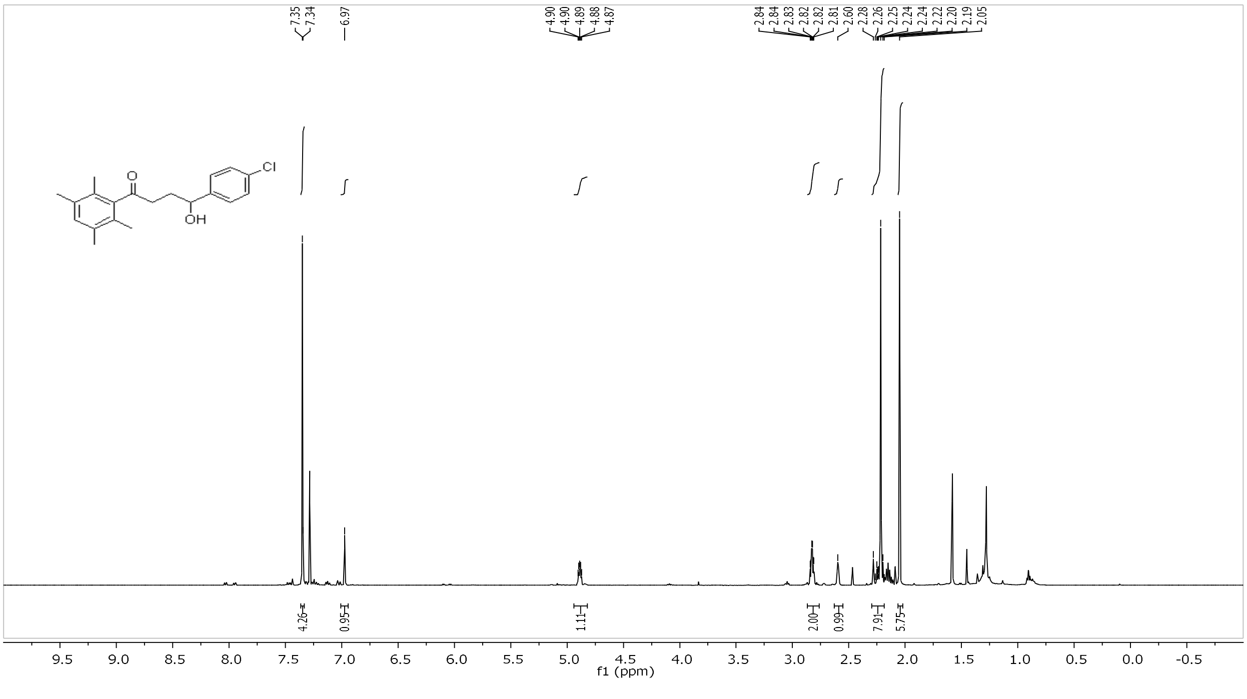

COSY (500 MHz, $CDCl_3$) of 4-(4-chlorophenyl)-4-hydroxy-1-(2,3,5,6-tetramethylphenyl)butan-1-one **28b.**

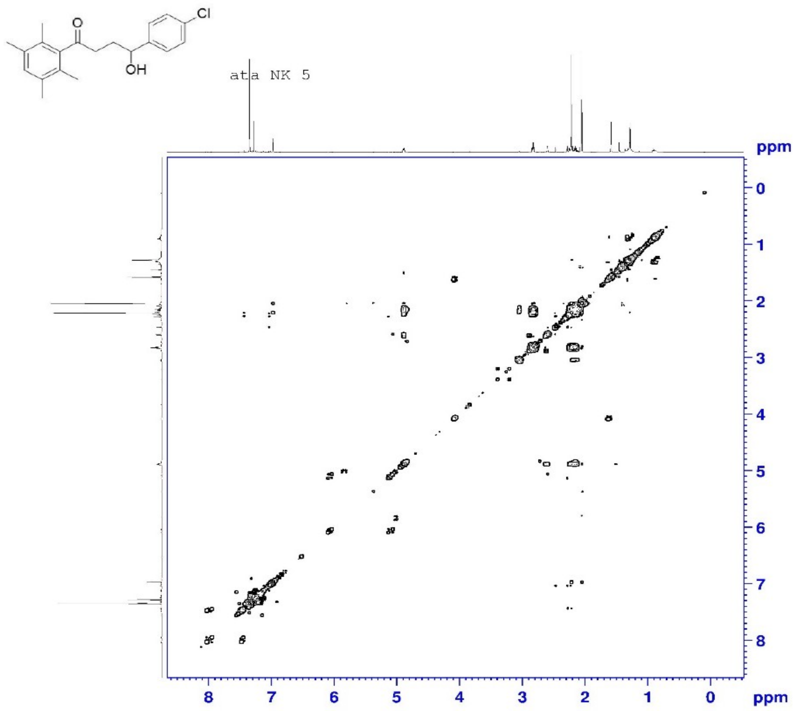

HSQC (126 MHz, $CDCl_3$) of 4-(4-chlorophenyl)-4-hydroxy-1-(2,3,5,6-tetramethylphenyl)butan-1-one **28b.**

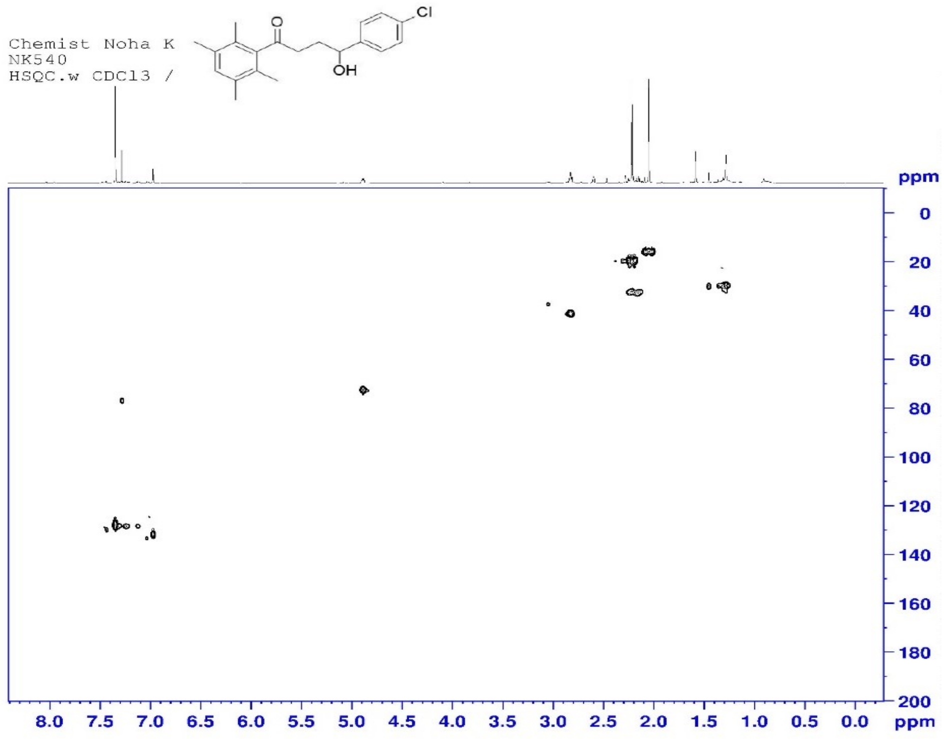

HMBC (126 MHz, $CDCl_3$) of 4-(4-chlorophenyl)-4-hydroxy-1-(2,3,5,6-tetramethylphenyl)butan-1-one **28b.**

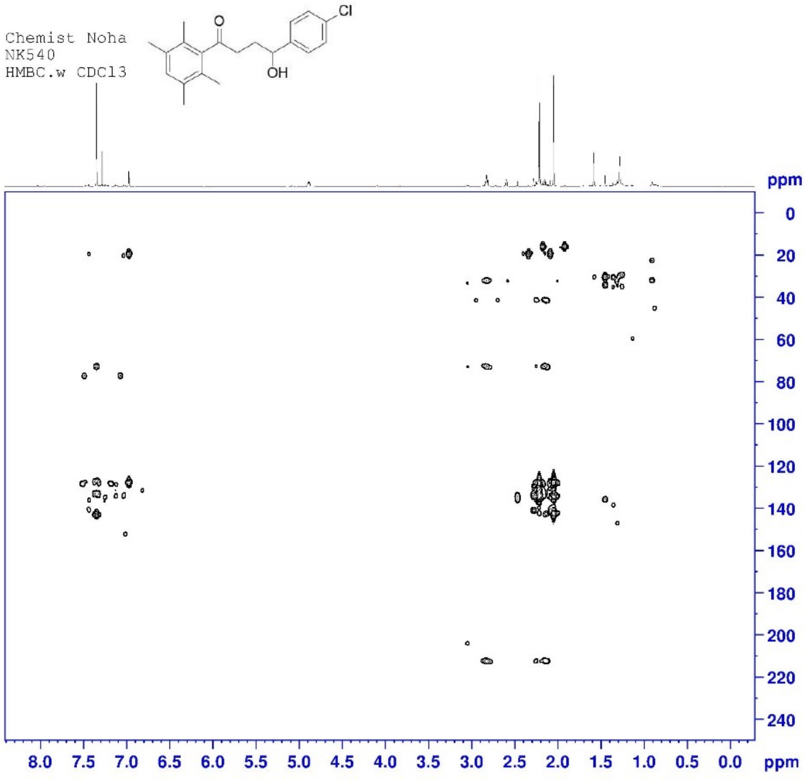

$^{13}C\{^1H\}$ NMR (126 MHz, $CDCl_3$) of 4-(4-chlorophenyl)-4-hydroxy-1-(2,3,5,6-tetramethylphenyl)butan-1-one **28b.**

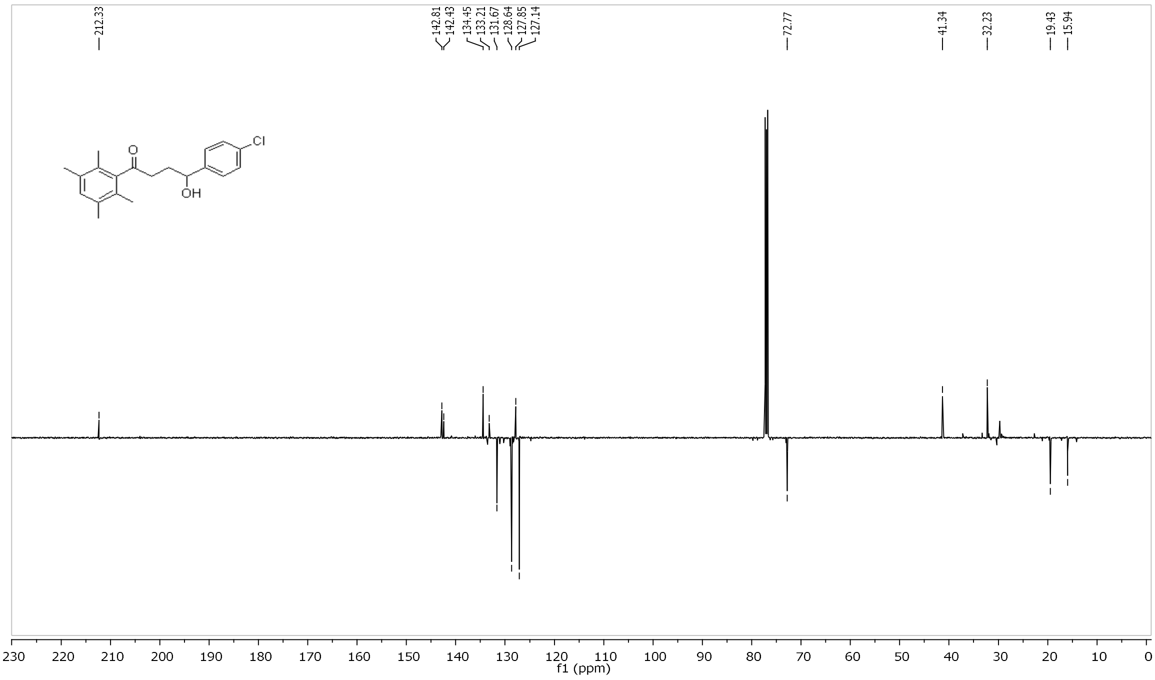

HPLC of racemic of 4-(4-chlorophenyl)-4-hydroxy-1-(2,3,5,6-tetramethylphenyl)butan-1-one **28b.**

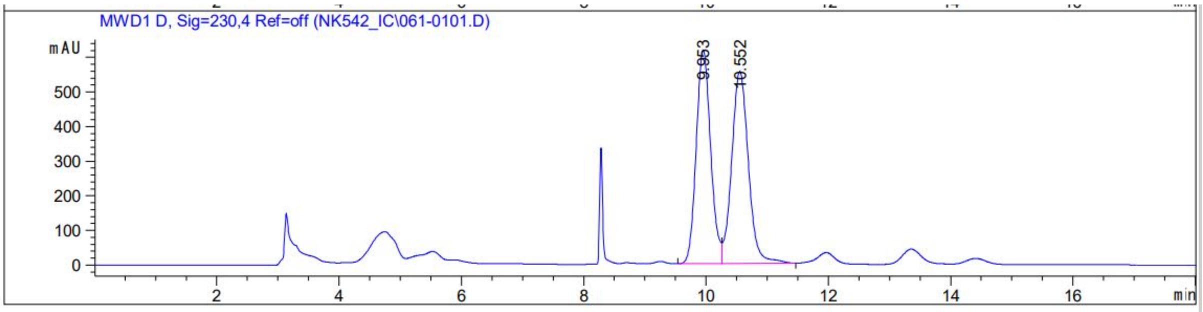


```
Signal 4: MWD1 D, Sig=230,4 Ref=off

Peak RetTime Type  Width     Area       Height      Area
  #   [min]         [min]   [mAU*s]      [mAU]        %
----|-------|----|-------|----------|----------|--------|
   1   9.953 BV    0.2531 1.01114e4   617.17426  48.9996
   2  10.552 VB    0.2933 1.05243e4   554.71332  51.0004

Totals :                  2.06358e4  1171.88757
```

HPLC o (*S*)-4-(4-chlorophenyl)-4-hydroxy-1-(2,3,5,6-tetramethylphenyl)butan-1-one **28b.**

(*S,S*)-3C-tethered Ru(II)-TsDPEN catalyst (after 48 h, 100% conversion, 85% ee (*S*).

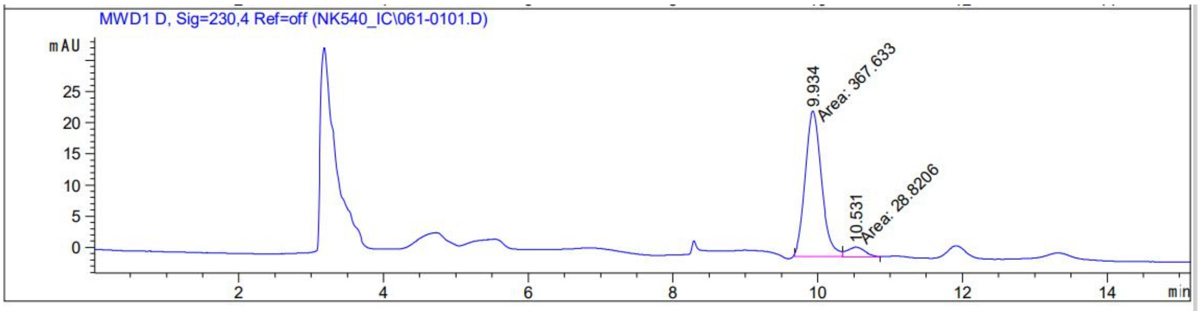


```
Signal 4: MWD1 D, Sig=230,4 Ref=off

Peak RetTime Type  Width     Area       Height      Area
  #   [min]         [min]   [mAU*s]      [mAU]        %
----|-------|----|-------|----------|----------|--------|
   1   9.934 MM    0.2623  367.63327   23.36027  92.7304
   2  10.531 MM    0.2973   28.82060    1.61563   7.2696

Totals :                   396.45387   24.97591
```

1-(2,3,5,6-Tetramethylphenyl)-4-(thiophen-2-yl)butane-1,4-dione 29a.

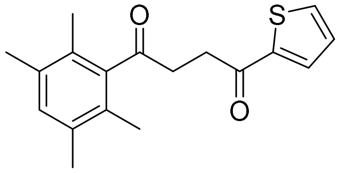

This compound is novel. DBU (131 mg, 0.86 mmol, 0.4 equiv) was added to a stirred solution of thiophene-2-carbaldehyde (362 mg, 3.23 mmol, 1.5 equiv) and thiazolium salt **32** (116 mg, 0.43 mmol, 0.2 equiv) in THF (21 mL). The resulting reaction mixture was stirred in room temperature for 10-15 minutes. (E)-4-Oxo-4-(2,3,5,6-tetramethylphenyl)but-2-enoic acid **31** (500 mg, 2.15 mmol, 1.0 equiv) was added and the mixture stirred at 60 ºC for 4 days and monitored by TLC. After completion of the reaction, it was cooled to room temperature and washed with a saturated solution of sodium bicarbonate and extracted with EtOAc, the organic layer was dried over sodium sulphate and concentrated under reduced pressure. The product was isolated via flash chromatography on silica eluted with 0-10% EtOAc in petroleum ether to yield 1-(2,3,5,6-tetramethylphenyl)-4-(thiophen-2-yl)butane-1,4-dione **29a** as a yellow solid (94.3 mg, 0.314 mmol, 15%). TLC: Rf ca 0.2 (9:1 hexane: EtOAc), strong UV and PMA active; Mp: 146.5 °C; HRMS (ESI+) *m/z*: [M+Na]+ Calcd for $C_{18}H_{20}NaO_2S$ 323.1076; Found 323.1067; 2.8 ppm error; ν_{max} 2915, 1860, 1696, 1666 cm^{-1}; ^1H NMR (400 MHz, $CDCl_3$): δ 7.88 – 7.85 (1H, m, ArH), 7.67 (1H, d, *J* = 5.0, ArH), 7.18 (1H, t, *J* = 4.3, ArH), 7.00 (1H, s, ArH), 3.39 (2H, t, *J* = 6.1, CH_2), 3.20 (2H, t, *J* = 6.2, CH_2), 2.24 (6H, s, CH_3), 2.15 (6H, s, CH_3); $^{13}C\{^1H\}$ NMR (101 MHz, $CDCl_3$): δ 209.6 (C), 191.2 (C), 143.9 (C), 142.1 (C), 134.4 (C), 133.5 (CH), 132.0 (CH), 131.7 (CH), 128.2 (C), 128.1 (CH), 39.1 (CH_2), 32.3 (CH_2), 19.5 (CH_3), 15.9 (CH_3); *m/z* (ES-API+) 323.1 (M^+ + Na, 100%); Enantiomeric excess and conversion determined by HPLC analysis (Chiralpak IA, 30 cm x 6 mm column, hexane:iPrOH 90:10, 1.0 mL/min, T = 25°C) ketone 6.2 min, *R* and *S* isomer 8.1 min and 7.2 min.

^{1}H NMR (500 MHz, $CDCl_3$) of 1-(2,3,5,6-Tetramethylphenyl)-4-(thiophen-2-yl)butane-1,4-dione **29a**.

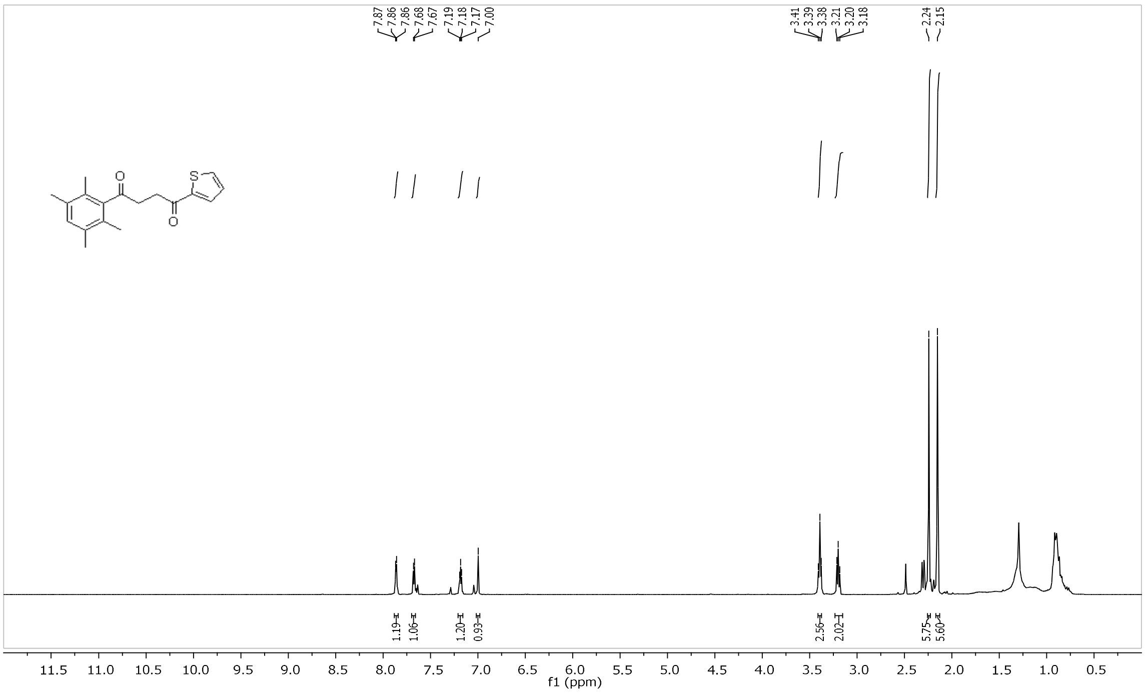

COSY (500 MHz, $CDCl_3$) of 1-(2,3,5,6-Tetramethylphenyl)-4-(thiophen-2-yl)butane-1,4-dione **29a**.

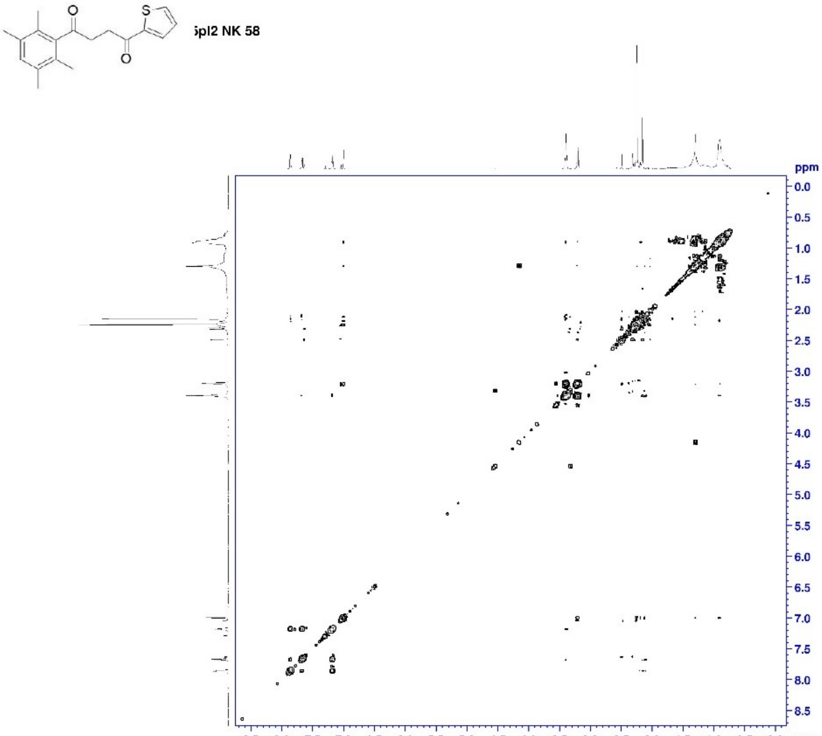

HSQC (126 MHz, $CDCl_3$) of 1-(2,3,5,6-Tetramethylphenyl)-4-(thiophen-2-yl)butane-1,4-dione **29a.**

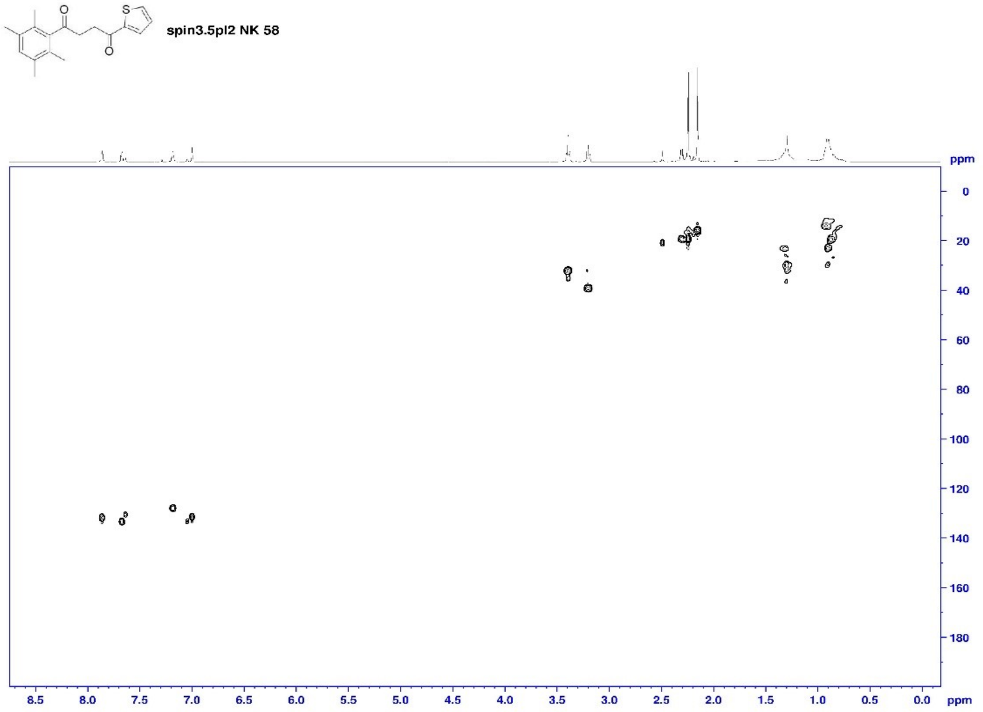

HMBC (126 MHz, $CDCl_3$) of 1-(2,3,5,6-Tetramethylphenyl)-4-(thiophen-2-yl)butane-1,4-dione **29a**.

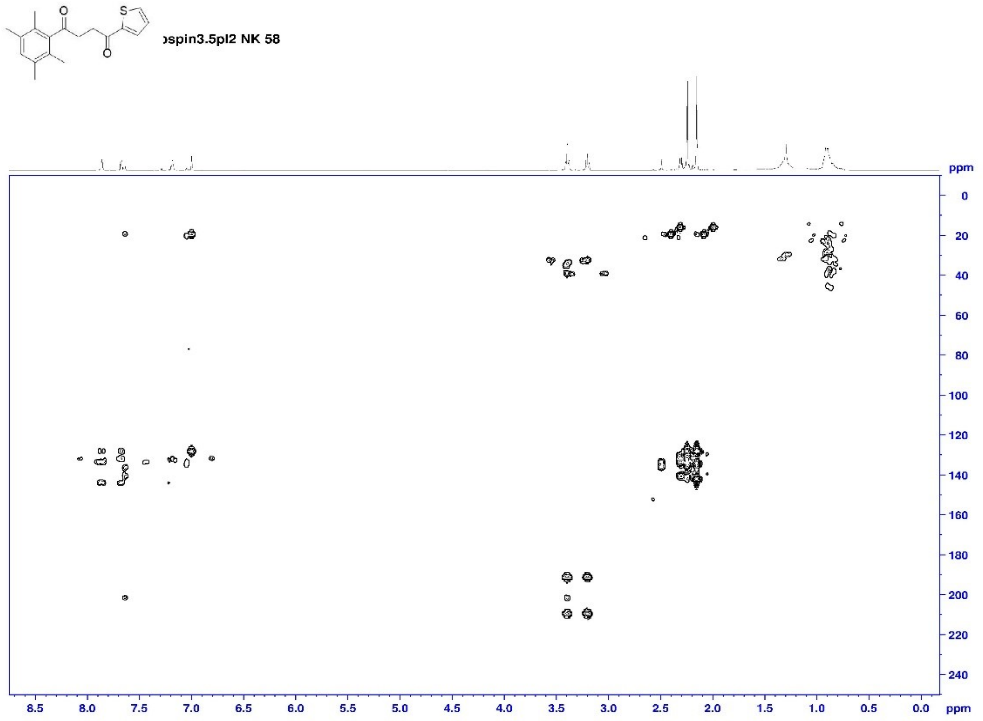

$^{13}C\{^1H\}$ NMR (126 MHz, $CDCl_3$) of 1-(2,3,5,6-Tetramethylphenyl)-4-(thiophen-2-yl)butane-1,4-dione **29a**.

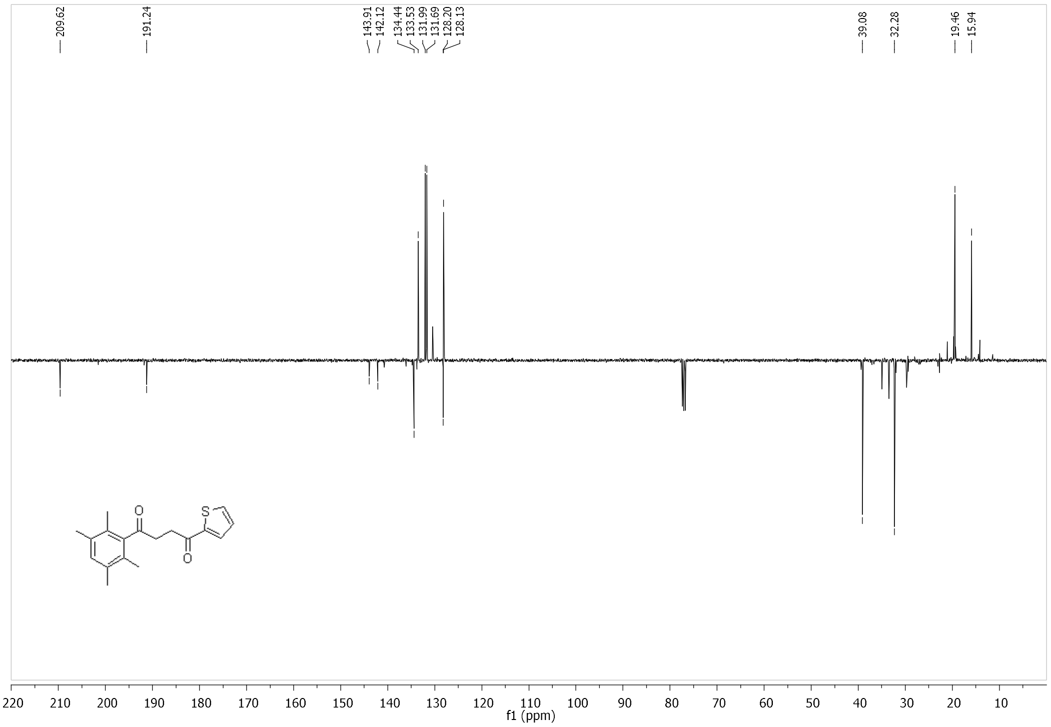

HPLC of 1-(2,3,5,6-Tetramethylphenyl)-4-(thiophen-2-yl)butane-1,4-dione **29a**.

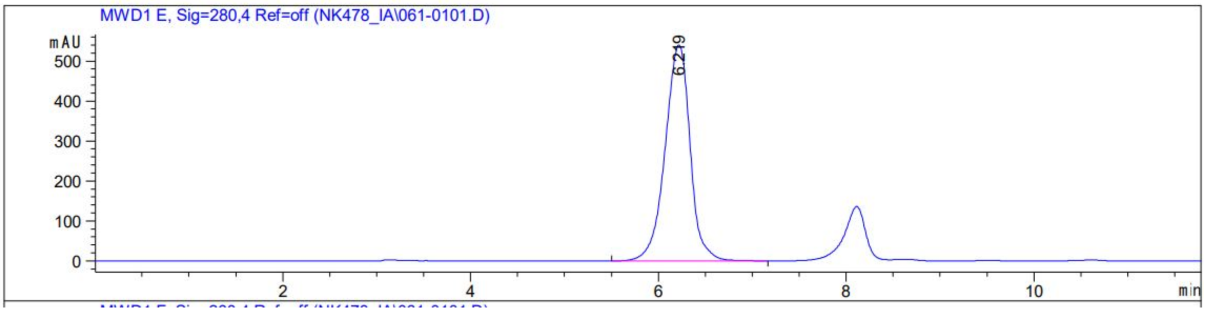

Signal 5: MWD1 E, Sig=280,4 Ref=off

Peak #	RetTime [min]	Type	Width [min]	Area [mAU*s]	Height [mAU]	Area %
1	6.219	VB	0.2880	1.00009e4	540.19489	100.0000
Totals :				1.00009e4	540.19489	

4-Hydroxy-1-(2,3,5,6-tetramethylphenyl)-4-(thiophen-2-yl)butan-1-one 29b.

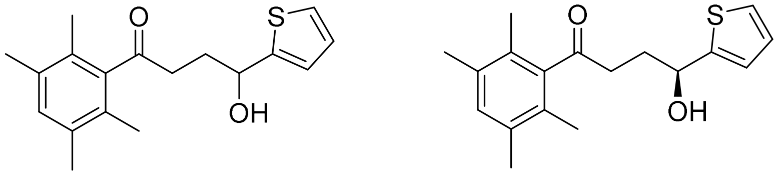

This compound is novel. **Synthesis of a racemic standard**: (*R,R*)-3C-Tethered Ru(II)-TsDPEN catalyst (0.4 mg, 0.0006 mmol, 0.5 mol%) and (*S,S*)-3C-tethered Ru(II)-TsDPEN catalyst (0.4 mg, 0.0006 mmol, 0.5 mol%) were added to FA: TEA (5:2 azeotropic mixture, 0.15 mL) at rt and the mixture was stirred under a nitrogen atmosphere for 15 minutes, after which 1-(2,3,5,6-tetramethylphenyl)-4-(thiophen-2-yl)butane-1,4-dione **29a** (40.0 mg, 0.133 mmol) was added in DCM (0.2 mL). The reaction mixture was stirred under a nitrogen atmosphere and followed by TLC (5:1 hexane: EtOAc). After 48 h, the reaction was quenched using saturated $NaHCO_3$ solution (20 mL). EtOAc (20 mL) was added, and the organic layer was separated. The aqueous layer was extracted with EtOAc (3 x 20 mL) and the combined organic layers were dried ($MgSO_4$) and filtered. The solvent was removed to give the crude product. The product was isolated via flash chromatography on silica eluted with 0-50% EtOAc in petroleum ether to give 4-hydroxy-1-(2,3,5,6-tetramethylphenyl)-4-(thiophen-2-yl)butan-1-one **29b** as a white solid (5 mg, 0.017 mmol, 12%). TLC: Rf ca 0.3 (4:1 hexane: EtOAc), PMA active; Mp: 101.9 °C; HRMS (ESI+) *m/z*: [M+Na]+ Calcd for $C_{18}H_{22}NaO_2S$ 325.1233; Found 325.1224; 2.6 ppm error; ν_{max} 3499, 2967, 1908, 1678 cm^{-1}; ^{1}H NMR (400 MHz, $CDCl_3$): δ 7.18 (1H, d, *J* = 6.4, ArH), 6.90 (2H, m, ArH), 6.87 (1H, s, ArH), 5.11 – 4.94 (1H, m, ArCH), 2.78 (2H, t, *J* = 6.7, CH_2), 2.55 (1H, s, OH), 2.29 – 2.20 (2H, m, CH_2), 2.12 (6H, s, CH_3), 1.96 (6H, s, CH_3); ^{13}C{^{1}H} NMR (101 MHz, $CDCl_3$): δ 212.0 (C), 148.3 (C), 142.5 (C), 134.4 (C), 131.6 (CH), 127.9 (C), 126.8 (CH), 124.6 (CH), 123.7 (CH), 69.5 (CH), 41.4 (CH_2), 32.4 (CH_2), 19.4 (CH_3), 15.9 (CH_3); *m/z* (ES-API+) 325.1 (M^+ + Na, 100%); Enantiomeric excess and conversion determined by HPLC analysis (Chiralpak IA, 30 cm x 6 mm column, hexane:iPrOH 90:10, 1.0 mL/min, T = 25°C) ketone 6.2 min, *R* and *S* isomer 8.1 min and 7.2 min.

(S)-4-Hydroxy-1-(2,3,5,6-tetramethylphenyl)-4-(thiophen-2-yl)butan-1-one **29b**.

(S,S)-3C-Tethered Ru(II)-TsDPEN catalyst (0.4 mg, 0.00064 mmol, 1 mol%) was added to FA: TEA (5:2 azeotropic mixture, 0.04 mL) at rt and the mixture was stirred under a nitrogen atmosphere for 10-15 minutes; after which 1-(2,3,5,6-tetramethylphenyl)-4-(thiophen-2-yl)butane-1,4-dione **29a** (20 mg, 0.066 mmol) was added in DCM (0.15 mL). The reaction mixture was stirred under a nitrogen atmosphere for 48 h. The reaction was followed by TLC (5:1 hexane: EtOAc). After 48 h, the reaction was quenched using saturated $NaHCO_3$ solution (20 mL). EtOAc (20 mL) was added and the organic layer was separated. The aqueous layer was extracted with EtOAc (3 x 20 mL) and the combined organic layers were dried ($MgSO_4$) and filtered. The solvent was removed to give the crude product. The product was isolated via flash chromatography on silica eluted with 0-50% EtOAc in petroleum ether to give (S)-4-hydroxy-1-(2,3,5,6-tetramethylphenyl)-4-(thiophen-2-yl)butan-1-one **29b** as a white solid (7.8 mg, 0.026 mmol, 39%). The reaction was also followed by HPLC (Chiralpak IA , 30 cm x 6 mm column, hexane:iPrOH 90:10, 1.0 mL/min, T = 25°C); $[\alpha]_D^{29}$ + 69.1 (c 0.03 in $CHCl_3$); (after 48 h, 75% ee (S)).

^{1}H NMR (400 MHz, $CDCl_3$) of (S)-4-hydroxy-1-(2,3,5,6-tetramethylphenyl)-4-(thiophen-2-yl)butan-1-one **29b**.

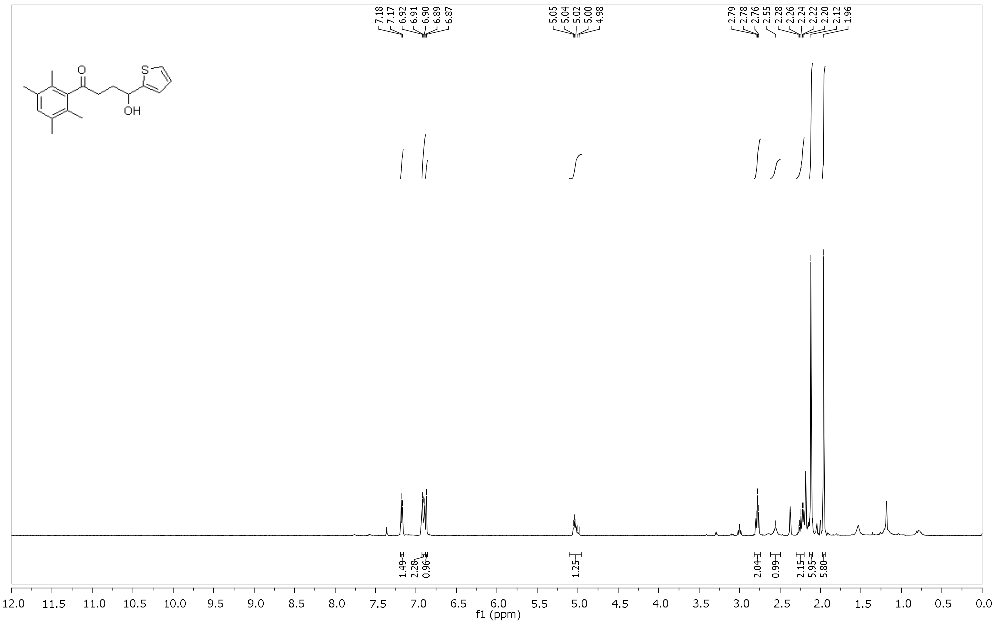

COSY (400 MHz, $CDCl_3$) of (*S*)-4-hydroxy-1-(2,3,5,6-tetramethylphenyl)-4-(thiophen-2-yl)butan-1-one **29b**.

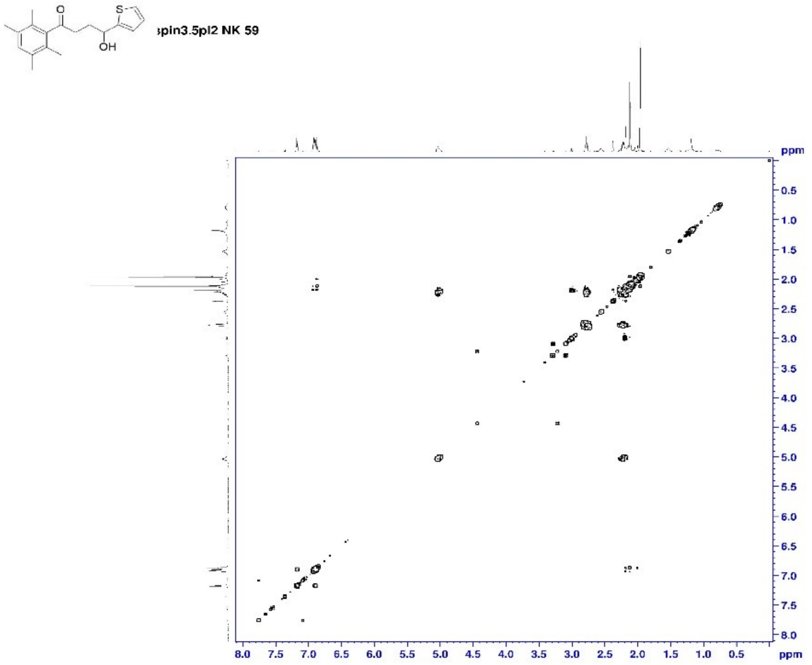

HSQC (126 MHz, $CDCl_3$) of (*S*)-4-hydroxy-1-(2,3,5,6-tetramethylphenyl)-4-(thiophen-2-yl)butan-1-one **29b**.

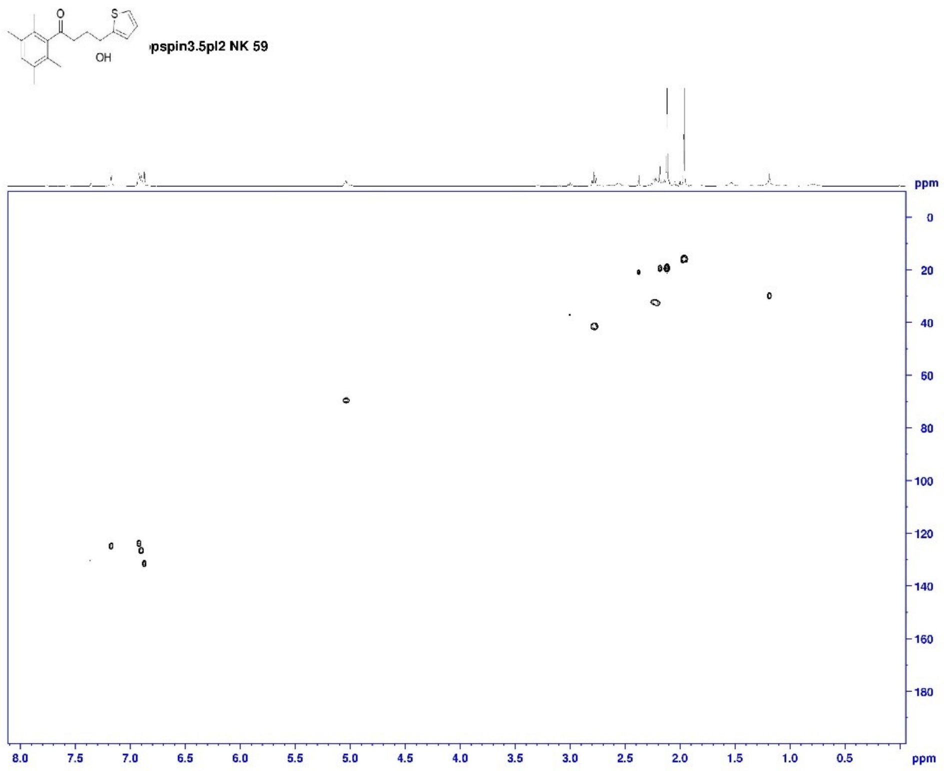

HMBC (126 MHz, $CDCl_3$) of (*S*)-4-hydroxy-1-(2,3,5,6-tetramethylphenyl)-4-(thiophen-2-yl)butan-1-one **29b**.

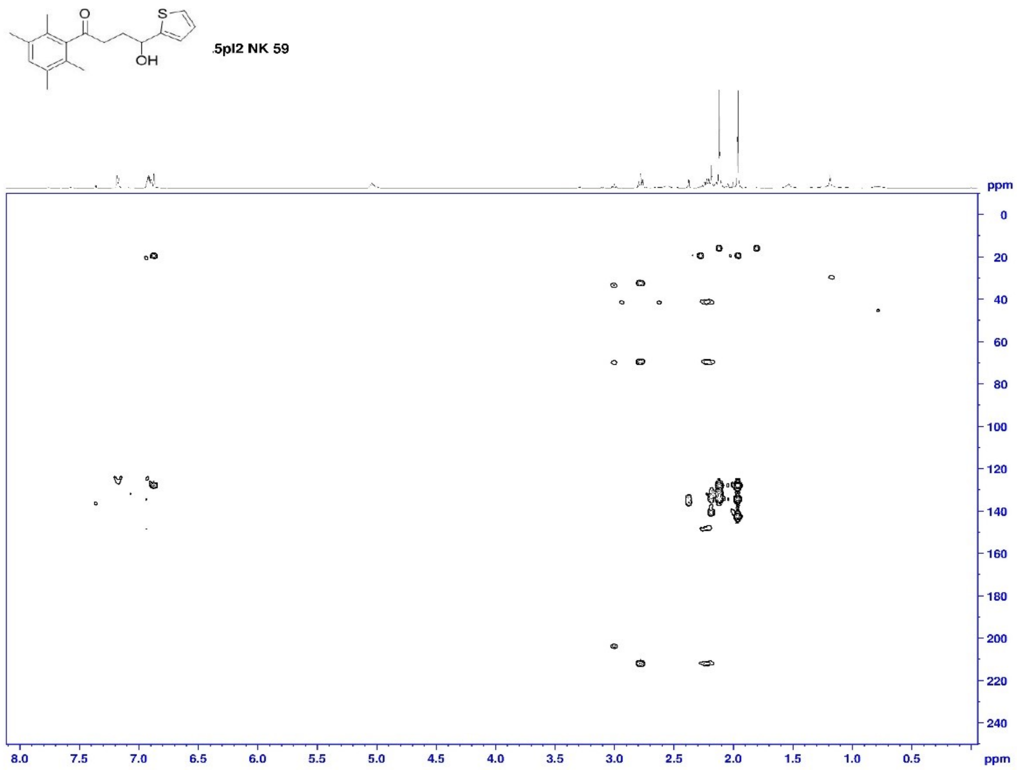

$^{13}C\{^{1}H\}$ NMR (101 MHz, $CDCl_3$) of (*S*)-4-hydroxy-1-(2,3,5,6-tetramethylphenyl)-4-(thiophen-2-yl)butan-1-one **29b**.

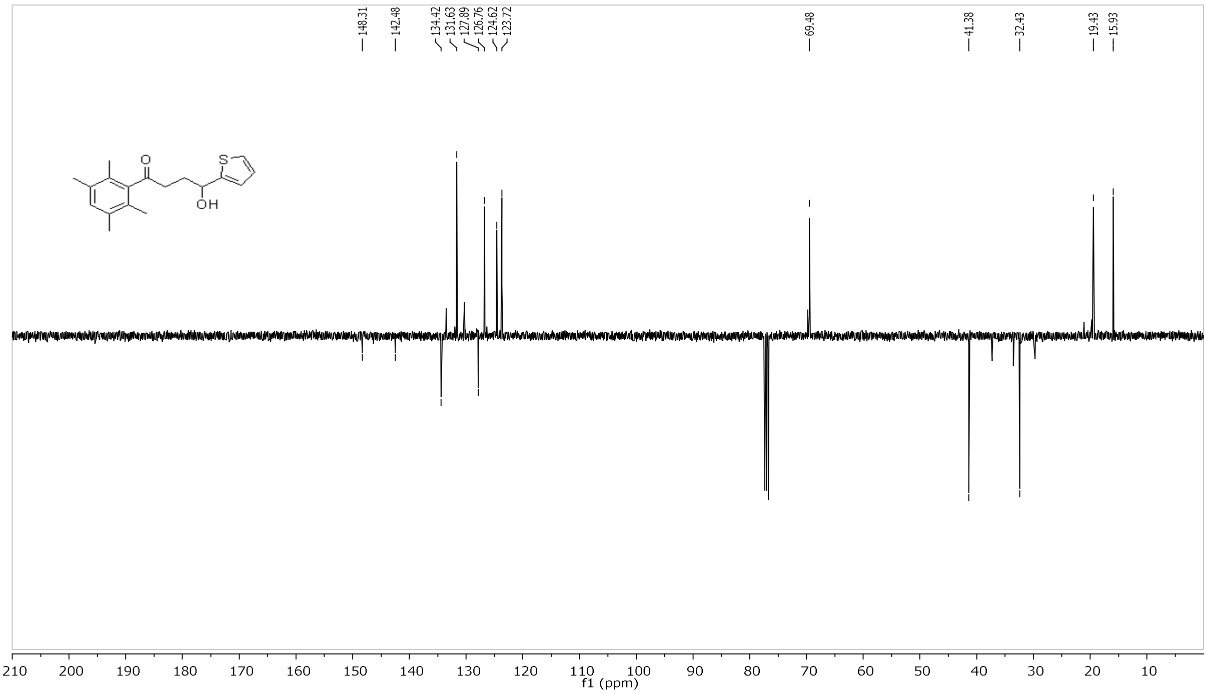

HPLC of racemic of (*S*)-4-hydroxy-1-(2,3,5,6-tetramethylphenyl)-4-(thiophen-2-yl)butan-1-one **29b**.

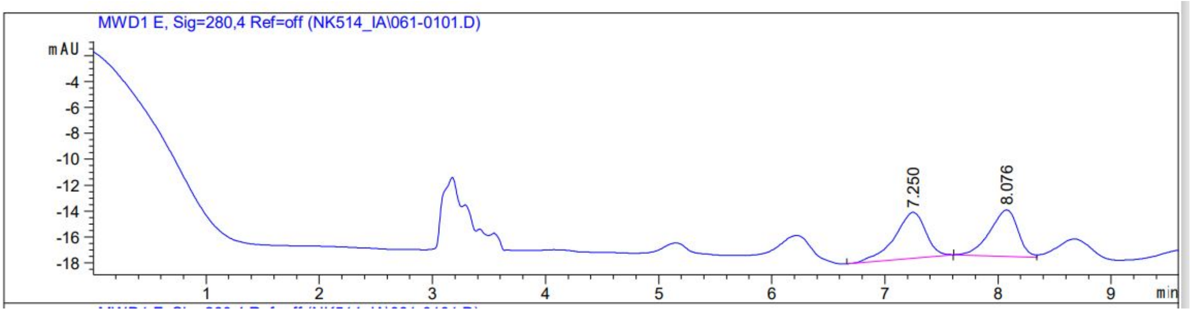

Signal 5: MWD1 E, Sig=280,4 Ref=off

Peak #	RetTime [min]	Type	Width [min]	Area [mAU*s]	Height [mAU]	Area %
1	7.250	BB	0.2854	67.67383	3.53482	52.1091
2	8.076	BV	0.2618	62.19559	3.59645	47.8909

HPLC of (*S*)-4-hydroxy-1-(2,3,5,6-tetramethylphenyl)-4-(thiophen-2-yl)butan-1-one **29b**.

(*S,S*)-3C-tethered Ru(II)-TsDPEN catalyst (after 48 h, 75% ee (*S*)).

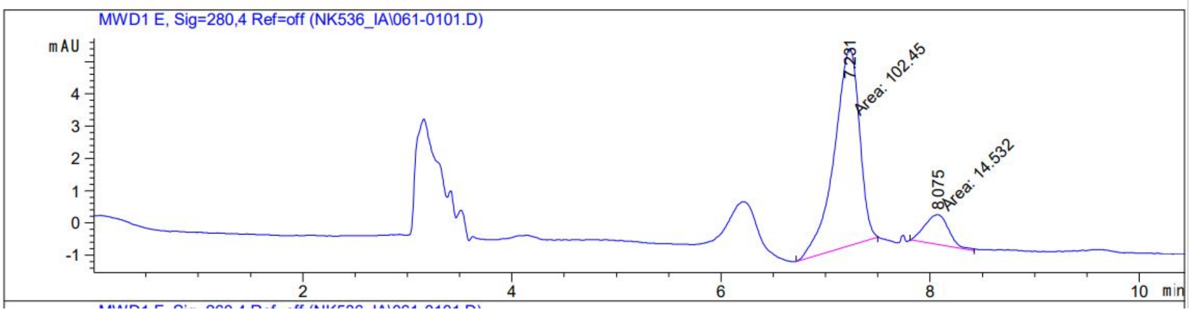

Signal 5: MWD1 E, Sig=280,4 Ref=off

Peak #	RetTime [min]	Type	Width [min]	Area [mAU*s]	Height [mAU]	Area %
1	7.231	MM	0.2795	102.45023	6.10863	87.5776
2	8.075	MM	0.2620	14.53197	9.24266e-1	12.4224

1-(Furan-2-yl)-4-(2,3,5,6-tetramethylphenyl)butane-1,4-dione 30a.

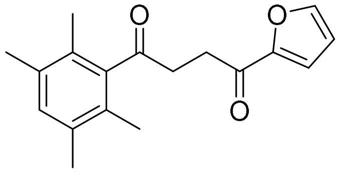

This compound is novel. DBU (131 mg, 0.86 mmol, 0.4 equiv) was added to the stirred solution of furan-2-carbaldehyde (374 mg, 3.9 mmol, 1.5 equiv) and thiazolium salt **32** (116 mg, 0.43 mmol, 0.2 equiv) in THF (21 mL). The resulting reaction mixture was stirred at room temperature for 10-15 minutes. After that (E)-4-oxo-4-(2,3,5,6-tetramethylphenyl) but-2-enoic acid **31** (500 mg, 2.15 mmol, 1.0 equiv) was added and the mixture stirred at 60 °C overnight and monitored by TLC. After completion of the reaction, it was cooled to room temperature and washed with saturated solution of sodium bicarbonate (20 mL) and extracted with EtOAc (3 x 50 mL), organic layer was dried over sodium sulphate and concentrated under reduced pressure. The product was isolated via flash chromatography on silica eluted with 0-25% EtOAc in petroleum ether to give 1-(furan-2-yl)-4-(2,3,5,6-tetramethylphenyl)butane-1,4-dione **30a** as a white solid (128 mg, 0.45 mmol, 21%). TLC: Rf ca 0.1 (10:1 hexane: EtOAc), strong UV and strong PMA; Mp: 139.0 °C; HRMS (ESI+) *m/z*: [M+Na]+ Calcd for $C_{18}H_{20}NaO_3$ 307.1305; Found 307.1297; 2.5 ppm error; ν_{max} 3136, 2962, 2931, 1696, 1563 cm^{-1}; ^1H NMR (400 MHz, $CDCl_3$): δ 7.61 (1H, s, ArH), 7.31 – 7.25 (1H, m, ArH), 6.96 (1H, s, ArH), 6.56 (1H, m, ArH), 3.27 (2H, t, *J* = 6.3, CH_2), 3.15 (2H, t, *J* = 6.2, CH_2), 2.21 (6H, s, CH_3), 2.11 (6H, s, CH_3); $^{13}C\{^1H\}$ NMR (101 MHz, $CDCl_3$): δ 209.6 (C), 187.6 (C), 152.6 (C), 146.3 (CH), 142.1 (C), 134.4 (C), 131.7 (CH), 128.2 (C), 117.1 (CH), 112.3 (CH), 38.7 (CH_2), 31.4 (CH_2), 19.5 (CH_3), 15.9 (CH_3); *m/z* (ES-API+) 307.1 (M^+ + Na, 100%); Enantiomeric excess and conversion determined by HPLC analysis (Chiralpak OD-H, 30 cm x 6 mm column, hexane:iPrOH 90:10, 1.0 mL/min, T = 25°C) ketone 8.0 min, *R* and *S* isomer 8.4 min and 9.6 min.

^{1}H NMR (400 MHz, $CDCl_3$) of 1-(furan-2-yl)-4-(2,3,5,6-tetramethylphenyl)butane-1,4-dione **30a**.

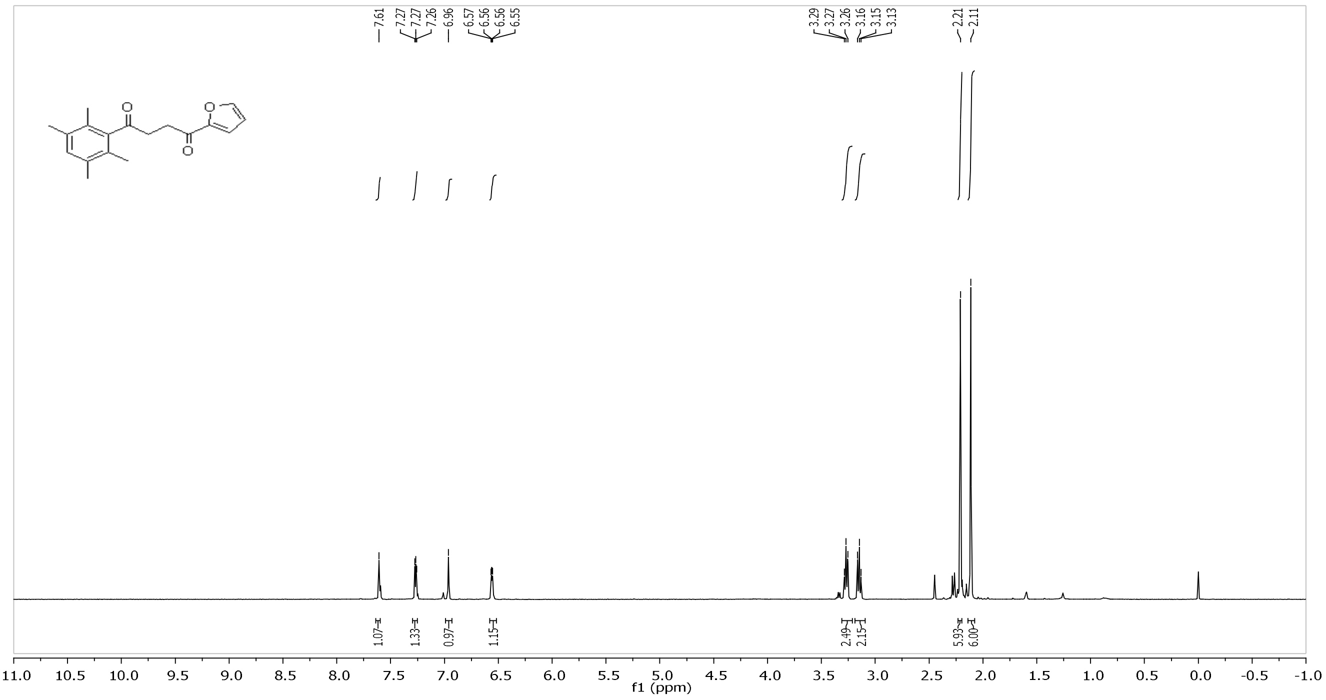

COSY (400 MHz, $CDCl_3$) of 1-(furan-2-yl)-4-(2,3,5,6-tetramethylphenyl)butane-1,4-dione **30a**.

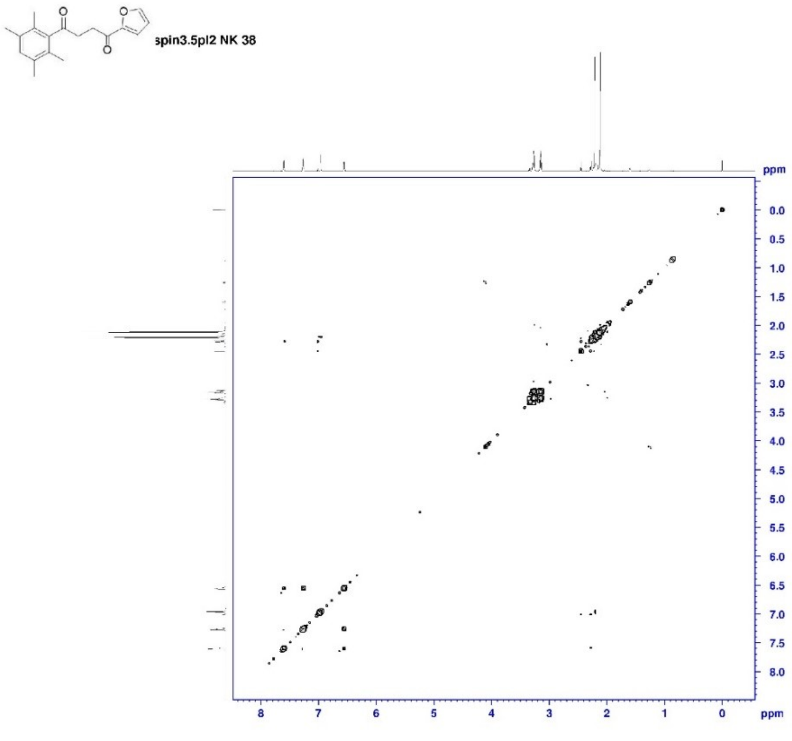

HSQC (101 MHz, $CDCl_3$) of 1-(furan-2-yl)-4-(2,3,5,6-tetramethylphenyl)butane-1,4-dione **30a**.

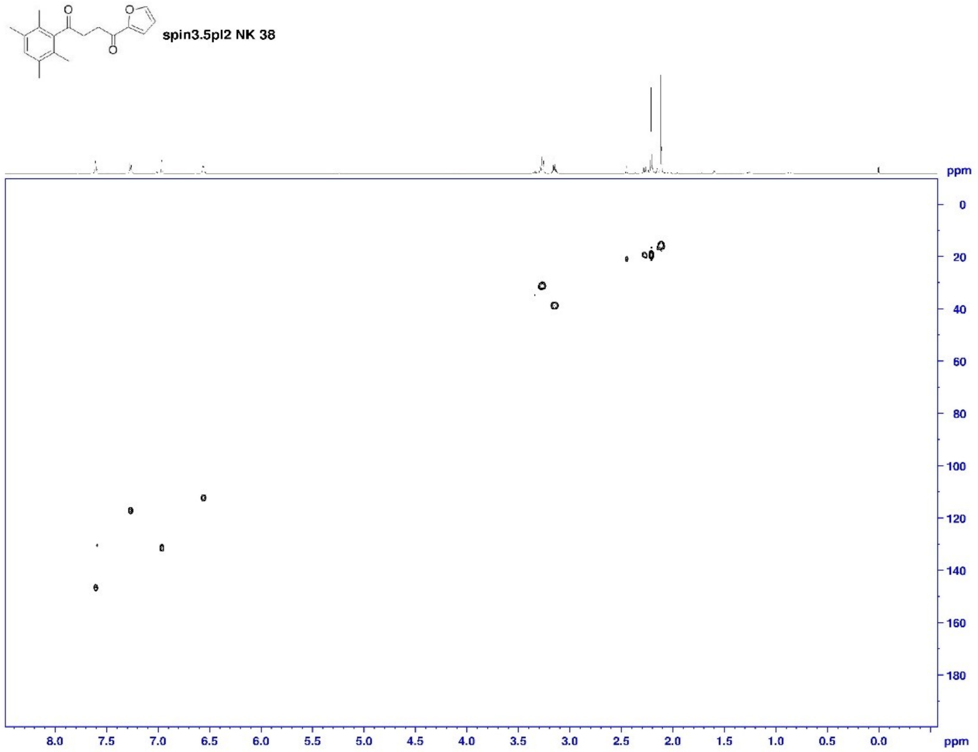

HMBC (101 MHz, $CDCl_3$) of 1-(furan-2-yl)-4-(2,3,5,6-tetramethylphenyl)butane-1,4-dione **30a**.

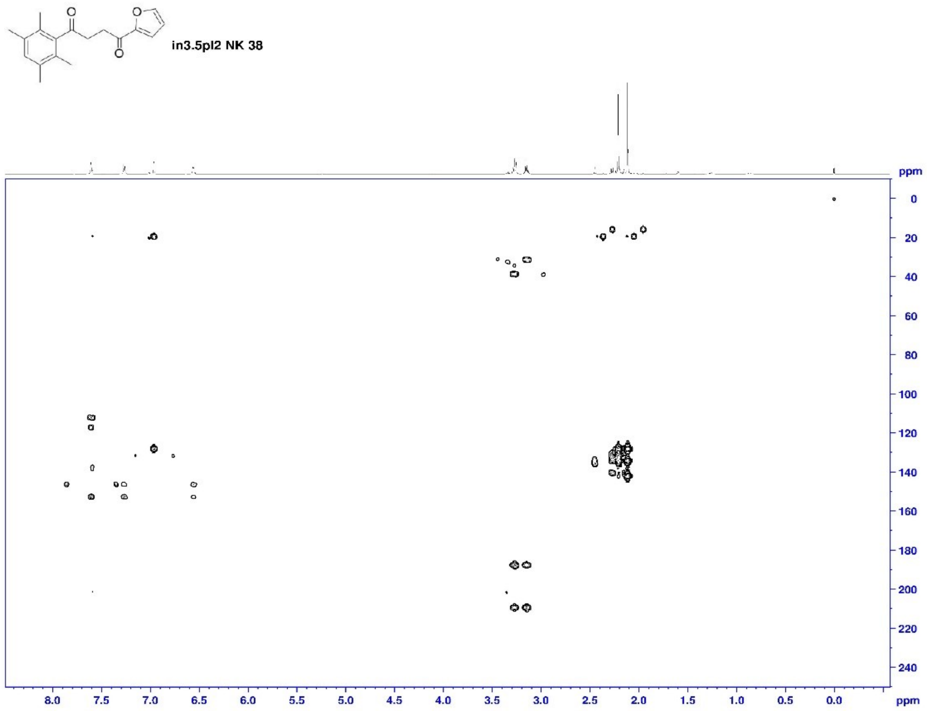

$^{13}C\{^{1}H\}$ NMR (101 MHz, $CDCl_3$) of 1-(furan-2-yl)-4-(2,3,5,6-tetramethylphenyl)butane-1,4-dione **30a**.

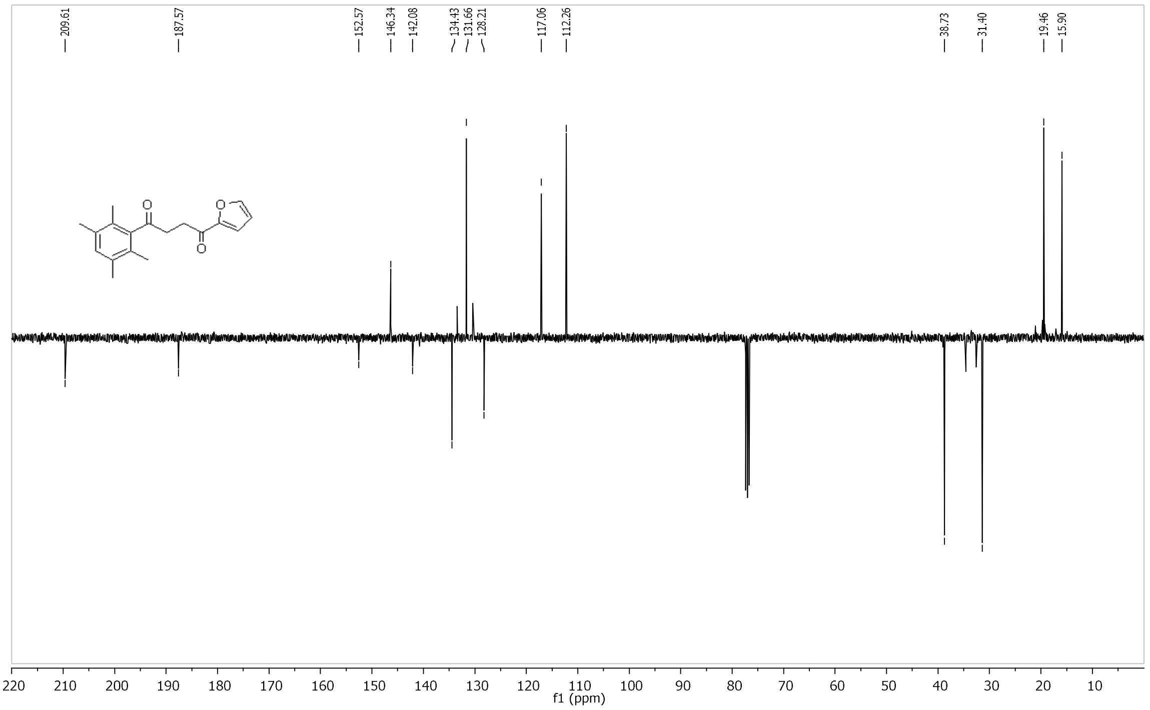

HPLC of of 1-(furan-2-yl)-4-(2,3,5,6-tetramethylphenyl)butane-1,4-dione **30a**.

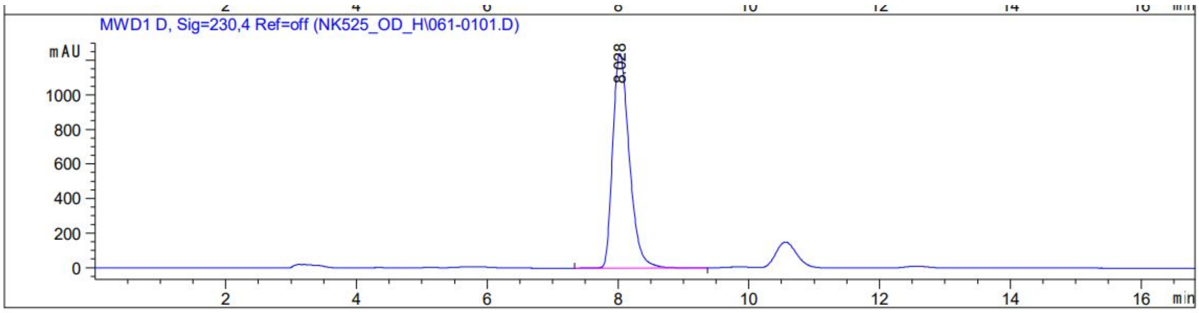

Signal 4: MWD1 D, Sig=230,4 Ref=off

Peak #	RetTime [min]	Type	Width [min]	Area [mAU*s]	Height [mAU]	Area %
1	8.028	BB	0.2764	2.19681e4	1241.60132	100.0000
Totals :				2.19681e4	1241.60132	

4-(Furan-2-yl)-4-hydroxy-1-(2,3,5,6-tetramethylphenyl)butan-1-one 30b.

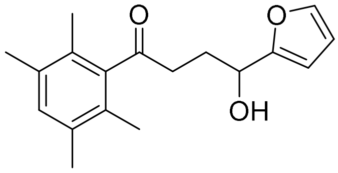

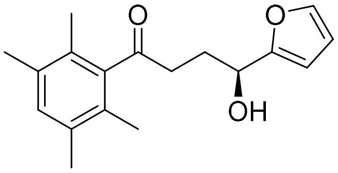

This compound is novel. **Synthesis of a racemic standard**: (*R,R*)-3C-Tethered Ru(II)-TsDPEN catalyst (0.4 mg, 0.00065 mmol, 0.5 mol%) and (*S,S*)-3C-tethered Ru(II)-TsDPEN catalyst (0.4 mg, 0.00065 mmol, 0.5 mol%) were added to FA: TEA (5:2 azeotropic mixture, 0.1 mL) at rt and the mixture was stirred under a nitrogen atmosphere for 15 minutes; after which 1-(furan-2-yl)-4-(2,3,5,6-tetramethylphenyl)butane-1,4-dione **30a** (40 mg, 0.14 mmol) was added in DCM (0.2 mL). The reaction mixture was stirred under a nitrogen atmosphere and followed by TLC (1:1 hexane: EtOAc). After 48 h, the reaction was quenched using saturated $NaHCO_3$ solution (20 mL). EtOAc (20 mL) was added, and the organic layer was separated. The aqueous layer was extracted with EtOAc (3 x 20 mL) and the combined organic layers were dried ($MgSO_4$) and filtered. The solvent was removed to give the crude product. The product was isolated via flash chromatography on silica eluted with 0-50% EtOAc in petroleum ether to give 4-(furan-2-yl)-4-hydroxy-1-(2,3,5,6-tetramethylphenyl)butan-1-one **30b** as a white solid (25.8 mg, 0.090 mmol, 69%). TLC: Rf ca 0.6 (1:1 hexane: EtOAc), strong UV and PMA active; Mp: 105 °C; HRMS (ESI+) *m/z*: [M+Na]+ Calcd for $C_{18}H_{22}NaO_3$ 309.1461; Found 309.1452; 3.1 ppm error; ν_{max} 3404, 2992, 2961, 1691 cm^{-1}; ^{1}H NMR (500 MHz, $CDCl_3$): δ 7.38 (1H, s ArH), 6.95 (1H, s, ArH), 6.34 (1H, m, ArH), 6.28 (1H, d, *J* 3.2, ArH), 4.92 – 4.78 (1H, m, ArCH), 2.84 (2H, t, *J* = 6.9, CH_2), 2.36 – 2.27 (2H, m, CH_2), 2.25 (1H, s, OH), 2.19 (6H, s, CH_3), 2.04 (6H, s, CH_3); $^{13}C\{^1H\}$ NMR (126 MHz, $CDCl_3$): δ 211.9 (C), 156.3 (C), 142.49 (C), 142.0 (CH), 134.4 (C), 131.6 (CH), 127.9 (C), 110.2 (CH), 106.1 (CH), 67.1 (CH), 41.2 (CH_2), 28.9 (CH_2), 19.4 (CH_3), 15.9 (CH_3); *m/z* (ES-API+) 309.1 (M^+ + Na, 100%); Enantiomeric excess and conversion determined by HPLC analysis (Chiralpak OD-H, 30 cm x 6 mm column, hexane:iPrOH 90:10, 1.0 mL/min, T = 25°C) ketone 8.0 min, *R* and *S* isomer 8.4 min and 9.6 min.

(*S*)-4-(Furan-2-yl)-4-hydroxy-1-(2,3,5,6-tetramethylphenyl)butan-1-one **30b**.

(*S,S*)-3C-tethered Ru(II)-TsDPEN catalyst (0.87 mg, 0.0014 mmol, 1 mol%) was added to FA: TEA (5:2 azeotropic mixture, 0.1 mL) at rt and the mixture was stirred under a nitrogen atmosphere for 10-15 minutes; after which 1-(furan-2-yl)-4-(2,3,5,6-tetramethylphenyl)butane-1,4-dione **30a** (40 mg, 0.14 mmol) was added in DCM (0.1 mL). The reaction mixture was stirred under a nitrogen atmosphere for 48 h. The reaction was followed by TLC (1:1 hexane: EtOAc). After 48 h, the reaction was quenched using saturated $NaHCO_3$ solution (20 mL). EtOAc (20 mL) was added, and the organic layer was separated. The aqueous layer was extracted with EtOAc (3 x 20 mL), and the combined organic layers were dried ($MgSO_4$) and filtered. The solvent was removed to give the crude product. The product was isolated via flash chromatography on silica eluted with 0-50% EtOAc in petroleum ether to give (*S*)-4-(furan-2-yl)-4-hydroxy-1-(2,3,5,6-tetramethylphenyl)butan-1-one **30b** as a white solid (26.7 mg, 0.093 mmol, 66%). The reaction was also followed HPLC (Chiralpak OD-H, 30 cm x 6 mm column, hexane:iPrOH 90:10, 1.0 mL/min, T = 25°C); $[\alpha]_D^{29}$ + 8.6 (c 0.06 in $CHCl_3$); (after 48 h, 100% conversion, 99% ee (*S*)).

^1H NMR (500 MHz, $CDCl_3$) of 4-(furan-2-yl)-4-hydroxy-1-(2,3,5,6-tetramethylphenyl)butan-1-one **30b**.

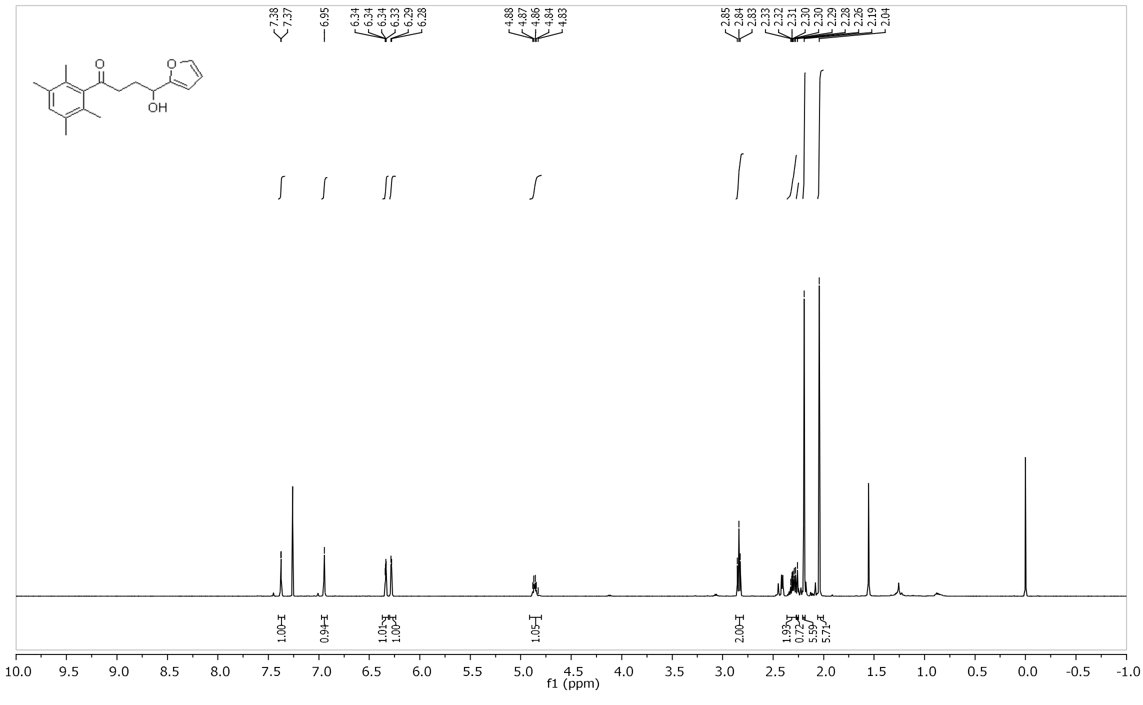

COSY (500 MHz, $CDCl_3$) of 4-(furan-2-yl)-4-hydroxy-1-(2,3,5,6-tetramethylphenyl)butan-1-one **30b**.

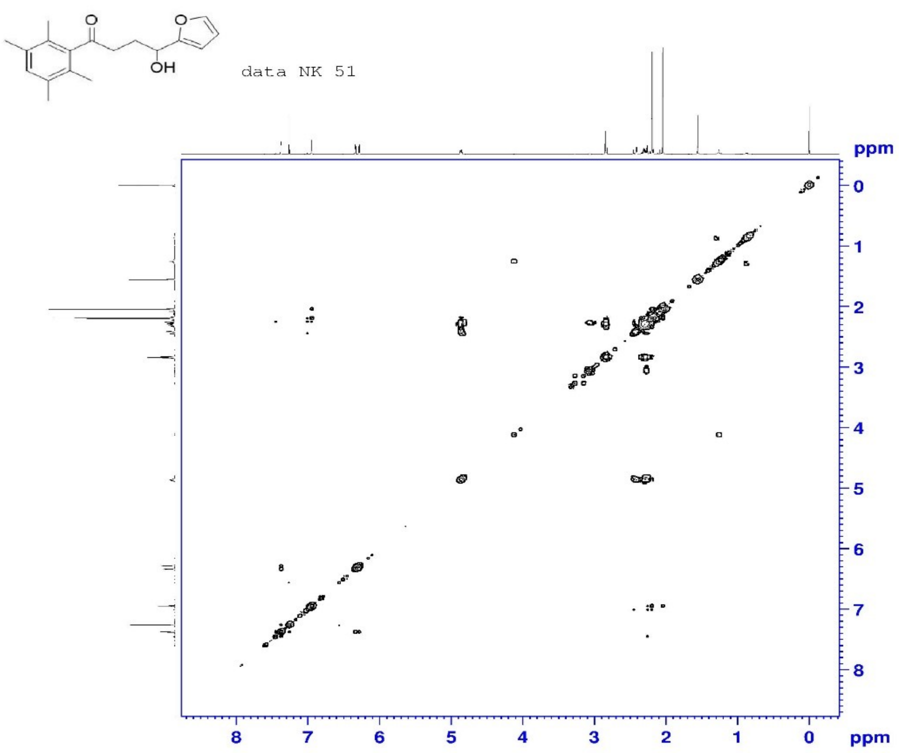

HSQC (101 MHz, $CDCl_3$) of 4-(furan-2-yl)-4-hydroxy-1-(2,3,5,6-tetramethylphenyl)butan-1-one **30b**.

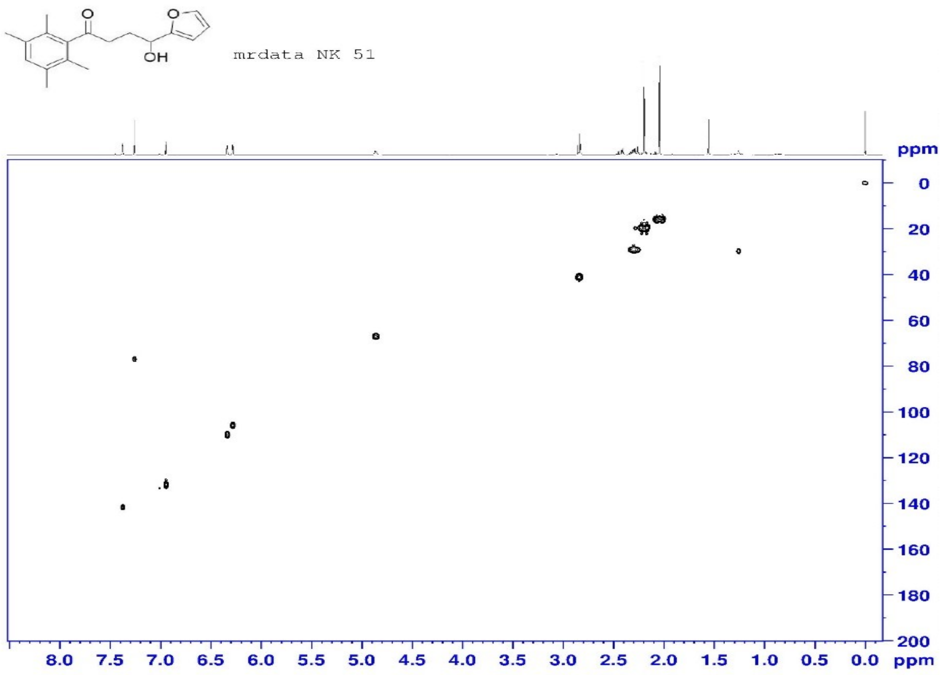

HMBC (101 MHz, $CDCl_3$) of 4-(furan-2-yl)-4-hydroxy-1-(2,3,5,6-tetramethylphenyl)butan-1-one **30b**.

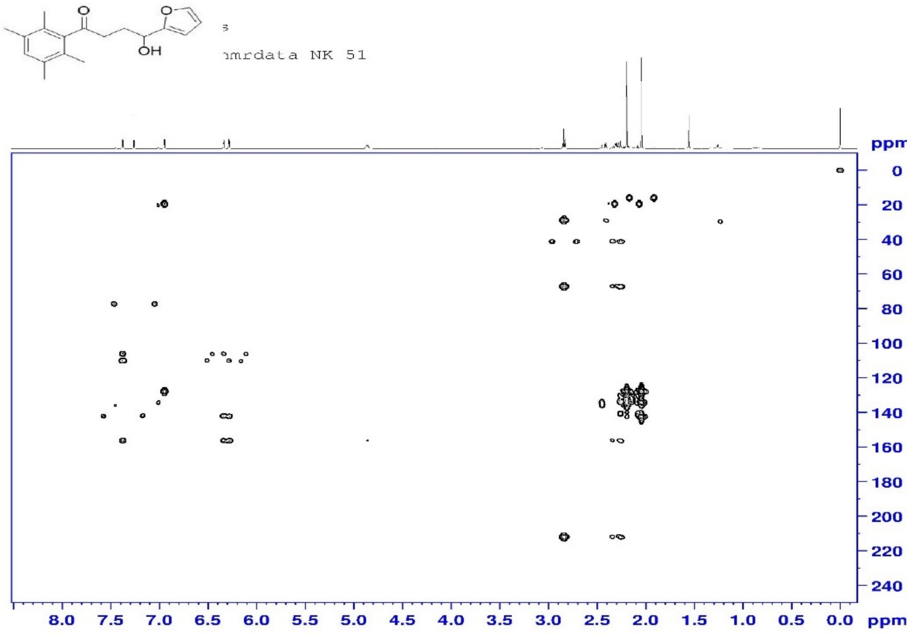

$^{13}C\{^1H\}$ NMR (101 MHz, $CDCl_3$) of 4-(furan-2-yl)-4-hydroxy-1-(2,3,5,6-tetramethylphenyl)butan-1-one **30b**.

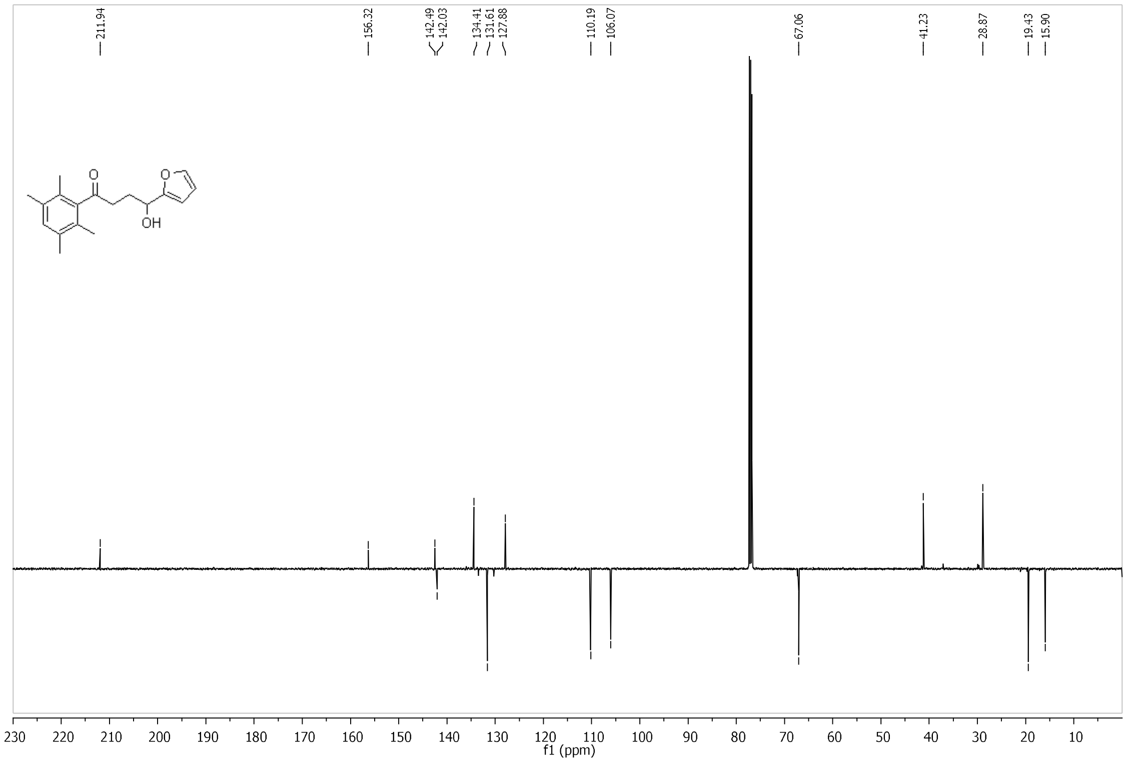

HPLC of racemic 4-(furan-2-yl)-4-hydroxy-1-(2,3,5,6-tetramethylphenyl)butan-1-one **30b**.

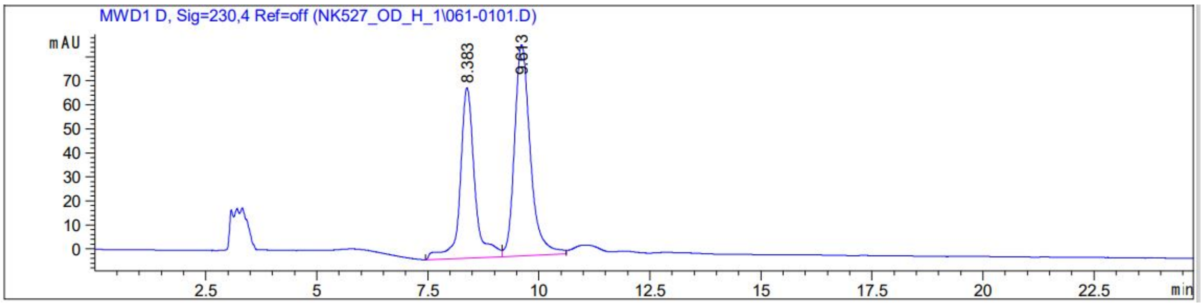

Signal 4: MWD1 D, Sig=230,4 Ref=off

Peak #	RetTime [min]	Type	Width [min]	Area [mAU*s]	Height [mAU]	Area %
1	8.383	BV	0.3424	1620.88550	70.99033	42.6278
2	9.613	VV	0.3796	2181.52661	87.87117	57.3722
Totals :				3802.41211	158.86150	

HPLC of (*S*)-4-(furan-2-yl)-4-hydroxy-1-(2,3,5,6-tetramethylphenyl)butan-1-one **30b**.

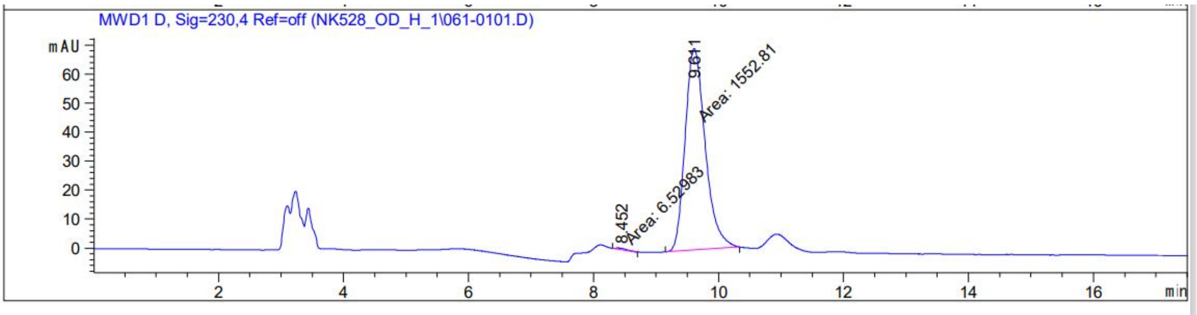

Signal 4: MWD1 D, Sig=230,4 Ref=off

Peak #	RetTime [min]	Type	Width [min]	Area [mAU*s]	Height [mAU]	Area %
1	8.452	MM	0.2222	6.52983	4.89866e-1	0.4188
2	9.611	MM	0.3727	1552.80859	69.43584	99.5812
Totals :				1559.33842	69.92570	

Procedures for 5-hydroxy-5-phenyl-1-(2,3,5,6-tetramethylphenyl)pentan-1-ones 33b and 34b.

Asymmetric Transfer Hydrogenation of 1.5-diketones, overall synthesis:

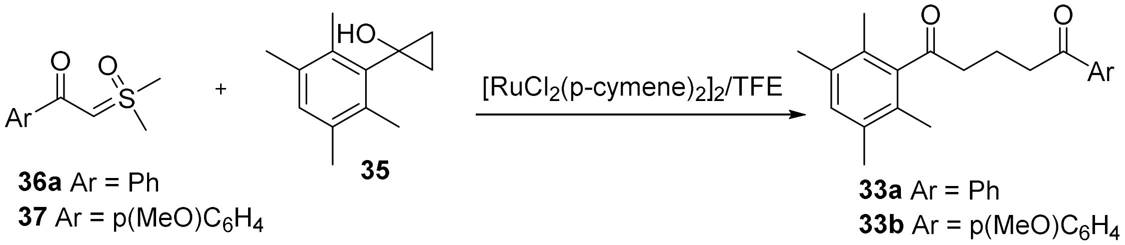

1-(2,3,5,6-Tetramethylphenyl)cyclopropan-1-ol 35.

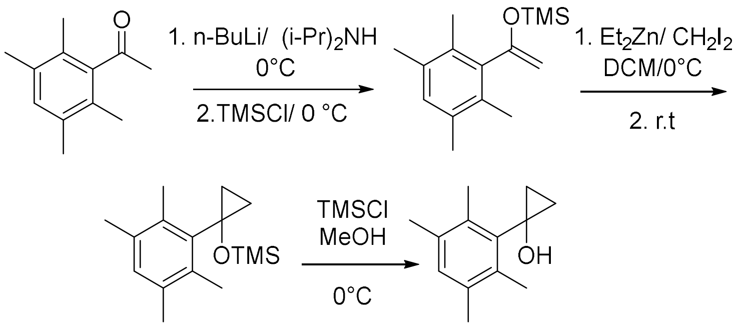

This compound is novel. A dried 100.0 mL round bottomed Schlenk flask was equipped with a stirrer bar. The flask was evacuated, heated, filled with nitrogen three times and sealed with a rubber septum. In a dry nitrogen atmosphere THF (20.0 mL, dry) and $(i\text{-}Pr)_2NH$ (0.72 mL, 12.5 mmol, 1.1 equiv) were added. At 0 °C n-BuLi (5 mL, 2.5 M in hexane, 12.5 mmol, 1.1 equiv) was added to the stirring mixture dropwise via syringe. The mixture was warmed to room temperature and stirred for 1 h. To this mixture at 0 °C, 1-(2,3,5,6-tetramethylphenyl)ethan-1-one **7** (2.00 g, 11.3 mmol, 1.0 equiv) was added by syringe followed by TMSCl (1.6 mL, 1.35 g, 12.4 mmol, 1.1 equiv). After stirring for 2 h at room temperature, it was quenched by addition of $NaHCO_3$ solution (30.0 mL). The layers were separated, and the aqueous layer was extracted with EtOAc (3 × 30 mL). The combined organic layers were washed with a $NaHCO_3$ and brine solution (3 × 20 mL) and dried with Na_2SO_4 followed by filtration. The solvent was removed in vacuum. The crude enol ether was used without further purification. A dried 100 mL round bottomed Schlenk flask was equipped with a stirrer bar. The flask was evacuated, heated, filled with nitrogen three times and sealed with a rubber septum. The crude enol ether was dissolved in dry DCM (23 mL) and was transferred to the prepared Schlenk flask. To the stirring

mixture, CH_2I_2 (4.55 g, 15.0 mmol, 1.5 equiv) was added followed by Et_2Zn (17.0 mL, 1M in hexane, 17.0 mmol, 1.5 equiv) at 0 °C. The mixture was stirred 2 days at room temperature. The reaction mixture was quenched with NH_4Cl solution (30.0 mL) and the precipitate was removed by filtration. The layers were separated, and the aqueous layer was extracted with DCM (3 × 30 mL). The combined organic layers were washed with NaCl solution (3 × 30 mL) and dried with Na_2SO_4 followed by filtration. The solvent was removed in vacuum. A dried 100 mL round bottomed Schlenk flask was equipped with a stir bar. The flask was evacuated, heated, filled with nitrogen three times and sealed with a rubber septum. The crude cyclopropanol ether was solved in MeOH (25.0 mL) and transferred to the prepared Schlenk flask. To the stirring mixture TMSCl (one drop) was added via syringe at 0 ºC. The reaction was monitored by TLC. After 4 days, the solvent was removed in vacuum. The product was isolated via flash chromatography on silica eluted with 0-10% EtOAc in petroleum ether to give 1-(2,3,5,6-tetramethylphenyl)cyclopropan-1-ol **35** as a white solid (686.4 mg, 3.61 mmol, 51%). TLC: Rf ca 0.2 (9:1 hexane: EtOAc), PMA active; Mp: 71.4 °C; HRMS (ESI+) *m/z*: [M+Na]+ Calcd for $C_{13}H_{18}NaO$ 213.1250; Found 213.1260; -4.6 ppm error; ν_{max} 3587, 3483, 3460, 2996, 2917 cm^{-1}; ^{1}H NMR (500 MHz, $CDCl_3$): δ 6.81 (1H, s, ArH), 2.28 (6H, s, CH_3), 2.08 (6H, s, CH_3), 1.18 – 1.08 (2H, m, CH_2), 0.75 – 0.66 (2H, m, CH_2); $^{13}C\{^{1}H\}$ NMR (126 MHz, $CDCl_3$): δ 138.1 (C), 134.6 (C), 134.3 (C), 131.4 (CH), 54.8 (C), 20.1 (CH_2), 16.5 (CH_3), 16.5 (CH_2); *m/z* (ES-API+) 213.1 (M^+ + Na, 100%)

^{1}H NMR (500 MHz, $CDCl_3$) of 1-(2,3,5,6-tetramethylphenyl)cyclopropan-1-ol **35.**

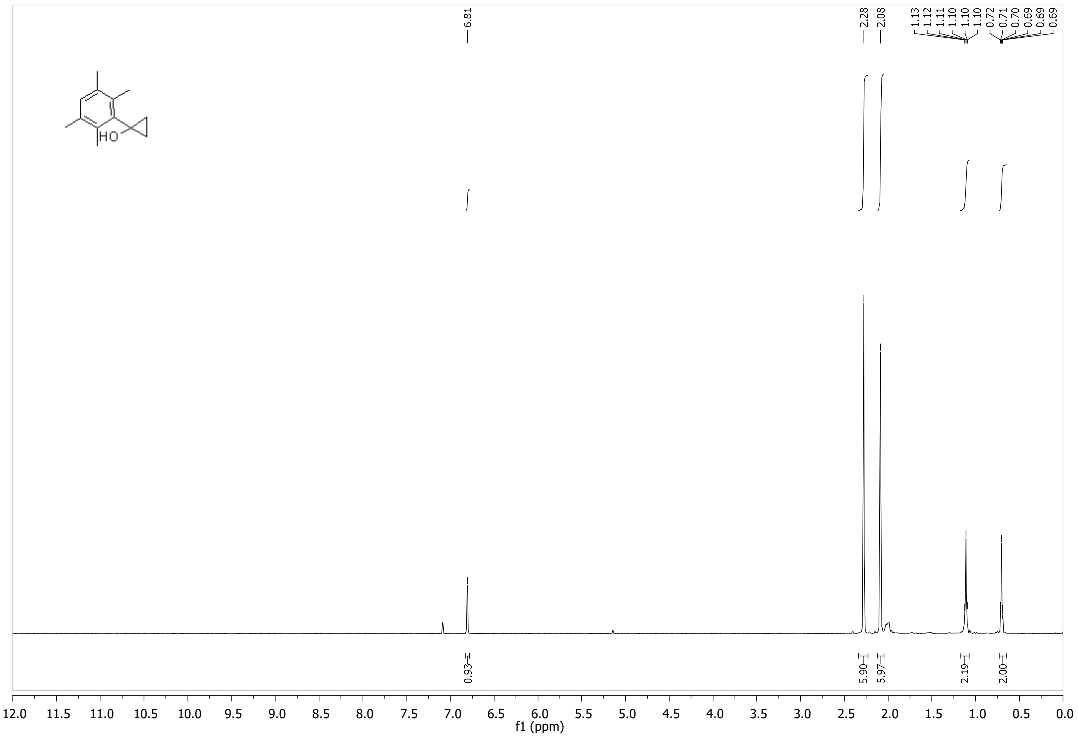

COSY (500 MHz, $CDCl_3$) of 1-(2,3,5,6-tetramethylphenyl)cyclopropan-1-ol **35.**

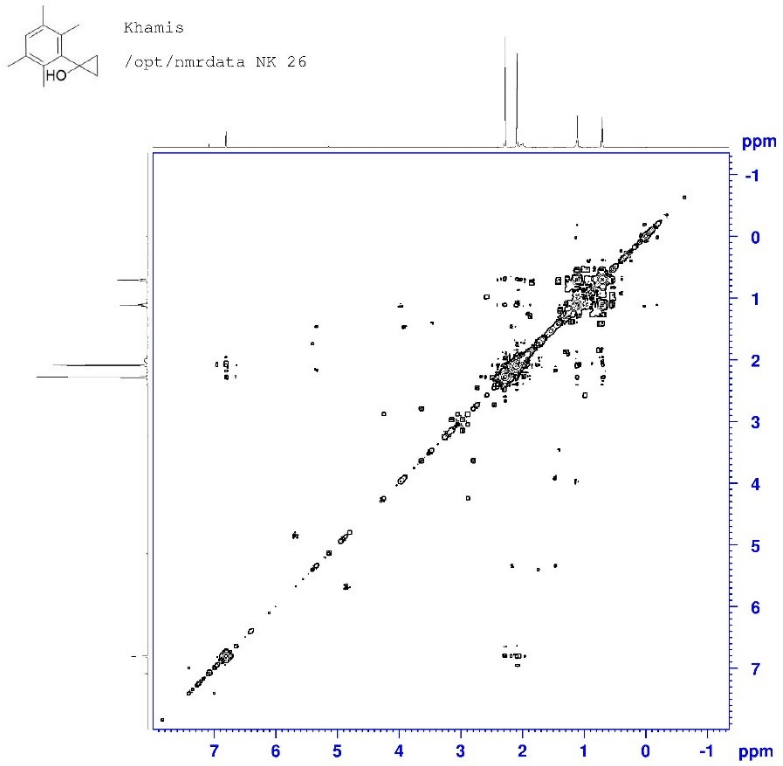

HSQC (126 MHz, $CDCl_3$) of 1-(2,3,5,6-tetramethylphenyl)cyclopropan-1-ol **35.**

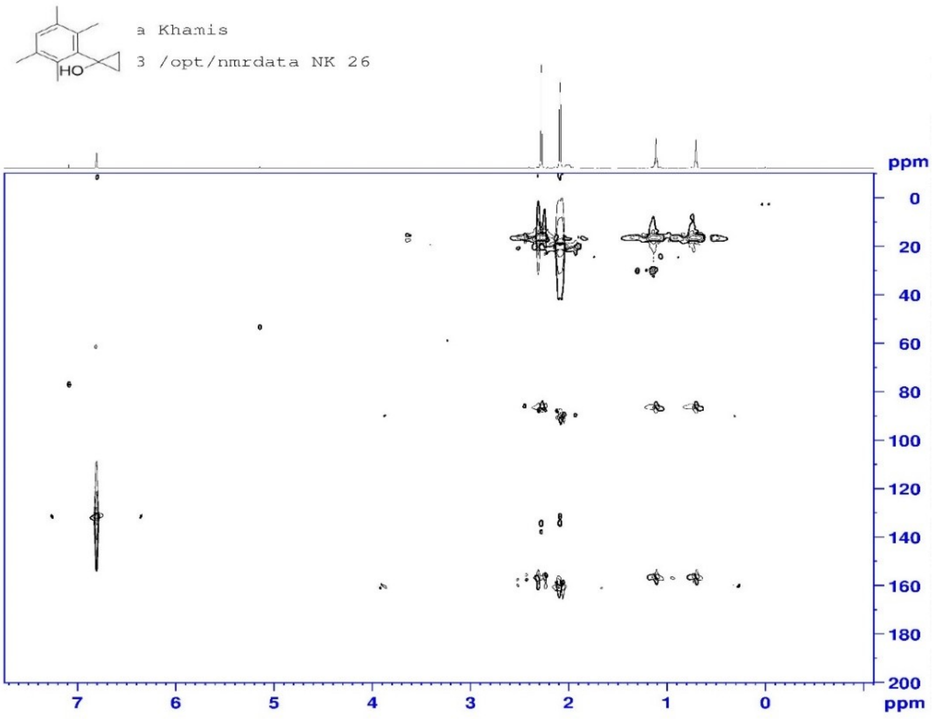

HMBC (126 MHz, $CDCl_3$) of 1-(2,3,5,6-tetramethylphenyl)cyclopropan-1-ol **35.**

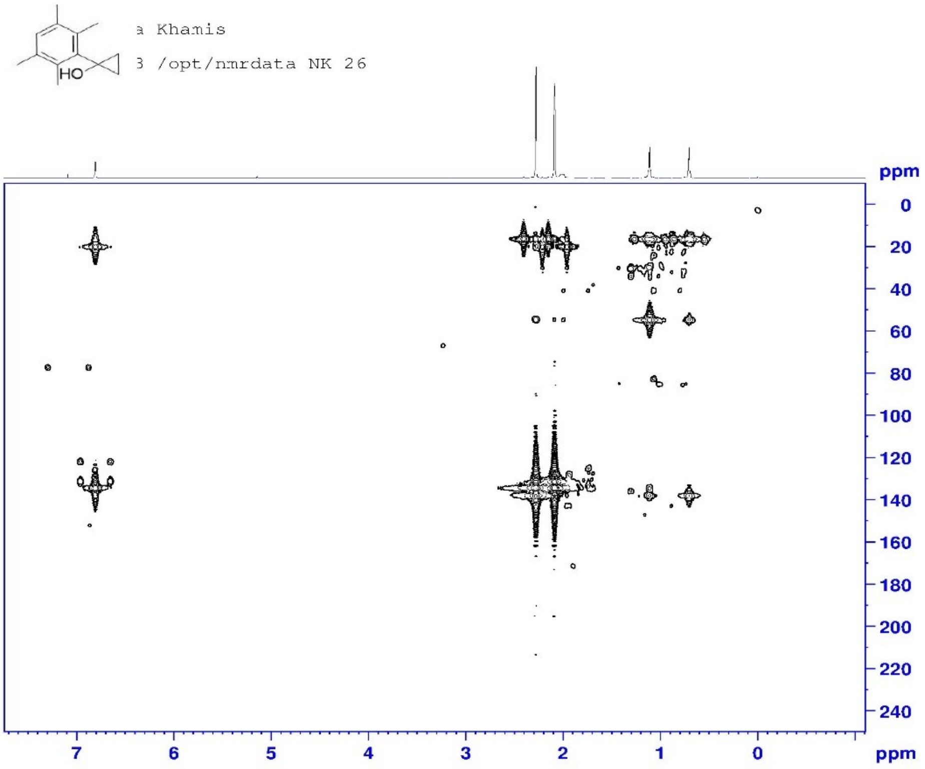

$^{13}C\{^{1}H\}$ NMR (126 MHz, $CDCl_3$) of 1-(2,3,5,6-tetramethylphenyl)cyclopropan-1-ol **35.**

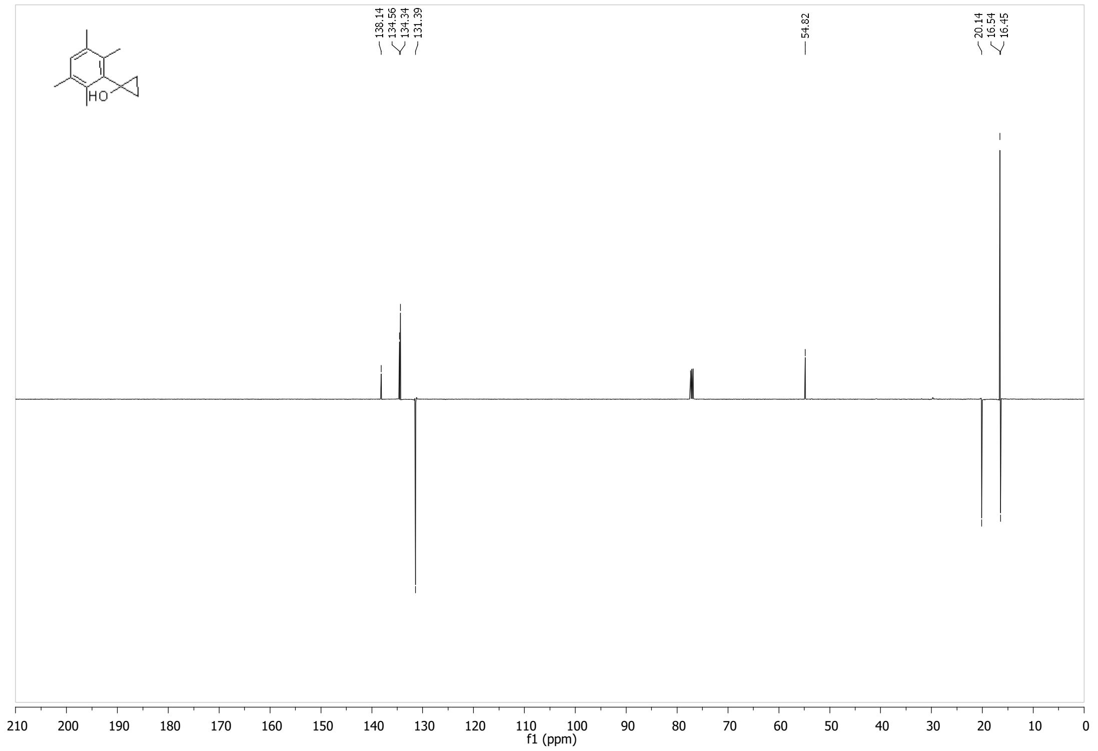

2-(Dimethyl(oxo)-λ⁶-sulfaneylidene)- 1-phenylethan-1-one 36.

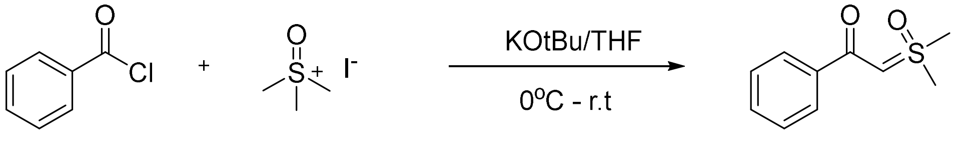

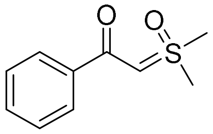

This compound has been reported and fully characterized. Zhu, S.; Shi, K.; Zhu, H.; Jia, Z.-K.; Xia, X.-F.; Wang, D.; Zou, L.-H., Copper-catalyzed annulation or homocoupling of sulfoxonium ylides: synthesis of 2, 3-diaroylquinolines or α, α, β-tricarbonyl sulfoxonium ylides. *Org Lett* **2020,** *22*, 1504-1509.

To a stirred solution of potassium tert-butoxide (1.28 mg, 11.4 mmol) in THF (2 mL) was added trimethylsulfoxonium iodide (1.89 mg, 8.55 mmol) at room temperature. The resulting mixture was refluxed for 2 h. Then the reaction mixture was cooled to 0 °C, followed by addition of benzoyl chloride (400 mg, 2.84 mmol) in THF (2 mL). The reaction was allowed to room temperature and stirred for overnight. After the solvent was evaporated, water (20 mL) and EtOAc (20 mL) were added to the residual crude product. The aqueous layer was separated and extracted with EtOAc (3 × 20 mL) and the organic layers were combined. The organic solution was dried over anhydrous Na_2SO_4 and evaporated under vacuum. The product was isolated via flash chromatography on silica eluted with 5% MeOH in EtOAc to give 2-(dimethyl(oxo)-λ^6-sulfaneylidene)- 1-phenylethan-1-one as a white solid (354 mg, 1.78 mmol, 64%). TLC: Rf ca 0.2 (9:1 EtOAc: MeOH), strong UV and PMA active; ^{1}H NMR (400 MHz, $CDCl_3$): δ 7.78 (2H, d, *J* 7.1, ArH), 7.47 – 7.29 (3H, m, ArH), 5.03 (1H, s, CH), 3.48 (6H, s, CH_3); $^{13}C\{^1H\}$ NMR (101 MHz, $CDCl_3$): δ 182.4 (C), 138.9 (C), 130.8 (CH), 128.2 (CH), 126.6 (CH), 68.5 (CH), 42.4 (CH_3); *m/z* (ES-API+) 219.0 (M^+ + Na, 100%). Data match that reported.

^{1}H NMR (400 MHz, $CDCl_3$) of 2-(dimethyl(oxo)-λ^6-sulfaneylidene)- 1-phenylethan-1-one **36**.

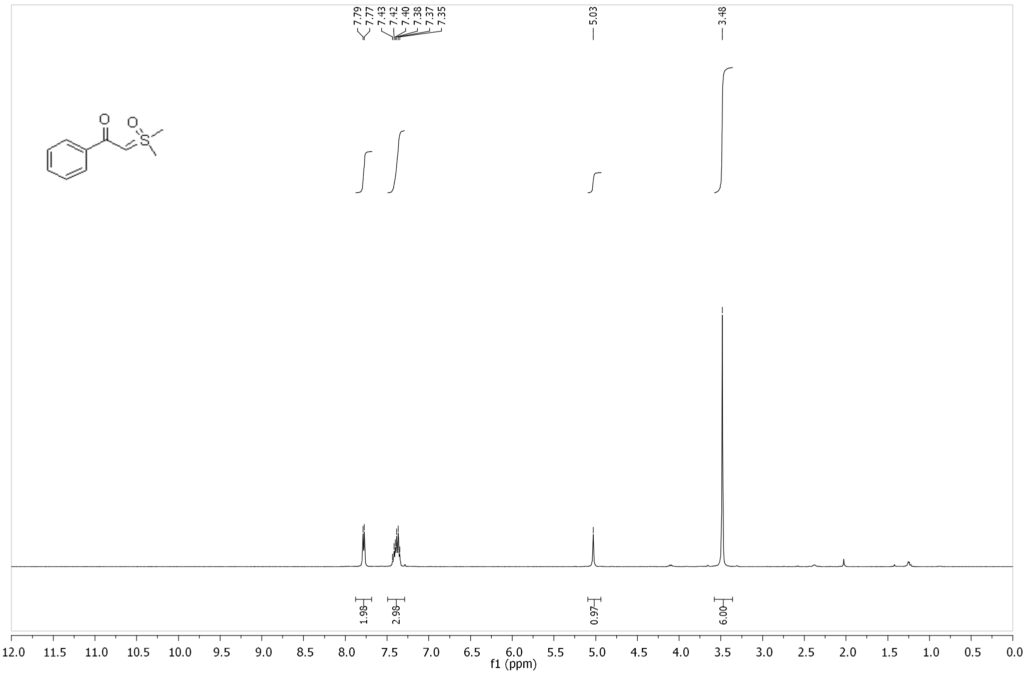

$^{13}C\{^1H\}$ NMR (101 MHz, $CDCl_3$) of 2-(dimethyl(oxo)-λ^6-sulfaneylidene)- 1-phenylethan-1-one **36**.

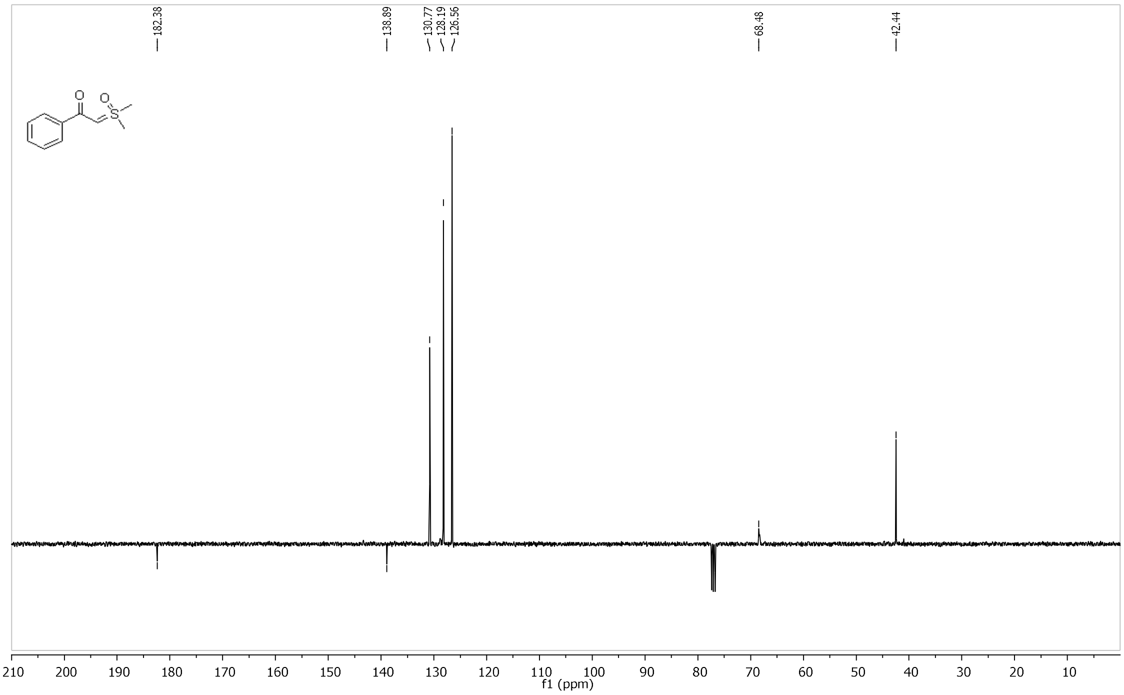

2-(Dimethyl(oxo)-λ^6-sulfaneylidene)-1-(4-methoxyphenyl)ethan-1-one) 37.

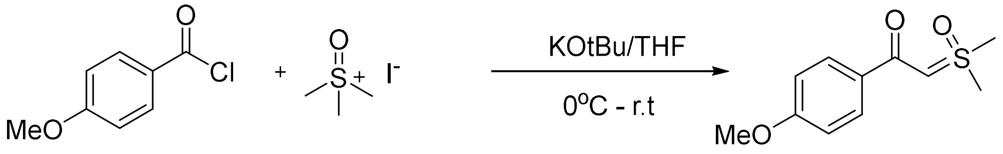

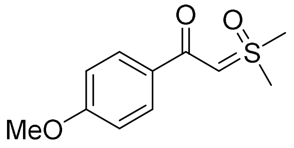

This compound has been reported and fully characterized. Liu, L.; Lin, J.; Pang, M.; Jin, H.; Yu, X.; Wang, S., Photo-Thermo-Mechanochemical Approach to Synthesize Quinolines via Addition/Cyclization of Sulfoxonium Ylides with 2-Vinylanilines Catalyzed by Iron (II) Phthalocyanine. *Org. Lett.* **2022,** *24*, 1146-1151.

To a stirred solution of potassium tert-butoxide (449 mg, 4.00 mmol) in THF (2 mL) was added trimethylsulfoxonium iodide (660 mg, 3.00 mmol) at room temperature. The resulting mixture was refluxed for 2 h. Then the reaction mixture was cooled to 0 °C, followed by addition of 4-methoxybenzoyl chloride (201 mg, 1.18 mmol) in THF (2 mL). The reaction was allowed to room temperature and stirred for overnight. After the solvent was evaporated, water (20 mL) and EtOAc (20 mL) were added to the residual crude product. The aqueous layer was separated and washed with EtOAc (3 × 20 mL) and the organic layers were combined. The organic solution was dried over anhydrous Na_2SO_4 and evaporated under vacuum. The product was isolated via flash chromatography on silica eluted with 5% MeOH in EtOAc to give 2-(dimethyl(oxo)-λ^6-sulfaneylidene)-1-(4-methoxyphenyl)ethan-1-one) **37** as a yellow solid (250 mg, 1.15 mmol, 97%); TLC: Rf ca 0.1 (9:1 EtOAc: MeOH), strong UV and PMA active; ^{1}H NMR (400 MHz, $CDCl_3$): δ 7.75 (1H, d, *J* 8.1, ArH), 6.99 – 6.70 (2H, m, ArH), 4.94 (1H, s, CH), 3.82 (3H, s, CH_3), 3.48 (6H, s, CH_3); $^{13}C\{^1H\}$ NMR (101 MHz, $CDCl_3$): δ 181.9 (C), 161.7 (C), 131.6 (C), 128.3 (CH), 113.4 (CH), 67.8 (CH), 55.3 (CH_3), 42.5 (CH_3); *m/z* (ES-API+) 227.0 (M^+ + Na, 100). The data matched that reported.

^{1}H NMR (400 MHz, $CDCl_3$) of 2-(dimethyl(oxo)-λ^6-sulfaneylidene)-1-(4-methoxyphenyl)ethan-1-one) **37**.

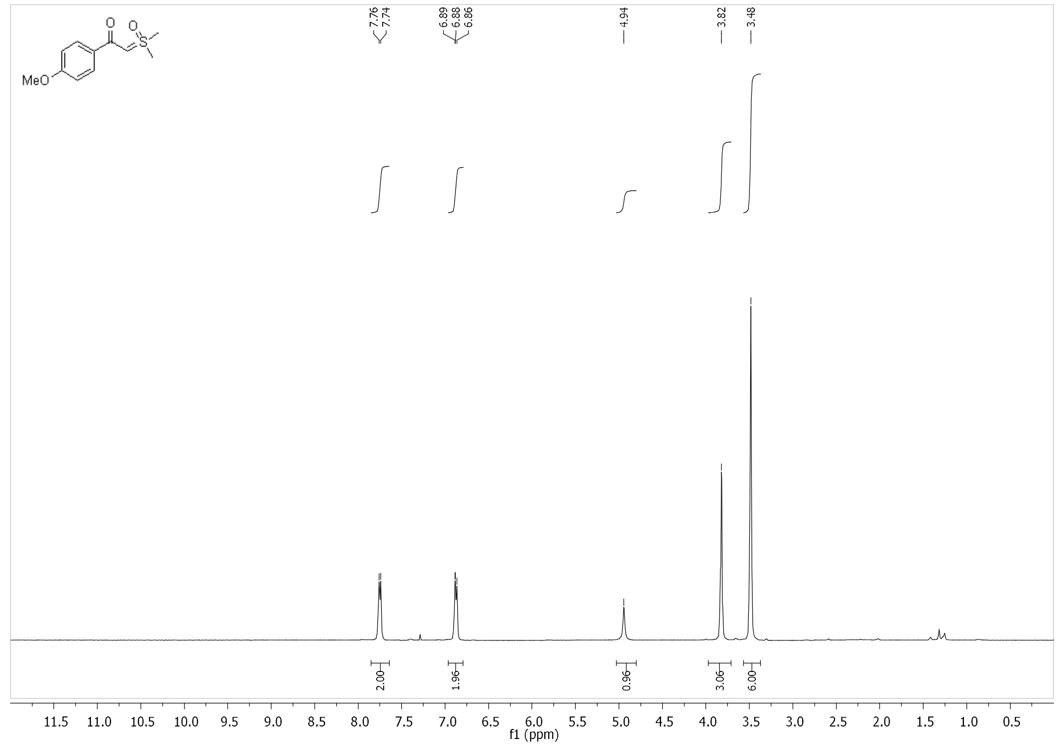

$^{13}C\{^1H\}$ NMR (101 MHz, $CDCl_3$) of 2-(dimethyl(oxo)-λ^6-sulfaneylidene)-1-(4-methoxyphenyl)ethan-1-one) **37**.

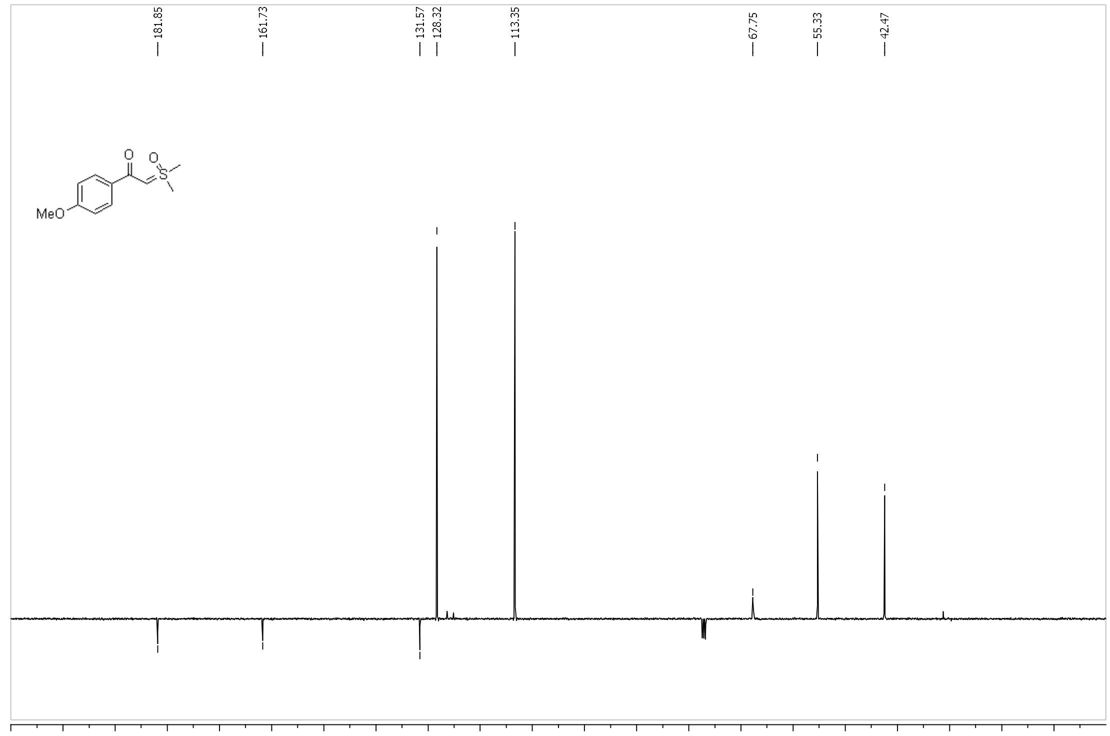

1-Phenyl-5-(2,3,5,6-tetramethylphenyl)pentane-1,5-dione 33a.

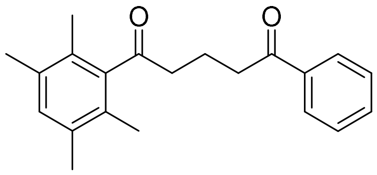

This compound is novel. In an oven-dried Schlenk tube, equipped with a magnetic stir bar, 1-(2,3,5,6-tetramethylphenyl)cyclopropan-1-ol **35** (95 mg, 0.50 mmol), 2-(dimethyl(oxo)-λ^6-sulfaneylidene)- 1-phenylethan-1-one (50 mg, 0.25 mmol), $[RuCl_2(p\text{-cymene})_2]_2$ (7.66 mg, 5 mol %) and trifluoroethanol (5 mL) were added. The tube was thoroughly flushed with argon, then the mixture was stirred for 144 h at 80 °C. After completion of the reaction, the solvent was removed under vacuum. The product was isolated via flash chromatography on silica eluted with 0-10% EtOAc in hexane to give 1-phenyl-5-(2,3,5,6-tetramethylphenyl)pentane-1,5-dione **33a** as a white solid (35.7 mg, 0.116 mmol, 45%). TLC: Rf ca 0.24 (9:1 hexane: EtOAc), strong UV and PMA active; Mp: 80.5 °C; HRMS (ESI+) *m/z*: [M+Na]+ Calcd for $C_{21}H_{24}NaO_2$ 331.1669; Found 331.1660; 2.4 ppm error; ν_{max} 3068, 2958, 2923, 1685, 1597 cm^{-1}; ^1H NMR (400 MHz, $CDCl_3$): δ 7.98 (2H, d, *J* 7.5, ArH), 7.57 (1H, t, *J* 7.2, ArH), 7.47 (2H, t, *J* 7.6, ArH), 6.94 (1H, s, ArH), 3.14 (2H, t, *J* 7.0, CH_2), 2.82 (2H, t, *J* 7.0, CH_2), 2.23 – 2.13 (8H, m, CH_3+CH_2), 2.05 (6H, s, CH_3); $^{13}C\{^1H\}$ NMR (101 MHz, $CDCl_3$): δ 211.4 (C), 199.8 (C), 142.7 (C), 136.9 (C), 134.4 (C), 133.1 (CH), 131.6 (CH), 128.6 (CH), 128.1 (CH), 127.9 (C), 44.2 (CH_2), 37.4 (CH_2), 19.5 (CH_3), 17.9 (CH_2), 15.9 (CH_3); *m/z* (ES-API+) 331.2 (M^+ + Na, 100%); Enantiomeric excess and conversion determined by HPLC analysis (Chiralpak OD-H, 30 cm x 6 mm column, hexane:iPrOH 90:10, 1.0 mL/min, T = 25°C) ketone 7.6 min, *R* and *S* isomer 9.1 min and 10.9 min.

^1H NMR (400 MHz, $CDCl_3$) of 1-Phenyl-5-(2,3,5,6-tetramethylphenyl)pentane-1,5-dione **33a**.

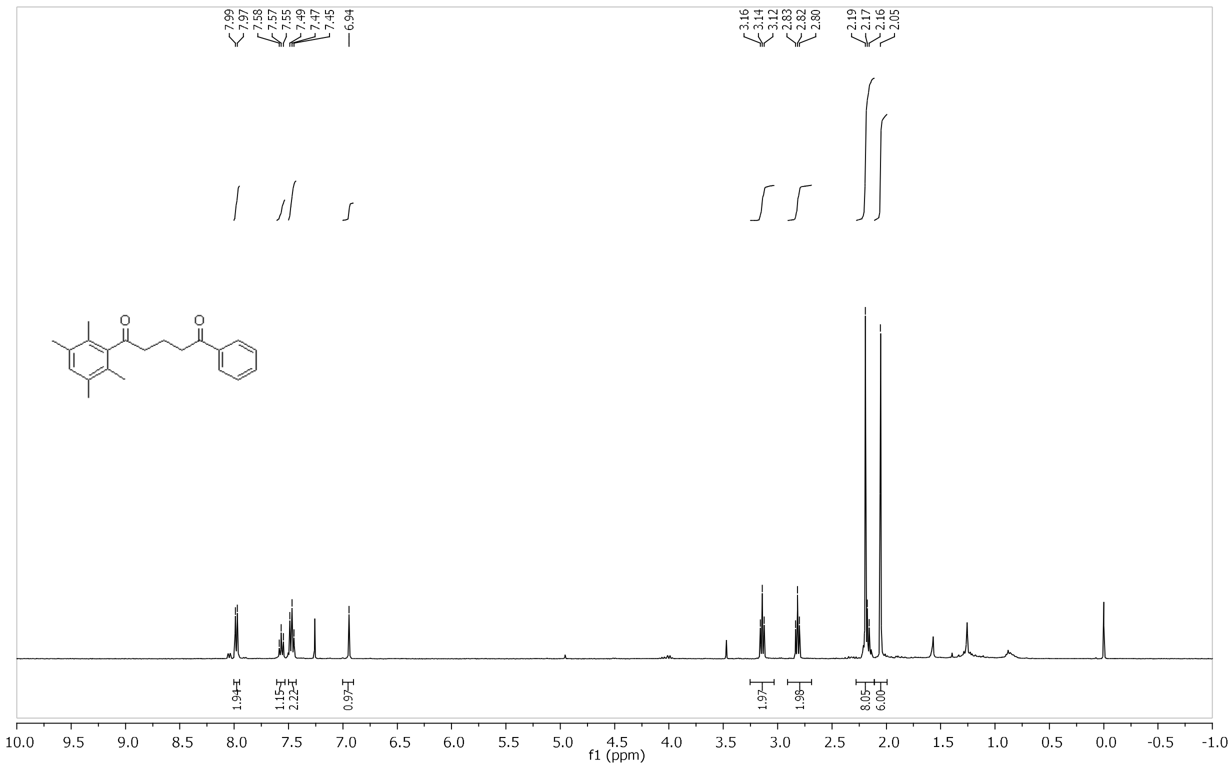

COSY (400 MHz, $CDCl_3$) of 1-Phenyl-5-(2,3,5,6-tetramethylphenyl)pentane-1,5-dione **33a**.

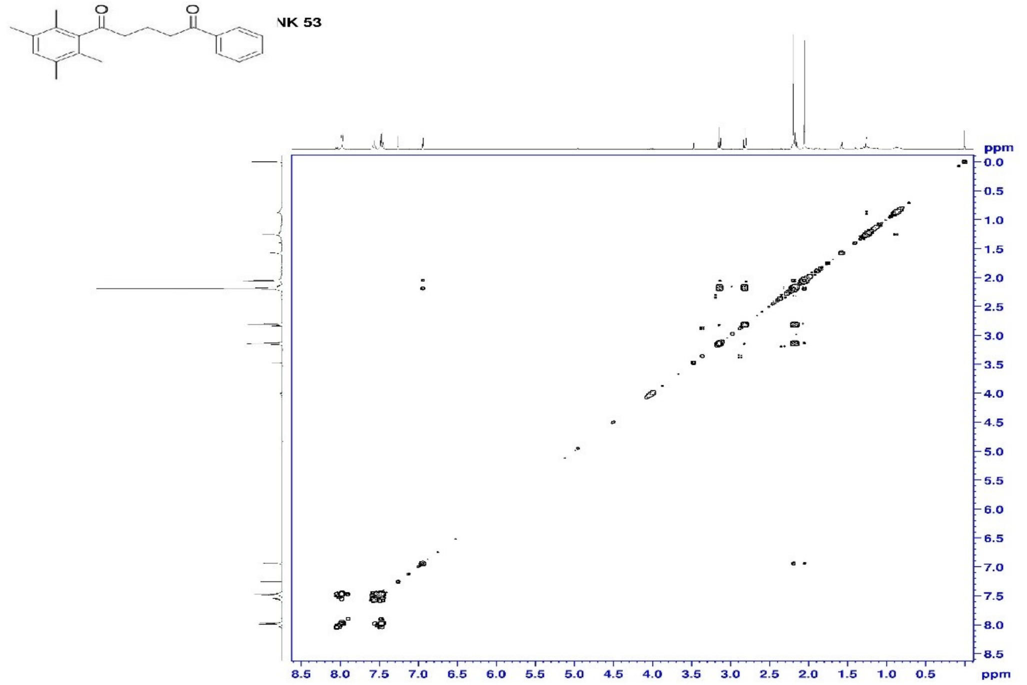

HSQC (101 MHz, $CDCl_3$) of 1-Phenyl-5-(2,3,5,6-tetramethylphenyl)pentane-1,5-dione **33a**.

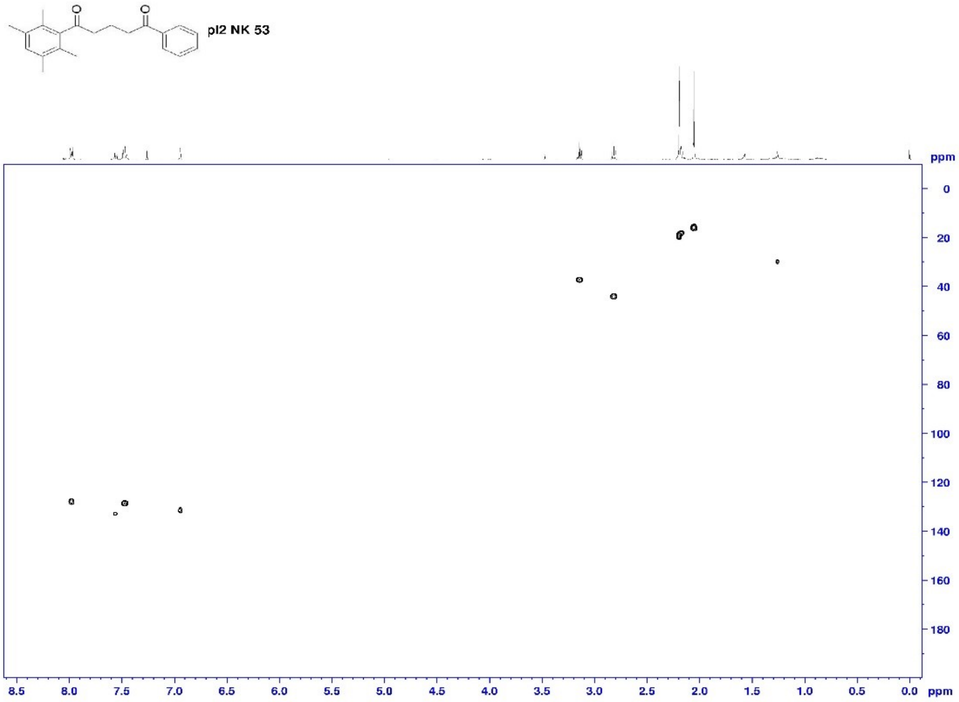

HMBC (101 MHz, $CDCl_3$) of 1-Phenyl-5-(2,3,5,6-tetramethylphenyl)pentane-1,5-dione **33a**.

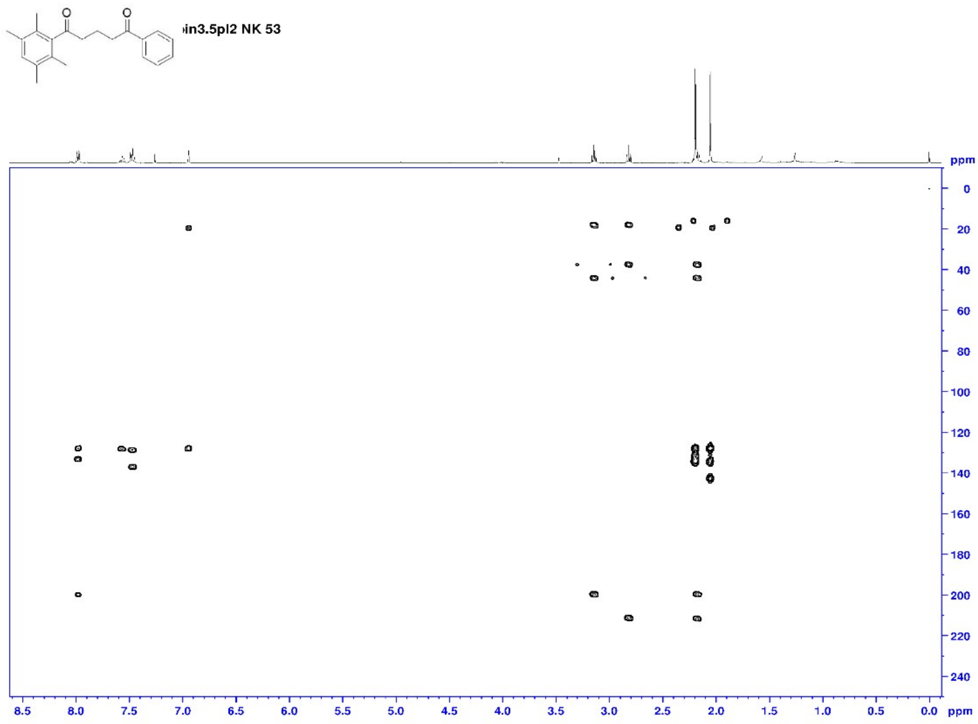

$^{13}C\{^{1}H\}$ NMR (101 MHz, $CDCl_3$) of 1-Phenyl-5-(2,3,5,6-tetramethylphenyl)pentane-1,5-dione **33a**.

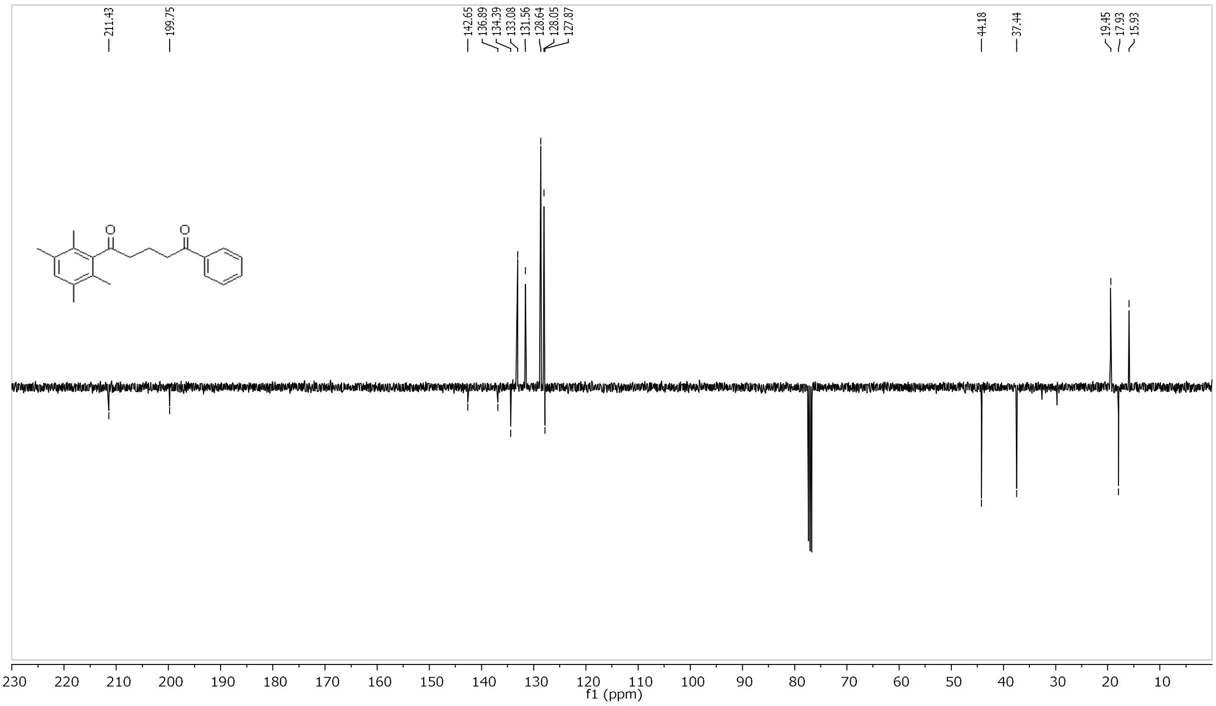

HPLC of 1-Phenyl-5-(2,3,5,6-tetramethylphenyl)pentane-1,5-dione **33a**.

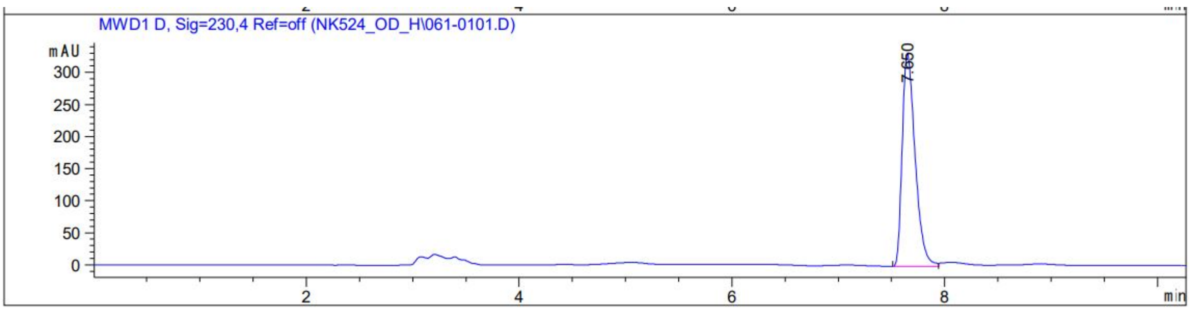

Signal 4: MWD1 D, Sig=230,4 Ref=off

Peak #	RetTime [min]	Type	Width [min]	Area [mAU*s]	Height [mAU]	Area %
1	7.650	BV	0.1282	2798.81396	332.35699	100.0000

Totals :				2798.81396	332.35699	

5-Hydroxy-5-phenyl-1-(2,3,5,6-tetramethylphenyl)pentan-1-one 33b.

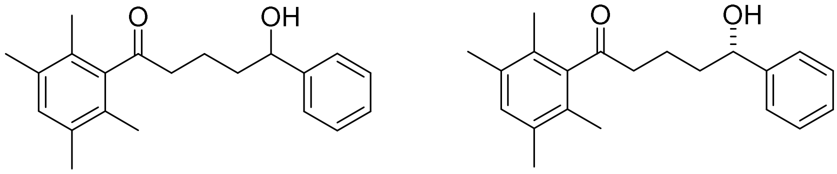

This compound is novel. **Synthesis of a racemic standard:** (*R,R*)-3C-Tethered Ru(II)-TsDPEN catalyst (0.16 mg, 0.00026 mmol, 0.5 mol%) and (*S,S*)-3C-tethered Ru(II)-TsDPEN catalyst (0.16 mg, 0.00026 mmol, 0.5 mol%) were added to FA: TEA (5:2 azeotropic mixture, 0.03mL) at rt and the mixture was stirred under a nitrogen atmosphere for 15 minutes; after which 1-phenyl-5-(2,3,5,6-tetramethylphenyl)pentane-1,5-dione **33a** (16.5 mg, 0.053 mmol) was added in DCM (0.13 mL). The reaction mixture was stirred under a nitrogen atmosphere and followed by TLC (5:1 hexane: EtOAc). After 48 h, the reaction was quenched using saturated $NaHCO_3$ solution (20 mL). EtOAc (20 mL) was added and the organic layer was separated. The aqueous layer was extracted with EtOAc (3 x 20 mL) and the combined organic layers were dried ($MgSO_4$) and filtered. The solvent was removed to give the crude product. The product was isolated via flash chromatography on silica eluted with 0-20% EtOAc in petroleum ether to give 5-hydroxy-5-phenyl-1-(2,3,5,6-tetramethylphenyl)pentan-1-one **33b** as a white solid (9.1 mg, 0.030 mmol, 54.8%); TLC: Rf ca 0.25 (4:1 hexane: EtOAc), strong UV and PMA; Mp: 83 °C; ν_{max} 3528, 2964, 2915, 1601 cm^{-1}; HRMS (ESI+) *m/z*: [M+Na]+ Calcd for $C_{21}H_{26}NaO_2$ 333.1825; Found 333.1819; 1.9 ppm error; ^{1}H NMR (500 MHz, $CDCl_3$): δ 7.38 (3H, m, ArH), 7.34 – 7.24 (2H, m, ArH), 6.97 (1H, s, ArH), 4.76 (1H, br. s, CH), 2.75-2.70 (2H, m, CH_2), 2.22 (6H, s, CH_3), 2.05 (6H, s, CH_3), 2.02 (1H, br. s, OH), 1.95 – 1.70 (4H, m, 2 x CH_2); $^{13}C\{^{1}H\}$ NMR (126 MHz, $CDCl_3$): δ 211.5 (C), 144.6 (C), 142.7 (C), 134.4 (C), 131.5 (CH), 128.6 (CH), 127.9 (C), 127.7 (CH), 125.9 (CH), 74.5 (CH_2), 45.0 (CH_3), 38.4 (CH_3), 19.5 (CH_3), 19.4 (CH_2), 16.0 (CH_2); *m/z* (ES-API+) 333.2 (M^+ + Na, 100%); Enantiomeric excess and conversion determined by HPLC analysis (Chiralpak OD-H, 30 cm x 6 mm column, hexane:iPrOH 90:10, 1.0 mL/min, T = 25°C) ketone 7.6 min, *R* and *S* isomer 9.1 min and 10.9 min.

(*S*)-5-Hydroxy-5-phenyl-1-(2,3,5,6-tetramethylphenyl)pentan-1-one **33b**..

(*S,S*)-3C-Tethered Ru(II)-TsDPEN catalyst (0.71 mg, 0.0011 mmol, 1 mol%) was added to FA: TEA (5:2 azeotropic mixture, 0.06 mL) at rt and the mixture was stirred under a nitrogen atmosphere for 10-15 minutes; after which 1-phenyl-5-(2,3,5,6-tetramethylphenyl)pentane-1,5-dione **33a** (35.0 mg, 0.114 mmol) was added in DCM (1 mL). The reaction mixture was stirred under a nitrogen atmosphere for 48 h. The reaction was followed by TLC (9:1 hexane: EtOAc). After 48 h, the reaction was quenched using saturated $NaHCO_3$ solution (20 mL). EtOAc (20 mL) was added and the organic layer was separated. The aqueous layer was extracted with EtOAc (3 x 20 mL) and the combined organic layers were dried ($MgSO_4$) and filtered. The solvent was removed to give the crude product. The product was isolated via flash chromatography on silica eluted with 0-50% EtOAc in petroleum ether to give (*S*)-5-hydroxy-5-phenyl-1-(2,3,5,6-tetramethylphenyl)pentan-1-one **33b** as a white solid (16.7 mg, 0.054 mmol, 47%). The reaction was also followed by HPLC (Chiralpak OD-H, 30 cm x 6 mm column, hexane:iPrOH 90:10, 1.0 mL/min, T = 25 °C); $[\alpha]_D^{29}$ + 36.4 (c 0.02 in $CHCl_3$); (after 48 h, 100% conversion, 93% ee (*S*)).

^{1}H NMR (500 MHz, $CDCl_3$) of (*S*)-5-hydroxy-5-phenyl-1-(2,3,5,6-tetramethylphenyl)pentan-1-one **33b**.

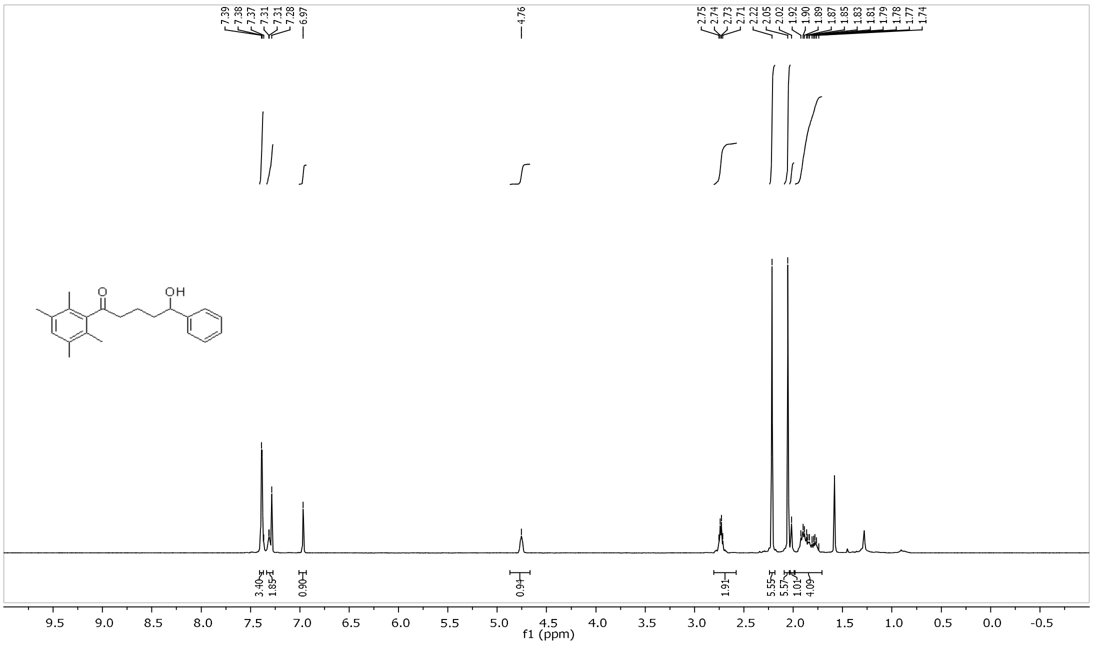

COSY (500 MHz, $CDCl_3$) of (*S*)-5-hydroxy-5-phenyl-1-(2,3,5,6-tetramethylphenyl)pentan-1-one **33b**.

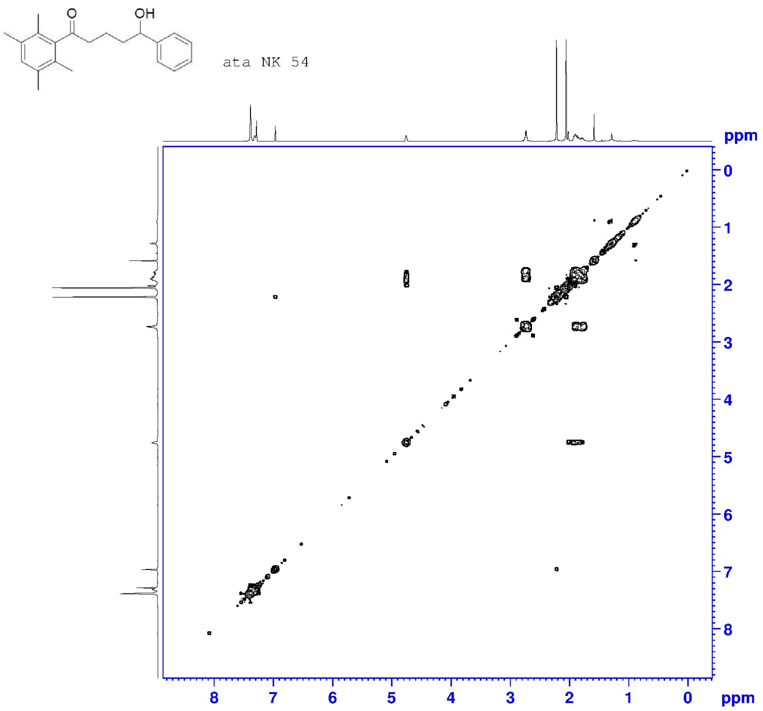

HSQC (126 MHz, $CDCl_3$) of (*S*)-5-hydroxy-5-phenyl-1-(2,3,5,6-tetramethylphenyl)pentan-1-one **33b**.

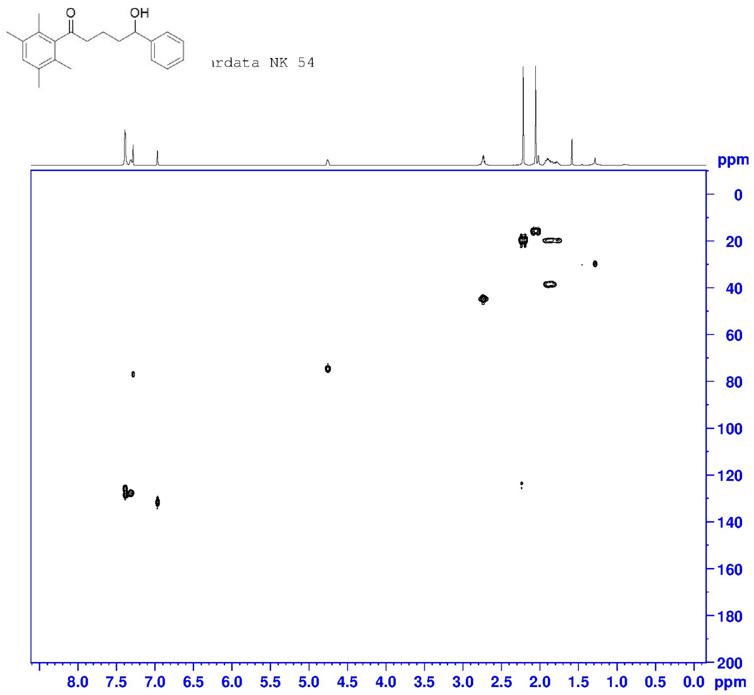

HMBC (126 MHz, $CDCl_3$) of (*S*)-5-hydroxy-5-phenyl-1-(2,3,5,6-tetramethylphenyl)pentan-1-one **33b**.

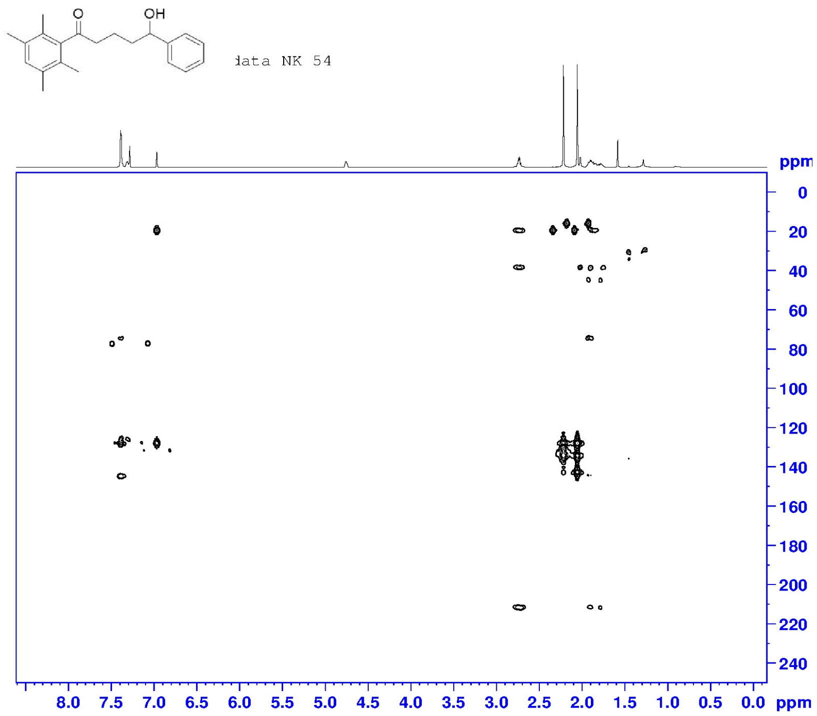

$^{13}C\{^1H\}$ NMR (126 MHz, $CDCl_3$) of (*S*)-5-hydroxy-5-phenyl-1-(2,3,5,6-tetramethylphenyl)pentan-1-one **33b**.

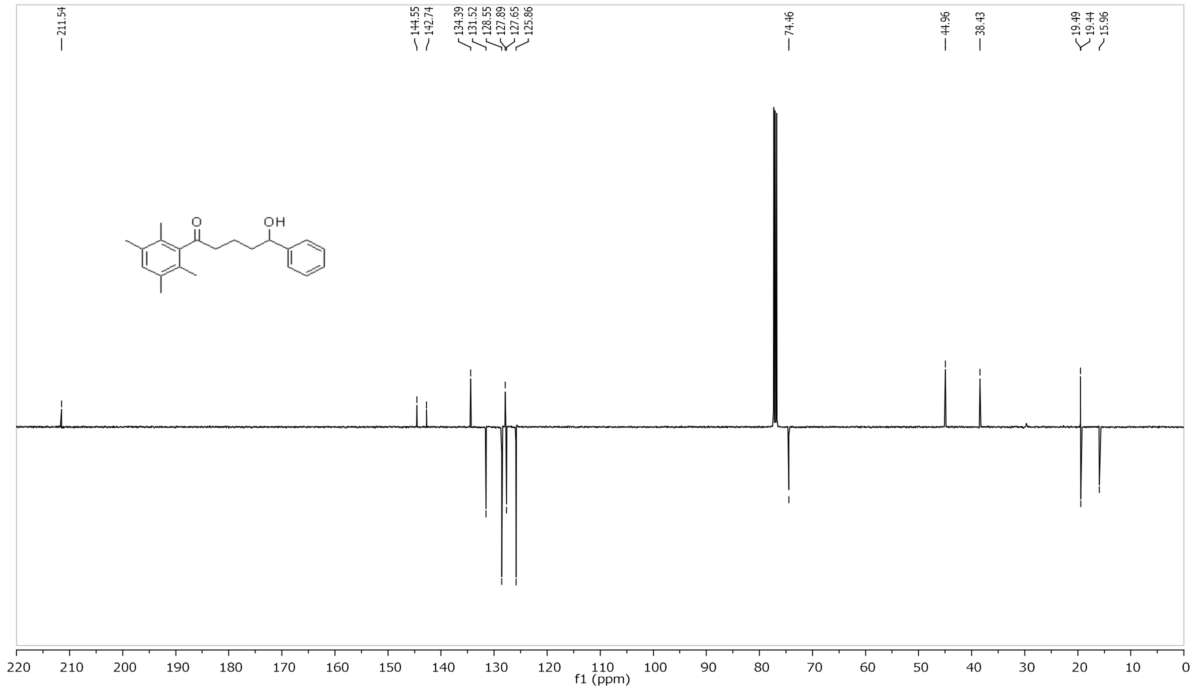

HPLC of racemic (5-hydroxy-5-phenyl-1-(2,3,5,6-tetramethylphenyl)pentan-1-one **33b**.

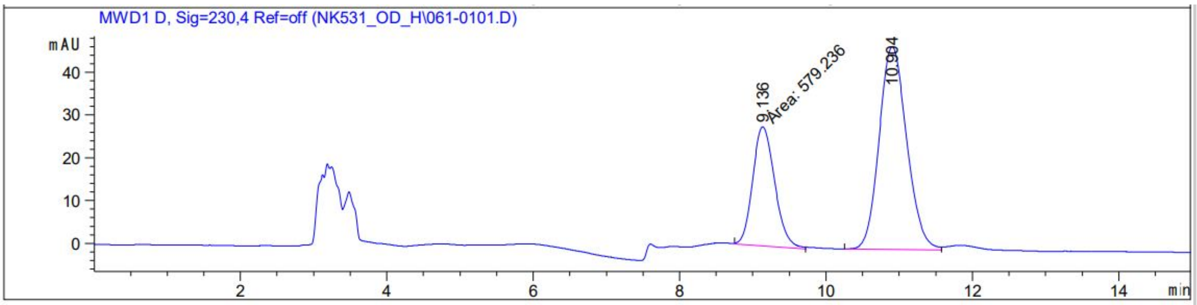

Signal 4: MWD1 D, Sig=230,4 Ref=off

Peak #	RetTime [min]	Type	Width [min]	Area [mAU*s]	Height [mAU]	Area %
1	9.136	MM	0.3474	579.23633	27.79041	31.7883
2	10.904	BV	0.4078	1242.93457	47.42434	68.2117
Totals :				1822.17090	75.21474	

HPLC of (*S*)-5-hydroxy-5-phenyl-1-(2,3,5,6-tetramethylphenyl)pentan-1-one **33b**. (*S,S*)-3C-tethered Ru(II)-TsDPEN catalyst (after 48 h, 100% conversion, 93% ee (*S*)).

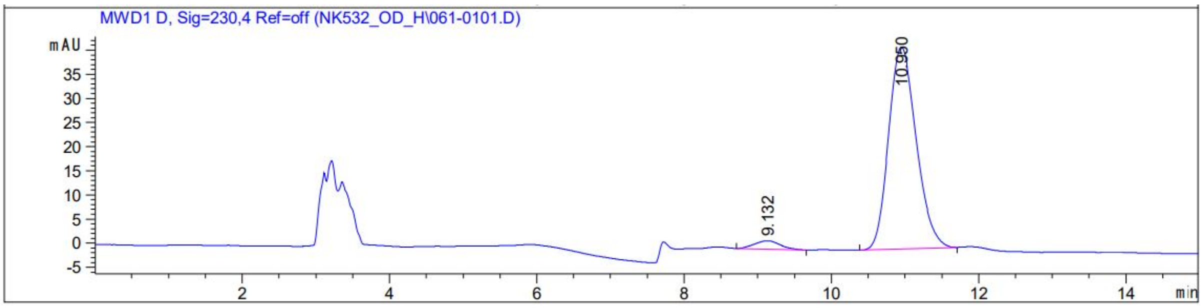

Signal 4: MWD1 D, Sig=230,4 Ref=off

Peak #	RetTime [min]	Type	Width [min]	Area [mAU*s]	Height [mAU]	Area %
1	9.132	BB	0.3448	42.01260	1.73290	3.7126
2	10.950	BB	0.4058	1089.59937	41.84201	96.2874
Totals :				1131.61196	43.57491	

1-(4-Methoxyphenyl)-5-(2,3,5,6-tetramethylphenyl)pentane-1,5-dione 34a.

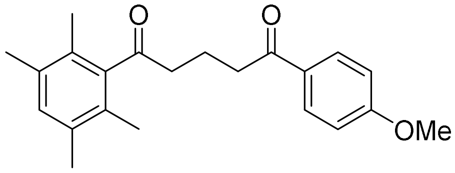

This compound is novel. In an oven-dried Schlenk tube, equipped with a magnetic stirrer bar, 1-(2,3,5,6-tetramethylphenyl)cyclopropan-1-ol **35** (126 mg, 0.66 mmol), 2-(dimethyl(oxo)-λ^6-sulfaneylidene)-1-(4-methoxyphenyl)ethan-1-one) (50 mg, 0.22 mmol), $[RuCl_2(p\text{-cymene})_2]_2$ (6.8 mg, 5 mol %) and 4.5 mL trifluoroethanol were added. The tube was thoroughly flushed with argon, then the mixture was stirred for 144 h at 80 °C. After completion of the reaction, the solvent was removed under vacuum, and the product was isolated via flash chromatography on silica eluted with 0-20% EtOAc in petroleum ether to give 1-(4-methoxyphenyl)-5-(2,3,5,6-tetramethylphenyl)pentane-1,5-dione **34a** as a yellow solid (32.6 mg, 0.965 mmol, 44%). TLC: Rf ca 0.17 (9:1 hexane: EtOAc), strong UV and PMA active; Mp: 70.9 °C; HRMS (ESI+) *m/z*: [M+Na]+ Calcd for $C_{22}H_{26}NaO_3$ 361.1774; Found 361.1766; 2.2 ppm error; ν_{max} 3007, 2955, 2922, 1688 cm^{-1}; ^{1}H NMR (400 MHz, $CDCl_3$): δ 7.97 (2H, d, *J* 8.8, ArH), 6.98 – 6.89 (3H, m, ArH), 3.87 (3H, s, OCH_3), 3.08 (2H, t, *J* 7.1, CH_2), 2.81 (2H, t, *J* 7.0, CH_2), 2.23 – 2.10 (8H, m, CH_3+CH_2), 2.05 (6H, s, CH_3); $^{13}C\{^1H\}$ NMR (101 MHz, $CDCl_3$): δ 211.5 (C), 198.3 (C), 163.5 (C), 142.7 (C), 134.4 (C), 131.5 (CH), 130.3 (CH), 130.0 (C), 127.9 (C), 113.8 (CH), 55.5 (CH_3), 44.3 (CH_2), 37.1 (CH_2), 19.5 (CH_3), 18.2 (CH_2), 15.9 (CH_3); *m/z* (ES-API+) 361.2 (M^+ + Na, 100%); Enantiomeric excess and conversion determined by HPLC analysis (Chiralpak OD-H, 30 cm x 6 mm column, hexane:iPrOH 90:10, 1.0 mL/min, T = 25°C) ketone 10.6 min, *R* and *S* isomer 12.9min and 15.3 min.

^{1}H NMR (400 MHz, $CDCl_3$) of 1-(4-methoxyphenyl)-5-(2,3,5,6-tetramethylphenyl)pentane-1,5-dione **34a**.

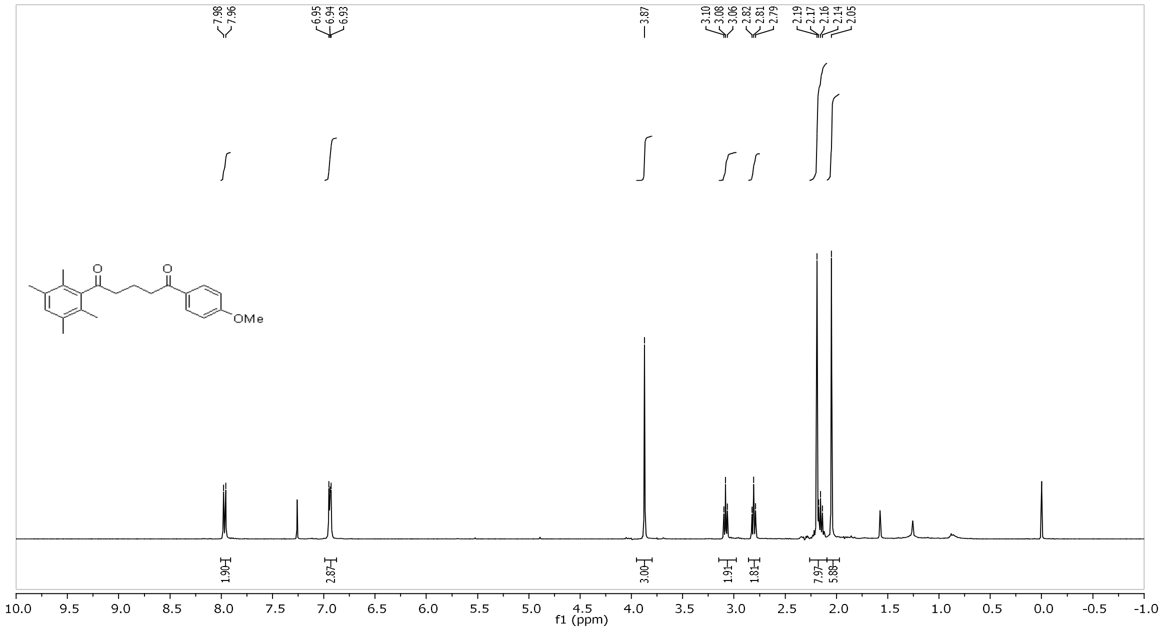

COSY (400 MHz, $CDCl_3$) of 1-(4-methoxyphenyl)-5-(2,3,5,6-tetramethylphenyl)pentane-1,5-dione **34a**.

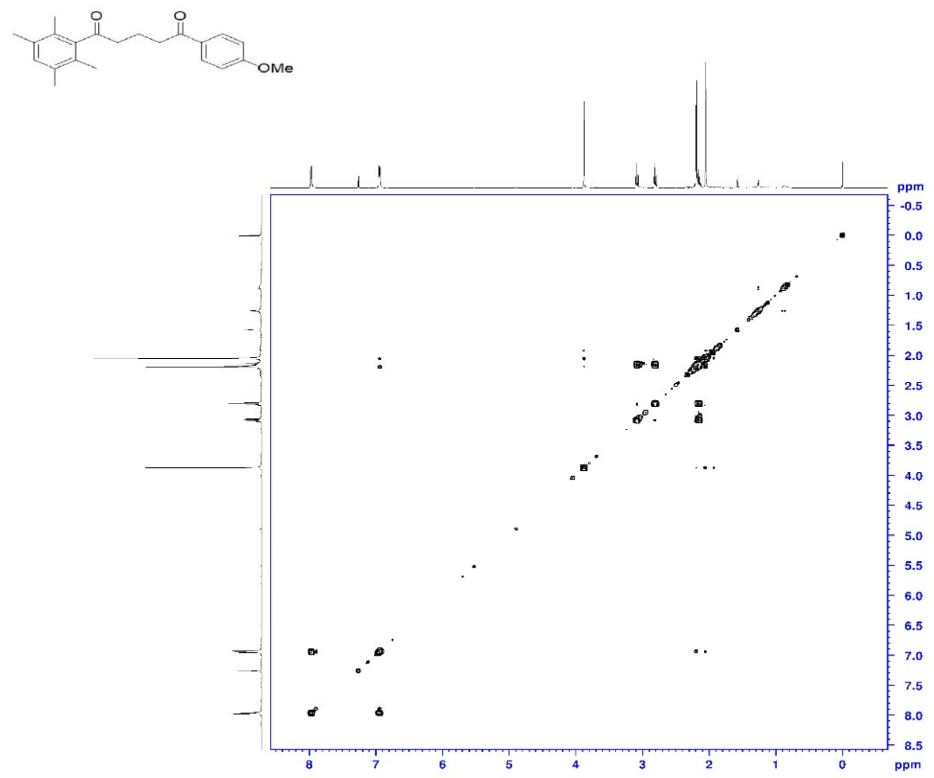

HSQC (101 MHz, $CDCl_3$) of 1-(4-methoxyphenyl)-5-(2,3,5,6-tetramethylphenyl)pentane-1,5-dione **34a**.

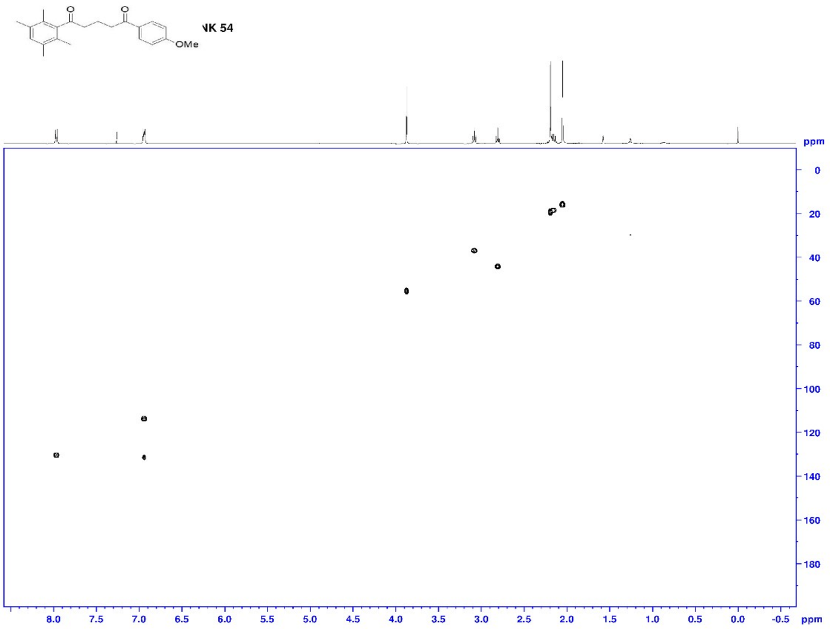

HMBC (101 MHz, $CDCl_3$) of 1-(4-methoxyphenyl)-5-(2,3,5,6-tetramethylphenyl)pentane-1,5-dione **34a**.

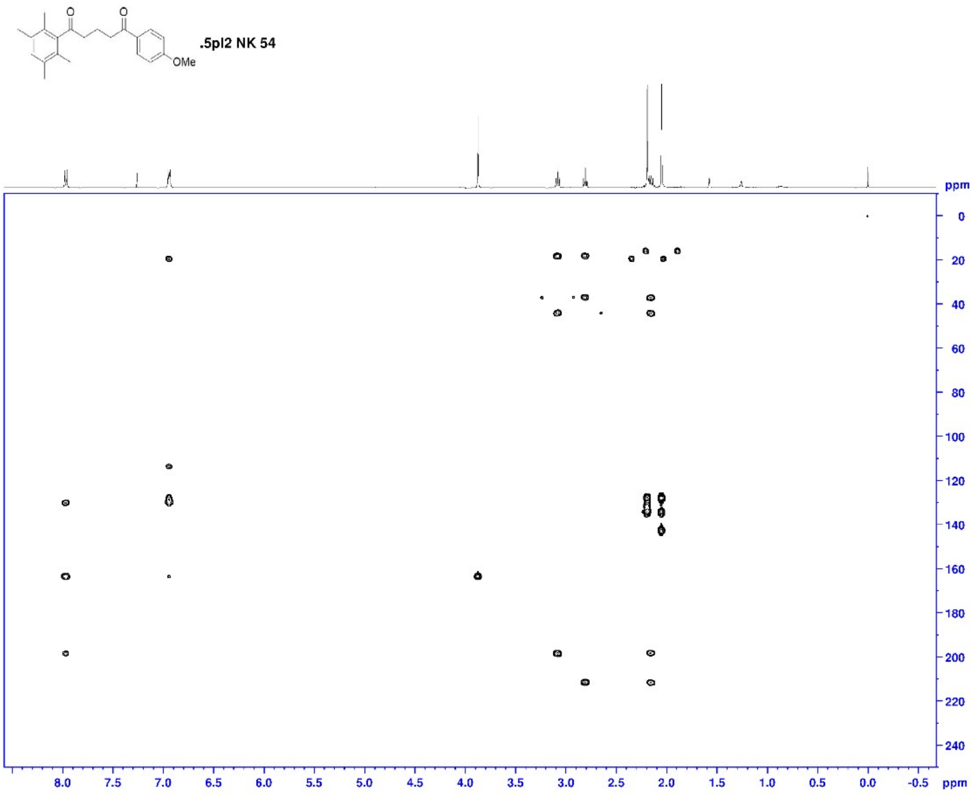

$^{13}C\{^1H\}$ NMR (101 MHz, $CDCl_3$) of 1-(4-methoxyphenyl)-5-(2,3,5,6-tetramethylphenyl)pentane-1,5-dione **34a**.

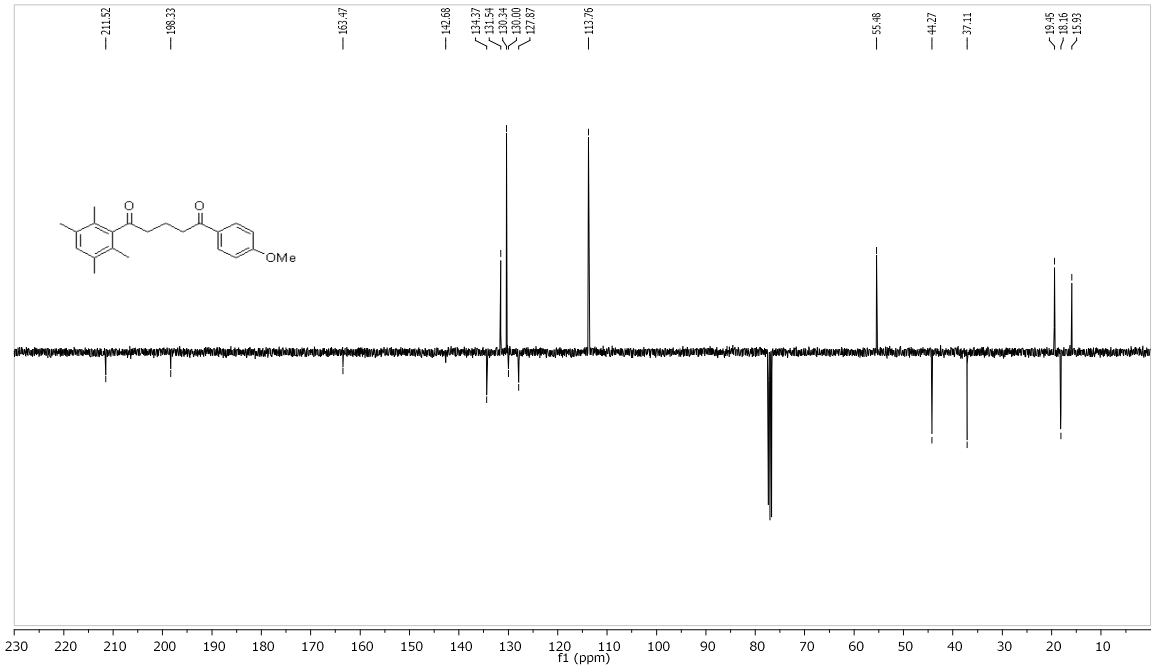

HPLC of 1-(4-methoxyphenyl)-5-(2,3,5,6-tetramethylphenyl)pentane-1,5-dione **34a**.

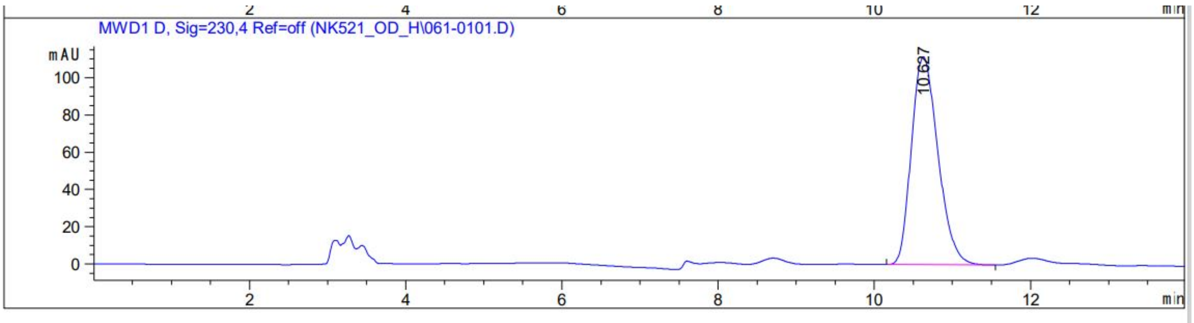

Signal 4: MWD1 D, Sig=230,4 Ref=off

Peak #	RetTime [min]	Type	Width [min]	Area [mAU*s]	Height [mAU]	Area %
1	10.627	BB	0.3701	2641.38037	111.61935	100.0000

Totals : 2641.38037 111.61935

5-Hydroxy-5-(4-methoxyphenyl)-1-(2,3,5,6-tetramethylphenyl)pentan-1-one 34b.

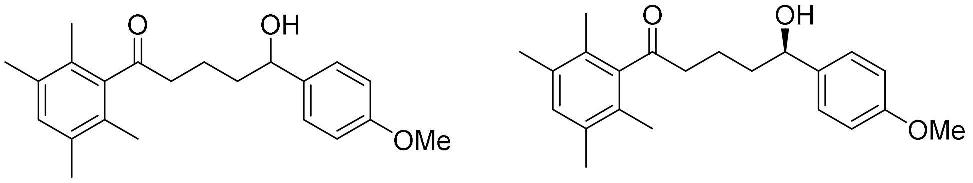

This compound is novel.

Synthesis of a racemic standard: (*R,R*)-3C-Tethered Ru(II)-TsDPEN catalyst (0.12 mg, 0.00019 mmol, 0.5 mol%) and (*S,S*)-3C-tethered Ru(II)-TsDPEN catalyst (0.12 mg, 0.00019 mmol, 0.5 mol%) were added to FA: TEA (5:2 azeotropic mixture, 0.02 mL) at rt and the mixture was stirred under a nitrogen atmosphere for 15 minutes; after which 1-(4-methoxyphenyl)-5-(2,3,5,6-tetramethylphenyl)pentane-1,5-dione **34a** (12.5 mg, 0.037 mmol) was added in DCM (1 mL). The reaction mixture was stirred under a nitrogen atmosphere and followed by TLC (5:1 hexane: EtOAc). After 48 h, the reaction was quenched using saturated $NaHCO_3$ solution (20 mL). EtOAc (20 mL) was added, and the organic layer was separated. The aqueous layer was extracted with EtOAc (3 x 20 mL) and the combined organic layers were dried ($MgSO_4$) and filtered. The solvent was removed to give the crude product. The product was isolated via flash chromatography on silica eluted with 0-50% EtOAc in petroleum ether to give 5-hydroxy-5-(4-methoxyphenyl)-1-(2,3,5,6-tetramethylphenyl)pentan-1-one **34b** as a yellow solid (10.8 mg, 0.032 mmol, 86%); ν_{max} 3533, 2963, 2925, 2854, 1688 cm^{-1}; TLC: Rf ca 0.1 (4:1 hexane: EtOAc), PMA active; Mp: 104 °C; HRMS (ESI+) *m/z*: [M+Na]+ Calcd for $C_{22}H_{28}NaO_3$ 363.1931; Found 363.1925; 1.7 ppm error; ^1H NMR (500 MHz, $CDCl_3$): δ 7.29 (2H, d, *J* 8.6, ArH), 6.94 (1H, s, ArH), 6.89 (2H, d, *J* 8.7, ArH), 4.73 – 4.63 (1H, m, ArCH), 3.81 (3H, s, CH_3), 2.78 – 2.60 (2H, m, CH_2), 2.19 (6H, s, CH_3), 2.03 (6H, s, CH_3), 1.91 (1H, br.s, OH), 1.89 – 1.66 (4H, m, 2 x CH_2); $^{13}C\{^1H\}$ NMR (126 MHz, $CDCl_3$) 211.6 (C), 159.1 (C), 142.8 (C), 136.7 (C), 134.4 (C), 131.5 (CH), 127.9 (C), 127.1 (CH), 113.9 (CH), 74.1 (CH), 55.3 (CH_2), 45.0 (CH_3), 38.3 (CH_3), 19.5 (CH_3), 19.4 (CH_2), 16.0 (CH_2); *m/z* (ES-API+) 363.2 (M^+ + Na, 100%); Enantiomeric excess and conversion determined by HPLC analysis (Chiralpak OD-H, 30 cm x 6 mm column, hexane:iPrOH 90:10, 1.0 mL/min, T = 25°C) ketone 10.6 min, *R* and *S* isomer 12.9 min and 15.3 min.

(*R*)-5-Hydroxy-5-(4-methoxyphenyl)-1-(2,3,5,6-tetramethylphenyl)pentan-1-one **34b**. (*R,R*)-3C-tethered Ru(II)-TsDPEN catalyst (0.37 mg, 0.00060 mmol, 1 mol%) was added to FA: TEA (5:2 azeotropic mixture, 0.03 mL) at rt and the mixture was stirred under a nitrogen atmosphere for 10-15 minutes; after which 1-(4-methoxyphenyl)-5-(2,3,5,6-tetramethylphenyl)pentane-1,5-dione **34a** (20.0 mg, 0.059 mmol) was added in DCM (1 mL). The reaction mixture was stirred under a nitrogen atmosphere for 48 h. The reaction was followed by TLC (9:1 hexane: EtOAc). After 48 h, the reaction was quenched using saturated $NaHCO_3$ solution (20 mL). EtOAc (20 mL) was added and the organic layer was separated. The aqueous layer was extracted with EtOAc (3 x 20 mL) and the combined organic layers were dried ($MgSO_4$) and filtered. The solvent was removed to give the crude product. The product was isolated via flash chromatography on silica eluted with 0-50% EtOAc in petroleum ether to give (*R*)-5-hydroxy-5-(4-methoxyphenyl)-1-(2,3,5,6-tetramethylphenyl)pentan-1-one **34b** as a yellow solid (6.6 mg, 0.019 mmol, 33%). The reaction was also followed by HPLC (Chiralpak OD-H, 30 cm x 6 mm column, hexane:iPrOH 90:10, 1.0 mL/min, T = 25°C); $[\alpha]_D^{29}$ + 17.17 (c 0.132 in $CHCl_3$); (after 48 h, 100% conversion, 93% ee (*R*)).

^1H NMR (500 MHz, $CDCl_3$) of 5-hydroxy-5-(4-methoxyphenyl)-1-(2,3,5,6-tetramethylphenyl)pentan-1-one **34b**.

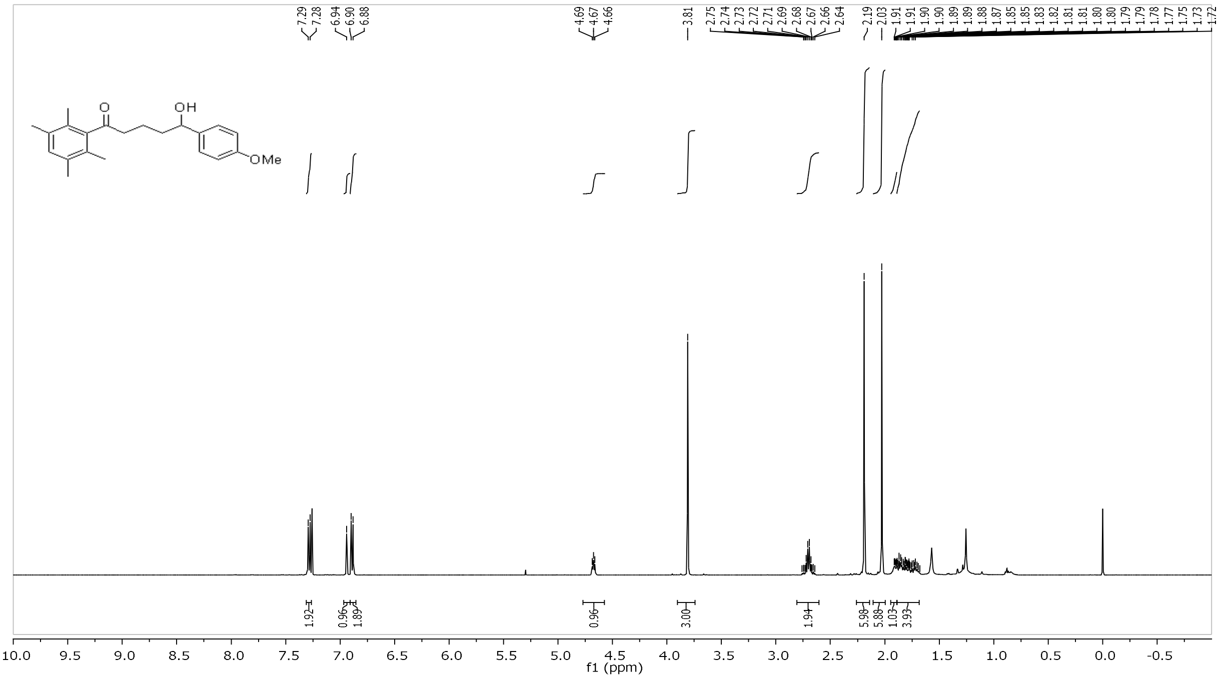

COSY (500 MHz, $CDCl_3$) of 5-hydroxy-5-(4-methoxyphenyl)-1-(2,3,5,6-tetramethylphenyl)pentan-1-one **34b.**

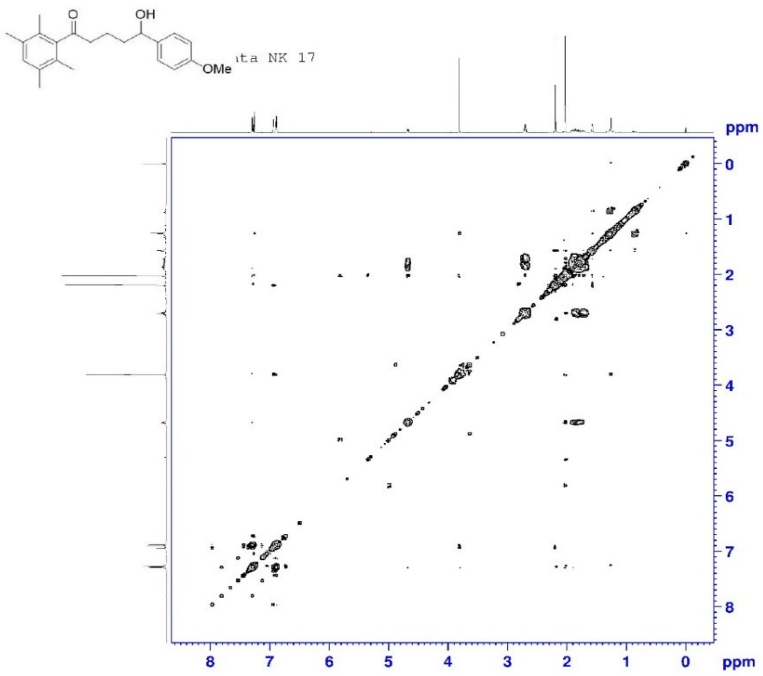

HSQC (126 MHz, $CDCl_3$) of 5-hydroxy-5-(4-methoxyphenyl)-1-(2,3,5,6-tetramethylphenyl)pentan-1-one **34b.**

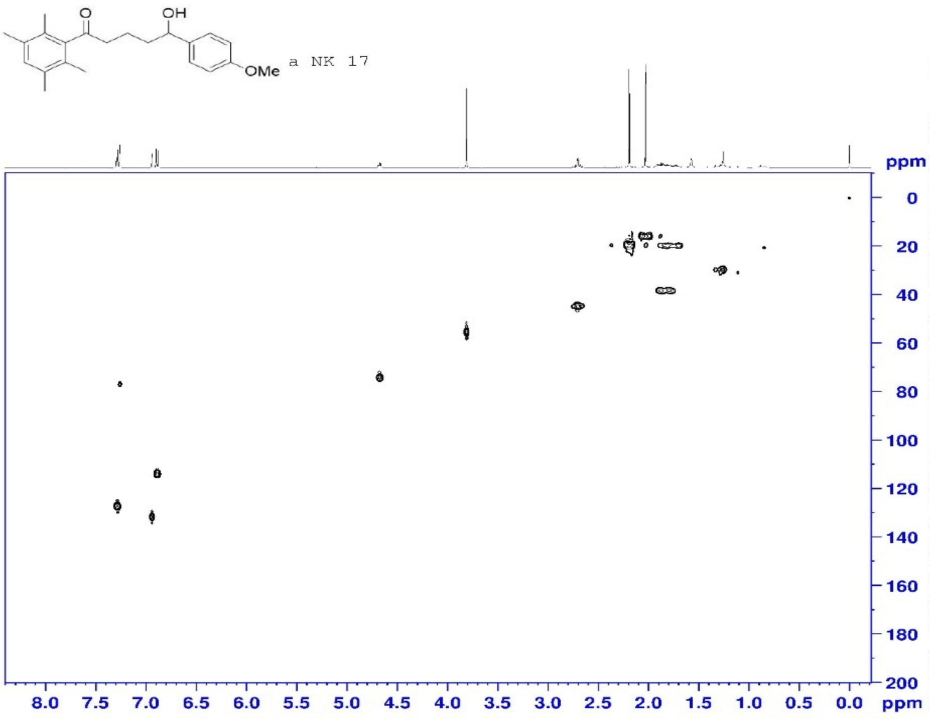

HMBC (126 MHz, $CDCl_3$) of 5-hydroxy-5-(4-methoxyphenyl)-1-(2,3,5,6-tetramethylphenyl)pentan-1-one **34b.**

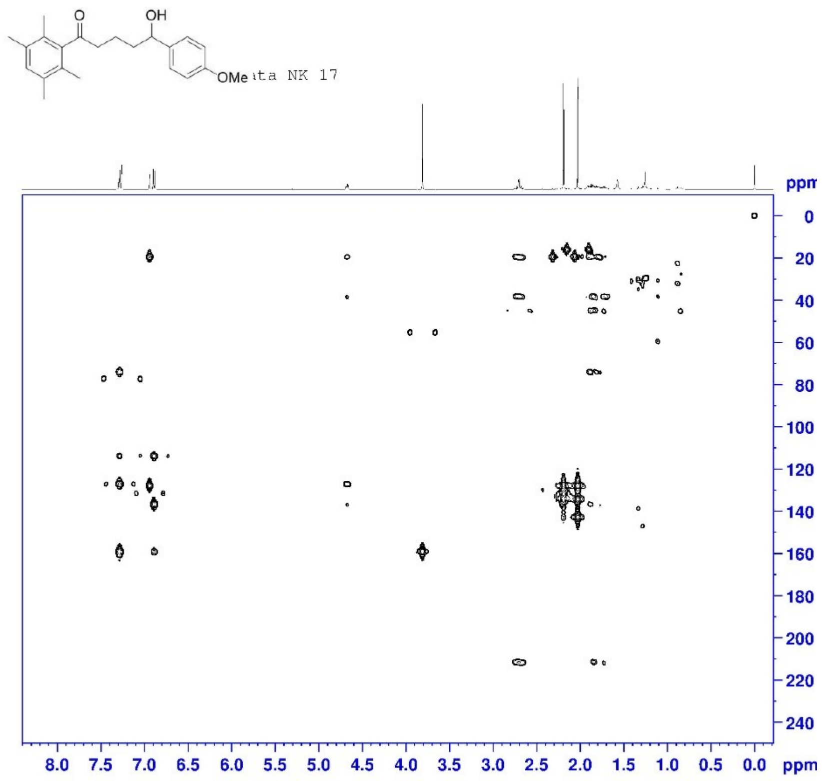

$^{13}C\{^1H\}$ NMR (126 MHz, $CDCl_3$) of 5-hydroxy-5-(4-methoxyphenyl)-1-(2,3,5,6-tetramethylphenyl)pentan-1-one **34b.**

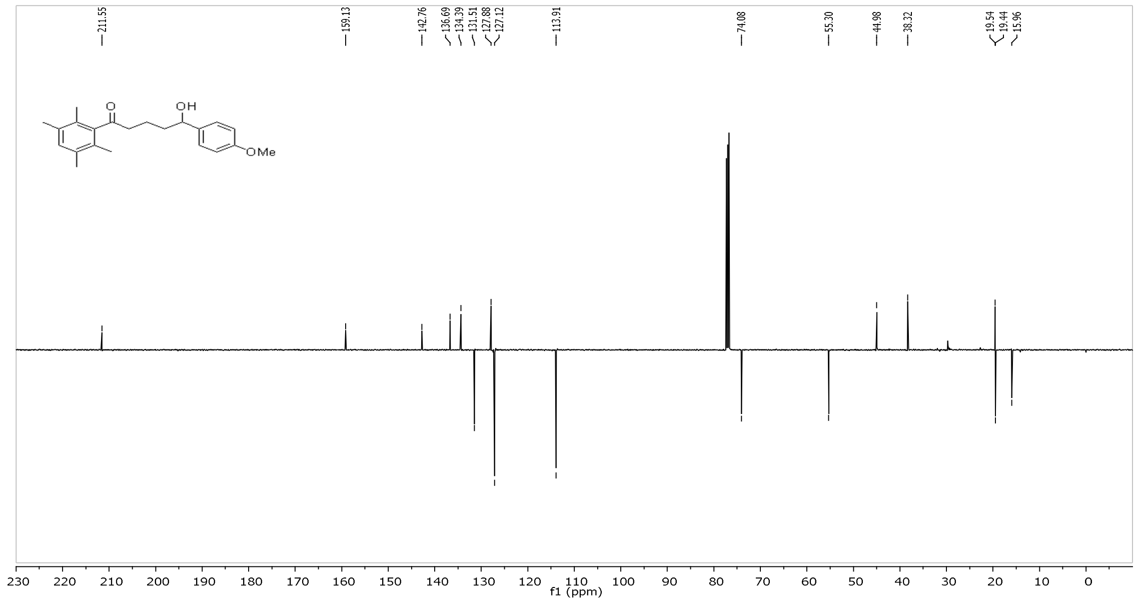

HPLC of racemic of 5-hydroxy-5-(4-methoxyphenyl)-1-(2,3,5,6-tetramethylphenyl)pentan-1-one **34b.**

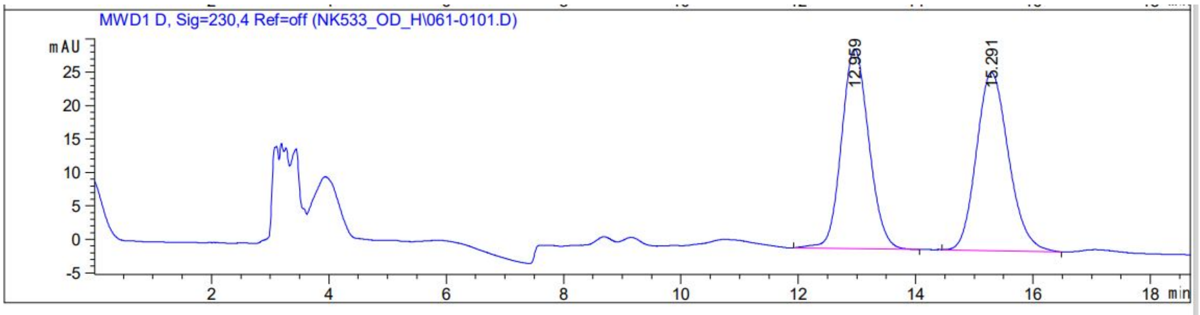

Signal 4: MWD1 D, Sig=230,4 Ref=off

Peak #	RetTime [min]	Type	Width [min]	Area [mAU*s]	Height [mAU]	Area %
1	12.959	BB	0.4993	965.28320	29.85157	48.7291
2	15.291	BB	0.5869	1015.63556	26.63434	51.2709
Totals :				1980.91876	56.48590	

HPLC of (*R*)-5-hydroxy-5-(4-methoxyphenyl)-1-(2,3,5,6-tetramethylphenyl)pentan-1-one **34b.**: (*R,R*)-3C-tethered Ru(II)-TsDPEN catalyst(after 48 h, 100% conversion, 93% ee (*R*)).

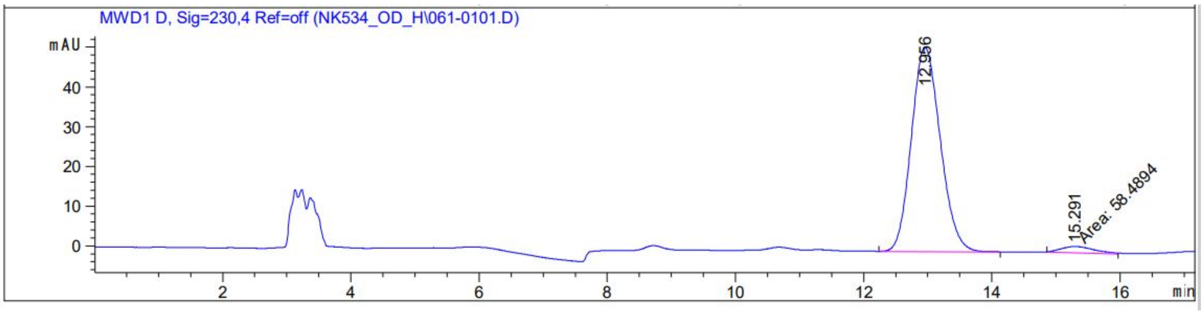

Signal 4: MWD1 D, Sig=230,4 Ref=off

Peak #	RetTime [min]	Type	Width [min]	Area [mAU*s]	Height [mAU]	Area %
1	12.956	BB	0.4916	1613.94519	51.50835	96.5027
2	15.291	MM	0.6107	58.48936	1.59625	3.4973
Totals :				1672.43456	53.10461	

Procedures for Unsymmetrical hydroxyketones 39b-41b.

2-(4-Acetylphenoxy)-1-phenylethan-1-one 39a.

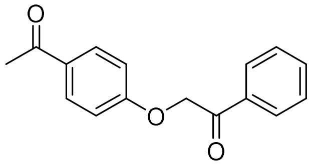

This compound has been reported; Ma, Z.; Zhou, M.; Ma, L.; M. Zhang, M. Synthesis of benzofurans from the cyclodehydration of α-phenoxy ketones mediated by Eaton's reagent. *J. Chem. Res.* **2020**, *44*, 426–436. To a dry 100 mL round-bottom flask containing a stirred solution of 4-hydroxyacetophenone (500 mg, 3.67 mmol) in acetone (23 mL), potassium carbonate (1.04 g, 7.50 mmol) was added under nitrogen atmosphere and the formed suspension was allowed to stir vigorously for 30 min at room temperature. Bromoacetophenone (1.49 g, 7.50 mmol)* was dissolved in acetone (1 mL) and added dropwise over 45 minutes, and the mixture was stirred at reflux for 24 h. After allowing to cool to room temperature, the mixture was quenched with water (20 mL), followed by addition of EtOAc (20 mL). The organic layer was separated, and the aqueous layer was extracted with EtOAc (2 x 20 mL). The combined organic layer was then dried ($MgSO_4$) and concentrated under reduced pressure to give the crude product which was purified using flash chromatography on silica gel eluted with 0-70% EtOAc in hexane to afford 2-(4-acetylphenoxy)-1-phenylethan-1-one **39a** as an orange solid (820 mg, 3.22 mmol, 88%). TLC: R_f ca 0.2 (4:1 hexane: EtOAc), UV active, strong $KMnO_4$. Mp 115.0-115.9 °C; HRMS (ESI+) *m/z*: $[M+Na]^+$ Calcd for $C_{16}H_{14}NaO_3$ 277.0835; Found 277.0832 (error 1.2 ppm); ν_{max} 1786, 1703, 1671, 1257, 1224, 1170, 976, 959 cm^{-1}; ^1H NMR (400 MHz, $CDCl_3$) δ 8.03-7.98 (2H, m, ArH), 7.96-7.92 (2H, m, ArH), 7.65 (1H, t, *J* 7.4, ArH), 7.53 (2H, t, *J* 7.7 ArH), 7.01-6.95 (2H, m, ArH), 5.38 (2H, s, OCH_2), 2.56 (3H, s, CH_3); $^{13}C\{^1H\}$ NMR (126 MHz, $CDCl_3$): δ 196.7 (C), 193.5 (C), 161.8 (C), 134.3 (C), 134.2 (C), 131.1 (CH), 130.6 (CH), 129.0 (CH), 128.1 (CH), 114.5 (CH), 70.5 (CH_2), 26.4 (CH_3); *m/z* (ES-API+) 277.0 (M^+ + Na, 100%). *The use of an excess of bromide over hydroxyacetophenone facilitates the purification, since the product co-elutes with the hydroxyphenone starting material.

^{1}H NMR (400 MHz, $CDCl_3$) of 2-(4-acetylphenoxy)-1-phenylethan-1-one **39a**.

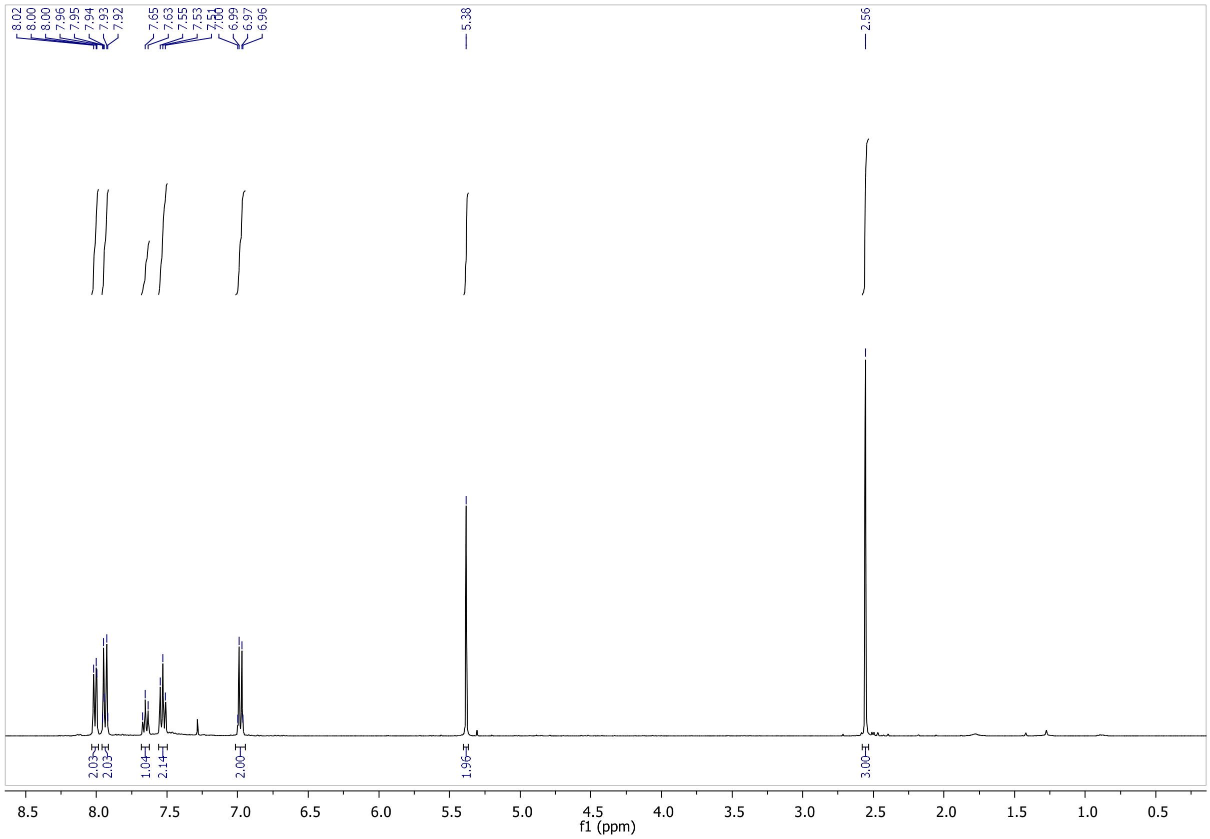

Expanded Aromatic Region (6.70-8.30 ppm) (400 MHz, $CDCl_3$)

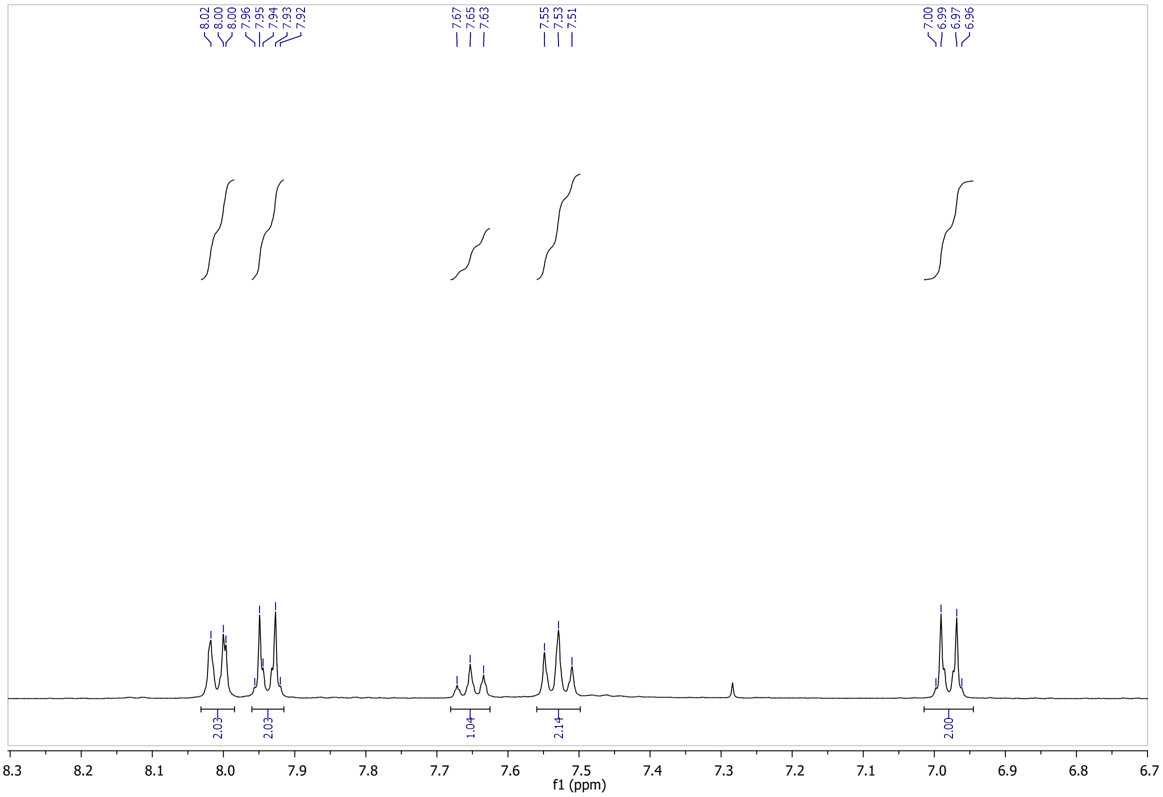

COSY (400 MHz, $CDCl_3$) of 2-(4-acetylphenoxy)-1-phenylethan-1-one **39a**.

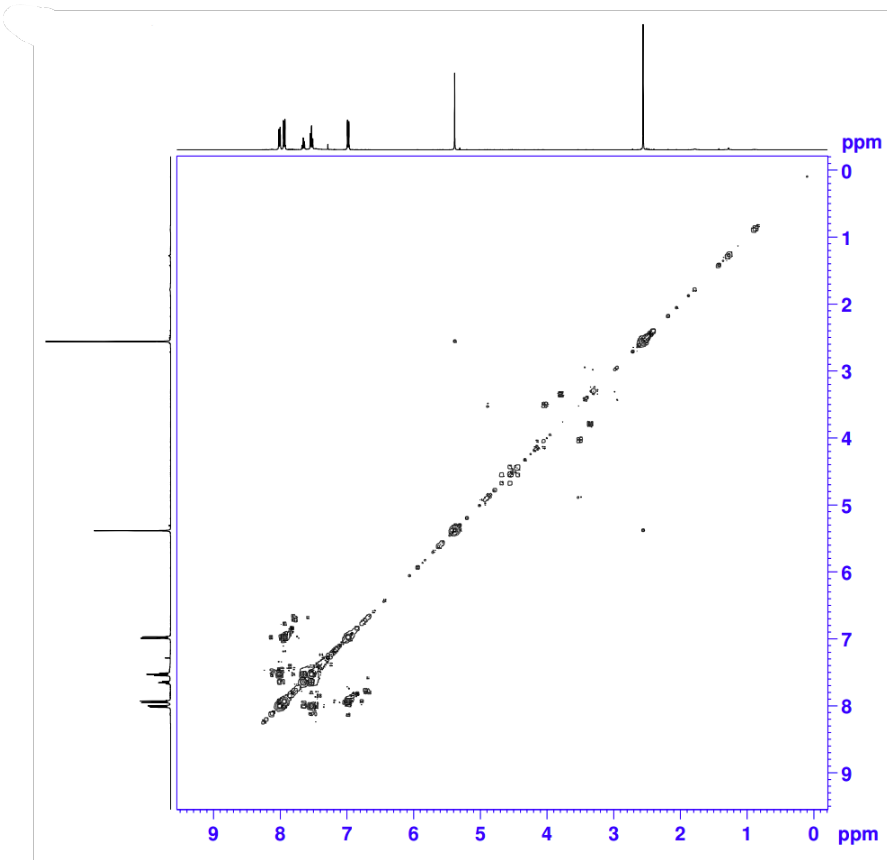

HSQC (400 MHz, $CDCl_3$) of 2-(4-acetylphenoxy)-1-phenylethan-1-one **39a**.

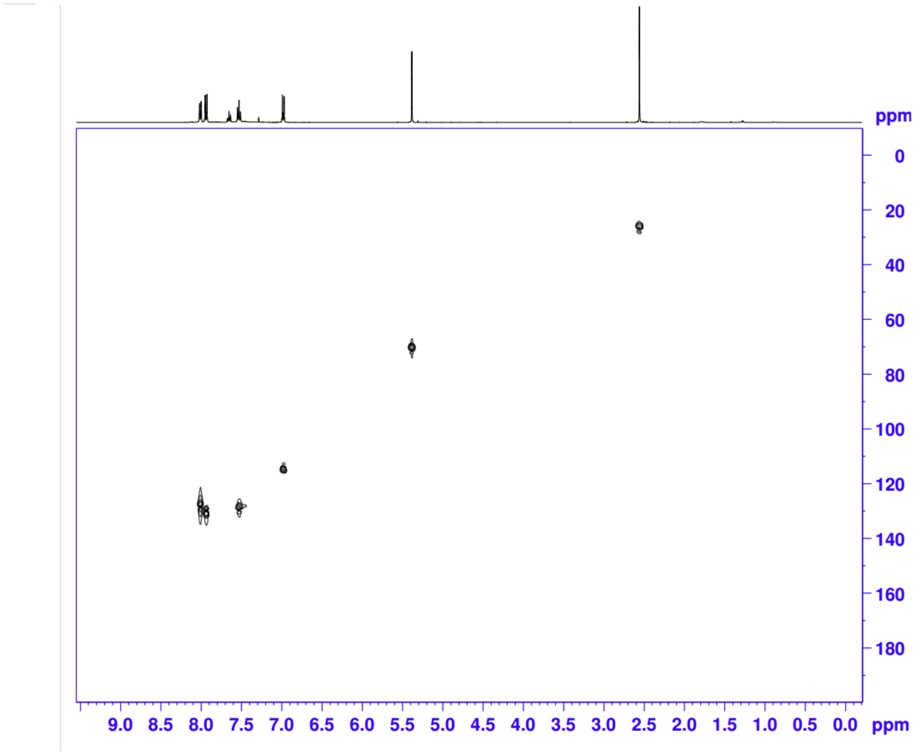

HMBC (400 MHz, $CDCl_3$) of 2-(4-acetylphenoxy)-1-phenylethan-1-one **39a**.

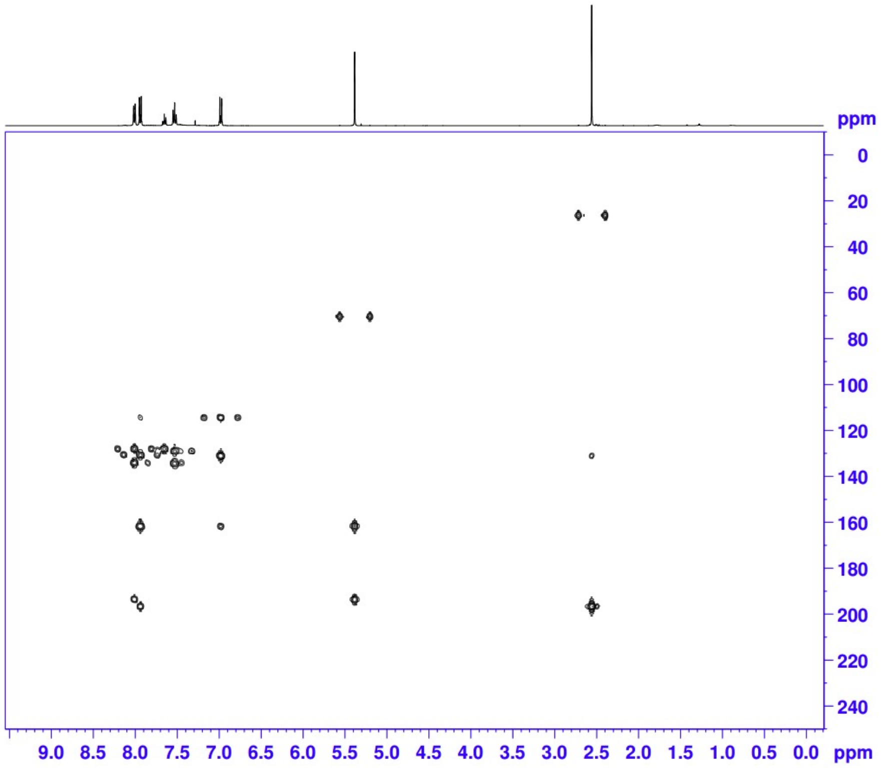

$^{13}C\{^1H\}$ NMR (400 MHz, $CDCl_3$) of 2-(4-acetylphenoxy)-1-phenylethan-1-one **39a**.

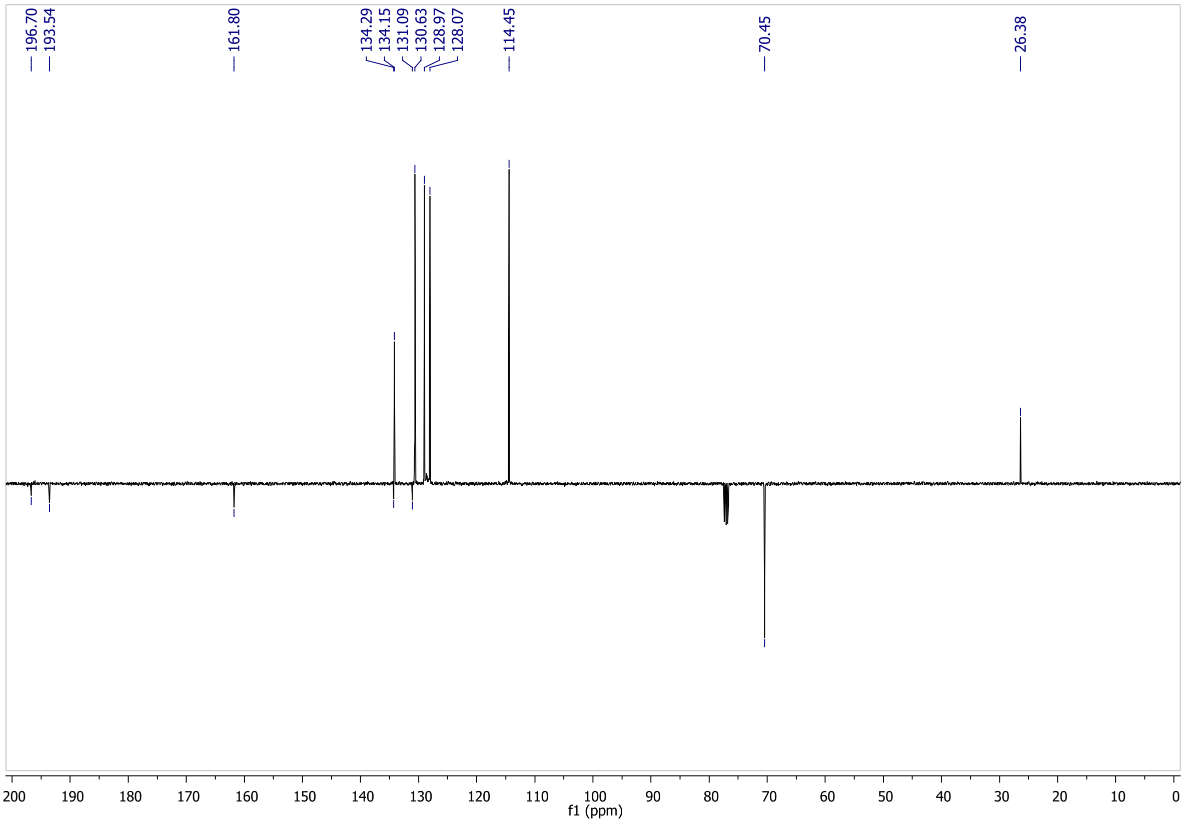

HPLC of 2-(4-acetylphenoxy)-1-phenylethan-1-one **39a**.

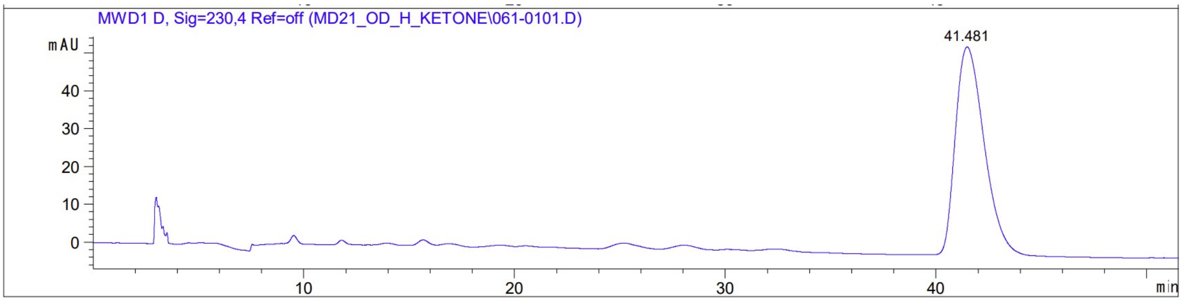

Signal 4: MWD1 D, Sig=230,4 Ref=off

Peak #	RetTime [min]	Type	Width [min]	Area [mAU*s]	Height [mAU]	Area %
1	41.481	BB	1.4243	5231.48584	55.07052	100.0000

Totals : 5231.48584 55.07052

(*S*)-2-(4-((*R*)-1-Hydroxyethyl)phenoxy)-1-phenylethan-1-ol 39b.

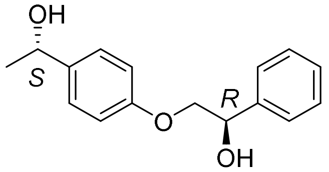

This compound is novel. A FA/TEA solution (0.30 mL) was added to a 50 mL Schlenk under a nitrogen atmosphere containing 0.5 mol% (*S,S*)-Ru-3C-Teth (1.8 mg, 0.0030 mmol) and the mixture was stirred for 10 min to allow for the catalyst to be activated. 2-(4-Acetylphenoxy)-1-phenylethan-1-one **39a** (150 mg, 0.60 mmol) was dissolved in DCM (0.30 mL) and added to the mixture which was allowed to stir for 24 h. The crude product which was purified using flash chromatography on silica gel eluted with 0-70% EtOAc in hexane to yield (*S*)-2-(4-((*R*)-1-hydroxyethyl)phenoxy)-1-phenylethan-1-ol **39b** as a white solid (89 mg, 0.35 mmol, 58%). TLC: R_f ca 0.6 (1:1 hexane: EtOAc), UV active, strong $KMnO_4$; Mp 89.2-90.0 °C; HRMS (ESI) *m/z*: $[M+Na]^+$ Calcd for $C_{16}H_{18}NaO_3$ 281.1148; Found 281.1148 (error 0.1 ppm); ν_{max} 3342, 3276, 2992, 2966, 1678, 1612, 1284, 1254, 1025, 1014 cm^{-1}; $[\alpha]_D^{22}$ = -33.1 (c=0.4, $CHCl_3$, T = 20.5°C); ^1H NMR (400 MHz, $CDCl_3$): δ 7.35-7.30 (2H, m, ArH), 7.30-7.19 (3H, m, ArH), 7.15 (2H, d, *J* 8.6, ArH), 6.76 (2H, d, *J* 8.6 ArH), 4.97 (1H, dd, *J* 3.0, 8.6, CH_2CH), 4.69 (1H, q, *J* 6.4, CH_3CH), 3.96 (1H, dd, *J* 9.6, 3.0, OCH_2), 3.92-3.84 (1H, m, OCH_2), 1.34 (3H, d, *J* 6.4, CH_3); $^{13}C\{^1H\}$ NMR (126 MHz, $CDCl_3$): δ 157.8 (C), 139.8 (C), 138.7 (C), 128.6 (CH), 128.2 (CH), 126.8 (CH), 126.3 (CH), 114.6 (CH), 73.5 (CH), 72.5 (CH), 69.9 (CH_2), 25.1 (CH_3); *m/z* (ES-API+) 281.1 (M^+ + Na, 100%). Enantiomeric excess and conversion determined by HPLC analysis (Chiralcel ODH, 30 cm x 6 mm column, iPrOH:hexane 1:9, 1 mL/min, T = 25 °C) ketone 41.4 min, (*S,R*) isomer 52.3 min, (*S,S)*/(*R,R)* isomers 44.4/37.3 min (cannot distinguish) *R,S* isomer 35.5 min. >99% ee (*S,R*), dr 96.5:3.5. (Retention times are taken from the racemic reduction product – see below). The configurations of the chiral centers are based on precedents for the catalysts used.

^1H NMR (400 MHz, $CDCl_3$) of (*S*)-2-(4-((*R*)-1-hydroxyethyl)phenoxy)-1-phenylethan-1-ol **39b**.

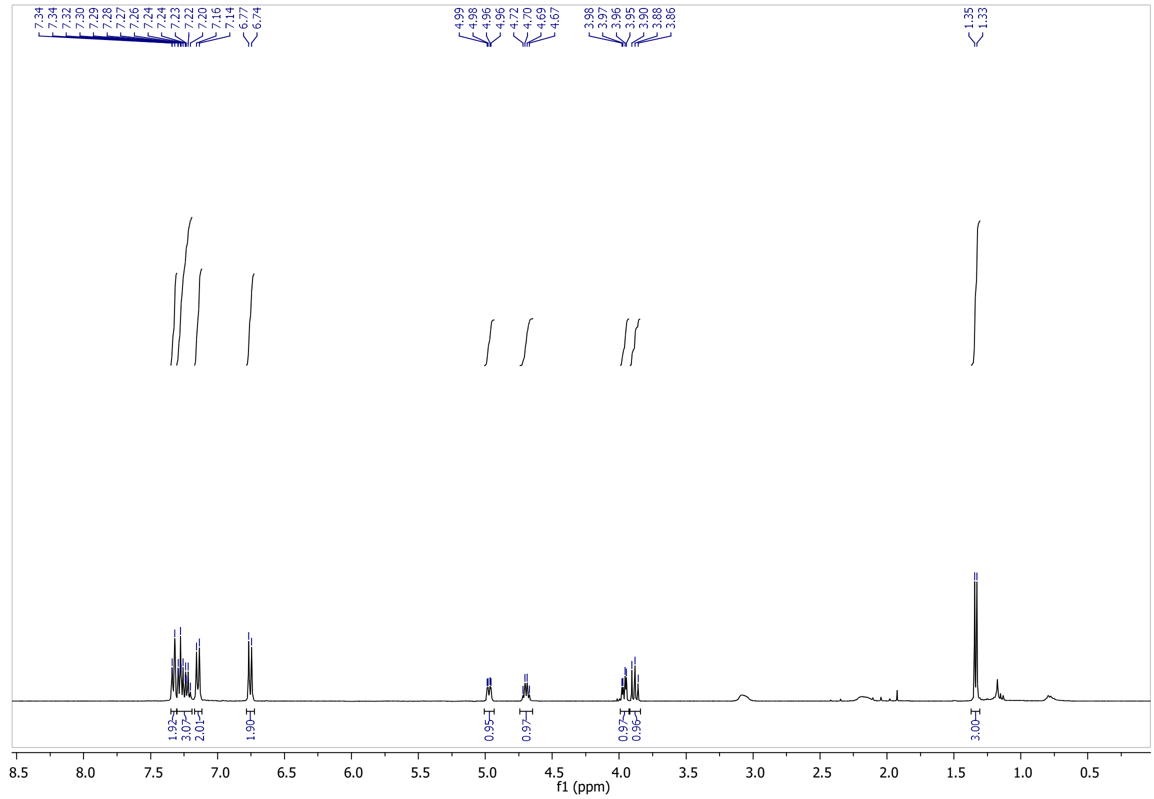

Expanded Aromatic Region (6.50-7.50 ppm)

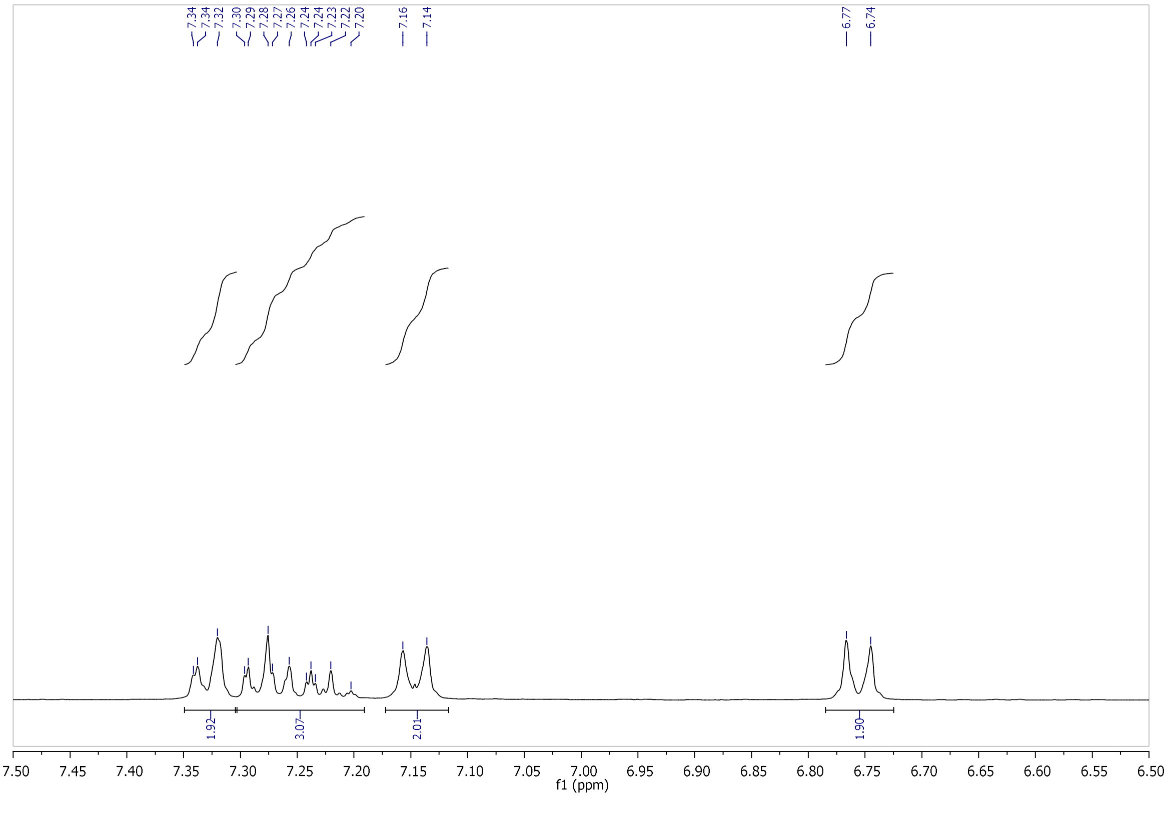

Expanded Region (3.80-5.10 ppm) of (*S*)-2-(4-((*R*)-1-hydroxyethyl)phenoxy)-1-phenylethan-1-ol **39b**.

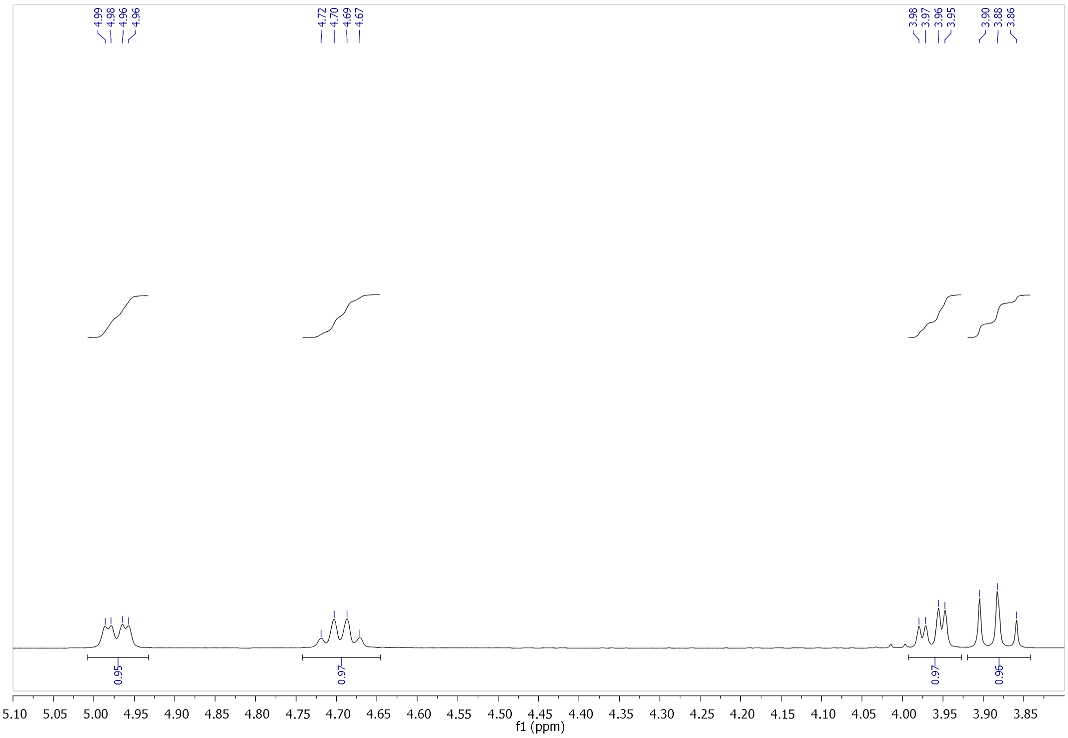

5c

COSY (400 MHz, $CDCl_3$) of (*S*)-2-(4-((*R*)-1-hydroxyethyl)phenoxy)-1-phenylethan-1-ol **39b**.

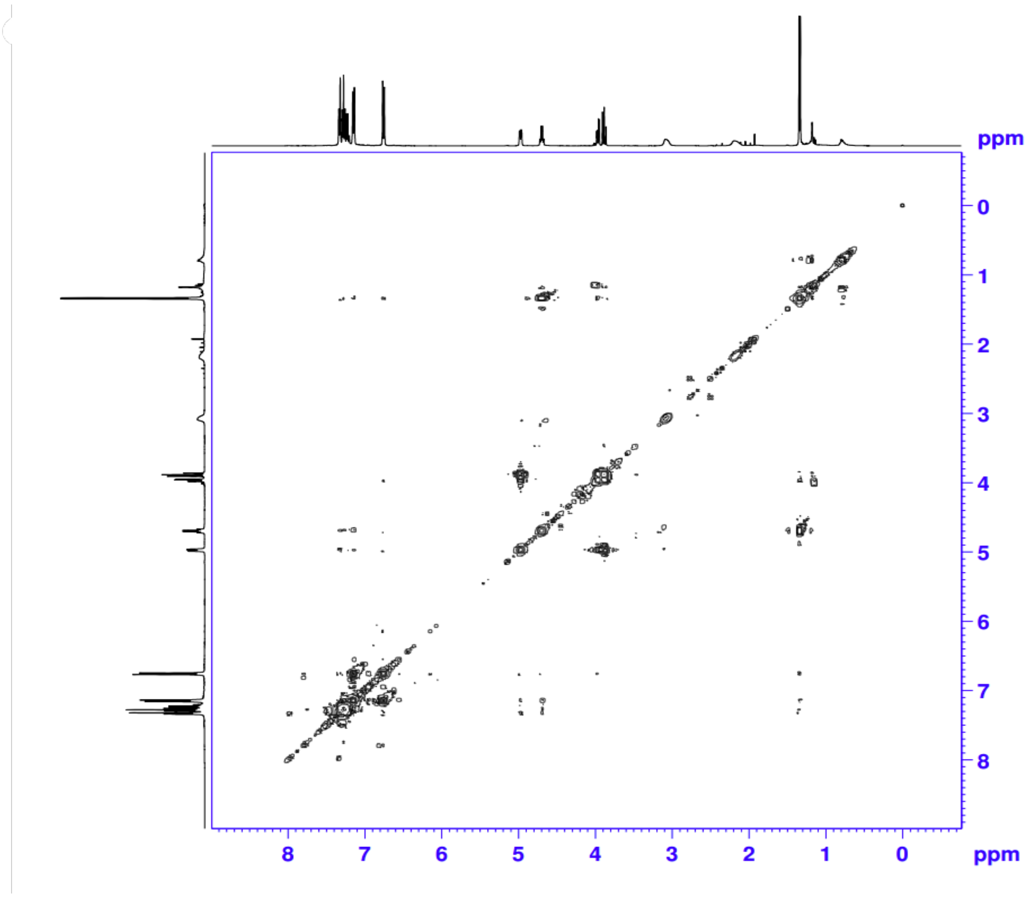

HSQC (400 MHz, $CDCl_3$) of (*S*)-2-(4-((*R*)-1-hydroxyethyl)phenoxy)-1-phenylethan-1-ol **39b**.

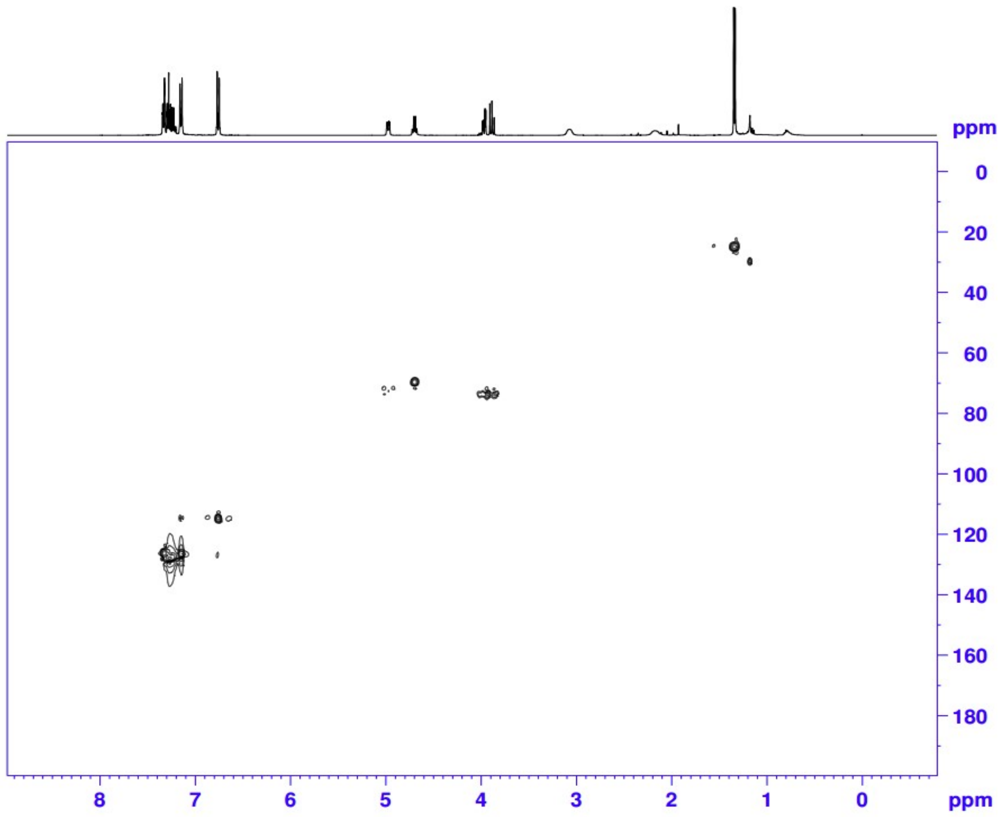

HMBC (400 MHz, $CDCl_3$) of (*S*)-2-(4-((*R*)-1-hydroxyethyl)phenoxy)-1-phenylethan-1-ol **39b**.

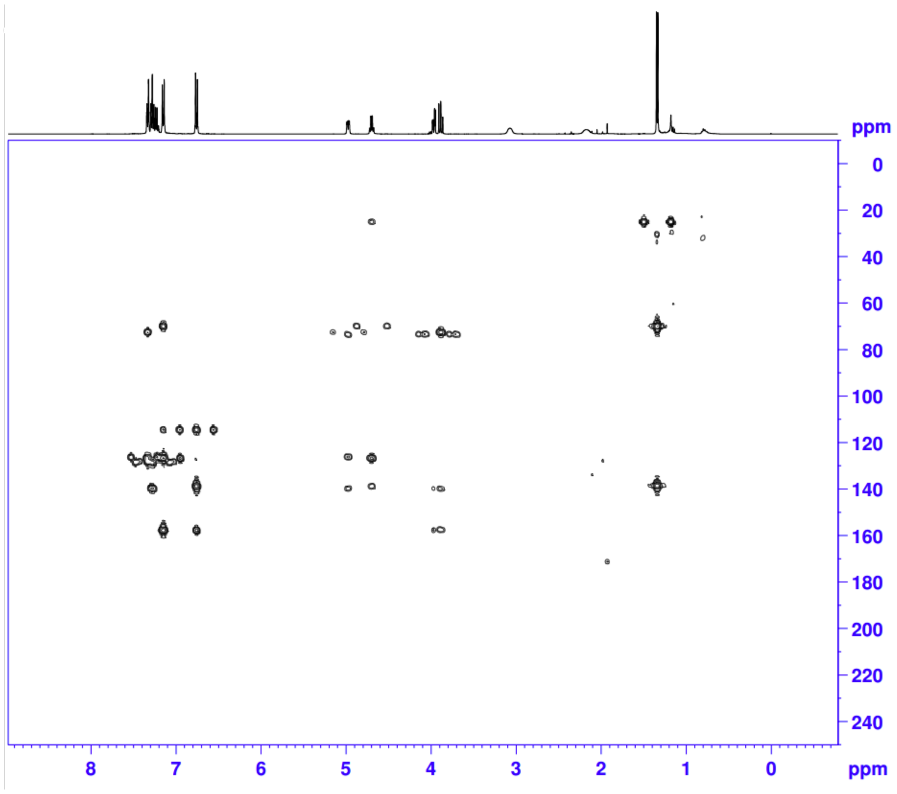

$^{13}C\{^{1}H\}$ NMR (400 MHz, $CDCl_3$) of (*S*)-2-(4-((*R*)-1-hydroxyethyl)phenoxy)-1-phenylethan-1-ol **39b**.

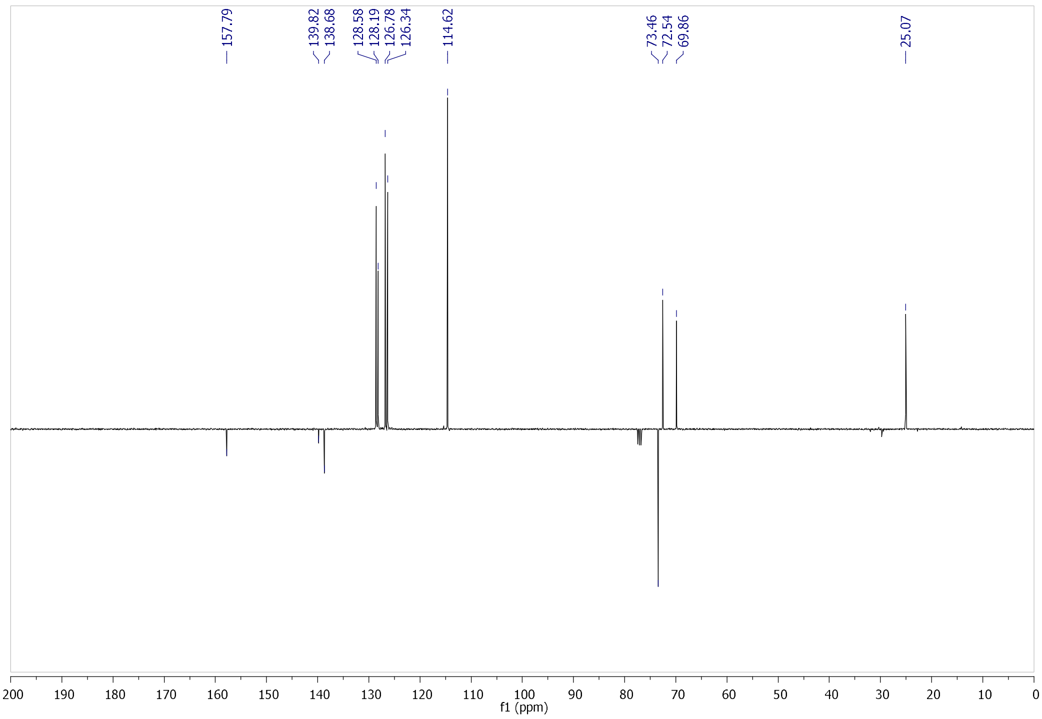

HPLC of (*S*)-2-(4-((*R*)-1-hydroxyethyl)phenoxy)-1-phenylethan-1-ol **39b.** (*S,S*)-3C-tethered Ru(II)-TsDPEN catalyst

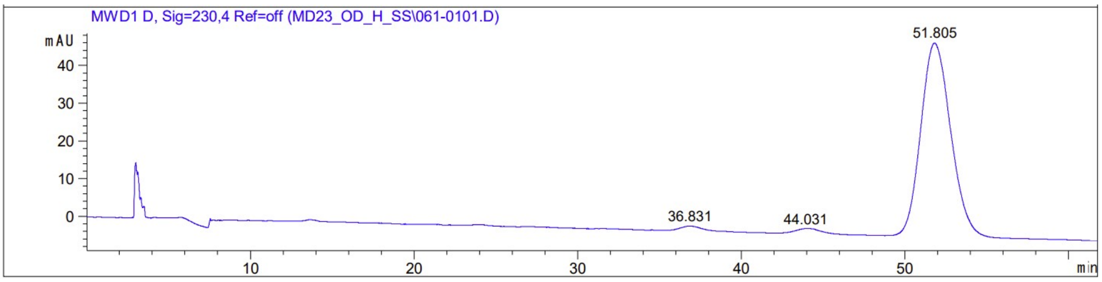

Signal 4: MWD1 D, Sig=230,4 Ref=off

Peak #	RetTime [min]	Type	Width [min]	Area [mAU*s]	Height [mAU]	Area %
1	36.831	MM	1.4202	108.57578	1.27423	1.6062
2	44.031	MM	1.6546	131.56030	1.32520	1.9463
3	51.805	MM	2.1178	6519.47803	51.30639	96.4475

Totals : 6759.61411 53.90581

(*R*)-2-(4-((*S*)-1-Hydroxyethyl)phenoxy)-1-phenylethan-1-ol 39b.

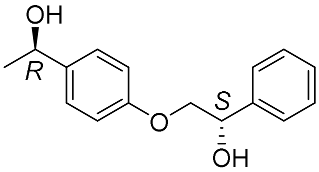

This compound is novel. A FA/TEA solution (0.30 mL) was added to a 50 mL Schlenk under nitrogen atmosphere containing 0.5 mol% (*R,R*)-Ru-3C-Teth (1.8 mg, 0.0030 mmol) and the mixture was stirred for 10 min to allow for the catalyst to be activated. 2-(4-Acetylphenyl)-1-phenylethan-1-one (150 mg, 0.60 mmol) was dissolved in DCM (0.30 mL) and added to the mixture which was allowed to stir for 24 h. (*R*)-2-(4-((*S*)-1-hydroxyethyl)phenyl)-1-phenylethan-1-ol was isolated as a white solid (140 mg, 0.580 mmol, 96%), >99% ee (*R,S*), dr 99:1. The data for this compound corresponded to that formed using the (*S,S*)-catalyst, with the exception of chiral HPLC data. The configurations of the chiral centers are based on precedents for the catalysts used.

HPLC of (*R*)-2-(4-((*S*)-1-hydroxyethyl)phenoxy)-1-phenylethan-1-ol **39b.** (*R,R*)-3C-tethered Ru(II)-TsDPEN catalyst.

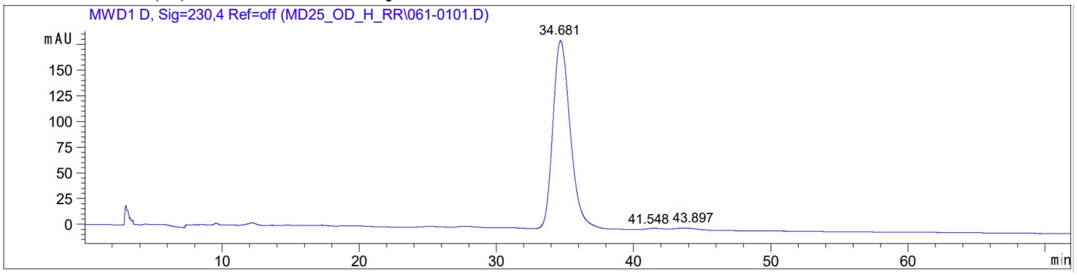


```
Signal 4: MWD1 D, Sig=230,4 Ref=off

Peak RetTime Type  Width     Area      Height     Area
  #   [min]        [min]   [mAU*s]     [mAU]       %
----|-------|----|-------|----------|----------|--------|
   1  34.681 BB    1.3439 1.61270e4   183.03528  98.9804
   2  41.548 MM    1.0941   59.23521 9.02344e-1  0.3636
   3  43.897 MM    1.3584  106.88706    1.31139   0.6560

Totals :                  1.62931e4   185.24901
```

Racemic 2-(4-(1-hydroxyethyl)phenoxy)-1-phenylethan-1-ol 39b.

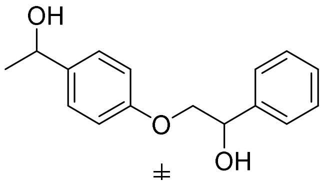

This compound is novel. For racemic reduction by $NaBH_4$, to a 25 mL round-bottom flask containing 2-(4-acetylphenyl)-1-phenylethan-1-one (50 mg, 0.20 mmol) dissolved in methanol (4.5 mL) and water (0.5 mL), was added sodium borohydride (23 mg, 0.60 mmol) and the mixture was stirred for 24 h. The solvent was removed at the end of this time and EtOAc (20 mL) and water (20 mL) was added. The organic layer was separated and the water layer was extracted with further EtOAc (2 x 20 mL). the combined organic extracts were dried ($MgSO_4$) and filtered and the solvent was removed under vacuum. Crude 2-(4-(1-hydroxyethyl)phenyl)-1-phenylethan-1-ol **39a** was isolated as a white solid (8.3 mg, 0.034, 17%). The ^{1}H NMR spectrum contained signals corresponding to the major diastereoisomer formed in the asymmetric reductions, although the HPLC analysis revealed the presence of two diastereoisomers formed in a 1:1 ratio. This indicates that both product diastereoisomers have overlapping NMR signals.

HPLC of racemic 2-(4-(1-hydroxyethyl)phenoxy)-1-phenylethan-1-ol **39b.**

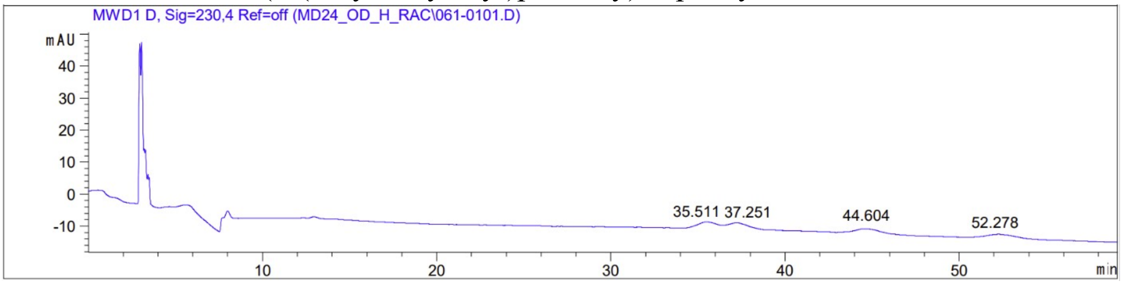

Signal 4: MWD1 D, Sig=230,4 Ref=off

Peak #	RetTime [min]	Type	Width [min]	Area [mAU*s]	Height [mAU]	Area %
1	35.511	MF	1.3554	166.50038	2.04732	27.2757
2	37.251	FM	1.3374	159.11349	1.98291	26.0656
3	44.604	MM	1.6768	148.00191	1.47104	24.2454
4	52.278	MM	1.8164	136.81808	1.25540	22.4133
Totals :				610.43387	6.75667	

Data for the conversion of compound 39a over time.

Time (minutes)	Starting Material **39a** Conversion/ mole fraction	Intermediate **42** formation/ mole fraction	Product **39b** formation/ mole fraction
0	1.000	0.000	0.000
90	0.462	0.538	0.000
178	0.306	0.694	0.000
322	0.072	0.928	0.000
429	0.050	0.886	0.071
1244	0.000	0.658	0.342
1508	0.000	0.585	0.415
1824	0.000	0.505	0.495
2693	0.000	0.332	0.668
3204	0.000	0.255	0.745
4175	0.000	0.160	0.840
4733	0.000	0.139	0.861
5627	0.000	0.098	0.902
6226	0.000	0.086	0.914

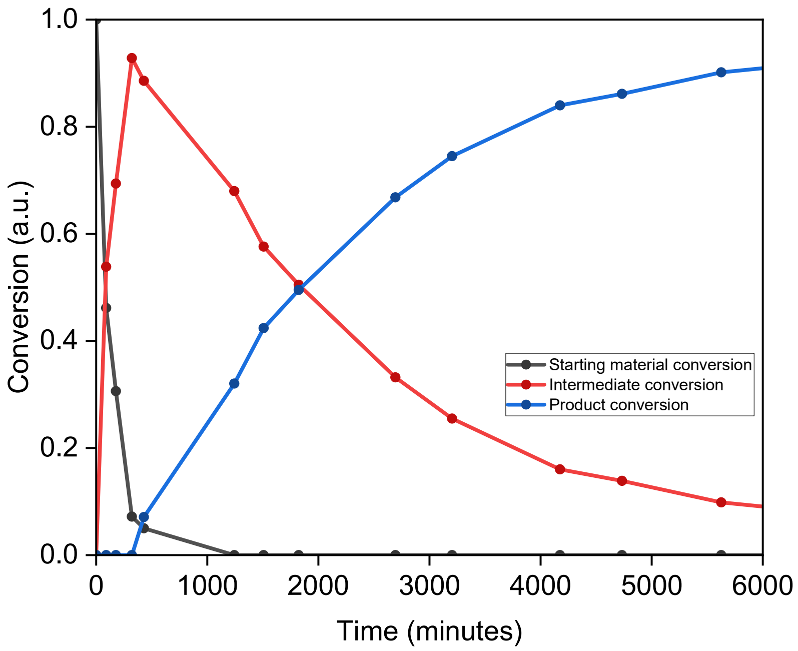

Graph to show how the conversion of product varies with starting material and intermediate conversion in the reduction of **39a**. The reaction was followed for a period of 5 days, with ^{1}H NMR spectra obtained at regular intervals. Time recorded from the point in which substrate was added to catalyst/FA/TEA mixture. Data obtained from integration of (1H, d, ArH) environment of starting material, intermediate and product in the region 6.7-6.9 ppm.

4’-Hydroxy-2’,3’,5’,6’-tetramethylacetophenone.

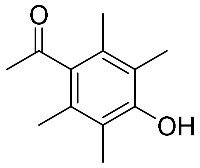

This compound is novel. To a dry 50 mL round-bottom flask containing a stirred solution of 2,3,5,6-tetramethylacetophenone (88 mg, 0.50 mmol) dissolved in hexafluoro-2-propanol (5 mL), phthaloyl peroxide was added (123 mg, 0.75 mmol) under nitrogen atmosphere and the mixture was left to stir for 12 h at 40°C. After addition of a 1:9 mixture of methanol/sodium bicarbonate (10 mL), the mixture was set to stir at 40°C before being quenched with brine (20 mL), followed by addition of DCM (20 mL). The organic layer was separated, and the aqueous layer was extracted with DCM (3 x 15 mL). The combined organic layer was then dried ($MgSO_4$) and concentrated under reduced pressure to give the crude product which was purified using flash chromatography on silica gel eluted with 0-70% EtOAc in hexane to afford 4’-hydroxy-2’,3’,5’,6’-tetramethylacetophenone as a white solid (78 mg, 0.41 mmol, 81%). TLC R_f ca 0.4 (4:1 hexane: EtOAc), UV active, faint $KMnO_4$; Mp 161.7-163.0 °C; HRMS (ESI) *m/z*: $[M+Na]^+$ Calcd for $C_{12}H_{16}NaO_4$ 215.1043; Found 215.1040 (error 0.9); ν_{max} 3438, 2976, 2916, 1680, 1573, 1222, 1172 cm^{-1}; ^1H NMR (300 MHz, $CDCl_3$): δ 4.74 (1H, s, OH), 2.47 (3H, s, $COCH_3$), 2.16 (12H, d, *J* 7.7, CCH_3); $^{13}C\{^1H\}$ NMR (126 MHz, $CDCl_3$): δ 209.8 (C), 151.8 (C), 136.2 (C), 128.7 (C), 119.7 (C), 33.2 (CH_3), 16.6 (CH_3), 11.7 (CH_3); *m/z* (ES-API+) 215.0 (M^+ + Na, 100%).

^{1}H NMR (400 MHz, $CDCl_3$) of 4’-hydroxy-2’,3’,5’,6’-tetramethylacetophenone.

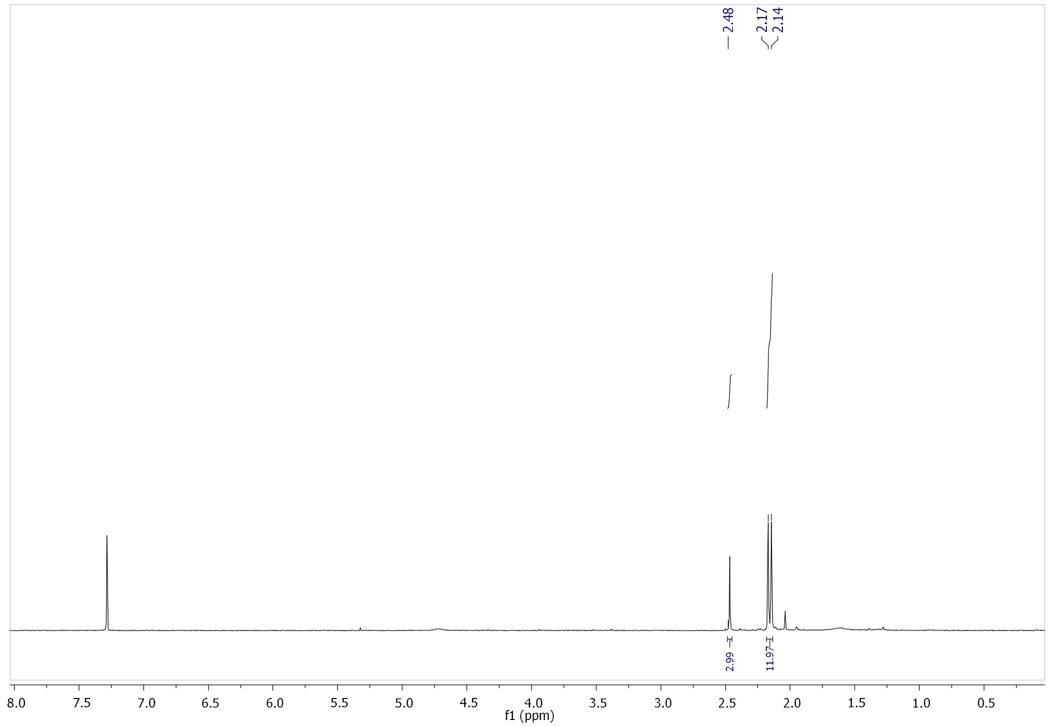

COSY (500 MHz, $CDCl_3$) 4’-hydroxy-2’,3’,5’,6’-tetramethylacetophenone.

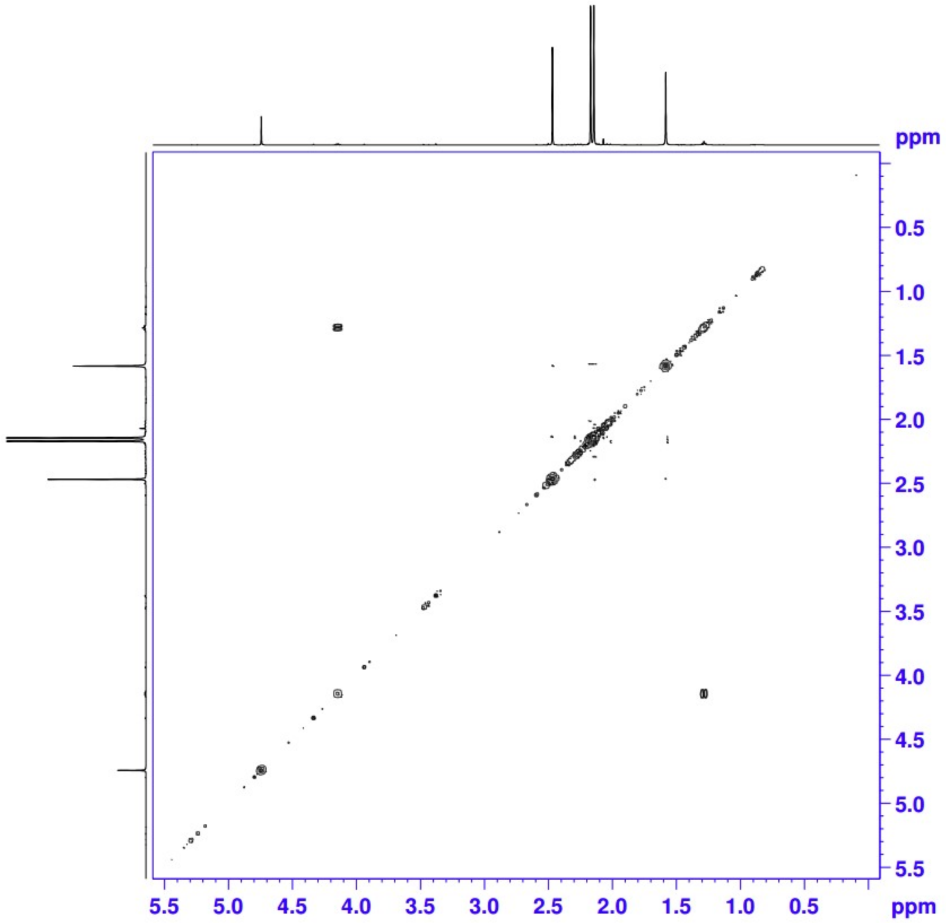

HSQC (500 MHz, $CDCl_3$) 4’-hydroxy-2’,3’,5’,6’-tetramethylacetophenone.

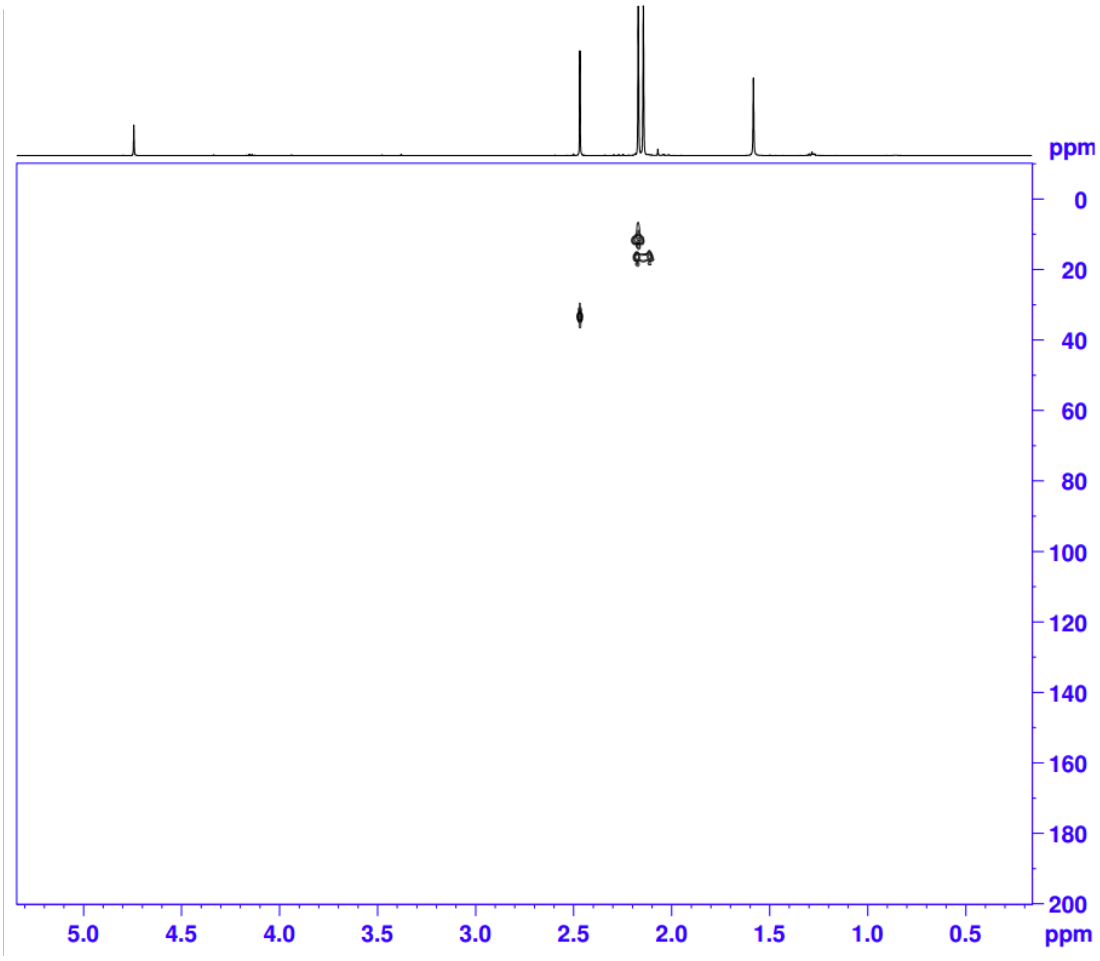

HMBC (500 MHz, $CDCl_3$) 4’-hydroxy-2’,3’,5’,6’-tetramethylacetophenone.

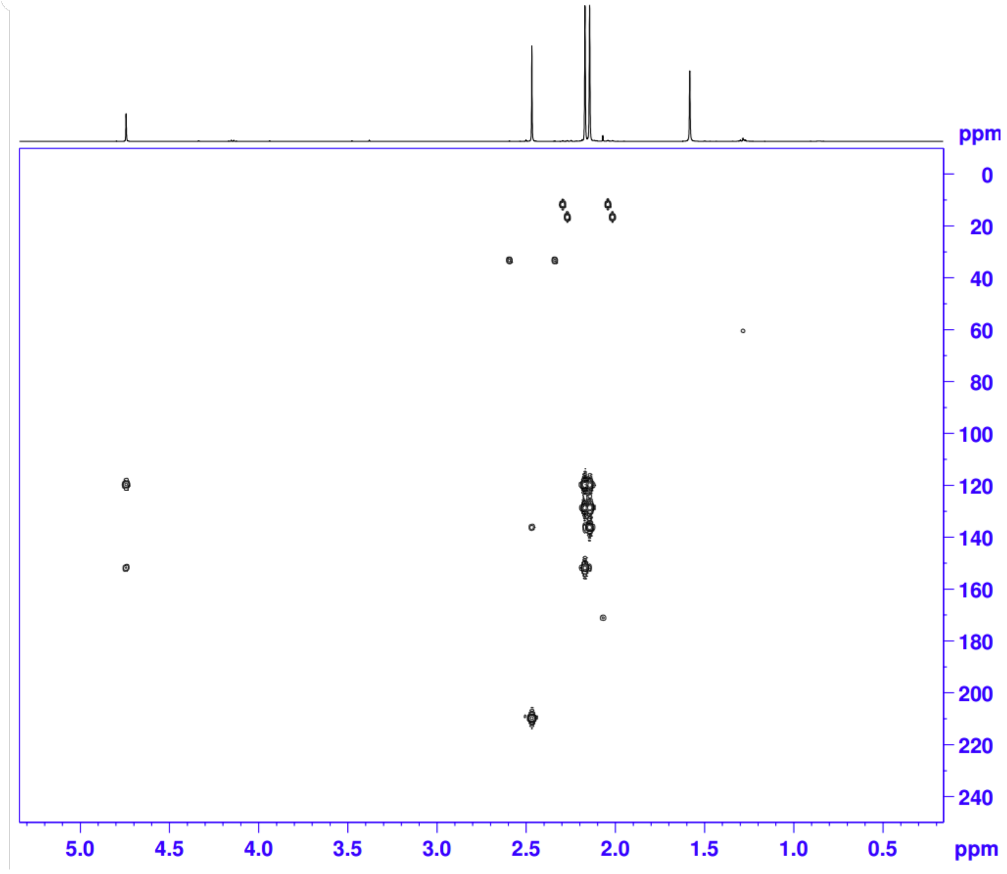

$^{13}C\{^{1}H\}$ NMR (500 MHz, $CDCl_3$) 4’-hydroxy-2’,3’,5’,6’-tetramethylacetophenone.

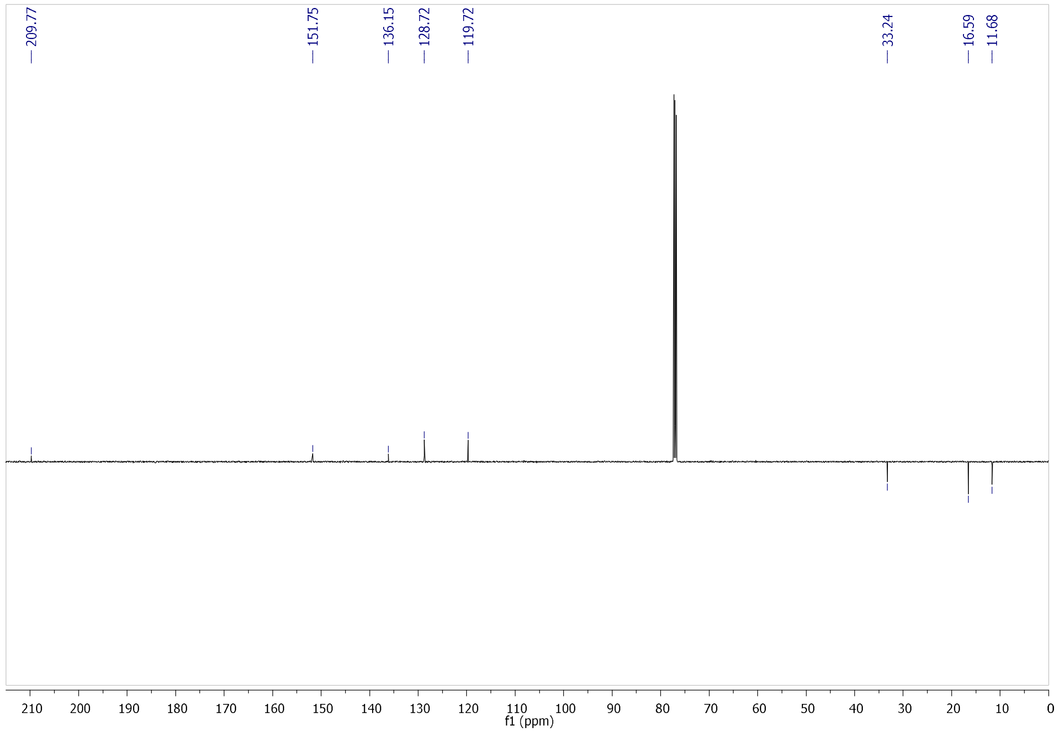

2-Bromo-1-(2,3,5,6-tetramethylphenyl)ethan-1-one.

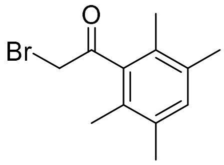

This compound is known but not fully characterized. Yamamoto, T.; Togo, H. One-Pot Preparation of Aromatic Amides, 4-Arylthiazoles, and 4-Arylimidazoles from Arenes. *Eur. J. Org. Chem.*, **2018**, 4187–4196. A dry 25 mL round-bottom flask containing a stirred suspension of copper(II) bromide (580 mg, 2.6 mmol) and EtOAc (2 mL) was heated to reflux, followed by the addition of 2,3,5,6-tetramethylacetophenone (230 mg, 1.3 mmol) dissolved in chloroform (1.5 mL). After stirring at reflux for 3 h, the mixture was cooled to room temperature and filtered on a pad of celite to remove the white precipitate. After washing the precipitate with chloroform (3 x 10 mL), the filtrate was then washed with 5% sodium bicarbonate (3 x 10 mL), dried ($MgSO_4$), and concentrated under reduced pressure to afford 2-bromo-1-(2,3,5,6-tetramethylphenyl)ethan-1-one as a clear oil that was used with no further purification. TLC R_f ca 0.8 (95:1 hexane: EtOAc), UV active, strong $KMnO_4$; HRMS (ESI+) *m/z*: $[M+Na]^+$ Calcd for $C_{12}H_{15}BrNaO$ 277.0198; Found 277.0196 (error 1.1); ν_{max} 2936, 1720, 1469, 1386 cm^{-1} ^1H NMR (400 MHz, $CDCl_3$): δ 7.03 (1H, s, ArH), 4.30 (2H, s, CH_2), 2.24 (6H s, $COCCH_3$), 2.11 (6H, s, CCH_3). $^{13}C\{^1H\}$ NMR (126 MHz, $CDCl_3$): δ 201.0 (C), 139.1 (C), 134.7 (C), 132.6 (CH), 128.9 (C), 37.7 (CH_2), 19.4 (CH_3), 16.3 (CH_3); *m/z* (ES-API+) 277.0 (M^+ + Na, 100%).

^1H NMR ($CDCl_3$, 500 MHz) of 2-bromo-1-(2,3,5,6-tetramethylphenyl)ethan-1-one.

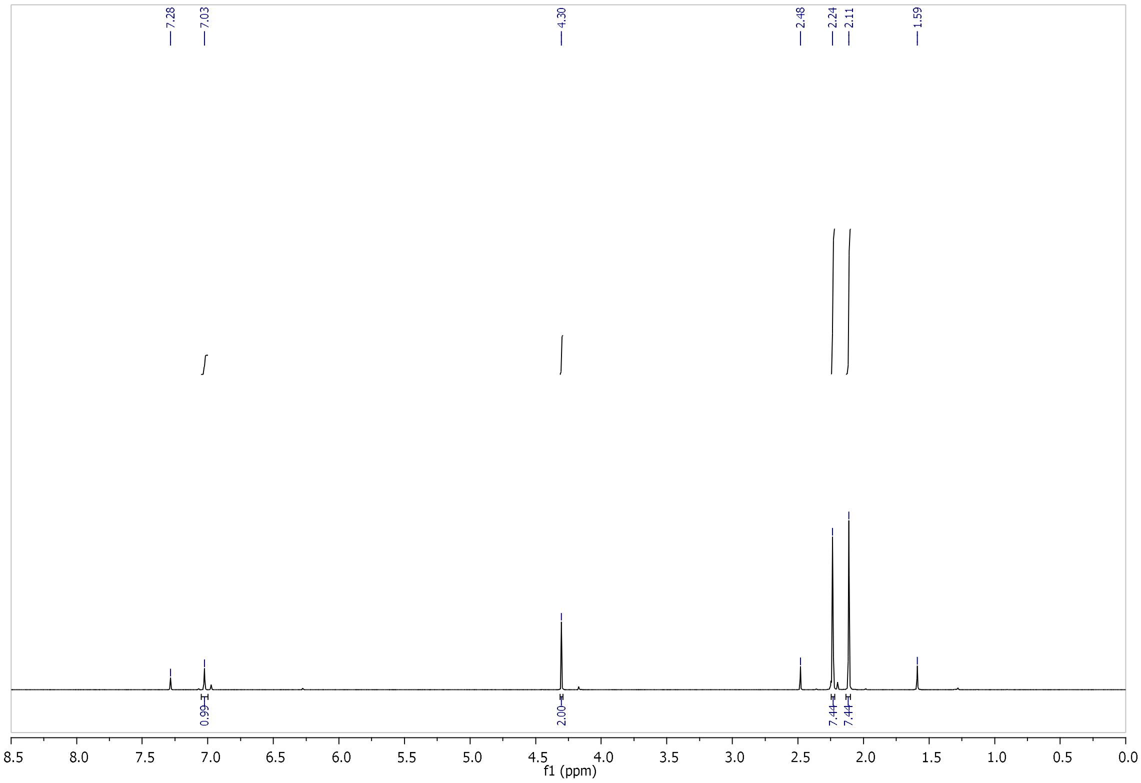

COSY ($CDCl_3$, 500 MHz) of 2-bromo-1-(2,3,5,6-tetramethylphenyl)ethan-1-one.

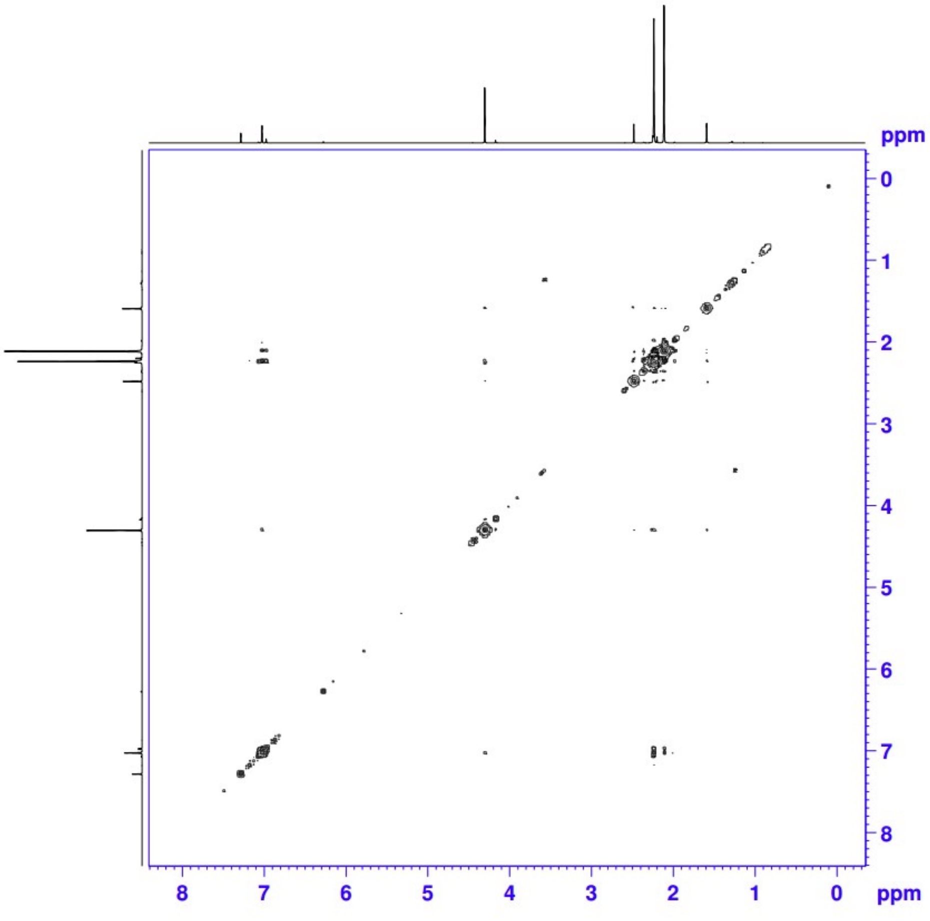

HSQC ($CDCl_3$, 500 MHz) of 2-bromo-1-(2,3,5,6-tetramethylphenyl)ethan-1-one.

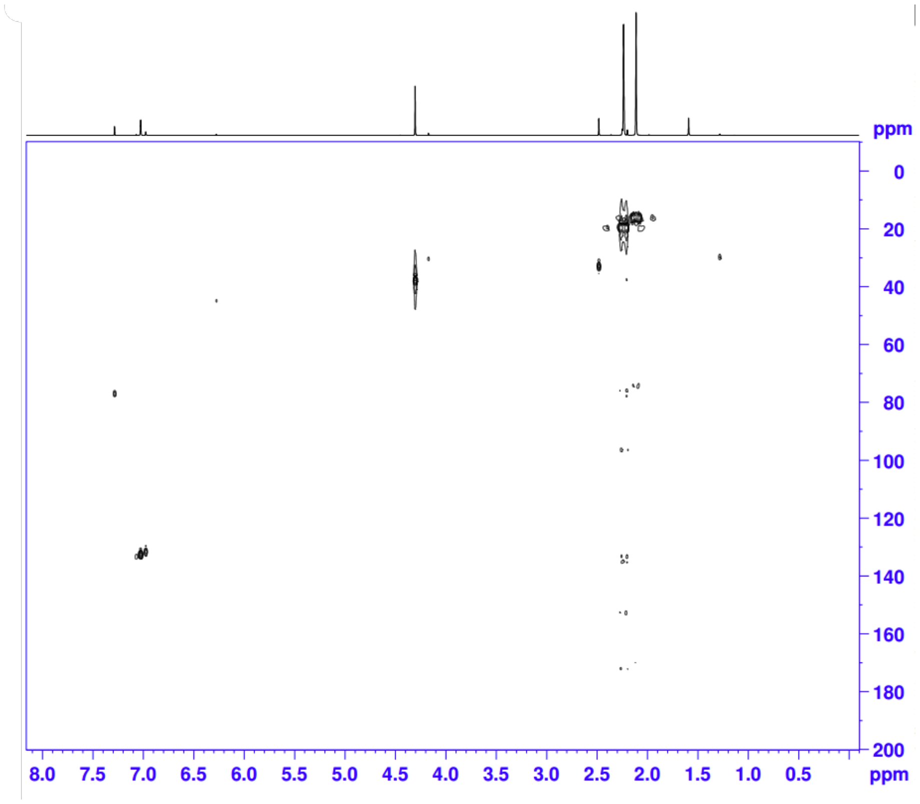

HMBC ($CDCl_3$, 500 MHz) of 2-bromo-1-(2,3,5,6-tetramethylphenyl)ethan-1-one.

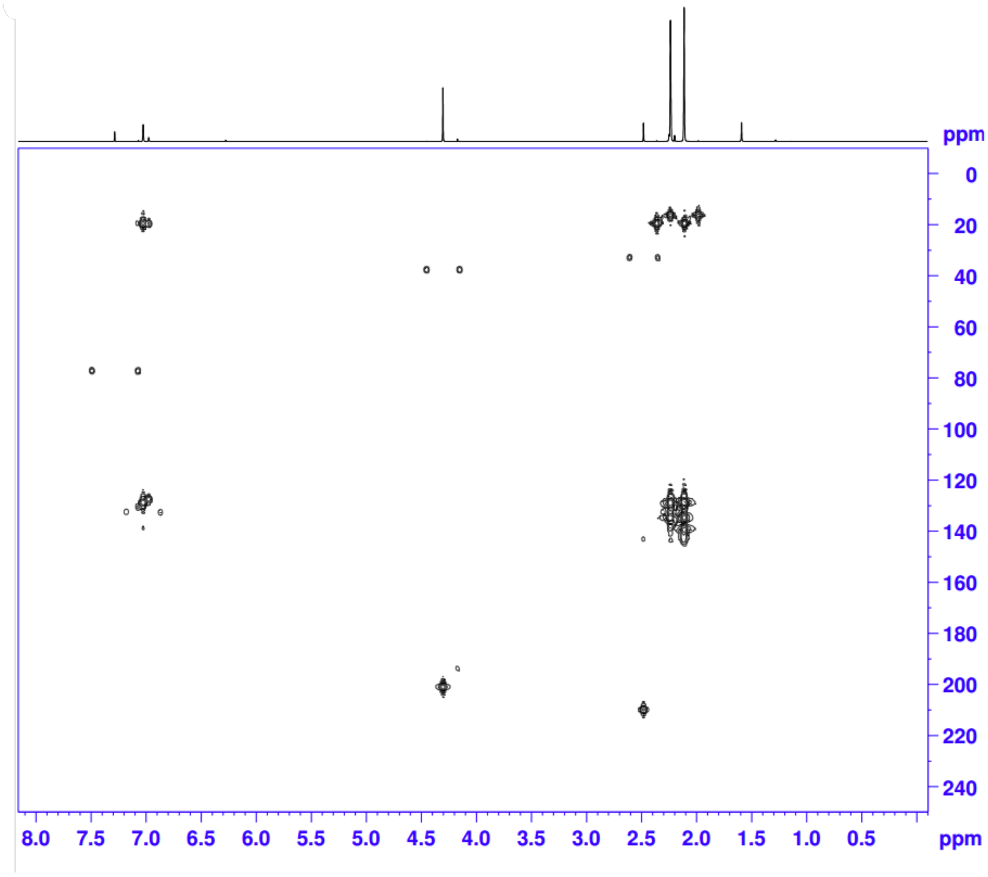

$^{13}C\{^{1}H\}$ NMR ($CDCl_3$, 500 MHz) of 2-bromo-1-(2,3,5,6-tetramethylphenyl)ethan-1-one.

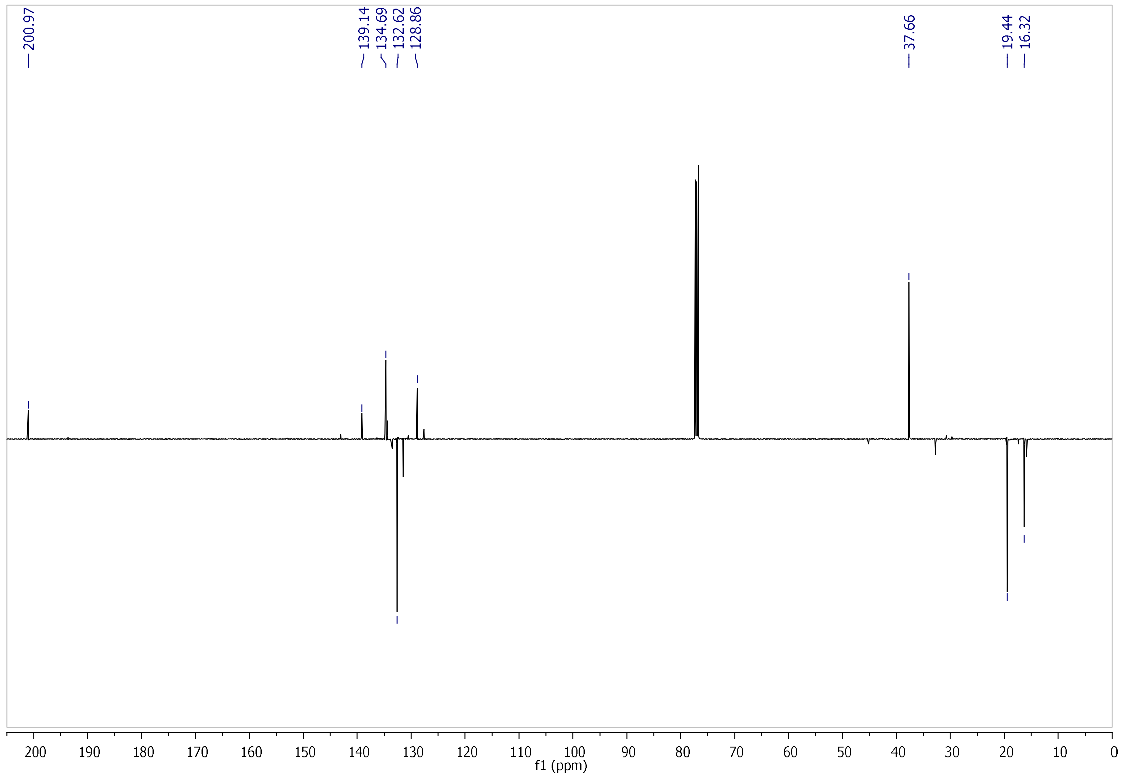

2-(4-Acetyl-2,3,5,6-tetramethylphenoxy)-1-phenylethan-1-one 40a.

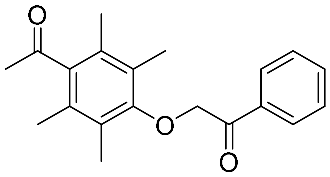

This compound is novel. To a dry 25 mL round-bottom flask containing a stirred solution of 1-(4-hydroxy-2,3,5,6-tetramethylphenyl)ethan-1-one (100 mg, 0.521 mmol) in acetone (5 mL), potassium carbonate (145 mg, 1.04 mmol) was added under a nitrogen atmosphere and the formed suspension was allowed to stir vigorously for 30 min at room temperature. Bromoacetophenone (155 mg, 0.780 mmol) dissolved in acetone (1 mL) was then added dropwise over 45 minutes, and the mixture was stirred at reflux for 24 h. After allowing to cool to room temperature, the mixture was quenched with water (20 mL), followed by addition of EtOAc (20 mL). The organic layer was separated, and the aqueous layer was extracted with EtOAc (2 x 20 mL). The combined organic layer was then dried ($MgSO_4$) and concentrated under reduced pressure to give the crude product which was purified using flash chromatography on silica gel eluted with 0-70% EtOAc in hexane to afford 2-(4-acetyl-2,3,5,6-tetramethylphenoxy)-1-phenylethan-1-one **40a** as a white solid (135 mg, 0.435 mmol, 84%). TLC: R_f ca 0.2 (9:1 hexane: EtOAc), UV active, strong $KMnO_4$. Mp 107.1 – 109.3 °C; HRMS (ESI) *m/z*: $[M+Na]^+$ Calcd for $C_{20}H_{22}NaO_3$ 333.1461; Found 333.1452 (error 2.6 ppm); ν_{max} 2983, 2921, 2900, 2851, 1695, 1596, 1576, 1228, 1168, 1119, 1081 cm^{-1}; ^1H NMR (400 MHz, $CDCl_3$): δ 7.98-7.94 (2H, m, ArH), 7.63 (1H, t, *J* 7.4, ArH), 7.51 (2H, t, *J* 7.7, ArH), 5.04 (2H, s, OCH_2), 2.49 (3H, s, $COCH_3$), 2.21 (6H, s, $COCCH_3$), 2.13 (6H, s, $COCCH_3$); $^{13}C\{^1H\}$ NMR (126 MHz, $CDCl_3$): δ 209.6 (C), 193.8 (C), 155.2 (C), 139.7 (C), 134.5 (C), 133. 8 (C), 129.4 (CH), 128.9 (CH), 127.8 (CH), 127.5 (C), 74.8 (CH_2), 33.0 (CH_3), 16.5 (CH_3), 12.5 (CH_3); *m/z* (ES-API+) 333.0 (M^+ + Na, 100%).

^{1}H NMR (400 MHz, $CDCl_3$) of 2-(4-acetyl-2,3,5,6-tetramethylphenoxy)-1-phenylethan-1-one **40a**.

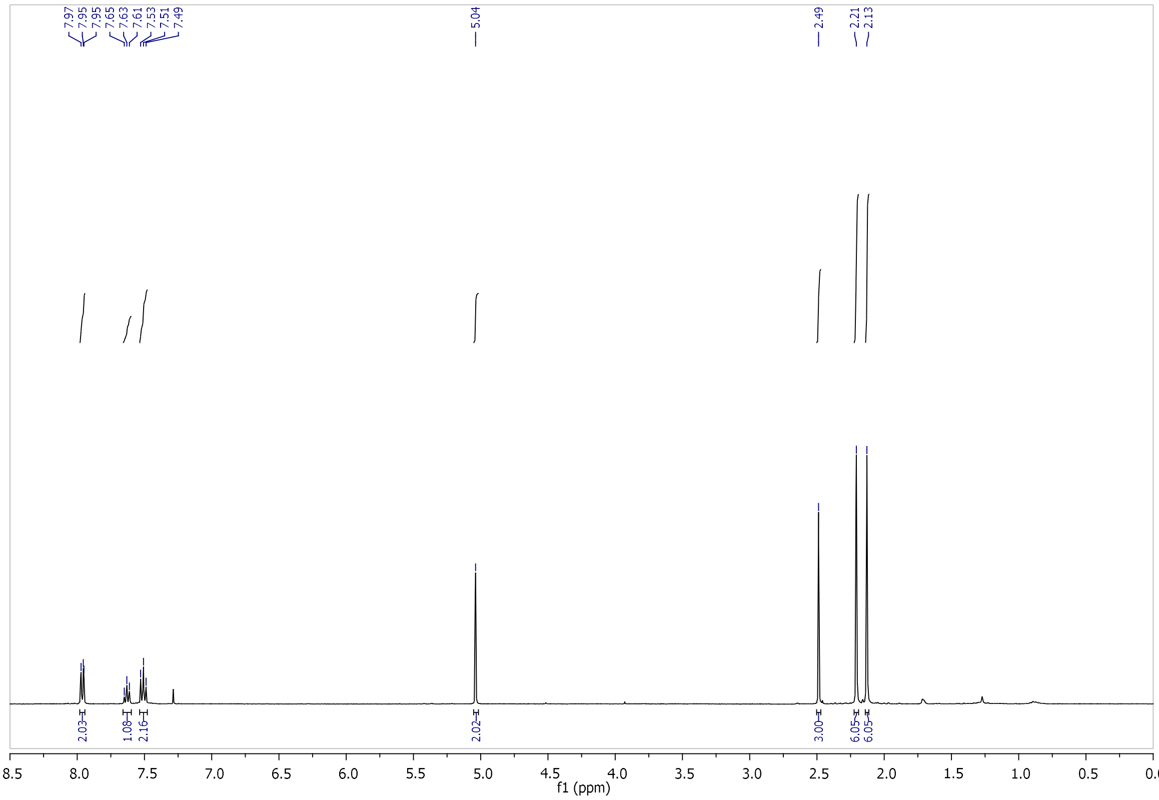

Expanded Aromatic Region (7.35-8.35 ppm) (400 MHz, $CDCl_3$)

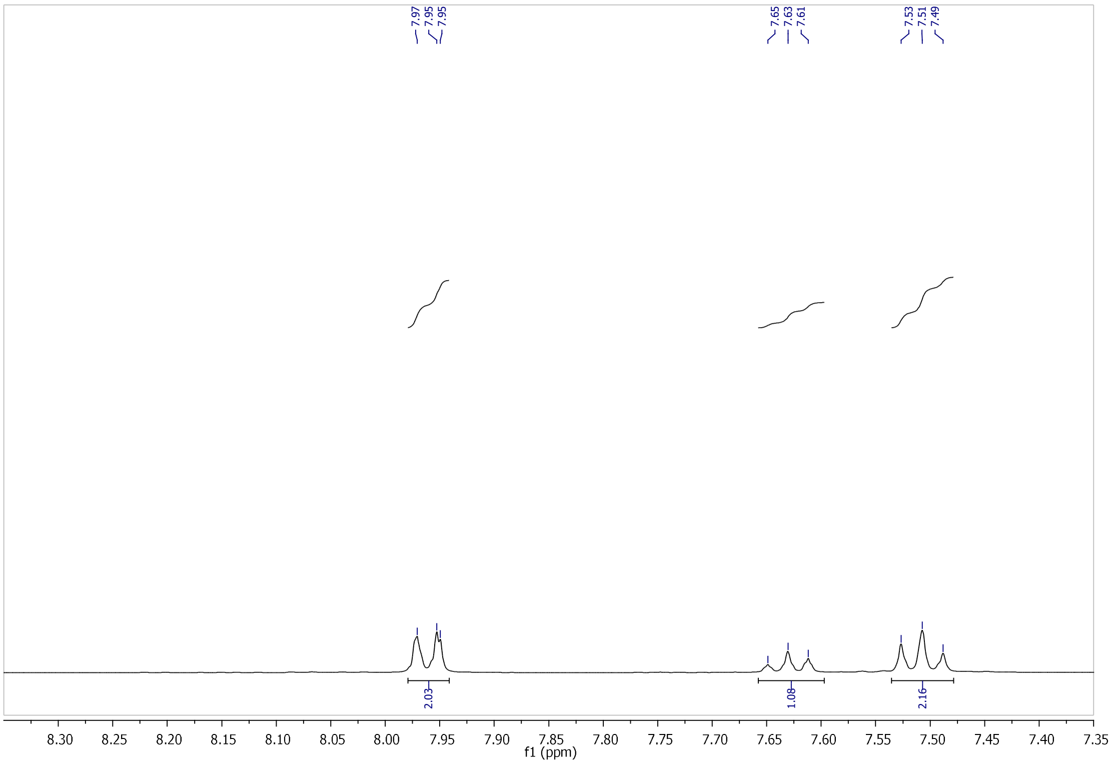

COSY (400 MHz, $CDCl_3$) of 2-(4-acetyl-2,3,5,6-tetramethylphenoxy)-1-phenylethan-1-one **40a**.

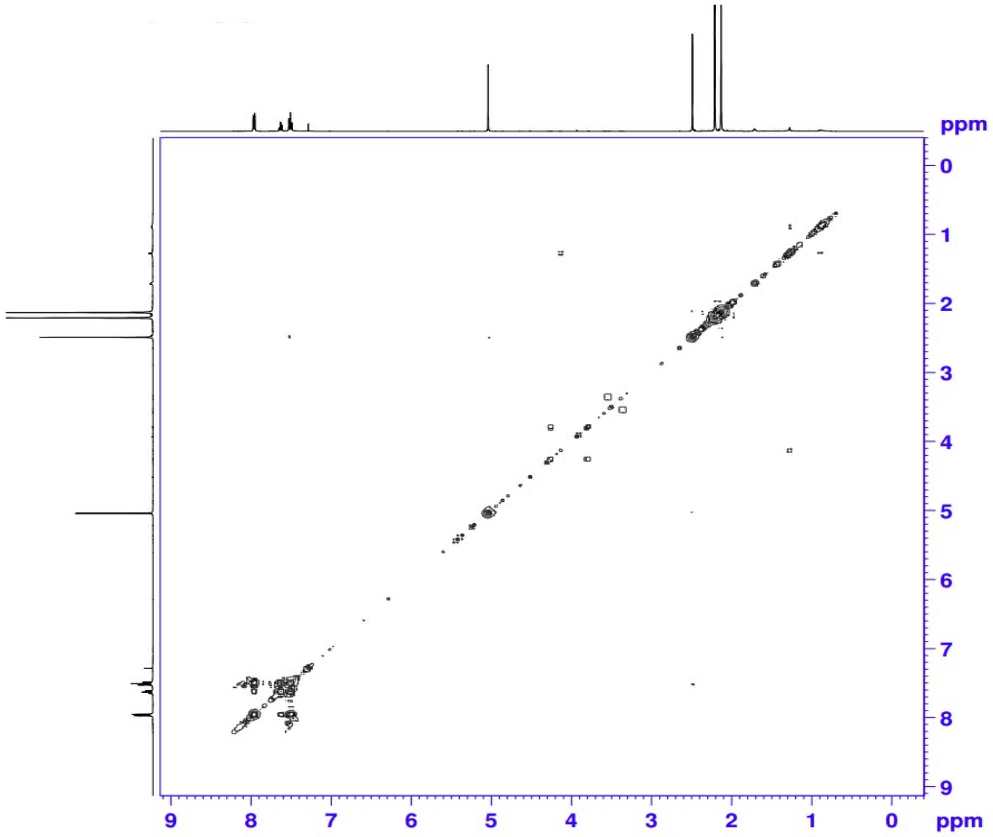

HSQC (400 MHz, $CDCl_3$) of 2-(4-acetyl-2,3,5,6-tetramethylphenoxy)-1-phenylethan-1-one **40a**.

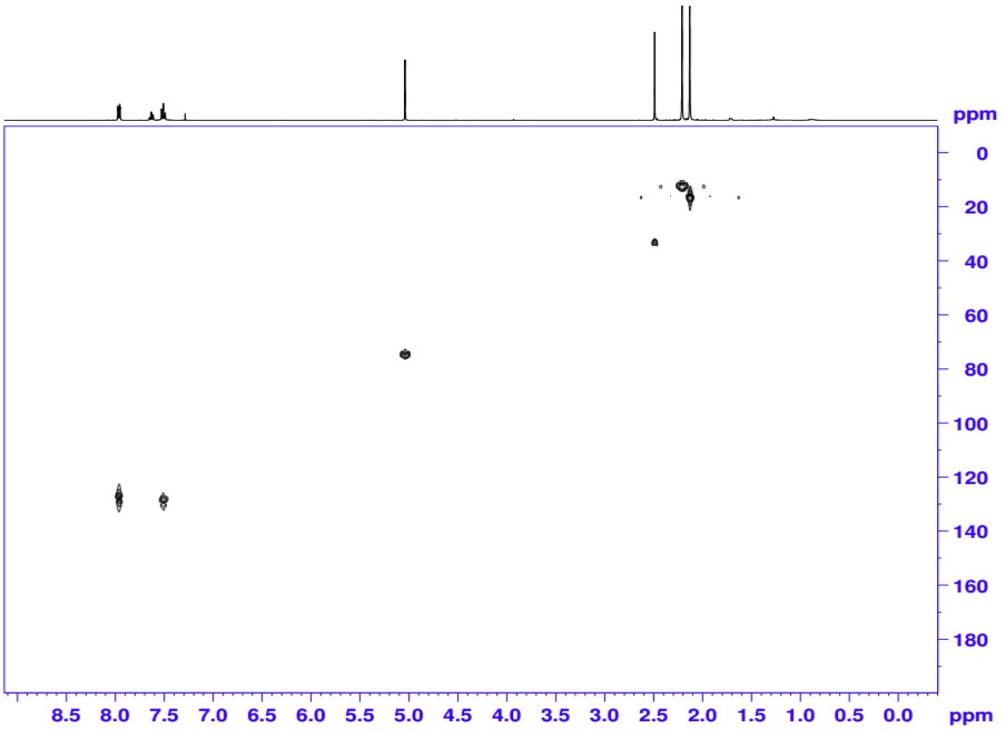

HMBC (400 MHz, $CDCl_3$) of 2-(4-acetyl-2,3,5,6-tetramethylphenoxy)-1-phenylethan-1-one **40a**.

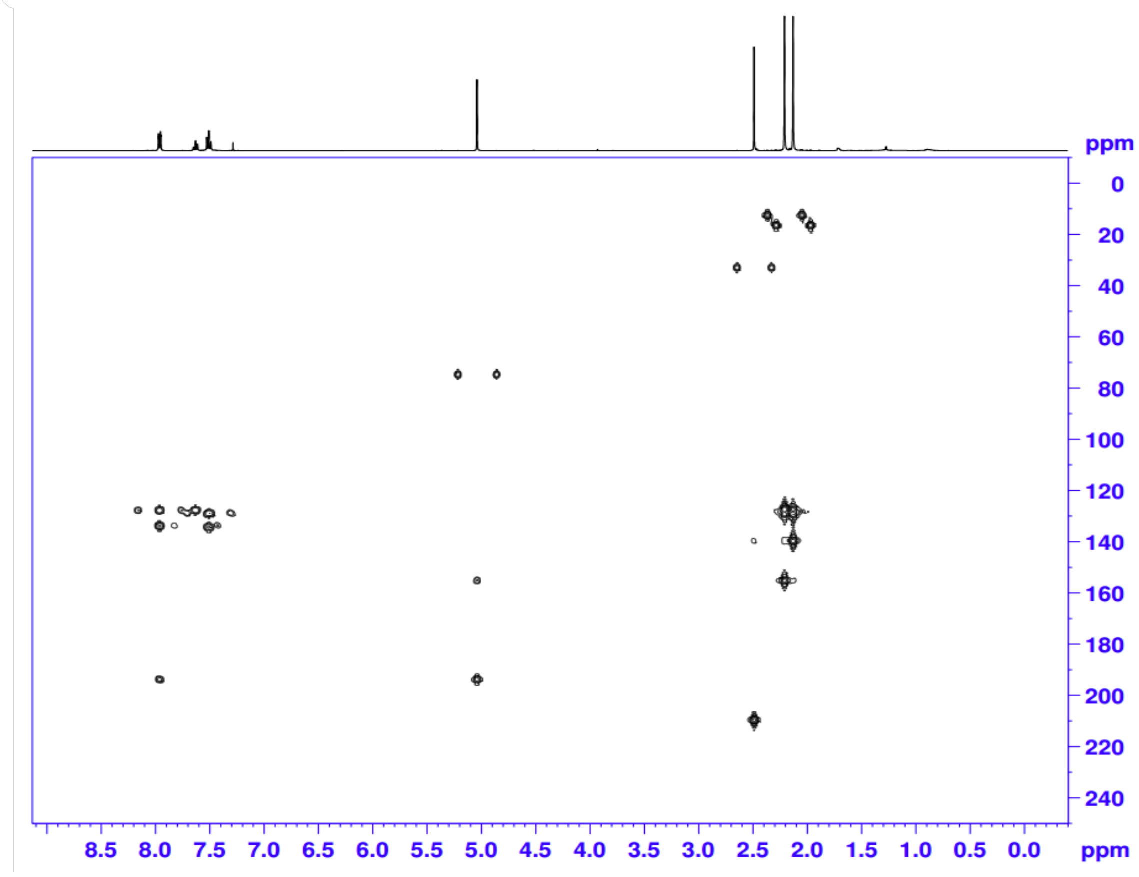

$^{13}C\{^1H\}$ NMR (400 MHz, $CDCl_3$) of 2-(4-acetyl-2,3,5,6-tetramethylphenoxy)-1-phenylethan-1-one **40a**.

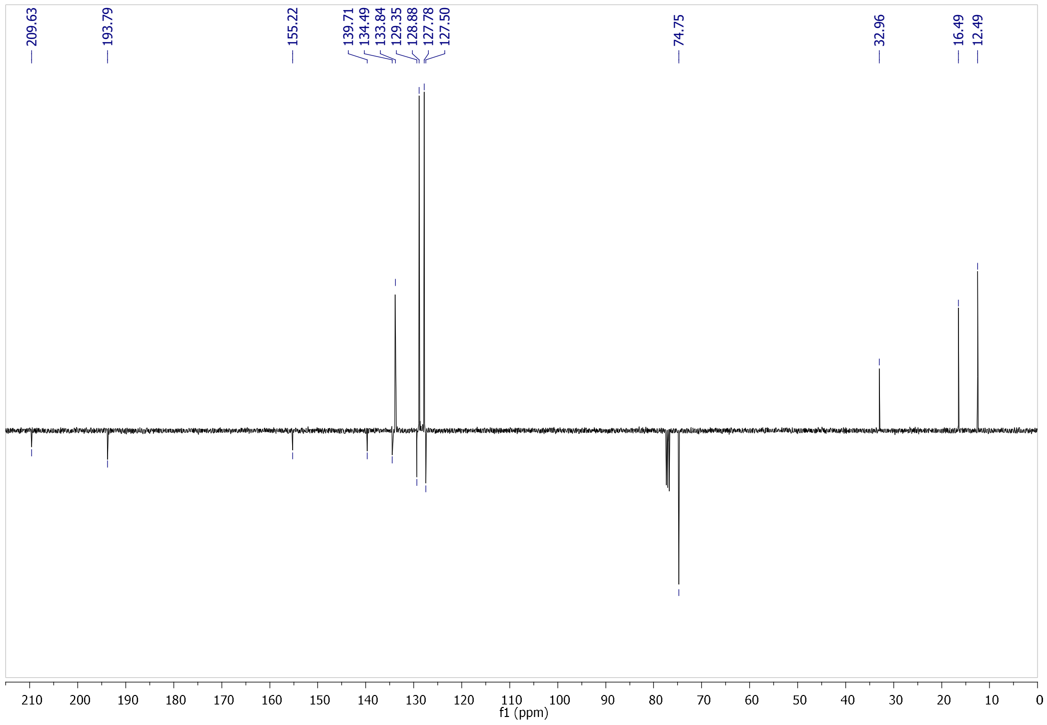

2-(4-Acetylphenoxy)-1-(2,3,5,6-tetramethylphenyl)ethan-1-one 41a.

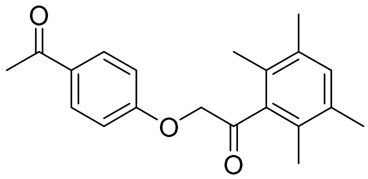

This compound is novel. To a dry 25 mL round-bottom flask containing a stirred solution of 4-hydroxyacetophenone (107 mg, 0.79 mmol) in acetone (5.5 mL), potassium carbonate (163 mg, 1.18 mmol) was added under nitrogen atmosphere and the formed suspension was allowed to stir vigorously for 30 min at room temperature. 2-Bromo-1-(2,3,5,6-tetramethylphenyl)ethan-1-one (300 mg, 1.18 mmol) dissolved in acetone (2 mL) was then added dropwise over 45 minutes, and the mixture was stirred at reflux for 24 h. After allowing to cool to room temperature, the mixture was quenched with water (20 mL), followed by addition of EtOAc (20 mL). The organic layer was separated, and the aqueous layer was extracted with EtOAc (2 x 20 mL). The combined organic layer was then dried (Mg_2SO_4) and concentrated under reduced pressure to give the crude product which was purified using flash chromatography on silica gel eluted with 0-70% EtOAc in hexane to afford 2-(4-acetylphenoxy)-1-(2,3,5,6-tetramethylphenyl)ethan-1-one **40a** as a white solid (59 mg, 0.19 mmol, 24%). TLC: R_f ca 0.4 (4:1 hexane: EtOAc), UV active, strong $KMnO_4$. Mp 107.9 – 111.2 °C; HRMS (ESI) *m/z*: $[M+Na]^+$ Calcd for $C_{20}H_{22}NaO_3$ 333.1461; Found 333.1451 (error 3.0 ppm); ν_{max} 2969, 2949, 2922, 2858, 1725, 1672, 1596, 1468, 1262, 1224, 1174 cm^{-1}; ^1H NMR (500 MHz, $CDCl_3$): δ 7.97 (2H, d, *J* 8.9, ArH), 7.05 (1H, s, ArH), 7.01 (2H, d, *J* 8.9, ArH), 4.93 (2H, s, OCH_2), 2.59 (3H, s, $COCH_3$), 2.25 (6H, s, $COCCH_3$), 2.16 (6H, s, CCH_3); $^{13}C\{^1H\}$ NMR (126 MHz, $CDCl_3$): δ 204.7 (C), 196.7 (C), 161.6 (C), 138.4 (C), 134.7 (C), 132.7 (CH), 131.2 (C), 130.7 (CH), 129.1 (C), 114.6 (CH), 73.6 (CH_2), 26.4 (CH_3), 19.4 (CH_3), 16.2 (CH_3); *m/z* (ES-API+) 333.0 (M^+ + Na, 100%).

^{1}H NMR ($CDCl_3$, 500 MHz) of 2-(4-acetylphenoxy)-1-(2,3,5,6-tetramethylphenyl)ethan-1-one **41a**.

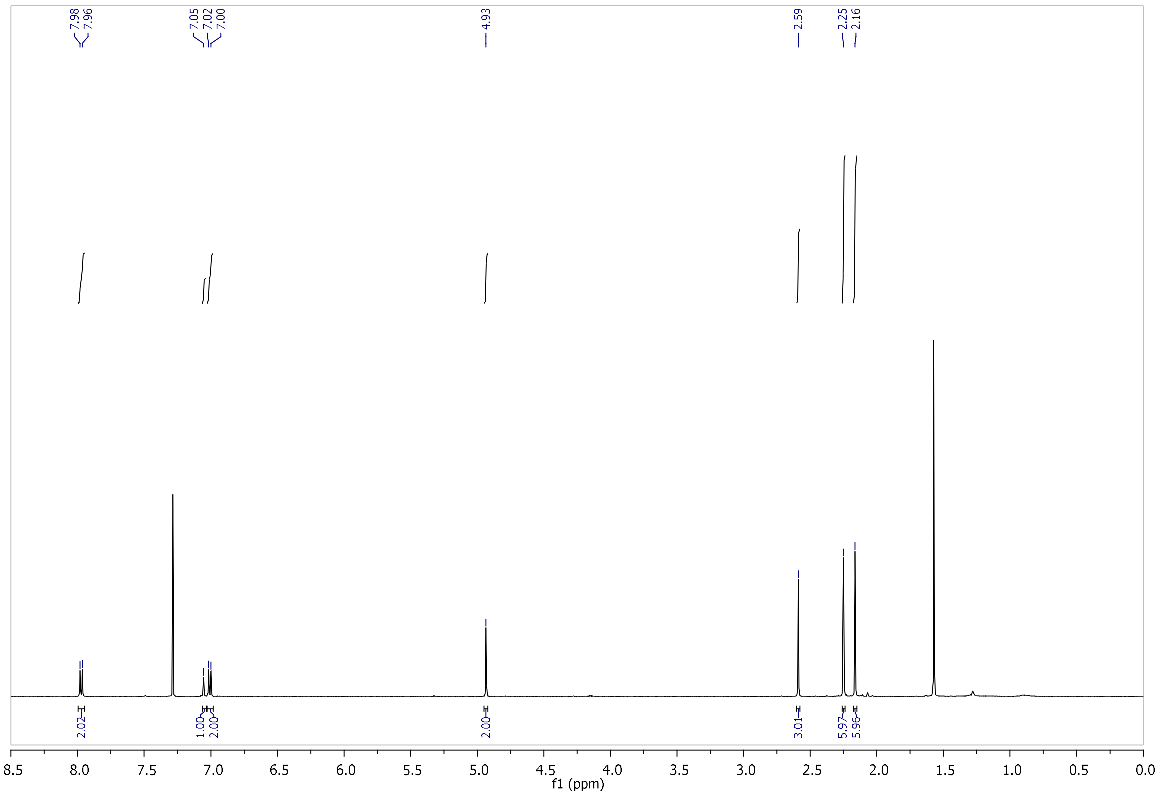

Expanded Aromatic Region (7.50-8.25 ppm) ($CDCl_3$, 500 MHz).

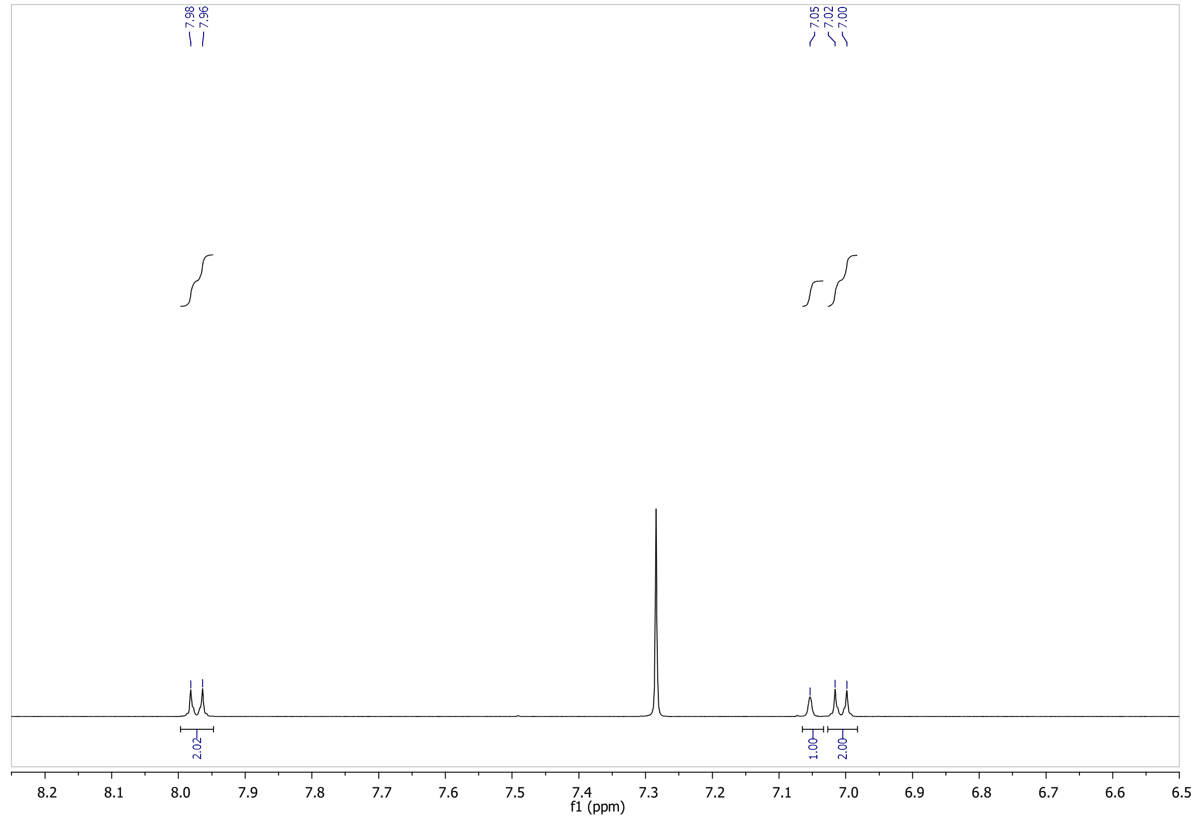

COSY ($CDCl_3$, 500 MHz) of 2-(4-acetylphenoxy)-1-(2,3,5,6-tetramethylphenyl)ethan-1-one **41a**.

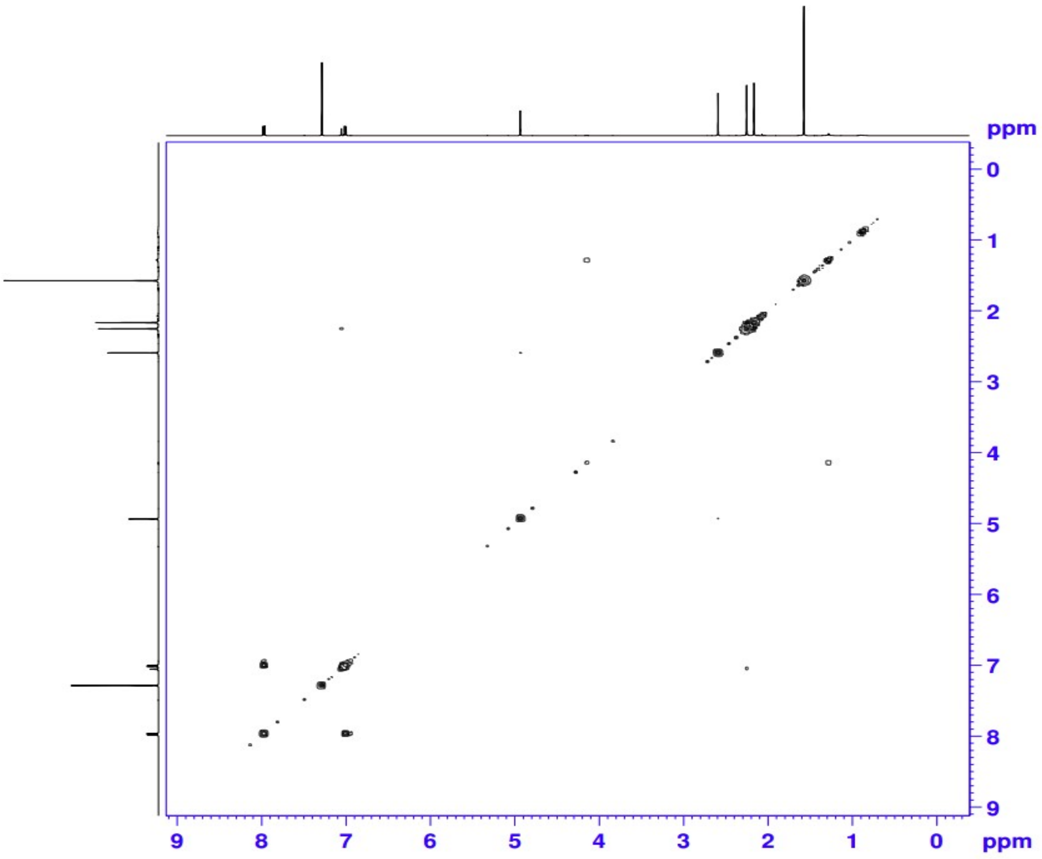

HSQC ($CDCl_3$, 500 MHz) of 2-(4-acetylphenoxy)-1-(2,3,5,6-tetramethylphenyl)ethan-1-one **41a**.

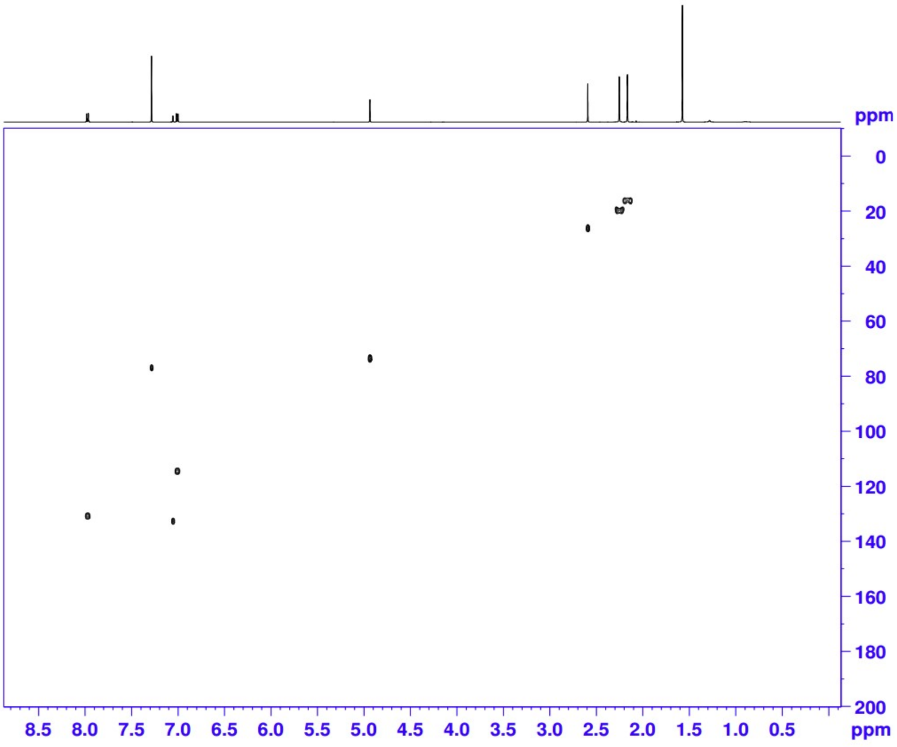

HMBC ($CDCl_3$, 500 MHz) of 2-(4-acetylphenoxy)-1-(2,3,5,6-tetramethylphenyl)ethan-1-one **41a**.

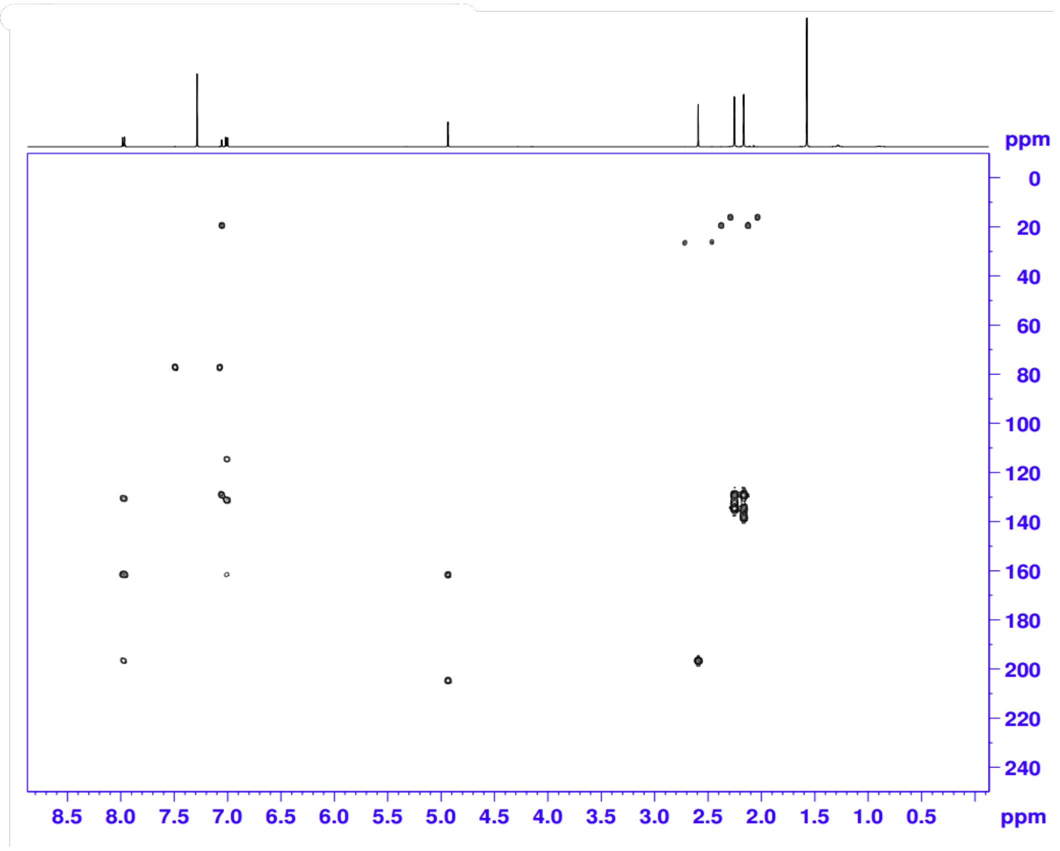

$^{13}C\{^1H\}$ NMR ($CDCl_3$, 500 MHz) of 2-(4-acetylphenoxy)-1-(2,3,5,6-tetramethylphenyl)ethan-1-one **41a**.

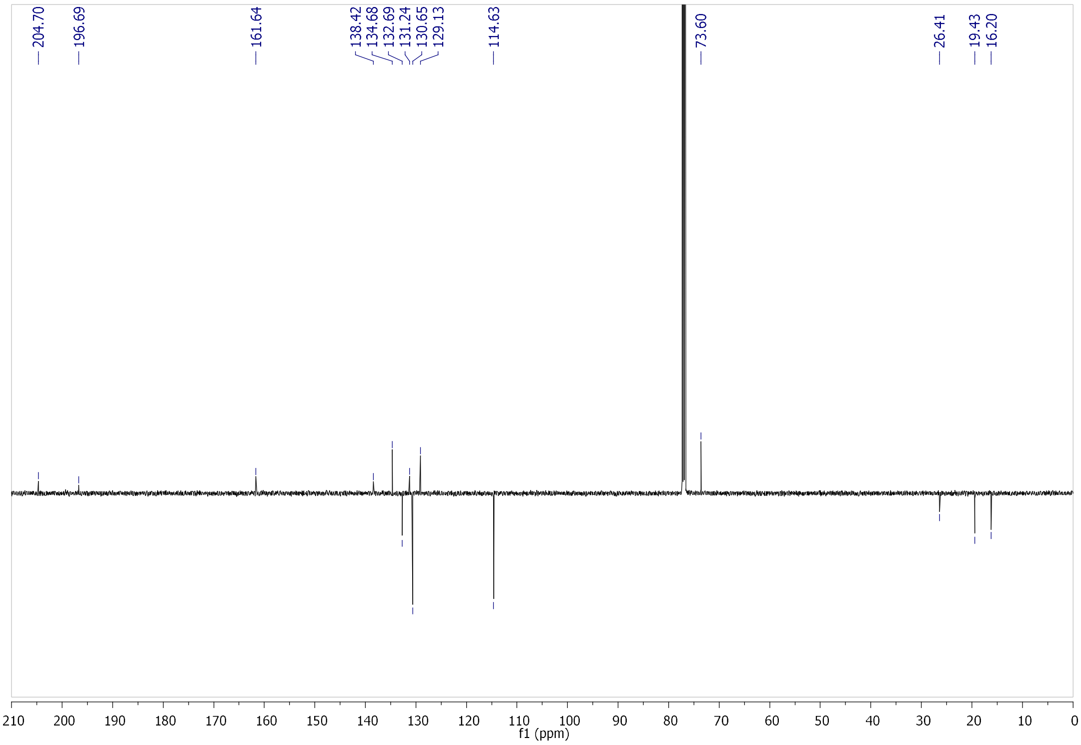

(*R*)-1-(4-(2-Hydroxy-2-phenylethoxy)-2,3,5,6-tetramethylphenyl)ethan-1-one 40b.

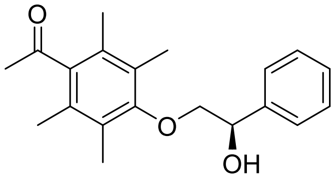

This compound is novel. A FA/TEA solution (0.07 mL) was added to a 50 mL Schlenk under nitrogen atmosphere containing 1 mol% (*S,S*)-Ru-3C-Teth (2.7 mg, 0.0044 mmol) and the mixture was stirred for 10 min to allow for the catalyst to be activated. 2-(4-Acetyl-2,3,5,6-tetramethylphenoxy)-1-phenylethan-1-one **40a** (135 mg, 0.0.435 mmol) was dissolved in DCM (0.22 mL) and added to the mixture which was allowed to stir for 48 h. Following flash chromatography on silica gel using DCM/ 1-5 % MeOH, (*R*)-1-(4-(2-hydroxy-2-phenylethoxy)-2,3,5,6-tetramethylphenyl)ethan-1-one **40b** was afforded as a white solid (118 mg, 0.378 mmol, 87%). TLC: R_f ca 0.6 (1:1 hexane: EtOAc), UV active (faint), faint $KMnO_4$; Mp 115.7 – 117.0 °C; HRMS (ESI) *m/z*: $[M+Na]^+$ Calcd for $C_{20}H_{24}NaO_3$ 335.1618; Found 335.1611 (error 1.9 ppm); ν_{max} 3351, 2918, 2855, 1697, 1351, 1313, 1194, 1168, 1077, 1062 cm^{-1}; $[\alpha]_D^{22}$ = 12.5 (c=0.04, $CHCl_3$, T = 20°C); ^1H NMR (500 MHz, $CDCl_3$): δ 7.35-7.19 (5H, m, ArH), 5.09 (1H, dd, *J* 8.1 3.8, C*H*(OH)), 3.76-3.68 (2H, m, CH_2), 2.37 (3H, s, CH_3CO), 2.10 (6H, s, (6H, s, $COCCH_3$), 2.02 (6H, s, CCH_3); $^{13}C\{^1H\}$ NMR (126 MHz, $CDCl_3$): δ 209.6 (C), 154.8 (C), 139.8 (C), 139.5 (C), 129.2 (C), 128.5 (CH), 128.1 (CH), 127.5 (C), 126.2 (CH), 73.4 (CH), 64.1 (CH_2) 32.9 (CH_3), 16.5 (CH_3), 12.5 (CH_3); *m/z* (ES-API+) 335.1 (M^+ + Na, 100%). Enantiomeric excess and conversion determined by HPLC analysis (Chiralcel ODH, 30 cm x 6 mm column, iPrOH:hexane 1:9, 1 mL/min, T = 25 °C) ketone 10.2 min, *S* isomer 11.0 min, *R* isomer 12.6 min. 97% ee (*R*). The configuration of the chiral centrers are based on precedents for the catalysts used.

^{1}H NMR ($CDCl_3$, 500 MHz) of 1-(4-(2-hydroxy-2-phenylethoxy)-2,3,5,6-tetramethylphenyl)ethan-1-one **40b**.

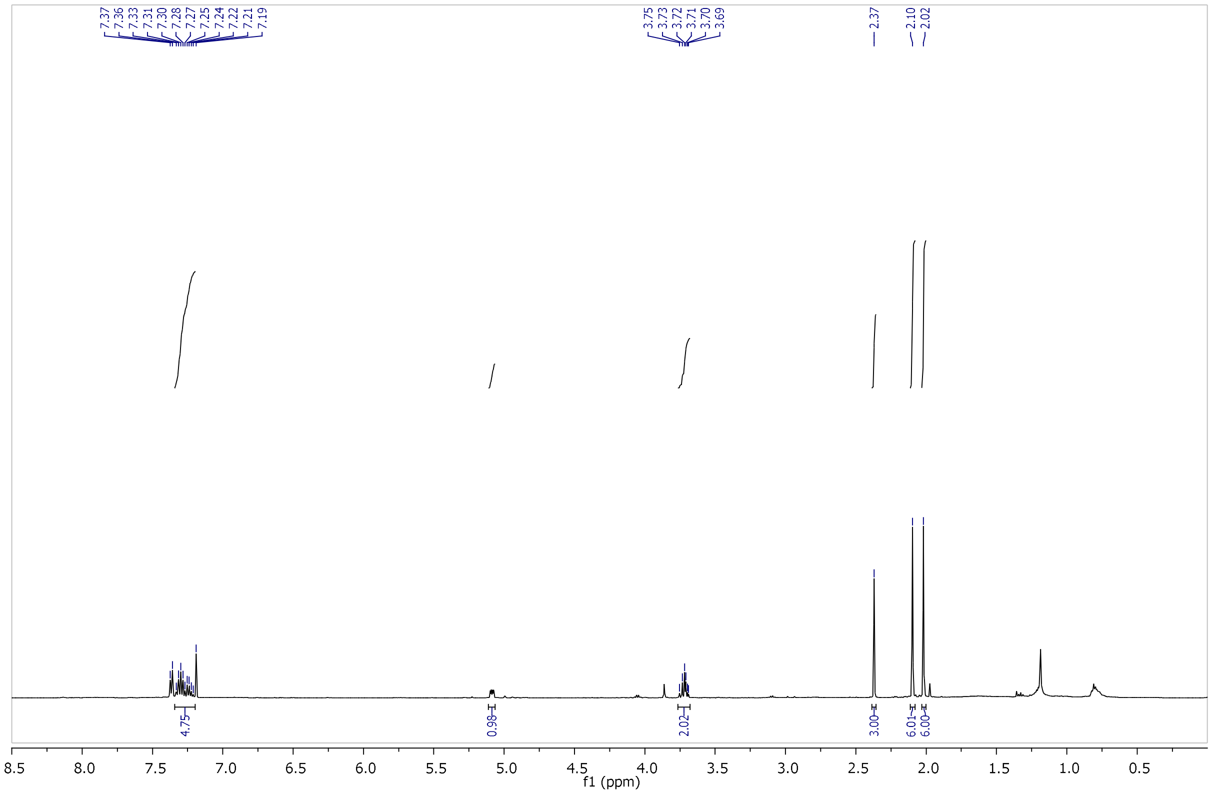

COSY ($CDCl_3$, 500 MHz) of 1-(4-(2-hydroxy-2-phenylethoxy)-2,3,5,6-tetramethylphenyl)ethan-1-one **40b**.

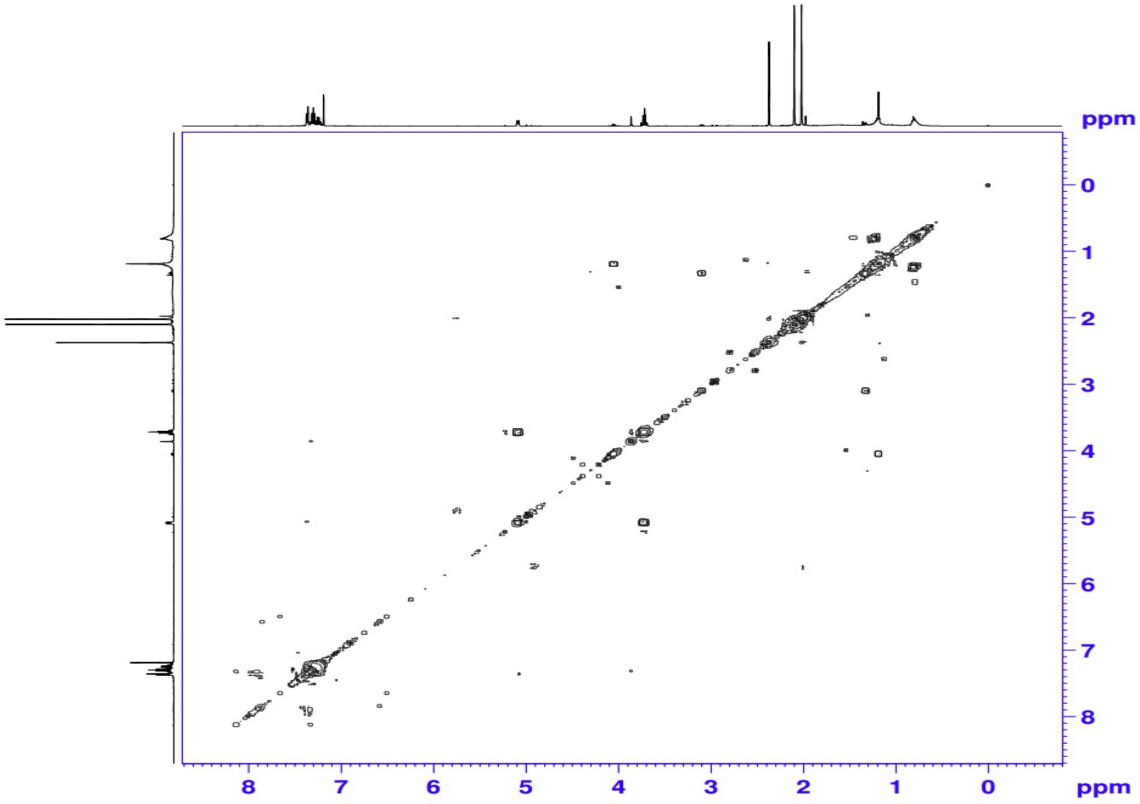

HSQC ($CDCl_3$, 500 MHz) of 1-(4-(2-hydroxy-2-phenylethoxy)-2,3,5,6-tetramethylphenyl)ethan-1-one **40b**.

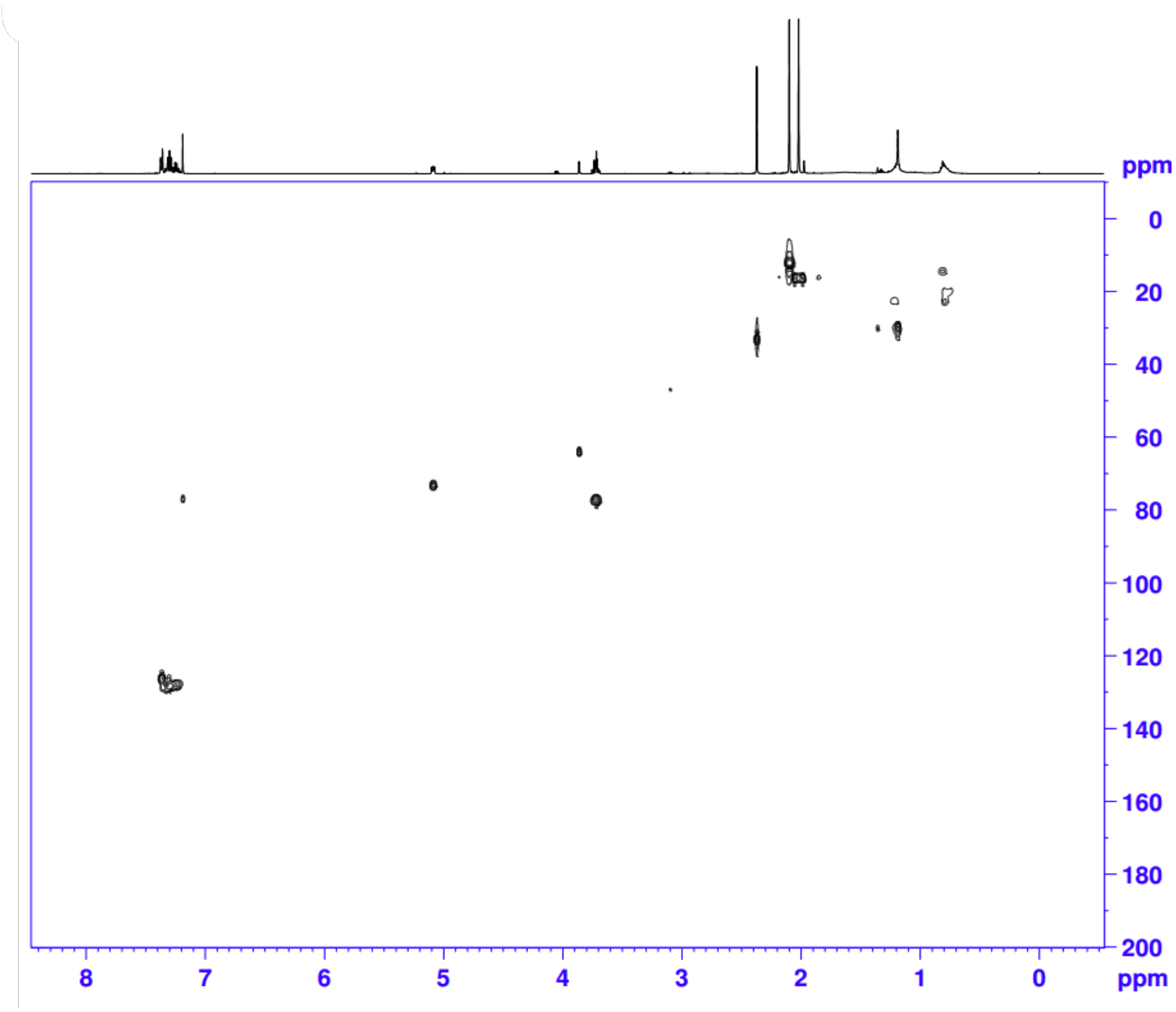

HMBC ($CDCl_3$, 500 MHz) of 1-(4-(2-hydroxy-2-phenylethoxy)-2,3,5,6-tetramethylphenyl)ethan-1-one **40b**.

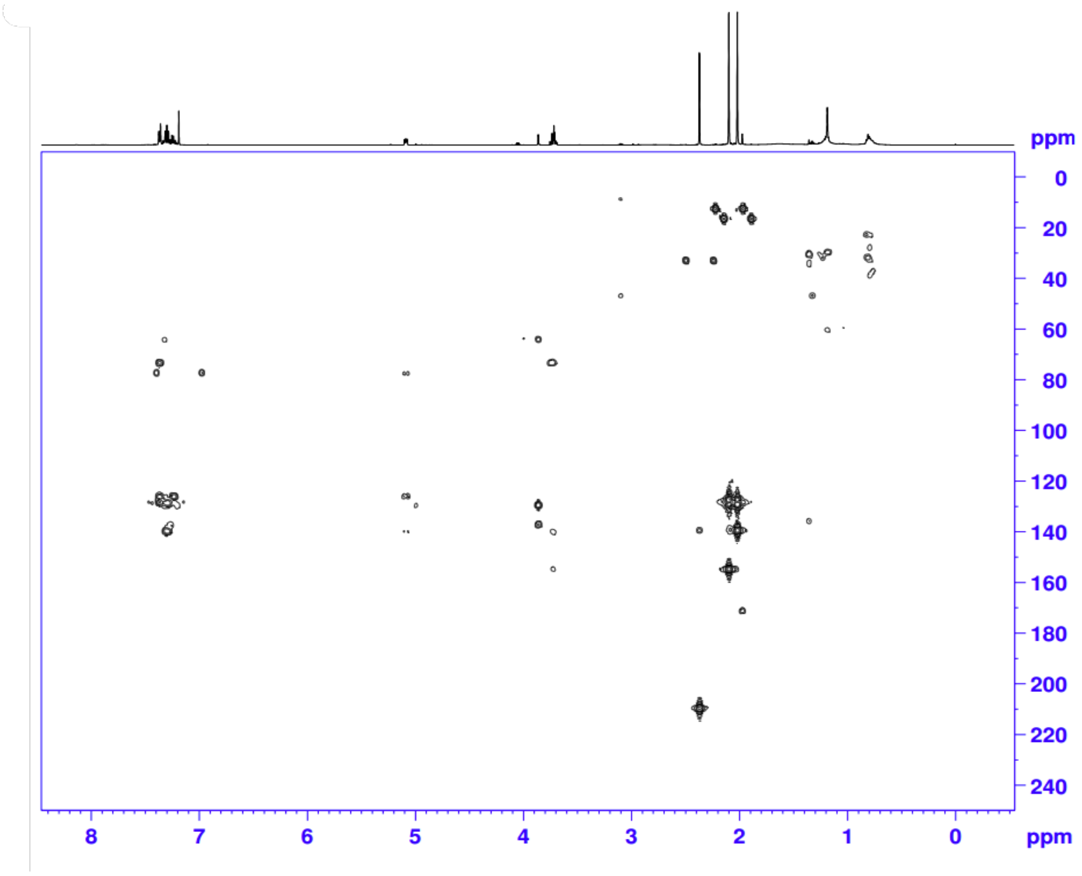

$^{13}C\{^{1}H\}$ NMR ($CDCl_3$, 500 MHz) of 1-(4-(2-hydroxy-2-phenylethoxy)-2,3,5,6-tetramethylphenyl)ethan-1-one **40b**.

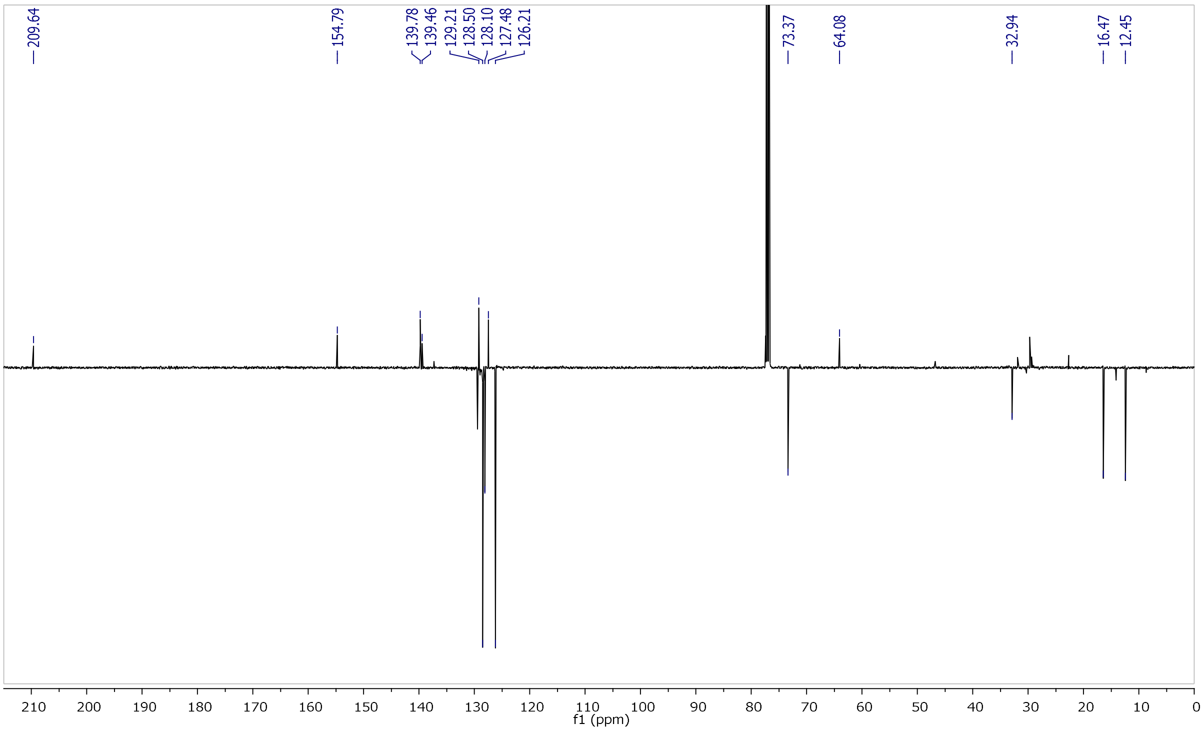

HPLC of (*R*)-1-(4-(2-hydroxy-2-phenylethoxy)-2,3,5,6-tetramethylphenyl)ethan-1-one **40b**. (*S,S*)-3C-tethered Ru(II)-TsDPEN catalyst (after 48 h, 100% conversion, 97% ee (*R*))

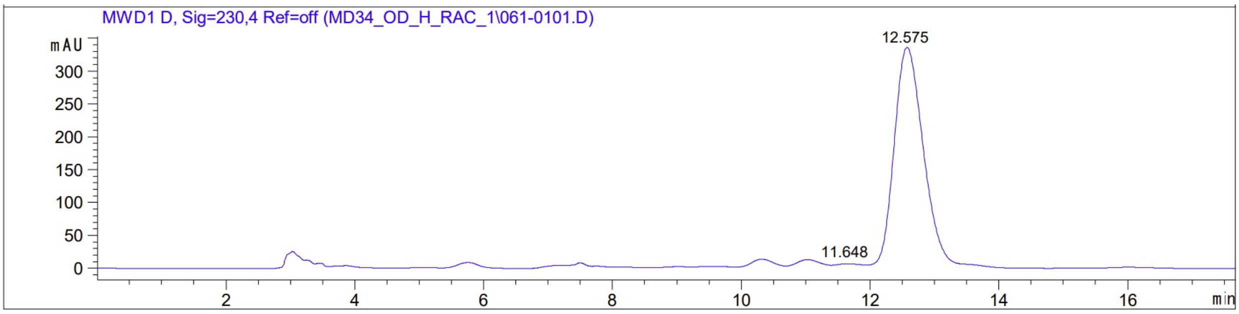

Signal 4: MWD1 D, Sig=230,4 Ref=off

Peak #	RetTime [min]	Type	Width [min]	Area [mAU*s]	Height [mAU]	Area %
1	11.648	VV	0.3862	157.06958	5.79557	1.4424
2	12.575	VB	0.4951	1.07322e4	335.55243	98.5576
Totals :				1.08893e4	341.34800	

X-ray Crystallography Data for (*R*)-40b. CCDC Deposition Number 2324377.

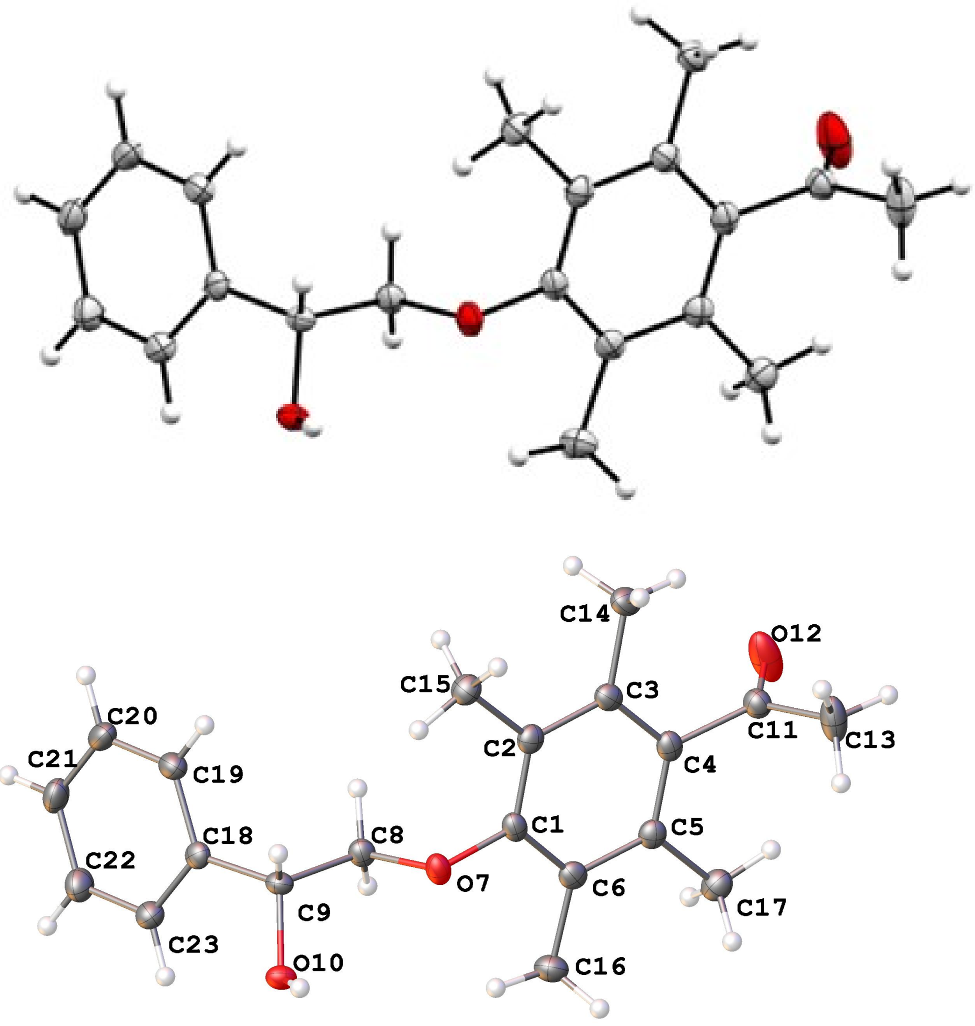

*Solid state ORTEP structure of (R)-**40b** with atoms unlabelled (above) and labelled (below) and thermal ellipsoids drawn at 50% probability level.*

Experimental.

Single crystals of $C_{20}H_{24}O_3$ (*R*)-**40b** were prepared by recrystallization from methanol. A suitable crystal was selected and mounted on a XtaLAB Synergy, Dualflex, HyPix-Arc **100** diffractometer. The crystal was kept at 100.00(19) K during data collection. Using Olex2 [1], the structure was solved with the SHELXT [2] structure solution program using Intrinsic Phasing and refined with the SHELXL [3] refinement package using Least Squares minimization.

1. Dolomanov, O.V., Bourhis, L.J., Gildea, R.J, Howard, J.A.K. & Puschmann, H. (2009), J. Appl. Cryst. 42, 339-341.

2. Sheldrick, G.M. (2015). Acta Cryst. A71, 3-8.

3. Sheldrick, G.M. (2015). Acta Cryst. C71, 3-8.

Crystal structure determination of (*R*)-40b.

Crystal Data for $C_{20}H_{24}O_3$ (*M*=312.39 g/mol): monoclinic, space group $P2_1$ (no. 4), a = 13.63062(18) Å, *b* = 4.86210(4) Å, c = 14.32493(19) Å, b = 117.7282(17)°, *V* = 840.34(2) Å^3, *Z* = 2, *T* = 100.00(19) K, μ(Cu Ka) = 0.649 mm^{-1}, *Dcalc* = 1.235 g/cm^3, 25632 reflections measured (6.972° < 2q < 154.516°), 3395 unique (R_{int} = 0.0163, R_{sigma} = 0.0079) which were used in all calculations. The final R_1 was 0.0257 (I > 2s(I)) and wR_2 was 0.0688 (all data).

Refinement model description

Number of restraints - 1, number of constraints - unknown.
Details:

1. Fixed Uiso

At 1.2 times of: All C(H) groups, All C(H,H) groups

At 1.5 times of: All C(H,H,H) groups

2.a Ternary CH refined with riding coordinates: C9(H9)

2.b Secondary CH2 refined with riding coordinates:
C8(H8A,H8B)

2.c Aromatic/amide H refined with riding coordinates:
C19(H19), C23(H23), C22(H22), C21(H21), C2O(H20)

2.d Idealised Me refined as rotating group:

C17(H17A,H17B,H17C), C16(H16A,H16B,H16C), C1S(H15A,H15B,H15C), C14(H14A,H14B, H14C), C13(H13A, H13B,H13C)

This report was created with Olex2, compiled on 2023.08.24 svn.relec1418 for OlexSys, and in part reformatted for the Supporting Information.

Table 1 Crystal data and structure refinement for *(R)*-40b.

Identification code	exp_40b_auto
Empirical formula	$C_{20}H_{24}O_{3}$
Formula weight	312.39
Temperature/K	100.00(19)
Crystal system	Monoclinic
Space group	$P2_1$
a/Å	13.63062(18)
b/Å	4.86210(4)
c/Å	14.32493(19)
α/°	90
β/°	117.7282(17)
γ/°	90
Volume/$Å^3$	840.34(2)
Z	2
$P_{calc}g/cm^3$	1.235
$μ/mm^{-1}$	0.649
F(000)	336.0
Crystal size/mm^3	0.5 x 0.3 x 0.05
Radiation	Cu Ka (λ = 1.54184)
20 range for data collection/°	6.972 to 154.516
Index ranges	-16 <h< 17, -6 <k < 6, -17 <1< 17
Reflections collected	25632
Independent reflections	3395 [R_{int} = 0.0163, R_{sigma} = 0.0079]
Data/restraints/parameters	3395/1/217
Goodness-of-fit on F^2	1.076
Final R indexes [I>=2σ (I)]	R_1 = 0.0257, wR_2 = 0.0685
Final R indexes [all data]	R_1 = 0.0261, wR_2 = 0.0688
Largest duff. peak/hole / $eÅ^3$	0.17/-0.18
Flack parameter	0.01(3)

Table 2 Fractional Atomic Coordinates (×10[4]) and Equivalent Isotropic Displacement Parameters (Å[2]×10[3]) for (*R*)-40b. U_{eq} is defined as 1/3 of the trace of the orthogonalised U_{IJ} tensor.

Atom	*x*	*y*	*z*	U(eq)
O(10)	56.3(8)	5864(2)	4565.9(8)	19.1(2)
O(7)	2405.3(8)	6383(2)	5884.8(8)	20.9(2)
O(12)	7468.2(10)	2476(3)	8630.1(10)	43.9(4)
C(9)	839.4(11)	6003(3)	4165.8(11)	17.1(3)
C(18)	410.8(11)	4484(3)	3124.5(11)	18.0(3)
C(11)	6895.3(12)	4171(3)	8746.1(11)	21.8(3)
C(19)	917.3(12)	4965(4)	2490.3(12)	23.3(3)
C(1)	3508.7(11)	5714(3)	6558.1(11)	18.5(3)
C(5)	4855.0(12)	3352(3)	8097.2(11)	19.2(3)
C(23)	-431.7(12)	2545(4)	2811.4(11)	21.7(3)
C(2)	4337.3(12)	6968(3)	6392.5(11)	18.9(3)
C(8)	1934.6(11)	4723(3)	4947.7(11)	19.2(3)
C(17)	5126.6(13)	1381(4)	8999.6(12)	24.5(3)
C(6)	3739.9(12)	3869(3)	7382.3(11)	18.8(3)
C(4)	5694.1(12)	4620(3)	7944.7(11)	19.3(3)
C(3)	5449.0(12)	6364(3)	7086.9(11)	19.3(3)
C(22)	-739.3(13)	1050(4)	1885.7(12)	27.1(3)
C(21)	-217.3(13)	1505(4)	1267.9(12)	27.3(3)
C(16)	2822.7(12)	2454(4)	7503.1(12)	25.0(3)
C(20)	604.0(12)	3482(4)	1567.0(12)	27.3(4)
C(15)	4051.4(13)	8962(3)	5497.8(12)	22.9(3)
C(14)	6354.6(12)	7647(4)	6905.0(12)	24.5(3)
C(13)	7359.0(14)	6043(4)	9682.8(13)	34.0(4)

Table 3 Anisotropic Displacement Parameters (Å^2×10^3) for (*R*)-40b. The Anisotropic displacement factor exponent takes the form: $-2\pi^2[h^2a^{*2}U_{11}+2hka^*b^*U_{12}+\ldots]$.

Atom	U_{11}	U_{22}	U_{33}	U_{23}	U_{13}	U_{12}
O(10)	18.9(5)	19.6(5)	22.9(5)	-1.8(4)	13.2(4)	-0.5(4)
O(7)	16.0(4)	24.7(6)	18.6(5)	-3.9(4)	5.3(4)	3.6(4)
O(12)	24.6(6)	51.7(8)	41.1(7)	-21.6(7)	3.3(5)	13.4(6)
C(9)	15.7(6)	18.1(7)	19.6(6)	0.8(6)	10.0(5)	-0.4(5)
C(18)	16.0(6)	20.0(7)	16.4(6)	3.5(6)	6.4(5)	4.2(6)
C(11)	19.0(7)	24.2(8)	21.1(7)	-0.4(6)	8.4(6)	1.7(6)
C(19)	17.8(7)	30.2(8)	21.6(7)	2.9(6)	9.0(6)	-0.7(6)
C(1)	15.6(6)	20.4(7)	17.5(6)	-4.1(6)	6.1(5)	2.1(6)
C(5)	20.8(7)	18.3(7)	18.7(6)	-2.5(6)	9.4(5)	2.4(6)
C(23)	21.1(7)	25.1(8)	19.5(7)	1.6(6)	9.8(5)	-0.7(6)
C(2)	21.3(7)	18.2(7)	16.9(6)	-3.3(5)	8.5(5)	0.1(6)
C(8)	17.7(6)	21.0(7)	18.8(6)	-2.9(6)	8.4(5)	1.5(6)
C(17)	26.3(7)	24.9(8)	23.0(7)	2.0(6)	12.0(6)	4.6(7)
C(6)	19.9(7)	18.5(7)	19.4(7)	-4.8(6)	10.3(5)	0.7(6)
C(4)	18.3(6)	20.0(7)	17.8(6)	-4.8(6)	7.0(5)	2.2(6)
C(3)	19.1(6)	19.5(7)	20.1(6)	-4.9(6)	9.9(5)	-1.6(6)
C(22)	26.3(7)	29.9(9)	21.6(7)	-3.2(7)	8.1(6)	-5.8(7)
C(21)	26.1(7)	36.4(9)	16.1(6)	-3.8(6)	7.1(6)	2.5(7)
C(16)	22.3(7)	27.5(8)	29.1(8)	0.5(7)	15.3(6)	-0.4(7)
C(20)	22.1(7)	43.2(10)	17.9(7)	4.0(7)	10.5(6)	4.4(7)
C(15)	25.2(7)	21.7(8)	21.7(7)	1.8(6)	10.8(6)	1.4(6)
C(14)	22.3(7)	28.6(8)	24.0(7)	-5.9(7)	11.9(6)	-6.1(6)
C(13)	26.6(8)	36.1(10)	25.1(8)	-8.8(8)	0.0(6)	9.7(8)

Table 4 Bond Lengths for (*R*)-40b.

Atom	Atom	Length/Å	Atom	Atom	Length/Å
O(10)	C(9)	1.4289(15)	C(1)	C(6)	1.397(2)
O(7)	C(1)	1.3958(16)	C(5)	C(17)	1.511(2)
O(7)	C(8)	1.4363(18)	C(5)	C(6)	1.4049(19)
O(12)	C(11)	1.199(2)	C(5)	C(4)	1.403(2)
C(9)	C(18)	1.5168(19)	C(23)	C(22)	1.394(2)
C(9)	C(8)	1.5212(19)	C(2)	C(3)	1.4033(19)
C(18)	C(19)	1.393(2)	C(2)	C(15)	1.507(2)
C(18)	C(23)	1.389(2)	C(6)	C(16)	1.506(2)
C(11)	C(4)	1.5156(19)	C(4)	C(3)	1.400(2)
C(11)	C(13)	1.496(2)	C(3)	C(14)	1.510(2)
C(19)	C(20)	1.388(2)	C(22)	C(21)	1.387(2)
C(1)	C(2)	1.397(2)	C(21)	C(20)	1.383(3)

Table 5 Bond Angles for (*R*)-40b.

Atom	Atom	Atom	Angle/°	Atom	Atom	Atom	Angle/°
C(1)	O(7)	C(8)	112.59(11)	C(18)	C(23)	C(22)	119.89(13)
O(10)	C(9)	C(18)	110.71(11)	C(1)	C(2)	C(3)	118.69(13)
O(10)	C(9)	C(8)	110.22(11)	C(1)	C(2)	C(15)	121.03(12)
C(18)	C(9)	C(8)	108.79(12)	C(3)	C(2)	C(15)	120.28(13)
C(19)	C(18)	C(9)	118.60(13)	O(7)	C(8)	C(9)	108.49(12)
C(23)	C(18)	C(9)	122.03(12)	C(1)	C(6)	C(5)	118.22(13)
C(23)	C(18)	C(19)	119.30(13)	C(1)	C(6)	C(16)	121.17(13)
O(12)	C(11)	C(4)	122.53(14)	C(5)	C(6)	C(16)	120.60(14)
O(12)	C(11)	C(13)	120.78(14)	C(5)	C(4)	C(11)	119.37(13)
C(13)	C(11)	C(4)	116.64(13)	C(3)	C(4)	C(11)	118.99(13)
C(20)	C(19)	C(18)	120.55(14)	C(3)	C(4)	C(5)	121.60(13)
O(7)	C(1)	C(2)	118.51(13)	C(2)	C(3)	C(14)	119.41(14)
O(7)	C(1)	C(6)	118.71(13)	C(4)	C(3)	C(2)	119.14(13)
C(6)	C(1)	C(2)	122.76(12)	C(4)	C(3)	C(14)	121.43(13)
C(6)	C(5)	C(17)	119.24(13)	C(21)	C(22)	C(23)	120.47(15)
C(4)	C(5)	C(17)	121.27(13)	C(20)	C(21)	C(22)	119.65(15)
C(4)	C(5)	C(6)	119.47(13)	C(21)	C(20)	C(19)	120.12(14)

Table 6 Hydrogen Atom Coordinates (Å×10^4) and Isotropic Displacement Parameters (Å^2×10^3) for (*R*)-40b.

Atom	*x*	*y*	*z*	U(eq)
H(9)	966.29	7974.43	4054.58	20
H(19)	1481.96	6318.7	2691.48	28
H(23)	-797.66	2236.74	3227.16	26
H(8A)	1810.31	2827.39	5121.89	23
H(8B)	2447.74	4640.26	4636.41	23
H(17A)	4749.65	1971.92	9404.61	37
H(17B)	5929.22	1363.26	9459.59	37
H(17C)	4876.99	-471.41	8720.17	37
H(22)	-1310.35	-288.46	1676.64	33
H(21)	-422.72	465.57	642.88	33
H(16A)	2104.93	3152.53	6970.17	37
H(16B)	2904.25	2820.69	8208.43	37
H(16C)	2861.97	467.33	7409.48	37
H(20)	954.18	3824.34	1139.51	33
H(15A)	4286.61	8202.13	5000.1	34
H(15B)	4432.77	10711.86	5777.04	34
H(15C)	3248.71	9270.24	5134.25	34
H(14A)	6184.42	7396.54	6166.14	37
H(14B)	7063.73	6761.5	7363.57	37
H(14C)	6403.32	9615.92	7067.18	37
H(13A)	8104.35	5425.25	10185.58	51
H(13B)	6878.99	6009.07	10022.05	51
H(13C)	7397.24	7922.19	9454.07	51
H(10)	54(19)	7360(60)	4833(18)	40(6)

Racemic 1-(4-(2-Hydroxy-2-phenylethoxy)-2,3,5,6-tetramethylphenyl)ethan-1-one 40b.

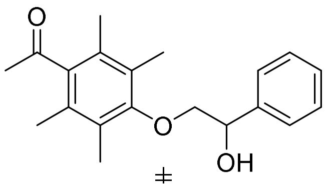

This compound is novel. To a 25 mL round-bottom flask containing 2-(4-acetyl-2,3,5,6-tetramethylphenoxy)-1-phenylethan-1-one (10 mg, 0.030 mmol) dissolved in methanol (0.9 mL) and water (0.1 mL), sodium borohydride (3.4 mg, 0.09 mmol) was added and the mixture was stirred for 96 h. The solvent was removed at the end of this time and EtOAc (20 mL) and water (20 mL) was added. The organic layer was separated and the water layer was extracted with further EtOAc (2 x 20 mL). the combined organic extracts were dried ($MgSO_4$) and filtered and the solvent was removed under vacuum. Crude 1-(4-(2-hydroxy-2-phenylethoxy)-2,3,5,6-tetramethylphenyl)ethan-1-one was afforded as a white solid (7.0 mg, 0.02 mmol, 74%). The NMR spectrum contained signals corresponding to the major enantiomer formed in the asymmetric reduction, although the HPLC analysis revealed the presence of two enantiomers formed in a 1:1 ratio. This indicates that both product enantiomers have overlapping NMR signals.

HPLC of racemic 1-(4-(2-hydroxy-2-phenylethoxy)-2,3,5,6-tetramethylphenyl)ethan-1-one **40b**.

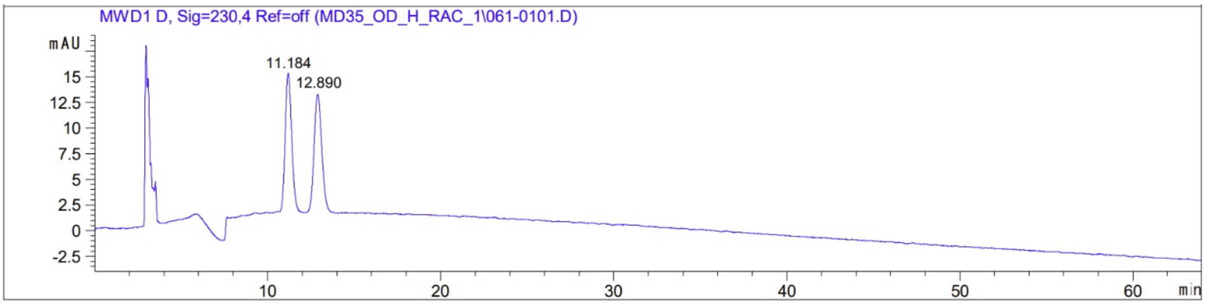

Signal 4: MWD1 D, Sig=230,4 Ref=off

Peak #	RetTime [min]	Type	Width [min]	Area [mAU*s]	Height [mAU]	Area %
1	11.184	BB	0.4042	352.25183	13.50865	49.3981
2	12.890	BB	0.4745	360.83585	11.54880	50.6019
Totals :				713.08768	25.05745	

(*S*)-2-(4-(1-Hydroxyethyl)phenoxy)-1-(2,3,5,6-tetramethylphenyl)ethan-1-one 41b.

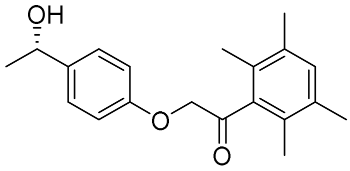

This compound is novel. A FA/TEA solution (0.07 mL) was added to a 50 mL Schlenk under nitrogen atmosphere containing 1 mol% (*S,S*)-Ru-3C-Teth (0.80 mg, 0.0013 mmol) and the mixture was stirred for 10 min to allow for the catalyst to be activated. 2-(4-Acetylphenoxy)-1-(2,3,5,6-tetramethylphenyl)ethan-1-one **41a** (40 mg, 0.13 mmol) was dissolved in DCM (0.6 mL) and added to the mixture which was allowed to stir for 96 h. (*S*)-2-(4-(1-Hydroxyethyl)phenoxy)-1-(2,3,5,6-tetramethylphenyl)ethan-1-one **41b** was afforded as a white solid (31 mg, 0.10 mmol, 77%). TLC: R_f ca 0.6 (1:1 hexane: EtOAc), UV active (faint), strong $KMnO_4$; Mp 99.8 – 101.4 °C; HRMS (ESI) *m/z*: $[M+Na]^+$ Calcd for $C_{20}H_{24}NaO_3$ 335.1618; Found 335.1610 (error 2.3 ppm); ν_{max} 3449, 2966, 2923, 1696, 1219, 1179, 1090,1065 cm^{-1}; $[\alpha]_D^{22}$ = 75 (c = 0.5, $CHCl_3$, T = 24 °C); ^{1}H NMR (500 MHz, $CDCl_3$): δ 7.33 (2H, d, *J* 8.7, ArH), 7.04 (1H, s, ArH), 6.95 (2H, d, *J* 8.7, ArH), 4.91-4.88 (1H, m, C*H*OH quartet overlapping with OCH_2 singlet), 4.87 (1H, s, OCH_2), 2.24 (6H, s, $COCCH_3$), 2.15 (6H, s, CCH_3), 1.50 (3H, d, *J* 6.4, C*H*$_3$COH); $^{13}C\{^1H\}$ NMR (126 MHz, $CDCl_3$): δ 205.7 (C), 157.3 (C), 139.2 (C), 138.8 (C), 134.6 (C), 132.5 (CH), 129.2 (C), 126.8 (CH), 115.1 (CH), 74.1 (CH_2), 69.9 (CH), 25.1 (CH_3), 19.4 (CH_3), 16.2 (CH_3); *m/z* (ES-API+) 335.1 (M^+ + Na, 100%). Enantiomeric excess and conversion determined by HPLC analysis (Chiralcel ODH, 30 cm x 6 mm column, iPrOH:hexane 1:9, 1 mL/min, T = 25 ºC) ketone 15.0 min, *R* isomer 17.7 min, *S* isomer 18.9 min. 99% ee (*S*). The configuration of the chiral centers are based on precedents for the catalysts used.

^1H NMR ($CDCl_3$, 500 MHz) of 2-(4-(1-hydroxyethyl)phenoxy)-1-(2,3,5,6-tetramethylphenyl)ethan-1-one **41b**.

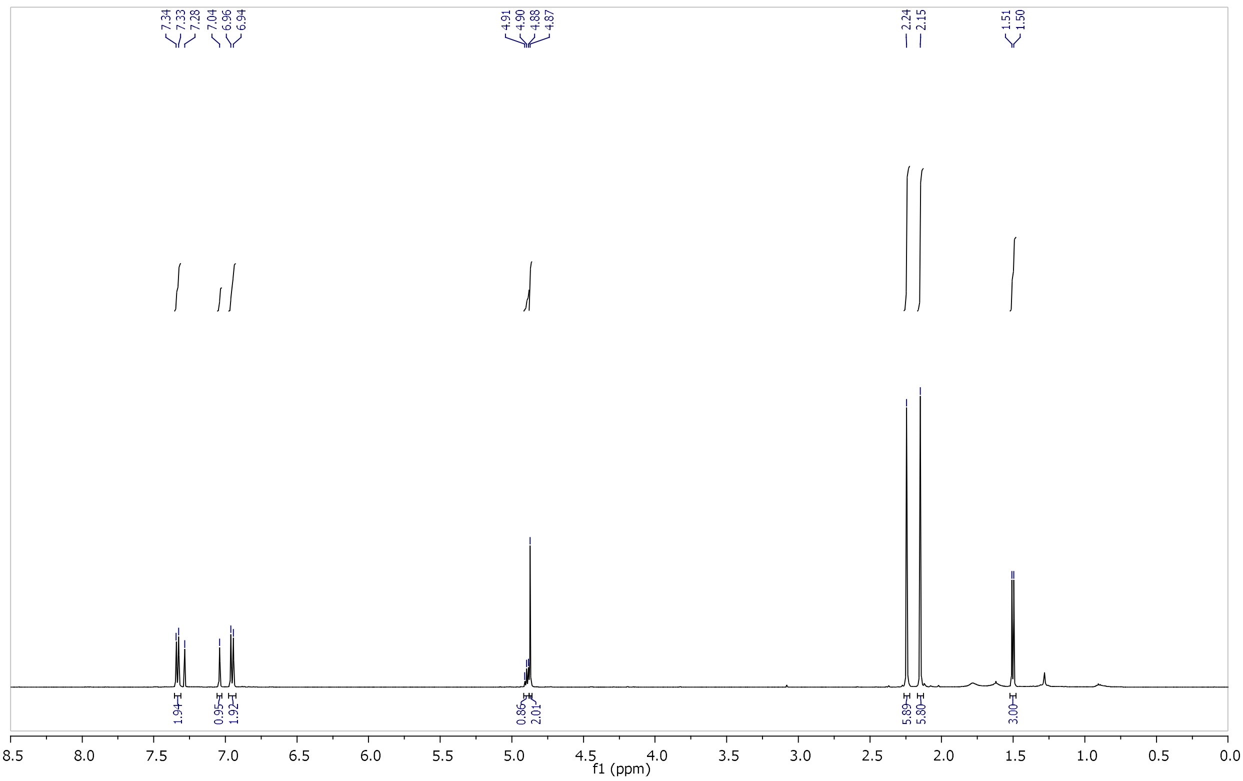

COSY ($CDCl_3$, 500 MHz) of 2-(4-(1-hydroxyethyl)phenoxy)-1-(2,3,5,6-tetramethylphenyl)ethan-1-one **41b**.

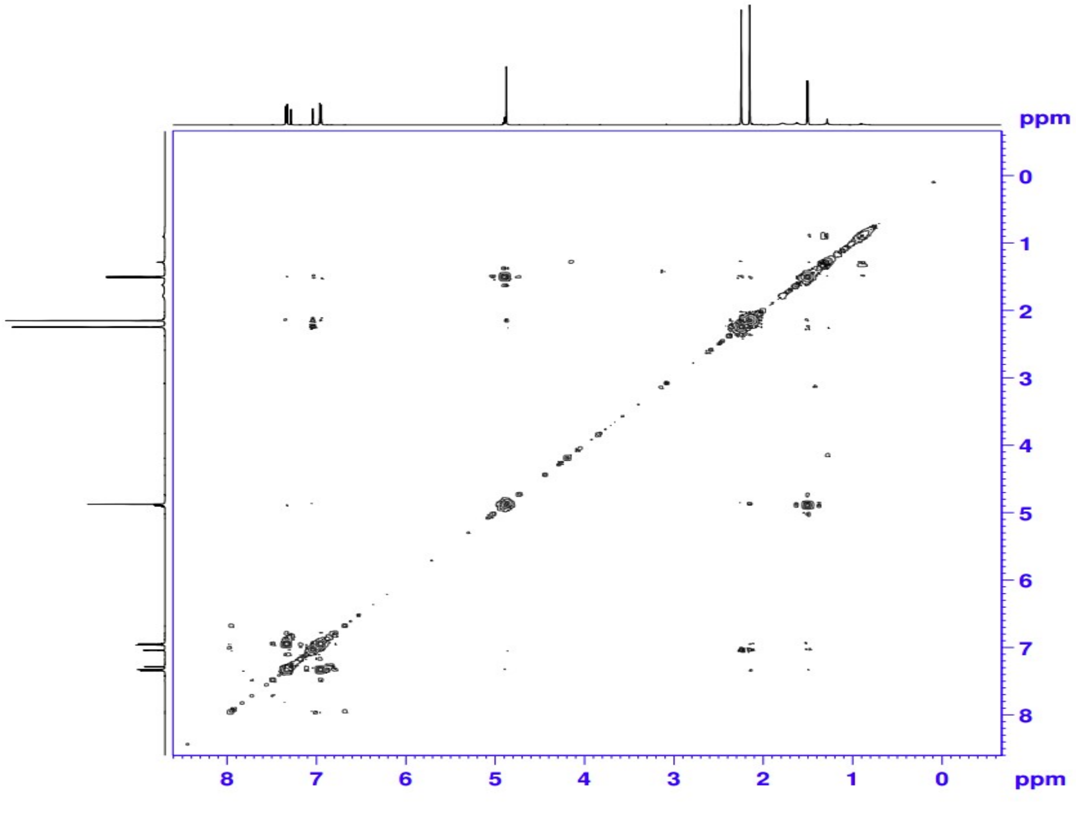

HSQC ($CDCl_3$, 500 MHz) of 2-(4-(1-hydroxyethyl)phenoxy)-1-(2,3,5,6-tetramethylphenyl)ethan-1-one **41b**.

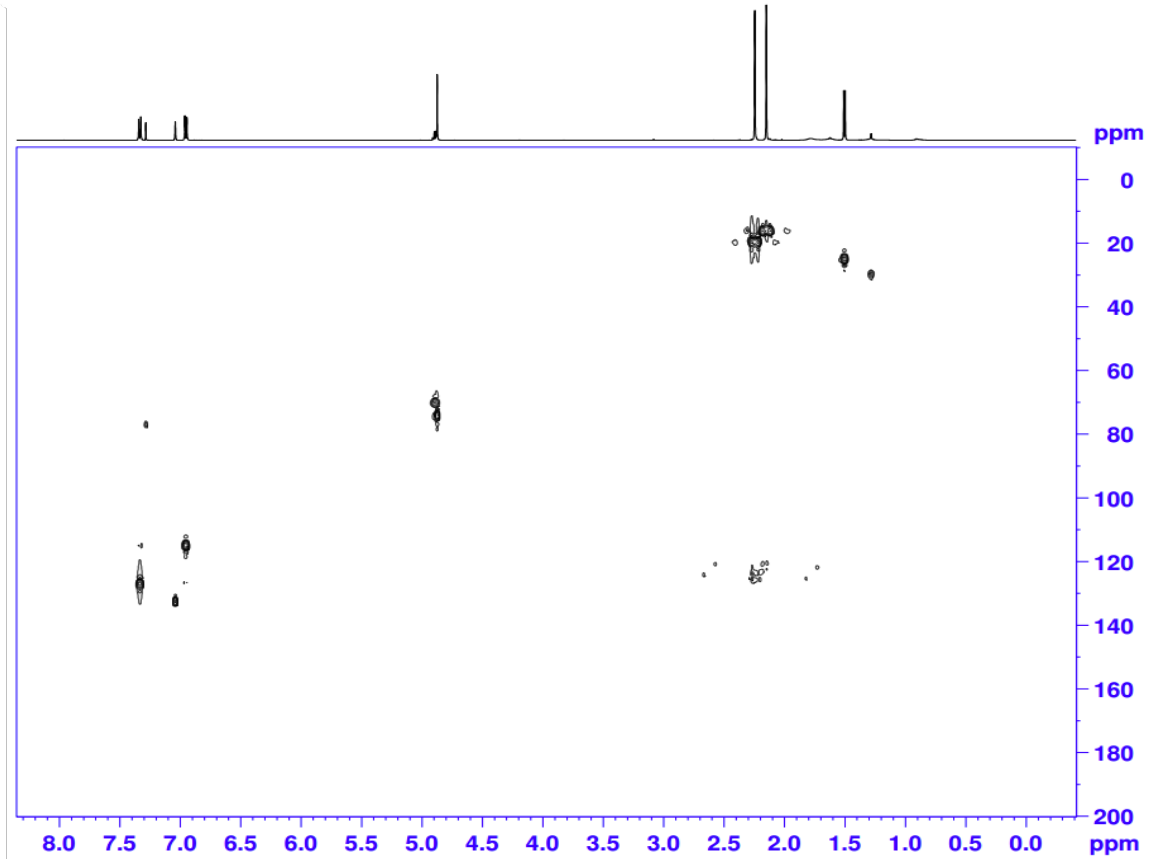

HMBC ($CDCl_3$, 500 MHz) of 2-(4-(1-hydroxyethyl)phenoxy)-1-(2,3,5,6-tetramethylphenyl)ethan-1-one **41b**.

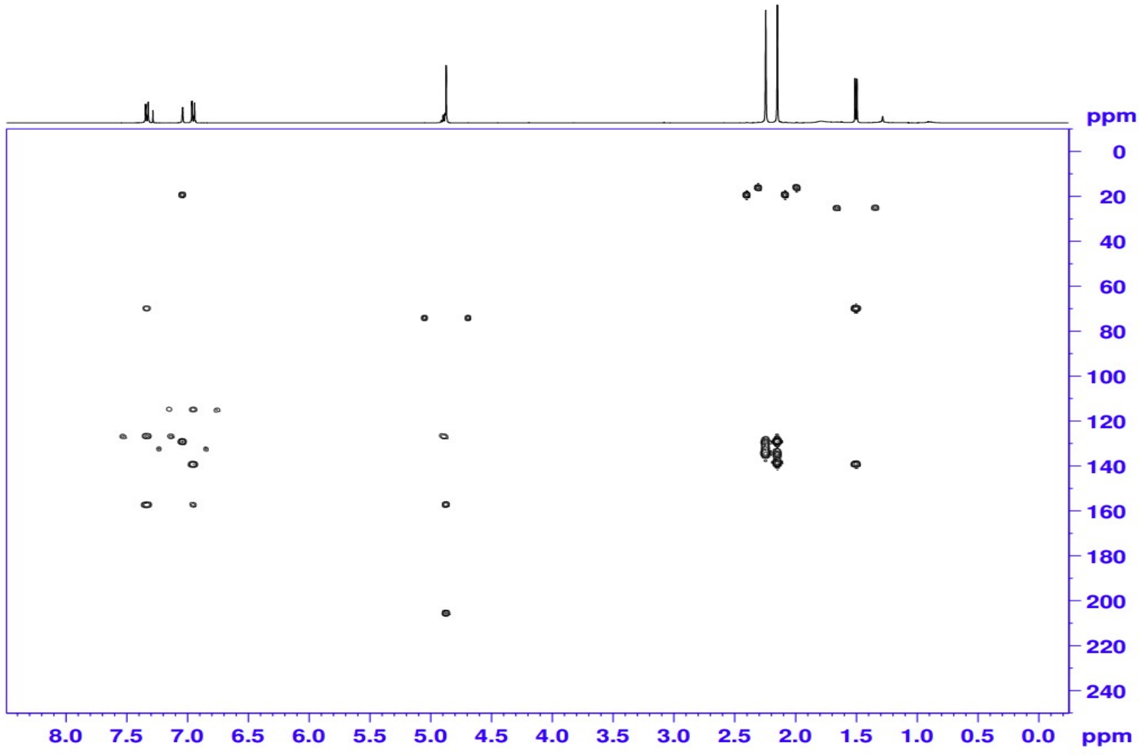

$^{13}C\{^{1}H\}$ NMR ($CDCl_3$, 500 MHz) of 2-(4-(1-hydroxyethyl)phenoxy)-1-(2,3,5,6-tetramethylphenyl)ethan-1-one **41b**.

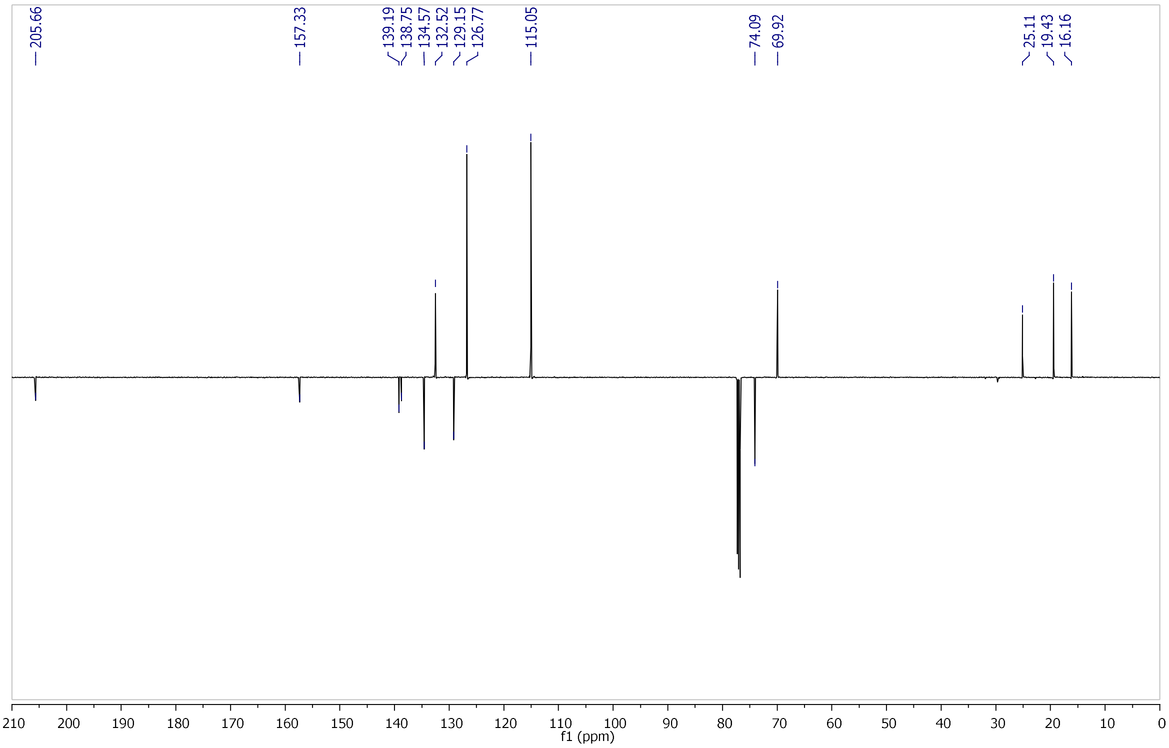

HPLC of (*S*)-2-(4-(1-hydroxyethyl)phenoxy)-1-(2,3,5,6-tetramethylphenyl)ethan-1-one **41b**. (*S,S*)-3C-tethered Ru(II)-TsDPEN catalyst (after 96 h, 100% conversion, 99% ee (*R*))

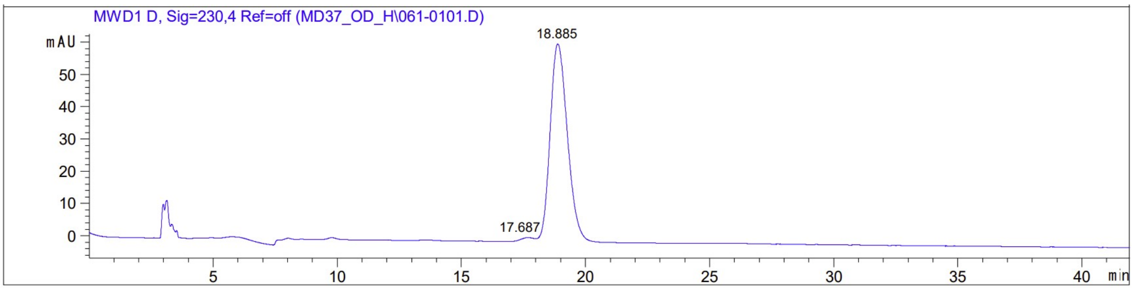

Signal 4: MWD1 D, Sig=230,4 Ref=off

Peak #	RetTime [min]	Type	Width [min]	Area [mAU*s]	Height [mAU]	Area %
1	17.687	MM	0.3520	17.32988	5.95982e-1	0.5788
2	18.885	VB	0.7462	2976.84814	61.33960	99.4212

Totals : 2994.17802 61.93558

Racemic 2-(4-(1-Hydroxyethyl)phenoxy)-1-(2,3,5,6-tetramethylphenyl)ethan-1-one 41b.

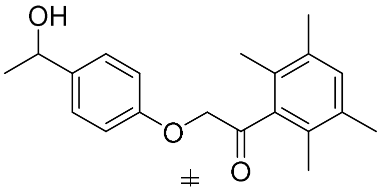

This compound is novel. To a 25 mL round-bottom flask containing 2-(4-acetylphenoxy)-1-(2,3,5,6-tetramethylphenyl)ethan-1-one **41a** (10 mg, 0.030 mmol) dissolved in methanol (0.9 mL) and water (0.1 mL), sodium borohydride (3.4 mg, 0.090 mmol) was added and the mixture was stirred for 96 h. The solvent was removed at the end of this time and EtOAc (20 mL) and water (20 mL) was added. The organic layer was separated and the water layer was extracted with further EtOAc (2 x 20 mL). the combined organic extracts were dried ($MgSO_4$) and filtered and the solvent was removed under vacuum. Crude 2-(4-(1-hydroxyethyl)phenoxy)-1-(2,3,5,6-tetramethylphenyl)ethan-1-one **41b** was afforded as a white solid (6.1 mg, 0.02 mmol, 65%). The ^{1}H NMR spectrum contained signals corresponding to the major enantiomer formed in the asymmetric reductions.

HPLC of racemic 2-(4-(1-hydroxyethyl)phenoxy)-1-(2,3,5,6-tetramethylphenyl)ethan-1-one **41b**.

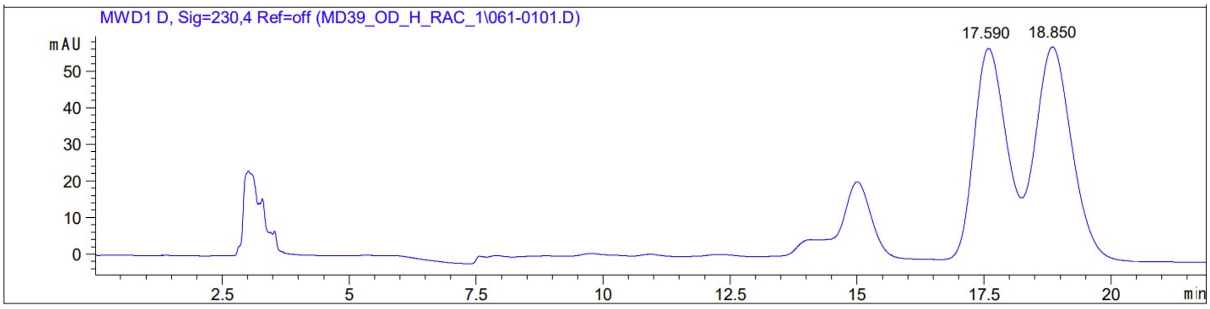

Signal 4: MWD1 D, Sig=230,4 Ref=off

Peak #	RetTime [min]	Type	Width [min]	Area [mAU*s]	Height [mAU]	Area %
1	17.590	BV	0.6809	2552.57324	57.84084	46.4329
2	18.850	VB	0.7618	2944.76392	58.44710	53.5671
Totals :				5497.33716	116.28794	

Summary of unsuccessful reactions.

Figure S1. Attempted synthesis of oxidation products from **8b** or TBS-protected **8b** which did not work.

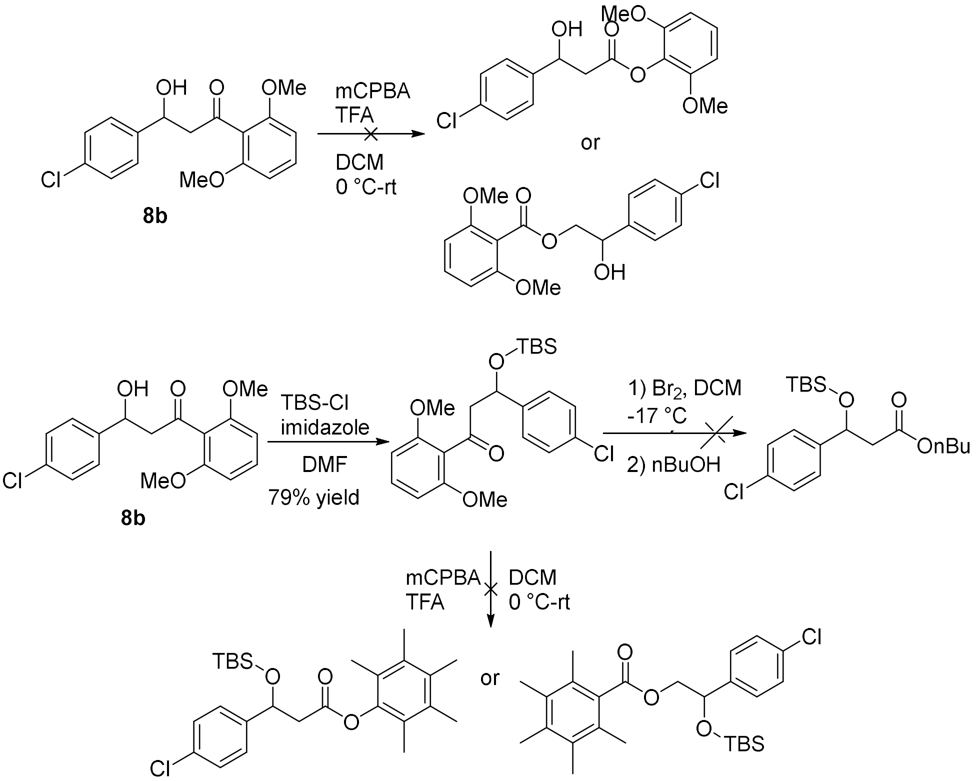

Figure S2. Investigations into the formation of esters from an alcohol product which were unsuccessful. Note that the derivative here is not in the main paper because its ee could not be determined, but details are given in the SI for this compound.

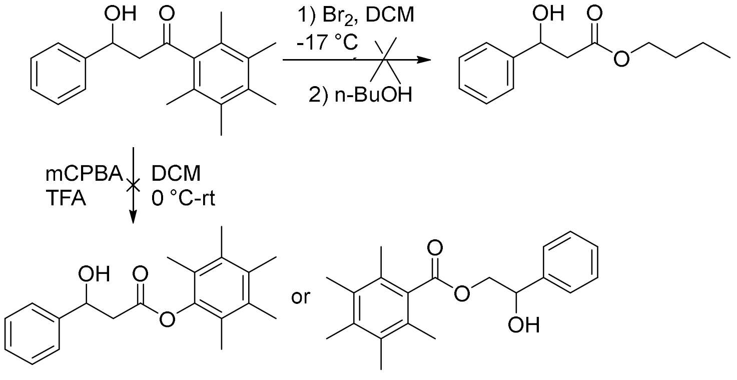

Supplement: Supplementary file 1 — jo3c01950_si_001.pdf [file jo3c01950_si_001.pdf]
